# 39th International Symposium on Intensive Care and Emergency Medicine: Brussels, Belgium, 19-22 March 2019

**DOI:** 10.1186/s13054-019-2358-0

**Published:** 2019-03-19

## Accepted abstracts for 39th International Symposium on Intensive Care and Emergency Medicine

### P001 Prognostic value of a genetic polymorphism of AQP5 in sepsis depends on a source of infection

#### V Pisarev^1^, A Chumachenko^1^, I Tyurin^2^, R Cherpakov^2^, A Tutelyan^3^

##### ^1^Federal Research and Clinical Center of Intensive Care Medicine and Rehabilitology, V.A.Negovsky Institute of General Reanimatology, Moscow, Russia; ^2^V.M.Buiyanov City Clinical Hospital, Anesthesia-Reanimatology Department, Moscow, Russia; ^3^Central Institute of Epidemiology, Moscow, Russia

**Introduction:** The purpose of the study was to determine whether the preferential localization of the infection and age affect the prognostic value of the genetic marker AQP5 (1364A/C, rs3759129) in outcome prediction in sepsis patients. Studies by Adamzik and colleagues have demonstrated that aquaporin AQP5 polymorphism (1364A/C, rs3759129) associates with increased 30-day survival in sepsis patients presumably due to increased gene expression that enhance the leukocyte migration. To increase the informative value of the prediction and decrease the cost, it might be crucial to determine at a pre-test level the subset of patients who might benefit most from the prognostic genotyping.

**Methods:** Sepsis and septic shock were defined in patients according to SEPSIS-3 (2016) recommendations. Study groups (n=152) included ICU patients with abdominal sepsis (AS, including pancreatitits, peritonitis, cholecystitis, appendicitis; n=98) and sepsis patients with other sources of infections. AQP5 polymorphism was studied by analyzing PCR products in a 2% agarose gel using a AQP5 1364A/C specific tetra primer set. Data were analyzed by Kaplan-Meyer plot and Fisher test, and odds ratios were calculated.

**Results:** Distribution of alleles (A and C) and genotypes (AA, CA and CC) AQP5 1364A/C in patients with sepsis or sepsis subgroups (sepsis with no septic shock and sepsis shock patients) versus control group (healthy volunteers) did not differ. Although there was a trend to preferential survival of sepsis patients with genotype C AQP5 despite the source of infection, only patients with AQP5 CC or CA genotype and abdominal sepsis (Sepsis-3), or a subgroup of the same AQP5 genotype experiencing septic shock, demonstrated increased 30-day survival versus AA homozygotic patients (P<0.002).

**Conclusions:** The informative value of detecting the AQP5 CC or CA genotype for prognosis of 30-day survival versus AA homozygotic patients is increased only in abdominal sepsis patients.

### P002 Depressed expression of FCER1A gene is associated with increased mortality in infected surgical patients

#### R Almansa^1^, C Andrés^2^, M Martín-Fernández^3^, S Montero^4^, C Jambrina^5^, C Doncel^6^, J Sánchez-Crespo^5^, M Heredia-Rodríguez^7^, J Rico^4^, C González^8^, E Sánchez-Barrado^5^, M Lorenzo-López^7^, S Martín^4^, L Muñoz-Bellvis^8^, M Vaquero^5^, E Tamayo^7^, C Aldecoa^4^, J Bermejo-Martín^6^

##### ^1^Hospital Clínico Universitario de Valladolid/IECSCYL, BioSepsis (Group of Biomedical Research in Sepsis), Valladolid, Spain; ^2^Hospital Clínico Universitario de Valladolid, Clinical Analysis Service, Valladolid, Spain; ^3^Hospital Clínico Universitario de Valladolid/IECSCYL, BioSepsis (Group for Biomedical Research in Sepsis), Valladolid, Spain; ^4^Hospital Universitario Rio Hortega, Anesthesiology and Reanimation Service, Valladolid, Spain; ^5^Hospital Clínico Universitario de Salamanca, Anesthesiology and Reanimation Service, Salamanca, Spain; ^6^Hospital Clínico Universitario de Valladolid/IECSCYL, BioSepsis (Group for Biomedical Research in Sepsis), Valladolid, Spain; ^7^Hospital Clínico Universitario de Valladolid, Anesthesiology and Reanimation Service, Valladolid, Spain; ^8^Hospital Clínico Universitario de Salamanca, Department of General and Gastrointestinal Surgery, Salamanca, Spain

**Introduction:** Increasing evidence supports a central role for “immunosuppression” in sepsis. It is necessary to develop biomarkers of immune dysfunction that could help to identify patients at risk of poor outcomes [1]. The decreased expression of human leucocyte antigen (HLA)-DRA is proposed as a major feature of immunodepression and its persistent decrease is associated with mortality in sepsis [2]. In a previous study, we evidenced that FCER1A (Fc Fragment Of IgE Receptor Ia) is the gene showing the lowest expression levels of the entire transcriptome in sepsis [3]. Here we studied the association between FCER1A expression and mortality in infected surgical patients.

**Methods:** FCER1A and HLA-DRA expression levels were quantified by droplet digital PCR in blood of 257 infected surgical patients. 26 patients died within 28 days (10.11%). Spearman test was used to evaluate the association between gene expression and the Sequential Organ Failure Assessment (SOFA) score. Areas under Receiver Operating Curves (AUROC) were used to determine the gene expression cut-off values predicting mortality. Kaplan-Meier survival curves were obtained and differences in survival between groups were evaluated using the Log rank test. Cox regression was employed to assess mortality risk at 28 days.

**Results:** Gene expression levels of FCER1A and HLA-DRA correlated inversely with patients’ severity (r: -0.5 p<0.001; r: -0.3, p<0.001 respectively). Both genes showed significant AUROCs to predict survival, but FCER1A showed the best accuracy (Fig. 1). Patients with low levels of FCER1A or HLA-DRA had an increased risk of mortality and died 3 days earlier than non survivors with higher expression levels of these genes (Fig. 2, Table 1-2).

**Conclusions:** Depressed FCER1A gene expression is associated with severity and increased mortality in surgical patients with infection.

**References**

1 Hotchkiss R et al. Lancet Infect Dis 13(3): 260–268, 2013

2 Cazalis MA et al. Crit Care 10;17(6):R287, 2013

3 Almansa R et al. J Infect 70(5):445-56, 2015

**Table 1 (abstract P002).** Predictive capacity of FCER1A gene expression cut-off for 28-day mortality in surgical patients with infection. (COX regression)

	Hazard Ratio	95% CI	P
Age	1.05	(1.00-1.09)	0.038
Diabetes	1.98	(0.85-4.62)	0.112
Respiratory focus	1.63	(0.66-4.01)	0.289
OOP FCER1A: 17.4 copies/ng RNA	4.18	(1.52-11.47)	0.005

**Table 2 (abstract P002).** Predictive capacity of HLA-DRA gene expression cut-off for 28 day mortality in surgical patients with infection. (COX regression)

	Hazard Ratio	95% CI	P
Age	1.05	(1.00-1.10)	0.011
Diabetes	1.90	(0.85-4.31)	0.120
Respiratory focus	2.0	(0.86-4.64)	0.108
OOP HLA-DRA: 2744 copies/ng RNA	2.36	(1.01-5.53)	0.048

**Fig. 1 (abstract P002).**
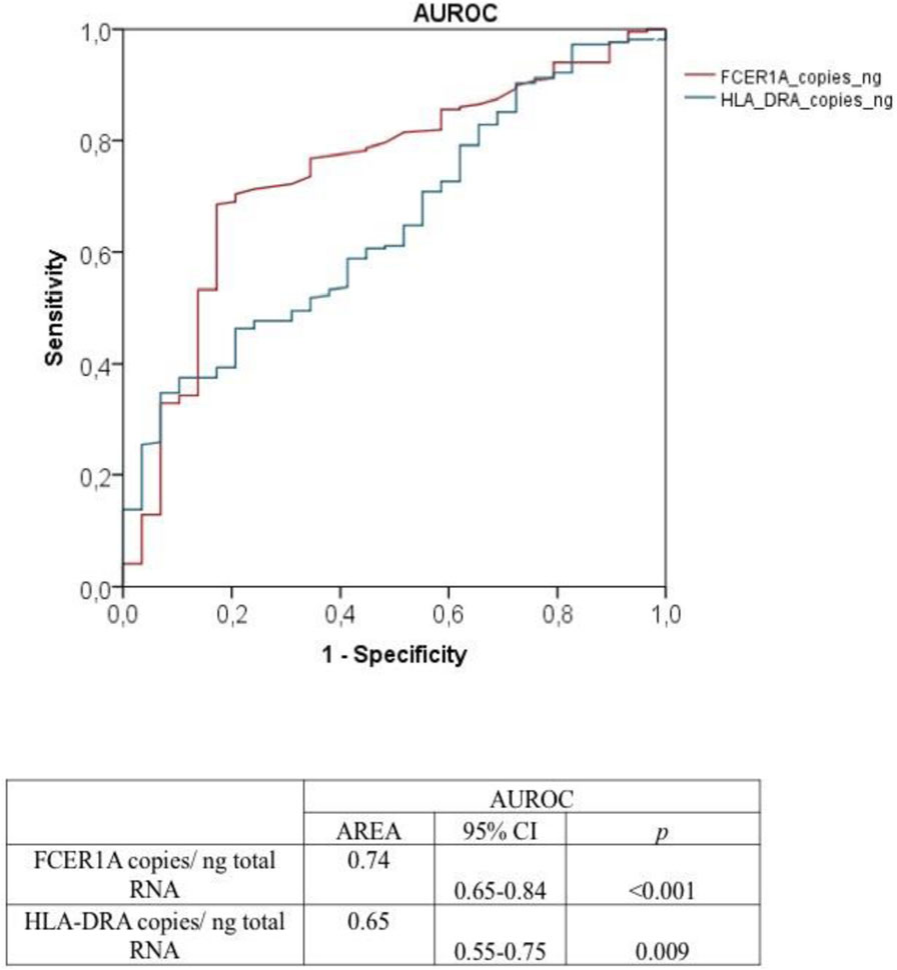
AUROCs for differential diagnosis of mortality in surgical patients with infection

**Fig. 2 (abstract P002).**
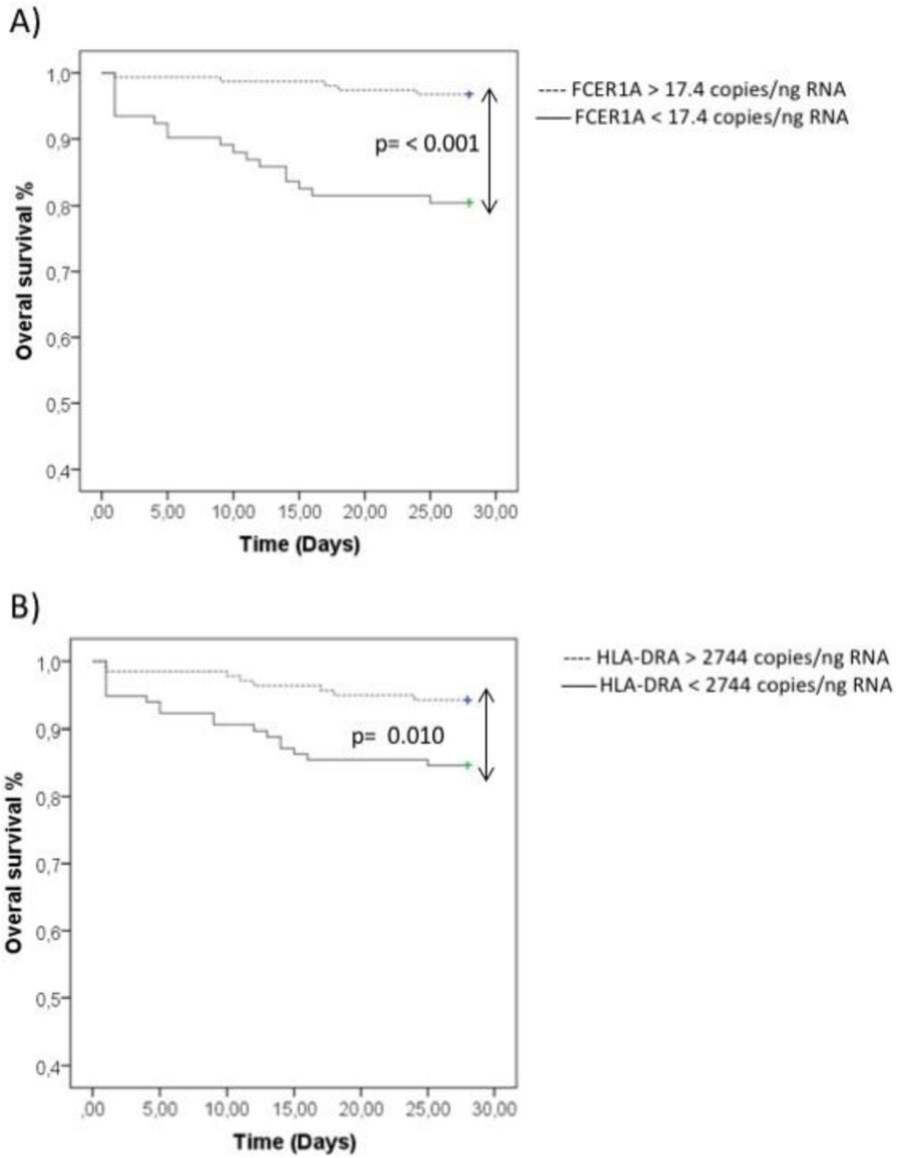
Kaplan-Meier survival curves. Kaplan-Meier survival curves were established after stratification based on calculated thresholds (optimal operating points of FCER1A (A) and HLA-DRA (B) expression levels)

### P003 Genetic markers of nosocomial pneumonia, acute respiratory failure and renal insufficiency in critically ill patients

#### M Khadzhieva^1^, O Belopolskaya^1^, T Smelaya^2^, A Kuzovlev^2^, L Salnikova^3^

##### ^1^N.I. Vavilov Institute of General Genetics, Russian Academy of Sciences, Moscow, Russia; ^2^Federal Research and Clinical Center of Intensive Care Medicine and Rehabilitology, Moscow, Russia; ^3^Federal Research and Clinical Center of Intensive Care Medicine and Rehabilitology, N.I. Vavilov Institute of General Genetics, Russian Academy of Sciences, Moscow, Russia

**Introduction:** Severe pulmonary and renal conditions such as acute respiratory distress syndrome (ARDS), respiratory failure, and deterioration in kidney function often occur in patients with nosocomial pneumonia (NP). The emergence and course of infection is genetically determined, hence host genetic landscape may influence an ability to resist infection.

**Methods:** Variants for genotyping were selected using the PheWAS Catalog which presents genotypic data for 13835 Caucasian patients, 1358 phenotypes and 3144 single nucleotide polymorphisms (SNPs) with P < 0.05 [1]. SNPs with the lowest P-values for phenotypes with both, respiratory and renal manifestations were selected: intergenic variants rs7130588 and rs4980785, rs347344 (EDIL3) and rs2470893 (CYP1A1). CYP1A1 gene was associated with pneumonia and ARDS in our previous investigations, so we included in our analysis three sites of CYP1A1 gene (rs2606345, rs4646903 and rs1048943) studied on a smaller sample. Genotyping was performed on 7 sites for a sample of resuscitation patients with or without NP and other pulmonary complications (n = 354 and n = 216, respectively).

**Results:** Allele rs2606345-G of the CYP1A1 gene was protective against ARDS and an increase in creatinine level (Fig. 1). The rs7130588-G allele was associated with lung complications and with the development of severe respiratory insufficiency (Fig. 2).

**Conclusions:** The SNPs rs2606345 and rs7130588 can influence the aggravation of pulmonary and renal symptoms through genetically mediated response to infection.

**Reference**

1. Denny JC et al. Nat Biotechnol 31: 1102–1110, 2013.

**Fig. 1 (abstract P003).**
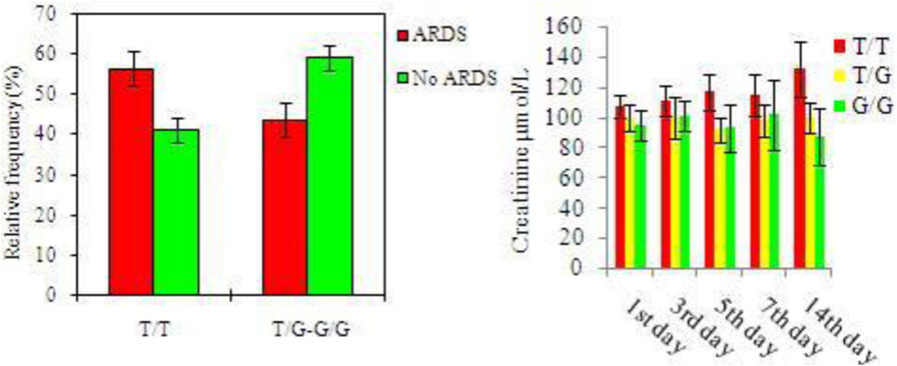
Protective effect of the rs2606345-G allele (CYP1A1 gene) on the risk of ARDS development (left) and an increase of serum creatinine level on the 14th day after hospitalization (right)

**Fig. 2 (abstract P003).**
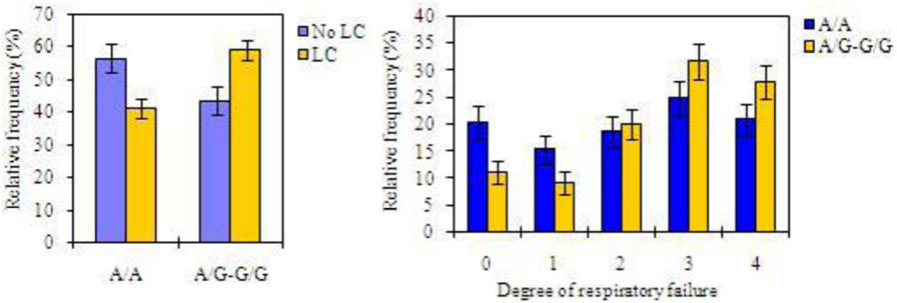
Association of the rs7130588-G allele with lung complications (LC) (NP, ARDS, pleurisy, abscess, etc.) (left) and with the development of severe respiratory failure (right)

### P004 Mesenchymal stem cells regulate LPS–stimulated macrophages polarization balance by paracrine transforming growth factor beta

#### F Liu

##### School of Medicine, Zhongda Hospital, Southeast University, Department of Critical Care Medicine, Nanjing, Jiangsu, China

**Introduction:** An uncontrolled inflammatory response plays a major role in the sepsis related organ dysfunction. Mesenchymal stem cells(MSCs) can improve survival of sepsis experimental models by modulating the inflammatory response. Macrophages have been considered as important immune effector cells and their polarization imbalance aggravates the disordered inflammation reaction. The project aims to identify the effects of MSCs on macrophages polarization against dysregulated inflammatory response.

**Methods:** RAW264.7 cells were plated in the lower chambers of transwell system in the presence or absence of Lipopolysaccharide (LPS). Then, MSCs were seeded in the upper chambers and incubation for different time. Finally, transforming growth factor beta (TGF-β) receptor (TGF-βR) inhibitor was added in transwell system. The phenotype of RAW264.7 cells were analyzed by flow cytometry, the levels of inflammatory cytokines were detected by Enzyme-linked immunosorbent assay (ELISA).

**Results:** Our data showed that LPS increased the level of interleukin (IL)-6 in RAW264.7 cells (p<0.001) (Fig. 1). In line with IL-6 expression, LPS induced the expression of M1 macrophage (p<0.001). Moreover, LPS stimulated RAW264.7 cells co-culture with MSCs in transwell system, MSCs inhibited the expression of IL-6 and M1 macrophages, while increased M2 macrophages (p<0.001). Compared with LPS group, the concentration of TGF-Β was obviously increased in MSCs treatment groups (p<0.001), furthermore, there were no significantly difference between MSCs directed and indicted groups. More significantly, TGF-βR inhibitor abolished the impact of MSCs on LPS stimulated RAW264.7 cells (p<0.001) (Fig. 2).

**Conclusions:** MSCs polarized M1 macrophages into M2 macrophages and decreased pro-inflammatory cytokine levels by paracrining TGF-β.

**Fig. 1 (abstract P004).**
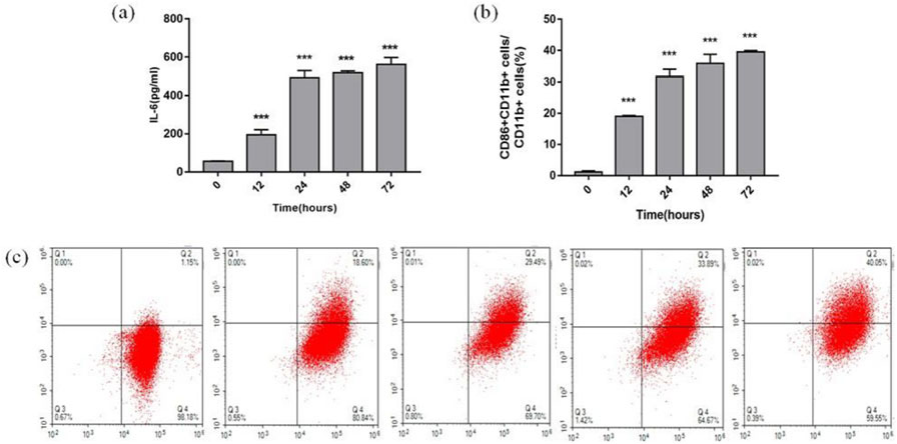
Lipopolysaccharide (LPS) promoted RAW 264.7 M1 polarization. (a): LPS 500 ng/ml stimulated RAW264.7 for 12, 24, 48 and 72 hours, the level of IL-6 significantly increased in a time-dependent manner. (b, c): Flow cytometry showed LPS enhanced the expression of M1 macrophages at all time points. *** P<0.001 compared with control group

**Fig. 2 (abstract P004).**
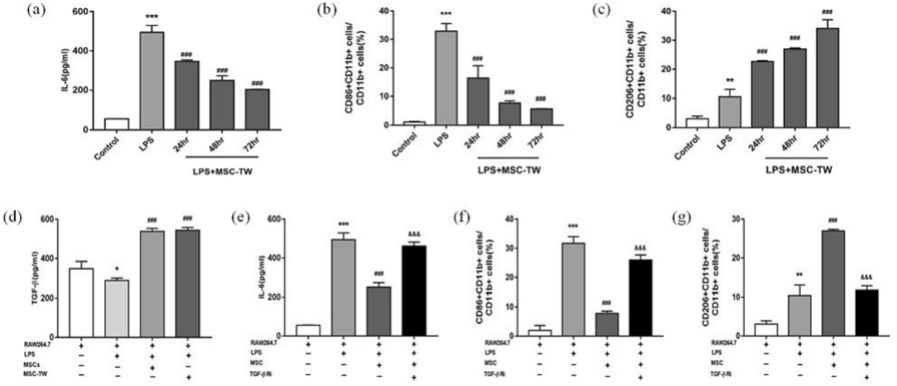
TGF-β secreted by MSCs regulated the M1 to M2 in LPS-Stimulated RAW264.7. LPS stimulated RAW264.7 co-culture with MSCs in transwell system for 24, 48 and 72 hours. (a): LPS increased the level of IL-6, whereas MSCs inhibited expression of IL-6. (b, c): MSCs reduced M1 macrophages while increased M2 macrophages in LPS stimulated RAW264.7. (d): The concentration of TGF-β was obviously increased in MSCs directed or in-directed group. (e, f, g): LPS increased IL-6 and M1 macrophages, and MSCs inhibited the IL-6 and M1 macrophages while increased M2 macrophages. TGF-βR inhibitor reversed the effect of MSCs on LPS-stimulated RAW264.7. *P<0.05, **P<0.01, *** P<0.001 compared with control group. # # # P<0.001 compared with LPS stimulated group. &&& P<0.001 compared with MSCs treatment group

### P005 Chronomics in ICU: effect of timing of septic shock onset on circadian rhythm profiles of melatonin and cortisol

#### E Sertaridou^1^, I Chouvarda^2^, K Arvanitidis^3^, E Filidou^4^, G Kolios^3^, I Pnevmatikos^1^

##### ^1^University Hospital of Alexandroupolis, Intensive Care Unit, Alexandroupolis, Greece; ^2^Aristotle University of Thessaloniki, Faculty of Medicine, Thessaloniki, Greece; ^3^Democritus University of Thrace, Faculty of Medicine, Alexandroupolis, Greece; ^4^Democritus University of Thrace, Faculty of Mrdicine, Alexandroupolis, Greece

**Introduction:** Circadian rhythmicity of melatonin and cortisol has been found to be affected by sepsis in both experimental and clinical studies.

**Methods:** In this study, we evaluated the potential effect of septic shock on circadian rhythms of urinary excreted aMT6s, a melatonin’s metabolite and cortisol in 26 patients, divided into two groups: Group A (N=15) included subjects with septic shock upon admission to the ICU and Group B (N= 11) included patients who developed septic shock during ICU stay. Urine samples were collected every 4 h over a 24-h period, whereas data were available during entry and before discharge from the ICU in Group A and during entry, septic shock and before exit from the ICU in Group B. Circadian analysis was performed leading to the estimation of mesor (mean value), amplitude of the oscillation and acrophase (phase shift of maximum values in hours).

**Results:** Circadian markers of aMT6s and cortisol exhibited inverse changes, both within and between groups, since amplitude of aMT6s was reduced in entry in relation to exit (437.2±309.2 vs 674.1±657.6 ng/4h, p<0.05), whereas amplitude of cortisol was increased upon admission compared to exit (13.3±31 ng/4h vs 8.7±21.2 ng/4h p<0.05), in Group A. Furthermore, in Group B, mesor of aMT6s was increased during septic shock (2492.2± 1709.1 ng/4h) compared to both entry (895.4±715.5 ng/4h) and exit (1308.6± 1214.4 ng/4h, p<0.05 for all comparisons). However, cortisol’s mean values were reduced during septic shock (10±5.3 ng/4h) compared to both entry (30±57.9 ng/4h) and exit (14.4±20.7 ng/4h, p<0.05 for all comparisons) and correlated with higher APACHE II score and longer ICU and hospital stay (p<0.05 for all comparisons).

**Conclusions:** Septic shock induced inverse changes of aMT6s and cortisol circadian rhythm profiles, depending on timing of onset.

### P006 Investigation of the relationship between organ damage, microcirculatory dysfunction and reactive oxygen intermediate formation in experimental sepsis

#### J Kaszaki, A Rutai, R Fejes, S Tallósy, M Poles, D Érces, M Boros, A Szabó

##### Universitiy of Szeged, Institute of Surgical Research, Szeged, Hungary

**Introduction:** Sepsis is dysregulated response to an infection, which can lead to progressive microcirculatory dysfunction, release of reactive oxygen intermediates (ROI) and life-threatening organ dysfunction. Our aim was to investigate the relationship between organ damage - characterized by the Sequential Organ Failure Assessment (SOFA) scores, microcirculatory failure and ROI production, in a large animal model of experimental sepsis.

**Methods:** Fecal peritonitis was induced in anesthetized minipigs (n=28; 0.5g/kg autfeces containing 5-9 x10^6^ CFU bacteria i.p.), control animals (n=9) received sterile saline i.p. Invasive hemodynamic monitoring and blood gas analyses were performed between 16-24 hrs, the signs for failure of circulatory, respiratory and urinary systems were evaluated in accordance with the SOFA score. The microcirculatory perfusion rate in the sublingual region was measured by orthogonal polarization spectral imaging technique (Cytoscan A/R). The leukocyte-origin ROI production was determined by lucigenine (mostly O_2_^-.^) and luminol-based (H_2_O_2_) chemiluminescence methods.

**Results:** Between 16-24 hrs after induction the SOFA score indicated moderate organ failure in 19 animals (M: 1.9; 25p: 1.5, 75p: 2.9) and the change was statistically significantly higher in 9 pigs, suggesting severe organ dysfunction (M: 4.1; 25p: 3.5, 75p: 5.2). The microcirculation was significantly deteriorated in all cases, independently of SOFA score data. The H_2_O_2_ production was significantly lower in septic animals as compared to controls, while the lucigenine enhanced ROI production correlated with the SOFA score-indicated moderate and severe organ dysfunction.

**Conclusions:** Sublingual microcirculatory parameters are not correlating with the severity of SOFA score-indicated organ dysfunction in abdominal sepsis. The measurement of ROI production of the whole blood seems to be better biomarker for the detection of the progression of events from moderate to severe organ damages.

Grant supports: NKFIH K116689; EFOP-3.6.2-16-2017-00006

### P007 Increased rate of mechanical ventilation in septic patients with left ventricular dysfunction

#### A Newsome, S Smith, T Jones

##### The University of Georgia College of Pharmacy, Pharmacy, North Augusta, United States

**Introduction:** The purpose of this study was to characterize differences in sepsis management in patients with and without left ventricular (LV) dysfunction. Septic patients with LV dysfunction have higher mortality, and limited guidance exists for sepsis management of patients with LV dysfunction. The possibility exists that the cornerstones of sepsis management may contribute to these poor outcomes.

**Methods:** A retrospective chart review was conducted from May 2016 - January 2018 at two centers. Adult patients who had a diagnosis of sepsis, were treated with vasopressors for > 3 hours, and had an echocardiogram within 12 months were included. Patients were divided into two groups: reduced ejection fraction (EF) of < 40% and preserved EF defined as EF ≥40%. Information about patient outcomes and sepsis management were collected. The primary outcome was the need for mechanical ventilation (MV). Categorical and continuous data were analyzed using the Chi-Squared and Mann-Whitney U tests, respectively. The IRB has approved this project.

**Results:** A total of 37 patients with EF < 40% and 42 patients with EF ≥40% were included. No significant differences in fluid management, vasoactive agent maximum rate or duration, or steroid use were observed. Net fluid balance between low and preserved EF was positive 4.6 liters vs. 5.1 liters (p = 0.814), respectively. The number of patients that needed MV was higher in the low EF cohort (86% vs. 57%, p = 0.004), and this cohort had fewer MV-free days (20, IQR 0-25 vs. 24 (IQR 0 -28), p=0.064.

**Conclusions:** No significant differences were observed with regard to sepsis management, reflecting current guidelines. The significantly increased need for MV is a provocative result. A potential mechanism is the inability of a patient with reduced LV dysfunction to maintain appropriate cardiac and respiratory function in the face of fluid overload. Prospective analysis of the role of fluid balance in septic patients with LV dysfunction is warranted.

### P008 Biomarkers of myocardial injury and cytokine plasma levels in septic patients

#### M Assuncao^1^, FR Machado^2^, MK Brunialti^3^, O Rigato^4^, R Salomao^3^

##### ^1^Hospital Israelita Albert Einstein, Department of Critical Care, Sao Paulo, Brazil; ^2^Federal University of Sao Paulo, Department of Anesthesiology, Pain and Intensive Care, Sao Paulo, Brazil; ^3^Federal University of Sao Paulo, Division of Infections Diseases, Sao Paulo, Brazil; ^4^Hospital Sirio Libanes, Department of Critical Care, Sao Paulo, Brazil

**Introduction:** The relationship between myocardial injury and systemic inflammation in sepsis response is not well understood [1]. It´s proposed to evaluate the association between myocardial injury biomarkers, high-sensitive troponin T (hs-cTnT) and N-terminal pro-brain natriuretic peptide (NT-ProBNP), with inflammatory mediators (IL-6, IL-1Β , IL-8, IL-10, IL-12 / IL-23p40, IL17A, IL- 21 and TNF-α ) and biomarkers, C protein reactive (CPR) and procalcitonin (PCT), in septic patients

**Methods:** This was a prospective cohort study performed in three intensive care units, from September 2007 to September 2010 enrolling patients with sepsis (infection associated with organ dysfunction), and septic shock (hypotension refractory by fluids infusion requiring vasopressor). Blood samples were collected up to 48h after the development of first organ dysfunction (D0) and on the 7th day after inclusion in the study (D7)

**Results:** Ninety-five patients were enrolled, with median age 64 years (interquatile?48–78), APACHE II: median 19 (14-22), SOFA: median 8 (5-10); 24.2% were admitted in ICU with sepsis and 75.8% with septic shock. Hospital mortality was 34.7%. In D0, NT-ProBNP correlated with IL-8 (r = 0.495, p <0.001) and IL-10 (r = 0.471, p <0.001). In D7, hs-cTnT and NT-ProBNP correlated with PCT (r = 0.446, p < 0.001 and r = 0.495, p < 0.001; respectively). NT-ProBNP D0 was higher in non-survivors than in survivors on mortality in seventh day (p = 0.029) and in-hospital mortality (p = 0.030). hs-cTnT D7 (p = 0.030) and NT-ProBNP D7 (p <0.001) were significantly higher in non-survivors on in-hospital mortality. NT-ProBNP D7 (OR 9.28; IC95% 2.05-41.94, p=0,004) and hs-cTnT D7 (OR 10,93; IC95% 2.139 – 55.795, p=0,04) were independently associated with in-hospital mortality

**Conclusions:** NT-ProBNP plasma levels at D0 correlated with IL-8 and IL-10, and both NT-ProBNP and hs-cTnT at D7 correlated with PCT. In addition, NT-ProBNP has been shown to be an important predictor of mortality

**Reference**

1. Landesberg G et al. Chest. 2015;148:93-102.

### P009 Repeated measures of heparin-binding protein correlate with mean arterial pressure and systemic vascular resistance index in septic shock: a pilot study on biomarker kinetics from a Swedish intensive care unit

#### J Tverring^1^, N Nielsen^2^, F Kahn^3^, A Linder^3^, P Åkesson^3^

##### ^1^Division of Infection Medicine, BMC, B14, Faculty of Medicine, Department of Clinical Sciences, Lund, Sweden; ^2^Division of Anesthesiology and Intensive Care, Department of Clinical Sciences, Lund, Sweden; ^3^Division of Infection Medicine, BMC, B14, Lund, Sweden

**Introduction:** Heparin-binding protein (HBP) acts proinflammatory on immune cells and induces vascular leakage through cytoskeletal rearrangement and cell contraction in the endothelium and is a promising novel prognostic biomarker in sepsis and septic shock. However, studies on repeated measures of HBP are lacking. Our objective was to describe the kinetics of plasma HBP during septic shock and correlate it to hemodynamic parameters.

**Methods:** We included patients with septic shock (sepsis-3) on admission to Helsingborg hospital’s intensive care unit (ICU) during September 2016 to February 2018. Patients were sampled from ICU admission and every 4 hours for 72 hours or until death or ICU discharge. The plasma samples were analyzed for HBP and converted using the natural log (lnHBP) for normality. lnHBP was then evaluated against mean arterial pressure (MAP) as primary analysis and against systemic vascular resistance index (SVRI) as a secondary analysis, using mixed-effects linear regression models, treating patient id as a random intercept and adjusting for hemodynamic parameters.

**Results:** A total of 22 patients were included with median age 67 years, 9 females (41%), 7 surgical admissions (32%), median SOFA-score 12 points on day one and 6 deaths from all causes within 90 days (27%). Plasma HBP ranged from 0 to 932 ng/ml with a median of 47 ng/ml (lnHBP range 1.6 to 6.8, median: 3.9). An increase lnHBP was significantly associated with a decrease in MAP (Coef. -2.58 mmHg, 95% CI: -0.62 to -4.55, p=0.010, n=22), when adjusting for heart rate (HR), noradrenaline (NA), vasopressin (VP), dobutamine (DBT) and levosimendan (LS). In a secondary subgroup analysis, an increase in lnHBP was also significantly associated with a decrease in SVRI (Coef. -94.2 dyne*s*cm-5*m-2, 95% CI: -1.3 to -187.1, p=0.047, n=13), when adjusting for MAP, HR, NA, VP, DBT, LS and cardiac index.

**Conclusions:** Repeated measures of plasma HBP during septic shock were correlated with important hemodynamic parameters in this small pilot study.

### P010 Mid-regional pro-adrenomedullin (MR-proADM) as early mortality predictor in septic shock

#### V Lovati, F Marsigli, E Pierucci

##### Azienda Ospedaliero Universitaria Policlinico Sant´Orsola Malpighi, Dipartimento di Scienze Mediche e Chirurgiche, Bologna, Italy

**Introduction:** Mid-regional pro-Adrenomedullin (MR-proADM) comes from the synthesis of the hormone adrenomedullin (ADM), which is overexpressed during inflammation and progression from sepsis to septic shock. Thus, MR-proADM can be a useful biomarker for the clinical management of septic patients [1]. The aim of our study was to understand the ability of MR-proADM to predict 30-day (30-d) mortality and to find a correlation between MR-proADM and Sequential Organ Failure Assessment (SOFA) score in the first 24 hours from Intensive Care Unit (ICU) admission.

**Methods:** We evaluated 28 consecutive septic shock patients according to 2016 Sepsis III definitions. Clinical data from the medical records included demographics, comorbidities, laboratories, microbiology and biomarker levels. Whole blood samples for biomarker profiling were collected at 24, 72 and 120 hours from ICU admission. MR-proADM measurement was detected in EDTA plasma using a sandwich immunoassay by TRACE® (Time Resolved Amplified Cryptate Emission) technology (Kryptor Thermo Fischer Scientific BRAHMS).

**Results:** Overall 30-d mortality rate was 50.0%. MR-proADM [odds ratio (OR) = 1.195], SOFA score (OR = 2.174) and Lactate (Lac) levels (OR = 1.956) in the first 24 hours were associated with 30-d mortality in univariate logistic analysis (P value < 0.05, Table 1). 30-d mortality rate was not associated with procalcitonin (PCT) levels (OR = 1.002). Further linear regression analysis showed significant correlation between MR-proADM and SOFA score at 24 hours from ICU admission (P value<0.001, Fig. 1, Table 2).

**Conclusions:** MR-proADM demonstrated superior accuracy to predict 30-d mortality compared to PCT levels and is directly linked to SOFA score at 24 hours from admission. MR-proADM may aid early identification of poor prognosis septic patients who could benefit a more intensive management.

**Reference**

1. Andaluz-Ojeda D. et al. Ann Intensive Care 7, 15 (2017)

**Table 1 (abstract P010).** Univariate logistic analysis between 30-day mortality, MR-proADM, SOFA score, lactate levels, PCT levels

	Odds Ratio	Standard Error	P value	95% Confidence Intervals
MR-proADM	1.195	0.102	0.037	1.011 - 1.413
SOFA score	2.174	0.693	0.015	1.164 - 4.061
Lactate	1.956	0.652	0.044	1.018 - 3.760
PCT	1.002	0.004	0.680	0.994 - 1.010

**Table 2 (abstract P010).** Linear regression analysis between MR-proADM and SOFA score related to Fig. 1

Coeff.	Standard Error	P value	95% Confidence Intervals
0.321	0.073	<0.001	0.170 - 0.472

**Fig. 1 (abstract P010).**
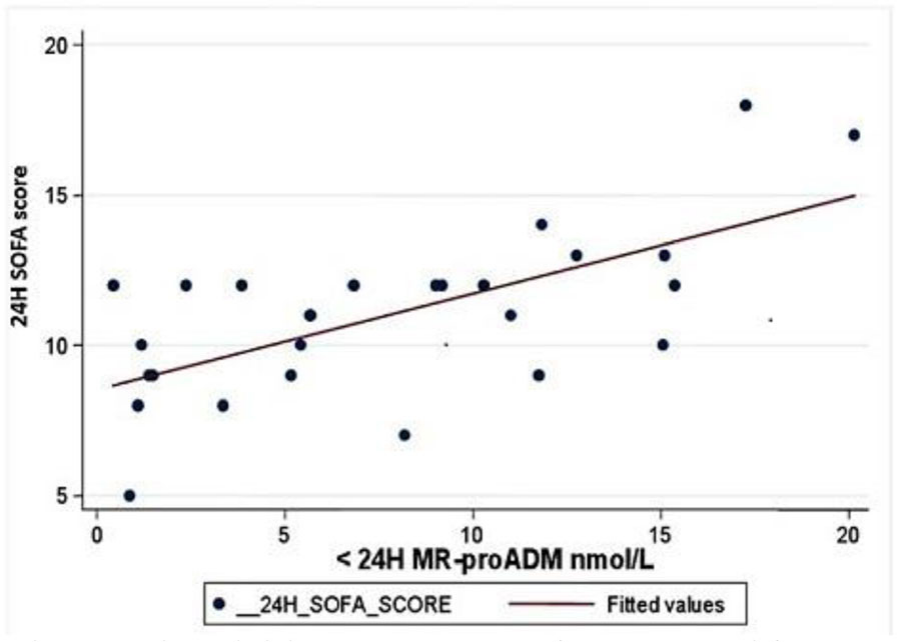
Linear regression analysis between MR-proADM and SOFA score at 24h from ICU admission: SOFA score = Coeff. x MR-proADM + Const. Coeff. = 0.3211 L/nmol Const. = 8.5158

### P011 Pre-sepsin as diagnostic and prognostic marker in sepsis: prospective evaluation by test and confirmation cohorts

#### N Melachroinopoulos^1^, S Pouriki^2^, A Prekates^3^, K Toutouzas^4^, C Mathas^5^, E Giamarellos-Bourboulis^6^

##### ^1^National and Kapodistrian University of Athens, Athens, Greece; ^2^Hippokrateion General Hospital, Athens, Greece; ^3^Tzaneion General Hospital, Intensive Care Unit, Piraeus, Greece; ^4^National and Kapodistrian University of Athens, 1st Department of Propedeutic Surgery, Athens, Greece; ^5^Konstantopouleion General Hospital, Intensive Care Unit, Athens, Greece; ^6^National and Kapodistrian University of Athens, 4th Department of Internal Medicine, Athens, Greece

**Introduction:** Biomarkers have not yet been studied in prospective studies using Sepsis-3. The diagnostic and prognostic validity of pre-sepsin (soluble CD14) was studied in both one test and one confirmation cohort.

**Methods:** The test cohort was the prospective clinical study INTELLIGENCE-1 (ClinicalTrials.gov NCT03306186) enrolling patients with documented infections and at least one qSOFA sign. The confirmation cohort was the prospective clinical study INTELLIGENCE-2 (ClinicalTrials.gov NCT03306186) with patients admitted in the emergencies with at least one qSOFA sign. Blood samples were collected within the first 24 hours of the presence of the qSOFA criteria and pre-sepsin was measured in plasma using the PATHFAST assay. Patients were classified as sepsis and non-sepsis using Sepsis-3 definitions; 28-day mortality was recorded.

**Results:** In the test cohort, 62 patients were classified as non-sepsis and 111 as sepsis. Using ROC curve analysis, it was found that the best trade-off between sensitivity and specificity was provided at 350 pg/ml. The odds ratio for sepsis with presepsin above 350 pg/ml was 4.04 (p<0.0001) providing diagnostic sensitivity 80.2%. In logistic regression analysis, it was found that Charlson’s comorbidity index more than 2, history of type 2 diabetes mellitus and of chronic obstructive pulmonary disease and presepsin more than 350 pg/ml were the only variables independently associated with sepsis. Presepsin above 350 pg/ml was associated with sensitivity 91.5% for 28-day mortality. The odds ratio for mortality with presepsin above 350 pg/ml was 6.84 (p: 0.001). In the confirmation cohort, 59 patients were enrolled. The sensitivity of presepsin above 350 pg/ml for the diagnosis of sepsis was 85.7% and for the prediction of 28-day mortality 100%.

**Conclusions:** Using a test and confirmation cohort approach, presepsin above 350 pg/ml was proved a valuable indicator for the diagnosis of sepsis and outcome prognosis among the most severe patients with one qSOFA sign.

### P012 Toll-like receptors as biomarkers of sepsis in the emergency department

#### C Graham, LY Leung, R Lo, YK Leung, K Hung

##### The Chinese University of Hong Kong, Accident and Emergency Medicine Academic Uint, Hong Kong, China

**Introduction:** We aimed to investigate circulating TLRs gene signatures in Emergency Department (ED) patients at high risk of developing sepsis. Sepsis is “life-threatening organ dysfunction due to dysregulated host responses to infection”. Toll-like receptors (TLRs) are proteins that play a key role in the immune system’s response to infection. Thus, TLRs may act as early markers to identify patients at high risk of sepsis.

**Methods:** This is single-center, prospective study conducted in the ED of Prince of Wales Hospital, Hong Kong (July-September 2017). Patients presented with suspected infection were recruited. Blood samples were collected and buffy coat TLR mRNA levels were measured by real-time polymerase chain reaction (PCR). Beta-2-microglobulin (B2M) was used as a control gene.

**Results:** Among 67 patients recruited (median age 69 years, IQR: 56-84; 46.3% male), we analyzed TLR gene signatures in 21 infection patients and 13 sepsis patients. We recruited 10 gout patients and 10 healthy controls (HC). Median buffy coat TLR-3 mRNA levels were lower in sepsis patients compared with infection, gout and HC groups (0.26 vs 1.67 vs 1.15 vs 1.25 ng/ng B2M, p<0.05). Higher TLR-7 levels were found in infection patients than in the gout and HC groups (0.46 vs 0.28 vs 0.30 ng/ng B2M, p<0.05), whereas lower TLR-9 levels were found in sepsis than infection and HC groups (0.015 vs 0.034 vs 0.025 ng/ng B2M, p<0.05). Receiver operator curve analysis of TLR-3, -7 & -9 for discriminating sepsis and non-sepsis patients, the areas under the curve (AUC) were 0.82, 0.61 and 0.68 respectively. The combination of TLR-3, -7 and -9 demonstrated the largest AUC: 0.94.

**Conclusions:** TLRs mRNA signatures in buffy coat vary among different pathological conditions and have the potential to be an early marker to identify patients at high risk of development of sepsis. Combinations of TLR-3, -7 and -9 could further improve the diagnostic potential of the prediction of sepsis development.

### P013 Evaluation of cell-free DNA (cfDNA) as predictor of mortality and severity in hospitalized septic patients

#### L Maia, P Frizera Vassallo, R Caldeira Machado Berger, V Garrone Barauna, V M. Curty, D Zaniqueli

##### UFES, Physiology, Vitória, Brazil

**Introduction:** Study of the expression of cell free DNA (cfDNA) in the search for new biomarkers for infection, sepsis and septic shock.

**Methods:** The population studied was all patients included in the sepsis protocol from March 2017 to January 2018, hospitalized patients of a federal public hospital. Plasma samples were collected for quantification of cfDNA, which after centrifugation were stored at -80 ° C and then thawed and analyzed by fluorescence using a Varioskan Flash fluorometer). CfDNA values were expressed as ng/mL. The patients were divided into 2 groups: Infection and sepsis/septic shock. We analyzed mortality, Sequential Organ Failure Assessment Score (SOFA score), qSOFA (quick SOFA), comorbidities, cfDNA and laboratory parameters of 111 patients.

**Results:** Among the 111 patients, 28% were classified as infection and 72% sepsis/septic shock. Overall lethality was 33%, infection 9.7%, and sepsis/septic shock 42.5% (p<0.001). The mean of cfDNA, SOFA and lactate was higher according to the classification of infection and sepsis/septic shock: CfDNA (159.4±117.3 and 282.7±358.6, p=0.006), SOFA (1.9±2.1 and 6.6±4.3, p<0.001), QSOFA (positive in 25% and 75%, lactate (1.6±0.8 and 3.8±3.5, p<0.001). We analyzed leukocytes, creatinine, CRP (C reactive protein), INR (International Normalized Ratio), as predictors of severity and only CRP showed no association with disease severity (P=0.84). Levels of cfDNA and qSOFA showed worse prognostic utility as a predictor of sepsis / septic shock when compared to lactate and SOFA: OR 1.00 (95% CI 0.41-2.45), p=0.98 for cfDNA, OR 2.4 (95% CI 1.37-4.21), p=0.002 for SOFA and OR 2.00 (95% CI 0.94-4.28), p=0.072 for lactate. Negelkerke R Square was 0,633 for cfDNA. In addition, area under the curve for cfDNA mortality was 0.60 (95% CI 0.46-0.73) and SOFA 0.81 CI 95% 0.19-0.91).

**Conclusions:** Our study suggests that cfDNA and qSOFA have worse prognostic accuracy when compared to lactate and SOFA, variables already used in clinical practice and easily measured.

### P014 LCN2 expression correlates with organ failure in surgical patients with infection

#### M Martín-fernández^1^, R Almansa^1^, S Montero^2^, J Almeida Cristo-Barbosa^3^, A Ortega^1^, A Hernández Valero^3^, E Gómez Sánchez^4^, E Gómez Pesquera^4^, J Rico-Feijoo^2^, MC Esteban-Velasco^5^, JM Calvo-Vecino^3^, M Vaquero^3^, C Aldecoa^2^, E Tamayo^4^, J Bermejo-Martín^1^

##### ^1^Hospital Clínico Universitario de Valladolid/IECSCYL, BioSepsis (Group for Biomedical Research in Sepsis), Valladolid, Spain; ^2^Hospital Universitario Río Hortega, Anesthesiology and Reanimation Service, Valladolid, Spain; ^3^Hospital Universitario de Salamanca, Anesthesiology and Reanimation Service, Salamanca, Spain; ^4^Hospital Clínico Universitario de Valladolid, Anesthesiology and Reanimation Service, Valladolid, Spain; ^5^Hospital Universitario de Salamanca, Department of General and Gastrointestinal Surgery, Salamanca, Spain

**Introduction:** The aim of this study is to develop a “molecular equivalent” to Sequential Organ Failure Assessment (SOFA) score, which could identify organ failure in an easier, faster and more objective manner, based on the evaluation of Lipocalin-2 (LCN2/NGAL) expression levels by using droplet digital PCR (ddPCR). Sepsis has been classically defined as the exuberant, harmful, pro-inflammatory response to infection. This concept is changing [1] and the presence of a life-threatening organ dysfunction caused by a dysregulated host response to infection is now considered a central event in the pathogenesis of sepsis [2].

**Methods:** LCN2 expression levels were quantified by ddPCR in blood of a total of 257 surgical patients with a diagnosis of infection. Spearman analysis was used to evaluate if LCN2 correlated in a significant manner with SOFA score. Area under the receiver operating curve (AUROC) analysis and multivariate regression analysis were employed to test the ability of LCN2 to identify organ failure and mortality risk.

**Results:** Spearman analysis showed that there was a positive, significant correlation between LCN2 expression levels and SOFA score (Fig. 1). AUROCs analysis showed that LCN2 presents a good diagnostic accuracy to detect organ failure and mortality risk (Fig 2). In the multivariate regression analysis, patients showing LCN2 expression levels over the Optimal Operating Points (OOPs) identified in the AUROCs showed a higher risk of developing organ failure (Table 1) and a higher mortality risk (Table 2).

**Conclusions:** Quantifying LCN2 expression levels by ddPCR is a promising approach to improve organ failure detection and mortality risk in surgical patients with infection.

**References**

1. Bermejo-Martin JF et al. J Infect 72: 525–536, 2016.

2. Singer M et al. JAMA 315: 801–810, 2016.

**Table 1 (abstract P014).** Multivariate analysis for evaluating the risk of organ failure based on the LCN2 expression levels. Adjusting variables were [age], [chronic cardiac disease], [cancer], [immunosuppression], [hypertension], [chronic respiratory disease], [chronic renal failure], [respiratory focus], [abdomen focus]

	OR	Multivariate analysis	p
		[CI 95%]	
LCN2 (copies/ng) Ln	2.20	[1.61-3.02]	< 0.001
LCN2 OOP (638 copies/ng)	11.13	[4.59-26.96]	< 0.001

**Table 2 (abstract P014).** Multivariate analysis for evaluating the risk of mortality based on the LCN2 expression levels. Adjusting variables were [age], [chronic renal failure], [diabetes], [respiratory focus]

	OR	Multivariate analysis	p
		[CI 95%]	
LCN2 (copies/ng) Ln	1.85	[1.36-2.53]	< 0.001
LCN2 OOP (3458 copies/ng)	9.03	[3.23-25.21]	< 0.001

**Fig. 1 (abstract P014).**
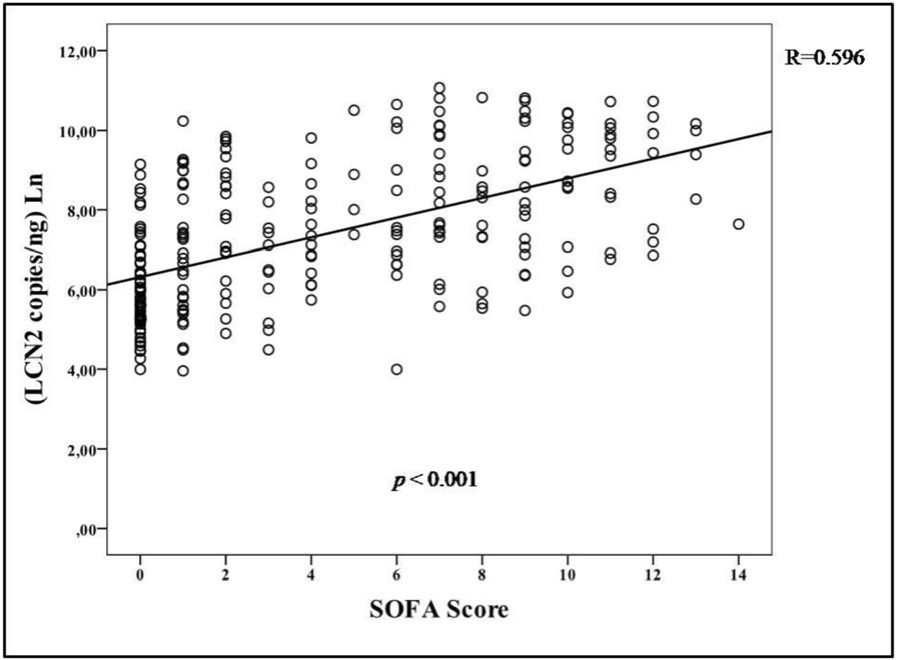
Dot plot showing the correlation between LCN2 expression levels and SOFA score in surgical patients with infection

**Fig. 2 (abstract P014).**
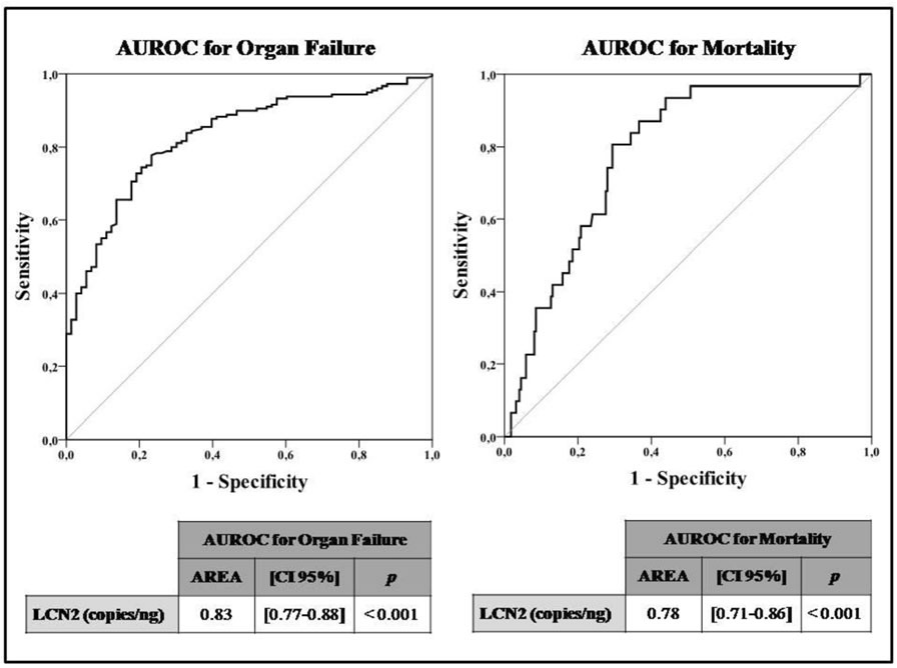
AUROCs for differential diagnosis of organ failure and mortality in surgical patients with infection

### P015 Prospective validation of an 18-mRNA score for diagnosis of infection in critically ill patients

#### TE Sweeney^1^, AR Moore^2^, J Roque^2^, B Shaller^2^, T Asuni^2^, JE Levitt^3^, JG Wilson^2^, P Khatri^4^, M Remmel^1^, D Rawling^1^, O Liesenfeld^1^, AJ Rogers^2^

##### ^1^Inflammatix, Burlingame, United States; ^2^Stanford University, Medicine, Palo Alto, United States; ^3^Stanford University, Levitt, Palo Alto, United States; ^4^Stanford University, Immunity, Transplantation and Infections, Palo Alto, United States

**Introduction:** There is an urgent need for improved diagnostics for acute infections to help physicians decide whether to treat with antibiotics. We previously described an 18-host-mRNA diagnostic consisting of an 11-mRNA Sepsis MetaScore (SMS) to determine the presence of an infection, and a 7-mRNA bacterial-viral score (BVS) to discriminate between bacterial and viral sources [1,2].

**Methods:** We collected PAXgene™ RNA blood from 165 patients enrolled in the Stanford ICU Biobank from 2016-2017. Infection status was adjudicated by Stanford physicians post-hoc using clinical data from the electronic medical record. Low-RNA samples were removed, and target mRNAs were quantitated using NanoString nCounter™ and difference-of-geometric-mean mRNA scores were calculated by Inflammatix [2], blinded to clinical phenotyping. Primary outcome was performance of the two scores in correctly diagnosing physician-adjudicated bacterial or viral infection. Secondary outcome was comparison to procalcitonin (PCT), which was drawn only according to treating physician preference.

**Results:** Of 165 patients, physicians adjudicated patients as: 29 noninfected, 102 infected (71 bacterial, 14 viral, 2 fungal, 15 mixed infections), and 33 with uncertain status. The SMS had an AUROC of 0.83 for separating infection of any type from noninfected status. The BVS had an AUROC of 0.95 for separating bacterial from viral infection. Both SMS and BVS were substantially better than PCT across all adjudicated patients and in matched pairs (PCT AUROC 0.7 for any infection vs noninfected; PCT AUROC 0.82 for bacterial vs. viral; Fig. 1). When used together, the SMS and BVS were able to separate patients based on infection status (Fig. 2).

**Conclusions:** We prospectively validated an 18-mRNA host-response diagnostic module in a blinded, independent study, confirming high accuracy for the presence and type of infection in a critically ill population.

**References**

1) Sweeney TE et al, Sci Transl Med, 287ra71, 2015

2) Sweeney TE et al, Sci Transl Med, 346ra91, 2016

**Fig. 1 (abstract P015).**
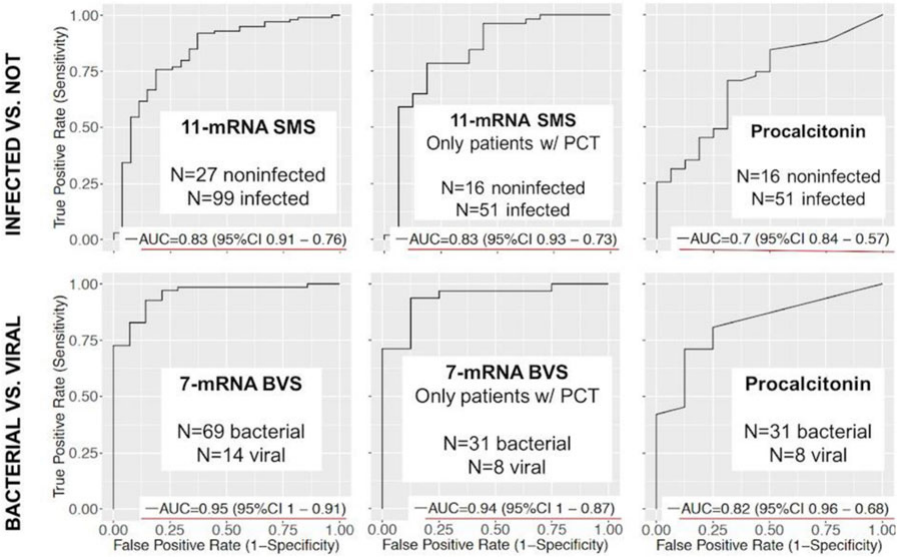
ROC curves of the infection-diagnostic Sepsis MetaScore (SMS) and the bacterial-vs-viral BVS score, shown both for all patients, and in head-to-head comparison with procalcitonin

**Fig. 2 (abstract P015).**
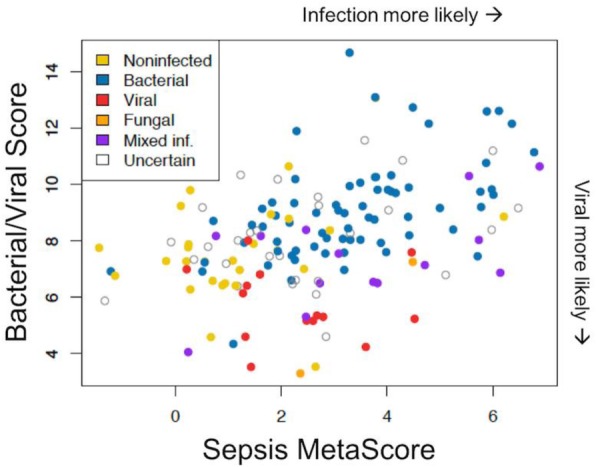
Plotting patients according to transcriptomic scores in two dimensions (x-axis, SMS; y-axis, BVS) shows accurate discrimination by adjudicated infection type

### P016 Low IgG and IgM levels do not predict mortality in cancer patients with septic shock

#### G Oliveira, C Park, J Almeida, M Mourão, S Rizk, J Fukushima, L Hajjar

##### Instituto do Cancer do Estado de São Paulo, Intensive care unit, São Paulo, Brazil

**Introduction:** Sepsis is an inflammatory state due to an exacerbated immune response against infection. In cancer patients, sepsis presents a 10-fold higher mortality than in general population and leads to longer intensive care unit (ICU) and hospital lengths of stay. It has been shown that reduced levels of circulating immunoglobulins (Ig) might be a surrogate marker of unfavorable outcome in sepsis [1]. The aim of this study was to evaluate the association between Ig levels in plasma and 60-day mortality rate in cancer patients with septic shock.

**Methods:** From December 2017 to November 2018, we conducted a prospective study in the intensive care unit (ICU) of Cancer Institute of State of Sao Paulo, an 84-bed ICU linked to University of Sao Paulo. Patients ≥18 years old with cancer and septic shock were enrolled. Descriptive statistics were computed for demographic and outcome variables. Laboratory data and Ig levels were collected at ICU admission and at days 1, 2 and 3. A multivariate analysis was performed to evaluate predictors of 60-day mortality.

**Results:** A total of 190 patients were included in the study. The 30-day and 60-day mortality were 40.5% and 45.3%, respectively. No significant differences in IgM and IgG levels were observed between survivors and non-survivors. In both groups, the median IgM levels were low and the median IgG levels were normal. In the multivariate analysis for 60-day mortality, a favorable status performance measured by the Eastern Cooperative Oncology Group (ECOG) was associated with better survival; metastatic disease, higher Sequential Organ Failure Assessment (SOFA) score at admission and higher levels of initial lactate were associated with increased mortality.

**Conclusions:** Low levels of serum endogenous immunoglobulins are not predictors of 60-day mortality in cancer patients with septic shock.

**Reference**

1. Bermejo-Martín JF et al. J Intern Med. 276(4):404-12, 2014.

### P017 Measurement of plasticity (deformability) of neutrophils and monocytes provides a rapid and early indicator of sepsis

#### R Sheybani^1^, M Hem^1^, A Shah^1^, M Samoszuk^1^, H Omran^1^, T Caffery^2^, C Thomas^2^, H Tse^1^, H O’Neal^2^

##### ^1^Cytovale, San Francisco, United States; ^2^Louisiana State University Health Sciences Center, Baton Rouge, Louisiana, United States

**Introduction:** Cytovale has developed a rapid biophysical assay of the host immune response which can serve as a rapid and reliable indicator of sepsis. Neutrophils and monocytes undergo characteristic structural and morphologic changes in response to infection. One type of response is the generation of neutrophil extracellular traps (NETs), these have been proposed as potential mediators for widespread tissue damage. During NETosis there is a fundamental reorganization of a cell’s chromatin structure – a signal that we have shown is sensitively measured by the Cytovale cytometer. We hypothesized that quantification of plasticity (deformability) of leukocytes in the peripheral blood provides an early indicator of sepsis. The Cytovale assay uses microfluidic cytometry to measure the plasticity of up to 100,000 white blood cells from EDTA-anticoagulated, peripherally-collected whole blood and provides a result in 5 minutes.

**Methods:** In two prospective studies conducted in two academic medical centers in Baton Rouge, LA, the Cytovale test was performed on peripheral blood samples obtained from 500 patients who presented to the emergency department with signs or symptoms suggestive of infection. The two studies included high acuity patients (400 patient study) and low acuity patients (100 patient study). An adjudicated reference diagnosis of sepsis or no sepsis was established for each subject, using consensus definitions, by review of the complete medical records.

**Results:** The Receiver Operator Curve (ROC) performance of the Cytovale assay for both studies demonstrated an Area Under the Curve (AUC) greater than 0.85 (Fig. 1).

**Conclusions:** Measurement of neutrophil and monocyte plasticity by a novel assay provides an accurate and rapid indication of sepsis in patients who present to an emergency room with signs or symptoms of infection.

**Fig. 1 (abstract P017).**
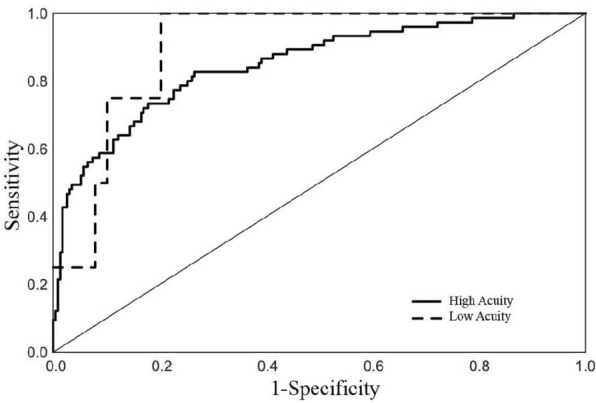
ROC performance of Cytovale test

### P018 Plasma hepatocyte growth factor in sepsis and its association with mortality: a prospective observational study

#### F Peng, C Liang, W Chang, Q Sun, J Xie, H Qiu, Y Yang

##### Zhongda Hospital, School of Medicine, Southeast University, Department of Critical Care Medicine, Nanjing, China

**Introduction:** Sepsis and septic shock are commonly associated with endothelial cell injury. Hepatocyte growth factor (HGF) is a multifunctional protein involved in endothelial cell injury and plays a pivotal role in sepsis. This study assesses its correlation with relevant endothelial cell injury parameters and prognostic value in patients with sepsis.

**Methods:** A prospective, observational cohort study was conducted in patients with sepsis admitted to the department of critical care medicine at the Zhongda Hospital from November 2017 to March 2018. The plasma HGF level was collected on the first 24h after admission (day 1) and day 3, then was measured by enzyme-linked immunosorbent assay. The primary endpoint was defined as all-cause 28-day mortality. Furthermore, we analyzed the correlation of HGF with relevant endothelial cell injury markers.

**Results:** Eighty-six patients admitted with sepsis were included. HGF levels of non-survivors were elevated upon day 1 (1940.62 ± 74.66pg/mL vs. 1635.61 ± 47.49pg/mL; p = 0.002) and day 3 (1824.82 ± 137.52pg/mL vs. 1309.77 ± 83.49pg/mL; p = 0.001) compared with that in survivors, and showed a strong correlation with von Willebrand factor (r = 0.45, p <0.0001), lactate (r = 0.35, p = 0.0011), pulmonary vascular permeability index (r = 0.38, p = 0.0241), first 24 h fluid administration (r = 0.38, p <0.0001) and sequential organ failure assessment score (r = 0.40, p = 0.0001) (Fig. 1). Plasma levels were able to discriminate prognostic significantly on day 1(AUC: 0.72, 95%CI: 0.60-0.84) and day 3 (AUC: 0.77, 95%CI: 0.63-0.91) (Fig. 2).

**Conclusions:** HGF levels are associated with sepsis and are correlated with established markers of endothelial cell injury. Elevated HGF level in sepsis patients is a predictor of mortality.

**Fig. 1 (abstract P018).**
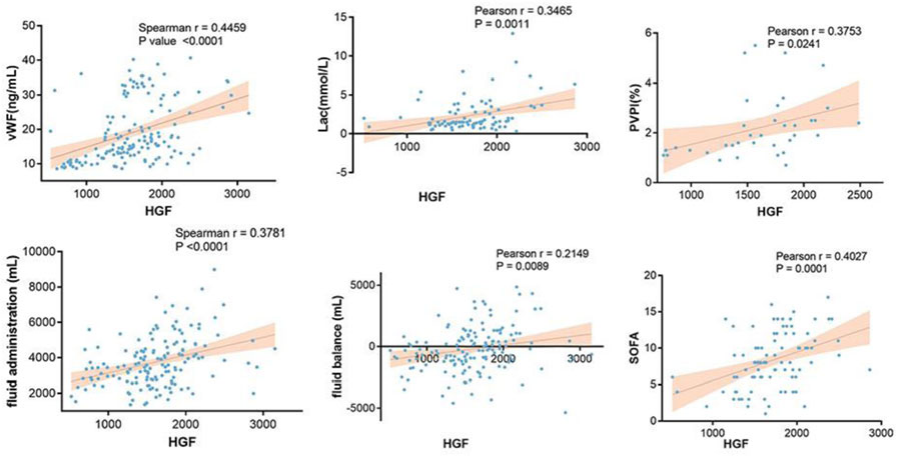
Correlation between HGF levels with markers of endothelial cell injury and SOFA

**Fig. 2 (abstract P018).**
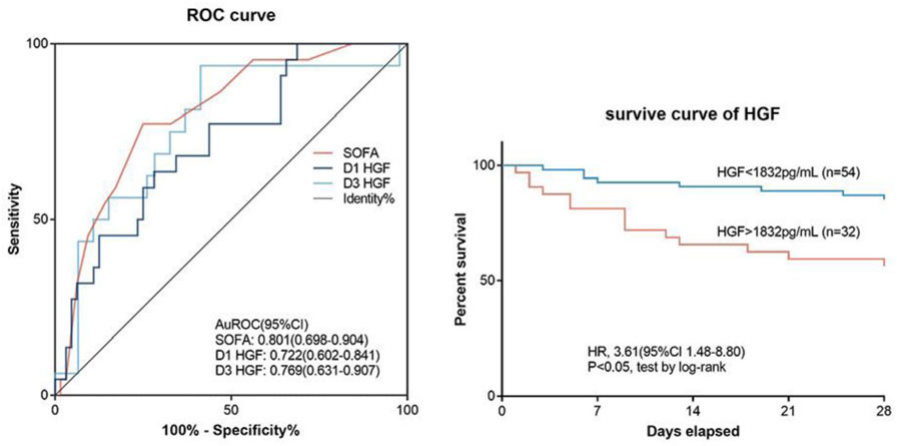
Prognostic value of HGF level for sepsis patients

### P019 Calprotectin, a powerful biomarker for the diagnosis of bacterial infections

#### A Havelka^1^, P Venge^2^, A Larsson^2^

##### ^1^Department of Molecular Medicine and Surgery, Karolinska Institute, Stockholm, Sweden; ^2^Department of Medical Sciences, Uppsala University, Uppsala, Sweden

**Introduction:** The rapidly growing problem with antibiotic resistance has resulted in demand for more specific and restricted use of antibiotics. Biomarkers which can diagnose an infection in early stage and distinguish between bacterial and viral infections could possibly reduce the use of antibiotics. Calprotectin is one of the most abundant proteins in the cytosol of neutrophil granulocytes and is released upon activation of neutrophils. The aim of this study was to investigate the performance of calprotectin as a marker for bacterial infection and its possibility to distinguish between bacterial and viral infections.

**Methods:** The study group consisted of 432 subjects including 144 healthy, noninfected, control patients and 288 patients with confirmed etiology of their infections, 185 patients with bacterial infection, 54 with viral infection, 26 with mycoplasma infection, and 23 with a bacterial infection as a secondary infection to influenza. Calprotectin was measured in serum samples with a particle enhanced turbidimetric assay (Gentian AS, Norway). Heparin Binding Protein (HBP) and Procalcitonin were analyzed by sandwich immunoassays (Hycult Biotech and Thermo Fisher Scientific).

**Results:** Performance of Calprotectin in the diagnosis of bacterial infections as well as in distinguishing between bacterial and viral infections was compared to performance of Procalcitonin and HBP. Calprotectin was superior in diagnosis of bacterial infections as well as in differentiating bacterial from viral infections. Results are presented in Table 1**.** Interestingly, calprotectin was the only biomarker with ability to distinguish between mycoplasma and viral infections.

**Conclusions:** Calprotectin is a promising biomarker for diagnosis of bacterial infections. Our results indicate that Calprotectin is superior to Procalcitonin and HBP in diagnosis of bacterial infections and in differentiation between bacterial and viral infections including mycoplasma infections.

**Table 1 (abstract P019).** Diagnostic performance of the studied biomarkers

Biomarker in group comparison	AUROC (95% CI)	Specificity (%)	Sensitivity (%)
Healthy vs bacteria			
Calprotectin cut-off 1.19 mg/L	0.950	93.0	87.8
HBP cut-off 5.7 μgL	0.888	85.4	76.0
PCT cut-off 0.08 μg/L	0.883	94.4	78.4
	AUROC (95% CI)		
Bacteria vs virus	Calprotectin	HBP	PCT
Mycoplasma pneumoniae	0.872	0.519	0.529

### P020 A personalized randomized trial of validation and restoration of immune dysfunction in severe infections and sepsis: PROVIDE

#### N Antonakos^1^, I Tsangaris^2^, D Markopoulou^3^, N Rovina^4^, A Prekates^5^, E Antoniadou^6^, V Theodorou^7^, A Stefos^8^, G Vlachogianni^9^, E Giamarellos-Bourboulis^1^, G Dimopoulos^2^, M Netea^10^

##### ^1^National and Kapodistrian University of Athens, 4th Department of Internal Medicine, Athens, Greece; ^2^National and Kapodistrian University of Athens, 2nd Department of Critical Care Medicine, Athens, Greece; ^3^KAT General Hospital, Intensive Care Unit, Athens, Greece; ^4^National and Kapodistrian University of Athens, Intensive Care Unit 1st Department of Respiratory Medicine, Athens, Greece; ^5^Tzaneion General Hospital, Intensive Care Unit, Piraeus, Greece; ^6^G.Gennimatas General Hospital, Intensive Care Unit, Thessaloniki, Greece; ^7^Dimocriteion University of Thrace, Intensive Care Unit, Alexandroupolis, Greece; ^8^University of Thessaly, Department of Internal Medicine, Larissa, Greece; ^9^Aghios DImitrios General Hospital, Intensive Care Unit, Thessaloniki, Greece; ^10^Radboud University, Department of Internal Medicine, Nijmegen, Netherlands

**Introduction:** Based on the recent post-hoc analysis of previous trials of the efficacy of anakinra in patients with macrophage-like activation syndrome (MALS), PROVIDE (ClinicalTrials.gov registration NCT03332225) is the first double-blind, double-dummy on-going trial aiming to the impact of immunotherapy according to personalized needs.

**Methods:** Adult patients with septic shock by the Sepsis-3 classification due to lung infection or primary bacteremia or acute cholangitis are screened using two consecutive measurements of ferritin and of HLA-DR/CD14 co-expression for MALS (ferritin above 4,420 ng/ml) or immunosuppression (HLA-DR/CD14 less than 30%) and randomized into immunotherapy with either anakinra (targeting MALS) or recombinant IFNγ (targeting immunosuppression) and into placebo treatment. Main exclusion criteria are primary and secondary immunodeficiencies and solid and hematologic malignancies.

**Results:** 101 patients have been screened so far. Most common infections are community-acquired pneumonia (41.6%), hospital-acquired pneumonia (26.7%) and primary bacteremia (12.9%). Mean +/- SD SOFA score is 12.6 +/- 2.9 and Charlson’s comorbidity index 5.21 +/- 2.44; 25 patients have MALS (24.8%); two immunosuppression (2%); the majority remain unclassified for immune state.

**Conclusions:** Current screening suggests greater frequency of MALS than recognized so far in a setting of septic shock due to lung infection or primary bacteremia or acute cholangitis.

### P021 Development of an algorithm to predict mortality in patients with sepsis and coagulopathy

#### D Hoppensteadt^1^, A Walborn^2^, M Rondina^3^, J Fareed^1^

##### ^1^Loyola University Medical center, Pathology, Maywood, United States; ^2^Loyola University Medical center, Pharmacology, Maywood, United States; ^3^University of Utah and the GRECC, Internal Medicine and the Molecular Medicine Program, Salt Lake City, United States

**Introduction:** Sepsis is a systemic response to infection which involves inflammation, infection response, hemostatic dysregulation, endothelial dysfunction, and platelet activation. The purpose of this study was to develop an equation incorporating biomarker levels at ICU admission to predict mortality in patients with sepsis, to test the hypothesis that using a combination of biomarkers of multiple systems would improve predictive value.

**Methods:** Plasma samples were collected from 103 patients with sepsis at the time of ICU admission. Biomarker levels were measured using commercially available, ELISA methods. Clinical data, including the ISTH DIC score, SOFA score, and APACHE II score were also collected. 28-day mortality was used as the primary endpoint. Stepwise linear regression modeling was performed to generate a predictive equation for mortality.

**Results:** Differences in biomarker levels between survivors were quantified and using the Mann-Whitney test and the area under the receiver operating curve (AUC) was used to describe predictive ability. Significant differences (p<0.05) were observed between survivors and non-survivors for PAI-1 (AUC=0.70), procalcitonin (AUC=0.77), HMGB-1 (AUC=0.67), IL-6 (AUC=0.70), IL-8 (AUC=0.70), protein C (AUC=0.71), Angiopoietin-2 (AUC=0.76), endocan (AUC=0.58), and platelet factor 4 (AUC=0.70). A predictive equation for mortality was generated using stepwise linear regression modeling. This model incorporated procalcitonin, VEGF, the IL-6:IL-10 ratio, endocan, and PF4, and demonstrated a better predictive value for patient outcome than any individual biomarker (AUC=0.87).

**Conclusions:** The use of a mathematical modeling approach resulted in the development of a predictive equation for sepsis-associated mortality with performance than any individual biomarker or clinical scoring system. Furthermore, this equation incorporated biomarkers representative of multiple physiological systems that are involved in the pathogenesis of sepsis.

### P022 The effects of biomarker clearances as markers of improvement of severity in abdominal septic shock during blood purification

#### T Taniguchi^1^, K Sato^2^, M Okajima^2^

##### ^1^Kanazawa University, Anesthesiology and Intensive Care Medicine, Kanazawa, Japan; ^2^Kanazawa University Hospital, Intensive Care Unit, Kanazawa, Japan

**Introduction:** Recently the clearances of biomarkers (BM) such as procalcitonin (PCT) and presepsin (p-SEP), appear to be better indicators than single cutoff values to diagnose septic complications or predict outcomes. Moreover, blood purification (BF) such as endotoxin absorption therapy (PMX) and continuous renal replacement therapy (CRRT) has been carried out for abdominal septic shock (ASS). However, there are few studies about BM clearances in ASS during BF. Therefore, the present study retrospectively evaluated the effects of BM clearances on improvements of severity in critical patients with ASS during BF.

**Methods:** Thirty-three patients (M/F 21/12, mean age 69 years) were entered. Septic shock was defined in sepsis-3 criteria. PMX was undergone twice and CRRT was undergone for 5 days. BM levels were measured for 5 days after ICU admitted. Moreover, SOFA scores were measured for 5 days after ICU admitted. BM clearances were determined at the entering ICU and 1, 3, and 5 days after ICU admitted. The improvements of severity were determined the differences of SOFA scores after ICU admitted. Primary outcome is the correlation between BM clearances and the improvement of severity. Secondary outcomes are the changes of BM after ICU admitted, and mortality in ICU.

**Results:** Two of 33 patients died after ICU admission. PCT and lactate levels were improved 5 days after ICU admitted (61.1 to 10.8 ng/mL; p<0.05, 4.2 to 1.1 mmol/L; p<0.05). CRP and p-SEP levels were not improved. SOFA scores decreased (13.4 to 6.5; p<0.05) at 5 days after ICU admitted. There were significantly correlations between PCT and lactate clearances and the improvement of severity (Y=4.02 +0.11X; R2=0.07, p=0.0102, Y=2.75 +0.03X; R2=0.226, p<0.0001). There were not significantly correlations between CRP and p-SEP clearances with the improvement of severity.

**Conclusions:** The present study showed that PCT and lactate clearances significantly correlated the improvement of severity in critical patients with ASS during BF.

### P023 Decreased thrombin generation potential is associated with increased thrombin generation markers in sepsis associated coagulopathy

#### J Fareed^1^, F Siddiqui^1^, R Laddu^1^, D Hoppensteadt^1^, M Rondina^2^, E Brailovsky^3^

##### ^1^Loyola University Medical Center, Pathology, Maywood, United States; ^2^University of Utah School of Medicine, Department of Internal Medicine, Salt Lake City, United States; ^3^Loyola University Medical Center, Cardiology, Maywood, United States

**Introduction:** Sepsis associated coagulopathy (SAC) is commonly seen in patients which leads to dysfunctional hemostasis. The purpose of this study is to determine the thrombin generation potential of baseline blood samples obtained from SAC patients and demonstrate their relevance to thrombin generation markers.

**Methods:** Baseline citrated blood samples were prospectively collected from 49 patients with SAC at the University of Utah clinic. Citrated normal controls (n=50) were obtained from George King Biomedical (Overland Park, KS). Thrombin generation studies were carried out using a flourogenic substrate method. TAT and F1.2 were measured using ELISA methods (Seimens, Indianapolis, IN). Functional antithrombin levels were measured using a chromogenic substrate method.

**Results:** The peak thrombin levels were lower (82 ± 40nM) in the DIC patients in comparison to higher levels observed in the normal plasma (133 ± 10nM). The AUC was lower (561 ± 280) in the DIC group in comparison to the normals (624 ± 18). The DIC group showed much longer lag time (4.1 ± 2.1) in comparison to the normal group (2.1 ± 2.2). Wide variations in the results were observed in these parameters in the DIC group. The F1.2 levels in the DIC group were much higher (570±48 pmol) in comparison to the normal (210 ± 25 pmol). The TAT levels also increased in the DIC group (27.9 ± 5.1 ng/ml) in comparison to the normal (2.8 ± 0.8 ng/ml). The functional antithrombin levels were decreased in the DIC group (64 ± 11%).

**Conclusions:** These results validate that thrombin generation such as F1.2 and TAT are elevated in patients with DIC. However thrombin generation parameters are significantly decreased in this group in comparison to normals. This may be due to the consumption of prothrombin due to the activation of the coagulation system. The decreased functional AT levels observed in the DIC group are due to the formation of the complex between generated thrombin and antithrombin.

### P024 Relationship of markers of inflammation, infection, and endothelial function to mortality and severity of coagulopathy in patients with sepsis-associated DIC

#### D Hoppensteadt^1^, A Walborn^2^, M Rondina^3^, J Fareed^1^

##### ^1^Loyola University Medical center, Pathology, Maywood, United States; ^2^Loyola University Medical center, Pharmacology, Maywood, United States; ^3^University of Utah School of Medicine, Internal medicine, Salt Lake City, United States

**Introduction:** Sepsis-associated disseminated intravascular coagulation (DIC) is a complex clinical scenario involving derangement of many processes, including hemostasis. Assessment of markers including inflammation, endothelial function, and endogenous anticoagulants may provide insight into DIC pathophysiology and lead to improved methods for assessment of patient condition and response to treatment.

**Methods:** Citrated plasma samples were collected from 102 patients with sepsis and suspected DIC at ICU admission and on days 4 and 8. DIC score was determined using the ISTH scoring algorithm (e.g. platelet count, PT/INR, fibrinogen and D-Dimer). CD 40 Ligand (CD40L), Plasminogen inhibitor 1 (PAI-1), nucleosomes, Procalcitonin (PCT), Microparticle tissue factor (MP-TF) and Prothrombin 1.2 (F1.2) were measured using commercially available ELISA kits. Protein C activity was measured using a clot-based assay. Interleukin 6 (IL-6), Interleukin 8 (IL-8), Interleukin 10 (IL-10), Tumor necrosis factor alpha (TNFα), and Monocyte chemoattractant protein (MCP-1) were measured using biochip technology.

**Results:** Significant differences in levels of Protein C (p=0.009), PCT (p=0.0005), IL-6 (p=0.019), IL-8 (p=0.0149), PAI-1 (p=0.015), were observed between survivors and non-survivors. Significant variation of Protein C (p=0.002), nucleosomes (p=0.05), PCT (p<0.0001), IL-6 (p=0.001), IL-8 (p=0.003), IL-10 (p=0.011), TNFα (p=0.021) and MCP-1 (p=0.021) were observed based on severity of DIC score.

**Conclusions:** Markers from multiple systems perturbed in DIC were associated with mortality, suggesting that while these systems may not be routinely evaluated in the normal course of patient care, dysfunction of these systems contributes significantly to mortality. In addition, numerous inflammatory cytokines showed an association with DIC score. This suggests that the measurement of additional markers in sepsis-associated DIC may be of value in the prediction of mortality and may be helpful in guiding treatment for these patients.

### P025 Usefulness of plasminogen activator inhibitor-1 (PAI-1) as a predictive marker for identification of sepsis-induced DIC

#### J Maruyama, K Hoshino, Y Irie, S Miyagawa, R Hokama, M Koie, M Nakashio, Y Kawano, T Kitamura, H Ishikura

##### Fukuoka University Hospital, Department of Emergency and Critical Care Medicine, Fukuoka, Japan

**Introduction:** There is a crosstalk between inflammation and coagulation and disseminated intravascular coagulation (DIC) especially sepsis-induced DIC is a one of the most significant causes of mortality in intensive care units. Meanwhile, there are various types of DIC and sepsis-induced DIC is a type of suppressed fibrinolysis DIC. In this study, we aimed to identify coagulation/fibrinolysis markers useful for discriminating whether sepsis-induced DIC or not.

**Methods:** This is a single-center retrospective observational study of 233 patients with DIC according to the Japanese Association for Acute Medicine (JAAM) DIC criteria (JAAM DIC score ≥4) from July 2017 to June 2018. We divided DIC patients into sepsis and non-sepsis using Sepsis-3 diagnosed criteria and univariate and multivariate logistic regression analyses were performed to identify an independent predictive marker of sepsis-induced DIC among coagulation/fibrinolysis markers on ICU admission.

**Results:** Sepsis-induced DIC (S-DIC) group (n=62) was significantly higher DIC score and SOFA score rather than non-sepsis-induced DIC (NS-DIC) group (n=171) [DIC score; 5 (5-7) vs. 4 (4-5), P<0.01. SOFA score; 11 (9-14) vs. 6 (4-10), P<0.01.].

About coagulation/fibrinolysis markers, S-DIC group was significantly lower the FDP, D-dimer, and PIC rather than NS-DIC group (P<0.01), and higher the PAI-1 (P<0.01). Moreover, PAI-1 was identified as one of the independent predictive markers of sepsis-induced DIC by multivariate logistic regression.

**Conclusions:** Recently, we reported that PAI-1 was a useful predictive marker of mortality in sepsis. From this study we suspected that PAI-1 is a useful marker for discriminating sepsis-induced DIC. Furthermore, we confirmed that sepsis-induced DIC was a type of DIC with suppressed fibrinolysis. Therefore, we recommend measuring PAI-1 against sepsis and sepsis-induced DIC patients.

### P026 Does EAA value reflect severity of condition in patients admitted to intensive care unit?

#### T Ikeda^1^, S Ono^1^, S Suda^1^, T Nagura^1^, M Tomino^2^, M Sugi^2^, Z Wajima^2^

##### ^1^Tokyo Medical University, Hachioji Medical University, Division of Critical Care Medicine, Tokyo, Japan; ^2^Tokyo Medical University, Hachioji Medical University, Department of Anesthesiology, Tokyo, Japan

**Introduction:** The Endotoxin Activity Assay (EAA) is a rapid immunodiagnostic test based on chemiluminescence. It was approved by the FDA in 2003 as a diagnostic reagent for risk assessment of severe sepsis in the ICU. Ascertaining endotoxin levels in the bloodstream is important in targeting patients and determining the appropriate timing for initiation of treatment. It has high sensitivity and specificity for endotoxin, and is considered to be useful in predicting clinical symptoms and determining prognosis. The usefulness of the EAA has yet to be fully clarified.

**Methods:** A total of 142 patients admitted to the ICU between January 2014 and June 2018 with suspected sepsis or sepsis were enrolled. The EAA was conducted within 24 hr after admission. Patient characteristics were determined, together with levels of IL-6, procalcitonin, presepsin, and PaO2/FiO2. Thereafter, the patients were classified into 5 groups depending on their EAA value: 1) < 0.2; 2) from ≤ 0.2 to < 0.4; 3) from ≤ 0.4 to < 0.6; 4) from ≤ 0.6 to < 0.9; and 5) ≤0.9). The transition of various markers was also examined. The Spearman rank correlation, Wilcoxon rank sum test, and a non-repeated ANOVA were used for the statistical analysis. A P-value of < 0.05 was considered statistically significant.

**Results:** The EAA values showed a positive correlation with both the APACHE II (r=0.48) and SOFA scores (r=0.56)(P<0.01), although that with the latter was stronger. A significant correlation was also observed with levels of procalcitonin (r=0.45) and presepsin (r=0.51). The EAA showed a high value (P<0.01) only in patients showing a positive result for blood culture; no other marker showed a significant difference between those showing a positive or negative result. At 28 days, significantly higher values were observed in the non-survival group (P<0.05) in values for the EAA and other markers.

**Conclusions:** EAA value tended to correlate with disease severity (APACHEII and SOFA scores) in patients admitted to the ICU.

### P027 Intraperitoneal microdialysis detects early peritonitis caused by intraabdominal bacterial infection in a pig model

#### S Hødnebø, S Pischke, A Barratt-Due, E Lindholm, T Tønnessen

##### Oslo University Hospital, Division of Emergencies and Intensive Care, Cardiothoracic clinic, Oslo, Norway

**Introduction:** Common complications following abdominal surgery are intestinal leaks, with subsequent abdominal sepsis. Early diagnosis is important to allow early intervention. The current clinical methods are insufficient for early detection. We hypothesized that intraperitoneal microdialysis allows detection of peritonitis prior to changes in standard clinical parameters in a pig model.

**Methods:** Bacterial peritonitis was induced in 5 pigs by bowel perforation and intraperitoneal fecal instillation, one pig underwent sham surgery. Intraperitoneal microdialysis catheters were placed in each abdominal quadrant. The observation time was 10 hours.

**Results:** In peritonitis pigs the intraperitoneal lactate increased during the first two hours and remained elevated throughout the observation time (Table 1), whereas the arterial lactate remained within reference range (<1.6 mM). Intraperitoneal glucose decreased significantly. Hemodynamics were hardly influenced during the first two hours, and decreased thereafter. Sham surgery did not influence in any of the parameters.

**Conclusions:** A rapid and pronounced increase in intraperitoneal lactate and decrease in intraperitoneal glucose was observed after instillation of intraabdominal feces. Systemic lactate increase was absent, and the hemodynamic response was delayed. Postoperative intraperitoneal microdialysis is applicable in detecting peritonitis earlier than standard clinical monitoring and should be evaluated in a clinical study in order to explore if early intervention based on MD data will reduce ICU length of stay, morbidity and mortality.

**Table 1 (abstract P027).** Intraperitoneal microdialysis lactate values, arterial lactate values, mean arterial pressure (MAP) and cardiac output (CO) at different time points after induced bacterial peritonitis in pigs

Time (h)	Lactate (mM), intraperitoneal	Lactate (mM), arterial	MAP (mmHg)	CO (l/min)
Baseline	2.0 (1.3-2.6)	0.6 (0.5-0.8)	62 (62-71)	4.8 (4.5-5.2)
1	3.0 (2.8-3.3)	0.7 (0.5-0.8)	62 (58-83)	5.2 (4.9-5.5)
2	6.3 * (5.7-6.7)	0.8 (0.7-0.8)	67 (63-78)	5.4 (5.0-5.8)
5	5.5 *(4.8-6.0)	1.1 (0.9-1.5)	60 (58-60)	3.6 (3.3-3.8)
10	7.0 * (6.7-7.2)	1.1 (1.1-1.7)	41* (35-48)	3.1 (2.5-3.6)

### P028 Validation of B·R·A·H·M·S PCT direct, a new sensitive point-of-care device for rapid measurement of procalcitonin

#### L Velly^1^, P Hausfater^1^, M Seidel^2^, J Lotz^3^, C Rechner^4^, C Brochet^5^, M Oppert^6^

##### ^1^Emergency dpt Pitié-Salpêtrière hospital APHP and Sorbonne Universités GRC-14 BIOSFAST, Paris, France; ^2^Unfallkrankenhaus Berlin, Berlin, Germany; ^3^Universitätsmedizin Johannes Gutenberg-Universität Mainz, Mainz, Germany; ^4^Thermo Fisher Scientific, Hennigsdorf, Germany; ^5^Biochemistry dpt Pitié-Salpêtrière hospital, APHP, Paris, France; ^6^Klinikum Ernst von Bergmann gGmbH, Potsdam, Germany

**Introduction:** Procalcitonin (PCT) is a highly sensitive and specific biomarker for bacterial infection.

B·R·A·H·M·S PCT direct is a new point of care test (POC) for fast measurement of PCT in whole blood (capillary or venous) with a measuring range of 0.1-10 μg/L.

**Methods:** This multicentre study examined the correlation of capillary and venous whole blood samples measured on B·R·A·H·M·S PCT direct compared to established reference methods B·R·A·H·M·S PCT sensitive KRYPTOR and Elecsys B·R·A·H·M·S PCT using EDTA plasma. The design was based on the related CLSI Guidelines EP09-A3.

**Results:** 279 patients for venous EDTA whole blood and 93 patients for capillary blood (fingertip) were included in this study. The Pearson correlation coefficient (log transformed) between venous or capillary whole blood and the reference method was r2=0.95. The concordance to reference methods was 93% for venous blood and 95% for capillary blood related to a clinical cut-off of 0.5 μg/L, with a sensitivity of 92% (venous and capillary blood) and a specificity of 95% (venous blood) and 97% (capillary blood), respectively. The concordance at the clinical cut-off 0.25 μg/L was 93% for venous blood and 91% for capillary blood. No significant bias was observed compared to the reference method.

**Conclusions:** This study found a very good correlation and diagnostic accuracy of the new, sensitive POC device for rapid measurement of PCT (TAT 20 min). The B·R·A·H·M·S PCT direct test allows an accurate measurement of PCT in whole blood at a point of care comparable to established lab based B·R·A·H·M·S PCT assays.

### P029 Is procalcitonin a reliable biomarker for infection in trauma patients? Our experience

#### E De sanso^1^, C Morena^2^, MA Palazzo^3^, E Gamberini^2^, F Avolio^4^, E Russo^3^, V Agnoletti^2^

##### ^1^University of Piemonte Orientale A. Avogadro, Anesthesia and Intensive Care, Turin, Italy; ^2^Anesthesia and Intensive Care AUSL Romagna Anesthesia and Intensive Care Unit, AUSL Romagna Anesthesia and Intensive Care Unit, Cesena, Italy; ^3^Anesthesia and Intensive Care AUSL Romagna Anesthesia and Intensive Care Unit, Anesthesia and Intensive Care AUSL Romagna Anesthesia and Intensive Care Unit, Cesena, Italy; ^4^Anesthesia and Intensive Care AUSL Romagna Anesthesia and Intensive Care Unit, Cesena, Italy

**Introduction:** Procalcitonin (PCT) is a serum biomarker suggested by the Surviving Sepsis Campaign to aid in determination of the appropriate duration of therapy in septic patients. Trauma patients have a high prevalence of septic complications, often difficult to distinguish from inflammatory response. PCT values typically declined after 72h from trauma and increased only during secondary systemic bacterial infections. The aims of the study are to evaluate reliability and usefulness of PCT serum concentration in trauma.

**Methods:** We retrospectively analyzed data from 40 trauma patients admitted to ICU at Bufalini Hospital - Cesena, from July 2017 to August 2018. We collected data about antimicrobial therapy, Injury severity score (ISS), first arterial Lactate in emergency room, SOFA score and Sepsis severity. Plasma PCT concentration was measured using an automate analyzer (Modular E-Brahms) on 1st day of antimicrobial therapy and every 48h hours. Antimicrobial therapy was stopped according to a local protocol; however medical judgment was considered the overriding point for therapeutic decision.

**Results:** Median ISS of patients was 33.5, inter quartile range (IQR) 13.5. PCT mean concentration at the starting of antimicrobial treatment was 13.07 μg/L (d.s 40.1), median 0.72 (IQR 10.13). No significative correlation (Spearman´s Rho Test) was found between PCT at day 1 of antimicrobial therapy and ISS (Rho -0.186), between first arterial Lactate in ER and PCT (Rho 0.158). Daily course of PCT was not related to distance from trauma (Rho -0.116). In 21 of 40 patients (52.2 %) PCT measurement led physician to save days of antimicrobial therapy compared with standard clinical practice. We couldn´t find any cut off value.

**Conclusions:** Our experience suggests that PCT could help physician to optimize duration of antimicrobial therapy in trauma patients. No standard approach can be recommended at present.

### P030 Α randomized prospective clinical trial to assess the role of procalcitonin (PCT)-guided antimicrobial therapy to reduce long-term infections sequelae (PROGRESS)

#### E Kyriazopoulou^1^, A Panagaki^1^, L Liaskou^1^, E Drakou^2^, K Marousis^2^, V Apostolopoulos^3^, A Makina^4^, V Kolonia^5^, S Lagou^6^, EJ Giamarellos-Bourboulis^1^

##### ^1^4th Department of Internal Medicine, National and Kapodistrian University of Athens, Athens, Greece; ^2^1st Department of Internal Medicine, G.Gennimatas General Hospital, Athens, Greece; ^3^1st Department of Internal Medicine, Thriasio Elefsis General Hospital, Athens, Greece; ^4^2nd Department of Internal Medicine, Thriasio Elefsis General Hospital, Athens, Greece; ^5^2nd Department of Internal Medicine, Sismanogleio General Hospital, Athens, Greece; ^6^3rd Department of Internal Medicine, National and Kapodistrian University of Athens, Athens, Greece

**Introduction:** Long duration of antimicrobial treatment may predispose to colonization and subsequent infections by multidrug-resistant organisms (MDRO) and Clostridium difficile. PROGRESS (ClinicalTrials.gov registration NCT03333304) is an on-going trial aiming to use PCT for the restraining of this calamity.

**Methods:** Adult patients with sepsis by the Sepsis-3 classification and any of five infections (pneumonia community-acquired; hospital- acquired or ventilator-associated; acute pyelonephritis; primary bacteremia) are randomized to PCT-guided treatment or standard of care (SOC) treatment. In the PCT arm antibiotics are discontinued when PCT on or after day 5 is decreased by more than 80% of the baseline or remains below 0.5 ng/ml; in the SOC arm antibiotics are discontinued at the discretion of the attending physician. Patients are followed for six months. Primary endpoint is the rate of infections by MDRO and/or C.difficile or death. Serial stool samples are cultured for MDRO and screened for glutamate dehydrogenase antigen and toxins of C.difficile.

**Results:** 201 patients have been enrolled so far. Mean ± SD SOFA score is 4.4 ± 2.4. Most common diagnoses are community-acquired pneumonia (57.2%) and acute pyelonephritis (40.3%). At baseline, 10.4% were colonized by MDRO and 3% by C.difficile. Residency in health-care facilities was the only variable associated with C.difficile colonization (odds ratio 7.04; 95% CI: 1.22-40.44). MDRO colonization was associated with residency in health-care facilities (odds ratio 6.74; 95% CI: 2.07-21.93) and hospitalization the last three months (odds ratio 4.29; 95% CI: 1.48-12.45)

**Conclusions:** The PROGRESS trial is the first trial assessing the probable benefit from PCT guidance to reduce ecological sequelae from long-term antibiotic exposure. Analysis of baseline patient characteristics indicates that PROGRESS is a real-world trial so that results can have major clinical impact.

### P031 Prospective multi-site validation of 11-gene host response signature for influenza diagnosis

#### S Thair^1^, S Schaffert^1^, M Shojaei^2^, T Sweeney^3^, B Tang^2^, P Khatri^1^

##### ^1^Stanford Univeristy, Biomedical Informatics Research, Stanford, United States; ^2^Nepean Hospital, Department of Intensive Care Medicine, Sydney, Australia; ^3^Inflammatix, Burlingame, United States

**Introduction:** Influenza causes 650 000 deaths per year globally. It caused 80 000 deaths in the US in 2017. Rapid diagnostic tests (RIDTs) have very low sensitivity, whereas RT-PCR based tests have high sensitivity, but requires trained technicians. There are no blood-based diagnostics able to identify influenza infection and distinguish it from other infections. We have previously described a blood-based 11-gene influenza meta-signature (IMS) score to differentiate influenza from bacterial and other viral respiratory infections.

**Methods:** We prospectively validated the IMS in a multi-site validation study by recruiting 654 individuals (608 patients with suspected influenza, 46 healthy controls) in 10 community or hospital clinics across Australia. We assayed the IMS and 15 genes from viral genome of 3 influenza strains to generate the Blood Flu Score (BFS) as a measure of viremia using Nanostring from whole blood RNA.

**Results:** Using clinically determined phenotypes, the IMS score distinguished patients with influenza from healthy (AUC=0.95), non-infected (AUC=0.83), bacterial (AUC=0.88), other viruses (AUC=0.77) (Figure 1A). Interestingly, probes of BFS were found in all phenotypic groups (non-infected, bacterial, and other viral infections) to varying degrees, and positively correlate with the IMS score (r=0.53). IMS AUROCs improve when the BFS is used to inform the phenotypic groups: healthy (AUC=0.92), non-infected (AUC=0.90), bacterial (AUC=0.93), other viruses (AUC=0.88) (Figure 1B). Patients who were clinically influenza negative but had a high IMS and BFS were admitted less often, yet had ~4-fold higher mortality than those who were clinically influenza negative with low IMS and no BFS (Table 1).

**Conclusions:** Collectively, our prospective multi-center validation of the IMS demonstrates its potential in diagnosis of influenza infections.

**Table 1 (abstract P031).** Summary of outcomes for patients. Clinically negative influenza patients with a high IMS and BFS were admitted less often yet had ~ 4 fold higher mortality than those who were influenza negative with low IMS and no BFS

Clinical Influenza	+	+	-	-
Viremia (Nanostring)	-	+	-	+
N	27	112	96	55
Age (years)	56	51	55	47
Admitted (%)	77.8	60.7	64.6	47.2
ICU (%)	22.2	22.3	8.3	10.9
Mortality (%)	3.7	4.5	2.0	7.3
IMS score	11.1	12.7	9.4	11.4

**Fig. 1 (abstract P031).**
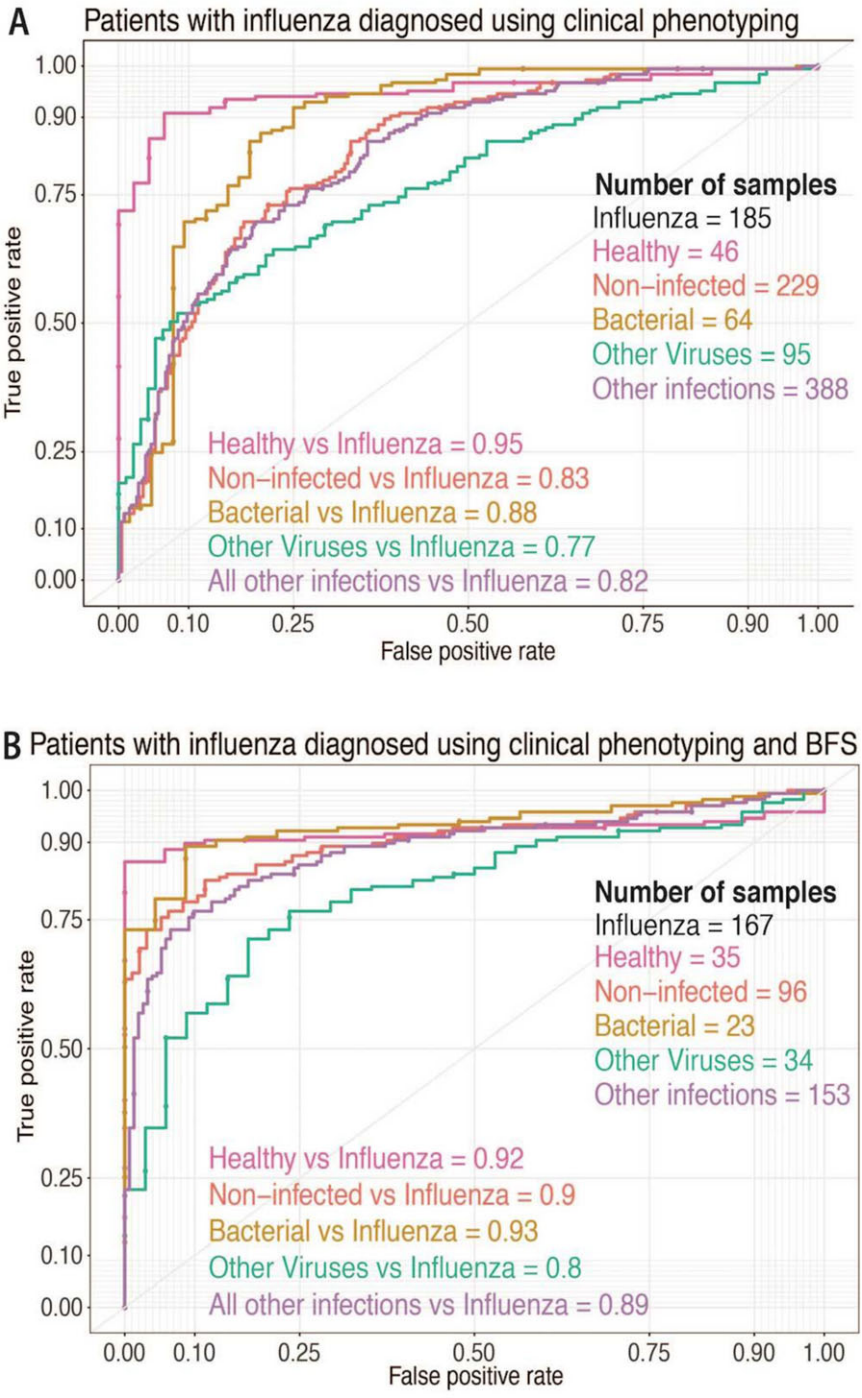
IMS area under the receiver operator characteristic (AUROC) curves for distinguishing patients with influenza from healthy, non-infected, bacterial, other viruses or all other infections combined when using using (A) clinically informed phenotyping only or (B) using clinical phenotyping and BFS ≥19. All samples with 15 >BFS<19 were removed in (B)

### P032 Modulation of innate immune responses in experimental pyelonephritis by preceding osteomyelitis

#### S Goumenos^1^, G Renieris^2^, D Droggiti^2^, L Sabracos^2^, P Papagelopoulos^1^, O Savvidou^1^, E Giamarellos-Bourboulis^2^

##### ^1^National and Kapodistrian University of Athens, 1st Department of Orthopedic Surgery, Athens, Greece; ^2^National and Kapodistrian University of Athens, 4th Department of Internal Medicine, Athens, Greece

**Introduction:** Previous findings of our group suggest that patients with Gram-negative hospital-acquired severe sepsis have better prognosis when sepsis is developing after recent multiple trauma through stimulation of favorable interleukin (IL)-10 responses [1]. Under a similar rationale, we investigated if preceding osteomyelitis may affect experimental osteomyelitis.

**Methods:** Sham or experimental osteomyelitis was induced in 32 male New Zealand white rabbits after drilling a hole at the upper metaphysis of the left tibia and implementing diluent or 5log10 of Staphylococcus aureus using foreign body. After three weeks, the foreign body was removed and experimental pyelonephritis or sham surgery was induced after ligation of the right pelvo-ureteral junction and instillation of 6log10 of Escherichia coli in the renal pelvis. Survival was recorded and circulating mononuclear cells were isolated and stimulated for the production of tumour necrosis factor-alpha (TNFa) and IL-10. At death or sacrifice, tissue outgrowth and myeloperoxidase (MPO) were measured.

**Results:** Four sham-operated rabbits (S), 16 rabbits subject to sham surgery and then pyelonephritis (SP) and 12 rabbits subject to osteomyelitis and then pyelonephritis (OP) were studied. Survival after 14 days of group SP was 56.3% and of group OP 100% (log-rank 6.59; p: 0.010). Lab findings are shown in Figure 1. Il-10 production was blunted. Negative correlation between E. coli outgrowth and tissue MPO was found at the right kidney of the OP group (rs: -0.767, p: 0.016) but not of the SP group (rs: -0.318, p: 0.340).

**Conclusions:** Preceding staphylococcal osteomyelitis provides survival benefit to subsequent experimental osteomyelitis through down-regulation of innate immune responses leading to efficient phagocytosis.

**Reference**

1. Mandragos E, et al. J Infect 2017; 74:163-171

**Fig. 1 (abstract P032).**
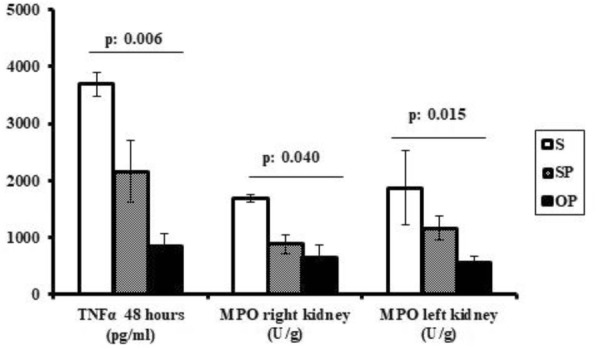
See text for description.

### P033 Synthetic analogue of leu-enkephalin prevents neutrophil activation by bacterial compounds

#### V Likhvantsev^1^, O Grebenchikov^2^, A Prykhodko^1^, A Kuzovlev^2^

##### ^1^Vladimirsky Moscow regional research and clinical center, Moscow, Russia; ^2^Federal Research and Clinical Center of Intensive Care Medicine and Rehabilitology, Moscow, Russia

**Introduction:** Activation of neutrophils is a mandatory step and a sensitive marker of a systemic inflammatory response syndrome (SIRS) which is closely related to development of multiple organ failure. The search for drugs that can prevent SIRS and reduce mortality in critically ill patients remains significant. The aim of this study was to study the anti-inflammatory effect of the synthetic analogue of leu-enkephalin (Dalargin) on human neutrophils.

**Methods:** The study was conducted on isolated from the blood of healthy donors neutrophils. Their activation was assessed by fluorescent antibodies to markers of degranulation CD11b and CD66b (SD11b-FITC and CD66b-AlexaFluor647 (BD Biosciences, USA). As inductors of inflammation lipopolysaccharide (LPS) and the peptide formyl Met-Leu-Pro (fMLP) were used. 100mkM fMLP and dalargin in concentrations of 50 and 100 μ g / ml were added to neutrophils at a concentration of 4 ppm / ml and incubated for 30 min at 37°C; then antibodies were added and incubated for 30 min on ice; then fluorescence was assessed by flow cyto flow meter Beckman-Coulter FC 500. Non-parametric criteria were used; data were presented as a median and 25%—75% interquartile intervals. The statistical significance was estimated using Mann-Whitney test. The difference was considered statistically significant at P<0.05

**Results:** Synthetic analogue of leu-enkephalin in various concentrations has an anti-inflammatory effect on both intact and pre-activated with bacterial components neutrophils, reducing their activation and degranulation in a dose-dependent manner (Figs. 1, 2).

**Conclusions:** Synthetic analogue of leu-enkephalin prevents neutrophil activation by bacterial compounds. This has a potential of translation into clinical practice for sepsis treatment.

**Fig. 1 (abstract P033).**
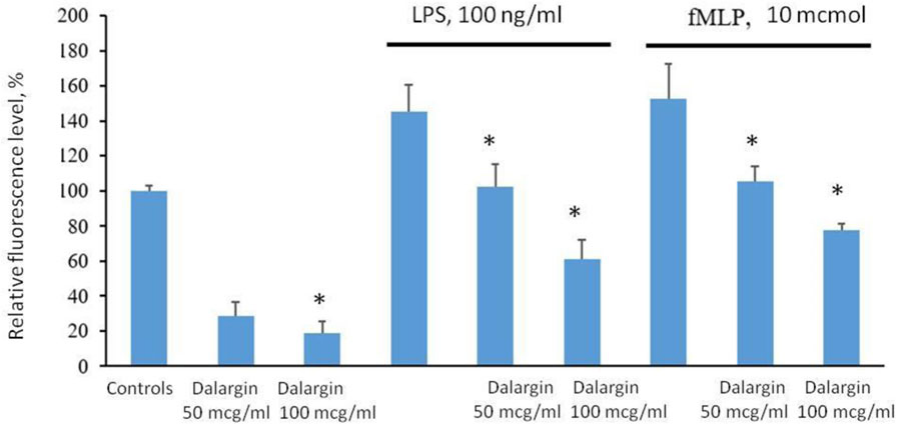
Synthetic analogue of leu-enkephalin reduces the expression of the degranulation marker CD11b in intact and pre-activated human neutrophils (* - significant difference compared to controls, Mann-Whitney test)

**Fig. 2 (abstract P033).**
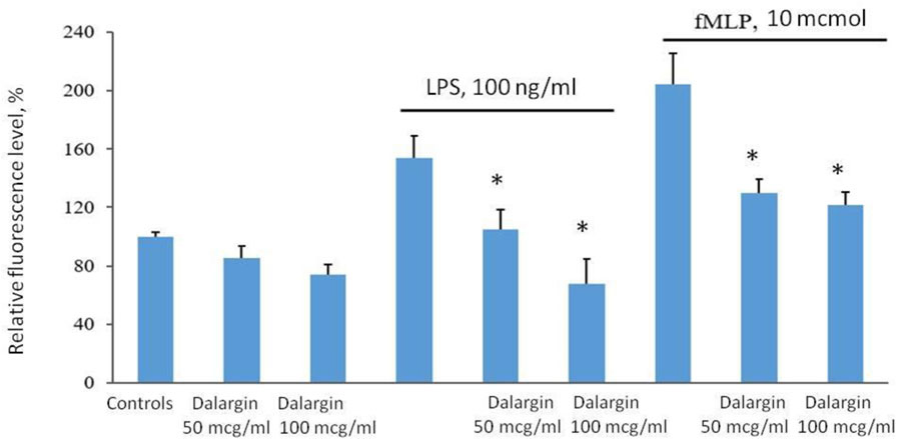
Synthetic analogue of leu-enkephalin reduces the expression of the CD66b degranulation marker in intact and pre-activated human neutrophils (* - significant difference compared to controls, Mann-Whitney test)

### P034 Potential role of endothelin receptors in the therapy of experimental sepsis

#### A Rutai, R Fejes, SZ Tallósy, M Poles, L Juhász, A Mészáros, M Boros, J Kaszaki

##### University of Szeged, Institute of Surgical Research, Szeged, Hungary

**Introduction:** The endothelin system plays important roles in circulatory regulation through vasoconstrictor ET-A and ET-B2 receptors and vasodilator ET-B1 receptors (ETAr; ETBr, respectively). Tissue hypoxia during the progression of sepsis is associated with microcirculatory and mitochondrial disturbances. Our aim was to investigate the possible influence of ETAr antagonist, ETBr agonist or combined treatments on oxygen dynamics, microcirculatory and mitochondrial respiration parameters in experimental sepsis.

**Methods:** Male Sprague-Dawley rats (n=8/group) were subjected to faecal peritonitis (0.6 g/kg faeces ip) or sham-operation. Septic animals were treated with sterile saline solution, or received the ETAr antagonist ETR-p1/fl peptide (100 nmol/kg iv), ETBr agonist IRL-1620 (0.55 nmol/kg iv) or same doses as combination therapy, 22 hr after sepsis induction. Invasive hemodynamic monitoring and blood gas analyses were performed during a 90-min observational window. Intestinal microcirculation (perfusion rate, red blood cell velocity - RBCV) was investigated by intravital videomicroscopy. Complex I and II-linked (CI; CII,) mitochondrial respiration (oxidative phosphorylation - OxPhos) was evaluated by high resolution respirometry (O2k, Oroboros, Austria).

**Results:** The septic reaction was characterized by significant hypotension and decreased microperfusion, oxygen extraction and CI - CII-linked OxPhos values. The ETAr antagonist treatment significantly increased the oxygen extraction, RBCV and CII-linked OxPhos capacity. The ETBr agonist treatment prevented the sepsis-induced hypotension, decrease in oxygen extraction, and significantly increased the perfusion rate. The combined therapy amplified the beneficial mitochondrial and microcirculation effects of selective ETAr antagonist and ETBr agonist compounds.

**Conclusions:** The combination of ETAr antagonism and ETBr agonism may offer a novel tool for a simultaneous microcirculatory and mitochondrial resuscitation strategy in sepsis. Grant supports: NKFIH K116689

### P035 Pharmacokinetics and pharmacodynamics of nivolumab in Japanese patients with immunosuppressive sepsis: determining safety and tolerability in a multicenter, open-label, phase 1/2 study

#### E Watanabe^1^, O Nishida^2^, Y Kakihana^3^, M Odani^4^, T Okamura^5^, T Harada^5^, S Oda^6^

##### ^1^Chiba University Graduate School of Medicine, Department of General Medical Science, Chiba, Japan; ^2^Fujita Health University School of Medicine, Department of Anesthesiology & Critical Care Medicine, Aichi, Japan; ^3^Kagoshima University Graduate School of Medical and Dental Sciences, Department of Emergency and Intensive Care Medicine, Kagoshima, Japan; ^4^Ono Pharmaceutical Co., Ltd., Data Science, Osaka, Japan; ^5^Ono Pharmaceutical Co., Ltd., Clinical Development Planning, Osaka, Japan; ^6^Chiba University Graduate School of Medicine, Department of Emergency and Critical Care Medicine, Chiba, Japan

**Introduction:** Sepsis often induces immunosuppression, which is associated with high mortality rates. Nivolumab is a human IgG-4 antibody directed against the programmed cell death 1 (PD-1) immune-checkpoint inhibitor, which disrupts PD-1-mediated signaling and restores antitumor immunity. Nivolumab is an approved anti-cancer drug that may have the potential to improve sepsis-induced immunosuppression.

**Methods:** This multicenter, open-label study investigated the safety, pharmacokinetics and pharmacodynamics of a single intravenous infusion of 480 or 960 mg nivolumab in Japanese patients with immunosuppressive sepsis (lymphocytes ≤ 1100 /μL). The dosing of nivolumab was set using the predicted steady state concentration of nivolumab at 3 mg/kg every 2 weeks (Q2W), which was the approved dosage for cancer patients at the time of planning.

**Results:** Five and eight patients were assigned to the 480 and 960 mg groups, respectively. The mean (standard deviation) peak serum drug concentration in the 480 mg group was comparable to the predicted median concentration (90% PI [prediction interval]) at the end of infusion with 3 mg/kg Q2W (132 [39.5] μg/mL vs. 117 [56.4-239] μg/mL) (Table 1). In addition, the median (range) concentration on day 28 in the 960 mg group was within the range of the predicted minimum concentration (median [90% PI]) after dosing with 3 mg/kg Q2W (33.1 [6.47-44.8] μg/mL vs. 57.7 [19.0-163] μg/mL). Lymphocyte counts and monocytic human leukocyte antigen DR-1 appeared to increase over time in both groups (Figures 1 and 2). Adverse events (AEs) were observed in four patients in each group. Drug related-AEs were observed in only one patient in the 480 mg group (Table 2). No deaths related to nivolumab occurred.

**Conclusions:** A single dose of 960 mg nivolumab appeared to be well tolerated and sufficient to maintain nivolumab blood concentration in patients with sepsis. Results suggest both 480 and 960 mg nivolumab therapy could improve relevant immune indices.

**Table 1 (abstract P035).** See text for description

Pharmacokinetic parameters	480 mg single dose (n=5)	960 mg single dose (n=8)	3 mg/kg Q2W* (predicted value)
C_max_, mean (SD), μg/mL	132 (39.5)	195 (46.9)	
C_28d_, n	4	5	
C_28d_, median (range), μg/mL	14.3 (6.51-40.1)	33.1 (6.47-44.8)	
C_eoi_ at steady state**, μg/mL			117 (56.4-239)
C_min_ at steady state**, μg/mL			57.7 (19.0-163)

*Predicted values calculated from a population pharmacokinetic model (n=1000) based on ten clinical studies that included 187 Japanese cancer patients. **Median (90% prediction interval). C28d, serum drug concentration on Day 28; Ceoi, end of infusion drug concentration; Cmax, maximum (peak) serum drug concentration; Cmin, minimum (trough) serum drug concentration; SD, standard deviation; Q2W, every two weeks

**Table 2 (abstract P035).** See text for description

Drug-related adverse events (Grade ≥ 2)	Grade	480 mg nivolumab (n=5)	960 mg nivolumab (n=8)
Alanine aminotransferase increased	4	1 (20.0)*	0 (0.0)
Aspartate aminotransferase increased	3	1 (20.0)*	0 (0.0)
Pruritus	2	1 (20.0)*	0 (0.0)
Rash	2	1 (20.0)*	0 (0.0)
Organizing pneumonia	2	1 (20.0)*	0 (0.0)

n (%), *All drug-related adverse events were observed in the same patient

**Fig. 1 (abstract P035).**
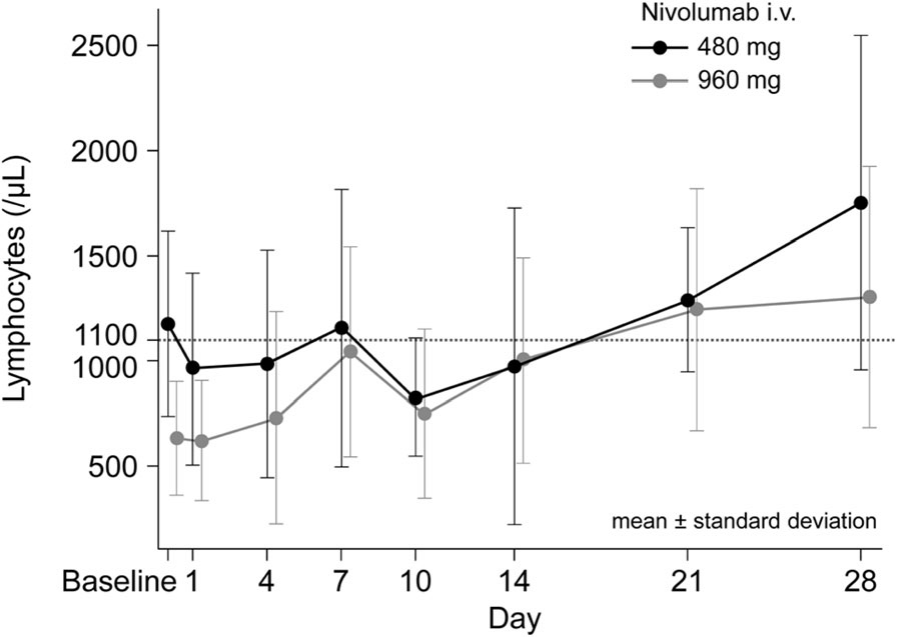
Change in lymphocyte count over time

**Fig. 2 (abstract P035).**
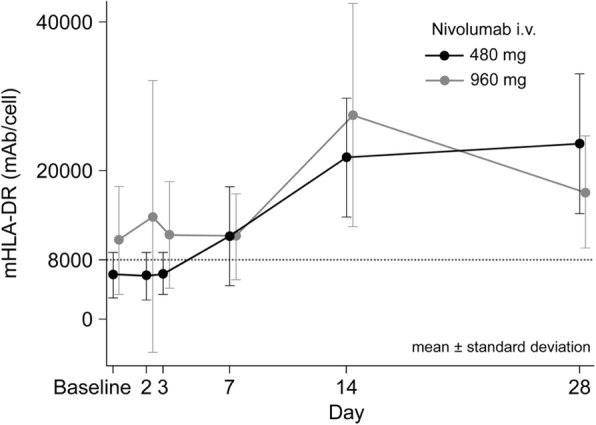
Change in monocyte HLA-DR antigen expression over time

### P036 Biological effects of low dose aspirin in critically ill patients with the systemic inflammatory syndrome (SIRS): a pilot feasibility multi-center randomised placebo-controlled trial (PROTECTIN trial)

#### N Luethi^1^, L Cioccari^1^, G Eastwood^1^, L Peck^1^, H Young^1^, SL Cutuli^1^, P Lloyd-Donald^1^, M Bailey^2^, C French^3^, N Orford^4^, J Dwivedi^5^, T Duong^6^, E Ryan^6^, G Reid^6^, R Bellomo^1^

##### ^1^Austin Hospital, Intensive Care Unit, Heidelberg, Australia; ^2^Australian and New Zealand Research Centre, Melbourne, Australia; ^3^Western Health, Intensive Care Unit, Footscray, Australia; ^4^University Hospital Geelong, Intensive Care Unit, Geelong, Australia; ^5^Bankstown Hospital, Intensive Care Unit, Bankstown, Australia; ^6^University of Melbourne, School of Chemistry, Department of Biochemistry and Molecular Biology, Melbourne, Australia

**Introduction:** The systemic inflammatory response syndrome (SIRS) accompanies tissue trauma and infection and, when severe or dysregulated, contributes to multiple organ failure and critical illness. Observational studies in man and animal have shown that low-dose acetyl-salicylic acid promotes resolution of inflammation and might attenuate excessive inflammation by increasing the synthesis of specialised pro-resolving lipid mediators (SPMs).

**Methods:** We randomly assigned patients with SIRS who were expected to stay in ICU for more than 48 hours to receive enteral aspirin (200 mg per day) or placebo for 7 days or until death or discharge from the ICU, whichever came first. The primary outcome was IL-6 serum concentration at 48h after randomisation. The secondary outcomes included safety and feasibility outcomes. In one center, additional blood samples were taken during the first three days for exploratory analysis of SPMs using reversed-phase high-performance liquid chromatography - tandem mass spectrometry (RP-HPLC-MS/MS).

**Results:** From March 2015 through December 2017 a total of 48 patients across four general ICUs in Australia underwent randomization (Table 1). Compared to placebo patients, IL-6 serum concentration after 48h in aspirin-treated patients was not significantly lower (40 [16-166] pg/ml vs 44 [7.4-85] pg/ml; p=0.66). There were no significant differences for control vs. aspirin-treated patients in the change of pro-resolving/anti-inflammatory lipids between the time points (Figure 1, 2). There were no between-group differences with respect to ICU or hospital mortality, number of bleeding episodes or requirements for red cell transfusions (Table 2).

**Conclusions:** In patients admitted to the ICU with SIRS, low-dose aspirin did not result in a decreased concentration of inflammatory biomarkers compared with placebo.

**Table 1 (abstract P036).** See text for description

Baseline characteristics	Aspirin (n=23)	Placebo (n=25)	p-value
Age, years [IQR]	60 [41-71]	60 [46-73]	0.64
Male sex – no. (%)	15 (65.2)	13 (48.0)	0.35
APACHE III score	53 [47-62]	52 [42-64]	0.87
Sepsis – no. (%)	19 (82.6)	21 (84)	0.92
Mechanical ventilation – no. (%)	15 (65.2)	14 (56.0)	0.51
Interleukin-6 level, pg/ml	94 [44-376]	121 [39-459]	0.57
C-reactive protein, mmol/L	130 [48-228]	143 [16-307]	0.99

Data presented as number (percentage) and median [interquartile range]

**Table 2 (abstract P036).** See text for description

Secondary Outcomes	Aspirin (n=23)	Placebo (n=25)	p-value
Length of ICU stay, days	4.1 [2.2-8.7]	4.9 [3.4-8]	0.37
ICU mortality – no (%)	0	2 (8)	0.49
Hospital mortality – no (%)	1 (4.3)	2 (8.0)	>0.99
28-day mortality – no. (%)	1 (4.3)	2 (8.0)	>0.99
Patient with transfusion in ICU – no. (%)	3 (13.0)	3 (12.0)	>0.99
Episodes of clinically identified bleeding in ICU	0	0	-
Lowest platelet count in ICU, x1000/mm3	186 [112-228]	164 [120-238]	0.88

Data presented as number (percentage) and median [interquartile range]

**Fig. 1 (abstract P036).**
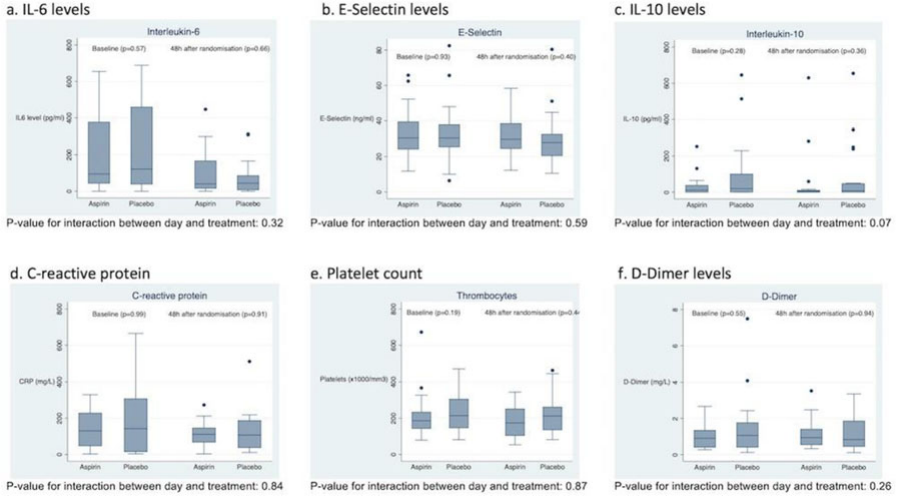
Boxplots of biomarker levels at baseline and 48h after randomisation

**Fig. 2 (abstract P036).**
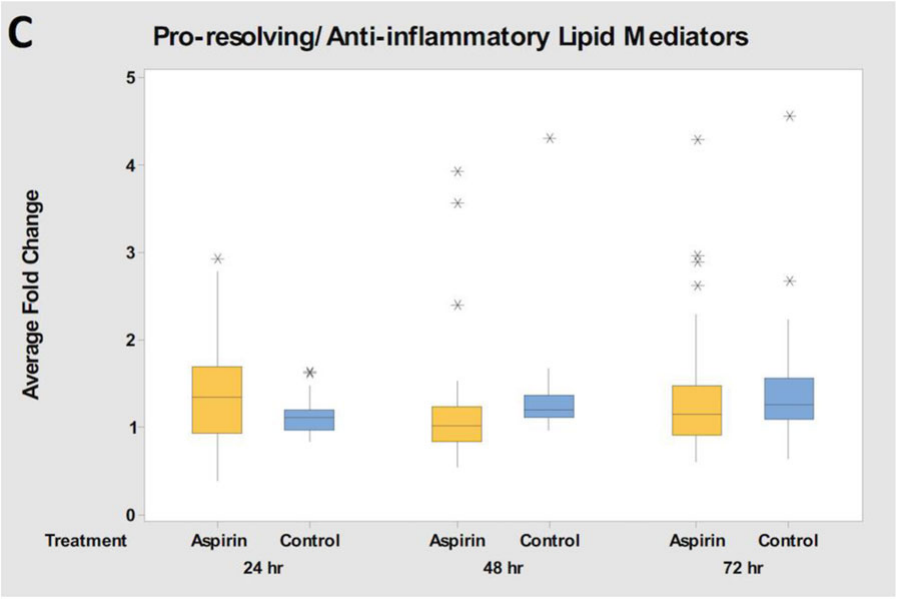
Boxplots of the fold change of the total pro-resolving/anti-inflammatory lipid mediators (28 lipids) for control and aspirin-treated patients

### P037 Ketone body 3-hydroxybutyrate protects against muscle weakness and stimulates muscle regeneration in septic mice

#### C Goossens, S Derde, S Vander Perre, R Weckx, G Van den Berghe, L Langouche

##### KU Leuven, Laboratory of Intensive Care Medicine, Leuven, Belgium

**Introduction:** Debilitating muscle weakness and impaired muscle regeneration is prevalent in ICU patients. Remarkably, premorbid obesity has been shown to protect against this weakness in both ICU patients and septic mice, which coincided with markers of elevated ketogenesis [1]. We here assessed whether ketone body supplementation could directly protect the muscle during sepsis.

**Methods:** In a resuscitated, antibiotic-treated mouse model of prolonged (5 days) abdominal sepsis (cecal ligation and puncture), lean ill mice received standard parenteral nutrition (5.8kcal/day) supplemented with D,L-3-hydroxybutyrate (PN+3HB; 150mg/day; n=17) or isocaloric glucose (PN+gluc; 187.5mg/day; n=17). Pair-fed healthy mice served as controls (n=15). Markers of muscle weakness and regeneration were assessed.

**Results:** Compared to controls, absolute maximal EDL muscle force was reduced with 44% in PN+gluc septic mice (p<0.0001) and with 29% in PN+3HB septic mice (p=0.0001 vs. controls and p=0.002 vs. PN+gluc). Specific maximal muscle force, which is corrected for muscle mass, was reduced with 29% in PN+gluc septic mice compared to controls (p=0.0001), whereas PN+3HB septic mice maintained their specific maximal muscle force up to control levels (p=0.1 vs. controls and p=0.01 vs. PN+gluc), implying preservation of muscle quality, but not quantity with 3HB. Furthermore, PN+3HB increased gene expression of regeneration marker Myod1 (p≤0.03 vs. PN+gluc and controls), and of myogenic regulatory factors Myog and Myf5 (p<0.05 vs. PN+gluc). Stimulation of regeneration markers with PN+3HB coincided with a decreased expression of Hdac4, Hdac5 and upregulation of downstream gene Mef2c (known to stimulate muscle regeneration; p<0.05 vs. PN+gluc).

**Conclusions:** PN+3HB in lean septic mice protected against muscle weakness and elevated muscle regeneration markers. These data identify nutritional 3HB supplementation as a potential preventive therapy for muscle weakness, requiring further investigation.

**Reference**

[1] Goossens et al. JCSM 8:89-101, 2017

### P038 Vitamin C and thiamine in septic shock: a retrospective before-after analysis.

#### A Casazza^1^, U Suppo^2^, M Delorenzo^2^, L Marinelli^2^, S Testa^2^, E Bellazzi^1^, R Boschi^1^, D Ciprandi^1^, C Gigliuto^1^, R Preda^1^, R Vanzino^1^, M Vetere^1^, L Carnevale^1^

##### ^1^ASST Pavia, Anaesthesia and Intensive Care Vigevano, Vigevano, Italy; ^2^Università degli Studi di Pavia, Scuola Specialità MEU, Pavia, Italy

**Introduction:** Sepsis is associated with excessive ROS production, NF-kB, iNOS and inflammatory mediators overexpression. Vitamin C is a cellular antioxidant, it increases eNOS and decreases NF-kB; it has several immune-enhancing effects and is crucial for endogenous vasopressors synthesis. Vitamin C reserves in sepsis are often as poor as in scurvy [1]. In recent studies, intravenous high Vitamin C dose seems to reduce organ failure and improve outcome in septic shock.

**Methods:** We treated all septic shock patients admitted to our ICU in 7 months (from 3/2018 to 9/2018) with intravenous Vitamin C 1.5g/6h and Thiamine 200 mg/12h (for its synergistic effects) [2] as adjunctive therapy for 4 consecutive days and we compared data to septic shock patients admitted in the previous 7 months period. We enrolled 24 patients: 13 received Vitamins supplementation, 11 standard of care. We analysed 28-days mortality, SOFA at 48 and 96 hours, PCT variation from baseline in first 5 days, vasoactive therapy length and DAF 28 (Days Alive and Free from vasopressors, mechanical ventilation and RRT in 28 days follow up). Patients with end stage kidney disease were ruled out. We analysed data with Mann-Whitney and Wilcoxon tests.

**Results:** Vit C group showed lower 28-days mortality (23% vs 54.5%: NS); SOFA improvement at 48 (-2.4±1.5 vs -0.9±1.9: p=0.01) and 96 hours (-4.2±2 vs -1.9±2: p<0.01) was higher in Vit C group; Vit C patients had faster PCT reduction without statistical significance. Mean vasoactive therapy length was quite similar. DAF was 16.5 (±9.1) days in Vit C group and 9.3 (±8.9) in controls (p=0.03). 3 control patients needed RRT, none in Vit C group.

**Conclusions:** Despite small study size, we found that Vit C has positive effects on survival and improves SOFA score (Fig.1) and DAF (Fig.2) in septic shock. No Vit C patient developed oxalate nephropathy nor worsened renal function.

**References**

1. Marik PE et al. Crit Care 22:23, 2018

2. Marik PE et al. Chest 151:1229-1238, 2017

**Fig. 1 (abstract P038).**
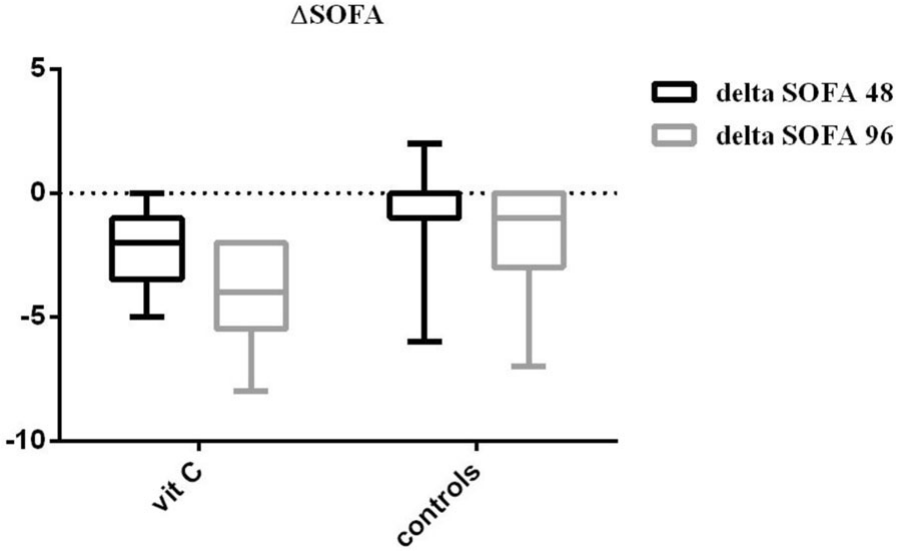
SOFA variation

**Fig. 2 (abstract P038).**
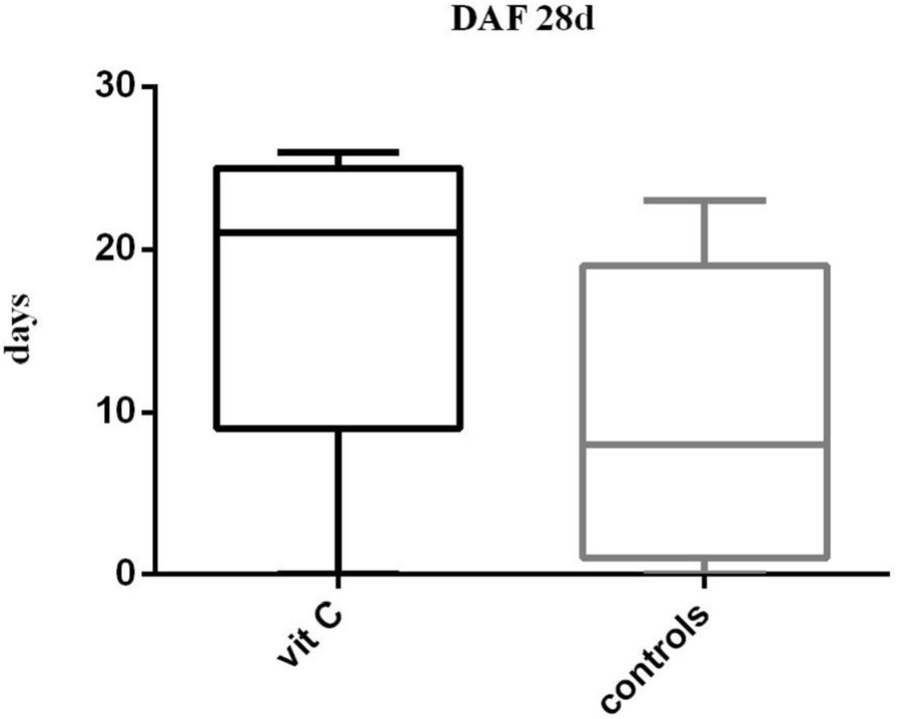
DAF variation

### P039 A double- blind, randomized, placebo-controlled clinical study of the efficacy of intravenous clarithromycin as adjunctive treatment in patients with sepsis and respiratory and multiple organ dysfunction syndrome: INCLASS study

#### E Karakike^1^, M Roumpoutsou^1^, N Karampela^2^, E Massa^3^, E Antypa^4^, K Psaroulis^5^, P Chaloulis^6^, E Pappa^7^, V Kolonia^8^, EJ Giamarellos-Bourboulis^1^, I Tsangaris^9^

##### ^1^National and Kapodistrian University of Athens, 4th Department of Internal Medicine, Athens, Greece; ^2^Korgialeneio Benakeio General Hospital, Intensive Care Unit, Athens, Greece; ^3^Hippokration General Hospital, Intensive Care Unit, Thessaloniki, Greece; ^4^G. Gennimatas General Hospital, Intensive Care Unit, Thessaloniki, Greece; ^5^Aghios Dimitrios General Hospital, Intensive Care Unit, Thessaloniki, Greece; ^6^Theageneion General Hospital, Intensive Care Unit, Thessaloniki, Greece; ^7^Laiko General Hospital, Intensive Care Unit, Athens, Greece; ^8^Sismanogleion General Hospital, 2nd Department of Internal Medicine, Athens, Greece; ^9^National and Kapodistrian University of Athens, 2nd Department of Critical Care Medicine, Athens, Greece

**Introduction:** In the light of new insight on pathogenesis of sepsis and after inconclusive randomized clinical trials (RCTs), the benefit of macrolides as adjunctive, low-cost and promising molecules in sepsis, remains to be assessed. The INCLASS study (clinicaltrials.gov NCT: 03345992) is an ongoing RCT aiming to evaluate clarithromycin as immune modulator in high-risk septic patients.

**Methods:** Adult patients with sepsis according to Sepsis-3 definitions with respiratory failure (defined as PaO_2_/FiO_2_< 200) and total SOFA score equal to or more than 7 can be enrolled provided that they present with one of the following infections: hospital-acquired pneumonia; healthcare-associated pneumonia (HCAP); ventilator-associated pneumonia (VAP); intra-abdominal infections (IAIs) and primary gram-negative bacteremia . Patients are blindly randomized to receive either 1gr of intravenous clarithromycin or placebo once daily for four consecutive days. The primary endpoint is survival at 28 days. The study is powered for 110 patients.

**Results:** Sixty-nine patients have been enrolled so far. The most common infections are VAP 33%, IAI 22% and HCAP 20%. Most common isolated pathogens are *Acinetobacter baumannii* 37%; *Klebsiella pneumoniae* 27% and *Escherichia coli* 20%. Mean ± SD Charlson’s comorbidity index is 5.0 ± 2.7 and APACHE score 21.9 ± 6.6. SOFA score on enrollment is 10.4 ± 2.5.

**Conclusions:** The INCLASS study is an on-going pragmatic trial enrolling very severe patients with high SOFA score.

### P040 IVIG administration in ECMO patients with toxin-mediated shock

#### M Peetermans, R Wan, C Meadows, N Ioannou, G Glover, L Camporota, D Wyncoll, N Barrett, A Retter

##### St. Thomas´ Hospital, London, Critical Care, London, United Kingdom

**Introduction:** Toxin-producing gram-positive organisms cause some of the most severe forms of septic shock [1,2]. Adjunctive therapies such as intravenous immunoglobulins (IVIG) have been proposed for these patients [3,4]. However, at patient presentation, the presence of a toxin-producing organism is most often unknown.

**Methods:** We reviewed the use of IVIG in our patients requiring extracorporeal membrane oxygenation (ECMO) in a 2-year period between February 2016 and March 2018.

**Results:** In 44% (15/34) of the patients that received IVIG for presumed toxin-mediated shock, group A *Streptococcus* or Panton-Valentine leukocidin producing *S. aureus* was isolated, but the clinical characteristics of these 15 patients were not significantly different from the ones with other final diagnoses, except for a predisposing influenza infection and the presence of an often very high procalcitonin level. These 34 patients were extremely unwell at presentation with a SOFA score of 15 ± 3, high lactate levels (8.6 ± 5.8 mmol/L) and need for vasopressors (equivalent norepinephrine dose of 0.93 ± 0.62 μ g/kg/min). They had very high inflammatory parameters with a procalcitonin ≥ 100 ng/mL in more than half of patients (18/34). IVIG use in these patients was generally safe, with only 1 possible transfusion reaction. The mortality of 35% (12/34) was lower than predicted based on the SOFA scores.

**Conclusions:** IVIG administration can be considered in a selected group of patients presenting with acute and very severe septic shock, as part of a multimodal approach [5].

**References**

1. Burnham JP and Kollef MH. Intensive Care Med 2015;41:1707-10.

2. Gillet Y, et al. Lancet, 2002;359:753-9.

3. Alejandria MM, et al. Cochrane Database Syst Rev 2013:CD001090.

4. Shankar-Hari M, et al. Crit Care 2012;16:206.

5. Nandhabalan P, et al. Crit Care 2018;22:215.

### P041 Immunological effect of the adsorbing membrane oXiris on septic patients with AKI

#### F Turani^1^, S Busatti^1^, V Cotticelli^1^, R Barchetta^1^, F Leonardis^2^, F Candidi^1^, M Dauri^2^

##### ^1^Aurelia And European Hospital, Anesthesia And Intensive Care, Rome, Italy; ^2^University Of Tor Vergata, Anesthesia And Intensive Care, Rome, Italy

**Introduction:** Extra corporeal treatments are used in septic patients to decrease the inflammatory mediators, but definitive conclusions are lacking . More over in many studies the effect of AKI isn’t evaluated and this may be an important bias. . The aim of this study is to evaluate in septic patients with AKI: 1- the effect of the adsorbing membrane Oxiris on the immunological response 2- the different response in survivors and non survivors

**Methods:** From our local data base we analyzed retrospectively 50 septic shock patients with AKI (KDIGO classification) submitted to CRRT with the adsorbing membrane oXiris (Baxter, USA ) . At basal time ( T0 ) and at the end of the treatment ( T1 ) we evaluated the following variables: IL 6 IL 10 Procalcitonin Endotoxin (EAA). All data are expressed as mean ±SD or median and IQR. Student T test or Mann- Whitney was used to compare values changes. P < 0.05 was considered statistically significant.

**Results:** Thirty patients with sepsis /septic shock and AKI were enrolled in this study. 10 patients had AKI 3, 15 patients AKI 2, 5 patients AKI 1. The duration of treatment was 38± 10 hours. 20 patients had citrate as anticoagulation and 10 heparine continous ev. At Table 1 are shown the main results of this study in all the patients. Survivors vs non survivors had a significant decrease of IL 6, Procalcitonin and EAA.

**Conclusions:** Data of this study confirm on clinical ground previous study “in vitro” [1] that the adsorbing membrane oXiris has important immunological effect during septic shock with AKI. This must be confirmed in a RCT.

**Reference**

1 Malard et al. Intensive Care Medicine Experimental (2018) 6:12

**Table 1 (abstract P041).** Septic patients with AKI

	T0	T1
IL6 pg/mL	367±44	126±52**
IL 10 pg/mL	106±57	28±17*
Procalcitonin ng/ml	35±14	8±4*
EAA	0.74±0.15	0.58±0.18**

** p < 0.001 * p <0.05

### P042 Hemoadsorption (CytoSorb) therapy in septic shock and MODS patients - a prospective multi-centre investigator initiated study

#### R Paul^1^, P Sathe^2^, R Senthil^3^, S Prasad^4^, M Aleem^1^, S Prashant^2^

##### ^1^Apollo Health City, Department of Internal Medicine and Critical Care, Telangana State, India, India; ^2^Ruby Hall Clinic, Department of Critical Care Medicine, Pune, India; ^3^Apollo Hospital, Greams Lane, Department of Critical Care Medicine, Chennai, India; ^4^Narayana Institute of Cardiac Sciences, Department of Anesthesiology and Critical Care, Bangalore, India

**Introduction:** Sepsis is common and often fatal, representing a major public health problem. Hemoadsorption (CytoSorb) therapy aims to reduce cytokines and stabilise the overall immune response in septic shock patients.

**Methods:** A prospective, multi-centre, investigator initiated study to evaluate Hemoadsorption (CytoSorb) Therapy in septic shock patients admitted to a tertiary ICU’s in India during 2016 to 2018. All centres followed a common protocol and received ethics committee approval.

**Results:** A total of 45 patients were administered CytoSorb in addition to standard of care. A total of 26 patients (62%) survived out of 45 patients. Among survival group, 17 patients (71%) were administered CytoSorb within 48 hours of ICU admission resulting in significant reduction in Sepsis Scores, APACHE II (24.42 vs 19.33) and SOFA (12.46 vs 8.71) post CytoSorb therapy. Also there was reduction in inflammatory markers like Cytokines IL6 in most of the patients. All patients in survivor group showed a significant improvement in MAP (69.2 vs 76.8) and reduction in vasopressors (Epinephrine 0.3 to 0.03 mcg/kg/min, Nor-Epinephrine 0.34 to 0.04 mcg/kg/min) after CytoSorb therapy. No device related adverse effect was observed in any of the patients. Among the non-survivor group, (16 patients, 38%) we observed that CytoSorb was administered after 48 hours of ICU admission. Although a few patients showed improvement in SOFA score, majority did not show a significant improvement with MAP (68.05 vs 60.4 mm of Hg) and required increased demand in Vasopressors.

**Conclusions:** In this multi-centered prospective IIS study, we could observe clinical benefits of Hemoadsorption (CytoSorb) therapy in Septic shock patients if the therapy was initiated early. Larger randomised study are required to establish the above clinical benefits in larger patient population.

### P043 A single centre experience with hemoadsorption (CytoSorb) in varied causes of sepsis and MODS

#### Y Mehta^1^, C Mehta^1^, A Kumar^1^, J George^2^, A Gupta^3^, S Nanda^1^, G Kochar^1^, A Raizada^3^

##### ^1^Medanta The Medicity, Institute of Critical Care and Anesthesiology, New Delhi, India; ^2^Medanta The Medicity, Institute of Critical Care and Anesthesiology, Clinical Co-Ordinator, New Delhi, India; ^3^Medanta The Medicity, Department of Biochemistry, New Delhi, India

**Introduction:** Sepsis and the multiorgan failure is a leading cause of mortality in the intensive care unit. Promising new therapies continue to be investigated for the management of septic shock. We tried to evaluate a novel Hemoadsorption therapy (CytoSorb) through a retrospective evaluation of patient’s data in our centre. We used it as an adjuvant therapy in our patients with Sepsis due to varied causes.

**Methods:** We retrospectively analysed data of 100 Sepsis & Septic shock patients admitted between 2016 to 2018, who had received CytoSorb as adjuvant therapy along with standard of care. Institutional ethics committee approval was taken before initiating the study.

**Results:** A total of 100 patients (77 Male and 23 Females) with a mean age of 52.53 years were administered CytoSorb in addition to standard of care. A total of 40 patients survived out of 100 patients. Among 40 patients who survived 28 patients (70%) were administered CytoSorb within 48 hours of ICU admission. There was a significant reduction in scores like APACHE (26.4 vs 18.02) and SOFA (15.05 vs 10.32) post CytoSorb therapy. All the survivors showed a significant improvement in MAP (62.8 vs 68.2) and reduction in vasopressors need (Epinephrine 0.1 to 0.05 mcg/kg/min, Nor-Epinephrine 0.5 to 0.05 mcg/kg/min) after CytoSorb initiation. Out of 60 Patients who didn’t survive, 43 patients (72%) received CytoSorb therapy after 48 hours of ICU admission. In majority of these patients, there was no improvement of SOFA Score, MAP and increased need of Vasopressor demand as compared to the survival group.

**Conclusions:** Retrospective analysis showed significant reduction of vasopressors, Sepsis Score and improvement in MAP in survived group versus non-survived group. Looking into the positive outcome of this case series, randomized controlled studies are required to define the potential benefits of this new treatment option.

### P044 Extracorporeal cytokine removal in septic shock patients: two-years experience

#### A Gulleroglu, E Gedik, H Sahinturk, A Ozdemirkan, P Zeyneloglu, A Pirat

##### Baskent University Faculty of Medicine, Anesthesiology and Critical Care, Ankara, Turkey

**Introduction:** Septic shock is a life-threatening multiple organ dysfunction that has high morbidity and mortality in critically ill patients, due to a dysregulated host response to infection. The aim of this study was to evaluate the efficacy of therapeutic cytokine removal (CytoSorb®) in the management of patients with septic shock.

**Methods:** We retrospectively analyzed patients admitted to ICU with septic shock between June 2015 and November 2017. Patients included in the study were diagnosed according to The Third International Consensus Definitions for Sepsis and Septic Shock (Sepsis-3), received maximal supportive care including continuous veno-venous hemodiafiltration (CVVHDF) for acute kidney injury and Cytosorb® haemoadsorption column was added to return limb of the CVVHDF circuit. Demographic data, procalcitonin and leukocyte levels before and after therapeutic cytokine removal and duration of Cytosorb® haemoadsorption column application and APACHE II scores were recorded.

**Results:** The mean age of 48 patients included in the study was 54±16.4 years (74% male) and the mean body mass index was 23.1±5.9. The mean APACHE II score was 21.5 with an expected and actual mortality rates of 40% and 25%, respectively. 18% of the patients were admitted with sepsis and 82% of them with septic shock. 37.5% (n=18) of the cases were solid organ transplant recipients. CVVHDF was applied in all patients during therapeutic cytokine removal. Treatment was combined with ECMO in 8 patients. While the mean duration of CVVHDF was 102.1 hours, the duration of Cytosorb® haemoadsorption column application was 24.1±13.4 hours. Procalcitonin (16.5 ± 25ng/ml vs 25±34ng/ml) and leucocyte levels (14048±10490/mm3 vs 9278±8693 mm3) after therapeutic cytokine removal were found significantly lower than the pretreatment values (respectively p=0.0013, p=0.006).

**Conclusions:** Therapeutic cytokine removal applied with CVVHDF in septic shock patients have positive contributions to biochemical parameters and provide survival advantage.

### P045 Potential benefits of a hemoadsorption column in septic ICU patients

#### M Popescu, A Marcu, C David, D Tomescu

##### Fundeni Clinical Institute, Anaesthesia and Critical Care, Bucharest, Romania

**Introduction:** Recent studies have focused on demonstrating the potential benefits of immunomodulation in the management of septic patients. The aim of our study was to assess the effects of a hemoadsorption column (CytoSorb®) in critical ill septic patients.

**Methods:** After ethical approval was obtained, we prospectively included 39 patients admitted to the general ICU of Fundeni Clinical Institute. Three consecutive sessions of renal replacement therapy (continuous venovenous hemodiafiltration) in combination with CytoSorb® were applied after ICU admission. Clinical (heart rate, arterial pressure, temperature, Glasgow coma scale) and paraclinical data (PaO2, serum bilirubin and creatinine, platelet count, white blood cell count, pH, C-reactive protein and procalcitonine), vasopressor support and need for mechanical ventilation were recorded before and after the three sessions.

**Results:** The mean age in the study group was 57±15 years. Median number of organ dysfunction at the time of ICU admission was 4 [1-5] and the mean SOFA score was 10.0±3.7. The use of CytoSorb® was associated with a non-significant increase in PaO2/FiO2 ratio from 216±80 to 248±94 (p=0,278) and creatinine levels from 1.8±1.3 to 1.6±1.2 mg/dL (p=0.685). Although we observed a non-significant increase in C-reactive protein levels from 136±66 mg/L to 144±72 mg/L (p=0.577), we noted a significant decrease in procalcitonine levels from a median of 20.0 [2.2, 100.0] ng/dL to a median of 9.1 [0.7, 55.2] ng/dL (p=0.041). A significant decrease in platelet count was also noted from 135384±99263 /mm3 to 78470±61624 /mm3 (p=0.002). Mean SOFA score decreased non-significantly from 10.0±3.7 to 9.1±4.1 (p=0.533).

**Conclusions:** The use of CytoSorb was associated with a slight non-significant improvement in organ function and a decrease of procalcitonine levels. Thrombocytopenia remains one of the most important complications of renal replacement therapy.

### P046 Removal of circulating NETs-related components with an immobilized polymyxin B filter

#### N Takeyama, T Gocho, Y Maruchi, N Takenaka, H Mori, M Islam, MA Huq

##### Aichi Medical University, Department of Emergency and Critical Care Medicine, Aichi, Japan

**Introduction:** Circulating cell-free neutrophil extracellular traps (NETs) would induce a microcirculatory disturbance of sepsis. The removal of NETs remnants from the circulation could reduce NETs-dependent tissue injury. To address this issue, we evaluated the effect of hemoperfusion with a polymyxin B cartridge (PMX-DHP; Toray, Japan), which was originally developed for the treatment in patients with Gram-negative bacterial infection, on circulating cell-free NETs in patients with septic shock and in phorbol myristate acetate (PMA)-stimulated neutrophils obtained from healthy volunteer.

**Methods:** Ex vivo closed loop hemoperfusion was performed through a circuit formed by connecting the small PMX module to a tube and a peristalsis pump. Whole blood from healthy volunteers incubated with or without PMA or from septic shock patients were applied to circuit and perfused. Blood was collected at 0, 1 and 2 hr after perfusion. Circulating cell-free NETs were assessed by myeloperoxidase (MPO)-, neutrophil elastase (NE)-, and cell free (cf)-DNA.

**Results:** Plasma MPO-DNA, NE-DNA and cf-DNA levels were significantly increased at 2 hr after PMA stimulation when compared with plasma levels without PMA. When either blood from septic shock patients or PMA-stimulated neutrophils obtained from volunteers were applied to circuit, circulating MPO-DNA, NE-DNA and cf-DNA were significantly reduced in perfusion with PMX filter than in perfusion without PMX filter at times 1 and 2 hr.

**Conclusions:** In the ex vivo experiments, MPO-DNA, NE-DNA and cf-DNA were found to decrease after ex vivo perfusion through PMX filters. Selective removal of circulating components of NETs may improve the remote organ damage in patients with septic shock.

### P047 A retrospective study of septic shock patients who were treated with direct hemoperfusion with polymyxin B-immobilized fibers based on the levels of endotoxin activity assay

#### S Sekine, H Imaizumi, I Saiki, A Okita, H Uchino

##### Tokyo Medical University, anesthesiology/ICU, Tokyo, Japan

**Introduction:** The purpose of this study was to evaluate the outcomes for septic shock patients with direct hemoperfusion with polymyxin B-immobilized fibers (PMX-DHP) and endotoxin activity assay (EAA).

**Methods:** According to the levels of EAA, 41patients were classified for three groups (low group (GL); EAA <0.4, intermediate group (GM); EAA >0.4 or EAA <0.6, high group (GH); EAA >0.6). In order to evaluate the severity of illness, acute physiology and chronic health evaluationII (APACHE II) score, the sequential organ failure assessment (SOFA) score, catecholamine index (CAI) were recorded. And the presence of PMX-DHP treatments were also recorded. Blood samples were obtained to measure EAA levels, inflammatory markers (procalcitonin (PCT), C-reactive protein (CRP), and white blood cell count (WBC)), serum lactate level as an indicator of tissue hypoxia, and for blood culture. APACHE II score, SOFA score, CAI, inflammatory markers, serum lactate levels (Lac) and blood culture results were examined for diagnosis of septic shock and prognosis of 30-days mortality. Each values were also compared to EAA levels.

**Results:** 41 septic shock patients were included (GL/ GM/ GH: 12/ 13/ 16). In GH, APACHE II and SOFA score was significantly higher than that in GL (p< 0.05). EAA levels were significantly increased in gram-negative bacteremia patients compared to the patients with gram-positive bacteremia or fungemia. There was no relationship between EAA levels and other inflammation markers, CAI, and Lac. In GM, 30-days mortality in patient with PMX-DHP treatments was lower than that of without PMX-DHP treatments (0.14 (1/7) vs 0.5 (3/6), p=0.27). In GH, 30-days mortality in patient with PMX-DHP treatments was same as that of without PMX-DHP treatments (0.5 (3/6) vs 0.5 (5/10), p=1.0).

**Conclusions:** These results of this study suggest PMX-DHP treatment may improve the outcome of septic shock patients with intermediate EAA levels.

### P048 Exploring alternative trial designs for pragmatic clinical studies: a Bayesian decision-theoretic model applied on a real ongoing one-stage trial

#### E Karakike^1^, A Alban^2^, EJ Giamarellos-Bourboulis^1^, SE Chick^2^

##### ^1^National and Kapodistrian University of Athens, 4th Department of Internal Medicine, Athens, Greece; ^2^INSEAD, Technology and Operations Management, Fontainebleau, France

**Introduction:** Numerous inconclusive randomized clinical trials (RCTs) in sepsis in the past years suggest a need to re-think trial design to improve resource allocation and facilitate policy adoption decisions. The INCLASS study (clinicaltrials.gov NCT: 03345992) is an ongoing RCT evaluating clarithromycin as an immune modulator in high-risk septic patients with clinical and cost-effectiveness outcomes. We aim to compare the original one-shot trial with an alternative sequential design that balances trial costs and value of information.

**Methods:** Adult patients with sepsis, respiratory failure and total SOFA score of at least 7, are randomized to receive intravenous clarithromycin or placebo adjunctive to standard-of-care therapy. For the cost-effectiveness study, efficacy is measured in Quality-Adjusted Life Years (QALYs) by EQ-5D-3L questionnaire at 90 days. The endpoint is the Incremental Net Monetary Benefit (INMB) of clarithromycin compared to placebo, defined as WTP x (Increment in QALY) – (increment in costs), where WTP is willingness to pay per QALY gained. Fixed and variable costs of trial execution (including administrative, insurance, supplies, tests) are calculated; hospitalization cost is extracted from patient records; medical care beyond day 28 is recorded; cost of adoption in the general population is estimated. Previous data from RCTs using clarithromycin are used to form a prior belief about the INMB. Known incidence of sepsis with respiratory failure allows estimation of the population to benefit from trial decision. A Bayesian model is used to determine the sequential design that maximizes trial value.

**Results:** We will compare the performance of the sequential trial design with the one-shot design of INCLASS trial in terms of sample size, cost, social-welfare, and probability of correctly identifying the best treatment.

**Conclusions:** In this protocol we validate a Bayesian model for sequential clinical trials and assess the benefits for the patient population and health care system.

### P049 The effect on the outcome of critically ill patients with catecholamine resistant septic shock and acute renal failure through implementation of adsorption therapy

#### G Schittek^1^, H Simonis^1^, O Huhn^2^, J Soukup^2^, J Soukup^2^

##### ^1^Medical University of Graz, Department of Anesthesiology and Intensive Care Medicine/ Division of General Anesthesiology and Intensive Care Medicine, Graz, Austria; ^2^Carl-Thiem-Klinikum, Klinik für Anästhesiologie, Intensivtherapie und Palliativmedizin, Cottbus, Germany

**Introduction:** CytoSorb-Adsorption has been described as an effective way for hemodynamic stabilisation in septic shock [1]. Aim of this study was to examine whether the adsorption-therapy could influence patient-outcome with catecholamine resistant septic shock (CRSS) and acute renal failure(ARV). Furhtermore we tried to identify clinical constellations that would predict an effective use of adsorbers.

**Methods:** We evaluated 44 adult patients with CRSS, ARV and CytoSorb-Therapy during the period 11/14-3/18. Data were collected according to the Cytosorb-Registry and with permission of the local chamber of physicians [AS 88(bB)/2015]. Furthermore we collected data from a matched patient group from the year 2012 (N=14) with septic shock and increasing noradrenaline dependency and ARV. The efficacy was assesed by means of laboratory tests, catecholamine dependency and outcome. Calculations were done with non-parametric-tests (depicted as median values [Q25, Q75]).

**Results:** Initial IL-6 was 5000 ng/l [908,5000] and could be reduced to 302 ng/l [99,891]. PCT was non-significantly reduced from 27μg/l [11, 66] to 20μg/l [6,40]. Vasopressor-dependency could be reduced in 19 patients from 60 μg/´ [50,66] to 24 μg/´ [7,34]. Initial IL-6 in patients with catecholamine-reduction through adsorption was non-significantly different to those with no reduction (2376 ng/l [838, 5000] vs. 5000 ng/l [3488, 5000]). Mortality did not differ significantly between the groups (71% vs 79%). Length of intensive care unit stay (LOS) did differ significantly (13 days [4,24] vs 22 days [19,29]).

**Conclusions:** IL-6 can be reduced with adsorption. Patients with catecholamine-reduction did not differ in regard to their initial IL-6. LOS was shorter for patients treated with adsorption. According to our experience adsorption can be taken into consideration when CRSS is beginning.

**Reference**

1. Hinz B, et al. Int J Artif Organs. 2015;38:461-4.

### P050 Determination of the started period of expanded application to the contact precautions among the scheduled surgical patients with prolonged stay in the mixed intensive care unit

#### K Ishii^1^, H Hidaka^1^, Y Koyama^1^, K Abo^1^, M Arai^1^, M Kosaka^1^, S Takenaka^1^, C Yokoo^1^, Y Yajima^1^, Y Kimura^1^, H Ohmura^1^, K Morinobu^1^, I Shimada^1^, Y Kimura^2^, M Sato^3^, H Morimatsu^4^

##### ^1^Fukuyama City Hospital, Department of Anesthesiology and Oncological Pain Medicine, Fukuyama, Japan; ^2^Fukuyama City Hospital, Central Surgery Department Intensive Care Unit, Fukuyama, Japan; ^3^Fukuyama City Hospital, Medical department (medical information engineer), Fukuyama, Japan; ^4^Okayama University Graduate School of Medicine, Dentistry and Pharmaceutical Sciences, Department of Anesthesiology and Resuscitology, Okayama University Graduate School of Medicine, Okayama, Japan

**Introduction:** In our Intensive Care Unit (ICU), we have already started expanded application to the contact precautions. Applied patients are; 1) Emergency admission, 2) Patients who had already had bacteria* that are required to contact precautions, 3) Scheduled surgical patients with prolonged ICU stay, although we have not yet decided the started period of expanded application exactly. *Detected Bacteria(DB);MRSA, CD, MDRP, ESBL, Pseudomonas A, PISP, PRSP, VRSA. The aim of this study was to determine the adequate starting period of expanded application to the contact precautions in the scheduled surgical patients in the mixed ICU.

**Methods:** We performed retrospective observational study on 4221 patients who were admitted to our ICU after planed surgery from May 2013 to Dec. 2017. We detected the patients who acquired BD newly and investigated the relation to the length of ICU stay. The relationship between detection rate and categorized date was also analyzed using logistic regression adjusted for age, gender, APACHE2, and SOFA score. Using Youden´s index and ROC curve, we also calculated cutoff point of the duration of ICU stay related to detection rate. Finally, we made the logistic regression model of each cutoff day(day1 to 7) and compared Odds Ratio(OR) and AUC of each models using stata.

**Results:** Category day 2 or more, especially day 4 or more had significantly higher detection rate of DB compared to day 1 (Table 1). Similar results were observed in OR according to logistic regression. Day 2, 3 or 4 (Youden´s index) and day 3 (ROC curve) were recommended as the cutoff point. According to each cutoff day models of logistic regression, the day 4 model had the highest OR (51.3) and AUC (0.935).

**Conclusions:** Day 3 or day 4 in mixed ICU stay may be the most adequate period to start expanded application to the contact precautions if the scheduled surgical patient´s stay is prolonged.

**Table 1 (abstract P050).** Relationship between duration of ICU stay and detection of bacteria and toxin that required contact precautions

ICU Stay (day)	Number of patients (n total 4221)	Detection number (n total 26)	Detection rate(%) (total 0.62%)	Logistic regression; Odds ratio (95% CI)
Day 1 or lower	3458	2	0.06	Reference
Day 2	279	2	0.71	12.8 (1.76 - 93.3)
Day 3	139	1	0.71	13.3 (1.17 - 151.8)
Day 4	80	2	2.44	49.2 (6.44 - 375.4)
Day 5	87	3	3.33	70.2 (10.64 - 462.6)
Day 6	37	1	2.63	58 (4.77 - 704.9)
Day 7 or higher	115	15	11.54	255.6 (52.0 - 1257.0)

### P051 Prevention of urinary tract infection in neurocritical patients - a reality

#### P Travassos, L Silva, G Costa, S Chaves, W Costa, R Vale, V Veiga, S Rojas

##### Hospital Bp - A Beneficência Portuguesa De São Paulo, Neurocritical Care Unit, Sao Paulo, Brazil

**Introduction:** The objective of this study was to evaluate the incidence density of urinary tract infection associated with bladder catheter in neurological intensive care unit and identification of actions that were related to low prevalence.

**Methods:** A retrospective analysis of the hospitalized patients from December 2014 to January 2017 was carried out, considering the patients who used the bladder catheter and the cases of urinary tract infection, correlating with improvement actions implemented in the period.

**Results:** In the analyzed period, 3837 patients were hospitalized in the unit, with mean age of 63.77 years, SAPS 3 of 44.58 points, SMR 0.59 and residence time of 4.79 days, with 50.92% using a catheter bladder of delay. Of these, 27 had a urinary tract infection, which represented 1.37% of the patients. During the analyzed period, urological physiotherapy was monitored, daily check of the urinary tract infection prevention bundle, analysis of all cases of infection with search of barriers breaking through Ishikawa methodology, feedback to the multiprofessional team of indicators related to the presence of invasive device, monthly monitoring of the mean time of bladder catheter with established goals.

**Conclusions:** It is possible to guarantee low prevalence of urinary tract infection, in a complex profile of patients, through a multiprofessional approach, accompanied by structured management of data analysis and monitoring

### P052 Risk factors of surgical site infections after thoracic and lumbar surgery: a 6-year single centre prospective cohort study

#### V Spatenkova^1^, O Bradac^2^, Z Jindrisek^1^, J Hradil^3^, P Suchomel^3^, D Fackova^4^, M Halacova^5^

##### ^1^Regional Hospital, Neurocenter, NICU, Liberec, Czech Republic; ^2^Military University Hospital and First Medical School, Charles University, Department of Neurosurgery, Prague, Czech Republic; ^3^Regional Hospital, Neurocenter, Department of Neurosurgery, Liberec, Czech Republic; ^4^Regional Hospital, Department of Clinical microbiology and immunology, Antibiotic Centre, Liberec, Czech Republic; ^5^Na Homolce Hospital, Department of Clinical Pharmacology, Prague, Czech Republic

**Introduction:** Surgical site infection (SSI) is a risk in every operation wound, as it negatively impacts patient morbidity and mortality, and also increases financial demands, such as prolonged hospital stay, further antibiotics and surgical procedures. The aim of this study was to analyse SSI and its risk factors after thoracic and lumbar surgery.

**Methods:** A six-year monocentric observation prospective cohort study monitored the incidence of SSI, wound complications and further risk factors in 274 consecutive patients after planned thoracic and lumbar surgery for degenerative disease, trauma and tumour. All patients received short antibiotic prophylaxis (before and during long operations). All wound complications and SSI were monitored up to 30 days and 1 year after operations. We searched for risk factors for SSI in multivariate logistic regression analysis.

**Results:** We recorded 22 incidences of SSI (8.03%; superficial 5.84%, deep 1.82%, organ 0.36%). There were no differences between these two groups in age (p=0.906), gender (p=0.545), body mass index (p=0.858), spine diagnoses (p=0.745), number of vertebrae (p=0.815), spine localization (p=0.808), use of metal (p=0.428), American Society of Anesthesiologists, ASA Score (p=0.766), urine catheters (p=0.206), drainage (p=0.498), corticoids (p=0.409), transfusions (p=0.262), ulcer prophylaxis (p=0.409) and diabetes mellitus (p=0.811), but SSI had longer hospital stay (p=0.003) and hospital wound complication (p<0.001). Predictor of SSI in multivariate logistic regression analysis was hospital wound complications (OR 20.40, 95% CI 7.32-56.85, (p<0.001) and warm season (OR 2.92, 95% CI 1.03-8.27, p=0.044).

**Conclusions:** Contrary to the prevailing literature, our study did not identify corticoids, diabetes mellitus or transfusions as risk factors for the development of SSI, but only wound complications and warm seasons.

### P053 Measuring the impact of verbal immediate feedback at the end of observational session, on hand hygiene compliance among health care workers (HCWS) in a general intensive care unit (GICU), preliminary results

#### I Livshiz-riven^1^, L Koyfman^2^, A Borer^1^, A Gushanski^1^, S Askira^1^, V Habar^1^, E Ivanov^3^, M Klein^2^, A Danziger^2^, O Azulay^3^, R Nativ^1^, E Brotfain^4^

##### ^1^Ben Gurion University of the Negev, Infection Control Unit, Soroka Medical Center. Faculty of Health Science, Ben-Gurion University of the Negev, Beer Sheva, Israel, Beer Sheva, Israel; ^2^Ben Gurion University of the Negev, Department of Anesthesiology and Critical Care, General Intensive Care Unit, Soroka Medical Center. Faculty of Health Science, Ben-Gurion University of the Negev, Beer Sheva, Israel, Beer Sheva, Israel; ^3^Ben Gurion University of the Negev, Department of Internal Medicine A, Soroka Medical Center. Faculty of Health Science, Ben-Gurion University of the Negev, Beer Sheva, Israel, Beer Sheva, Israel; ^4^Ben Gurion University of the Negev, Department of Anesthesiology and Critical Care, Beer Sheva, Israel

**Introduction:** Health care associated infections (HCAI) are a major problem for patient safety in intensive care units (ICUs). There are different education measures (written material with reminders, continuous feedback, interventions involving novel equipment) on performance of hand hygiene. In the present study, we assessed the impact of immediate verbal feedback on performance of hand hygiene by health care workers using a new Continuous Closed-circuit Television Monitoring (CCTV) method and direct, overt analog observation method.

**Methods:** This is an interventional study. We conducted overt – direct observations and covert - CCTV observational sessions to measure hand hygiene compliance before and after interventional measures of health care workers (HCWs) in our ICU. As interventional measures, we used personal verbal immediate feedback at the end of the overt observational session, performed by infection control nurse.

**Results:** Overall, 2500 opportunities to perform hand hygiene. The compliance rate at the beginning four months of the study, was 190/319 (59.6%), then, it increased to 293/309 (77%) when measured during sessions with feedback, and 112/167 (66%) when measured by overt observations without immediate feedback. The measurements dropped again to 487/990 (49.2%) in overt observations without immediate feedback, while the compliance rate using the feedback sessions continued being the highest measurement (250/403, 62%). Covert, CCTV sessions increased only from 130/533 (24.3%) (before intervention) to 35/120 (27.1%) after intervention measures.

**Conclusions:** We consider that our findings reflecting the inefficacy of the “verbal feedback on performance” method. We believe that it needs additional scrutiny and combining additional intervention strategies to improve hand hygiene compliance.

### P054 Cytomegalovirus infection in immunocompetent intensive care unit patients

#### W Sellami, I Ben mrad, Z Hajjej, M Chniti, I Labbene, M Ferjani

##### Department of Critical Care Medicine and Anesthesiology, Military Hospital, Tunis, Tunisia

**Introduction:** Cytomegalovirus (CMV) has been recognized as an important pathogen in immunocompromised individuals for as long time. In recent years, some studies have focused on CMV infection among immunocompetent intensive care patients. The results are inconsistent and the impact of this virus on the prognosis of these patients is not solved. Our purpose were to determine the prevalence, the risk factors and the consequence of CMV infection in immunocompetent intensive care unit patients.

**Methods:** Observational retrospective case-control study comparing two groups of intensive care patients: CMV-positive and CMV-negative. Patients suspected of developing CMV infection were included. Clinical, demographic and biological characteristics and patient´s care were pointed out to identify risk factors. CMV impact on prognosis was judged by the complications developed and mortality. Another comparison among infected patients between the deceased and the living was carried out in order to determine CMV morbidity and mortality factors.

**Results:** CMV prevalence was 21% in immunocompetent patients suspected of having CMV infection. No significant differences in age, sex, comorbidities, severity, ventilation, use of amines and corticosteroids were found. Transfusion history (p=0.003) ans sepsis (p=0.013) were identified as risk factors in the univariate analysis. In the multivariate analysis, only transfusion history was a risk factor (p=0.004). CMV was not associated with significant morbidity and mortality. Severity score (IGS II) (p=0.02), use of corticosteroids (p=0.002), mechanical ventilation p=0.023), and bacteremia (p=0.001) were associated with mortality in the comparison between the deceased and the living.

**Conclusions:** CMV infection is common in immunocompetent intensive care patients. Transfusion history is a risk factor of infection. CMV is a marker of the severity of the underlying disease of patients rather than a cause of morbidity and mortality

### P055 Necrotising soft tissue infections: the first 24h on ICU. Impact of inflammation and organ dysfunction on mortality

#### A Ogica, C Burdelski, S Kluge, G De Heer

##### Uniklinikum Eppendorf, Klinik für Intensivmedizin, Hamburg, Germany

**Introduction:** Necrotizing soft tissue infections (NSTI) are characterised by extensive tissue necrosis, triggering an overwhelming inflammatory response like sepsis or septic shock [1]. The mortality rate is high and the search for predicting factors has brought conflicting results. We hypothesized that inflammation parameters and organ dysfunctions in the first 24h may correlate with mortality on the intensive care unit (ICU).

**Methods:** We analysed retrospectively electronic data from patients who were admitted to our University Hospital during 2009-2017. For the statistical analysis we used SPSS, version 25.0.

**Results:** 59 patients with NSTI were admitted during the study period. There were 41 males (69.5%) and the median age was 53 years. 15 patients (25.4%) died while on ICU, all of them presented a septic shock at admission. In the univariate analysis just the SOFA Score correlated significantly to mortality (p=0.007). Plotting a receiver operator characteristic curve for the SOFA score against mortality, we obtained an area under the curve of 0.755. A SOFA score ≥7 showed 86% sensitivity and 64% specificity in predicting ICU mortality. Both kidney and liver dysfunction were significantly linked to a higher risk of mortality. An association of four or more organ dysfunctions increased the risk of death by a factor of 8.7.

**Conclusions:** SOFA score and presence of liver or kidney dysfunction respectively in the first 24h correlated well with an increased risk of death. The different inflammatory markers showed no predicting value towards the risk of mortality.

**Reference**

1. Jabbour G et al. World J Emerg Surg 2016; 11: 40.

### P056 Ventilator-associated pneumonia and PaO2/FiO2 value accuracy: a new paradigm?

#### C Dominedò^1^, T Sequeira^2^, C Cillòniz^3^, I Martin-Loeches^4^, M Ferrer^3^, A Torres^3^

##### ^1^Policlinico Agostino Gemelli - Università Cattolica del Sacro Cuore, Department of Intensive Care and Anestesiology, Rome, Italy; ^2^Hospital Prof. Doutor Fernando Fonseca, EPE, Amadora, Pulmonology Department, Lisbon, Portugal; ^3^Hospital Clinic of Barcelona; August Pi i Sunyer Biomedical Research Institute - IDIBAPS, University of Barcelona; Biomedical Research Networking Centres in Respiratory Diseases (Ciberes), Department of Pneumology, Barcelona, Spain; ^4^St. James’s Hospital, Multidisciplinary Intensive Care Research Organization (MICRO), Dublin, Ireland

**Introduction:** Ventilator-associated pneumonia (VAP) is one of the leading infection in critically ill patients. Lacking definitive diagnostic criteria, worsening gas exchange assessed by PaO2/FiO2 ≤240 mmHg has been proposed as a diagnostic criterion. The aim of the study was to assess the adequacy of PaO2/FIO2 ≤240 mmHg to diagnose VAP.

**Methods:** We prospectively included 255 adult patients admitted between 2007 and 2017 to the ICUs of the Hospital Clinic of Barcelona with a clinical diagnosis of VAP. Patients were divided according to PaO2/FIO2 ≤240 mmHg (group 1) or >240 mmHg (group 2) at pneumonia onset. The study was approved by the Ethics Committee of our institution. Patients next of kin provided written informed consent.

**Results:** PaO2/FiO2 was lower than 240 mmHg in 171 (67%) patients. Compared to patients in group 2, patients in group 1 were less severely ill at admission but presented a higher SOFA and CPIS score and a greater incidence of ARDS and shock at pneumonia onset (Fig 1). 117 (69%) patients in group 1 had a microbiological diagnosis of pneumonia, compared to 71 patients (85%) in group 2 (p=0.007). PaO2/FIO2 ≤240 mmHg was associated with less probability of having microbiological diagnosis of pneumonia (OR 0.40, 95% CI 0.21 to 0.79, p=0.008). When adjusted for other variables significantly associated with positive microbiology, PaO2/FIO2 ≤240 mmHg remained significantly associated with less probability of a microbiological diagnosis (adjusted OR 0.34, 95% CI 0.12 to 0.94, p=0.038). Hospital mortality was significantly higher in patients in group 1 compared to group 2 (42% vs 29%, p=0.044). However, no difference was found in non-response to treatment, ICU and hospital stay, ICU mortality (Table 1) and 90-days survival (Fig 2).

**Conclusions:** A significant higher number of patients with VAP didn’t have a definitive etiological diagnosis when using the proposed threshold criteria of PaO2/FIO2 ≤240 mmHg. PaO2/FiO2 ratio does not seem a good predictor of etiology in patients with VAP.

**Table 1 (abstract P056).** Outcome variables

	PaO2/FIO2 ≤ 240 N= 171	PaO2/FIO2 > 240 N= 84	P Value
ICU stay, days	25 ± 21	23 ± 17	0.46
Hospital stay, days	43 ± 36	45 ± 32	0.69
Non-response to treatment, n (%)	94 (55)	48 (57)	0.74
ICU mortality, n (%)	53 (31)	18 (24)	0.11
Hospital mortality, n (%)	71 (42)	24 (29)	0.044

**Fig. 1 (abstract P056).**
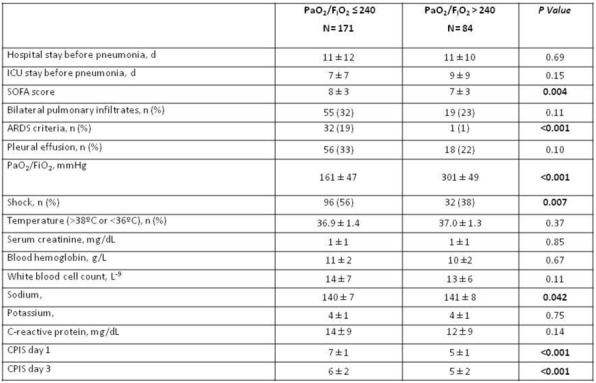
See text for description

**Fig. 2 (abstract P056).**
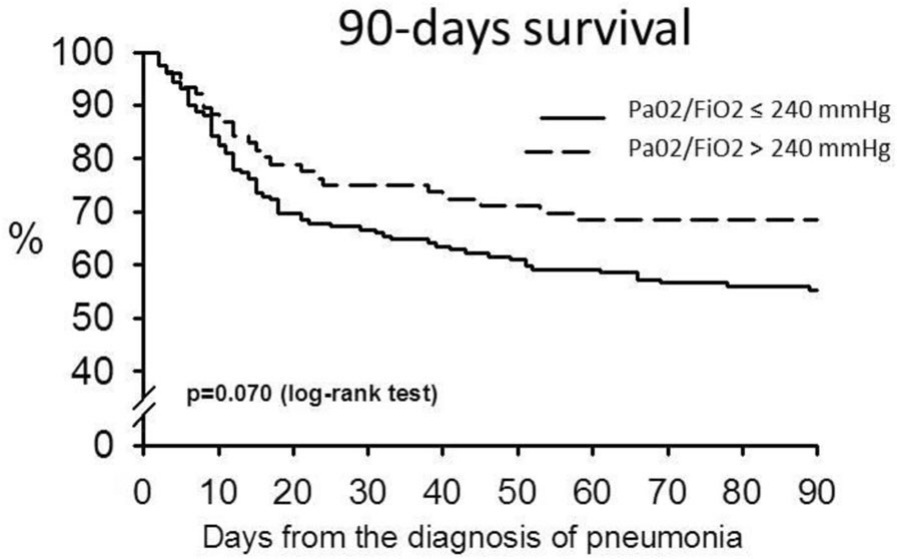
90-days survival

### P057 The relationship between ex-vivo stimulated TNF-alpha levels and the development of nosocomial infections in critically ill mechanically ventilated patients

#### G Levin, J Boyd, D Maslove, S Sibley, P Norman, A Day, J Muscedere

##### Queen´s University, Department of Critical Care Medicine, Kingston, Canada

**Introduction:** Immunological dysfunction is common in critically ill patients but the optimal method to measure it and its clinical significance are unknown. Levels of tumor necrosis factor alpha (TNF-α) after ex-vivo whole blood stimulation with lipopolysaccharide has been proposed as a possible method to quantitate immunological function. We hypothesized that patients with a lower post-stimulation TNF-α level would have increased rates of nosocomial infections (NIs) and worse clinical outcomes.

**Methods:** A secondary analysis of a phase 2 randomized, multi-centre, double-blinded placebo controlled trial [1]. There were no differences in allocation groups; all the patients were analyzed as one cohort. On enrolment, whole blood was incubated with LPS ex-vivo and TNF-α level was measured. Patients were grouped in tertiles according to delta and peak TNF-α level. The primary outcome was the development of NIs; secondary outcomes included 90-day mortality.

**Results:** Data was available for 201 patients. Baseline characteristics and outcomes are reported in Tables 1 and 2. Patients in the highest tertile for post LPS stimulation delta TNF-α compared to the lowest tertile were younger, had a lower acuity of illness and had lower baseline TNF-α. When grouped according to peak post-stimulation TNF-α levels, patients in the highest tertile had higher serum TNF-α at baseline. Both comparisons showed no difference between NIs and clinical outcomes between tertiles. In multi-variate analysis peak or delta TNF-α were not associated with the occurrence of NIs.

**Conclusions:** Admission ex-vivo stimulated TNF-a level is not associated with the occurrence of NIs or clinical outcomes. Further study is required to evaluate the ability of this assay to quantify immune function over the course of critical illness.

**Reference**

1. Muscedere J et al. Crit Care Med 46:1450, 2018

**Table 1 (abstract P057).** Characteristics and outcomes of patients based on admission peak TNF-α levels post LPS stimulation (Age, APACHE II, baseline TNF-α, peak TNF-α post LPS stimulation: mean ± SD; sex, patients with an NI, 90-day mortality: n (%); TNF- a measured in pg/mL)

	Low peak TNF-α (n = 67)	Med peak TNF-α (n= 67)	High peak TNF-α (n =67)
Age	65.9±15.4	64.8±13.9	61.4±15.8
Sex: Female	31 (46.3)	33 (49.3)	40 (59.7)
APACHE II	24.2±7.0	25.6±7.8	25.5±9.5
Baseline TNF-α	6.5±9.0	16.4±27.7	21.3±66.7
Peak TNF-α	0.6±0.5	9.3±8.0	255.4±299.4
Patients with an NI	15 (22)	18 (27)	20 (30)
90 Day Mortality	24 (35.8)	24 (35.8)	28 (41.8)

**Table 2 (abstract P057).** Characteristics and outcomes of patients based on admission change in TNF-α levels post LPS stimulation (Age, APACHE II, baseline TNF-α, peak TNF-α post LPS stimulation: mean ± SD; sex, patients with an NI, 90-day mortality: n (%); TNF-α measured in pg/mL)

	Low delta TNF-α (n = 66)	Med delta TNF-α (n= 67)	High delta TNF-α (n =67)
Age	68.6±12.8	62.6±15.7	61.1±15.7
Sex: Female	36 (54.5)	32 (47.8)	29 (43.3)
APACHE II	26.7±6.1	23.8±7.7	25.0±9.7
Baseline TNF-α	31.0±68.5	3.7±3.4	9.9±19.0
Peak TNF-α	6.4±18.0	6.0±7.8	253.0±301.0
Patients with an NI	17 (26)	15 (22)	20 (30)
90 Day Mortality	26 (39.4)	24 (35.8)	26 (38.8)

### P058 Focused study on ventilator associated condition (VAC) with infection-related ventilator associated complication (IVAC) in a mixed adult unit

#### D Terzi, K Qamar, N Parekh

##### Queen Elizabeth Hospital, Department of Critical Care, Birmingham, United Kingdom

**Introduction:** We believe traditional ventilator associated pneumonia (VAP) is limited by its complexity, subjectivity and marginal attributable mortality. It generates debate but not a matrix. The new paradigm VAC, designed by CDC in 2013 broadens the focus of surveillance, is simple, objective & automatable [1].

**Methods:** Inclusion Criteria: All patients intubated for at least 48 hours. Exclusion Criteria: All elective post-cardiac surgery. Data Capture: Specifically designed excel spreadsheet allowing classification of events into VAC, IVAC & use of antimicrobials. Follow Up: Extubation or death.

**Results:** A total of 133 patients were enrolled between 3rd September to 20th October in 2018. Sixteen patients were confirmed with IVAC (12%) (Fig 1). Our past focused studies based on same tool showed IVAC rate of 17% in 2014 & 14% in 2015 (Table 1). The major reason for this reduction is decrease in percentage of ventilated patients (45 vs 40) as well as slight reduction in length of stay on ventilator (3 vs 2.6 days) (Fig 2).

**Conclusions:** We believe nominal reduction in IVAC rate is due to decrease in % of ventilated patients since 2011 & a slight reduction in ventilator days. We continue to explore possibility of automation of VAC & IVAC data [2].

**References**

1. Klompas M, NEJM 368:1472-5, 2013

2. VAE calculator https://www.cdc.gov/nhsn/vae-calculator/index.html

**Table 1 (abstract P058).** Trends (%) in VAC, IVAC and associated microbial usage

	2014	2015	2015	2018
VAC only, On antibiotics	13	0	6	0.75
No VAC, No antibiotics	35	6	33	5.2
IVAC Possible Pneumonia On Antibiotics	17	6	17	12
No VAC, On Antibiotics	35	88	44	43.6

**Fig. 1 (abstract P058).**
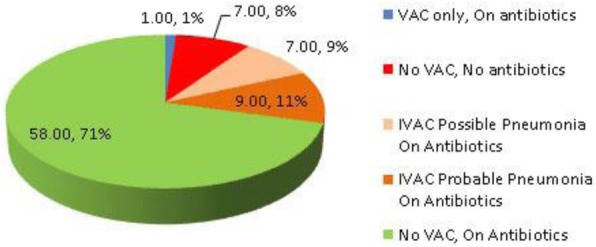
Data analysis of 133 patients in current study

**Fig. 2 (abstract P058).**
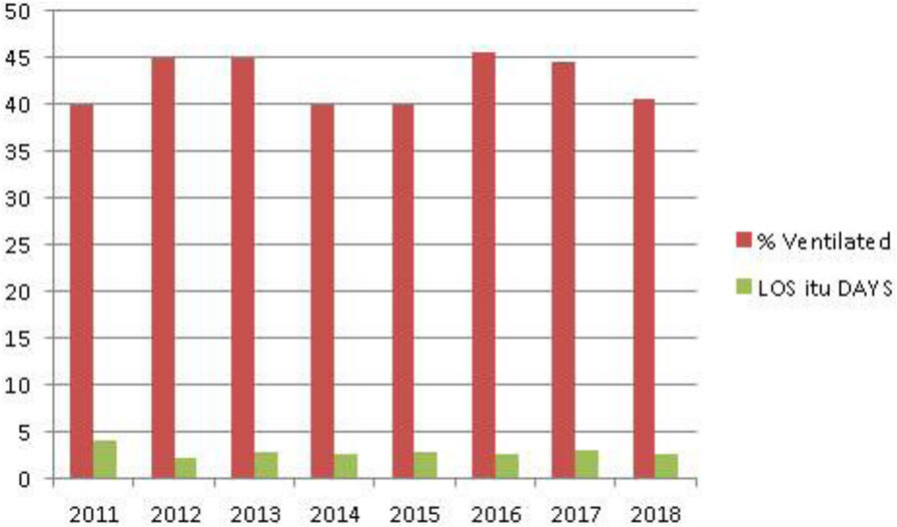
Percentage ventilated and LOS (days) on ventilator

### P059 Risk factors for sepsis in very old patients with community-acquired pneumonia

#### C Dominedò^1^, C Cillóniz^2^, A Ielpo^3^, M Ferrer^2^, A Gabarrús^2^, D Battaglini^4^, J Bermejo-Martin^5^, A Meli^6^, C García-Vidal^7^, A Liapikou^8^, A Torres^2^

##### ^1^Policlinico Agostino Gemelli - Università Cattolica del Sacro Cuore, Department of Intensive Care and Anestesiology, Rome, Italy; ^2^Hospital Clinic of Barcelona; August Pi i Sunyer Biomedical Research Institute - IDIBAPS, University of Barcelona; Biomedical Research Networking Centres in Respiratory Diseases (Ciberes), Department of Pneumology, Barcelona, Spain; ^3^University of Parma, Departments of Medicine and Surgery, Respiratory Disease and Lung Function Unit, Parma, Italy; ^4^Policlinico San Martino, University of Genova, Department of Surgical Sciences and Integrated Diagnostic, Genova, Italy; ^5^Hospital Clínico Universitario de Valladolid/IECSCYL, Group for Biomedical Research in Sepsis (Bio Sepsis), Valladolid, Spain; ^6^University of Milan, Department of Anesthesiology and Intensive Care Medicine, Milan, Italy; ^7^Hospital Clinic of Barcelona, Infectious Diseases Department, Barcelona, Spain; ^8^Sotiria Chest Diseases Hospital, Mesogion, Respiratory Department, Athens, Greece

**Introduction:** There is limited information about sepsis in very old patients hospitalized with community-acquired pneumonia (CAP).

**Methods:** We conducted a retrospective study using data that were prospectively collected at the Hospital Clinic of Barcelona. We included all very old patients (>80 years), with no severe immunosuppression, hospitalized with CAP between 2005 and 2017. We aimed to investigate the prevalence, etiology, risk factors and clinical outcomes of this population, comparing patients with and without sepsis (defined according to SEPSIS-3 criteria). The study was approved by the Ethics Committee of our institution (register: 2009/5451). Written informed consent was waived because of the non-interventional study design.

**Results:** Among 4,190 patients hospitalized with CAP, 1,136 (27%) were very old (>80 years). The incidence of sepsis in very old patients was 70% (n = 795). There was no significant difference in the distribution of pathogens in patients with and without sepsis (Figure 1). Male sex (OR 1.86 [95% CI 1.21 to 3.25]) and chronic renal disease (OR 2.01 [95% CI 1.22 to 3.29]) were independent risk factors for sepsis in the multivariable analysis, while prior antibiotic therapy before admission (OR 0.71 [95% CI 0.53 to 0.95]) was independently associated to a lower risk of sepsis (Figure 2). One-year mortality was higher in very old patients with sepsis compared with those without sepsis (Table 1). A propensity-adjusted multivariable analysis showed that risk factors for 30-day mortality in septic patients were chronic renal disease (OR 2.62 [95% CI 1.36 to 5.03]) and neurological disease (OR 2.56 [95% CI 1.47 to 4.46]), while diabetes mellitus (OR 0.43 [95% CI 0.21 to 0.87]) was a protective factor.

**Conclusions:** In very old patients hospitalized with CAP, antibiotic therapy before admission was associated with a decreased risk of sepsis, whereas diabetes mellitus was associated with a decreased risk of 30-day mortality.

**Table 1 (abstract P059).** Clinical outcomes according to sepsis

Variable	No Sepsis (N = 341)	Sepsis (N = 795)	p value
Length of hospital stay, median (IQR), days	7 (6; 11)	8 (6; 13)	0.017
In-hospital mortality, n (%)	29 (9)	99 (13)	0.051
30-day mortality, n (%)	34 (10)	104 (13)	0.14
1-year mortality, n (%)	49 (14)	154 (20)	0.035
ICU admission, n (%)	17 (5)	75 (9)	0.012
ICU mortality, n (%)	1 (6)	7 (9)	>0.99

**Fig. 1 (abstract P059).**
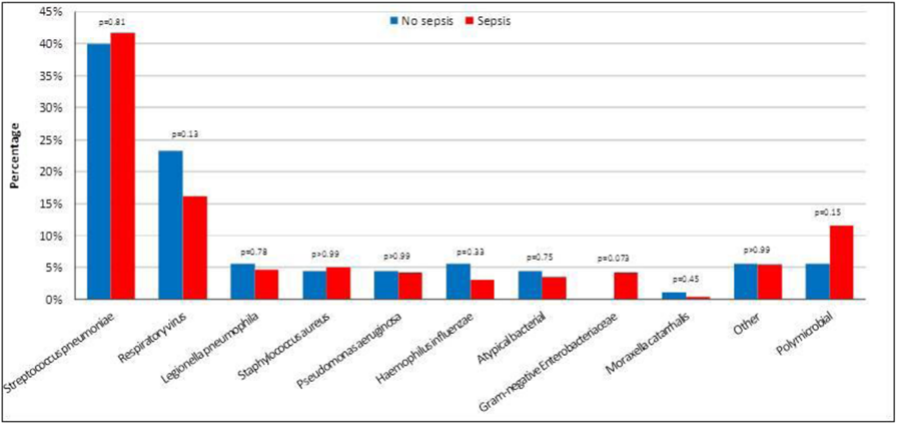
Distribution of microbial aetiology according to sepsis

**Fig. 2 (abstract P059).**
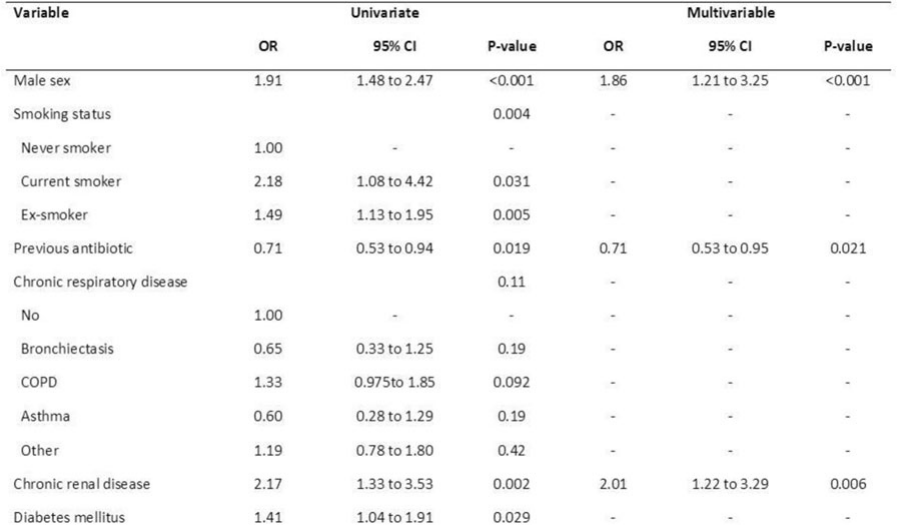
Significant univariate and multivariable logistic regression analyses for sepsis (N = 1,136)

### P060 Can we predict Legionnaire´s disease in patients presenting with community-acquired pneumonia to the emergency department by clinical parameters?

#### R Bolliger, O Neeser

##### Kantonsspital Aarau, Innere Medizin, Aarau, Switzerland

**Introduction:** Legionella species may cause life-threatening pneumonia and thus need early treatment. Differentiating Legionella pneumoniae (LP) from other types of pneumonia including Mycoplasma pneumoniae (MP), Steptococcus pneumoniae (SP) and viral types of community-acquired pneumonia (CAP) has important implications regarding antibiotic therapy. Current testing options for LP infection have limited sensitivity leading to time delays in treatment and to usage of empirical broad-spectrum antibiotics. Recently, a Legionella Scoring system based on six parameters has been proposed. We aimed to independently validate this score and investigate whether additional clinical and laboratory parameters would further improve its accuracy.

**Methods:** We analyzed patients hospitalized in a tertiary care hospital between 2013 and 2017 with CAP and a defined etiology. Association and discrimination were assessed using logistic regression analysis and area under the receiver operator characteristic curve (ROC AUC).

**Results:** We included 713 patients with CAP including 33 (5%) with LP; 56 (8%) with MP; 164 (23%) with SP and 460 (64%) with viruses. All clinical predictors included in the Legionella score were associated with LP CAP. The score overall showed high discrimination between LP and other CAP etiologies with an AUC of 0.83 (95% CI 0.76 to 0.90), which was better than each parameter alone. Results were similar for subgroups based on each of the different CAP types. Additionally, we found that a history of nausea further improves the diagnostic accuracy of the legionella score to an AUC of 0.84 (95% CI 0.77 to 0.91).

**Conclusions:** In patients hospitalized with CAP, a high Legionella score on admission strongly predicts LP infection and thereby can optimize the empiric antibiotic management. A clinical history of nausea further improves diagnosis. Systematic use of this scoring system in conjunction with other diagnostic tests may improve the diagnostic and therapeutic management of patients presenting with CAP.

### P061 Ventilator-associated pneumonia due to multidrug-resistant Acinetobacter baumannii: risk factors and mortality relation with resistance profiles

#### A Ciginskiene^1^, A Dambrauskiene^2^, D Adukauskiene^3^, V Pilvinis^3^, I Zubaviciute^3^

##### ^1^Hospital of Lithuanian University of Health Sciences Kauno Klinikos, Department of Intensive Care, Kaunas, Lithuania; ^2^Hospital of Lithuanian University of Health Sciences Kauno Klinikos, Infection Control Service, Kaunas, Lithuania; ^3^Hospital of Lithuanian University of Health Sciences Kauno Klinikos, Department of Intensive Care, Kaunas, Lithuania

**Introduction:** Acinetobacter baumannii (AcB) remains one of the most prevalent ventilator-associate pneumonia (VAP) causing pathogen. In recent years, share of drug resistant AcB strains across Europe was found to be steadily increasing. Consequently, in 2017, AcB was included in the WHO global priority list of drug-resistant bacteria to highlight the need for the research development. The aim of this study was to identify the relation of risk factors for ventilator-associated pneumonia (VAP) and mortality with drug resistance profiles of AcB.

**Methods:** A retrospective cohort study of patients treated in medical-surgical ICUs with drug-resistant strains of AcB as pathogens of VAPover a 2-year period was carried out.

**Results:** The data of 60 medical-surgical ICUs patients with VAP due to drug-resistant AcB were analysed. The proportions of multidrug-resistant (MDR), extensively drug-resistant (XDR), and potentially pandrug-resistant (pPDR) AcB were 13.3%, 68.3%, and 18.3%, respectively (p<0.05). The SAPS II scores on ICU admission were 42.6, 48.7, and 49 (p<0.048); hospital length of stay prior to ICU was 0, 1, and 2 days (p<0.036), prior to mechanical ventilation - 0, 0, and 3 days (p<0.013), and carbapenem use prior to VAP - 50%, 29.3%, and 18.2% (p <0.036) in MDR, XDR, pPDR AcB VAP groups respectively. The overall in-hospital mortality rate was 63.3%. In MDR, XDR, and pPDR AcB VAP groups it was 62.5%, 61.3%, and 72.7%, respectively (p = 0.772).

**Conclusions:** The VAP risk factors: higher SAPS II score, increased hospital length of stay prior to ICU and mechanical ventilation were related to higher resistance profile of AcB. Carbapenem use was found to be associated with the risk of MDR AcB VAP. Mortality due to drug-resistant AcB VAP was high, but not associated with AcB drug resistance profile. Thus, timely mechanical ventilation and ICU treatment may reduce the risk of VAP due to higher drug-resistant AcB, especially in more severely ill patients.

### P062 Nosocomial wound infections in intensive care unit review of microbiology over 5 years period (2013-2017)

#### A Al Bshabshe^1^, M Joseph^2^, A Assiri^3^, I Asiri ^4^, M Hamid^1^, Y Alqarni ^4^, K Sinnah^4^

##### ^1^King Khalid University, Medicine/Critical Care, Abha, Saudi Arabia; ^2^King Khalid University, Department of Microbiology, Abha, Saudi Arabia; ^3^KKUMC, Critical Care, Abha, Saudi Arabia; ^4^Critical care department, ACH, Abha, Saudi Arabia

**Introduction:** sepsis following traumatic and surgical intervention increases morbidity, mortality, cost and length of patient stay in hospital. The aim of this study was to identify the major pathogens associated with wounds infection and to review their antimicrobial reactions.

**Methods:** A 5-year review of nosocomial wound infection and colonization in patients admitted to the intensive care unit of tertiary care Hospital southern region of Saudi Arabia from Jan. 2013 to Aug. 2017. Patients of all ages and gender who required ICU attention at some point and defined as nosocomial infection using standard CDC criteria and presented with various degrees of wound and bed sore infections were included in the study. Data on bacterial isolates (n= 536) and reactions to antimicrobials (n= 51) were analyzed .

**Results:** There were 379 episodes of wound and 157episodes of bedsore infections. The most common organisms Klebsiella pneumoniae (22.8%) followed by Proteus mirabilis (15.1%); Acinetobacter baumannii (12.7%); Escherichia coli (10.8%); Pseudomonas aeruginosa (10.1%); Morganella morgani (7.6%); Providencia stuartii (3.4%); Staphylococcus aureus (3.0%); Enterobacter aerogenes (1.5%) and Methicillin-resistant Staphylococcus aureus (MRSA) (1.3%) (Fig 1). The percentage sensitivity of the 536 organisms to the 51 antimicrobial agents was 39.2%; intermediate sensitivity was 3.3% and resistant was 57.5% (Fig 2).

**Conclusions:** Data from this and other studies supports the hypothesis that high incidence of gram negative bacilli (91.4%) in particular Klebsiella pneumoniae, Proteus mirabilis and Acinetobacter baumannii are more common in tropical regions compared to gram positive bacteria (8.6%) mainly Staphylococcus aureus. This requires strong infection control actions to enhance patient care.

**Fig. 1 (abstract P062).**
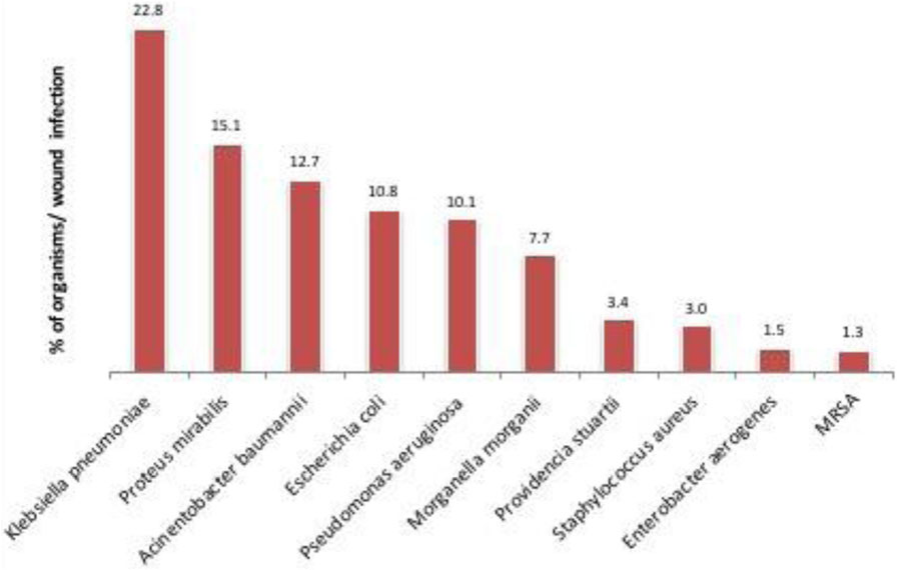
Major organisms (%) causing wound infections in ICU (2013-2017)

**Fig. 2 (abstract P062).**
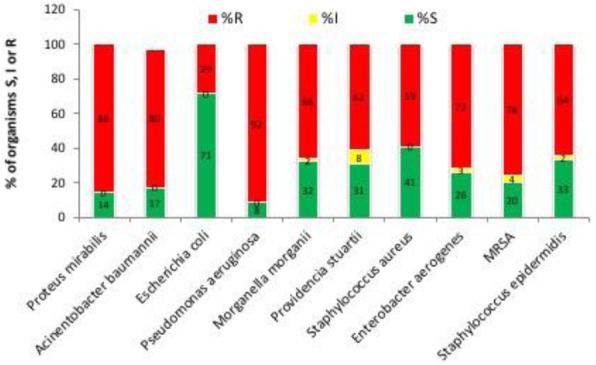
sensitivity pattern of the major organism causing wound infection in ICU

### P063 Predicting performance of risk factors for MDR pneumonia according to the 2017 international ERS/ESICM/ESCMID/ALAT guidelines

#### C Dominedò^1^, A Ceccato^2^, M Ferrer^2^, A Gabarrús^2^, A Torres^2^

##### ^1^Policlinico Agostino Gemelli - Università Cattolica del Sacro Cuore, Department of Intensive Care and Anestesiology, Rome, Italy; ^2^Hospital Clinic of Barcelona; August Pi i Sunyer Biomedical Research Institute - IDIBAPS, University of Barcelona; Biomedical Research Networking Centres in Respiratory Diseases (Ciberes), Department of Pneumology, Barcelona, Spain

**Introduction:** After the 2017 International ERS/ESICM/ESCMID/ALAT guidelines publication [1], no study has evaluated the identified risk factors for multi drug resistant pathogens (MDRP). We aimed to assess the predictive performance of these risk factors.

**Methods:** We analyzed a cohort of patients prospectively collected between 2004 and 2017 in six ICUs of the Hospital Clinic of Barcelona, Spain. Adult patients with no severe immunosuppression and a diagnosis of hospital-acquired pneumonia and ventilator-associated pneumonia with confirmed microbiology were enrolled. The study was approved by the Ethics Committee of our institution. Patients or their next of kin provided written informed consent.

**Results:** Among the 314 patients included in the study, 312 were at high MDR risk: 158 (51%) had no septic shock (group 1) whereas 154 (49%) had septic shock (group 2) (Table 1). Despite no difference in the microbiology, at pneumonia diagnosis, compared to group 1, patients in group 2 presented a higher SOFA [5 (4;7) and 9 (8;12), p<0.001] and ARDS [12 (8) and 26 (17), p 0.014]. Admission to hospital settings with high rate of MDRP and prior antibiotic use showed the highest prevalence in the overall population (81% and 79% respectively), with a high sensitivity (91% and 84% respectively) and negative predictive value (NPV) (83% and 75% respectively). Previous isolation of MDRP was the only risk factor presenting a specificity and a positive predictive value (PPV) of 100% and a high accuracy [AUC 0.80 (0.68 to 0.93)] (Fig 1). Despite the combination hospital settings with high rates of MDRP/previous antibiotic use had the highest prevalence (64%) in the overall population, only the combinations including previous isolation of MDRP showed a specificity and a PPV of 100%, with an AUC > 0.6 (Fig 2).

**Conclusions:** The guidelines categorized a higher proportion of patients with high risk of MDRP. Previous isolation of MDRP showed the best performance for MDR prediction.

**Reference**

Torres A et al. Eur Respir J. 50(3); 2017

**Table 1 (abstract P063).** Patients baseline characteristics at ICU admission

	High MDR risk; no septic shock (n=158)	High MDR risk; septic shock (n=154)	p value
Age (years), median (p25; p75)	67 (54; 76)	65 (53; 74)	0.981
Male sex, n (%)	117 (74)	107 (70)	0.370
Chronic heart diseases, n (%)	59 (37)	36 (23)	0.007
COPD, n (%)	47 (30)	27 (18)	0.012
Chronic liver diseases, n (%)	19 (12)	38 (25)	0.004
SOFA score, median (p25; p75)	6 (4; 8)	8 (6; 10)	<0.001

**Fig. 1 (abstract P063).**
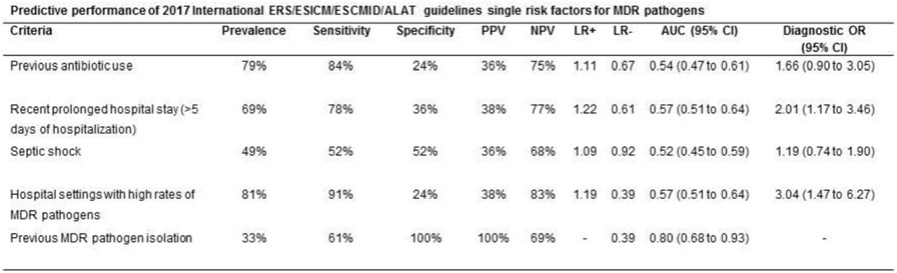
Predictive performance of 2017 International ERS/ESICM/ESCMID/ALAT guidelines single risk factors for MDR pathogens

**Fig. 2 (abstract P063).**
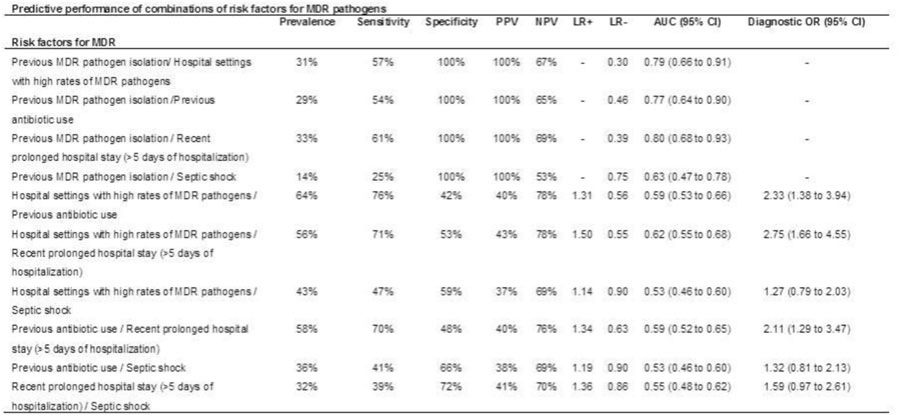
Predictive performance of combinations of risk factors for MDR pathogens

### P064 Monobacteremia of hospital-acquired gram-negative rods in Lithuanian university’s hospital intensive care unit

#### D Adukauskiene^1^, D Valanciene^2^, A Dambrauskiene^3^, A Adukauskaite^4^, L Jazokaite^5^, V Micpovilyte^5^, A Vitkauskiene^6^

##### ^1^Hospital of Lithuanian University of Health Sciences Kaunas Clinics, Intensive Care Unit, Kaunas, Lithuania; ^2^Klaipeda Seamen´s Hospital, Anaesthesiology and intensive care department, Klaipeda, Lithuania; ^3^Hospital of Lithuanian University of Health Sciences Kaunas Clinics, Infection Control Service, Kaunas, Lithuania; ^4^Medical University of Innsbruck, Cardiology, Innsbruck, Austria; ^5^Hospital of Lithuanian University of Health Sciences Kaunas Clinics, Intensive Care Clinic, Kaunas, Lithuania; ^6^Hospital of Lithuanian University of Health Sciences Kaunas Clinics, Laboratory Medicine Clinic, Kaunas, Lithuania

**Introduction:** Growing antimicrobial resistance among Gram-negative rod (GNR) strains is a worldwide issue. Flora monitoring is associated with right first choice of antimicrobial treatment. The aim of study was to analyze sensitivity of hospital-acquired (HA) GNR monobacteremia strains to antimicrobial drugs and risk factors for mortality in Intensive Care Unit (ICU).

**Methods:** Ongoing retrospective cohort study of patients treated in ICUs of Kaunas Clinic’s with positive blood culture for GNR taken after 72 hrs of hospitalisation during 7yrs period was carried out.

**Results:** We’ve found 196 cases of HA bacteremia due to GNR: 72 (36.7%) caused by Acinetobacter spp. (P=.03), 38 (19.4%) by Escherichia coli, 34 (17.3%) by Klebsiella spp., 19 (9.69%) by Serratia spp. and just few others. Sensitivity to carbapenems (n=182, 92.9%), amikacin (n=131, 66.8%) cefoperazon/sulbactam (n=116, 59.2%), and piperacillin/tazobactam (n=93, 47.4%) was found. There were found 151 (77%) multi-drug-resistant (MDR) and among them 64 (42.4%) – extra-drug-resistant strains. Among Acinetobacter spp. there were found 61 (84.7%) MDR strain (P=.04). In general it was related with male gender (n=90/118, P=.04, OR=4.56, CI95%=2.4-15.4), elderly (P=.001, RR=13.3), acute kidney injury (AKI) (n=98/128, P=.03, OR=0.84, CI95%=0.35-0.92). In multi-variate analysis lethal outcome 73.5% (n=144) was associated with male gender (P=.04, OR=2.92, CI95%=1.98-8.67), mechanical ventilation (P=.04, OR=4.125, CI95%=2.70-6.34), septic shock (P=.001, OR=8.21, CI95%=1.33-6.26), AKI (P=.02, OR=5.67, CI95%=1.18-27.25), MDR strain (P=.01, OR=3.47, CI95%=2.46-7.56).

**Conclusions:** 7yrs study revealed Acinetobacter spp. as predominant, mostly MDR pathogen of HA GNR bacteremia in ICUs. HA MDR GNR bacteremia was related with male gender, elderly and AKI. High sensitivity of GNR to carbapenems was found. High rate of mortality 74% was associated with male gender, mechanical ventilation, septic shock, AKI and MDR strain.

### P065 Risk factors and prognosis of critically ill patients colonized with KPC-producing enterobactereacea in Brazil

#### E Bogossian^1^, D Salgado^1^, S Nouér^2^

##### ^1^Universidade Federal do Rio de Janeiro, Intensive Care department, Rio de Janeiro, Brazil; ^2^Universidade Federal do Rio de Janeiro, Infectious Disease Department, Rio de Janeiro, Brazil

**Introduction:** Of great concern is the dissemination in health care environments of multi-drug resistant (MDR) bacteria, especially Klebsiella pneumoniae carbapenemase (KPC)-producing enterobactereaceae.

**Methods:** Our objective is to identify the differences in risk factors and outcomes between patients who did and did not acquire KPC- producing bacteria during their stay in the ICU of an university hospital in Rio de Janeiro, Brazil. We designed a nested case – control study of a retrospective cohort from May 2014 to June 2015. Three hundred and thirty nine patients were admitted to the ICU. 36 cases (patients infected or colonized by KPC-producing bacteria and 292 ‘controls’ (all other patients who did not have MDR isolation). The two groups were compared according to demographic clinical and microbiological data.

**Results:** There was no significant differences in gender, age, severity scores between the two groups. Patients with KPC- producing bacteria had longer ICU and hospital stays than control patients and they required more life-support such as mechanical ventilation, vasopressor use and dialysis. In hospital mortality rate was higher in KPC- producing bacteria group (63.9% vs 35.3% p=0.001). A multivariate analysis showed that mechanical ventilation (OR: 2.92; CI 1.02-8.32) and previous use of antibiotics (OR: 13.39; CI 1.62-111.20) were risk factors for acquiring KPC.Factors associated with in-hospital mortality were: male gender, (OR: 3.29; CI 1.54-7.05), Charlson score (OR: 1.26; CI 1.09-1,45) and mechanical ventilation (OR: 16.63; CI 4.79-57.70).

**Conclusions:** Colonization was associated with longer ICU and hospital LOS, more requirements of life-support and higher in-hospital mortality rate. Previous antibiotic use and mechanical ventilation were the most important risk factors for patients becoming colonized. Factors associated with mortality were male gender, Charlson score and mechanical ventilation.

### P066 Infection with ESBL-E in the ICU: analysis of antibiotic therapy

#### S Cuypers, L De Bus, J De Waele, P Depuydt

##### UZ Gent, Intensive Care, Gent, Belgium

**Introduction:** Carbapenems are usually the preferred antibiotics for the treatment of severe infections caused by extended-spectrum Β -lactamase-producing Enterobacteriaceae (ESBL-E), even in the presence of documented susceptibility for other antibiotic classes. Ecological arguments, on the other hand, provide rationale to limit carbapenem use as much as possible.

**Methods:** We retrospectively evaluated carbapenem and non-carbapenem antibiotic treatment for ESBL-E infections in the intensive care unit (ICU) of Ghent University Hospital during a 4-year period (2013-2016). Clinical cure was defined as stop of antibiotics together with resolution of clinical signs of infection.

**Results:** Empirical antibiotics were appropriate in 98 of 132 episodes (74%) and consisted of carbapenem and non-carbapenem monotherapy in 37% and 63%, respectively. Non-carbapenem antibiotics were appropriate in 37%. Appropriate empirical therapy (n=98) consisted of carbapenem and non-carbapenem antibiotics in 50.0% of episodes each. Carbapenems were de-escalated in 10/49 episodes (20%); appropriate non-carbapenem antibiotics were changed in 18/49 episodes (36%); in 1, respectively 3 episodes, this was due to clinical failure or emerging resistance. Clinical cure was observed in 86% and 90% of episodes appropriately treated with carbapenem or non-carbapenem antibiotics, respectively; in this subgroup, a bivariate analysis showed absence of clinical cure to be associated with septic shock (OR 1.8; 1.1-3.2), but not with carbapenem antibiotics (OR 1.3; 0.5-3.6).

**Conclusions:** In our ICU, non-carbapenem empirical therapy in ESBL-E infection carried a high risk for inappropriateness. However, no clear relationship between the use of carbapenem versus non-carbapenem antibiotics and clinical cure was found.

### P067 The favorable outcome of Burkholderia cenocepacia blood stream infection (BSI) in cancer patients receiving chemotherapy is associated with early administration of antibiotic therapy

#### A Pronina, I Kurmukov, O Sekhina, S Kashia, S Menshikova

##### “N.N. Blokhin National Medical Research Center of Oncology” of the Ministry of Health of the Russian Federation, Moscow, Russia

**Introduction:** B. cenocepacia BSI is a rare and dangerous complication in cancer patients (pts) receiving chemotherapy. We described the causes of B. cenocepacia associated bacteremia, characteristics of clinical presentation and outcomes in patients receiving chemotherapy for solid tumors.

**Methods:** 11 adult patients (median age 53 years, 2 males, 9 with permanent vascular access) receiving chemotherapy with B. cenocepacia BSI confirmed by a culture-based study (identification by MALDI-TOF with a confidence index over 2,300).

**Results:** Sanitary and epidemiological examination revealed the connection between infection and intravenous infusion of dexamethasone performed concurrently with chemotherapy. In 5 patients fever with chills and hypertension developed within 2 hours after infusion of the infected drug; empirical intravenous antibiotic therapy started immediately after collecting blood culture. In 6 patients fever appeared after 2-4 days outpatiently, so they received antibiotics per os. All these patients had permanent vascular access, and BSI was detected either the next chemotherapy course when fever reappeared (3 pts) while using vascular access, or as a result of a specific examination (3 pts). In all cases empirical antibiotic therapy started on the first day of fever, drug correction was performed in 6 patients according to results of bacteriological research. Septic shock developed in 1 patient, pneumonia in 3 patients. Permanent vascular access was preserved only in 1 case. All patients were cured and continued to receive antitumor treatment.

**Conclusions:** Detection of more than 1 case of B. cenocepacia BSI should be the reason for sanitary and epidemiological examination. A favorable outcome of BSI treatment is associated with the early start of antibiotic therapy and its correction after microbiological examination.

### P068 Emerging infections due to Shewanella spp.: a case series in Hong Kong

#### WS Ng, HP Shum, WW Yan

##### Pamela Youde Nethersole Eastern Hospital, Department of Intensive Care, Chai Wan, Hong Kong

**Introduction:** Shewanella species are emerging opportunistic pathogens that can cause severe soft-tissue, respiratory, hepatobiliary, gastrointestinal infections, and bacteraemia. Most of the published information for this organism are limited to isolated case reports and small case series. Here we report the largest case series of infections caused by Shewanella species.

**Methods:** Patients admitted to a regional hospital in Hong Kong with Shewanella species infection from 1st Apr, 2010 to 30th Sep, 2018 were included. Demographics, antibiotics, microbiology and outcomes were retrospectively analyzed.

**Results:** In an 8.5-year period, we identified 71 patients with Shewanella species infection and 65% of them were male. Their median age was 76 (IQR 66-84). Among them, 27% had diabetes mellitus, 20% had chronic kidney disease and 31% had an underlying malignancy. Hepatobiliary sepsis (45%) was the most common presentation, while soft tissue and respiratory tract infection accounted for 28 and 17% of infection respectively. Among 93 collected specimens, 48% were S. algae, 9% were S. putrefaciens and 43% could be identified up to species only. The identified organisms were usually sensitive to Ceftazidime (98%), ciprofloxacin (95%), gentamicin (96%), piperacillin (88%) and sulperazon (89%). Seven patients (9.9%) required ICU care and their APACHE IV predicted risk of death was 0.34 (IQR 0.14-0.77). Their median ICU length of stay was 5 days (IQR 4-8) and all of them could be discharged from ICU. The median hospital length of stay for all patients was 6 days (IQR 3-16) and the hospital mortality was 13%.

**Conclusions:** This large case series suggested that Shewanella infections are commonly associated with underlying comorbidities, especially with malignancy. Antibiotic resistant isolates are uncommon.

### P069 Multidrug-resistant bacteria in burns: incidence, and risk factors

#### L Debbiche^1^, A Mokline^1^, H Fraj^1^, M Ben Saad^1^, I Rahmani^1^, K El Feleh^1^, L Thabet^2^, A Messadi^1^

##### ^1^Trauma and Biurn Center, Intensive Burn Care Department, Tunis, Tunisia; ^2^Trauma and Burn Center, Laborabory of Biology, Tunis, Tunisia

**Introduction:** Despite improvements in early treatment, survival following burn injury remains challenged by sepsis and emergence of Multidrug-Resistant Bacteria (MRB). The objective of our study was to assess epidemiological aspects and bacterial resistance patterns of bacteria isolated from intensive burn care unit and to identify risk factors.

**Methods:** A prospective, monocentric study was conducted from May to September 2018 in a burn unit in Tunisia. In all patients, a search for BMR carriage (skin, rectal, bronchial, urinary, and catheter samples) and for carbapenemases (VIM, NDM and OXA 48) by polymerase chain reaction (PCR) were carried out initially at admission then weekly.

**Results:** During the study period, 31 patients were included. The mean age was 35 years. They were 17 men and 14 women. The average burned surface area was 39%. Patients were transferred from another hospital structure in 80% of cases with a delay of 36 hours. Isolated MRB were: Enterobacteriaceae producing extended-spectrum Β -lactamases (ESBLs) (49%), Carbapenemase-producing Enterobacteriaceae (CPE) (87%), Methicillin- Resistant Staphylococcus aureus Strains (19%) and glycopeptide-resistant enterococci (GRE) (13%). Carbapenemase-encoding genes were detected in 22 patients: 12 at admission and 10 at the first week post admission. The genes detected were New Delhi metallo-Β -lactamase (92%), Verona integron-encoded metallo-Β -lactamase (58%), and oxacillinase-48 (58%). Univariate analysis identified risk factors associated with acquiring ESBLs and CPE were: secondary transfert; indwelling device, antibiotic use of aminosids and amox-clavulanic acid and non-compliance with hygiene measures.

**Conclusions:** Detection of Multidrug-Resistant Bacteria in our study was higher: ESBLs in 49% of cases and CPE in 87% .So, detection and isolation of these patients and strengthen infection control measures allows us to improve therapeutic efficacy and improves their prognosis.

### P070 Incidence of developing repeated hospital acquired infection in intensive care unit; an emerging challenge in resource limited settings

#### E Shimber

##### Hawassa University Comprehensive Specialized Hospital, Emergency and Critical Care Medicine, Hawassa, Ethiopia

**Introduction:** Repeated incidence of acquiring infection in Critical care unit of a hospital is one of the burdens for the countries’ health system. Despite various researches have been done regarding hospital acquired infections, developing repeated attack still needs further investigation. This research tries to magnify the threats and suggest a way forward.

**Methods:** A retrospective survey was conducted on all patients admitted to an ICU of Hawassa University Comprehensive Specialized Hospital from July 2018 to October 2018. Repeated ICU-acquired infections were defined as reinfection with different strain of organism or relapse of previous infection with the same pathogen during patient’s stay in ICU after being admitted for more than 48 hours and being treated for initial infection appropriately. Infection diagnosis was made based on Centers for Disease Control criteria. Multivariate analysis was used. Finally, ICU-mortality rate was tasted among patients who developed the repeated ICU-acquired infection.

**Results:** A total of ninety-four patients were admitted to ICU. The rate of developing ICU acquired infection was 22% with repeated rate of infection being 8.5%. Overall mortality rate was 44%. Out of these, patients with ICU acquired repeated infection took only 4% of the proportion. Those of who stayed more than two weeks develop repeated attack at a rate of 87.5%. All Patients were having indwelling device. 50 % of them had chronic co morbid medical disease. Duration of ICU stay and indwelling device use had positive association with a strong Pearson correlation of 1. Drug sensitivity test revealed around 62.5% of them have developed resistance or intermediate sensitivity for initial antibiotics of choice.

**Conclusions:** Despite the low rate of repeated infection, multi-drug resistance pattern is high. The research reveals the need for proper antibiotic stewardship and application of standard infection control measures in ICUs.

### P071 Changes of pathogens and clinical outcomes of ICU-acquired bacteremia as results of 5-year antimicrobial stewardship implementation

#### M Dementienko, V Gusarov, D Kamyshova, M Zamyatin

##### Federal State Public Institution “National Medical and Surgical Center named after N.I.Pirogov” of the Ministry of Healthcare of the Russian Federation, ICU, Moscow, Russia

**Introduction:** Conflicting results have been found regarding impact of the antibiotic stewardship program (ASP) on pathogens resistance and clinical outcomes of intensive care unit (ICU)-acquired bacteremia. The aim of the study was to evaluate the effect of ASP implementation on the incidence of ESKAPE-bacteremia and candidemia in ICU patients, level of antimicrobial resistance among causative microorganisms, as well as the hospital length of stay (LOS) and mortality of ICU patients with bacteremia.

**Methods:** Prospective interventional single-center study in the period Jan 2011 to Dec 2017. Intervention onset: 2013. The intervention: ASP, including multidisciplinary team building, antimicrobial therapy and prophylaxis protocols, infection control measures, education, internal audit. The data from 2012 (pre-intervention) and 2017 (intervention periods) reports were compared. We assessed incidence of ESKAPE-bacteremia and candidemia. The hospital LOS and mortality of ICU’s patients with bacteremia were secondary end-points.

**Results:** 4960 blood samples were analyzed, 917 (18.5%) positive blood cultures from 374 IÑU’s patients were obtained. Changes of the pathogens, causing ICU-acquired bacteremia are presented in the Table 1. The overall proportion of ESKAPE-bacteremia and candidemia decreased from 69 (53.9%) to 41 (29.3%), p <0.001.

**Conclusions:** Implementation of ASP in hospital allows to decrease incidence of ESKAPE-bacteremia and candidemia, which may lead to improved clinical outcomes in ICU’s patients (Fig 1).

**Table 1 (abstract P071).** Changes of the pathogens, causing ICU-acquired bacteremia

Etiological agent, n(%)	2011	2012	2013	2014	2015	2016	2017	p-value
The number of positive blood cultures, n	145	128	125	119	131	129	140	
MRSA	2(1.2)	0	2(1.6)	1(0.8)	2(1.5)	2(1.6)	9(6.4)	0.004
E. faecium VR	7(4.8)	0	0	0	0	0	1(0.7)	0.999
ESBL-producing Enterobacteriacae spp.	26(17.9)	45(35.2)	42(33.6)	32(26.9)	37(28.2)	19(14.7)	23(16.4)	<0.001
K. pneumoniae CPR	5(3.5)	21(16.4)	24(19.2)	11(9.2)	14(10.7)	8(6.2)	7(5.0)	<0.01
A. baumannii + P. aeruginosa MDR	34(23.5)	16(12.5)	4(3.2)	12(10.1)	4(3.1)	13(10.1)	6(4.3)	0.024
Candida spp.	19(13.1)	7(5.5)	5(4.0)	5(4.2)	4(3.1)	2(1.1)	2(1.4)	0.091

**Fig. 1 (abstract P071).**
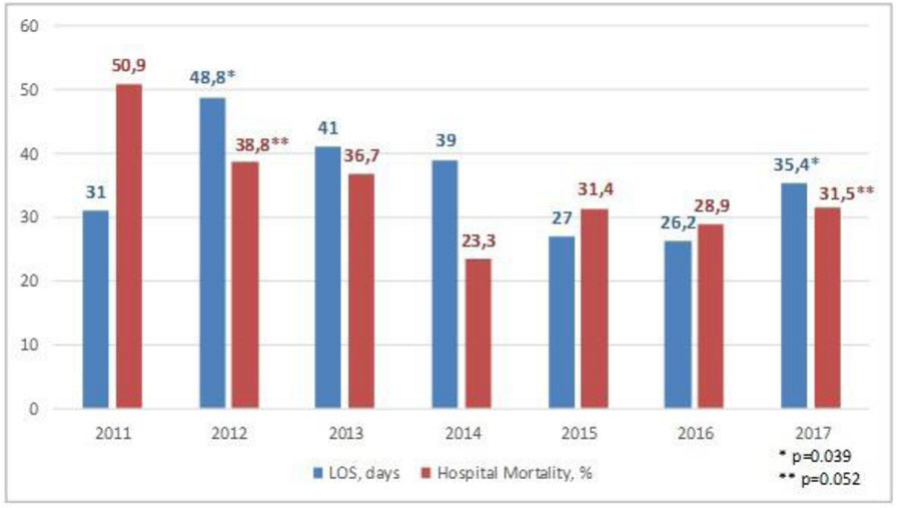
Clinical outcomes of the patients with ICU-acquired bacteremia

### P072 Association of multi-drug resistant (MDR), extended-drug resistant (XDR) and pan-drug resistant (PDR) gram negative bacteria and mortality in an intensive care unit(icu)

#### S Chatterjee^1^, S Sinha^1^, A Bhakta^2^, T Bera^3^, T Chatterjee^3^, S Todi^1^

##### ^1^AMRI Hospitals, Critical Care, Kolkata, India; ^2^AMRI Hospitals, Microbiology, Kolkata, India; ^3^Jadavpore University, Clinical Research, Kolkata, India

**Introduction:** The study assessed mortality associated with non-MDR, MDR, XDR and PDR gram-negative bacterial (GNB) infections in the ICU.

**Methods:** Retrospective cohort study conducted in the ICU of a tertiary-care hospital in India between July2017 to January2018. All consecutive infectious episodes with positive culture for GNB and treated with antibiotic therapy within 24 hours of sample collection were included. Variable information collected included patient demography, APACHE IV score, culture report, drug resistance pattern of organism isolated(non-MDR, MDR, XDR/PDR). Primary outcomes were all cause ICU mortality which was compared between three groups-non-MDR, MDR, and XDR/PDR. All analysis were done after adjusting for disease severity.

**Results:** Of 323 eligible ICU patients 59.4% were males. Mean age significantly differed between non-MDR, MDR and XDR/PDR groups(68.2±13.6, 67.3±16.2 and 62.6±19.1 years respectively, p=0.003). Mean APACHE IV score was similar between groups(67.1±28.2, 69.7±24.9 and 75±27.7 respectively, p=0.07). Commonest isolated GNB was Klebsiella pneumoniae(35%). 39%(n=126) of patients had non-MDR organism, 26%(n=84) were infected with MDR and 35%(n=113) had XDR/PDR organisms in their bio-specimen. Commonest isolated MDR GNB was E.Coli(31%) while commonest XDR/PDR GNB was Klebsiella pneumoniae(55.8%). Overall ICU mortality did not significantly differ between non-MDR and MDR groups(19.4%vs.21.4%; p=0.7); there was significant difference in mortality between MDR and XDR/PDR groups(21.4%vs.41.6%; p =0.003). Risk-adjusted analysis showed higher odds of mortality for MDR infected patients compared to non-MDR group but this was not statistically significant(OR 1.14 95%CI 0.6-2.8; p=0.7). However, compared to MDR, XDR/PDR infected patients were significantly more likely to die in adjusted analysis(OR 2.5 95% CI 1.4-4.9, p=0.004).

**Conclusions:** Prevalence of XDR/PDR GNB and its associated mortality is high in our ICU compared to MDR GNB.

### P073 First outbreak of colistin resistant Klebsiella pneumoniae in an adult intensive unit in South India

#### J Gopaldas^1^, G Raju^1^, B Hosdurg^2^, B Malavalli^2^

##### ^1^Manipal Hospital, Bangalore, India, Critical care medicine, Bangalore, India; ^2^Manipal Hospital, Bangalore, India, Laboratory Medicine, Bangalore, India

**Introduction:** Colistin-resistant Klebsiella pneumoniae (CR-KP) is increasingly reported around the world. It is worrying to note emergence of resistance to last line of defence against MDR gram negative infections in regions endemic to Carbapenem resistance. We report the first outbreak of CR-KP co-producing carbapenemases in an adult Intensive Care Unit (ICU) from South India.

**Methods:** Retrospective analysis of all patients with Carbapenem resistant Klebsiella pneumoniae blood stream infection (BSI) was done between January 2017 and December 2017. Microbiological and clinical variables along with outcomes were analysed.

**Results:** Seven patients had CR-KP with no prior exposure to Colistin. All seven were modified Hodge test (MHT) negative making probability of blaKPC unlikely. In resource limited setting, analysis beyond MHT could only be performed for CR-KP samples. 2/7 samples belonging to CR-KP isolates produced the blaNDM-1 whilst 5/7 CR-KP isolates did not produce either blaKPC or blaNDM carbapenemases prompting hypothesis of blaOXA-48 or blaVIM as the causative factor. Compared to Carbapenem resistance only group, CR-KP group had higher APACHE II, ICU length of stay and mechanical ventilation duration. 28 day mortality was noted to be 56.3% for Carbapenem resistant and 43% for CR-KP groups. Aggressive infection control measures were undertaken with successful containment of CR-KP strains along with reduction in overall BSI.

**Conclusions:** Infection control measures form the backbone of patient care in centres showing endemicity for Carbapenem resistant Klebsiella to prevent Colistin resistance and also to reduce occurrence of overall blood stream infections.

### P074 Rapid diagnosis of carbapenem resistance: experience of a tertiary care cancer center with multiplex PCR

#### S Mukherjee

##### Tata Medical Center, Critical Care Medicine, Kolkata, India

**Introduction:** Sepsis due to carbapenem resistant organisms has high mortality; inappropriate empirical antibiotic is one of the main causes of this poor outcome. On the contrary, “Too much” broad spectrum empiric antibiotics will increase drug resistance, even in community, because of selection pressure. So, early diagnosis of resistance pattern (carbapenemase genes) is crucial. Aim of this study is to compare rapid diagnostic test like polymerase chain reaction (PCR) with conventional culture sensitivity (C/S) to identify carbapenem resistance.

**Methods:** This is a prospective observational study done in Tata Medical Center, Kolkata, India. Real time multiplex PCR technique has been developed “in house” in our microbiology lab and can identify NDM, NDM1, KPC, OXA - 23, OXA - 48, OXA - 58 & VIM carbapenemase genes. Blood cultures were sent as per clinical & laboratory diagnosis of sepsis in ICU patients. Culture positive samples had been used for conventional C/S by VITEK 2 system along with PCR study to identify carbapenemase genes. Result of PCR technique was been compared with conventional C/S method.

**Results:** Multiplex PCR results were available within 3-4 hours of positive blood culture compared to conventional C/S method that takes 2 -3 days. Among 392 positive blood cultures, 146 samples were positive for carbapenemase genes. Most common gene identified was OXA - 48 (43%), followed by NDM1 (25%). Our PCR technique has very high sensitivity, specificity, positive & negative predictive value (99.35%, 94.65%, 93.1% & 99.43% respectively) while comparing with final C/S report by VITEK 2 system (Table 1). There was only one false negative diagnosis for carbapenem resistance.

**Conclusions:** Real time multiplex PCR for carbapenemase gene can be helpful for early diagnosis of carbapenem resistance and can help us to choose / modify antibiotics or to use ‘targeted therapy’. It is more practical to “rule - in” infection rather than “rule - out” by this technique.

**Table 1 (abstract P074).** Comparison of positive Carbapenemase Genes by bottle PCR with detection of carbapenem resistance by VITEK 2 method

Sr. No	"Type of genes detected	“Numbers (total = 146)”	Matched with Vitek-2 (i.e. final carbapenem resist	Carbapenem sensitive by Vitek-2 final report (Resu
1.	KPC	2	2	0
2.	NDM	10	9	1
3.	NDM-1	36	32	4
4.	OXA-23	4	4	0
5.	OXA-48	63	60	3
6.	VIM	2	2	0
7.	Combination of genes	29	27	2

### P075 Carbapenemase producing enterobacteriaceae colonization in an ICU: risk factors and clinical outcomes

#### M Miranda, JP Baptista, J Janeiro, P Martins

##### Centro Hospitalar e Universitário de Coimbra, Intensive Care Unit, Coimbra, Portugal

**Introduction:** Carbapenemase-producing Enterobacteriaceae (CPE) colonization has been increasingly reported in Intensive Care Units (ICUs) since their first identification more than 20 years ago. Colonization with CPE seems to constitute a risk factor for mortality. The aim of our study was to identify associated risk factors and clinical outcomes among patients with fecal colonization by CPE admitted to a Portuguese tertiary hospital ICU.

**Methods:** A 2-year retrospective study was performed in patients with previous unknown CPE status (colonization or infection), admitted to our ICU. Rectal swabs were performed and analyzed using real-time polymerase chain reaction testing. Clinical records were reviewed to obtain demographic and clinical data.

**Results:** Of 903 patients admitted, 38 (4.2%) harbored CPE, 15 (39.5%) were colonized at admission and 23 (60.5%) acquired CPE colonization during ICU stay. The most frequent carbapenemase genes detected were KPC (87.5%) and VIM (12.5%). CPE carriers had high rates of hospitalization (previous or ongoing), invasive procedures (mainly intraabdominal surgery), malignancy (hematopoietic or solid tumor), antibiotic intake as well as slightly higher severity scores (SOFA, SAPS II and APACHE II). Nine patients developed CPE-related infections during ICU stay (4 pneumonia, 3 urinary tract infections, 1 peritonitis and 1 surgical wound infection). CPE colonization was associated with an increased length of stay (26.3 versus 14.3 days in the overall population, p=0.01) but not with ICU or in-hospital mortality (26.3% and 42% in CPE colonized patients versus 28.2 and 36.5%).

**Conclusions:** Hospitalization was the most important risk factor for CPE colonization. In this study and when compared with overall ICU population, patients colonized with CPE had significantly increased ICU length of stay. There seems to be a slightly higher in-hospital mortality rate, even though with no statistical significance.

### P076 Indian experience with ceftazidime-avibactam used in treatment of serious infections in ICU setting: subset analysis from the REPROVE and RECLAIM trials

#### M Naik, C Adhav, P Gupta

##### Pfizer, Medical Affairs, Mumbai, India

**Introduction:** Gram-negative pathogens—particularly Pseudomonas aeruginosa and Enterobacteriaceae—predominate in nosocomial pneumonia (NP) and cIAI both. These infections are becoming difficult to treat with available treatment options due to growing antimicrobial resistance in India. Ceftazidime-avibactam has in-vitro activity against Gram-negative organisms producing class A, class C and some class D beta-lactamases. We carried out a qualitative analysis to assess the safety and efficacy outcomes of the Indian population cohorts involved in the REPROVE and RECLAIM trials.

**Methods:** In line with the global REPROVE protocol, Indian patients enrolled in the study with NP, were randomly assigned (1:1) to 2000 mg ceftazidime and 500 mg avibactam or 1000 mg meropenem. In the RECLAIM study, Indian patients with a diagnosis of cIAI were enrolled in the study and were randomly assigned (1:1) to receive either ceftazidime-avibactam (2000 mg of ceftazidime and 500 mg of avibactam) followed by metronidazole (500 mg); or meropenem (1000 mg). The primary efficacy outcome measure in the REPROVE and RECLAIM studies was clinical cure rate of CAZ-AVI compared with that of meropenem at TOC (test-of-cure) visit in pre-defined analysis sets. In both studies, Non-inferiority was concluded if the lower limit of the two-sided 95% CI for the treatment difference was greater than –12·5% in the primary analysis sets. As the Indian subset study was not statistically powered to detect a difference in the sub-group, we descriptively analysed the efficacy results in the Indian population and compared them with the overall results in the global trial. In addition, the study also analysed the safety of CAZ-AVI in the Indian patients by monitoring the number and severity of adverse events.

**Results:** In the REPROVE study, 90 patients across 4 centres from India while the RECLAIM study comprised of 142 patients from 8 centers in India. The efficacy results from the Indian subset analysis are summarized in Table 1. The safety analysis set in the REPROVE study included 78 Indian patients and in the RECLAIM study included 125 Indian patients, results summarized in Table 2. Overall, efficacy and safety outcomes were comparable with the global study.

**Conclusions:** The safety and efficacy outcomes in Indian population treated with CAZ-AVI as part of the REPROVE and RECLAIM studies were in line with the global trials. Although there are inherent limitations to this analysis due to the subgroup size, it provides information on clinical efficacy and safety relevant for the intended use of CAZ-AVI in Indian patients. CAZ-AVI showed to be an effective option for critically patients with HAP and cIAI and may be considered a good alternative to carbapenems in the ICU setting for the treatment of resistant pathogens.

**Table 1 (abstract P076).** Clinical efficacy in Indian subset from REPROVE and RECLAIM studies

Clinical Cure at TOC (%) in CE population		Clinical Cure at TOC in cMITT(%)	
CAZ‑AVI	Meropenem	CAZ‑AVI	Meropenem
REPROVE study			
22/25 (88.0)	27/35 (77.1)	25/31 (80.6)	28/39 (71.8)
RECLAIM study			
45/46 (97.8)	42/44 (95.5)	50/56 (89.3)	50/59 (84.7)

**Table 2 (abstract P076).** Summary of adverse events (AE) upto last follow up visit (LFU) in REPROVE and RECLAIM studies

	INDIAN POPULATION		OVERALL POPULATION	
REPROVE study				
n (%)	CAZ-AVI (n=36)	MER (n=42)	CAZ-AVI (N=529)	MER (N=529)
Any AE	23 (63.9)	33(78.6)	302 (74.6)	299 (74.2)
Any AE leading to death	0	0	26(6.4)	23 (5.7)
Any serious AE	0	0	75(18.5)	54(13.4)
Any AE leading to discontinuation	0	0	16(4.0)	11(2.7)
Any AE of severe intensity	0	0	66 (16.3)	51 (12.7)
RECLAIM study				
n (%)	CAZ-AVI + MTZ (n=62)	MER (n=63)	CAZ-AVI + MTZ (N=529)	MER (N=529)
Any AE	44 (71.0)	35(55.6)	243 (45.9)	227 (42.9)
Any AE leading to death	1(1.6)	0	8 (1.5)	5 (0.9)
Any serious AE	1(1.6)	1(1.6)	42(7.9)	40(7.6)
Any AE leading to discontinuation	1(1.6)	2(3.2)	14(2.6)	191.6)
Any AE of severe intensity	1(1.6)	1(1.6)	30 (5.7)	36 (6.8)

### P077 Is there a difference in vancomycin level in patients admitted to medical compared to surgical ICU: a single center retrospective study in a mixed ICU setting

#### H Farhat^1^, R Jaafar^1^, R Farsakoury^1^, A Kassab^1^, D Ghaziri^2^, A Hallal^1^

##### ^1^American University of Beirut Medical Center, Surgery, Beirut, Lebanon; ^2^American University of Beirut Medical Center, Pharmacy, Beirut, Lebanon

**Introduction:** Adequate utilization of vancomycin is essential for achieving therapeutic targets while avoiding clinical failure and development of antimicrobial resistance.Our aim is to determine whether there is a difference in vancomycin therapeutic blood level between surgical and medical patients in intensive care unit (ICU) post-therapeutic dose.

**Methods:** A retrospective study was carried out in ICU at a tertiary health care center in Lebanon.Electronic health records of patients admitted to ICU who have received vancomycin between 2012 and 2017were reviewed.The sample size was calculated to detect 30%difference in sub-therapeutic level of vancomycin in patients admitted to medical compared to surgical ICU.Our power analysis estimated the sample to be 40 patients per group for ß=0.8 and a=0.05.Trough vancomycin levels below 15mg/l before the 4th dose were reported as sub-therapeutic. Descriptive analyses were conducted using number and percent.The Fisher exact test was used to compare outcomes across the different ICU services.Statistical analyses were performed using Statistical Analysis Software version 9.4.Statistical significance was set at a two-sided p-value of 0.05.

**Results:** A total of 44 surgical ICU patients with mean age 54.5+/-20.5 years and 41 medical ICU patients with mean age 60.6+/-17.93 years were obtained.Sixteen out of 44 surgical patients (36.3%) as compared to 8 out of 41 medical patients (19.5%) had sub-therapeutic levels (p= 0.068). Eight out of 44 surgical patients (18.1%) as compared to 17 out of 41 medical patients (41.4%) had trough level >20 mg/L (p=0.017). Twenty out of 44 surgical patients (45.4%) and 16 out of 41medical patients (39.02%) had therapeutic levels.

**Conclusions:** Our study showed that a higher percentage of surgical ICU patients have a sub-therapeutic level. We also noted that a statistically significant higher percentage of medical ICU patients had a supra-therapeutic vancomycin trough levels. Further studies are needed to reach optimal dosage of vancomycin in surgical ICU patient.

### P078 Piperacillin-tazobactam (TZP) dosing in septic patients: does obesity weight against efficiency?

#### P Alexandrzak^1^, C Lu^1^, N Van Grunderbeeck^2^, O Pouly^3^, F Lambiotte^4^, C Vinsonneau^5^, B Hennart^6^, D Thévenin^7^

##### ^1^CH Lens, Pharmacie Médicaments, Lens, France; ^2^CH Lens, Réanimation Polyvalente / USC, Lens, France; ^3^CHRU Lille, Réanimation Médicale, Lille, France; ^4^CH Valenciennes, Réanimation Polyvalente, Valenciennes, France; ^5^CH Beuvry, Réanimation Polyvalente, Béthune, France; ^6^CHRU Lille, Laboratoire de Toxicologie, Lille, France; ^7^CH Lens, Réanimation Polyvalente/USC, Lens, France

**Introduction:** Obese ICU patients display various disorders likely to impact drugs pharmacokinetic/pharmacodynamic (PK/PD) parameters. These phenomena make it hard to predict antibiotics PK/PD behavior, and thus to choose the appropriate dosing [1, 2]. Our study aims to determine whether the doses of TZP used to treat obese critically ill patients in ICU allow reaching an effective concentration, at the early phase of sepsis (24 hours after initiation).

**Methods:** A prospective observational study was performed on 12 obese (BMI >30 kg/m²) patients treated with TZP in 5 ICU. TZP maximal and minimal concentrations and concentration at 50% of the time between 2 administrations were determined and compared with targets of 8 and 4 Minimal Inhibitory Concentration (MIC) of Pseudomonas aeruginosa. Data such as treatment modalities, sequential organ failure assessment score, renal clearance and albumin were collected.

**Results:** Zero patient achieved the target of 8 MIC during 50% of the dosing interval. Four patients achieved the target of 4 MIC. The results are presented in Table 1. Several patients were treated using standard dosing or without loading dose. Continuous infusion was frequent.

**Conclusions:** TZP doses used in obese ICU patients do not reach targets to treat infections with resistant bacteria. It may exist room for improvement in this setting. Considering the risks of underdosing or toxicity, it seems preferable to use therapeutic drug monitoring to adapt TZP dosing in this population. Higher SOFA was associated with correct exposition, but doses used and renal function were different in these patients.

**References**

1) Roberts JA et al. Lancet Infect Dis. 2014 (6):498–509.

2) Taccone FS et al. Crit Care. 2010;14(4):R126.

**Table 1 (abstract P078).** impact of factors likely to influence PK/PD parameters on target achievement

		C<4MIC	C>4MIC	p value
Administration method	intermittent/extended/continuous	0/0/8	1/1/2	0.333
Dosing	Standard dose / Adjusted Body Weight /other	5/2/1	1/1/2	1
Loading dose		4	4	0.208
SOFA at TZP initiation	Median [Q1; Q3]	7 [5;8] (n=8)	11.5 [8;14] (n=4)	0.039
SOFA at sepsis peak	Median [Q1; Q3]	7.5 [5;9] (n=8)	12 [11;15] (n=3)	0.031
Albumin (g/L)	Median [Q1; Q3]	23.7 [20.6; 25.2] (n=6)	21 [18.4; 33] (n=3)	0.905
Renal clearance (ml/min)	Median [Q1; Q3]	45.2 [39; 100](n=5)	13.05 (n=1)	-

### P079 Pharmacokinetics of meropenem during continuous renal replacement therapy

#### N Hattori^1^, N Takahashi^1^, T Nakada^1^, Y Niibe^2^, T Suzuki^2^, S Yamazaki^2^, T Suzuki^2^, I Ishii^2^, S Oda^1^

##### ^1^Chiba university, Graduate school of medicine, Department of Emergency and Critical Care Medicine, Chiba-city, Japan; ^2^Chiba University Hospital, Department of Pharmacy, Chiba-city, Japan

**Introduction:** Early administration of effective intravenous antimicrobials is recommended for the management of the patients with sepsis. Although Meropenem (MEPM) is one of the first-line drugs in patients with sepsis because of its broad spectrum, the optimal dose in the critical care settings especially during continuous renal replacement therapy (CRRT) has not been established since therapeutic drug monitoring of MEPM has not been popular.

**Methods:** Eighteen critically ill patients who received CRRT were enrolled in this study. One gram of MEPM was administered over 1 hour, every 12 hours, and blood samples at 1, 2, 6, 9 and 12 hours after administration were collected on day 1, 2 and 5. All samples were stored at -80°C until analysis. The measurement of the blood concentration of MEPM was performed using high performance liquid chromatography with ultraviolet detection (HPLC-UV).

**Results:** The patients were 66.2±15.0 years old, 8 were male, their mean body weight was 71.7±20.8 kg, and their mean estimated creatinine clearance was 36.2±20.0 mL/min. CRRT was provided at 22.5±6.0 ml/kg/h. The peak concentration of the first administration of MEPM was 51.0±14.8 μ g/mL. All patients achieved time above minimum inhibitory concentration (TAM)> 50%, if minimum inhibition concentration (MIC) ≤4μ g/mL. Seventy eight % and 60% of the patients achieved TAM>50%, if MIC ≤8μ g/mL and ≤16μ g/mL, respectively.

**Conclusions:** One gram of MEPM every 12 hours was an appropriate dose for the patients who received CRRT.

### P080 Augmented renal clearance in critically ill patients: a single centre cohort study of creatinine clearance in intensive care.

#### B Johnston^1^, M Habgood^2^, D Perry^3^, M Joshi^4^, A Krige^3^

##### ^1^Royal Liverpool and Broadgreen University Hospital Trust, Critical Care, Liverpool, United Kingdom; ^2^East Lancashire Hospital Trust, Anaesthesia, Blackburn, United Kingdom; ^3^East Lancashire Hospital Trust, Critical Care, Blackburn, United Kingdom; ^4^University of Central Lancashire, Statistics, Preston, United Kingdom

**Introduction:** Augmented renal clearance (ARC) is defined as a creatinine clearance (CrCl) greater than 130ml/min/1.73m2. In patients with ARC, dose escalation may be appropriate to avoid sub-therapeutic plasma concentration of many drugs. [1]

The East Lancashire Hospital Trust ICU retrospectively analysed admissions for the development of ARC with the aim of characterising those patients who develop ARC.

**Methods:** We conducted a single centre retrospective cohort study between 2014 - 2016. Daily CrCl was recorded. Patients with renal impairment (serum creatinine levels >110mmol/l) or patients that underwent renal replacement therapy were excluded. The prevalence of ARC was determined, and multiple logistic regression was used to identify risk factors for developing ARC.

**Results:** ARC was observed in 624/1337 (47%) admissions. ARC was more common in males 56.9% compared to females 35.5% (p<0.001). ARC was significantly associated with younger age (p<0.001). There was no threshold phenomenon with age before which ARC was more likely. ARC was associated with lower APACHE II scores (p<0.001).

**Conclusions:** ARC is common in patients admitted to ITU. Our study is consistent with previous reports that suggest ARC affects between 35%-65% of admissions. [1] ARC in ITU patients can potentially lead to increased renal clearance of drugs and has been related to sub-therapeutic levels of antimicrobials, anticoagulants and anti-epileptic drugs. Research is needed to define the impact of ARC on drug clearance and dosage in ITU.[2]

**References**

[1] Bilbao-Meseguer I, et al. Clin. Pharmacokinet. 57:1107, 2018

[2] Udy AA, et al. Intensive Care Med;39:2070–2082, 2013

### P081 Concentration of meropenem in cerebrospinal fluid and serum in patients with meningoencephalitis

#### A Kuzovlev^1^, A Shabanov^2^, A Goloubev^1^, I Chernenkaya^3^, S Petrikov^3^, A Grechko^1^

##### ^1^Federal Research and Clinical Center of Intensive Care Medicine and Rehabilitology, Moscow, Russia; ^2^Federal Research and Clinical Center of Intensive Care Medicine and Rehabilitology, N.V. Sklifosofsky research center of emergency medicine, Moscow, Russia; ^3^N.V. Sklifosofsky research institute of emergency medicine, Moscow, Russia

**Introduction:** Meningitis is one of the complications of severe traumatic brain injury, and it is often associated with encephalitis (incidence from 1.3–4.8% to 10–20%). The aim of the investigation was to study the dynamics of the concentration of meropenem in serum and cerebrospinal fluid (CSF) with intravenous and intrathecal administration of meropenem.

**Methods:** In eight patients with bacterial meningoencephalitis blood serum and CSF were studied prior to the administration of meropenem and 5-10 min, 1, 2.5 and 5 hrs after it. Antibiotic regimen: 2000 mg of vancomycin (1000 mg BID) and meropenem (2000 mg TID diluted in 100 ml of saline IV + 20 mg BID diluted in 5 ml of saline bolus slowly intrathecally). Meropenem infusion was carried out for 30 minutes, 5 mins after it 5 ml of blood and 1 ml of CSF were sampled. Prior to antibiotics administration blood and CSF were taken for microbiological examination. To determine the concentration of antibiotics iquid chromatography/mass spectrometry was used. The samples were analyzed on an Agilent 1260 Infinity liquid chromatograph coupled to a Sciex QTrap 6500 mass detector (Sciex, US).

**Results:** Microbial growth was not detected in blood samples. Acinetobacter spp. was isolated in CSF (MIC of meropenem ≤8 mg/ml). Dynamics of meropenem concentrations: time 0 1.9 mg/ml (significantly lower than MIC – 8 mg/ml); directly after the infusion concentration of meropenem increased to 55.3 mg/ml and then gradually decreased reaching 5.4 mg/ml at 6 hrs. Concentration of meropenem in the CSF in all patients and at all time points significantly exceeded the MIC for Acinetobacter spp.

**Conclusions:** Combined (intravenous+intrathecal) administration of meropenem allows to achieve and maintain a constant concentration of meropenem in CSF, significantly exceeding the MIC of the antibiotic.

### P082 Carbapenems for the treatment of nosocomial pneumonia: a systematic review and meta-analysis

#### M Howatt^1^, J Muscedere^1^, A Kalil^2^, M Klompas^3^, M Metersky^4^

##### ^1^Queen´s University, Department of Critical Care, Kingston, Canada; ^2^University of Nebraska Medical Center, Department of Medicine, Omaha, United States; ^3^Harvard Medical School and Brigham & Women´s Hospital, Department of Medicine, Boston, United States;^4^University of Connecticut, Department of Medicine, Omaha, United States

**Introduction:** Inadequate empiric therapy for Hospital Acquired Pneumonia (HAP) and Ventilator Associated Pneumonia (VAP) is associated with increased morbidity and mortality. Carbapenems are an attractive choice for empiric therapy but there are concerns about overuse and development of resistance. To better understand the benefits and risks of using carbapenems in empiric treatment regimens for HAP and VAP, we conducted a systematic review and meta-analysis.

**Methods:** This review was conducted according to PRISMA guidelines and was registered (CRD42018093602). We included randomized controlled trials comparing carbapenem versus non-carbapenem regimens. Data were abstracted in duplicate. The primary outcome was all-cause mortality. Secondary outcomes included mortality by proportion of VAP and overall clinical response. We determined risk ratios (RR) for each outcome using a random-effects model.

**Results:** Of 14,369 unique references, 20 trials enrolling a total of 5534 patients were included. For the primary outcome of mortality, carbapenem use had a RR of 0.84 (95% CI 0.74 – 0.96, p = 0.01) (Figure 1). When stratified by proportion of VAP (<33%, 33-66%, >66%) RRs for mortality were 0.95 (0.77 – 1.17, p = 0.66), 0.78 (0.57 – 1.07, p = .13), and 0.81 (0.66 – 0.99, p = 0.04), respectively (not shown). Overall clinical response had a RR of 1.04 (0.99 – 1.09, p = 0.12) (Figure 2). Significant statistical heterogeneity was not found.

**Conclusions:** Carbapenem use for the treatment of HAP and VAP may be associated with a mortality benefit, particularly in populations with a high prevalence of VAP. However, there were no differences in clinical response rates. Limitations of available data include short-term patient follow up, high levels of potential bias, and heterogenous definitions of clinical response. Additional high-quality studies are needed to determine when the inclusion of carbapenems in empiric regimens is warranted.

**Fig. 1 (abstract P082).**
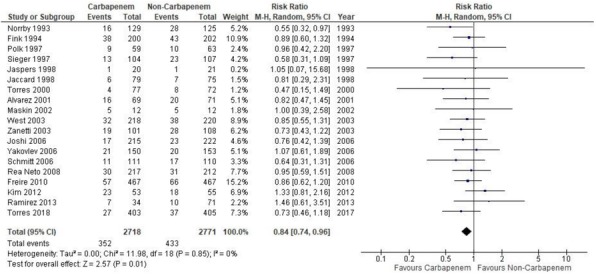
Patient mortality in carbapenem vs non-carbapenem regimens

**Fig. 2 (abstract P082).**
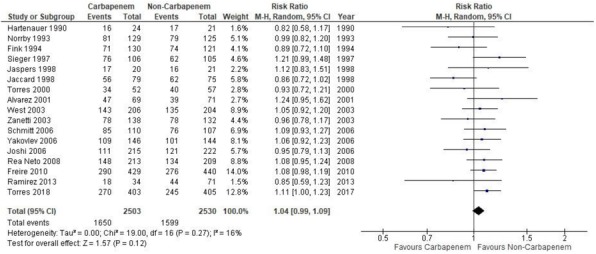
Overall clinical response

### P083 Impact of piperacillin/ tazobactam versus amoxicillin/ clavulanate as empirical treatment for Klebsiella pneumoniae bacteremia: a propensity score matched analysis

#### CY Chan, HP Shum, WW Yan, MY Man

##### Pamela Youde Nethersole Eastern Hospital, ICU, Chai Wan, Hong Kong

**Introduction:** Klebsiella pneumoniae (KP) bacteraemia is associated with high short-term mortality. As Extended-spectrum β-lactamases (ESBL) producing KP is less common in our locality, either Piperacillin/ Tazobactam (PIP-TAZO) or Amoxicillin/ Clavulanate (AMC) was commonly given as empirical treatment. However, whether PIP-TAZO could achieve a better clinical outcome as compared with AMC is not clear.

**Methods:** Adults admitted into a regional hospital in Hong Kong with KP bacteraemia from January 2009 to June 2017 were retrospectively reviewed. Demographics, antibiotics, microbiology and outcomes were identified and analyzed. Isolates resistant to either PIP-TAZO or AMC were excluded. Cox regression analysis and propensity score matching methods were used to determine predictors for 30-day mortality.

**Results:** A total of 322 patients with KP bacteremia were identified. Forty-nine patients (15.2%) required critical care and 56 (17.4%) died within 30 days of hospital admission (Table 1). Among all patients, 82 (25.5%) received PIP-TAZO while 240 (74.5%) received AMC. The unadjusted 30-day mortality was higher in PIP-TAZO group (25.6%) as compared with AMC group (14.6%) but patients in PIP-TAZO group were sicker. Propensity score matching yielded 61 matched pairs (122 patients). Their demographic and disease severity were well balanced (Table 2). No significant difference in 30-day mortality was noted between the matched cohorts (Fig 1). Cox regression analysis identified only respiratory tract and gastrointestinal tract infection as independent predictors for 30-day mortality while the use of PIP-TAZO was insignificant.

**Conclusions:** With the help of propensity score matching analysis, the apparently higher 30-day mortality among KP bacteremic patients received PIP-TAZO could be explained by their higher disease severity. This implies that the choice of empirical antibiotic, is affected by disease severity, and does not affect 30-day mortality.

**Table 1 (abstract P083).** Clinical characteristics and outcome parameters of all recruited patients

Parameters	Total (N=322)	Pip-Tazo (N=82)	Augmentin (N=240)	p-value
Septic shock	59 (18.3%)	29 (35.4%)	30 (12.5%)	<0.001
Total SOFA score (IQR)	3 (1-5)	4 (2-6)	2 (1-4)	<0.001
ICU care	49 (15.2%)	26 (31.7%)	23 (9.6%)	<0.001
30-day mortality	56 (17.4%)	21 (25.6%)	35 (14.6%)	0.023

**Table 2 (abstract P083).** Clinical characteristics and outcome parameters of matched cohort

Parameters	Total (N=122)	Pip-Tazo (N=61)	Augmentin (N=61)	p-value
Septic shock	35 (28.7%)	16 (26.2%)	19 (31.1%)	0.689
Total SOFA score (IQR)	4 (2-4)	4 (2-6)	4 (2-7)	0.601
ICU care	27 (22.1%)	13 (21.3%)	14 (23.0%)	1.000
30-day mortality	30 (24.6%)	17 (27.9%)	13 (21.3%)	0.529

**Fig. 1 (abstract P083).**
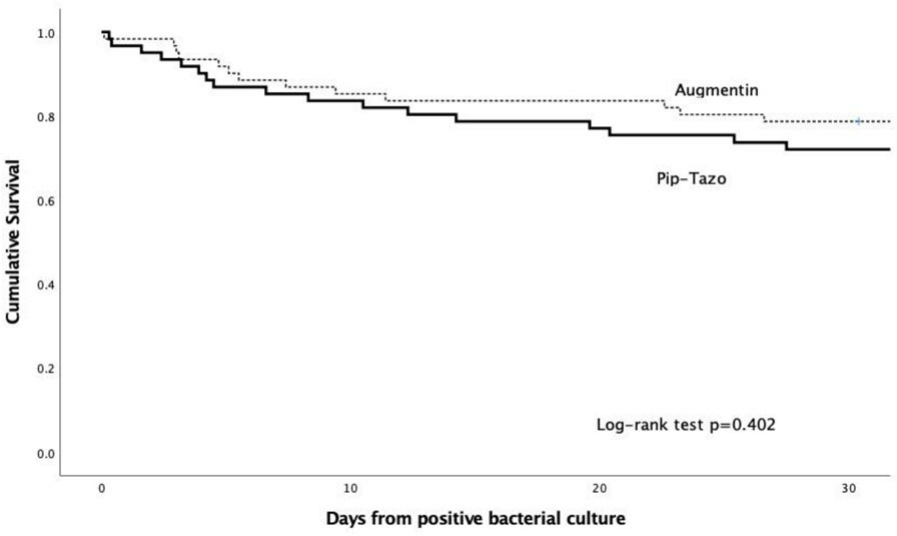
Kaplan-Meier Survival Plot showing probability of survival between two patient groups (matched cohort)

### P084 Ceftolozane-tazobactam in vitro activity in Pseudomonas aeruginosa and enterobacterales isolates recovered in intensive care units in Portugal. STEP study

#### M García-castillo^1^, S García-Fernández^1^, J Melo Cristino^2^, C Chaves^3^, H Ramos^4^, M Ribeiro^5^, M Feijó Pinto^6^, J Diogo^7^, E Gonçalves^8^, L Sancho^9^, R Ferreira^10^, V Alves^11^, E Ramalheira^12^, J Romano^13^, L Pássaro^13^, R Cantón^1^

##### ^1^Hospital Universitario Ramón y Cajal (IRYCIS), Madrid, Spain, Microbiology Department, Madrid, Spain; ^2^Centro Hospitalar Lisboa Norte – Hospital de Santa Maria, Lisboa, Portugal, Microbiology Department, Lisboa, Portugal; ^3^Centro Hospitalar Universitário de Coimbra, Coimbra, Portugal, Microbiology Department, Coimbra, Portugal; ^4^Centro Hospitalar do Porto, Porto, Portugal, Microbiology Department, Porto, Portugal; ^5^Centro Hospitalar de São João, Porto, Portugal, Microbiology Department, Porto, Portugal; ^6^Centro Hospitalar Lisboa Central, Lisboa, Portugal, Microbiology Department, Lisboa, Portugal; ^7^Hospital Garcia de Orta, Almada, Portugal, Microbiology Department, Almada, Portugal; ^8^Centro Hospitalar Lisboa Ocidental, Lisboa, Portugal, Microbiology Department, Lisboa, Portugal; ^9^Hospital Prof. Dr. Fernando da Fonseca, Amadora-Sintra, Portugal, Microbiology Department, Sintra, Portugal; ^10^Centro Hospitalar Universitário do Algarve, Portimão, Portugal, Microbiology Department, Portimao, Portugal; ^11^Unidade Local de Saúde de Matosinhos, Matosinhos, Portugal, Microbiology Department, Matosinhos, Portugal; ^12^Centro Hospitalar Baixo Vouga, Aveiro, Portugal, Microbiology Department, Aveiro, Portugal; ^13^MSD Portugal, MSD Portugal, Lisboa, Portugal

**Introduction:** Ceftolozane-tazobactam (C/T) is approved for complicated urinary tract infections (cUTIs), including pyelonephritis, and in combination with metronidazole for complicated intra-abdominal infections (cIAIs). The aim of the STEP study was to assess the in-vitro activity of C/T against clinical isolates prospectively collected from patients with cUTI, cIAIs and lower respiratory tract infections (LRTI) admitted ICUs in Portugal.

**Methods:** 396 Pseudomonas aeruginosa and 427 Enterobacterales isolates collected in 11 Portuguese hospitals (June 2017-July 2018) from patients with cUTIs, cIAIs and LRTI admitted at ICUs were included. Antimicrobial susceptibility was evaluated by standard ISO-broth microdilution for C/T and 16 other antimicrobials and interpreted using EUCAST guidelines.

**Results:** Isolates were recovered from cUTI (22.7%), cIAI (20.2%) and LRTI (57.1%). Activity of C/T and comparators by resistance phenotypes and source of infection in P. aeruginosa is shown in Table 1. The agents more active in P. aeruginosa were tobramycin, amikacin and C/T (88.9/88.9/86.4% susceptible, respectively). Activity of C/T in major species of Enterobacterales is shown in Table 2. The agents more active in E. coli were meropenem, colistin and ertapenem (98.3/98.3/98.3% susceptible), while in Klebsiella spp. were colistin, amikacin and meropenem (91.7/91.6/84.9% susceptible). Activity of C/T against ESBL-E. coli and -Klebsiella spp. was 76.3/21.3% susceptible. As expected, C/T had scarce activity against carbapenemase-phenotype.

**Conclusions:** C/T exhibited good overall activity against E. coli, although it might be affected in other Enterobacterales by local epidemiology, particularly due to carbapenemase producers. It was one of the most potent agents against P. aeruginosa, in which the activity was maintained regardless of the resistant phenotype. These results reinforce that C/T represents a therapeutic option in ICU patients with cUTIs, cIAIs and LRTI.

**Table 1 (abstract P084).** Activity of ceftolozane-tazobactam and comparators by resistance phenotypes in P. aeruginosa

	^3^C/T	PTZ	CAZ	MER
All P. aeruginosa (396)	86.4	64.1	57.8	75.5
^1^MDR (87)	86.2	51.7	41.4	79.3
^2^XDR (92)	54.3	6.5	8.7	19.6
cIAI-P. aeruginosa (80)	87.5	76.3	57.5	72.5
LTRI-P. aeruginosa (226)	87.6	59.7	55.3	75.7
cUTI-P. aeruginosa (90)	82.2	64.4	64.4	77.8

^1^MDR: Multi-drug resistant isolates; ^2^XDR: extremely drug resistant isolates; ^3^Ceftolozane-tazobactam (C/T), PTZ (Piperacillin-tazobactam), CAZ (Ceftazidime), MER (Meropenem)

**Table 2 (abstract P084).** Antimicromial activity of ceftolozane-tazobactam broken down by species and source of infection

	C/T Susceptibility (%)		
	IAI	LTRI	UTI
Enterobacterales (427)	69.7	51.6	73.2
E. coli (177)	94.4	64.7	93.5
Klebsiella spp. (132)	38.2	47.2	51.6
Enterobacter spp. (56)	61.1	56.5	26.7
Proteus spp. (25)	80.0	50.0	50.0

### P085 A high C-reactive protein/procalcitonin ratio predicts Mycoplasma pneumoniae infection

#### O Neeser^1^, T Vukajlovic^1^, L Felder^1^, S Haubitz^1^, A Hammerer-Lercher^2^, C Ottiger^2^, B Müller^1^, P Schütz^1^, C Fux^1^

##### ^1^Kantonsspital Aarau, Internal Medicine, Aarau, Switzerland; ^2^Kantonsspital Aarau, Laboratory Medicine, Aarau, Switzerland

**Introduction:** Differentiating Mycoplasma pneumoniae (MP) from Streptococcus pneumoniae (SP) and viral etiologies of community-acquired pneumonia (CAP) has important implications regarding empiric antibiotic therapy. We investigated parameters upon hospital admission to predict MP infection.

**Methods:** All patients hospitalized in a tertiary care hospital 2013 – 2017 for CAP with confirmed etiology were analyzed using logistic regression analyses and area under the receiver operator characteristics curves for associations between demographic, clinical and laboratory features and the causative pathogen.

**Results:** We analyzed 568 patients with CAP, including 47 (8%) MP; 152 (27%) with SP and 369 (65%) with influenza or other viruses. Comparing MP and SP, younger age (OR 0.56 per 10 years, 95% CI 0.42-0.73), lower neutrophil/lymphocyte ratio (OR 0.9, 0.82-0.99) and elevated CRP/PCT ratio (OR 15.04 (5.23-43.26) for a 400mg/g cut-off) independently predicted MP. With a ROC AUC of 0.91 (0.80 for the >400mg/g cutoff), the CRP/PCT ratio was the strongest predictor of MP versus SP. The CRP/PCT ratio also provided good discrimination between MP and viral infections (AUC 0.83; OR 5.55 for the >400mg/g cutoff, 2.26-13.64).

**Conclusions:** In patients hospitalized with CAP, a high admission CRP/PCT ratio predicts Mycoplasma pneumoniae infection and may improve empiric management.

### P086 Probiotics for the prevention of VAP and ICU-acquired infections in multi-trauma patients: a preliminary study

#### G Tsaousi, G Stavrou, Z Aidoni, K Fotiadis, K Kotzampassi

##### Department of Surgery, Aristotle University of Thessaloniki, Thessaloniki, Greece

**Introduction:** The prophylactic use of probiotics has emerged as a promising alternative to current strategies viewing to control nosocomial infections in a critically-ill setting. However, their beneficial role in VAP prevention remains inconclusive. Our aim was to delineate the efficacy of probiotics for both VAP prophylaxis and restriction of ICU-acquired infections in multi-trauma patients.

**Methods:** Randomized, placebo-controlled study enrolling 58 multi-trauma patients, requiring mechanical ventilation for >10 days. Participants were randomly assigned to receive either probiotic (n=28) or placebo (n=30) treatment. A four-probiotic formula was applied and each patient received two capsules per day from Day1 to Day15 post ICU admission. The content of one capsule was given as an aqueous suspension by nasogastric tube, while the other one was spread to the oropharynx after being mixed up with water-based lubricant. The follow-up period was 30 days, while ICU stay and mortality were also assessed.

**Results:** The use of probiotics reduced notably the incidence of VAP [32.1% vs 53.3%; p=0.001; RR placebo=3 (95%CI 1.6-5.4)], central venous line infection [21.4% vs 40%; p=0.031; RR placebo=4 (95%CI 2-7.9)], trauma-related infection [10.7% vs 26.6%; p=0.014; RR placebo=5.4 (95%CI 2.4-11.9)] or sepsis [14.3% vs 30%; p=0.005; RR placebo=5.2 (95%CI 2.3-11.4)] compared to placebo. Furthermore, fewer patients in probiotics group presented an ICU stay > 30 days [7.1% vs 40%; p=0.002; RR placebo=2.8 (95%CI 1.7-4.1)], while no difference in 30-day mortality rate was identified between groups (10.7% probiotics vs 6.7% placebo).

**Conclusions:** The prophylactic administration of probiotics exerted a positive effect on the incidence of VAP or other ICU- acquired infections and ICU stay in a critically-ill subpopulation being notorious for its high susceptibility to infections, namely multi-trauma patients.

### P087 Use of a C-reactive protein-based protocol to guide the duration of antibiotic therapy in critically ill patients: a randomized controlled trial

#### I Borges^1^, M Santana^1^, A Lana^1^, L Martins^2^, E Colosimo^2^, C Oliveira^3^, S Saturnino^3^, M Andrade^3^, C Ravetti^1^, V Nobre^1^

##### ^1^Faculdade de Medicina da Universidade Federal de Minas Gerais, Núcleo Interdisciplinar de Investigação em Medicina Intensiva - NIIMI, Belo Horizonte, Brazil; ^2^Faculdade de Medicina da Universidade Federal de Minas Gerais, Departamento de Estatística, Belo Horizonte, Brazil; ^3^Faculdade de Medicina da Universidade Federal de Minas Gerais, Departamento de Clínica Médica, Belo Horizonte, Brazil

**Introduction:** The rational use of antibiotics is one of the main strategies to limit the development of bacterial resistance. In this study we aimed to evaluate the effectiveness of a C reactive protein (CRP) based protocol in reducing antibiotic treatment time in critically ill patients.

**Methods:** An open randomized clinical trial was conducted in two adult intensive care units of a university hospital in Brazil (ClinicalTrials.gov: NCT02987790). Patients were randomly allocated to: i) intervention - duration of antibiotic therapy guided by CRP levels, and ii) control - duration of therapy based on best evidences for rational use of antibiotics. In the CRP group, antibiotic suspension was recommended after five full days of antibiotic therapy when CRP levels had fell > 50% of peak value (if peak > 100mg/L) or after three full days of antibiotic therapy when absolute values had reached values < 35mg/L (if peak <100mg/L). The primary outcome was days of antibiotic therapy in the index infection episode.

**Results:** 130 patients were included: 64 in the CRP group and 66 in the control group. The median (Q1-Q3) age was 61 (51-68) years, with SAPS 3 of 59 (50-70), without difference between groups. In the intention to treat analysis, the median (Q1-Q3) duration of antibiotic therapy for the index infection episode was 7.0 (5.0-8.8) days in the CRP group and 7.0 (7.0-11.3) days in the control group (p=0.011). In the cumulative suspension curve of antibiotics, a significant difference in the exposure time between the two groups was identified, with less exposure in the CRP group (p=0.007). In the pre-specified per protocol analysis, with 59 patients allocated in each group, the median duration of antibiotics was 6.0 (5.0-8.0) days in the CRP group and 7.0 (7.0- 10.0) days in the control group (p=0.011). Mortality and relapse rates were similar between groups.

**Conclusions:** Daily levels of CRP may aid in reducing the time of antibiotic therapy in critically ill patients, even in a scenario of judicious use of these drugs.

### P088 Hemophagocytic lymphohistiocytosis in the University Hospital of Infectious Diseases Cluj Napoca

#### L Herbel^1^, D Miclaus^1^, A Dicea^1^, A Muntean^1^, L Urian^2^, L Damian^3^, D Dima^2^, M Lupse^4^

##### ^1^University Hospital of Infectious Deseases, ICU, Cluj Napoca, Romania; ^2^Oncology Institute, Hematology, Cluj Napoca, Romania; ^3^University of Medecine and Pharmacy, Rhumatology, Cluj Napoca, Romania; ^4^University of Medecine and Pharmacy, ICU, Cluj Napoca, Romania

**Introduction:** The Macrophage Activation Syndrome (MAS) or hemophagocytic lymphohistiocytosis(HLH) is a life threatening complication characterized by pancytopenia, liver failure, coagulopathy and neurologic symptoms and is thought to be caused by the activation and uncontrolled proliferation of T lymphocytes and well differentiated macrophages, leading to widespread hemophagocytosis and cytokine overproduction [1,2].The etiology is unknown, but is considered to have an infectious trigger.The aim of our study is to evaluate the impact of HLH in our 9 beds infectious diseases ICU, during 83 months period (2012-2018).

**Methods:** A retrospective study based on electronic databases, including all patients admitted in our ICU, that have matched at least 5 out of 8 criteria for HLH diagnosis (1):fever; hepato-splenomegaly; >2 cytopenia (Hb <9 g/dl, PLT</dl, PMN -N<1000/mmc); hypertriglyceridemia>265mg/dl, fibrinogen<150mg/dl; hemophagocytosis- bone marrow, spleen, and/or lymphnodes; NK activity reduced/absent; ferritin level>500UI/L; CD 25>2400. We have evaluated the etiology established with cultures, serology, and molecular methods, treatment with corticosteroids, IV immunoglobuline, cyclosporine, etoposide and outcome (2).

**Results:** 2112 patients were admitted to ICU, 15 patients(0.71%) met the criteria for HLH. The average length of stay in ICU was 15 days; 6 patients died (40%) without relation with the followed treatment.

**Conclusions:** HLH is not a rare condition in infectious diseases ICU. The etiology is more frequent established compared with literature data. Treatment (corticosteroids, immunoglobuline, cyclosporine, etoposide) is not associated with increased survival

**References**

(1) Henter JL et al: Pediatr Blood Cancer 2007;48(2)124-31

(2) Stuart J.Carter et al: Rheumatology review, Oxford Univ Press 22 feb 2018

### P089 Forecasting hemorrhagic shock using patterns of physiologic response to routine pre-operative blood draws

#### X Li^1^, M Pinsky^2^, G Clermont^2^, A Dubrawski^1^

##### ^1^Carnegie Mellon University, Auton Lab, Pittsburgh, United States; ^2^University of Pittsburgh, School of Medicine, Pittsburgh, United States

**Introduction:** Irreversible hemorrhagic shock (IHS), a critical condition associated with significant blood loss and poor response to fluid resuscitation, can induce multiple organ failures and rapid death [1]. Determining the patients who are likely to develop IHS in surgeries could greatly help pre-operative assessment of patient outcomes and allocation of clinical resources.

**Methods:** machine learning model of IHS is developed and validated via porcine induced bleed experiment. 36 healthy sedated Yorkshire pigs first had one 20mL rapid blood draw during a stable period, and then were bled at 20mL/min to mean arterial pressure (MAP) of 30 mmHg. 10 subjects had IHS defined as MAP<20mmHg. Arterial, central venous and airway pressures collected at 250 Hz during the blood draw [Fig 1] were used to extract characteristic sequential patterns using Graphs of Temporal Constraints (GTC) methodology [2], and a decision forest (DF) model was trained on these patterns to determine subjects at high risk of impending IHS.

**Results:** In a leave-one-subject-out cross-validation, our method confidently identifies 30% (95% CI [15.6%, 44.4%]) of the subjects who are likely to experience IHS when subject to substantial bleeding, while only giving on average 1 false alarm in 10,000 such predictions. This method outperforms logistic regression and random forest models trained on statistically featurized data [Tab 1, Fig 2].

**Conclusions:** Our results suggest that by leveraging sequential patterns in hemodynamic waveform data observed in pre-operative blood draws, it is possible to predict who are prone to develop IHS resulting from blood loss in the course of surgery. Future work includes validating the proposed method on data collected from human subjects, and developing a clinically useful screening tool with our investigations.

Work partially funded by NIH GM117622.

**References**

[1] Gutierrez et al. Critical care. Crit Care, 8(5):373, 2004.

[2] Guillame-Bert et al. JMLR, 18(121):1–34, 2017.

**Table 1 (abstract P089).** Comparison of model performances with 95% confidence intervals in square brackets

Model	TPR at low FPR (0.0001)	TNR at low FNR (0.0001)	AUC
Logistic regression	0.1 [0, 0.201]	0.462 [0.307, 0.616]	0.819 [0.696, 0.943]
Random forest	0 [0, 0.048]	0.231 [0.097, 0.364]	0.519 [0.364, 0.674]
GTC-DF	0.3 [0.156, 0.444]	0.423 [0.269, 0.577]	0.810 [0.684, 0.935]

**Fig. 1 (abstract P089).**
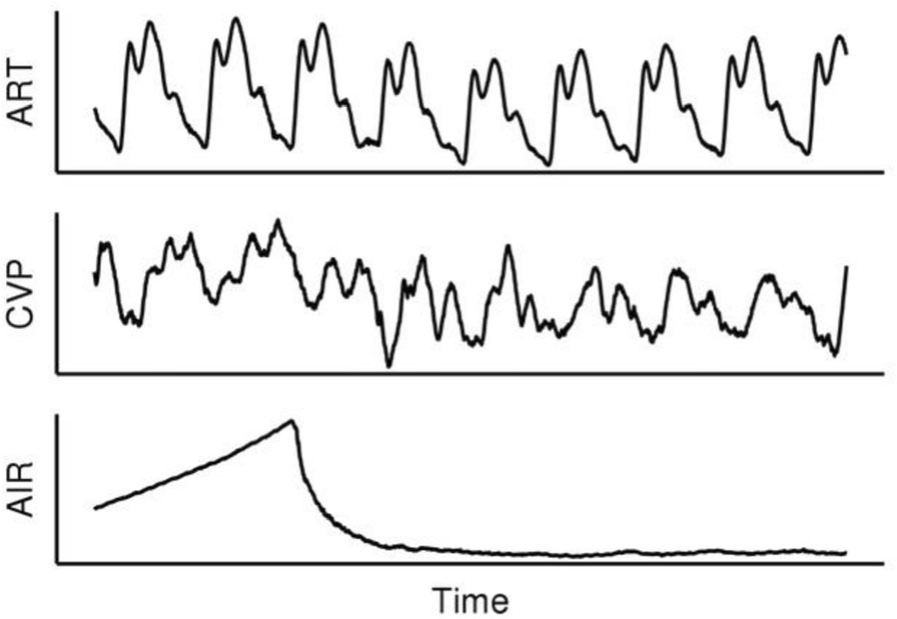
An example 5-second segment of three hemodynamic variables used in our models (Arterial Pressure (ART), Central Venous Pressure (CVP), Airway Pressure (AIR)) extracted from one subject

**Fig. 2 (abstract P089).**
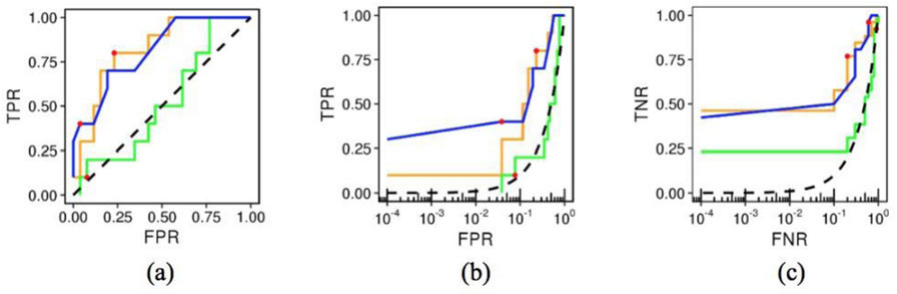
ROC curves of logistic regression (orange), random forest (green) and GTC-DF (blue). False Positive Rates (FPR) and False Negative Rates (FNR) in (b) and (c) are logarithmically scaled to emphasize performance at clinically relevant low FPR and FNR settings, respectively. FPR and TPR corresponding to 50% sensitivity threshold for each model is marked with a red dot

### P090 Oxytocin and hydrogen sulfide expression in porcine brain after hemorrhagic shock

#### N Denoix^1^, T Merz^2^, M Wepler^2^, H Guendel^1^, C Waller^3^, P Radermacher^2^, O McCook^2^

##### ^1^Ulm University Medical Center, Clinic for Psychosomatic Medicine and Psychotherapy, Ulm, Germany; ^2^Ulm University Medical Center, Institute for Anesthesiological Pathophysiology and Process Engineering, Ulm, Germany; ^3^Universitätsklinik der Paracelsus Medizinischen Privatuniversität, Clinic for Psychosomatic Medicine and Psychotherapy, Nuernberg, Germany

**Introduction:** The H_2_S and oxytocin(Oxy) systems are reported to interact with one another [1]. H_2_S plays a major role in the hypothalamic control of Oxy release during hemorrhage [2]. There is scarce information about Oxy receptor(OxyR) expression in the brain in general and what is there is ambivalent. OxyR has been immunohistochemically(IHC) detected in the human hypothalamus but not in the hippocampus, in contrast to rodents [3], which underscores the need for additional characterization in relevant animal models. Thus the aim of this study is to map the expression of the Oxy and H_2_S systems in the porcine brain in a clinically relevant model of hemorrhagic shock (HS).

**Methods:** Anesthesized atherosclerotic pigs (n=9) underwent 3h of HS (MAP 40+/-5mmHg) [4], followed by 72h resuscitation. IHC detection of Oxy, OxyR, the H2S producing enzymes cystathionine-γ -lyase (CSE) and cystathionine-β -synthase(CBS) was performed on formalin fixed brain paraffin sections.

**Results:** Oxy, OxyR, CSE and CBS were localized in the porcine brain. Proteins were differentially expressed in the hypothalamus (Fig 2), parietal cortex and cerebellum (Fig 1). Cell types positively identified were: magnocellular neurons of the hypothalamus, cerebellar Purkinje cells and granular neurons, and hippocampal pyramidal and granular neurons of the dentate fascia. Arteries and microvasculature were also positive for OxyR and CSE.

**Conclusions:** Our results confirm the presence of Oxy and OxyR in the hypothalamus similarly to the human brain. Novel findings were: OxyR in the cerebellum and CSE expression in the hypothalamus and cerebellum. The coexpression of OxyR and CSE may link and help better understand neurochemical systems and physiological coping in hemorrhagic shock. Funding: CRC1149

**References**

[1] Merz T et al. Intensive Care Med Exp 6:41, 2018

[2] Ciosek J et al. J Physiol Pharmacol 54, 2, 233-246, 2003

[3] Boccia ML et al. Neuroscience 253, 155–164,2013

[4] Hartmann C et al. Crit Care Med 45(12):e1270-e1279, 2017

**Fig. 1 (abstract P090).**
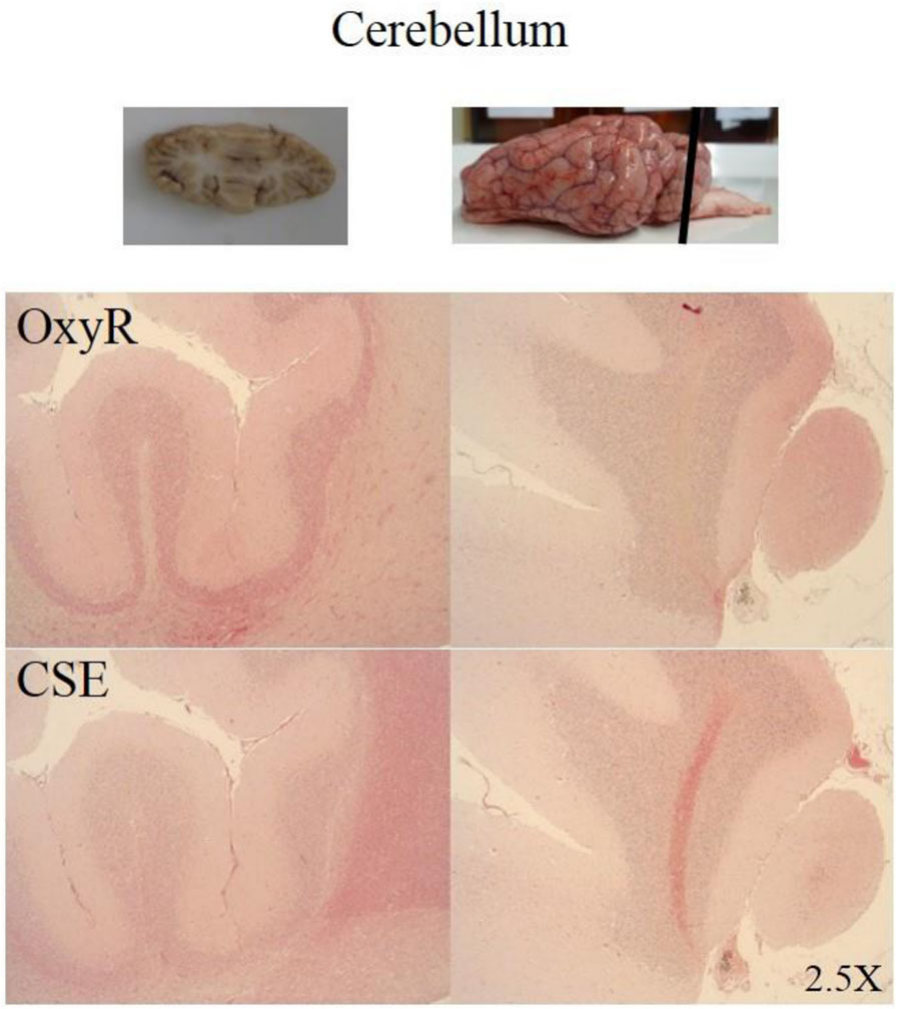
See text for description

**Fig. 2 (abstract P090).**
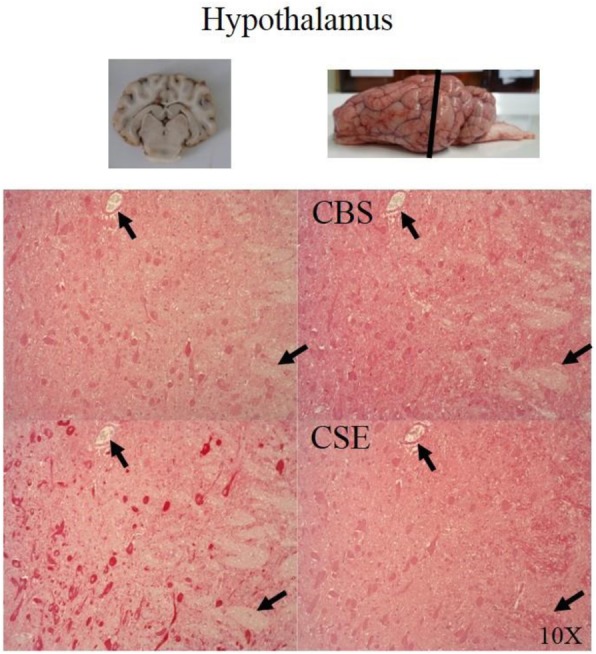
See text for description

### P091 Impact of mean arterial pressure and norepinephrine administration during the first 48 hours in septic shock patients

#### F Torrini, A Dell´Anna, S Muscolino, T Taccheri, S Carelli, R Maviglia, M Antonelli

##### Fondazione Policlinico Universitario A. Gemelli-IRCCS, Dept. of Anesthesia and Intensive Care Medicine, Rome, Italy

**Introduction:** Septic shock is one of the main causes of intensive care unit (ICU) admission, leading to mortality up to 50% of patients. Acute kidney injury (AKI) frequently occurs and is associated to great morbidity and mortality. Hemodynamic optimization may reduce the incidence of AKI, but the use of vasopressors to increase mean arterial pressure (MAP) could have deleterious effect on renal perfusion. We aimed at investigating the effect of MAP and norepinephrine (NE) on the incidence of AKI in septic shock patients

**Methods:** Retrospective study based on prospectively collected data on digital medical records (Digistat) at our ICU. Adult patients with a diagnosis of septic shock surviving at least 48 hours between 01/2010 and 01/2015 were included. We collected data on hemodynamic parameters, doses of vasoactive/inotropic drugs hourly in the first 48h and clinical scores. Hemodynamic data were analyzed individually as well as in their average, maximum and minimum value and the AUCs were calculated.Patients were categorized on the basis of NE dose (NE<0.5,0.5<NE<1,NE>1 mcg/kg/min). AKI was defined according to KDIGO guidelines.

**Results:** 220 patients were included. Among these, 131 (59.5%) died at day 60 and 134 (60.9%) developed AKI. Average MAP was lower in patients who developed AKI (79 vs 83 mmHg, p= .005), even after adjustment for the average dose of NE (mean difference 2.3 mmHg [95% CI 0.3-4.4]; p= .026). Conversely, different NE doses were not associated to a significant difference in the occurrence of AKI, while MAP was significantly lower at higher doses of NE (83 vs 82 vs 77 mmHg, p< .001). The average MAP had a low predictive value on the occurrence of AKI, but was significantly related to an increased risk of AKI even after adjustment for other variables (OR 0.96, 95% CI 0.93-0.99)

**Conclusions:** Average MAP in the first 48 hours in septic shock patients is significantly related to the occurrence of AKI, regardless of the average dose of norepinephrine administrated

### P092 Achievement of an international consensus target mean arterial pressure following initiation of angiotensin II in patients with catecholamine-resistant distributive shock

#### L Forni^1^, MT McCurdy^2^, AK Khanna^3^, LW Busse^4^, L Chawla^5^, J Hästbacka^6^

##### ^1^Royal Surrey County Hospital, Guildford, United Kingdom; ^2^University of Maryland School of Medicine, Baltimore, United States; ^3^Cleveland Clinic Foundation, Cleveland, United States; ^4^Emory University, Atlanta, United States; ^5^La Jolla Pharmaceutical Company, San Diego, United States; ^6^University of Helsinki and Helsinki University Hospital, Helsinki, Finland

**Introduction:** In patients with distributive shock, increasing mean arterial pressure (MAP) to a target of >65 mmHg can improve tissue perfusion. Patients unable to achieve the target MAP of >65 mmHg despite adequate fluid resuscitation as well as catecholamines and vasopressin standard care (SC), may benefit from the non-catecholamine vasopressor angiotensin II to increase MAP. This post-hoc analysis examined whether patients from the ATHOS-3 study with a baseline (BL) MAP <65 mmHg and treated with SC plus either angiotensin II (Ang II) or placebo achieved a MAP of >65 mmHg for 3 consecutive hours, without increasing the dose of SC therapy.

**Methods:** Patients were assigned in a 1:1 ratio to receive Ang II or placebo, plus SC. Randomization was stratified according to MAP (<65 or >65 mmHg) at screening. In patients with BL MAP <65 mmHg, we evaluated whether patients achieved a MAP of >65 mmHg for the first 3 hours after initiation (MAP measurements taken at hours 1, 2, and 3), without an increase in the dose of SC.

**Results:** Among 321 treated patients, 102 had BL MAP <65 mmHg (Ang II, 52; placebo, 50). Median BL MAP (IQR) was 61 (57-63) and 62 (59-64) mmHg for placebo and Ang II groups, respectively. Patients with BL MAP <65 mmHg who were treated with Ang II were more likely to achieve MAP ≥65 mmHg for 3 consecutive hours after initiation without an increase in SC dose (67%, 95%CI 53-80), compared with placebo-treated patients (24%, 95%CI 13-38, OR=6.52, p<0.0001).

**Conclusions:** In this post-hoc analysis of patients with BL MAP <65 mmHg, patients receiving Ang II plus SC were significantly more likely to achieve a MAP >65 mmHg for the first 3 consecutive hours after initiation than patients receiving SC only. This suggests that administering Ang II may help patients with catecholamine-resistant distributive shock to achieve the consensus standard target MAP.

### P093 Norepinephrine synergistically increases the efficacy of volume expansion on venous return in septic shock

#### I Adda, C Lai, JL Teboul, L Guerin, F Gavelli, C Richard, X Monnet

##### Hôpitaux universitaires Paris-Sud, Hôpital de Bicêtre, APHP, Service de médecine intensive-réanimation, Le Kremlin-Bicêtre, France

**Introduction:** Through reduction in venous capacitance, norepinephrine (NE) increases the mean systemic pressure (Psm) and increases cardiac preload. This effect may be added to the ones of fluids when both are administered in septic shock. Nevertheless, it could be imagined that NE potentiates in a synergetic way the efficacy of volume expansion on venous return by reducing venous capacitance, reducing the distribution volume of fluids and enhancing the induced increase in stressed blood volume. The purpose of this study was to test if the increase in Psm induced by a preload challenge were enhanced by NE.

**Methods:** This prospective study had included 30 septic shock adults. To reversibly reproduce a volume expansion and preload increase at different doses of NE, we mimicked fluid infusion through a passive leg raising (PLR). In patients in which the decrease of NE was planned, we estimated Psm (using respiratory occlusions) at baseline and during a PLR test (PLR_High_). The dose of NE was then decreased and Psm was estimated again before and during a second PLR (PLR_Low_).

**Results:** NE dose decreased from 0.32[0.18-0.62] to 0.26[0.13-0.50] μ g/kg/min (p<0.001). The increase in Psm induced by PLR_High_ (Δ Psm) at the highest dose of NE was significantly greater than the Δ Psm induced by PLR_Low_ (35[20-43]% vs 14[8.8-20]%, p<0.001). The increase in cardiac index induced by PLR_Low_ was significantly greater than that induced by PLR_High_ (p<0.001). Δ PsmHigh - Δ PsmLow was moderately correlated with the diastolic arterial pressure at BaselineHigh (p=0.03, r=0.41) and with the NE-induced change in mean arterial pressure (p=0.004, r=0.57).

**Conclusions:** NE enhances the increase in Psm induced by a PLR, which mimics a fluid infusion. This suggests that it may potentiate the effects of fluid in a synergetic way in septic shock patients. This may decrease the amount of administered fluids and contribute to decrease the cumulative fluid balance.

### P094 Predictors of the response to vasopressin in ICU patients with norepinephrine-resistant hypotension: a single-center, retrospective study

#### Y Yasuda, D Kasugai, M Nishikimi, M Higashi, T Yamamoto, M Ozaki, A Numaguchi, N Matsuda

##### Nagoya University Graduate School of Medicine, Department of Emergency and Critical Care, Nagoya, Aichi, Japan

**Introduction:** Arginine vasopressin (AVP) can be used in addition to norepinephrine (NE) for NE-resistant septic shock. However, a subgroup who will response to AVP is unknown. The purpose of this study was to determine factors which could predict the response to AVP in patients with NE-resistant hypotension.

**Methods:** This was a single-center, retrospective analysis of patients who administered AVP for NE-resistant hypotension in our intensive care units (ICUs). Eligible patients were adult patients who administered AVP in addition to NE due to hypotension (mean arterial pressure (MAP) < 65) in our ICUs between August 2014 and December 2017. We divided all patients into two groups by response to AVP; responders and non-responders. The responders were defined as an increase of MAP ≥ 10 mmHg at 1h after AVP initiation. We conducted univariate and multivariate logistic regression analysis to evaluate the effect of variables on AVP response.

**Results:** A total of 163 patients were included; 107 responders (66%), 56 non-responders (34%). There was no significant difference for MAP at the time of AVP initiation (51 vs 52 mmHg; p = 0.40), initiation dose of AVP (0.028 vs 0.031 U/min; p = 0.67), and dose of NE at the time of AVP initiation (0.18 vs 0.18 μ g/kg/min; p = 0.95). MAP at 1h after AVP initiation was significantly higher in responders than non-responders (74 vs 55 mmHg; p < 0.01). Responders were older (69 vs 66; p = 0.02) and had lower heart rate (HR) (99 vs. 108; p = 0.01) and lactate (3.2 vs. 4.8 mmol/L; p = 0.02) at the time of AVP initiation. The multivariate logistic analysis revealed that HR ≤ 103 (OR 2.56, 95% CI 1.29-5.10, p < 0.01), lactate ≤ 3 (OR 3.11, 95% CI 1.56-6.19, p < 0.01) and age ≥ 63 (OR 2.16, 95% CI 1.05-4.45, p = 0.04) were significantly associated with the response to AVP.

**Conclusions:** HR, lactate levels and age before AVP initiation can predict the response to AVP in ICU patients with NE-resistant hypotension.

### P095 The maximum norepinephrine dosage of initial 24 hours predicts early death in septic shock

#### D Kasugai^1^, A Hirakawa^2^, N Jinguji^3^, K Uenishi^3^

##### ^1^Nagoya university Gtaduate School of Medicine, Department of Emergency and Critical Care, Nagoya, Aichi, Japan; ^2^Fujita Health University, Department of Disaster and Traumatology, Fujita Health University, Toyoake, Japan; ^3^Fujita Health University Hospital, Department of Emergency and General Internal Medicine, Fujita Health University Hospital, Toyoake, Japan

**Introduction:** The mortality of septic shock refractory to norepinephrine remains high. To improve the management of this subgroup, the knowledge of early indicator is needed. We hypothesize that maximum norepinephrine dosage on the initial day of treatment is useful to predict early death in septic shock.

**Methods:** In this retrospective single-center observational study, septic shock patients admitted to the emergency intensive care unit (ICU) of an academic medical center between April 2011 and March 2017 were included. Cardiac arrest before ICU admission and those with do-not-resuscitate orders before admission were excluded. The maximum dosage of norepinephrine initial 24 hours of ICU admission (MD24) was used to assess 7-day mortality.

**Results:** One-hundred-fifty-two patients were included in this study. Median SOFA score was 11 (9-13), and median MD 24 was 0.24 (0.16-0.38) mcg/kg/min. Vasopressin and steroid were administered in 58 (38 %) and 67 (44 %) cases. Nineteen patients (13 %) died within a week. Non-survivors had higher MD24, higher SOFA score, and higher rate of vasopressin use. The higher MD24 predicted 7-day mortality (area under curve 0.797, threshold 0.60 mcg/kg/min, sensitivity 50 %, specificity 91%). After adjustment of inverse probability of treatment weighing method using propensity scoring, MD24 higher than 0.6 mcg/kg/min was independently associated with 7-day mortality (OR: 9.96, 95 %CI: 2.56-38.8, p < 0.001).

**Conclusions:** The maximum dosage of norepinephrine higher than 0.6 mcg/kg/min initial 24 hours was significantly associated with 7-day mortality in septic shock, and may be useful in the selection of higher severity subgroup.

### P096 The impact of norepinephrine on right ventricular function and pulmonary haemodynamics in patients with septic shock - a strain echocardiography study

#### K Dalla

##### Sahlgrenska University Hospital Mölndal, Göteborg, Sweden

**Introduction:** Septic shock is characterized by myocardial depression and severe vasoplegia. Right ventricle performance could be impaired in sepsis. The effects of norepinephrine on RV performance and afterload in septic shock are not immediately evident. The aim of the present study was to investigate the effects of norepinephrine on RV systolic function, RV afterload and pulmonary haemodynamics.

**Methods:** Eleven, volume-resuscitated and mechanically ventilated patients with norepinephrine-dependent septic shock were included. Infusion of norepinephrine was randomly and sequentially titrated to target mean arterial pressures (MAP) of 60, 75 and 90 mmHg. At each target MAP, strain- and conventional echocardiographic were performed. The pulmonary haemodynamic variables were measured by using a pulmonary artery thermodilution catheter. The RV afterload was assessed by calculating the effective pulmonary arterial elastance (Epa) and pulmonary vascular resistance index (PVRI).

**Results:** The norepinephrine-induced elevation of MAP increased central venous pressure (38%, p<0.001), stroke volume index (7%, p<001), mean pulmonary artery pressure (19%, p<0.001) and RV stroke work (20%, p=0.045), while neither pulmonary vascular resistance index nor Epa was affected. Increasing doses of norepinephrine improved RV free wall strain from -19% to -25% (32%, p=0.003), tricuspid annular plane systolic excursion (22%, p=0.010) and tricuspid annular systolic velocity (17%, p=0.029). There was a trend for an increase in cardiac index assessed by both thermodilution (p=0.079) and echocardiography (p=0.054).

**Conclusions:** The RV function was improved by increasing doses of norepinephrine, as assessed both by strain- and conventional echocardiography. This is explained by an increase of RV preload. Pulmonary vascular resistance is not affected by increased doses of norepinephrine.

### P097 Peripheral perfusion versus lactate-targeted fluid resuscitation in septic shock: the ANDROMEDA SHOCK physiology study. preliminary report

#### G Hernandez^1^, R Castro^1^, L Alegría^1^, S Bravo^1^, D Soto^1^, E Valenzuela^1^, M Vera^1^, V Oviedo^1^, C Santis^2^, G Ferri^2^, M Cid^2^, B Astudillo^2^, P Riquelme^2^, R Pairumani^2^, G Ospina-Tascón^3^, J Bakker^1^

##### ^1^Pontificia Universidad Católica de Chile, Departamento de Medicina Intensiva, Santiago, Chile; ^2^Hospital Barros Luco-Trudeau, Unidad de Pacientes Críticos, Santiago, Chile; ^3^Fundación Valle del Lili, Universidad ICESI, Department of Intensive Care Medicine, Cali, Colombia

**Introduction:** The potential role of peripheral perfusion as a target for fluid resuscitation (FR) in septic shock is unknown. We aimed to determine if capillary refill time-targeted FR (CRT) is superior to a lactate-targeted fluid resuscitation (L) regarding fluid balances and evolution of tissue-perfusion variables.

**Methods:** Clinical RCT in 2 ICUs (sample size of 46 pts). Fluid responsive septic shock patients were randomized into 2 groups with FR aimed at normalizing CRT or at normalizing or decreasing lactate at rates >20%/ 2h during a 6h period. Hemodynamic and perfusion variables were assessed at 2, 6 and 24h, as well as SOFA scores and fluid balances up to 72h. Tissue perfusion variables were assessed in parallel (NIRS, sublingual microcirculation, LiMON, lactate/pyruvate and central venous-arterial pCO2 to arteriovenous O2 content difference ratio)

**Results:** Eighteen patients (mean age 66 [42,74], APACHE II 23 [14,30], SOFA 10 [7,12] have been included until now (8 in L group and 10 in CRT group). Baseline lactate was 3.6 [3.1,5.0] vs 3.8 [3.0, 4.7] mmol/l; and CRT was 4.5 [2.5, 6.5] vs 4.5 [3.0, 6.0] secs, in L and CRT groups, respectively. No differences between groups were observed in macrohemodynamics at baseline. Resuscitation fluids at 6h were not different 1.00 [0.50,4.25] vs 1.13 [0.25,2.00] liters in L and CRT groups, respectively, with a trend to less positive 24h fluid balance in CRT group 3.25 [0.55,4.65] vs 2.25 [0.81,4.58] liters. StO2 and ICG/PDR were not different. The results of some perfusion related parameters are shown in Table 1.

**Conclusions:** This preliminary results suggest that using CRT as a target for FR in septic shock appears to be feasible, and not associated with impairment of tissue perfusion-related parameters as compared to lactate-targeted FR.

Grant FONDECYT Chile 1170043

**Table 1 (abstract P097).** Hemodynamics and Tissue perfusion variables

	Groups	Baseline	6 hours
L/P ratio	L	10.6 [6.4,20.9]	11.4 [8.0,12.7]
	CRT	11.2 [9.3,14.5]	15.1 [12.0,25.2]
Cv-aCO2/Da-vO2	L	1.1 [0.7,4.0]	0.8 [-0.8,1.5]
	CRT	0.4 [0.2,0.4]*	0.7 [0.2,1.1]
MFI	L	2.75 [2.25,3.00]	2.75 [2.75,3.00]
	CRT	1.63 [0.94,2.50]	2.50 [0.88,2.63]*

### P098 Efficacy comparison of angiotensin II and vasopressin in a norepinephrine-resistant hypotension pig model

#### S Li^1^, J Wilkie^1^, H Jin^1^, D Merrill^2^, M Frierson^1^, L Johnson^1^, D Zhou^1^, R Marsden^1^, S Chen^1^, A Seacat^1^, L Chawla^1^, G Tidmarsh^1^, J Rolke^1^

##### ^1^La Jolla Pharmaceutical Company, San Diego, United States; ^2^Da Vinci Biomedical Research Products, Inc., South Lancaster, United States

**Introduction:** Shock patients often become resistant to catecholamines which often require the addition of a non-catecholamine vasopressor. Preclinical studies suggest that in the presence of a-adrenoceptor antagonism, the renin-angiotensin aldosterone system exerts the major vasopressor influence. We sought to determine the effects of AngII or Lypressin (LYP [porcine vasopressin]) on blood pressure in a norepinephrine (NE)-resistant hypotension pig model.

**Methods:** Phentolamine (PHN), a reversible α-blocker that antagonizes the vasoconstriction by NE, was continuously infused to induce hypotension. After NE-resistant hypotension was established, LYP or AngII was then co-infused with PHN. Mean arterial pressure (MAP) and heart rate were continuously recorded (Fig. 1).

**Results:** As shown in Fig. 2, PHN reduced MAP to 75.3±9.2% from the baseline (100±13.8%) and blunted the MAP increase induced by 0.2μg/kg/min NE from 193±14.4% to 130.2±15.4%. Additional 0.3μg/kg/min NE didn’t further increase MAP (130±17.6%), indicating a steady state of NE resistance. AngII at 80 (1x highest label dose), 160 (2x) and 320 (4x) ng/kg/min all significantly increased the MAP over baseline (143.6±18.3, 158.3±13.5 and 168.5±14.4%, respectively) in a dose-dependent fashion. Prior to PHN infusion, a 2.1-unit bolus of LYP was administered which confirmed an increase in MAP. After PHN infusion, LYP was administered at 0.07 (1x highest label dose), 0.21 (3x), 0.7 (10x) and 2.1 (30x) U/min resulting in no increase over baseline MAP (76.9±14.7, 85.4±13.9, 98.8±14.1 and 93±9.7%, respectively).

**Conclusions:** In a background of α-adrenoceptor blockade, at clinically comparable doses, the vasopressor effect of Ang II was maintained while those of NE and LYP were attenuated. These data suggest that the blood pressure effect of vasopressin-like peptides may require a functioning α-adrenoceptor. Patients with shock who are resistant to increasing doses of catecholamines may also have vasopressin resistance potentially making angiotensin II a preferred vasopressor for these patients.

**Fig. 1 (abstract P098).**
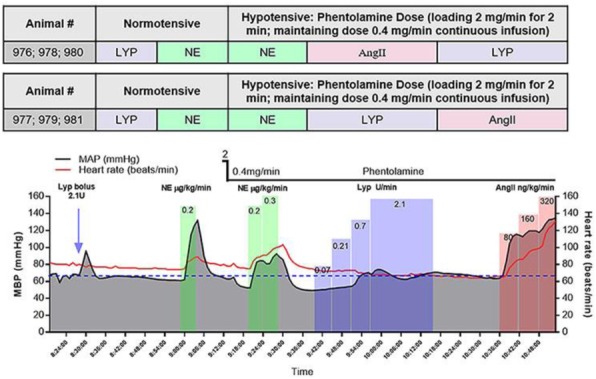
Study design and representative individual data

**Fig. 2 (abstract P098).**
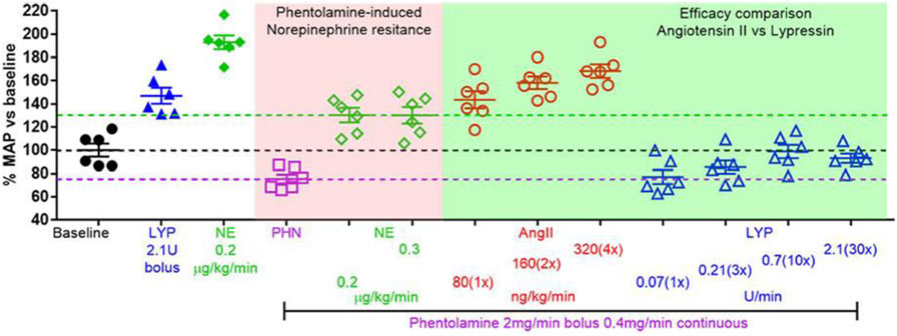
Average MAP of the last minute during each treatment

### P099 Use of a low-dose heparin anticoagulation protocol for Impella CP® devices in cardiogenic shock

#### A Newsome, AT Taylor, SG Garner

##### The University of Georgia College of Pharmacy, Pharmacy, North Augusta, United States

**Introduction:** Impella CP® is a percutaneous left ventricular assist device (LVAD) that requires a heparin-dextrose purge solution to decrease risk of device thrombosis. The manufacturer recommends heparin 50 units per mL, but supra-therapeutic anticoagulation has been observed with this concentration. The purpose of this evaluation was to observe the efficacy and safety of a low-dose heparin-based purge solution (25 units per mL in dextrose 20%) as part of an Impella CP® protocol.

**Methods:** A single site retrospective review was conducted for all adults with the Impella CP® from January 2015 to December 2017.The primary outcome evaluated percentage of activated clotting times (ACT) below therapeutic range, and safety was evaluated by bleeding events, as defined by the Bleeding Academic Research Consortium (BARC) bleeding criteria. Secondary objectives included evaluating the incidence of device thrombosis and rate of heparin-induced thrombocytopenia (HIT). Platelet trends following Impella CP® implantation were characterized.

**Results:** A total of 18 patients were included. The percentage of activated clotting times (ACT) readings within goal of 160 to 200 seconds was 49%, and 38% of readings were sub-therapeutic. No device thrombosis was observed. Per BARC bleeding criteria, 22% (n=4) patients experienced class 3a bleeding and 39% (n=7) experienced class 2 bleeding. Though four (22%) of patients were tested for HIT, no patients were positive. Patients showed universal reductions in platelet counts.

**Conclusions:** The use of a lower dose heparin concentration purge solution was not associated with increased device thrombosis although bleeding still occurred. The use of a low-dose anticoagulation protocol of heparin 25 units per mL in dextrose 20% with supplemental systemic anticoagulation as needed may be a safe and effective alternative and warrants further evaluation.

### P100 Use of resuscitative endovascular balloon occlusion of the aorta for massive gastrointestinal bleeding in ICU

#### Y Kato, A Kuriyama, M Onodera

##### Kurashiki Central Hospital, Department of Emergency Medicine, Kurashiki Okayama, Japan

**Introduction:** Resuscitative Endovascular Balloon Occlusion of the Aorta (REBOA) has been increasingly used for the management of both traumatic and non-traumatic hemorrhagic shock. However, there is limited evidence for its use in gastrointestinal bleeding (GIB), especially in the ICU setting. We successfully treated a patient with massive GIB using REBOA in the ICU. We will discuss the difficulty performing the procedure and its countermeasure.

**Methods:** A case report.

**Results:** An 83-year-old woman was transferred to our hospital with shock. Coffee grounds material was found in a nasogastric aspirate after intubation and upper gastrointestinal endoscopy identified a pulsating large duodenum ulcer without active bleeding, for which an elective procedure was planned. She was admitted to our ICU, responded to initial resuscitation, and thereafter extubated. Her systolic blood pressure (SBP) suddenly dropped to 40mmHg with massive hematochezia at that night, and did not increase despite resuscitation with blood products, crystalloid and norepinephrine. To buy time until measures for stop bleeding, we planned to place REBOA in the ICU. Following the placement of a sheath in the left femoral artery, we tried to place a 7 Fr intra-aortic balloon occlusion catheter, which unintentionally and repeatedly went into the right common iliac artery because her left femoral artery was tortuous. After compressing the right lower abdomen, we managed to introduce REBOA in Zone 1. It took approximately 60 minutes to successfully place the catheter. The patient’s SBP increased immediately after the balloon inflation and bleeding was endoscopically controlled. Inflation time was 36 minutes and no complications were observed.

**Conclusions:** Although REBOA could be a useful procedure for massive GIB, it could be difficult to place the catheter without fluoroscopy. Caution should be exercised with placement when REBOA is performed as a bedside technique in ICU.

**Consent:** Informed consent to publish has been obtained from the patient

### P101 Modulation of endothelial glycocalyx and microcirculation during anaerobic exercise by supplementation with pomegranate extract

#### Z Pranskuniene^1^, E Belousoviene^2^, N Baranauskiene^3^, N Eimantas^3^, M Brazaitis^3^, A Pranskunas^2^

##### ^1^Lithuanian university of health sciences, Department of Drug Technology and Social Pharmacy, Kaunas, Lithuaniadevices in cardiogenic; ^2^Lithuanian university of health sciences, Department of Intensive Care Medicine, Kaunas, Lithuania; ^3^Lithuanian Sports University, Sports Science and Innovation Institute, Kaunas, Lithuania

**Introduction:** The natural components of the pomegranate fruit may provide additional benefits for endothelial function and microcirculation. We hypothesized that chronic supplementation with pomegranate extract might improve glycocalyx properties and microcirculation during anaerobic condition.

**Methods:** Eighteen healthy and physically active male volunteers aged 22–28 years were recruited randomly to the pomegranate and control groups (9 in each group). The pomegranate group was supplemented with pomegranate extract for two weeks. At the beginning and end of the experiment, the participants completed a high intensity sprint interval cycling-exercise (anaerobic exercise) protocol. The systemic hemodynamics, microcirculation flow and density parameters, glycocalyx markers, and lactate and glucose levels were evaluated before and after the two exercise bouts.

**Results:** No significant differences in the microcirculation or glycocalyx were found over the course of the study. The lactate levels were significantly higher in both groups after the first and repeated exercise bouts, and were significantly higher in the pomegranate group relative to the control group after the repeated bout: 13.2 (11.9–14.8) vs. 10.3 (9.3–12.7) mmol/L, p = 0.017.

**Conclusions:** Chronic supplementation with pomegranate extract has no impact on changes to the microcirculation and glycocalyx during anaerobic exercise, although an unexplained increase in blood lactate concentration was observed.

### P102 Identification of cells associated with VA-ECLS mortality by single cell expression profiling

#### E Kort, M Weiland, E Eugster, E Grins, M Leacche, S Fitch, T Boeve, G Marco, M Dickinson, P Wilton, S Jovinge

##### Fredrik Meijer Heart And Vasc Inst, Spectrum Health/Van Andel Inst, DeVos Cardiovascular Research Program, Grand Rapids, United States

**Introduction:** Extracorporeal membrane oxygenation in adults in accompanied by high mortality. Our ability to predict who will benefit from ECMO based on currently available clinical and laboratory measures is limited. The advent of single cell sequencing approaches has created the opportunity to identify cell populations and pathophysiological pathways that are associated with mortality without bias from a priori cell type classifications. Identification of such cell populations would provide both an important prognostic markers and key insight into immune response mechanisms and therefore a possibility for advanced drug matching that may impact clinical response to ECMO in these patients.

**Methods:** Whole genome transcriptomic profiles were generated from a total of 40,935 peripheral blood monocytes obtained from 37 patients at the time of cannulation for ECMO (Fig 1). Differential gene expression analysis was performed with the Monocle package for the R statistical analysis framework. Time-to-event data were analyzed in a survival analysis with a log-rank test for differences.

**Results:** Genes encoding several members of the heat shock family of proteins were up-regulated in cells from non-survivors. Notably, these genes were expressed by a small fraction of cells (2.4% on average). Nevertheless, the proportion of cells expressing these genes was a significant predictor of survival to 30 days (p = 0.02 by log rank test), with a particularly pronounced effect in the first 5 days after initiation of ECMO support (Fig 2).

**Conclusions:** The proportion of cells expressing genes encoding members of the heat shock proteins is predictive of survival on ECMO.

**Fig. 1 (abstract P102).**
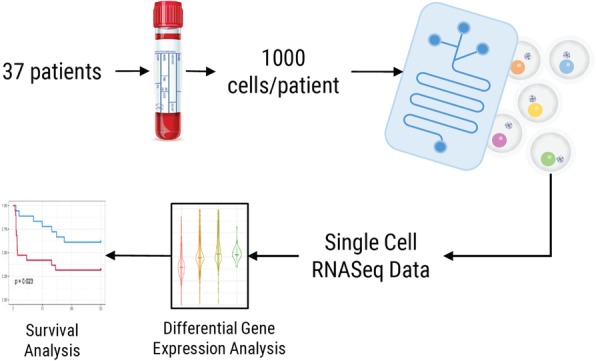
Project methodology - single cell whole transcriptome profiling

**Fig. 2 (abstract P102).**
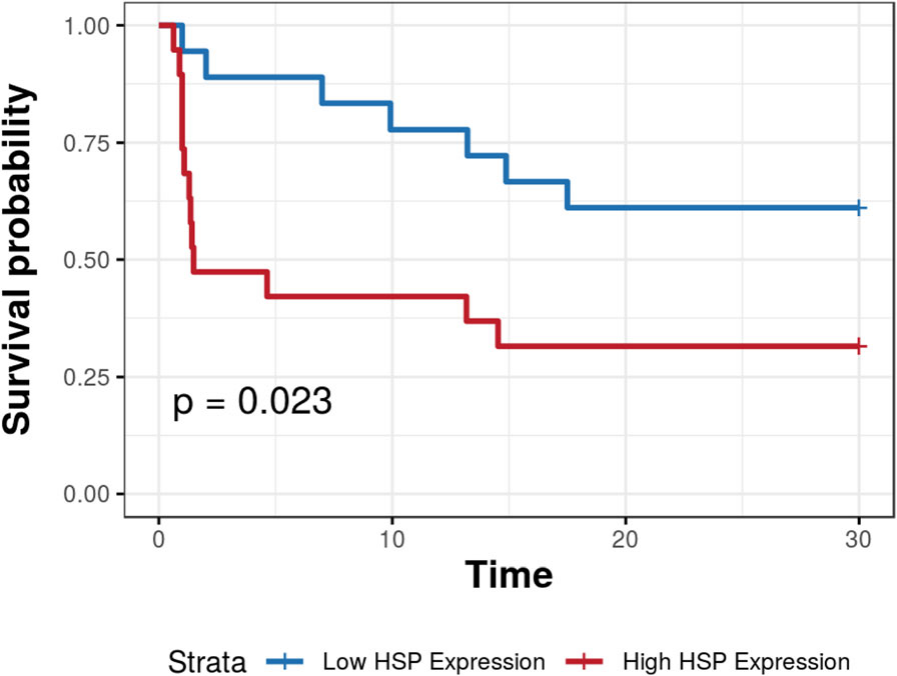
Kaplan Meier curves for patients on VA-ECLS stratified by proportion of HSP positive cells at time of cannulation as measured by single cell RNASeq

### P103 Clinical features and management of patients with high-risk pulmonary embolism in a Moroccan hospital: analysis of 6 years of data

#### A El Khaoudi, R Benmalek, S Ballali, E Bennouna, L Azzouzi, R Habbal

##### CHU iBN Rochd, Intensive care, Cardiology department, Casablanca, Morocco

**Introduction:** High-risk pulmonary embolism is a life-threatening disorder associated with fatal outcomes. The estimated mortality of PE complicated with cardiogenic shock is between 25% and 30%. The diagnosis of PE in hemodynamically unstable patients remains a challenge for both intensivists and cardiologists since the clinical presentation is variable and non specific. The aim of this study is to assess the clinical features, electrocardiographic (EKG) and echocardiographic findings associated with high risk PE, and to describe the management and prognostic of this disease.

**Methods:** a single-center, cross-sectional descriptive study including all patients admitted for PE confirmed by transthoracic echocardiography or Computed tomography angiography complicated with cardiogenic shock in the cardiology intensive care unit in the Casablanca university hospital, from September 2011 to July 2017. During the 6 years study period, 69 patients were included.

**Results:** Mean age was 55.5 years (+/- 8.3), females represented 71.3%. All the patients were hemodynamycally unstable at admission, presenting with dyspnea stage III (41.7%) to IV (52.6%), and/or acute chest pain (23.2%). Tachypnea, hypoxia, and signs of deep vein thrombosis were respectively present in 59, 41, and 24 %. EKG abnormalities were represented by sinus tachycardia in 62.5%, S1Q3 pattern in 27%, RBBB in 19,6% and atrial fibrillation in 17.4%. Echocardiographic aspect of acute cor pulmonale was found in all the patients, right atrial thrombus in 15 patients (21.7%), CT angiography was performed in 39 patients after stabilization. Thrombolytic therapy was performed in 46 patients (66.6%). The mortality rate of our serie was 41.8%.

**Conclusions:** High-risk PE is a severe disorder associated with high mortality and morbidity, thus a multidisciplinary coordination between cardiologists, radiologists and intensivists is crucial for the management of patients with high-risk PE.

### P104 Thrombolytic therapy for submassive pulmonary embolism – single centre experience

#### N Maric, M Mackovic, N Udiljak

##### Clinical hospital Sveti Duh, Intensive Care, Zagreb, Croatia

**Introduction:** Pulmonary embolism (PE) is one of the major causes of mortality, morbidity and hospitalization in Europe. Haemodynamic benefits of thrombolysis in high risk PE are undeniable, yet use of thrombolytic agents in intermediate risk category is controversial due to lack of evidence and fear of complications.

**Methods:** Single-centre retrospective study of 80 PE patients (pt) treated with alteplase (rtPA) in a medical ICU from January 2015 to November 2018 was performed. General data, risk category, PESI and Qanadli score, TnI, ECG and ABG were obtained. Primary outcome was the complication rate, especially bleeding and secondary the survival rate.

**Results:** PE comprised 8.7% (214/2457) of all admissions. 80 of them (37.4%) received rtPA. F:M ratio was 51:29, mean age of 68.9y (26-90). 40% of pt were high risk, 54% intermediate (45% high, 9% low) and 6% were in low risk category. Mean PESI was 139.3 suggesting overall high mortality risk. Mean Qanadli score was 21.97 representing high clot burden. Fibrinolytic therapy was applied during CPR in 11 pt with survival rate of 45%. 83% of pt had elevated TnI levels, and 62.5% had newly diagnosed DVT. Majority of pt (43%) had no known predisposing conditions, followed by immobility (21%) and cancer (15%). In ECG analysis tachycardia and V1-V3 T wave inversion were the most common findings whereas hypoxemia± hypocapnia were the most prominent features in ABG analysis. 8 pt (10%) had bleeding complications (none intracranial), 3 (3.7%) during rtPA, 5 (6.3%) in the first 36h and only 3 pt required transfusion. Mortality rate was 12%: 6% directly due to PE (all during CPR) and 6% due to late complications (newly diagnosed cancer and infections).

**Conclusions:** In our experience, fibrinolytic therapy is safe and effective but in submassive PE should be applied after thorough assessment of risks and benefits on individual basis aiming to patient tailored precision medicine.

### P105 Our experience of emergent cases: the surgical treatment of thrombo-embolus in transit

#### M Obeid^1^, A Abdurakhmanov^1^, O Mashrapov^1^, S Muminov^2^, N Rakhimov^1^, I Abdukhalimo^1^

##### ^1^Health Ministry Of The Republic of Uzbekistan Republic Research Center Of Emergency Medicine Of Uzbekistan, Cardiosurgery, Tashkent, Uzbekistan; ^2^Health Ministry Of The Republic Of Uzbekistan Republic Research Center Of Emergency Medicine Of Uzbekistan, Emergency Vascular Surgery, Tashkent, Uzbekistan

**Introduction:** Blood clots of the main veins of the lower extremities are the main cause of pulmonary thromboembolism (PE). Pulmonary thromboembolism is recorded in 2-15% of autopsies. Floating blood clots in the path from the legs to the pulmonary arteries are a severe form of venous thromboembolism with high early mortality.

Aim of the study: Our surgical experience and prophylaxis of transient pulmonary thromboembolism are demonstrated in the study.

**Methods:** During the period from 2015 to 2018, 6 patients with pulmonary thromboembolism have been on surgery at the RSCEMP. All patients were female. The average age of patients was 56.2 ± 3.4 years old. All patients came to us at an early stage with deep vein thrombosis, mainly after the removal of uterine fibroids and inadequate hormone therapy. In all patients, hypoechoic loose thromb were observed during the examination of the lower limb CDS, and the floating atrial right thrombus was visualized on the echocardiography. All operations were performed under the conditions of artificial blood circulation with sternotomy access. After connecting the CPB pump, as a rule, the surgery is ended with a thromboembolectomy from the right atrium. On the next stage, inferior vena cava clipping was performed through the retroperitoneal access (using the original clip).

**Results:** Mortality rate was 0% during the hospital stay. In the long-term period of 18 months, mortality was also not noted. The inferior vena cava syndrome was developed in 2 patients. In 4 patients, postthrombotic syndrome was developed during long-term period.

**Conclusions:** Thrombo-embolectomy from the right atrium and inferior vena cava clipping prevents massive pulmonary embolism and prolongs the life of the patients. However it can aggravate the development and course of chronic venous insufficiency.

### P106 New onset versus pre-existing atrial fibrillation in critically illness: Do patient populations differ?

#### B Johnston, N Miller, A Hampden-Martin, I Welters

##### Royal Liverpool University Hospital, Intensive Care Unit, Liverpool, United Kingdom

**Introduction:** Atrial fibrillation (AF) is the commonest arrhythmia in critical illness and is associated with mortality. Systemic inflammation and infection have been identified as triggers for fast heart rate and development of new-onset AF (NOAF) during critical illness. It is unknown, however, if patients with pre-existing AF (PEAF) who develop a fast heart rate differ from patients with NOAF with regard to organ function, biometric characteristics and disease severity.

**Methods:** This study was performed at a large inner-city University Hospital between January 1st and December 31st 2017. Patients who had fast AF during their critical illness were prospectively identified by the research team. Fast AF was defined as patients with AF and a heart rate above 110/minute. Biometric data, routine blood results, previous medical history, treatment of AF and duration of fast AF were collected. Patients with pre-existing AF (PEAF) were compared with those who had no history of arrhythmias documented.

**Results:** Atrial fibrillation was new-onset in 49 patients (50.5%), and pre-existing in the remaining 48 (49.5%). The APACHE II score on admission was higher for NOAF patients compared to PEAF patients (17.0 (13.5 – 21.5) vs 14.0 (11.0 - 17.0), p=0.005). In addition, a greater number of NOAF patients were admitted with sepsis, though these differences were not significant. Patient with NOAF were significantly younger than those with PEAF (67.0 (59.0 - 77.0 years vs 74.5 (66.5 - 79.0 years), p=0.008). Chronic cardiovascular comorbidities were more frequent in patients with PEAF. Kidney function was worse in patients with NOAF, platelet counts was lower and prothrombin times higher.

**Conclusions:** As expected, patients with PEAF suffered more often from cardiovascular comorbidities and were older. In contrast NOAF patients were sicker and had worse clotting profiles and kidney function. Our results indicate that patients with PEAF differ with regard to organ function and co-morbidities. Consequences for treatment remain unclear at this stage.

### P107 Dobutamine after cardiac surgery: a randomised controlled clinical trial

#### R Franco^1^, JL Vincent^2^, J Almeida^3^, J Fukushima^1^, G Oliveira^1^, S Rizk^1^, C Park^1^, M Mourão^1^, G Landoni^4^, L Hajjar^1^

##### ^1^Heart Institute, Cardiology, Sao Paulo, Brazil; ^2^Dept of Intensive Care, Erasme Hospital (Université libre de Bruxelles), Brussels, Belgium; ^3^Heart Institute, Surgical ICU, Sao Paulo, Brazil; ^4^IRCCS San Raffaele Scientific Institute, Milan, Italy

**Introduction:** The purpose of the study was to evaluate whether a restrictive strategy regarding dobutamine use was non-inferior to a liberal strategy in patients undergoing cardiac surgery.

**Methods:** Clinical trial, randomised, unicentric, controlled, parallel-group, non-inferiority trial. Patients were randomly assigned preoperatively to two distinct dobutamine strategies: a liberal strategy, in which all patients would receive dobutamine after weaning from CPB; or a restrictive strategy, in which the use of dobutamine after CPB weaning would be guided by hemodynamic evidence of low cardiac output. The primary outcome was composite endpoint of arrhythmias (ventricular or supraventricular tachyarrhythmias), acute myocardial infarction, stroke, and death from all causes within 30 days after cardiac surgery.

**Results:** A total of 160 patients were included in the final analysis; 80 assigned to the restrictive strategy and 80 to the liberal strategy. The use of dobutamine was lower in the restrictive group (67.1 vs. 100%, P <0.001). The primary outcome occurred in 31.3% of the restrictive group and 33.8% of the liberal group (P = 0.736). There were no significant differences between the restrictive and liberal strategies regarding the incidence of supraventricular or ventricular tachyarrhythmias (23.8% vs. 27.5%, P = 0.587), cardiogenic shock (11.3% vs. 13.8%, P = 0.633), low cardiac output syndrome (13.8% vs. 15%, P = 0.822), acute myocardial infarction (1.3% vs. 2.5%, P = 1,000), stroke, 5% vs. 0%, P = 0.497) and death from all causes (2.5% vs. 6.3%, P = .443).

**Conclusions:** The use of a restrictive strategy regarding the use of dobutamine, based on a clinical scenario of reduction of the cardiac index associated with signs of tissue hypoperfusion, is non-inferior to a liberal strategy in patients undergoing cardiac surgery.

### P108 Preconditioning with levosimendan is cost-effective

#### A Alvarez^1^, J Ferrer^2^, J Jiménez-Rivera^1^, J Iribarren^1^, J Montoto^3^, J Lacalzada-Almeida^4^, P Jorge^4^, MJ García-González^5^, R Martinez-Sanz^3^, M Mora^1^

##### ^1^Hospital Universitario de Canarias, Intensive Care, La Laguna, Spain; ^2^Hospital Universitario de Canarias, Health Economist. University La Laguna, La Laguna, Spain; ^3^Hospital Universitario de Canarias, Cardiovascular Surgery, La Laguna, Spain; ^4^Hospital Universitario de Canarias, Cardiology Department, La Laguna, Spain; ^5^Hospital Universitario de Canarias, La Laguna, Spain

**Introduction:** Coronary Artery Bypass Grafting (CABG) plays an important role in patients with left ventricular dysfunction and major vessels disease, in this context it becomes necessary to design cost-effective strategies like preconditioning with levosimendan.

**Methods:** Two strategies are compared in terms of cost-effectiveness: Preconditioning with levosimendan (n=15), compared to standard care (n=117). Both strategies were used in elective CABG with preoperative Left Ventricular Ejection Fraction less than 40%. The adverse effects studied included: postoperative de novo atrial fibrillation, low cardiac output (LCO), renal failure and prolonged mechanical ventilation. The costs of adverse effects were evaluated. Deterministic and probabilistic sensitivity analysis was performed, and Monte Carlo simulations.

**Results:** Average cost on levosimendan group was €14,601.72 while the average cost per patient without levosimendan was €18,401.71. Mean ICU stay per patient was estimated at 2.47 and 5.18 days with and without levosimendan use respectively. Patients with no complications represented 46.7% of the total in the levosimendan arm, as compared to 27.4% in the non-levosimendan arm. Figure 1 shows, in the cost-effectiveness plane, the cost and effectiveness pairs obtained as a result of each Monte Carlo simulation. In all simulations, use of levosimendan was less expensive and more effective. The variable incidence of LCO does have an impact on the potential complications resulting from it. Cost per patient in the base case was €14,601.73 ranging from €13,264.27 and €14,103.12 with administration of levosimendan, as compared to €18,401.71 for the base case, ranging from €16,899.89 and €17612.64. Low Cardiac Output, represents 6%-18% in the intervention arm vs 25%-35% in the control arm.

**Conclusions:** Preconditioning with levosimendan, is a cost-effective strategy preventing postoperative LCO in high-risk patients undergoing elective CABG.

**Fig. 1 (abstract P108).**
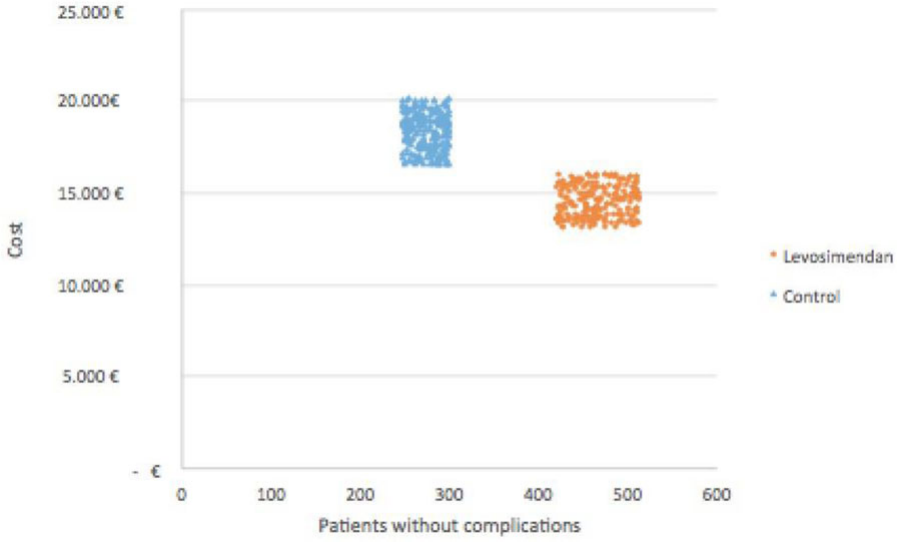
Cost-effectiveness plane

### P109 Analysis of the LEVO-CTS and LICORN trials on pre-operative levosimendan treatment in isolated CABG surgery

#### M Heringlake^1^, G Chatellier^2^, T Caruba^2^, P Pollesello^3^, M Kivikko^4^, T Sarapohja^5^, W Toller^6^

##### ^1^Universitätsklinikum Schleswig-Holstein, Klinik für Anästhesiologie und Intensivmedizin, Lübeck, Germany; ^2^Université Paris Descartes, Sorbonne Paris Cité, Paris, France; ^3^Orion Pharma, Hospital Care, Espoo, Finland; ^4^Orion Pharma, Global Medical Affairs, Espoo, Finland; ^5^Orion Pharma, Statistics, R&D, Espoo, Finland; ^6^Medical University, Universitätsklinik für Anästhesiologie und Intensivmedizin, Graz, Austria

**Introduction:** Both the LEVO-CTS[1] and LICORN[2] trials evaluated the role of levosimendan in preventing low cardiac output syndrome in patients undergoing cardiac surgery. The studies were similar in their design and recruited patients with preoperatively low LVEF undergoing either isolated CABG or valve surgery combined with CABG (Table 1). In both, a 24-hour levosimendan infusion was started at induction of anesthesia. Neither study met the primary efficacy composite enpoints, but both showed a clear tendency for better outcome in patients undergoing a CABG compared to a valve procedure. We are currently evaluating the solidity of a co-analysis based on shared end-points.

**Methods:** We are planning a shared analysed of the data related to the CABG settings and analyze the aggregated mortality data for both studies at 1 and 3 months by Cochran-Mantel-Haenszel odds ratio. Data from individual studies would be analysed as fixed effect and Breslow-Day test was used to evaluate homogeneity of the odds ratios

**Results:** In the placebo groups of the two studies, the mortality is similar; 7.9% (22/279) in LEVO-CTS and 7.3% (9/123) in LICORN, corroborating the working hypothesis that the two studies can be co-analysed. In a preliminary combined analysis (Fig 1), 90-day mortality was 7.7% (31/402) in the placebo group and 2.9% (12/407) in the levosimendan group. Odds ratio was significantly in favor of levosimendan (0.36; 95% confidence interval 0.18-0.72; p=0.0026, Fig. 1)

**Conclusions:** The LEVO-CTS and LICORN trials can be co-analysed in their sub-setting of patients requiring isolated CABG surgery for mortality at 1 and 3 months. A preliminary analysis on mortality reinforce the hypothesis that, in isolated CABG surgery, levosimendan lowers post-operative mortality significantly both at 1 and 3 months, when started at the induction of anesthesia

**References**

[1] Mehta RH et al. N Engl J Med. 2017;376(21):2032-2042.

[2] Cholley B et al. JAMA. 2017;318(6):548-556

**Table 1 (abstract P109).** The designs of LEVO-CTS and LICORN studies

	LEVO-CTS, N=849	LICORN, N=335
Isolated CABG surgery	66.3% (563/849)	73.4% (246/335)
Combined CABG & valve(s) OR valve(s) only surgery	33.7% (286/849)	26.6% (89/335)
Preoperative LVEF limit for enrollment	≤35%	≤40%
Dose of levosimendan	0.2 micg/kg/min for 1 h, followed by 0.1 for 23 h	0.1 mic/kg/min for 24 h
Longest follow-up	90 days	180 days

**Fig. 1 (abstract P109).**
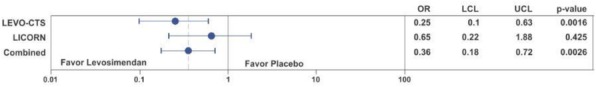
LEVO-CTS and LICORN CABG all-cause mortality at 3 months (combined n=809)

### P110 Relevance of mobile intensive care unit during pre-hospital phase treatment in ST elevation myocardial infarction

#### R Viejo Moreno^1^, A Cabrejas Aparicio^2^, M Gálvez Marco^3^, N Arriero Fernández^4^, Z Eguileor Marín^4^, J Balaguer Recena^5^, C Carriedo Scher^6^, C Marian Crespo^4^

##### ^1^GUETS - SESCAM, MICU Guadalajara, Guadalajara, Spain; ^2^MICU Azuqueca de Henares; GUETS - SESCAM, Guadalajara, Spain; ^3^MICU-Guadalajara; GUETS - SESCAM, Guadalajara, Spain; ^4^Intensive Care Unit - H.U Guadajara, Guadalajara, Spain; ^5^Cardiology department - H.U. Guadajara, Guadalajara, Spain; ^6^GUETS - SESCAM, Guadalajara, Spain

**Introduction:** Emergency medical system (EMS) -based ST elevation myocardial infarction (STEMI) networks allows not only STEMI diagnosis in the pre-hospital phase but also reduces treatment delays; treat your fatal complications and the immediate activation of the catheterization laboratory. The aim of study was to investigate the effect of out-of-Hospital by Mobile Intensive Care (MICU) versus Hospital beginning treatment in hospitalization length and survival of patients with STEMI diagnosis

**Methods:** Observational retrospective study on STEMI patients managed by prehospital MICU and the Emergency Department (ED) of Hospital Guadalajara (Spain) from January 2015-September 2018. Items compared were demographic variables, cardiovascular risk factors, treatment, time to first medical contact to percutaneous coronary intervention (PCI), location of coronary lesions, GRACE score, length in ICU and hospital stay and survival to discharge and to 30 days.

**Results:** 360 were evaluated by STEMI, 219 (60.8%) by MICU. 280 (77,8%) male, mean age was 61.0 (RIC: 53.0-71.0) years old (Fig.1). There were differences in P2Y12 inhibitor administration. Ticagrelor was more frequent in patients admitted directly in hospital 94 (66.7%) Vs 82 (37.4%) (p=0.00) instead of Prasugrel by MICU physicians 13 (9.2%) Vs 112 (51.2%) (p=0.00). Morphine was more administered in MICU 49 (34.8%) VS 121 (55.3%) patients (p=0.00). Initial attention of patients with STEMI by MICU reduced time to PCI, stay length in ICU: 76.8 (37.4-191.0) Vs 44.8 (36.6-53.1) hours (p=0.02), and was associates lower mortality at hospital discharge 13 (9.2%) Vs 8 (3.7%) (p=0.02) and to 30 days compared to those arriving directly the hospital by themselves 15 (10.6%) VS 10 (4.6%) (p=0.02).

**Conclusions:** Around 40% STEMI patients still come to hospital by their own. A STEMI out-and-in of Hospital network and MICU based EMS with presence of physician allows to reduce the time to PCI and decreases the mortality of patients with SCACEST in Guadalajara.

**Fig. 1 (abstract P110).**
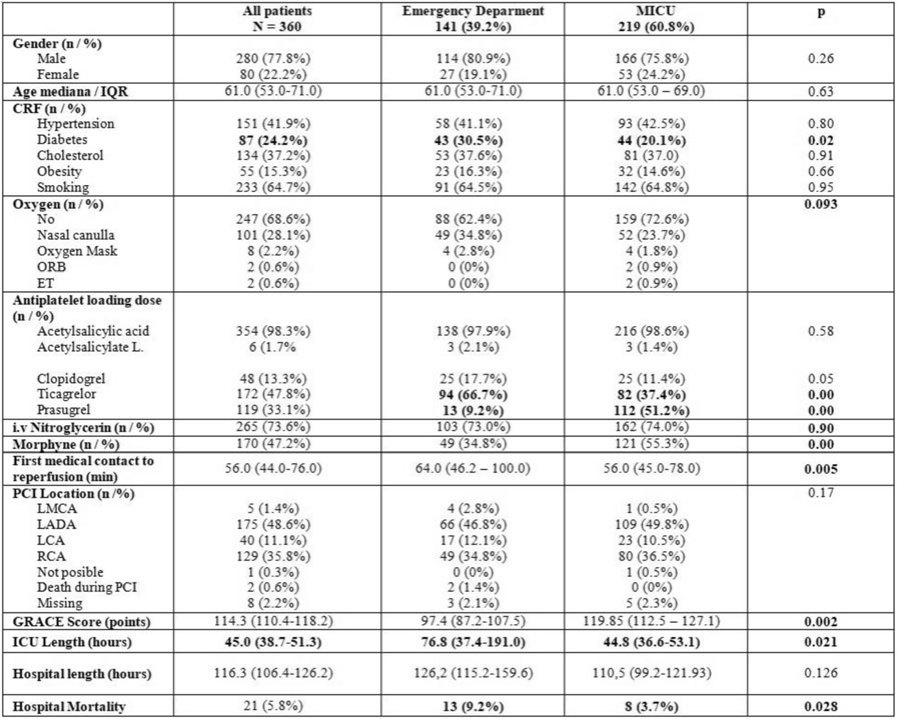
Demographic, clinical and prognosis characteristics of acute coronary syndrome groups. MICU: mobile intensive care unit. CRF: cardiovascular risk factor; ORB: oxygen reservoig bag; ET: endotracheal tube; PCI: percutaneous coronary intervention; LMCA: left main coronary artery; LADA; left anterior descending; LCA: left circumflex artery; RCA: right coronay artery. ICU: intensive care unit.

### P111 Contrast induced nephropathy after primary percutaneous coronary intervention in STEMI patients

#### M Trajkovic^1^, M Kovacevic^1^, A Vulin^1^, M Femic^2^, M Petrovic^1^, S Dimic^1^, M Jarakovic^1^, B Crnomarkovic^1^, S Keca^1^, I Srdanovic^1^

##### ^1^Insitute of Cardiovascular Diseases of Vojvodina, ICCU, Novi Sad, Serbia; ^2^Clinical Center of Vojvodina, ICU, Novi Sad, Serbia

**Introduction:** Contrast induced nephropathy (CIN) is a complex acute renal failure syndrome, which can occur after primary percutaneous coronary intervention (PCI) and is an important cause of morbidity and mortality in this subgroup of patients. The aim of our study was to establish the incidence and predictors of CIN after primary PCI.

**Methods:** We performed a retrospective analysis of STEMI patients treated with primary PCI in the period from January until September of 2017. CIN was defined as an absolute increase in baseline serum creatinine of ≥0.5 mg/dL (44 μmol/l) or >25% relative rise within 72 hours after primary PCI. We analyzed demographic characteristics, risk factors, clinical status at hospital admission, laboratory parameters, left ventricle ejection fraction and data regarding PCI procedure.

**Results:** The study included 729 patients, with an average age of 62.33 ± 11.78 years, 66.1% of the patients were males. An average of 175.67 ± 91.77ml of contrast medium per patient was utilized. CIN developed in 11(1.5%) patients and overall intra-hospital mortality was 8.4 %. In multivariate analysis, the independent predictors of CIN were age>75 years (OR 6.434; 95% CI (1.174 - 35.256); p=0.032), diabetes mellitus (OR 21.74; 95% CI (2.672 - 176.891); p=0.004), Killip class III (OR 17.394;95% CI (2.309 - 146.539); p=0.006), creatinine at admission (OR 1.011;95% CI (1.004-1.018);p=0.03), and hemoglobin level at admission (OR 0.960;95%CI (0.925 - 0.996); p=0.029). Patients with CIN had significantly longer length of hospitalization (10.63 ± 7.86 vs. 5.79 ± 5.89 days; p=0,007) and higher intra-hospital mortality (27.3% vs. 8,1%; p=0.023). CIN was independent predictor of mortality (OR 12.485; 95% CI (1.472-105.909); p=0.021).

**Conclusions:** In our study group of STEMI patients treating with primary PCI, the independent predictors of CIN were age >75 years, diabetes mellitus and higher Killip class at admission. CIN was the independent predictor of mortality.

### P112 Early outcome of left main stem disease coronary bypass surgery in intensive care unit

#### A Omar, S Hanoura, P Sivadasan, S Sudarsanan, H Osman, Y Shouman, A AlKhulaifi

##### Hamad Medical Corporation, Heart Hospital-Cardiothoracic surgery, Doha, Qatar

**Introduction:** Left main coronary artery (LMCA) disease is a disease of the main coronary branch that gives more than 80% of blood supply to the left ventricle, it carries high mortality without surgical intervention; [1] however the influence of LMCA surgery on morbidity ICU measures needs to be explored. We aim to determine whether LMCA is definitive risk factor for prolonged ICU stay as a primary outcome and whether LMCA is definitive risk factor for early morbidity

**Methods:** Retrospective descriptive study with purposive sampling analyzing 398 patients underwent isolated coronary artery bypass surgeries (CABG). Patients were divided into 2 groups those with LMCA disease as group 1 (75 patients) and those with coronary arty disease requiring surgery but without LMCA disease as group 2 (324 patients) then we will correlate with ICU outcome parameters including ICU stay length, post-operative atrial fibrillation, acute kidney injury, re-exploration, perioperative myocardial infarction, post operative bleeding and early mortality.

**Results:** Patients with LMS had significantly higher diabetes prevalence (43.3% vs 29%, p=0.001). However, we did not find a statistical significant difference regarding ICU stay, or other morbidity and mortality outcome measures

**Conclusions:** Diabetes was more prevalent in patients with LMS. The latter group showed similar outcome as those without LMS in this study these findings may help in guiding decision making for future practice and stratifying the patients care.

**Reference**

1) Göl MK, et al. Journal of cardiac surgery. 2000;15:217-22.

### P113 Impact of comorbidities and patient age on clinical outcomes in patients with myocardial infarction - a nationwide cohort study

#### C Bachli^1^, U Wagner^2^, B Müller^1^, P Schütz^1^, A Kutz^1^

##### ^1^Kantonsspital Aarau, Aarau, Switzerland; ^2^Swiss Federal Office for Statistics, Neuchâtel, Switzerland

**Introduction:** Multimorbidity in patients admitted for acute myocardial infarction [AMI] is associated with higher risk for in-hospital mortality and adverse clinical outcomes. We investigated to what extent an increasing number of comorbidities affects the age-stratified excess risk of death and other clinical outcomes among patients with myocardial infarction.

**Methods:** We analyzed nationwide administrative data of 174`803 admissions for an acute myocardial infarction between 2006 and 2016. We calculated multivariate regression models to study the association of four comorbidities (chronic kidney disease [CKD], diabetes mellitus, heart failure [HF], and atrial fibrillation) and excess risk of in-hospital mortality, length of hospital stay [LOS], and 30-day readmission and stratified the analysis for different age categories.

**Results:** The incidence of admissions for AMI increased continuously during the observed decade without an increase in in-hospital mortality, LOS, and 30-day readmission. Among admitted patients with AMI, there was a stepwise increase in risk for adverse outcomes for each comorbidity. Compared to patients with no comorbidity, patients with 4 comorbidities had 6-fold increased risk for mortality (adjusted odds ratio [OR] 6.6, 95% confidence interval [CI] 5.6 to 7.7) and a similar risk for readmission (OR 1.0, CI 0.9 to 1.2). The LOS was 5.3 days (CI 5.1 to 5.5) in patients with no comorbidity and increased by 2.5 days (CI 2.4 to 2.5) with each additional comorbidity. These associations were stronger in younger compared to older patients. CKD was the strongest predictor of in-hospital mortality and LOS, while HF was the strongest predictor of 30-day readmission.

**Conclusions:** This study of nationwide admitted patients with AMI found a stepwise increase in the risk for adverse outcome with increasing number of comorbidities, particularly in the younger patient population. Younger, multimorbid patients may thus have the largest benefits from multidisciplinary treatments.

### P114 Preferable quality of life on average 2 years after emergency ECLS therapy

#### C Lang^1^, F Schroth^1^, V Zotzmann^1^, T Wengenmayer^1^, B Schmid^2^, C Bode^1^, D Staudacher^1^

##### ^1^University Heart Center Freiburg, Cardiology, Freiburg, Germany; ^2^University Emergency Center, Freiburg, Germany

**Introduction:** Certified cardiac arrest centers, sophisticated post cardiac arrest care and prehospital ECLS teams aim to increase survivor rates with a preferable neurological outcome after cardiac arrest. Centers also provide emergency ECLS and ECLS pick ups for cardiogenic shock patients before arresting. Few data answer the question of the long-term quality of life after ECLS therapy.

**Methods:** In a retrospective single center register we included patients after emergency ECLS (eCPR and cardiogenic shock) between 10/2010 and 10/2017 discharged alive and performed a follow-up after 2 years on average at 6/2018. In our center criteria to initiate ECLS therapy in cardiogenic shock or under cardiac arrest are an observed collaps, shockable rhythm, absence of frailty and severe comorbidities. All patients were requested to take part in a telephone interview. Thus, we analyzed survival, CPC scores and SF36 scores.

**Results:** 97 patients with hospital survival after ECLS were screened. 44% (N=43) had survived until 6/2018; 38 patients were not accessible; 16 had ceased. 33 survivors (mean±SD; min-max; 53±17; 19-78 years, 8 women) answered SF36 questionaires 29±15; 7-64 months after ECLS (45% cardiogenic shock, 55% eCPR with shockable rhythm in 89%). The participants´ CPC scores were in median 1. The results of the SF36 were physical functioning 84±21, physical role functioning 84±28, bodily pain 92±17, general health 68±23, vitality 65±26, social role functioning 83±28, emotional role functioning 96±14 and mental health 81±22. Survivors who did not take part at the SF36 had a CPC score of in median 2 (N=10, 5 personally signed refusals, 3 language barriers, 2 vegetative states).

**Conclusions:** After emergency ECLS therapy and hospital survival 44% of our patients survived the following 2 years up to over 5 years with a preferable neurological outcome and a general mentally and physically satisfactory quality of life. A vague outcome in 39% limits the results of our study.

### P115 Cutaneous blood flow as a predictor of successful weaning from VA-ECMO

#### W Mongkolpun, P Bakos, L Peluso, F Annoni, JL Vincent, J Creteur

##### Erasme Hospital, Université libre de Bruxelles, Intensive Care Department, Brussels, Belgium

**Introduction:** Successful weaning from VA-ECMO requires the restoration of a sufficient cardiac function to ensure an adequate tissue perfusion. Skin blood flow (SBF) is among the first to deteriorate during circulatory shock and the last to be restored after resuscitation. SBF would be a good predictor of successful weaning from VA-ECMO.

**Methods:** Patients with VA-ECMO, who required a first weaning attempt, were included. Weaning procedure (WP) was performed by a reduction of VA-ECMO blood flow to 1 L/min for 10 minutes. The weaning criterion was an aortic velocity–time integral (VTI) > 10 cm. Successful weaning from VA-ECMO was defined as hemodynamic stabilization and without the need to increase the vasopressor dose during the next 24 hours. SBF, assessed by skin laser Doppler (Periflux5000, Perimed, right index finger); perfusion unit: PU), together with global hemodynamic parameters were obtained before and after 3 min of weaning. Receiver operating characteristic curves (ROC) were generated to assess the ability and reliability of baseline parameters to predict a successful weaning.

**Results:** We studied 22 WPs in patients with VA-ECMO for pulmonary embolism (n = 2), post cardiotomy (n = 3), acute coronary syndrome (n = 14), myocarditis (n = 3). These were successful (SW) in 12 and unsuccessful (NSW) in 10. At baseline, hemodynamic variables, lactate, ECMO blood flow were similar in both groups (Table1). SBF was greater in SW than NSW patients (Table1). During WP, CI rose from baseline and was similar in SW and NSW (p=0.1) (Table 2). VTIs were higher in SW than NSW (13 (12-14) vs 8 (6-8), respectively, p=0.03). SBF decreased in SW and remained low in NSW (Table 2). From the ROC curves analyses, baseline SBF had the highest area under the ROC curve with a cut off ≥ 34 PU (sensitivity 83%, specificity 92%) (Figure1).

**Conclusions:** SBF is a good predictor of successful weaning from VA-ECMO

**Table 1 (abstract P115).** Baseline clinical characteristic including SBF between successful and unsuccessful weaning groups

	Total patients (N=22)	Successful patients (N=12)	Unsuccessful patients (N=10)	p
Mean arterial pressure (MAP)(mmHg)	68 (65-76)	76 (70-77)	68 (65-69)	0.2
Cardiac index (CI) (L/min/m2)	1.6 (1.3-2.3)	1.4 (1.2-2.3)	1.5 (1.2-2.0)	0.2
Norepinephrine dose (mcg/kg/min)	0.21 (0.15-0.60)	0.18 (0.1-0.22)	0.3 (0.17-0.7)	0.2
ScvO2 (%)	74 (65-77)	74 (70-77)	74 (65-75)	0.7
Lactate (mmol/L)	2 (1.5-2.7)	2.1 (1.8-2.5)	2 (1.8-2.5)	0.8
ECMO blood flow (L/min)	2.8 (2.4-3.2)	2.5 (2.3-2.8)	2.8 (2.5-3.2)	0.2
SBF (PU)	22 (11-87)	100 (45-132)	11 (9-22)	<0.01

**Table 2 (abstract P115).** Hemodynamic parameters and SBF during WP between successful and unsuccessful weaning groups (*p<0.05 Before WP vs During WP)

	Successful patients (N=12)	Successful patients (N=12)	Unsuccessful patients (N=10)	Unsuccessful patients (N=10)
	Before WP	During WP	Before WP	During WP
MAP (mmHg)	76 (70-77)	73 (70-81)	68 (65-69)	71 (67-72)
CI (L/min/m2)	1.3 (1.2-2.3)	2.1 (1.8-2.6)*	1.5 (1.2-2.0)	1.9 (1.5-2.6)*
ScvO2 (%)	74 (70-77)	70 (69-72)	74 (65-75)	68 (65-72)
SBF (PU)	100 (45-132)	58 (34-112)*	11 (9-22)	13 (10-21)

**Fig. 1 (abstract P115).**
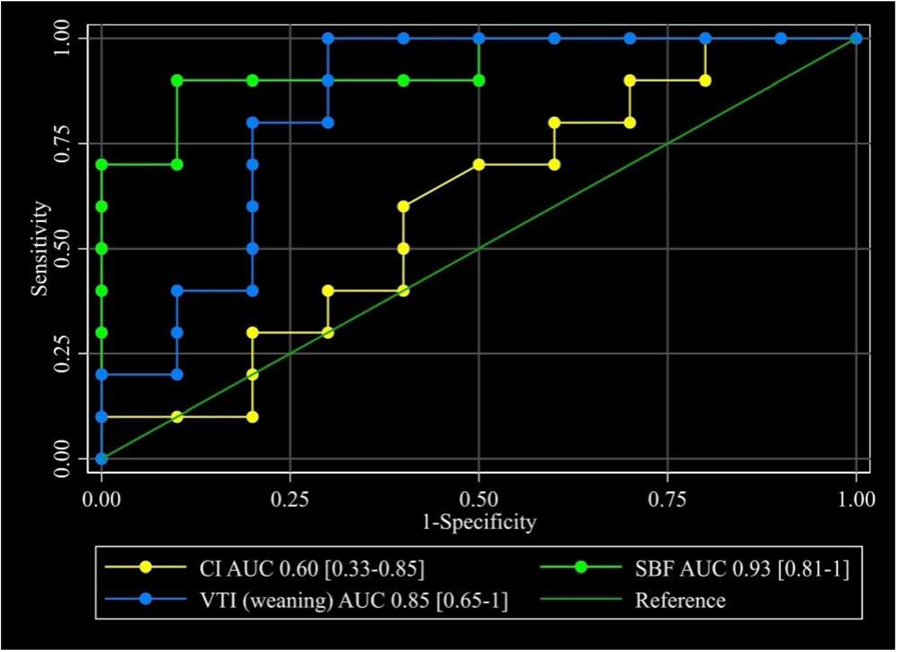
Baseline parameters and VTI during WP to predict successful weaning from VA-ECMO

### P116 Postoperative cognitive dysfunction after endo-CABG: an observational prospective cohort study

#### I Gruyters^1^, J Dubois^2^, J Vandenbrande^2^, A Yilmaz^3^, M Van Tornout^2^, JP Ory^2^, B Stessel^2^

##### ^1^Jessa Hasselt, Anesthesia, Hasselt, Belgium; ^2^Jessa Hasselt, Dept of anesthesia and intensive care, Hasselt, Belgium; ^3^Jessa Hasselt, Dept of cardiac-thoracic surgery, Hasselt, Belgium

**Introduction:** Postoperative cognitive dysfunction (POCD) is defined as a temporarily decline in cognition associated with surgery. Long-term POCD (3 months after surgery) occurs in 10-30% of cardiac patients and is associated with a higher morbidity and mortality. Endo-CABG is a new minimally invasive endoscopic coronary artery bypass grafting (CABG) technique that requires retrograde arterial perfusion which may be associated with a higher incidence of neurological complications. The aim of this study is to assess the incidence of POCD after endo-CABG.

**Methods:** Sixty consecutive patients undergoing an endo-CABG were enrolled. POCD was assessed following the recommendations of the “1995 statement of consensus on assessment of neurobehavioral outcomes after cardiac surgery”. A comparative group of 60 patients undergoing percutaneous coronary intervention (PCI) and a control group of 60 healthy volunteers were also enrolled. Additional tests included the digit span test and digit symbol-coding test. Patients were tested at baseline and at 3-month follow-up. POCD is defined as a Reliable Change Index (RCI) ≤ -1.645 (significance level 5%), or Z-score ≤ -1.645 in at least two different tests.

**Results:** After enrolling 60 patients in each group, respectively 46 in the Endo-CABG-group, 44 in the PCI-group and 48 healthy controls were analysed. Patients suffering from a CVA within three months after their procedure were automatically classified as having POCD (PCI: n= 1; Endo-CABG: n= 1). The total incidence of POCD was not different between groups (PCI: n= 7; Endo-CABG: n= 6, p=0.732).

**Conclusions:** Our results suggest that the risk of POCD after Endo-CABG is low and comparable with the risk of POCD after PCI.

### P117 30-day clinical outcome following endo-CABG: an observational cohort study

#### F Polus^1^, A Yilmaz^2^, P Starinieri^2^, B Robic^2^, J Dubois^1^, B Stessel^1^

##### ^1^Jessa Hospital, Anesthesiology & Intensive Care, Hasselt, Belgium; ^2^Jessa Hospital, Cardiothoracic Surgery, Hasselt, Belgium

**Introduction:** Traditional CABG via median sternotomy is associated with major disadvantages such as a prolonged recovery time, a poor cosmetic result, a significant risk of chronic post-sternotomy pain, sternal instability, delayed bone healing and wound infection. Therefore, a new minimally invasive endoscopic coronary artery bypass grafting (Endo-CABG) technique to treat patients with single- and multi-vessel coronary artery disease has been developed. Endo-CABG combines a thoracoscopic technique via 3 thoracic ports (5 mm) and a mini-thoracotomy utility port (3-4 cm) through the intercostal space. Hence, all walls of the heart can be easily grafted. The aim of this study is to discuss the short-term clinical results of endo-CABG.

**Methods:** From 01/2016 to 01/2018, data from 342 consecutive patients undergoing an Endo-CABG at the Jessa Hospital, Belgium, were prospectively entered into a customized database. This database was merged with data from the Belgian Association for Cardio-Thoracic Surgery (BACTS) and retrospectively reviewed. Subgroup analysis is performed based on the European system for cardiac operative risk evaluation (EuroSCORE). Data are presented as mean (SD) or number (%).

**Results:** Seven patients were excluded from the analyses because of missing data. Up to 85% of the patients presented with multivessel disease necessitating multivessel endo-CABG. A Y-graft anastomosis was performed in 40% of the patients. Respectively 78%, 18% and 2% of the patients had a low risk, a moderate risk and a high risk EuroSCORE. Mortality at 30 days was comparable with the predicted EuroSCORE: 0% in the low risk group, 6,5% in the moderate risk group and 25% in the high risk group. Graft failure was present in 1.2% of all cases. The overall incidence of in hospital revision was 7.3%.

**Conclusions:** Our results suggest that the Endo-CABG technique is a safe and effective procedure to treat single- and multi-vessel coronary artery disease without the need for a specific patient selection.

### P118 Incidence and outcome of rhabdomyolysis after aortic dissection surgery

#### P Sivadasan^1^, AS Omar^2^, C Carr^3^, A Pattath^4^, S Hanoura^4^, S Sudarsanan^4^, H Ragab^4^, A Karmakar^4^

##### ^1^Hamad Medical Corporation, Cardiac Anaesthesia & ICU Division, Department of Cardiothoracic Surgery, Doha, Qatar; ^2^Hamad Medical Corporation, Cardiothoracic intensive Care, Doha, Qatar; ^3^Hamad Medical Corporation, Cardiothoracic Surgery, doha, Qatar; ^4^Hamad Medical Corporation, Cardiac Anesthesia, Doha, Qatar

**Introduction:** Rhabdomyolysis ( RML) post aortic surgery probably affects the renal outcome adversely [1,2]. There is no robust data regarding the same in literature.

**Methods:** Retrospective single center data review; prior approval from Institutional review board. Patients were divided to two groups Group 1- with RML ( CK above cut off levels 1050 U/Litre) and Group 2 without RML. The determinants of RML and the impact of the same on outcome; predominantly renal function was evaluated. Chi-square tests are performed for categorical variables whereas, student t tests (un-paired ) are performed with continuous variables. Correlation is performed between Creatine kinase and creatinine rise. P value 0.05 (two tailed) is considered for statistical significant level.

**Results:** Out of 33 patients, 21 patients (63.64%) developed Rhabdomyolysis ( GROUP RML) and 12 did not( GROUP NON RML). Demographic and intraoperative factors had no significant impact on the incidence of RML. There was a significantly higher incidence of renal complications including new postoperative dialysis in the RML group. Other morbidity parameters were also higher in the RML group.

**Conclusions:** There is high prevalence of RML after aortic dissection surgery - Identification of risk factor and early intervention might help to mitigate the severity of renal failure

**References**

1. Omar AS, et al. BioMed Res Int, 2016;2016:7497936

2. Anthony DG, et al. Crit Care Med.2011; 39:1992-1994.

### P119 Early postoperative leukocytosis as a marker of cardiac surgery outcome. A single center retrospective study

#### S Hanoura, A Omar, H Osman, S Aboulnaga, M Eissa, Y Shouman, R Singh, A AlKhulaifi

##### Hamad Medical Corporation, Cardiothoracic surgery/Anesthesia and Intensive Care, Doha, Qatar

**Introduction:** Despite of the modern advances in cardiac surgeries, cardiopulmonary bypass (CPB) is still widely used that is associated with variant degrees of acute systemic inflammatory reaction where leukocytosis emerges as an early sign [1]. We hypothesize that early post operative leukocytosis (within 36 hours) could be associated with post cardiac surgeries morbidity.

**Methods:** Single center retrospective observational study over three years. We examined 1145 patients, out of these we recruited 924 patients underwent cardiac surgeries under CPB machine. Patients with off pump cardiac surgeries, preoperative sepsis, recent steroid use, renal failure, preoperative pneumonia, and emergently operated were excluded from the study. Patients were divided into 2 groups based on early white cell count (WBCs), group I: Control WBCs <12000 cells/mL (304 patients) and group II with WBCs > 12000 cells/mL (620 patients).

**Results:** Patients at group II had significantly higher incidence of acute kidney injury and ventilator associated pneumonia (180 vs 70, 20 vs 0 p= 0.03 and 0.00). The Length of mechanical ventilation and the length of hospital stay were significantly higher in group II (4.9±38.1 vs 9.6±20 hours, and 10±11.9 vs 7.8±5.3 days with P-Value 0.00 and 0.000 respectively). Moreover, surgical re-exploration was significantly higher in group II (66 vs 20, P-Value 0.05).in the meanwhile patients in group II had significant prolonged bypass time and aortic cross clamp time (117±46.6 vs 110.±36.5 and 74±36 vs 70±31 P=0.002 and 0.007) respectively.

**Conclusions:** Asymptomatic high WBCs post cardiac surgery had a prevalence of 67% in our study and carries a significant increased risk of morbidity intensive care stay. Further studies are needed for validating and explaining these findings.

**Reference**

Lamm G, et al: J Cardiothorac Vasc Anesth 20:51-56, 2006

### P120 Postoperative thrombocytopenia after isolated aortic valve replacement

#### F Ampatzidou, R Ioannidis, A Dimaki, G Kechagioglou, N Mihail, G Drossos

##### G.Papanikolaou General hospital Thessaloniki, Cardiac Surgery ICU, Thessaloniki, Greece

**Introduction:** Transient thrombocytopenia is not unusual after aortic valve replacement. The aim of the study was to investigate incidence and risk factors associated with this phenomenon

**Methods:** In this study we retrospectively analyzed platelet counts during the first five postoperative days, from patients who underwent isolated aortic valve replacement from January 2015 to November 2018 in our Cardiothoracic ICU department. Group A consisted of pts with nadir platelet count less than 60 x 109/L and Group B the rest of the cohort. The following factors were compared between the groups: age >75 years old, gender, pre-op platelet count less than 150x 109/L (and more than 80x 109/L), chronic renal failure(GFR < 60ml/min/1.73 m2), pre-op use of aspirin, pre-op use of clopidrogrel, cardiopulmonary bypass time (CPB)>120 min, use of sutureless prosthetic valve, and transfusion with more than 3 red blood cell units(RBC). Aspirin and clopidrogrel were stopped 5 days before the procedure. Chi square test was used for the statistical analysis

**Results:** A total of 198 patients underwent isolated aortic valve replacement, during the study period. Platelet count < 60 x 109/L was found in 28(14.1%) pts consisted group A mean aged 76.2±6.4 vs 68.8±11.1. No correlation was found with gender (p=0.85), aspirin use (p=0.06), clopidrogrel use (p=0.67) and CPB time>120 min(p=0.15) Statistical significant factors are shown in Table 1.

**Conclusions:** Postoperative thrombocytopenia after aortic valve replacement was associated with age >75, low pre-op platelets count, chronic renal failure, and sutureless aortic valve. All patients with thrombocytopenia were transfused with >3 RBC units

**Table 1 (abstract P120).** Statistical significant factors

	Group A n=28	Group B n=170	p value
Age>75 n,%	19(67.9%)	59 (34.7%)	0.001
Low pre-op PLT n,%	8(28.6%)	20(11.7%)	< 0.01
Chronic Renal Failure n,%	8(28.6%)	20(11.7%)	< 0.01
Sutureless valve n,%	19(67.9%	35(20.6%)	< 0.01
Transfusion>3RBC n,%	28(100%)	7(4.1%)	< 0.01

### P121 Characterization of multi-view hemodynamic data by learning mixtures of multi-output regressors

#### E Lei^1^, K Miller^1^, M Pinsky^2^, A Dubrawski^1^

##### ^1^Carnegie Mellon University, Auton Lab, Pittsburgh, United States; ^2^University of Pittsburgh, Department of Critical Care Medicine, Pennsylvania, United States

**Introduction:** We investigate whether central venous pressure (CVP) pressure waveform signal can be informative in detection of slow bleeding in post-surgical patients. We apply a novel machine learning method to analyze CVP datasets to characterize bleeding in a porcine model of fixed rate blood loss.

**Methods:** Thirty-eight pigs were anesthetized, instrumented with catheters, kept stable for 30 minutes, and bled at a constant rate of 20ml/min to mean arterial pressure of 30 mmHg. CVP waveforms were extracted from inspiration and expiration phases of respiration and statistically featurized. The proposed machine learning method, Canonical Least Squares (CLS) clustering, identifies correlation structures that differ between subsets of observations. We extend it to supervised classification. Both clustering and classification methods yield human-interpretable models that reflect distinctive patterns of correlations within CVP waveforms.

**Results:** We conducted three experiments to discover structure in the physiological response to bleeding. First, we clustered respiration cycles with full knowledge of blood loss. The color-coded cluster assignments are shown in the Figure 1. They are consistent with escalation of bleeding. Second, we deployed clustering on only CVP features without blood loss. Temporal structure was complemented with some subject-specific clusters (Fig 2). Third, we ran CLS classification to decide whether an observation came from before or after the onset of bleeding (performance shown in the Table 1).

**Conclusions:** Our results show that the CVP waveforms carry information about the physiologic status of the subject and indicate the amount of blood lost. Clusters still correspond to bleeding status even when no information about bleeding was available to the algorithm. CLS clustering enables a detailed yet interpretable view of discovered structures in complex waveform data.

Work partially funded by DARPA FA8750-17-2-0130 and NIH GM117622.

**Table 1 (abstract P121).** Bleeding Classification Performance

	Single cluster CLS	Final CLS	Random forest
AUC	.70 +/- .13	.86 +/- .06	.89 +/- .07
TPR @ .10 FPR	.47 +/- .19	.67 +/- .15	.76 +/- .17
TPR @ .01 FPR	.22 +/- .13	.50 +/- .19	.61 +/- .21
FPR @ .50 TPR	.24 +/- .15	.06 +/- .06	.07 +/- .08

**Fig. 1 (abstract P121).**
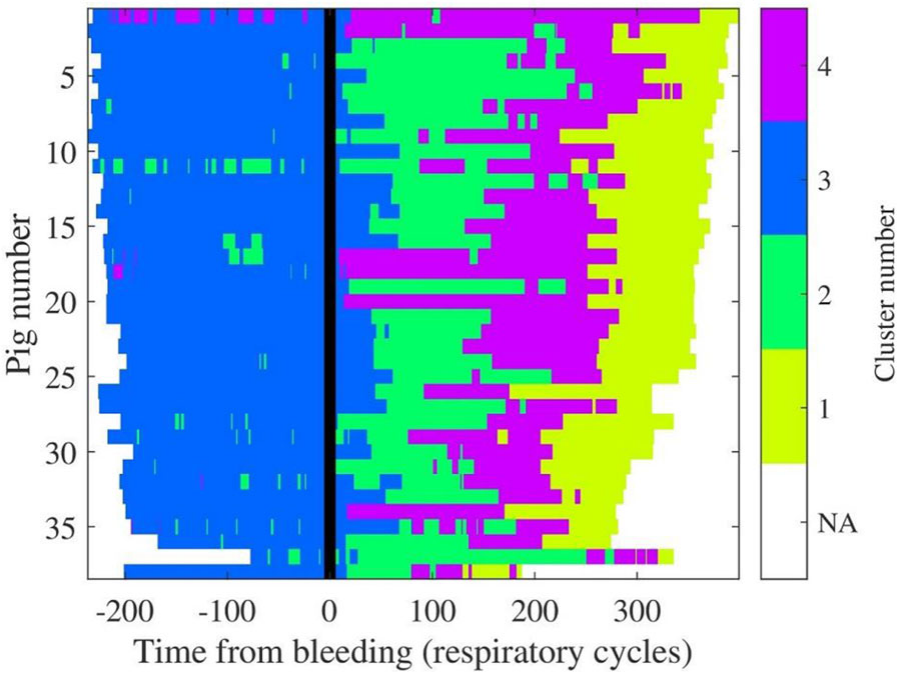
CLS cluster assignments with 4 clusters when blood loss is known

**Fig. 2 (abstract P121).**
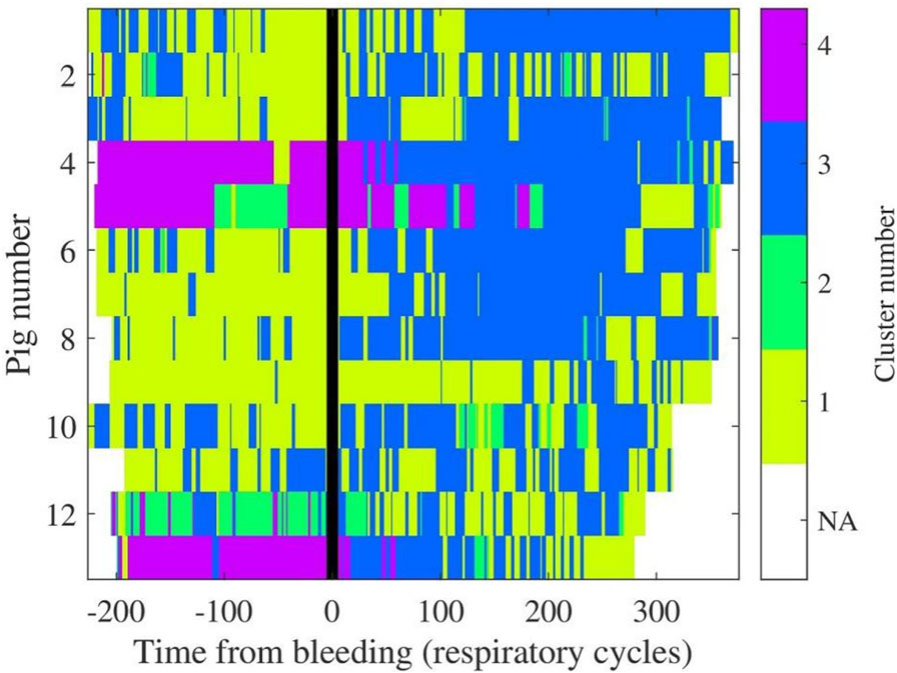
CLS cluster assignments with 4 clusters when blood loss is unknown

### P122 Minimally invasive thoracotomy is a ICU proven alternative approach for mitral valve surgery

#### E Van laeken^1^, T Bové^2^, K Vandewiele^2^, K Lapage^3^, H Peperstraete^1^

##### ^1^Ghent University Hospital, Intensive Care, Gent, Belgium; ^2^Ghent University Hospital, Cardiac Surgery, Gent, Belgium; ^3^Ghent University Hospital, Anesthesiology, Gent, Belgium

**Introduction:** We aimed to evaluate impact of minimally invasive thoracotomy (MIVT) versus sternotomy on patients (pts) receiving mitral valve surgery.

**Methods:** A retrospective observational study. Between 18/01/05 and 04/05/17 and after propensity score matching we retained 340 pts (95 sternotomy and 245 MIVT) (Table 1). We used SPSS 25 for parametric and non-parametric tests (p<0.05 = statistically significant).

**Results:** Hospital stay was 14 (4) days in the sternotomy group and 13 (4) days in the MIVT group, p=0.016.

On day (D) 1, ALT was higher in the MIVT group 30 ±28.6U/L vs. 22.1 ±19.0U/L, LDH was higher in the sternotomy group 533.5 ±187.0 U/L vs. 356.8±143.6U/L. Sternotomy gave lower thrombocyte counts 144.8 ±46.3 vs. 157.6 ±49.8 10³/μL, p=0.031; lower PT 67.8±9.5 vs. 70.8 ±11.0 %; and longer aPTT 39.7±8.2 vs. 37.4 ±5.57sec. So, clotting is less activated in MIVT. Inflammation is activated, at D2 MIVT led to higher white blood cell counts 10.9 (4.7) 10³/μL vs. 9.5 (4.4) 10³/μL; p=0.049, but a lower CRP 16.3 (9.4) mg/L vs 165.0 (67.0)mg/L, p<0.001.

Urea levels on D1 and 2 were lower in the MIVT group, 0.4 ±0.15 vs. 0.4 ±0.17g/L and 0.4 ±0.20 vs. 0.5 ±0.25g/L; p=0.001 & p<0.001. In the MIVT group creatinine kinase was higher 701.0 (595.0) U/L vs 431.0 (237.0), p<0.001, probably due to femoral cannulation and possible leg ischemia. We found no difference in: cardiac biomarkers, creatinine, glomerular filtration rate need for transfusion or revision, bleeding, length of ICU stay, cerebrovascular accidents, wound infections, pulmonary complications, prolonged ventilation, atrial fibrillation or need for dialysis.

**Conclusions:** We found no important differences in ICU outcomes when we compared sternotomy and MIVT approaches for mitral valve surgery.

**Table 1 (abstract P122).** Patient characteristics

	Sternotomy	MIVT	p
n	95	245	
mean age (SD)	72.0 (14.0)	68.0 (16.0)	0.044
Gender male (%)	37 (38.9)	117 (46.8)	0.225
Aortic clamp time (min)	61 (26)	87 (37)	<0.001
Extracorporeal circuit time (min)	96 (34)	134 (42)	<0.001
ICU stay (days)	1.4 (1.8)	1.3 (1.8)	0.366
Hospital stay (days)	14 (4)	13 (4)	0.016

### P123 Improving interstage mortality in hypoplastic left heart syndrome in an evolving congenital cardiac service in the UAE

#### V Sheward^1^, K Ramakrishnan^1^, L Kiraly^2^

##### ^1^Sheikh Khalifa Medical City, PCICU, Abu Dhabi, United Arab Emirates; ^2^Sheikh Khalifa Medical City, Paediatric Cardiac Surgery, Abu Dhabi, United Arab Emirates

**Introduction:** The Sheikh Khalifa Medical City (SKMC) Hospital is an 800 bed hospital, which has offered palliative cardiac surgery to babies with hypoplastic left heart syndrome (HLHS) for the last decade. During this time, the service has developed, with increasing patient numbers, increasing use of ECMO by the service, and expansion of the cardiac ICU.

**Methods:** A retrospective notes review was conducted of all admissions to the cardiac ICU since 2006. Patients with HLHS, who underwent Norwood 1 surgery, were analysed. Population statistics, surgical interventions, interstage events, and interstage mortality were reviewed.

**Results:** Over the last decade, the number of patients with HLHS who underwent Norwood 1 has increased. Interstage mortality has decreased, and is currently 30-40%. Significant morbidity was not seen at a rate higher than in the international literature. Discharge planning, and community access to allied health professional services remained a concern.

**Conclusions:** The paediatric congenital cardiac surgical service in the United Arab Emirates is relatively new (compared to some services around the world). Interstage mortality in HLHS is improving as a result of programme development, surgical progress and postoperative care. In the interstage period, there is currently no home monitoring programme in place. Some patients were found to have had very extended hospital admissions. Improved community support may reduce interstage mortality further, as well as improve the social situation of many of these patients.

### P124 Five year experience of off-pump surgical revascularization in patients with multi-vessel coronary disease

#### A Khadzhibaev^1^, A Abdurakhmanov^1^, M Obeid^1^, O Mashrapov^1^, N Rakhimov^1^, I Abdukhalimov^1^, U Ganiev^2^

##### ^1^Health Ministry Of The Republic Of Uzbekistan Republic Research Center Of Emergency Medicine Of Uzbekistan, Cardiosurgery, Tashkent, Uzbekistan; ^2^Health Ministry Of The Republic Of Uzbekistan Republic Research Center Of Emergency Medicine Of Uzbekistan, Cardiology, Tashkent, Uzbekistan

**Introduction:** Surgical revascularization in patients with multi-vessel coronary artery disease still raises many questions. The aim of the study was to analyze the single center results of off pump CABG in patients with multivessel coronary disease.

**Methods:** This retrospective study analyzed 1020 patients with history of coronary artery disease who underwent off-pump CABG surgery in 2013-2018 years at the Republican Research Center for Emergency Medicine. The average age of the patients was 58.2±0.95 years. Women were 309 (30.3%) and men - 711(69.7%). Most patients(76.9%) had III Class NYHA, and 12.3% and 10.8% patients IV and II class respectively. 283 patients had concomitant diseases: diabetes mellitus and COPD. The majority of patients were diagnosed with unstable coronary artery disease (94.4 %); 5.6 % of patients underwent surgery based on emergency indications or with acute myocardial infarction.

**Results:** The mean surgery duration was 210±1.7 minutes. The mean amount of bypasses (bypass index) was 3.2±0.2, in 840 (82.3%) cases LIMA was used as a preferred graft for LAD bypass, in 30 (3%) patients with concomitant carotid and coronary artery disease were performed simultaneous interventions. In 95 (9.3%) cases due to the haemodynamical instability we turned on-pump. Postoperative complications were observed in 112(10.9%) patients. We lined out the prevalence of cardiac complications, such as heart failure and rhythm disturbances, observed in 87 (8.5%) and 53 (5.1%) patients respectively. Hospital mortality rate was 2.8% (29/1020). The cause of mortality in all cases was acute heart failure, due to the initial severity of the disease, and in 11(1.07%) cases an acute myocardial infarction was diagnosed. Duration of postoperative period was 7.8 ± 0.9 days.

**Conclusions:** Off-pump Coronary artery bypass grafting can be safely performed with relatively low incidence of mortality and postoperative morbidity.

### P125 Prognostic value of mid-regional pro-adrenomedullin and mid-regional pro-atrial natriuretic peptide as predictors of multiple organ dysfunction development and ICU length of stay after cardiac surgery with cardiopulmonary bypass in adults

#### A Goncharov^1^, D Popov^2^, M Rybka^1^

##### ^1^Bakulev National Medical Research Center of Cardiovascular Surgery, Department of Anaesthesiology, Moscow, Russia; ^2^Bakulev National Medical Research Center of Cardiovascular Surgery, Laboratory of Clinical Microbiology and Antimicrobial Therapy, Moscow, Russia

**Introduction:** One of the most harmful complications after cardiac surgery with cardiopulmonary bypass is a syndrome of multiple organ dysfunction (MODS). We consider that mid-regional pro-adrenomedullin (MR-proADM) and mid-regional pro-atrial natriuretic peptide (MR-proANP) plasma concentrations can be used as predictors of MODS development and LOS in ICU.

**Methods:** Thirty six adult patients (mean age 60 years, 27 male) with cardiovascular diseases undervent cardiac surgery with cardiopulmonary bypass (heart valve(s) replacement – 20 (55.6%) patients, aorta and it`s branch surgery – 14 (38.9%) patients, valvular surgery and coronary artery grafting – 2 (5.5%) patients). NYHA heart failure class II was in 3 (8.3%) patients, III – in 27 (75%) patients, IV – in 6 (16.7%) patients. In the dynamics levels of MR-proADM and MR-proANP were measured in the venous blood with the Kryptor compact plus analyzer (Thermo Fisher Scientific, Germany) before 1 day and on the 1st and 6th days after surgery. All patients were divided into subgroups according to the lengths of stay in the ICU and the development of MOD in the postoperative period. The data are shown as median and 25th and 75th percentiles. The data were compared by Mann-Whitney U-test, p-value of <0.05 was considered statistically significant.

**Results:** Levels of MR-proANP did not significantly change at the study stages and did not have a significant difference between subgroups. The levels of MR-proADM increased in the first postoperative day and remained elevated for 6 days. This increase was significantly higher in subgroups of increased LOS in ICU and with MODS. The data are shown in the Table 1.

**Conclusions:** MR-proADM can be used as predictor of MODS and LOS in the ICU for adult patients underwent cardiac surgery with cardiopulmonary bypass.

**Table 1 (abstract P125).**
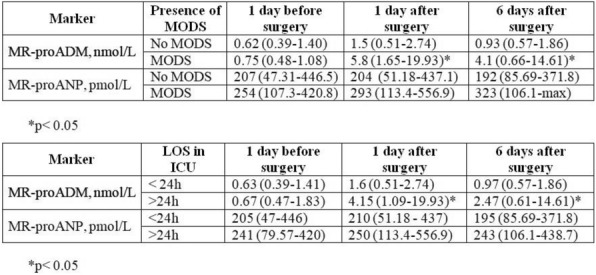
MODS development and LOS in ICU prediction

### P126 Predictive factors of prolonged postoperative ICU stay aftercardiac surgery

#### F Ampatzidou, R Ioannidis, A Dimaki, N Mihail, G Kechagioglou, G Drossos

##### G.Papanikolaou General hospital Thessaloniki, Cardiac Surgery ICU, Thessaloniki, Greece

**Introduction:** Prolonged intensive care unit (ICU) stay after cardiac surgery is associated with increased mortality and cost .The aim of this study was to investigate factors influencing prolonged ICU stay.

**Methods:** Consecutive patients who underwent cardiac surgery from June 2012 to October 2018 in our Cardiothoracic department, were retrospectively investigated. Group A consisted of pts with prolonged stay defined as more than 3 days and group B the rest of the cohort. The following characteristics and perioperative factors were compared between the groups: smoking, diabetes, COPD, REDO(re-operation), ejection fraction (EF)<50%, emergent procedure, cardiopulmonary bypass time (CPB)>120 min, low cardiac output syndrome (LCOS), acute kidney injury(KDIGO) and mortalityChi square test was used for the statistical analysis.

**Results:** From a total of 3163 patients who underwent cardiac surgery, 247 pts consisted group A mean aged 66.8±10.1 vs 65.2±10.6 and Euroscore II 5.7±7.8 vs 2.1±3.1.No correlation was found with smoking (p=0.28) and diabetes(p=0.67) Statistical significant factors are shown in Table 1. Mortality was 20.2% for group A and 1.9% for group B(p<0.01).

**Conclusions:** History of COPD, pre-op EF less than 50 %, redo and emergent procedures, prolonged CPB time, postoperative AKI and LCOS have statistical significant correlation with prolonged ICU stay after cardiac surgery. Mortality in this group of patients is higher.

**Table 1 (abstract P126).** Statistical significant factors

	Group A	Group B	p value
COPD n,%	51(20.6%)	426(14.6%)	0.012
REDO n,%	20(8.1%)	60(2.1%)	<0.01
EF<50% n,%	98(39.5%)	786(27%)	<0.01
Emergent n,%	57(23%)	139(4.8%)	<0.01
CPB n,%	148(59.9%)	749(25.7%)	<0.01
LCOS, n,%	72(29%)	121(4.2%)	<0.01
AKI n,%	146(58.9 )	347(11.9%)	<0.01

### P127 Identifying coagulation thresholds associated with bleeding complications in pediatric post-cardiotomy extracorporeal membrane oxygenation

#### L Thalji^1^, G Schears^1^, K Bohman^1^, D Kor^1^, J Stubbs^2^, M Nemergut^3^

##### ^1^Mayo Clinic, Department of Anesthesiology and Perioperative Medicine, Rochester, United States; ^2^Mayo Clinic, Department of Laboratory Medicine and Pathology, Rochester, United States; ^3^Mayo Clinic, Department of Anesthesiology and Perioperative MedicineMedicine, Rochester, United States

**Introduction:** Hemorrhagic complications of extracorporeal membrane oxygenation (ECMO) pose a major morbidity and mortality. Optimal anticoagulation strategies balancing risks of bleeding and thrombosis in children are poorly understood. We aimed to identify factors associated with non-surgical bleeding in the first 12 ECMO hours.

**Methods:** We evaluated all pediatric (<18 yrs) post-cardiotomy patients requiring ECMO between Dec 2002–July 2017 stratifying them by presence/absence of surgical bleeding. Non-surgical bleeding was defined as chest tube output >3cc/kg/hr during the first 12-hours not requiring reoperation. Patient characteristics and coagulation parameters at various time points after ECMO initiation were compared between groups, and receiver operator characteristic (ROC) curves were constructed to identify models and thresholds with optimal predictive performance.

**Results:** Amongst 117 patients, 63 (53.8%) were male, median (IQR) age and weight were 47.4 days (7.3 – 394.2), and 4.0kg (3.0 – 8.5). The 30 patients (25.6%) who experienced non-surgical bleeding were younger (7.3 vs 109.5 days, p<0.001) and smaller (3.3 vs 4.8kg, p<0.001). Operative and ECMO durations, and anticoagulant choices were comparable. Coagulation parameter differences between groups are shown in Table 1. Univariate ROC analysis revealed baseline kaolin thromboelastography R-time to best predict bleeding [OR per 10min increase 1.21 (1.06 - 1.37), p=0.004, AUC 0.76]. This persisted across multivariable models, with adjustment for weight [OR per 1kg decrease 1.70 (1.11 - 2.59), p=0.014] and the presence of hypoplastic left heart syndrome [OR 5.09 (1.21 - 21.32), p=0.026] producing the best predictive performance [OR per 10min R-time increase 1.26 (1.06 - 1.48), p=0.007, AUC 0.89], Figure 1.

**Conclusions:** Deranged coagulation parameters, particularly kaolin R-time may predict non-operative bleeding in pediatric ECMO patients. These findings may guide therapeutic anticoagulation while avoiding hemorrhagic sequelae in at risk patients.

**Table 1 (abstract P127).** Baseline post-cardiotomy coagulation parameters following ECMO initiation by bleeding status

Coagulation Parameter	Bleeders (n=30)	Non-Bleeders (n=87)	P-value
Activated Partial Thromboplastin Time (sec)	150.0 (125.5-150.0)	107.0 (67.0-150.0)	0.006
International Normalized Ratio	2.6 (1.7-3.0)	2.1 (1.6-2.8)	0.419
Activated Clotting Time (sec)	422.0 (248.2-1067.5)	259.0 (160.0-506.0)	0.008
Platelet Count (x109/L)	50.0 (38.7-81.2)	59.0 (40.0-82.0)	0.245
Fibrinogen (mg/dl)	106.5 (74.7-151.2)	139.5 (95.0-181.5)	0.029
Kaolin TEG: R-time, Alpha Angle, Max Amplitude	54.4(28.0-87.3), 13.9(2.6-34.6), 31.1(10.9-40.8)	18.5(11.8-43.6), 29.0(13.7-48.7), 42.4(29.9-52.4)	<0.001, 0.002, 0.002
Heparinase TEG: R-time, Alpha Angle, Max Amplitude	11.3(8.4-23.5), 31.9(20.8-43.9), 36.3(22.9-49.1)	9.4(7.2-14.2), 42.5(30.5-54.2), 45.6(37.9-54.9)	0.041, 0.029, 0.016

ECMO = extracorporeal membrane oxygenation, TEG = thromboelastography, R-time = reaction time. Values are median (interquartile range), p-values calculated using the Wilcoxon Rank Sum test. Median baseline labs values are shown, comparable findings were noted when evaluating labs at 4- and 8-hours, and when evaluating maximum and minimum values for patients within the initial 12 ECMO hours. No significant findings for TEG Lysis times

**Fig. 1 (abstract P127).**
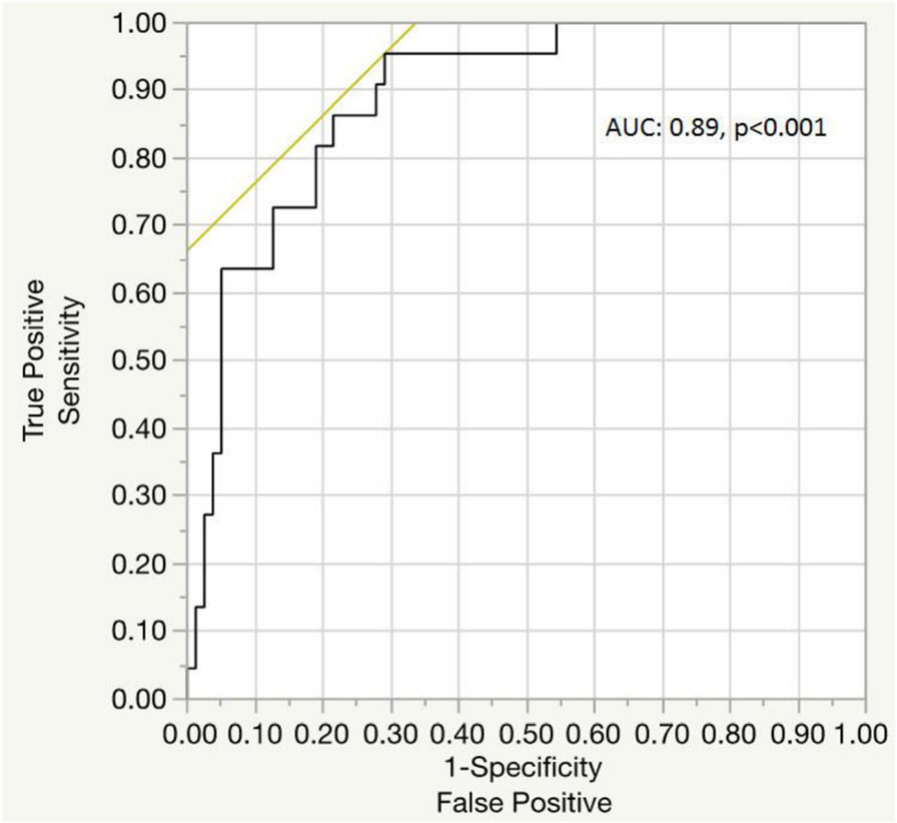
Multivariable ROC curve for Kaolin Thromboelastography R-time, adjusting for weight and the presence of hypoplastic left heart syndrome

### P128 Impact of technical features of intra-aortic balloon pump placement on device related morbidity and mortality in cardiac surgery patients

#### R Samalavicius^1^, L Puodziukaite^2^, I Norkiene^2^, I Misiuriene^1^, P Serpytis^3^

##### ^1^II Department of Anaesthesiology, Center of Anaesthesiology, Intensive Care and Pain Management, Vilnius University Hospital Santaros Klinikos, Vilnius, Lithuania; ^2^Clinic of Anaesthesiology and Intensive Care, Vilnius University, Faculty of Medicine, Vilnius, Lithuania; ^3^Clinic of Cardiac and Vascular Diseases, Vilnius University, Faculty of Medicine, Vilnius, Lithuania

**Introduction:** Intra-aortic counterpulsation remains the most frequently used mechanical assist device worldwide. We aimed to evaluate intra-aortic balloon pump (IABP) device related morbidity and mortality in cardiac surgery patients.

**Methods:** Single centre retrospective review of 402 cardiac surgical patients treated with IABP counterpulsation between 2013 and 2018 in the University Hospital. Patient outcomes, complications and IABP insertion technical characteristics were analysed. According to tip position patients were divided into three groups: acceptable (at T2-T4 vertebrae), malposition (T5-T6) or severe malposition (at T7 or below).

**Results:** Median patient age was 67[IQR, 59-75] years, 289(71.9%) were male, median EuroSCORE II was 4.55[IQR, 2.5-10.9]%. Indications for IABP treatment were: treatment before surgery in 44(10.9%), prophylactic use in high risk 171(42.5%), hemodynamically unstable patient before CPB in 5(1.3%), during off-pump CABG in 10(2.5%), facilitate weaning from CPB in 42(10.5%) and low cardiac output following surgery in 130(32.3%) patients. 327(86.3%) patients survived to ICU discharge. In hospital mortality was 5.3% in elective IABP placement group vs 26.4% in urgent IABP implementation group (p<0.001). In 23(5.7%) patients due to severe aortoiliac disease IABP was placed intraoperatively through ascending aorta. In remaining 379 patients percutaneous IABP placement was performed, balloon position was acceptable in 134(35.4%), malpositioned in 172(45.4%), severely malpositioned in 64(16.9%) and unavailable for 9(2.4%) cases. In 340(89.7%) patients sheathless technique was used. Vascular complications rate was 15(3.7%) and showed tendency to be lower (2.65% vs 10.25%, p<0.05) when sheathless technique was used.

**Conclusions:** Elective balloon placement was associated with lower morbidity and mortality. Sheathed insertion showed a tendency to be associated with higher rate of vascular complications. Suboptimal IABP tip position was not associated with higher morbidity or mortality rates.

### P129 A cut-off point of cardiac troponin elevation indicating poor prognosis for critically ill patients admitted in an intensive care unit: a retrospective study

#### P Vasileiou, A Boultadakis, E Tsigou, E Boutzouka, T Katsoulas, G Fildissis

##### Timiou Stavrou and Noufaron, ICU, Attiki, Greece

**Introduction:** Elevated cardiac troponin (cTn) level in patients (pts) admitted in the intensive care unit (ICU) is multifactorial and has been associated with a worse prognosis. The aim of the study was to review the frequency and the main cause of cTn elevation and to calculate a discriminating index.

**Methods:** We retrospectively assessed all pts admitted in our eight-bed general ICU during a 6-month period with at least one measurement of cTn during their ICU stay. We recorded clinical characteristics, the level of cTn on admission, the maximum cTn during ICU stay and the possible causes of elevation. Variables are expressed as mean ± SD or as median and Interquartile Ratio (IR), according to the normality of their distribution. Student´s Ô test or the Mann Whitney U tests were used to compare the group of elevated cTn with the group of normal cTn. The prognostic performance of elevated cTn was evaluated by the Receiver Operating Characteristics (ROC) curve. Statistical analysis was performed using SPSS version 21.0 (SPSS, Inc., Chicago, Illinois).

**Results:** In 84 out of 92 pts that cTn was measured at least once, abnormal levels (>15.6 pg/ml) were found in 58 (69%) of them, and the maximum cTn value was 633 (1718.25) pg/ml. The clinical characteristics of the pts are depicted in Table 1. Sepsis was the main cause of troponin elevation, which complicated by Acute Kidney Injury (AKI) in 20 pts (34%). Maximum cTn, AKI and the difference of maximum - admission cTn (ÄcTn) differed significantly between pts who survived and pts who died (p=0.029 and 0.001, respectively). The Area Under the Curve (AUC) was 0.753 and the optimal prognostic cut-off value of ÄcTn was 57 pg/ml with a sensitivity of 0.656 and a specificity of 0.833

**Conclusions:** Raised cardiac troponin values is a frequent finding in ICU pts and sepsis is the driving cause. AKI and the difference between maximum and admission cTn measurements differ significantly between pts who survive and pts who die. An elevation of cTn during ICU hospitalization >57 pg/ml seems to be a threshold indicating poor prognosis regarding both mortality and AKI.

**Table 1 (abstract P129).** Clinical characteristics of the pts

	High cTn (n=58)	Normal cTn (n=26)	P value
Gender (Male)	29	9	0.239
Age (years)	72 (16)	61 (33)	0.002
Mortality	32 (57.1%)	1 (3.8%)	<0.001
APACHE II	24.27 ±8.99	12.76 ±5.62	<0.001
SOFA	9.71 ±3.79	4.95 ±3.35	<0.001
LOS	14(23)	3(9)	0.004

### P130 The prognostic role of NT-pro-BNP in septic patients with elevated troponin T level

#### A Shilova^1^, D Shchekochikhin^2^, M Gilyarov^2^, A Svet^2^, M Petrushin^3^

##### ^1^Moscow City University Hospital #1 n.a. Pirogov, ICCU, Moscow, Russia; ^2^Moscow City University Hospital #1 n.a. Pirogov, Cardiology, Moscow, Russia; ^3^Tver Regional Hospital, ICU, Tver, Russia

**Introduction:** Sepsis is frequently accompanied with release of cardiac troponin T (TnT) and NT-pro-BNP, but the clinical significance of this myocardial injury and cardiac dysfunction remains unclear [1]. TnT is known to be an independent predictor of mortality, whereas the prognostic role of NT-pro-BNP is uncertain.

**Methods:** We have prospectively enrolled 67 patients with sepsis in our intensive care unit from June the 1st 2017 to June the 1st 2018. Subjects with a clinically apparent cause of troponin release were excluded. High-sensitivity cardiac troponin I (hs-cTnI), NT-pro-BNP, CRP and PCT and lactate concentration in plasma were measured at admission

**Results:** Mean SOFA on admission was 6,1 points. 38.8% of patients were women. Mean age ranged from 52 ±7.8 years in men and 54.38±9.2 years in women. 37.3% of patients were admitted in shock. 70.15% patients underwent noncardiac surgery. 30 days mortality rate was 62.7% (42 pts). Average TnT 0.21±0.09 ng/dl. Average Nt-proBNP 238.8±72.4 ng/ml. TnT and NT-pro-BNP levels on admission were significantly lower in survived patients. 0.11 vs 0.28 ng/ml, p=0.007 for TnT and 194.4 pg/ml vs 272.3 pg/ml, p= 0.05 for NT-pro-BNP. There was significant correlation between TnT level and PCT, APACHE-II, SOFA, lactate and NT-pro-BNP values (p < 0.05). We’ve performed stepwise multiple regression model, that included age, PCT, CRP, APACHE-II, SOFA, lactate, TnT and NT-pro-BNP. Elevation of NT-pro-BNP was founded to be the only independent predictor of mortality in septic patients with elevated HR 1.6 [1.15; 2.24], p= 0.013.

**Conclusions:** Elevated NT-pro-BNP is an independent predictor of unfavorable prognosis in septic patients with elevated TnT level.

**Reference**

1. Fei Wang et al. Crit Care. 2012; 16(3): R74.

### P131 The incidence and risk factors of acute kidney injury after left ventricular asisst device implantation

#### A Ozdemirkan, H Sahinturk, F Atar, O Ersoy, P Zeyneloglu, A Pirat

##### Baskent University Faculty of Medicine, Anesthesiology and Critical Care, Ankara, Turkey

**Introduction:** Cardiac surgery-associated acute kidney injury (AKI) is a serious complication of cardiac surgery with an incidence of 5-42%. The aim of the study was to evaluate AKI in the early postoperative period after left ventricular assist device (LVAD) surgery and to compare patients with and without AKI to determine incidence, risk factors and clinical outcomes.

**Methods:** In this retrospective cohort study, the medical records of all patients aged between 18-75 years undergoing LVAD implantation from January 2011 to December 2016 was reviewed. AKI was defined according to KDIGO criteria.

**Results:** Out of 57 patients, 10 (18%) were female and the mean age of the cohort was 44.6±16.1 years. Thirty six patients (63%) developed AKI following LVAD implantation. Duration of cardiopulmonary bypass was longer in patients with AKI (162.5±58.2 vs 128.7±48.6, p=0.039). Patients with AKI had lower mean blood pressures (54.9±13.1 vs 62.8±6.9, p=0.013), higher frequency of norepinephrine usage [16 (44%) vs 3(14%), p=0.020), and higher cumulative fluid balance on the postoperative first day (1.4±0.6 L vs 1.2±0.9L, p= 0.042). The frequency of prolonged mechanical ventilation among patients with AKI was higher when compared to those without AKI [21 (58%) vs 5 (24%), p= 0.012]. Logistic regression analysis revealed duration of cardiopulmonary bypass, mean blood pressure and cumulative fluid balance on the postoperative first day as independent risk factors for AKI (OR:1.013, CI 95% 1.000-1.025, p:0.045; OR: 0.929, CI 95% 0.873-0.989, p:0.021; OR: 1.001, CI 95% 1.000-1.001 respectively). Hospital mortality (58% vs 24%, p=0.012) and 30-day mortality (39 % vs 5%, p= 0.005) were significantly higher in patients who had AKI.

**Conclusions:** AKI develops in 63% of patients after LVAD surgery. Risk factors for occurance of AKI includes longer duration of cardiopulmonary bypass, lower mean blood pressures and higher cumulative fluid balance on the postoperative first day.

### P132 Early inotropic therapy in patients after venoarterial extracorporeal membrane oxygenation implantation - a single center experience

#### V Zotzmann, C Lang, T Wengenmayer, D Dürschmied, C Bode, D Staudacher

##### University Heart Center, Cardiology, Freiburg, Germany

**Introduction:** Venoarterial extracorporeal membrane oxygenation (VA-ECMO) increases afterload. This can negatively impact left ventricular (LV) output in case of decreased LV-function resulting in increased filling pressures, pulmonary edema, LV-distension or even stasis in the pulmonary circulation. If positive inotropic agents should be used is discussed controversial in literature.

**Methods:** Here, we report data of VA-ECMO-patients, treated with dobutamine, levosimendan, suprarenin or no inotropic agens, in respect of 30-day survival. All data were collected retrospectively (10/2010 to 10/2018) at a single center, all patients with a survival below 24 hours were excluded. While treatment of VA-ECMO patients is strongly guided by standard operation procedures at our institution, no recommendation on positive inotropic therapy could be made.

**Results:** A total of 232 VA-ECMO patients were evaluated, of which 47 patients were treated with levosimendan within 48 hours after cannulation. 30day survival in the whole cohort was 41.9%. A total of 99 patients did not receive any positive inotropic therapy at 24 hours after implantation (survival 48.0%). Survival was best in the levosimendan plus dobutamine group 50%, followed by dobutamine mono-therapy 46.2% and levosimendan mono 41.2%. Survival with suprarenin mono was 33.3%, suprarenin plus levosimendan 25.0% and suprarenin plus dobutamine 18,8%. Pooling data, we found no evidence that levosimendan and/or dobutamine (survival 45.8%, n=83, p=0.882) improves survival over no inotropic therapy (Fig 1). Therapy with any combination including suprarenin however resulted in poor survival (27.7%, n=65, p=0.009). Adjustment for lactate levels or eCPR did not change the results.

**Conclusions:** This retrospective analysis of 232 VA-ECMO patients shows no evidence that early inotropic therapy improves outcomes in VA-ECMO patients. This conclusion is obviously biased by retrospective design. Until randomized data are available, suprarenin however should be avoided.

**Fig. 1 (abstract P132).**
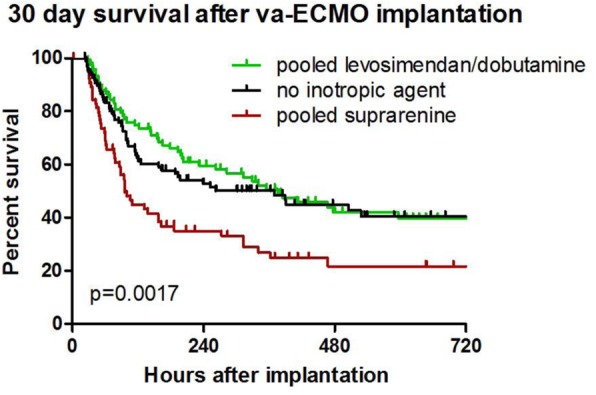
30 day survival after va-ECMO implantation

### P133 Survey of non-resuscitation fluids in septic shock

#### A Linden-sonderso^1^, P Bentzer^1^, M Spångfors^2^, J Undén^3^, T Kander^4^, D Griesdale^5^, J Boyd^6^, M Jungner^4^

##### ^1^Helsingborg Hospital, Department of Anesthesiology and Intensive Care, Helsingborg, Sweden; ^2^Lund University, Kristianstad Hospital, Department of Clinical Sciences Lund, Anesthesiology and Intensive Care Medicine, Kristianstad, Sweden; ^3^Hallands Hospital, Department of Operation and Intensive Care, Halmstad, Sweden; ^4^Skåne University Hospital, Department of Intensive and Perioperative Care, Lund, Sweden; ^5^Vancouver General Hospital, British Columbia, Department of Anesthesiology, Pharmacology and Therapeutics, Vancouver, Canada; ^6^St. Paul´s Hospital, British Columbia, Department of Intensive Care, Vancouver, Canada

**Introduction:** Positive fluid balance is associated with poor outcome in septic shock. The objective of the present study was to characterize non-resuscitation fluids in early septic shock.

**Methods:** Consecutive patients >18 years of age were screened for inclusion criteria during a 4-month period in 8 ICUs in Sweden and in Canada. Inclusion criteria were septic shock per SEPSIS-3 definition within 24 hrs of ICU admission. A maximum of 30 patients per center were included. Type, indication and volume of non-resuscitation fluids were recorded during the first 5 days of admission. Fluids other than colloids, blood products and crystalloids given at rate > 5ml/kg/h were considered to be non-resuscitation fluids. The study was registered on ClinicalTrials.gov (NCT03438097). Data are presented as median (interquartile range).

**Results:** A total of 201 patients were included between March 1st and June 30th 2018 (see Table 1 for demographics). Patients received 7886 (4051-12670) milliliters (ml) of non-resuscitation fluids and 3450 (2000-5625) ml of resuscitation fluids during the observation period. Non-resuscitation fluids consisted of vehicle for drugs 2418 (1289-4655) ml, glucose 1485 (0-3124) ml, crystalloids at a rate of < 5 ml/kg/h 600 (0-2000) ml, enteral nutrition 334 (0-1609) ml, enteral water 270 (0-995) ml and parenteral nutrition 0 (0-0) ml. Vehicles were mainly used for administration of vasoactive drugs and antibiotics, and total volumes were 455 (185-900) ml and 560 (125-1100) ml, respectively. Daily volume and type of fluid are presented in Figure 1. At day 2 and onwards non-reuscitation fluids represented the major part of total daily volume (Figure 1).

**Conclusions:** These recent data suggests that non-resuscitation fluids is the major source of fluids in septic shock already at day 2 after admission to the ICU. Strategies aimed at reducing this source of fluids may reduce positive fluid balance.

**Table 1 (abstract P133).** Patient characteristics

Number of patients	201
Female sex, n. (%)	71 (35)
Age, years, median (interquartile range)	69 (59–77)
Weight, kg, median (interquartile range)	78 (60–90)
Alive at 30 days, n. (%)	130 (64.6)
RRT, n. (%)	48 (23.9)
Mechanical ventilation, n. (%)	135 (67.1)
Surgery, n. (%)	58 (28.9)

Mechanical ventilation and RRT (renal replacement therapy) at any time during the study period. Surgery as a cause of sepsis or for source control

**Fig. 1 (abstract P133).**
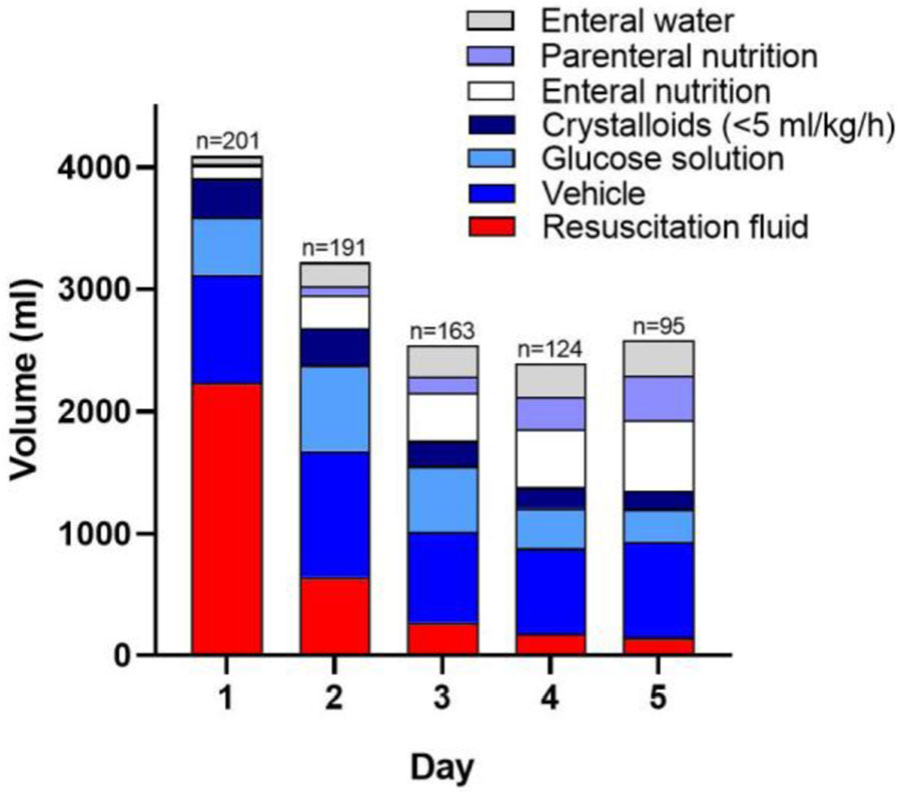
Median daily volume and type of fluids. Data for type of fluid each day are presented as percent of total daily volume. Please note that sum of the median daily volume does not equal sums of median non-resucitation and resuscitation fluids over the whole observation period as described in results because of the skewed distribution of data. Number of patients are (n)

### P134 Effect of targeted or standard protocols of intra-operative fluid therapy on early graft functions during live related renal transplantation- a randomized comparative trial

#### R Ramachandran^1^, A Singh^1^, V Rewari^1^, V Bansal^2^, B Ray^1^, A Trikha^1^

##### ^1^All India Institute of Medical Sciences, Anaesthesiology, Pain Medicine and Critical Care, New Delhi, India; ^2^All India Institute of Medical Sciences, Surgical Disciplines, New Delhi, India

**Introduction:** Aim was to compare different fluid hydration regimens, standard fluid replacement method and targeted fluid replacement before declamping of anastomotic renal vessels, on early graft function during live related renal transplantation (LRRT). Primary outcome of study was serum creatinine (creat) on first postoperative day

**Methods:** 40 consenting patients undergoing LRRT were randomized. In Group S- patients received intravenous fluid according to standard guidelines for intraoperative fluid infusion using Holliday-Segar rule. Patients in Group T- received intravenous fluids at start of vascular anastomosis to achieve a CVP of > 15 mm Hg at de-clamping of vessels. Serum creat, blood urea, serum electrolytes and urine output were measured in postoperative period. Hemodynamic parameters including blood pressure, CVP, cardiac output and cardiac index were compared in both groups

**Results:** Difference in serum creat on first post-operative day, 2.04 (0.73) and 2.31 (1.19) [Mean (SD)] in Group T and S, respectively, were statistically insignificant. Total urine output on first post-operative day was significantly more in Group T as compared to Group S (P value-0.03). Graft diuresis time and graft turgidity after vascular de-clamping were significantly better in Group T. Total volume of crystalloids infused during intra-operative period was significantly less in Group T (P value-<0.001)

**Conclusions:** Serum creat on first post-operative day was similar in patients receiving fluid according either to standard practice or according to focused infusion prior to de-clamping of vascular anastomosis despite intervention group receiving significantly less intravenous fluid intraoperatively. Graft turgidity score and diuresis time were significantly better with focused infusion

### P135 A retrospective analysis of intravenous (IV) fluid and medication administration in a United Kingdom district general Intensive Care Unit (ICU)

#### B Scrace, L Squire, D Ashton-Cleary, L Moore, M Spivey

##### Royal Cornwall Hospitals Trust, Critical Care, Truro, United Kingdom

**Introduction:** We aimed to ascertain the extent and make-up of fluid overload in critically ill patients and to identify whether delivery of more concentrated medications could reduce this. Positive fluid balance is associated with increased mortality [1]. A recent study has shown that the predominant component of fluid overload was from IV medications and maintenance fluid [2].

**Methods:** We reviewed 20 sequential patients admitted to our ICU with an APACHE II score of greater than 15 and a length of stay (LOS) greater than 72 hours. The patients’ electronic admission summary was interrogated to establish: length of stay (LOS) fluid balance at 72 hours, total volume administered as IV medications, total volume administered as maintenance fluid and total fluid administered as bolus fluid. Predicted reduction in volume resulting from administration of the most concentrated format of IV medications received in the first 72 hours LOS was calculated for each patient. The percentage reduction in total IV fluid administration was then calculated.

**Results:** 90% of patients had a positive fluid balance at 72 hours LOS. Median fluid balance at 72 hours was positive 4170.5 mls. IV medications (median 2423 mls) and maintenance fluid (median 3362.5 mls) represented a greater proportion of intravenous fluid administration than bolus fluid (median 1900 mls). Use of the most concentrated format of IV medications gave a predicted median volume reduction of 687.75 mls and a median percentage reduction in total IV administration of 11%.

**Conclusions:** The proportion of patients with a positive fluid balance and the breakdown of IV fluid administration reflect that seen in recent studies [2]. By administering medications in the highest concentration possible, reductions in fluid administration can be made. A shift away from using maintenance IV fluid in the acute phase of critical illness may lead to reductions in fluid overload.

**References**

1. Rhodes A et al: Intensive Care Med; 43:304–377. 2017

2. Silversides et al: Crit Care Med; 46:1600–1607. 2018

### P136 Impact of 10% intraoperative fluid overload on patients undergoing major abdominal surgery & admitted to general SICU

#### S Kongsayreepong, A Piriyapatsom

##### Siriaj Hospital, Mahidol University, Anesthesiology, Bangkok, Thailand

**Introduction:** Fluid overload(>10% of admission BW) has been associated with increased complications & organ dysfunction [1]. The aim of this study was to study the incidence &effects of intraop fluid overloads [IFO] on the incidence of prolonged ICU stays (>72 hr); 90 day mortality&periop complications in patients undergoing major abdominal surgery

**Methods:** A prospective, observational study was done on1,680 patients admitted to the general SICU from Jan 2014 - Dec 2017 after major abdominal surgery. study data included patients’demographic data; admitted & preop serum alb, Hb & creatinine up to 72 hrs; ASA physical status; surgery type & urgency; anesthesia type & duration; type & amount of intake & output; perioperative complications; septic shock on ICU admission; ventilator days, ICU & hospital LOS, APACHE II score on postop day 1, 90 day mortality.

**Results:** Incidence of IFO was 15.2%. Patients with IFO had significantly longer anesthetic times, lower preop & admitted serum alb; higher preop Hb, fluid balances; longer ICU & hospital LOS. Significantly higher combined general & neuraxial block & septic shock on ICU admission were seen in the IFO pts. Significantly higher periop complications (e.g. CHF, serious cardiac arrhythmias, intraabdominal hypertension, wound infection, anastomosis leakage) were found in IFO patients esp in septic shock patients. AKI was the most organ dysfunction (28.8%) found in IFO & 17% needed RRT from fluid overload. IFO was a significant predictor of prolonged ICU stay (OR 9.78; 95% CI 3.28 -10.9, p<0.001) & who had intraop fluid balances>130 mL/kg were significantly associated with higher 90-day mortality.

**Conclusions:** IFO had a high impact in critically ill patients undergoing major noncardiac surgery in terms of prolonged ICU stay, increasing periop major complications, and mortality. Care should be taken to prevent IFO.

**Reference**

1. Glatz T, BMC Surg 2017;17:6

### P137 Quantification of fluid needed for burns resuscitation in children

#### H Bangalore^1^, S Ray^2^, N Martin^3^

##### ^1^Great Ormond Street Hospital, Burns Intensive Care, London, United Kingdom; ^2^Great Ormond Street Hospital, Paediatric Intensive Care, London, United Kingdom; ^3^St Andrew´s Burns Center, Chelmsford, United Kingdom

**Introduction:** In children less than 30 kilograms, maintenance fluids are routinely added to the resuscitation requirements calculated using Parkland’s or other formulae. The contribution of this component for fluid resuscitation in children can add a significant quantity to total estimated fluid requirements. For example, in a child who is 10 kilograms with a 25% burn, the maintenance fluid requirement is 1000 mls per 24 hours and the resuscitation component per Parkland’s will be 4 x 10 x 25%=1000 mls. Hence, the maintenance requirement can exceed the resuscitation requirement in this child if the burn surface area is less than a 25 % burn. The contribution of maintenance fluids to the total fluid requirements in small children with thermal injuries is under-recognised and not frequently studied.

**Methods:** To understand the contribution of maintenance fluids to the total fluid requirements in children less than 30 kilograms who need resuscitation for thermal injuries of different sizes, we numerically simulated 1. children who had similar weights but different burn sizes and 2. Children with similar burn size but different weights. The results are as shown in Figure 1 and 2.

**Results:** 1. The contribution of maintenance fluids is greatest in smaller children with a smaller burn. 2. In bigger children and larger burns, as the total burn fluid requirements are higher, the proportion contributed by maintenance fluids to the total fluid requirements is smaller. 3. Decreasing the quantity of maintenance fluids (like in other areas of intensive care) may help decrease fluid overload, particularly in smaller children and smaller burns

**Conclusions:** Understanding of the contribution of maintenance to the total fluid requirements in children is important for accurate fluid management.

**Fig. 1 (abstract P137).**
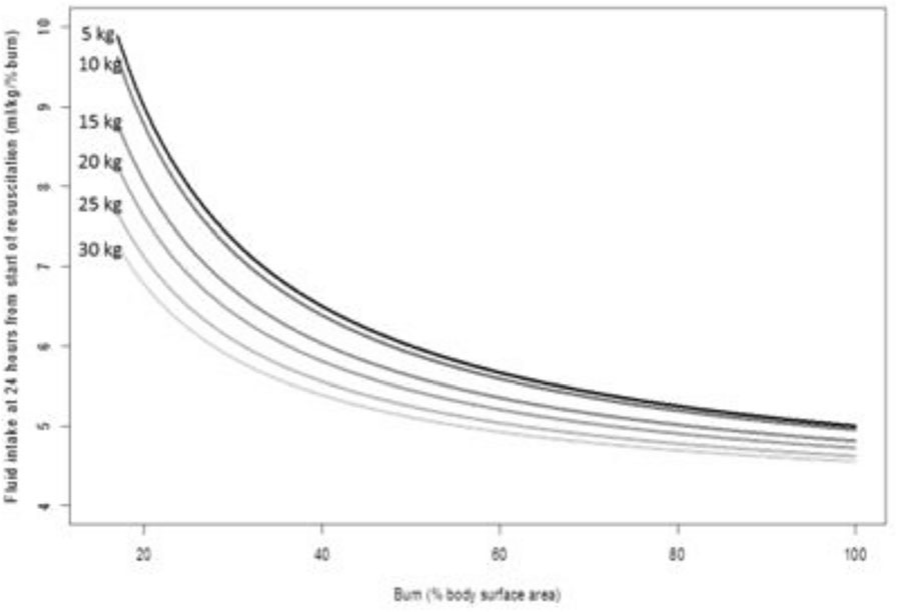
Total fluid requirements for different Burn sizes

**Fig. 2 (abstract P137).**
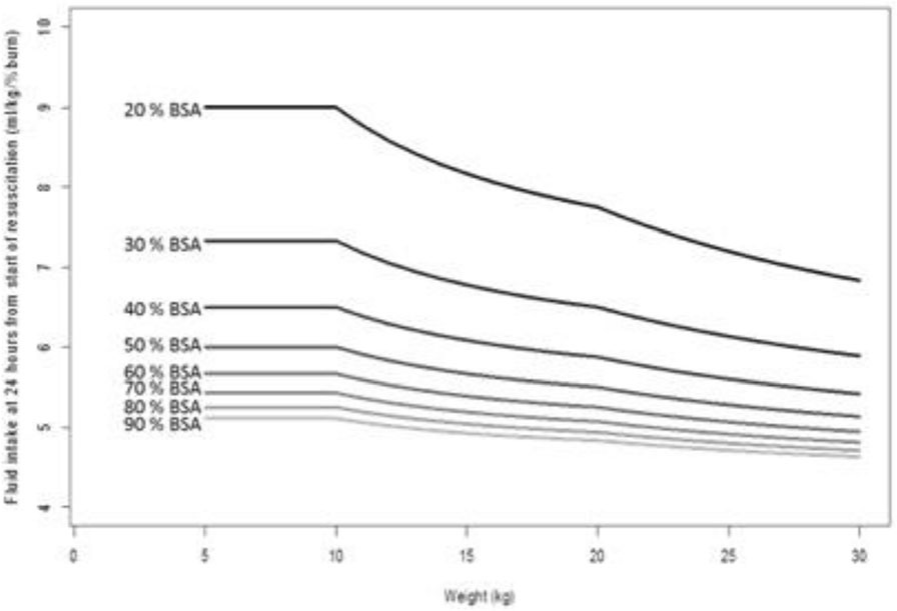
Total fluid requirements for different weights

### P138 Quantification of fluid needed for burns resuscitation in children

#### H Bangalore^1^, S Ray^2^, N Martin^3^

##### ^1^Great Ormond Street Hospital, Burns Intensive Care, London, United Kingdom; ^2^Great Ormond Street Hospital, Paediatric Intensive Care Uniti, London, United Kingdom; ^3^St Andrews Burns Center, Burns and reconstructive surgery, Chelmsford, United Kingdom

**Introduction:** Accurate quantification of fluid in resuscitation of thermal injuries is important for benchmarking, comparing and improving outcomes. In adults, it is usually expressed as mls/kg/%TBSA. In children, maintenance fluids are added to the resuscitation requirements. This is kept constant and the resuscitation component is titrated to meet pre-defined end points—usually urine output. Maintenance fluids are not uniformly stratified across the weight ranges. We propose that quantification of fluids in mls/kg/%TBSA in children does not accurately capture fluid needs for resuscitation due to the maintenance component of the fluid requirement.

**Methods:** We conducted this retrospective study in children admitted to a single-center Burns Intensive Care Unit (BICU) between January 2010 and December 2014. Children ≤30 kilograms with TBSA ≥15% admitted within 8 hours of their injury were included. OE (Observed to expected ratio) and fluid in mls/kg/% TBSA were calculated as shown in Figure 1.

**Results:** There were 33 children in the cohort with half requiring invasive mechanical ventilation in the BITU and nearly a quarter requiring inotropic support. The demographic details are as shown in Table 1. The OE ratio at the end of 24 hours in the cohort was 1.01 (0.68-1.36). The total fluid given was 6.9 (6.1, 8) mls/kg/ % TBSA. The Titrated resuscitation component was 4.2 (3.7, 5.6) mls/kg/TBSA. Total fluid (which included the maintenance fluid) had a poor correlation with OE ratio R2=0.34 (Fig 2). Exclusion of the maintenance fluid had a better correlation with the OE ratio R2=0.75

**Conclusions:** To capture differences in the titratable resuscitation component rather than differences in the maintenance requirements, fluid should be quantified in children by excluding the maintenance component when expressed as mls/kg/%TBSA.

**Table 1 (abstract P138).** Patient details

Age (Years)	2.2 (1.4, 3.2)
Weight (Kilograms)	13.8 (12, 15.3)
TBSA (%)	27 (17-90)
Hours at admission	6 (5,8)
Scald [N(%)]	26 (78%)
Flame [N(%)]	7 (21%)
LOS/%Burns	0.52 (0.36, 0.80)

**Fig. 1 (abstract P138).**
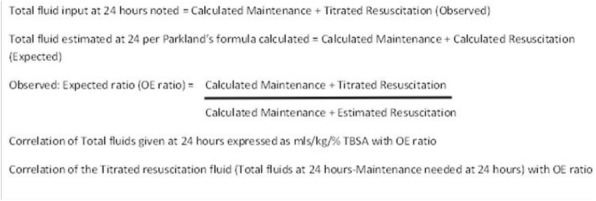
Fluid Calculations

**Fig. 2 (abstract P138).**
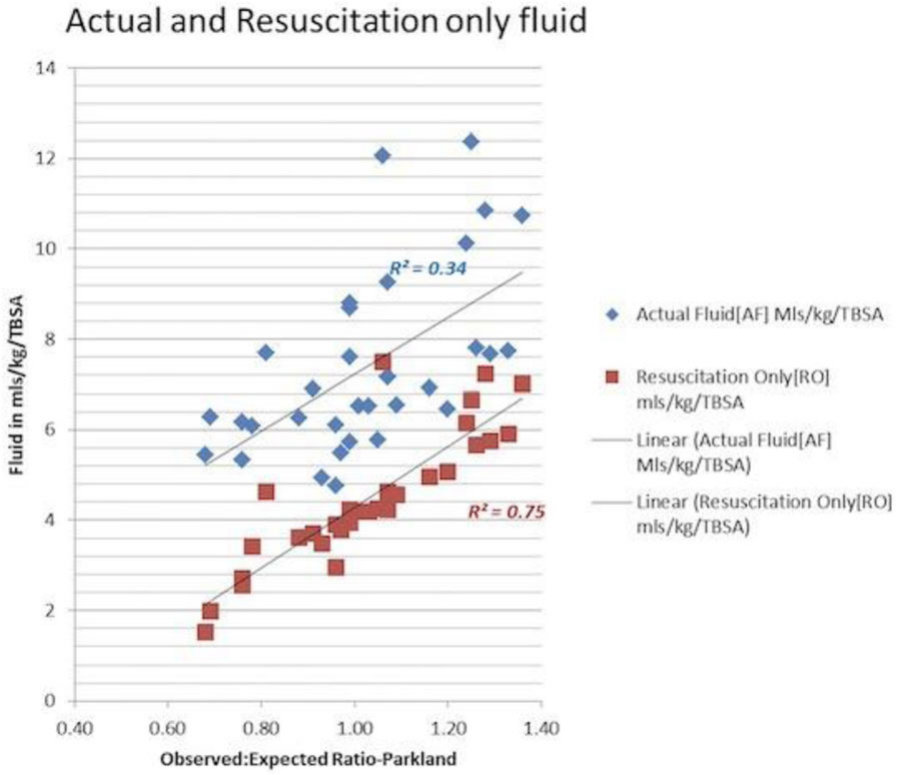
Correlation of OE ratio and fluids-total & titrated resuscitation component

### P139 Dynamic arterial elastance for predicting mean arterial pressure responsiveness after fluid challenges in acute respiratory distress syndrome patients

#### P Luetrakool^1^, S Morakul^1^, V Tangsujaritvijit^2^, C Pisitsak^1^

##### ^1^Ramathibodi, Department of anesthesiology, Bangkok, Thailand; ^2^Ramathibodi, Department of medicine, Bangkok, Thailand

**Introduction:** Dynamic arterial elastance (Eadyn; pulse pressure variation/stroke volume variation; PPV/SVV) is a dynamic parameter of arterial load that can be continuously monitored. Previous study proposed that Eadyn was able to predict mean arterial pressure (MAP) responsiveness after fluid challenge [1-5]. The objective of this study was to assess whether the Eadyn was able to predict MAP responsiveness in acute respiratory distress syndrome (ARDS) patients ventilated with low tidal volume.

**Methods:** We performed a prospective study of diagnostic test accuracy in adult ARDS patients with acute circulatory failure and fluid responsiveness. All patients are continuously monitored blood pressure via arterial line connected with Flotrac® transducer and Vigileo® monitor. Once the attending physicians decided to load intravenous fluid, we recorded PPV/SVV and also other hemodynamic parameters before and after fluid bolus. MAP responsiveness was defined as an increase in MAP ≥ 10% from baseline after fluid challenge.

**Results:** Twenty-three events were included. Nine events (39.13%) were MAP-responsive. Cardiac output, heart rate and stroke volume were similar in both MAP-responder and MAP-nonresponder group. Baseline MAP, diastolic blood pressure (DBP) and pulse pressure (PP) were significantly different after fluid challenge in MAP-responder group. Eadyn of preinfusion phase was failed to predict MAP responsiveness after fluid challenge (area under the curve = 0.603, 95%CI = 0.38-0.798).

**Conclusions:** One of the arterial load parameters such as Eadyn derived from non-calibrated pulse contour analysis method was unable to predict MAP responsiveness in ARDS patients with low tidal volume ventilation.

**References**

1. Monge Garcia MI, et al. Crit Care. 2011;15(1):R15.

2. Garcia MI et al. Crit Care. 2014;18(6):626.

3. Cecconi M et al. Anesth Analg. 2015;120(1):76-84.

4. Lanchon R et al. Anaesth Crit Care Pain Med. 2017;36(6):377-82.

5. Wu CYet al. Eur J Anaesthesiol. 2016;33(9):645-52.

### P140 The change of central venous pressure and mean systemic filling pressure during a fluid challenge in patients with septic shock

#### I Cisterna, R Barelli, T Taccheri, A Dell´Anna, M Antonelli

##### Catholic University of the Sacred Heart, Rome, Italy

**Introduction:** Fluids administration remains a cornerstone treatment for patient in shock state. Increase in Mean Systemic Filling Pressure (Pmsf) with minimal variation of Central Venous Pressure (CVP), should follow volume load in fluid responders (FR). Our aim is to test the hypothesis that in FR septic shock patients, fluid load will determine a significant increase in Pmsf but not in CVP.

**Methods:** We prospectively included all mechanically ventilated patients with diagnosis of septic shock with invasive hemodynamic monitoring (transpulmonary thermodilution VolumeView-EV1000 Edwards©). We collected hemodynamic and metabolic data and Pmsf with the inspiratory holds technique, before and after a fluid challenge (FC) of 500 ml of ringer lactate in 10 minutes). FR was defined as an increase in cardiac output (CO)>15%.

**Results:** 13 measures were obtained in 11 patients. In 8 case we observed FR. We found a significant increase in Pmsf after a FC (mean difference(md) 16.9±21.5 mmHg, p=.015). CVP increased significantly (md 2.5±2.4 mmHg, p=.002). Pmsf increased significantly in non-FR (md 27±10mmHg, p=.013) but not in FR while CVP was higher after FC only in FR (md 2.4±2.1mmHg, p=.008). Venous return gradient (Pmsf-CVP) globally increased after FC (md 14±22 mmHg, p=.03), but only in non-FR such increase was significant (md 24±12 mmHg, p=.03). No correlation was found between the variation CO and venous return gradient. We did not find any improvement in metabolic parameters after the fluid challenge.

**Conclusions:** Pmsf and combined CVP variations do not correlate with FR in our cohort of septic shock patients. Inspiratory holds may not be adequate to infer Pmsf in such context. Further studies are warranted to investigate the effect of FC on Pmsf in this field.

### P141 Evaluation of pre-load dependence over time in patients with septic shock

#### I Douglas^1^, P Alapat^2^, K Corl^3^, M Exline^4^, L Forni^5^, A Holder^6^, D Kaufman^7^, A Khan^8^, M Levy^3^, G Martin^9^, J Sahatjian^10^, W Self^9^, E Seeley^9^, J Weingarten^9^, M Williams^9^, C Winterbottom^11^, D Hansell^12^

##### ^1^Denver Health Medical Center, Denver, Colorado, United States; ^2^Ben Taub Hospital, Houston, United States; ^3^Rhode Island Hospital, Providence, United States; ^4^Ohio State University, Columbus, United States; ^5^Royal Surrey Hospital, Guilford, United States; ^6^Emory University, Atlanta, United States; ^7^NYU Langone Medical Center, New York, United States; ^8^Oregon Health and Sciences University, Portland, United States; ^9^Indiana University, Indianapolis, United States; ^10^Cheetah Medical, Newton Center, United States; ^11^Bridgeport Hospital, Bridgeport, United States; ^12^Massachusetts General Hospital, Boston, United States

**Introduction:** Cardiac function is known to be negatively impacted by sepsis. Stroke volume (SV) change in response to Passive Leg Raise (PLR) is an effective method to predict fluid responsiveness (FR) or cardiac response to preload expansion. We have previously shown that fluid responsiveness is a dynamic state, changing frequently over a 72 hour monitoring period.

**Methods:** FRESH is a currently enrolling prospective randomized controlled study, evaluating the incidence of FR and patient centered outcomes in critically ill patients with sepsis or septic shock (NCT02837731). Patients randomized to PLR guided resuscitation were evaluated every 6-12 hours over the first 72 hours of care and classified as FR if the SV increased > 10% when measured with non-invasive bioreactance (Starling SV, Cheetah Medical). The time of first FR was noted.

**Results:** A total of 608 PLR assessments were performed in 86 patients over a 72 hour monitoring period. 56 % were female, and the average age was 61 years. PLRs were evaluated over time, with time 0 representing initial fluid resuscitation (Figure 1). When individual subjects were evaluated over time, 100% of subjects who became FR only after 24 hours showed evidence of LV/RV dysfunction (Figure 2).

**Conclusions:** Fluid responsiveness or preload dependence frequently changes for septic shock patients over the first 72 hours of care. Evidence suggests it is beneficial to periodically perform an assessment of preload responsiveness to guide fluid administration, as preload dependence is a dynamic and changing state. Preload dependence provides additional information beyond fluid responsiveness. Those patients who remain primarily fluid non-responsive (preload independent) are more likely to demonstrate ECHO confirmed LV/RV dysfunction, as the delay in return to cardiac function may be related to underlying cardiac deficits. Further evaluation may be indicated in preload independent patients.

**Fig. 1 (abstract P141).**
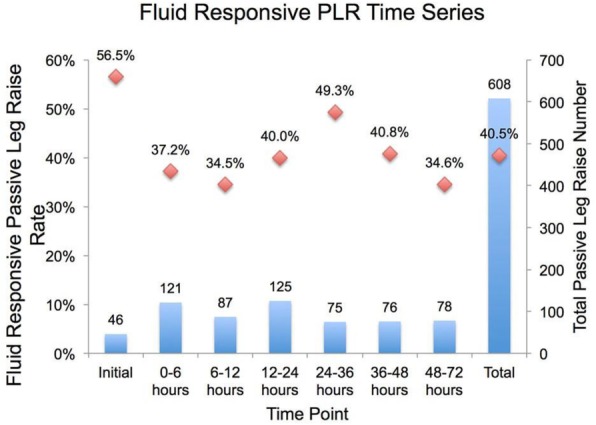
Fluid Responsive PLR Time Series

**Fig. 2 (abstract P141).**
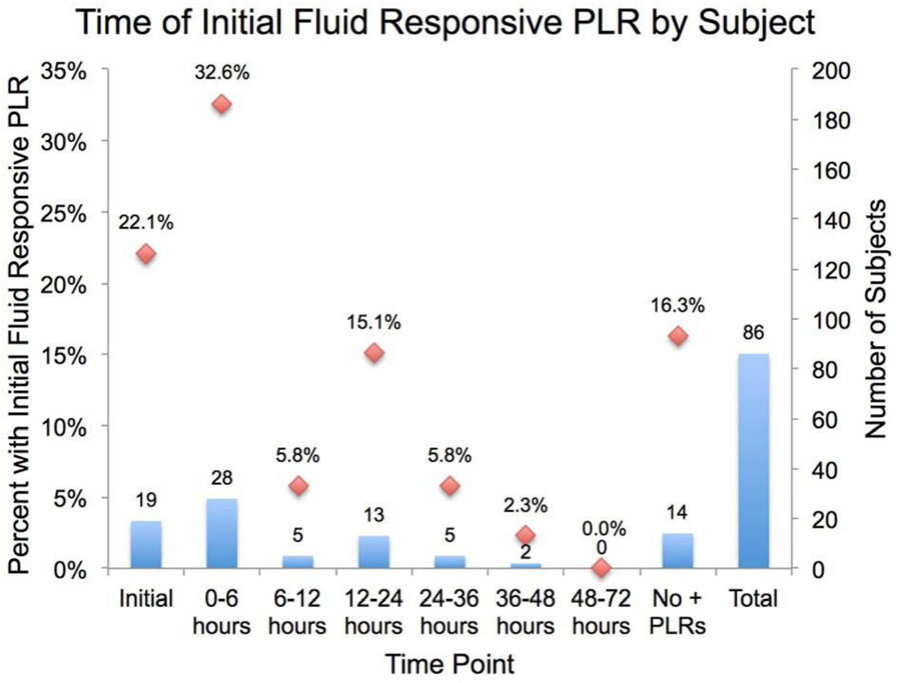
Time of Initial Fluid Responsive PLR by Subject

### P142 Early 20% vs 5 % albumin in addition to crystalloid resuscitation in surgical septic shock admitting to the general SICU

#### S Kongsayreepong, S Nonthiaraj

##### Siriaj Hospital, Mahidol University, Anesthesiology, Bangkok, Thailand

**Introduction:** Fluid resuscitation is one the main management in patients with septic shock. Large amount of fluid may be needed in surgical septic shock patients. The aim of this study was to compare the effect of 20% vs 5% albumin in addition to crystalloid solution in the early phase of septic shock resuscitation in surgical septic shock patient undergoing major surgery for source control on perioperative outcome.

**Methods:** This prospective observation study was done in consecutive surgical septic shock patients (age > 18 yrs) undergoing surgery for source control who received only crystalloid solution, 5% albumin or 20 % albumin in addition in crystalloid solution in the early period of shock resuscitation (12 hours after shock detection) and admitting to the general surgical ICU during October 2016-September 2018. Study data included patient demographic data, co-morbidities, ASA physical status, diagnosis, type and duration of operation, Type & amount of fluid used, fluid intake/output in the first 72 hrs, time to reverse shock, incidence of early postoperative complications (AKI, ARDS, intraabdominal hypertension), shock reversal time, ICU & hospital LOS), 28 & 90 day mortality

**Results:** There were no statistical significant different in the demographic data & comorbidity in the 3 groups (180 patients). Significant more patients in the 2016 received only crystalloid resuscitation. No allergy was found in the albumin groups. Patients who received co-administration of albumin solution had significant less shock reversal time, fluid balance, incidence of AKI, ARDS, intraabdominal hypertension, ICU length of stay & 28 days mortality. Patient who receive 20% albumin had significant less shock reversal time, fluid balanced, abdominal hypertension & ICU length of stay.

**Conclusions:** Hyperoncotic 20% albumin could be used as a safe additional resuscitation fluid to crystalloid solution in surgical septic shock patient with less shock reversal time & less complications from positive fluid balance.

### P143 Lack of association between low cumulative dose of hydroxyethyl starch and acute kidney injury in patients with acute ischemic stroke

#### SH Park, TJ Kim, HB Jeong, SB Ko

##### Seoul National University Hospital, Department of Neurology and Critical Care, Seoul, South Korea

**Introduction:** Hydroxyethyl Starch (HES), a synthetic colloid, has been used as a volume expander, and is associated with renal impairment in patients with sepsis. However, a small dose of HES (6%, 130/0.4) has sometimes been used in acute ischemic stroke. Therefore, we investigated whether a small dose of HES was linked with renal deterioration in patients with acute ischemic stroke.

**Methods:** A consecutive 524 patients with acute ischemic stroke within 7 days from onset were included between January 2012 and May 2016 (Fig 1). We collected admission serum creatinine (SCr), estimated glomerular filtration rate (eGFR), and renal function was assessed using KDIGO definition of acute kidney injury on hospital days 5 to 9 as to patient’s hospitalization period. Proportion of patients with good functional outcome [(mRS) 0-2] at 90 days were compared between HES group and controls.

**Results:** Among the included patients (mean age, 68.6; male, 56.5 %), 81 patients (15.5 %) were treated with HES (median cumulative dose, 1450 mL). Initial SCr was lower (0.87 ± 0.43 vs. 1.15 ± 1.15, P < 0.001) and initial eGFR was higher (86.91 ± 24.27 vs. 74.55± 29.58, P < 0.001) in HES group compared with controls (Table 1, 2). The rate of acute kidney injury (AKI) was not different between HES group and control (4.9 % vs. 8.1 %, P = 0.320) (Fig 2). Moreover, use of HES decreased followed-up SCr (0.81 ± 0.47, P= 0.001) and increased followed-up eGFR (97.02 ± 27.90, P < 0.001) similar to control group (SCr, 1.05 ± 0.97, P = 0.001; eGFR, 80.35 ± 32.91, P < 0.001). HES treatment did not lead to better functional outcome after 3 months of acute ischemic stroke (58.5% vs.64.3%, P = 0.279).

**Conclusions:** HES did not negatively affect renal outcome in patients with acute ischemic stroke if HES was used with a small dose.

**References**

1. Thanvi B et al. Postgrad Med J. 2008; 84:412-7.

2. van der Jagt M. Crit Care. 2016 May 31;20(1):126

**Table 1 (abstract P143).** Baseline characteristics of study population

Characteristic	Total (n =524)	HES Yes (n=81, 15.5 %)	HES No (n=443, 84.5 %)	P value
Age, years (mean ± SD)	68.6 ± 13.0	64.8 ± 14.7	69.3 ± 12.5	0.004
Gender (male), n (%)	296 (56.5 %)	42 (51.9 %)	254 (57.3 %)	0.360
Initial NIHSS*, median (IQR)	4 (2-9)	5 (3-9)	3 (1-9)	0.03
Discharge NIHSS*, median (IQR)	2 (0-5)	4 (1-7)	2 (0-4)	0.008
Previous stroke history, n (%)	115 (21.9 %)	13 (16.0 %)	102 (23.0 %)	0.163
Body mass index, m2	23.3 ± 3.3	23.5 ± 3.0	23.3 ± 3.3	0.457
Total Fluid balance(L/hospital day)	3.67 ± 2.95	4.21 ± 2.99	3.57 ± 2.92	0.072

**Table 2 (abstract P143).** Differentiation of initial and follow up laboratory finding

Values	Total (n =524)	HES Yes (n=81, 15.5 %)	HES No (n=443, 84.5 %)	P value
Initial SCr (mg/dL), mean ± SD	1.11 ± 1.07	0.87 ± 0.43	1.15 ± 1.15	<0.001
F/u SCr (mg/dL), mean ± SD	1.01 ± 0.91	0.81 ± 0.47	1.05 ± 0.97	0.001
Initial eGFR (mL/min/1.73 m2), mean ± SD	76.46 ± 29.15	86.91 ± 24.27	74.55 ± 29.58	<0.001
F/u eGFR (mL/min/1.73 m2), mean ± SD	82.93 ± 32.73	97.02 ± 27.90	80.35 ± 32.91	< 0.001
Increased SCr (mg/dL), n (%)	163 (31.1 %)	24 (29.6 %)	139 (31.4 %)	0.755
Increased SCr (> 0.3) (mg/dL), n (%)	40 (7.6 %)	4 (4.9 %)	36 (8.1 %)	0.320

**Fig. 1 (abstract P143).**
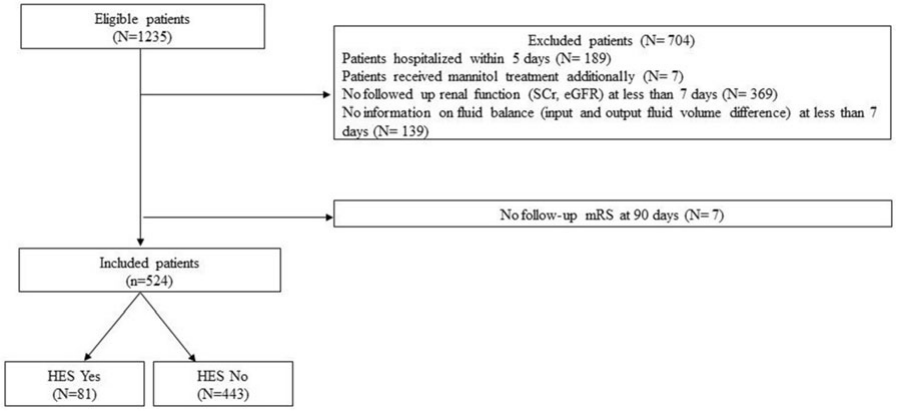
Flow diagram of patients selection

**Fig. 2 (abstract P143).**
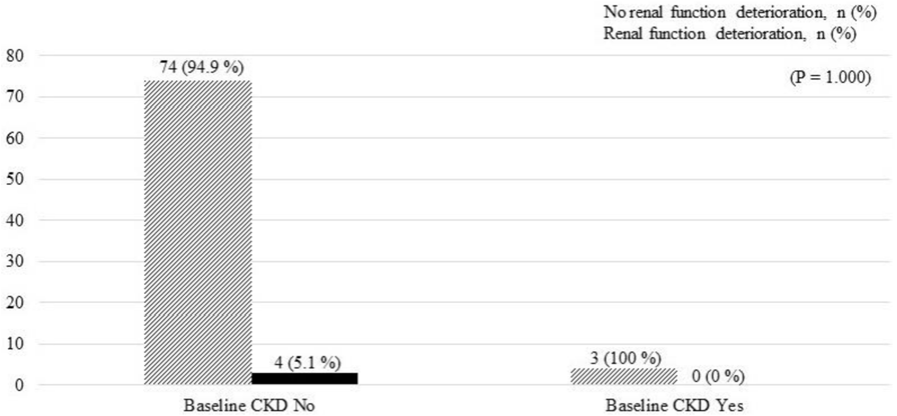
Baseline renal function and alteration of renal function in HES treatment (N= 81)

### P144 Dynamic in vivo assessment of mean systemic filling pressure: clinical application

#### F Policastro^1^, M Pelli^1^, RA De Blasi^2^

##### ^1^Sant´Andrea Hospital - Sapienza University of Rome, Scuola di Specializzazione in Anestesia e Rianimazione, Roma, Italy; ^2^Sant´Andrea Hospital - Sapienza University of Rome, Dipartimento di Scienze Medico-Chirurgiche e Medicina Traslazionale/Unità di Terapia Intensiva, Roma, Italy

**Introduction:** Venous return and systemic perfusion are determined by pressure and volumes within the Venular Network. Mean Systemic Filling Pressure (MSFP) is crucial for venous return and volaemic status, and as such it is a useful parameter in physiology and clinical settings alike. We tested whether: Near Infra-Red Spectroscopy (NIRS) could be effective at measuring MSFP both in healthy individuals and in conditions with a rise in interstitial pressures; after an occlusion pressure is relieved, the decrease in venular blood volume could allow calculation of τ (time constant) and thus Venous Resistances (Rv). In order to verify these hypotheses we used a forearm NIRS probe on healthy individuals at rest and during different degrees of Maximal Voluntary Contraction (MVC).

**Methods:** 10 healthy subjects volunteered in the study that took place at Sant’Andrea Hospital in Rome (Italy). All subjects had venular pressures and volumes assessed via a NIRS probe positioned on the forearm using a pressure-cuff in steps of 5 mmHg from 0 to 50 mmHg, at rest and at 10% and 20% MVC. For each patient MSFP, unstressed Volume (Vu) and stressed Volume (Vs) were measured. A temporary 30 mmHg occlusion was obtained and volume time course was calculated upon release, to derive τ .

**Results:** P-V relationship was found to have a 3-slopes shape reflecting venular network changes. We measured Vu, Vs, and obtained MSFP values of 6.74 ± 1.83 mmHg, p<0.001; during exercise no changes in Vu and Vs were noted but MSFP values rose; value was found to be 2.9 ± 0.1 sec at rest and 0.9 ± 0.03 sec after exercise, reflecting a reduction in Rv.

**Conclusions:** NIRS measurements on healthy subject may have implications in the clinical assessment of critical care patients where changes in interstitial pressure are possible.

**Reference**

1. De Blasi RA et al. Microcirculation 21:606-614, 2014

### P145 Diagnosis of hepatic arterial flow disorders in patients after abdominal surgery with development of multiple organ dysfunction syndrome

#### SA Tachyla^1^, AL Lipnitski^1^, AV Marachkou^1^, VA Livinskaya^2^

##### ^1^Mogilev Regional Hospital, Department of Anesthesiology and Intensive Care, Mogilev, Belarus; ^2^Belarusian-Russian University, Department “Economics and Management”, Mogilev, Belarus

**Introduction:** In the pathogenesis of multiple organ dysfunction syndrome (MODS) important role plays the development of hepatic dysfunction. A known method for assessing hepatic blood flow is reohepatography (RHG). However, it requires the analysis of a large number of parameters of the rheogram curve.

The aim of this study was to develop a method for assessing arterial hepatic blood flow based on the RHG in patients with MODS after abdominal surgery.

**Methods:** 55 patients in the department of anesthesiology and intensive care unit were included in a prospective study (36 men and 19 women, age 60.1 ± 16.1 years, weight 78.5 ± 13.9 kg.). All patients were divided into two groups: group 1 - patients after orthopedic and trauma surgery (n = 28), group 2 - patients after abdominal surgery with MODS (n = 27). Patients in the groups did not have statistical differences by sex, age, body weight, height. RHG was carried out using the “Reo-Spectr” (Russian Federation).

**Results:** We have compared the RHG indicators between the groups (Table 1). We have developed a method for assessing hepatic arterial blood flow, which consists in determining the area under the arterial part of RHG curve using the Simpson’s rule. Its normal values range from 43.2 mΩ *s to 49.0 mΩ *s. The method is non-invasive, can be applied at the patient´s bed. Its advantage is simplicity, it can be used for rapid diagnosis and monitoring the effectiveness of treatment. Area under the RHG curve in the group 1 were 46.1 ± 8.1 mΩ *s and 20.0 ± 16.8 mΩ *s in the group 2 (p <0.05).

**Conclusions:** Patients after abdominal surgery with MODS have impaired hepatic blood flow, which may be associated with liver pathology caused by main surgical disease (obstructive jaundice) and hemodynamic disorders caused by acute cardiovascular failure. The method we developed allows us to determine disorders of hepatic arterial blood flow in the early stages before signs of liver dysfunction appear.

**Table 1 (abstract P145).** Comparison of RHG indicators between groups, * – p<0.05

RHG indicators	Group 1	Group 2
fast blood filling time, s	0.08±0.02	0.06±0.02*
time of slow blood filling, s	0.13±0.02	0.09±0.03*
time of the ascending part of the wave, s	0.2±0.03	0.15±0.04*
total systole time, s	0.46±0.03	0.36±0.13*
time of the catacrots, s	0.72±0.04	0.57±0.21*
rheographic index, units	1.09±0.39	0.63±0.47*
maximum speed of fast filling, Ω /s	1.1±0.18	0.62±0.37*

### P146 Comparison of pulse oximetry hemoglobin with laboratory measurement of arterial and central-venous hemoglobin: the prospective CLIMATE-II study

#### W Huber, T Lahmer, U Mayr, G Batres-Baires, S Rasch, R Schmid, L Offman

##### II. Medizinische Klinik, Station 2/11, Munich, Germany

**Introduction:** Non-invasive pulse oximetry hb-monitoring (SpHb) has been validated predominatly in the peri-operative setting and with controversial results. Monitoring of SpHb in parallel with arterial and central-venous hb-monitoring is part of the CLIMATE-database. The CLIMATE-studies investigate clinical examination, arterial and central-venous blood gas analysis, circulating blood volume, perfusion index, SpHb (Radical-7; MASIMO; USA) and transpulmonary thermodilution. The CLIMATE-II study comapred SpHb with arterial (Hb_art) and central-venous Hb (HB_cv).

**Methods:** A total of 240 datasets were recorded within 24h in 30 patients of a general ICU (8 datasets per patient). Primary endpoint: Accuracy, precision and trending capacities of SpHb compared to Hb_art and Hb_cv (RapidPoint; Siemens; Germany). Statistics: IBM SPSS 25.

**Results:** Age 65+/-12 years; mechanical ventilation 240/240, vasopressors 156/240 (65%) of measurements.

SpHb was poorly correlated with Hb_art (r=0.306; p<0.001) and Hb_cv (r=0.312; p<0.001), while Hb_art and Hb_cv were strongly correlated (r=0.949; p<0.001). SpHb (9.60+/-1.11g/dL) overestimated Hb_art (8.83+/-1.64g/dL; p<0.001) and Hb_cv (9.07+/-1.69g/dL; p<0.001). This resulted in bias, lower (LLOA) and upper (ULOA) limits of agreement of 0.77, -2.17 and 3.71g/dl for SpHb vs. Hb_art and 0.53, -2.47 and 3.53g/dL for SpHb vs. Hb_cv. Percentage error values were 31.9 and 32.1%. Trending analysis (subtraction of two subsequent values) did not demonstrate a correlation of Delta-SpHb with Delta-Hb_art (r=0.31; p=0.654) and Delta-Hb_cv (r=0.094; p=0.171), whereas Delta_Hb_art and Delta-Hb_cv were significantly associated (r=0.543; p<0.001). 4-quadrant-plot concordance for Delta-SpHb was poor vs. Delta_Hb_art (59%) and vs. Delta-Hb_cv (59%).

**Conclusions:** In mechanically ventilated ICU patients SpHb provided a moderate bias, but inacceptable precision and trending compared to Hb_art and Hb_cv.

### P147 Clinical examination, LiMON, MASIMO and thermodilution for haemodynamic monitoring: the prospective CLIMATE-I study

#### W Huber, T Lahmer, G Batres-Baires, U Mayr, S Rasch, R Schmid, L Offman

##### II. Medizinische Klinik, Station 2/11, Munich, Germany

**Introduction:** There is a lack of studies comparing the use clinical examination to apparative haemodynamic monitoring. Therefore, the CLIMATE-studies compared clinical examination including recap-time (RCT), circulating blood volume (CBV; LiMON; Pulsion; Germany), perfusion index (MASIMO; USA) and transpulmonary thermodilution (TPTD; PiCCO; Pulsion; Germany).

**Methods:** The CLIMATE-I study compared PI with clinical findings and parameters of more invasive technologies. Primary endpoint: prediction of a cardiac index (CI) ≤ 2.5mL/min/sqm by non-invasive parameters. A total of 240 datasets were recorded within 24h in 30 patients (8 datasets per patient). Each dataset included clinical examination (RCT), infrared measurements of the surface temperatures on forehead, forearm, finger and great toe (Thermofocus; Technimed; Italy), CBV (only 1st measurement), perfusion index (PI; MASIMO) and data from TPTD. Statistics: IBM SPSS 25.

**Results:** Age: 65+/-12 years; mechanical ventilation 240/240, vasopressors 156/240 (65%) of measurements. In univariate analysis (p<0.001 except as indicated) PI had a stronger association to flow (Spearman-correlations with PI: stroke volume index: r=0.456; cardiac index CI: r=0.294) than to preload (CVP: r=0.214; p=0.001; GEDVI: r=0.254; SVV: r=-0.349). The strongest associations of PI were found with clinical markers as finger tip temperature (FTT; r=0.610) and RCT (r=-0.402). In multivariate analysis (R=0.607), PI was independently associated with FTT (p<0.001; T=7.859) and SVI (p<0.001; T=6.591), but not with CVP, GEDVI, SVV, CI, CBV and RCT.

Among the non-invasive technologies only FTT (ROC-AUC=0.681; p=0.028) and RCT (AUC=0.688; p=0.023) predicted CI ≤ 2.5mL/min/sqm. PI (AUC=0.446; p=0.512) was not predictive.

**Conclusions:** FTT and RCT are strongly associated with PI and predict a CI ≤ 2.5L/min/sqm. PI did not predict a CI ≤2.5L/min/sqm.

### P148 Subjective right ventricle assessment in the critically ill vs objective measures

#### S Orde^1^, M Slama^2^, S Yastrebov^3^, A Mclean^1^, S Huang^1^

##### ^1^Nepean Hospital, Intensive Care Unit, Sydney, NSW, Australia; ^2^Amiens University Hospital, Medical ICU, Amiens, France; ^3^St George Hospital, ICU, Sydney, Australia

**Introduction:** Subjective right ventricle (RV) assessment is common and guidelines suggest its utility in adequately trained clinicians. This has never been assessed in a robust manner. We compared subjective assessment of RV size and function in ICU patients by Intensivists with advanced echo qualifications vs RV free wall strain (RVfwS) assessed by speckle tracking echo, and conventional methods.

**Methods:** 52 Intensivists reviewed 2D loops from 80 critically ill patients. Inclusion criteria: mechanically ventilated with PaO2:FiO2 <300. Exclusion criteria: congenital heart disease, cardiac surgery, unable to perform RVfwS (19 patients, feasibility 80%). Echo measures included RV size: end diastolic area (EDA) and RV dimensions; RV function: fractional area change (FAC), S’, TAPSE, RVfwS. Binary (normal vs abnormal) and ordinal analysis (normal, mild/moderate or severe) was performed for RV size and function.

**Results:** 80 patients: 54% male, median 68years (59-73); P:F ratio 174 (132-208); PEEP 10 (7-12); APACHE III 80.5 (26); median ventilation time 6 days (3-9). Fair agreement was seen in subjective assessment vs objective measures with binary assessment of RV size and function. Ordinal data analysis showed poor agreement with RVfwS (Figure 1) and RV dimensions. If one-step disagreement was allowed the agreement was good (Table 1, 2). Significant overestimation of severity of abnormalities was seen comparing subjective assessment with RV EDA and TAPSE, s’ and FAC. There was no difference in agreement values when accounting for clinician echo experience, perceived expertise (at level of cardiologist) or type of qualifications.

**Conclusions:** Relatively low levels of agreement were seen with subjective assessment vs objective measures of RV size and function assessed by echo. It seems prudent to avoid subjective RV assessment in isolation and a combination of objective and subjective measures should be used.

**Table 1 (abstract P148).** Agreement of RV size assessment

Data type	Parameter	Agreement (B-score)	Weighted (one step disagreement allowed)	Bias (p value)
		Unweighted (exact agreement required)		
Binary	RV end diastolic area	0.26	-	<0.001
	RV dimensions	0.29	-	0.06
Ordinal	RV end diastolic area	0.26	0.62	<0.001
	RV dimensions	0	0.59	0.06

**Table 2 (abstract P148).** Agreement of RV function assessment

Data type	Parameter	Agreement (B-score)	Weighted (one step disagreement allowed)	Bias (p value)
		Unweighted (exact agreement required)		
Binary	RV free wall strain	0.27	-	0.35
	TAPSE	0.27	-	<0.001
	S’	0.29	-	<0.001
	Fractional Area Change	0.31	-	<0.001
Ordinal	RV free wall strain	0.14	0.6	0.16
	TAPSE	0.28	0.64	<0.001

**Fig. 1 (abstract P148).**
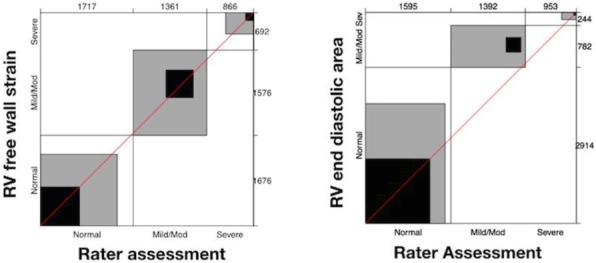
Subjective RV function and size assessment vs objective measures

### P149 Predicting hypotension episode with numerical vital sign signals in the intensive care unit

#### JH Yoon^1^, V Jeanselme^2^, A Dubrawski^2^, MR Pinsky^1^, G Clermont^1^

##### ^1^University of Pittsburgh, Critical Care Medicine, Pittsburgh, United States; ^2^Carnegie Mellon University, Auton Lab, Pittsburgh, United States

**Introduction:** Even short periods of hypotension are associated with increased morbidity and mortality. Using high-density numerical physiologic data, we developed a machine learning (ML) model to predict hypotension episodes, and further characterized risk trajectories leading to hypotension.

**Methods:** A subset of subjects with 1/60Hz physiological data was extracted from MIMIC2, a richly annotated multigranular database. Hypotension was defined as >5 measurements of systolic blood pressure ≤ 90 mmHg and mean arterial pressure ≤ 60 mmHg, within a 10-minute window. Derived features using raw measurements of heart rate, respiratory rate, oxygen saturation, and blood pressure were computed. Random Forest (RF), K-Nearest Neighbors (KNN), and Logistic Regression models were trained with 10-fold cross validation to predict instantaneous risk of hypotension using features extracted from the data leading to the first episode of hypotension (cases) or ICU discharge in subjects never experiencing hypotension (controls). For a given subject, risk trajectory was computed from the collation of instantaneous risks.

**Results:** From a source population of 2808 subjects, 442 subjects met our definition of hypotension, and 724 subjects without hypotension comprised the control group. 204 features were generated from the four vital signs. The area under the curve (AUC) for Random Forest classifier was 0.829, out-performing Logistic Regression (AUC 0.826) or K-Nearest Neighbors (AUC 0.783) (Fig 1). Risk trajectories analysis showed average controls risk scores <0.3 (<30% risk of future hypotension), while the hypotension group had a rising risk score (0.45 to 0.7) in the 8 hours leading to the first hypotension episode, and significantly higher scores leading into subsequent episodes (Fig 2).

**Conclusions:** Hypotension episodes can be predicted from vital sign time series using supervised ML. Subjects developed hypotension have an increased risk compared to controls at least 8 hours prior to the episode.

**Fig. 1 (abstract P149).**
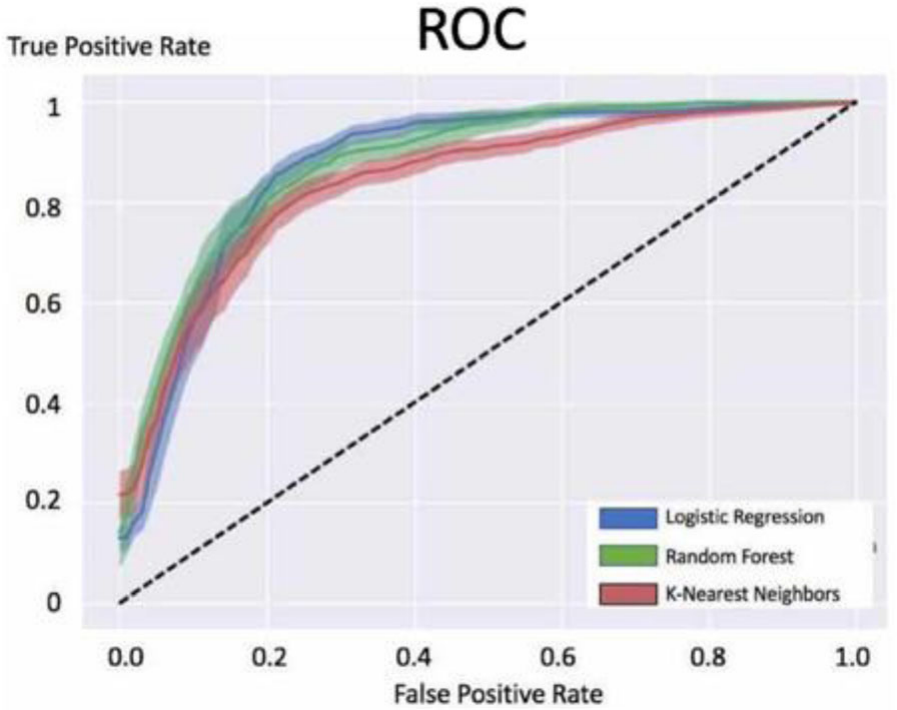
Comparison of performance of three different supervised machine learning algorithm with receiver operating characteristic (ROC) curve

**Fig. 2 (abstract P149).**
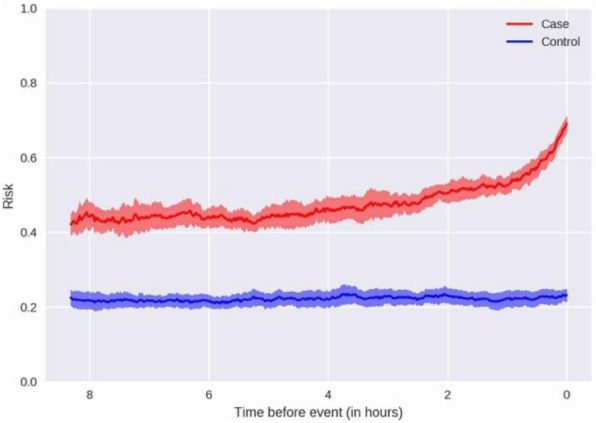
Risk evolution over the last 8 hours before the episode in the ICU. On the x-axis, time 0 is the time for hypotension episode (case: red) or time of discharge or end of monitoring (control: blue)

### P150 Cardiac output estimation using uncalibrated pulse wave analysis (PWA) in postoperative cardiac surgery patients: a method comparison study using pulmonary artery thermodilution as the reference method

#### G Greiwe^1^, K Luehsen^2^, B Saugel^1^

##### ^1^University Medical Centre Hamburg-Eppendorf, Department of Anesthesiology, Hamburg, Germany; ^2^University Medical Centre Hamburg-Eppendorf, Hamburg, Germany

**Introduction:** In critically ill patients or in patients undergoing major surgery, monitoring of CO is recommended[1-3]. Less-invasive advanced hemodynamic monitoring with PWA is increasingly used in perioperative and critical care medicine. In this study, we evaluate the measurement performance of an uncalibrated pulse wave analysis (PWA) device (MostcareUp, Vygon, Ecouen, France) compared with cardiac output (CO) assessment by pulmonary artery thermodilution (PATD) in patients after cardiac surgery.

**Methods:** In patients after cardiac surgery, we performed seven sets of PATD measurements to assess PATD-CO. Simultaneously, we recorded the PWA-CO and compared it to the corresponding PATD-CO. To describe the agreement between PWA-CO and PATD-CO we used Bland-Altman analysis showing the mean of the differences and 95%-limits of agreement and calculated the percentage error.

**Results:** We included 17 patients in the analysis. The bias between PWA-CO and PATD-CO was 0.01 L*min-1. Upper and lower 95% limits of agreement were +1.40 L*min-1 and -1.38 L*min-1. The percentage error was 28.1%.

**Conclusions:** PWA-CO estimated with using the MostcareUp device shows good agreement with pulmonary artery thermodilution-derived CO in patients after cardiac surgery.

**References**

1. Cecconi et al. (2014) Consensus on circulatory shock and hemodynamic monitoring. Task force of the European Society of Intensive Care Medicine.

2. Saugel B, Vincent JL (2018) Cardiac output monitoring: how to choose the optimal method for the individual patient.

3. Vincent JL et al. (2015) Perioperative cardiovascular monitoring of high-risk patients: a consensus of 12.

### P151 Non-invasive monitoring using photoplethysmography technology

#### K Tomita^1^, T Nakada^1^, T Oshima^1^, T Oami^1^, T Aizimu^2^, T Aizimu^2^, S Oda^1^

##### ^1^Chiba University Graduate School of Medicine, Emergency and Critical Care Medicine, Chiba, Japan; ^2^Chiba University, Center for Frontier Medical Engineering, Chiba, Japan

**Introduction:** Non-invasive continuous blood pressure monitoring devices have been investigated, however, these devices did not have sufficient accuracy and precision. We developed a continuous monitor using the photoplethysmographic technique and tested the accuracy and precision of this system to ensure it was comparable to conventional continuous monitoring methods used for critically ill patients.

**Methods:** The study device was developed to measure blood pressure, pulse rate, respiratory rate, and oxygen saturation, continuously with a single sensor using the photoplethysmographic technique. Patients who were monitored with arterial pressure lines in the ICU were enrolled. The physiological parameters were measured continuously for 30 minutes at 5-minute intervals using the study device and the conventional methods. The primary outcome variable was blood pressure.

**Results:** Pearson fs correlation coefficient between the conventional method and photoplethysmography device were 0.993 for systolic blood pressure, 0.985 for diastolic blood pressure, 0.998 for mean blood pressure, 0.996 for pulse rate, 0.995 for respiratory rate, and 0.963 for oxygen saturation. Percent errors for systolic, diastolic and mean blood pressures were 2.4% and 6.7% and 6.5%, respectively. Percent errors for pulse rate, respiratory rate and oxygen saturation were 3.4%, 5.6% and 1.4%, respectively.

**Conclusions:** The non-invasive, continuous, multi-parameter monitoring device presented high level of agreement with the invasive arterial blood pressure monitoring, along with sufficient accuracy and precision in the measurements of pulse rate, respiratory rate, and oxygen saturation.

### P152 Trending, accuracy and precision of stroke volume measurement from noninvasive bioreactance and pulse wave transit time compared with oesophageal Doppler

#### C Pisitsak

##### Ramathibodi Hospital, Department of Anesthesiology, Bangkok, Thailand

**Introduction:** Evidences have shown that perioperative goal directed hemodynamic management using oesophageal Doppler monitoring (ODM) can improve patient outcomes. The ODM is considered minimal invasive device. The authors compared the trending, accuracy and precision of non-invasive stroke volume measurement based on bioreactance technique and pulse wave transit time (PWTT) with ODM.

**Methods:** Ten patients who underwent abdominal surgery under general anesthesia were included for repetitive measurement of stroke volume on five minutes interval after anesthesia. The ODM, bioreactance and PWTT based stroke volume measurement were monitored in each patient. The correlation of stroke volume between bioreactance and PWTT with ODM were analyzed using Pearson correlation coefficient. The Bland and Altman analysis was performed to evaluate the accuracy and precision of PWTT and bioreactance against ODM.

**Results:** The total data sets of 227 were included for analysis. The results demonstrated the correlation coefficient of 0.75 (p<0.001, 95% CI 0.62-0.78), a bias of 0.28 ml (limits of agreement of -30.92 - 31.38 ml) and the percentage error of 46.72% between ODM and bioreactance (Fig 1) and the correlation coefficient of 0.48 (p<0.001, 95%CI 0.44-0.72), a bias of -0.18 ml (limits of agreement of -40.28 - 39.92 ml) and the percentage error of 60.25% between ODM and PWTT (Fig 2).

**Conclusions:** Stroke volume measurement using bioreactance technique had strong correlation with ODM while PWTT had moderate correlation. Both devices had small bias with wide limits of agreement and percentage error compared with ODM. Therefore, these devices are not interchangeable with ODM. However, using trends in stroke volume to guide treatment might still be acceptable.

**Reference**

1. Abbas SM, Hill AG. Anaesthesia 2008;63(1):44–51.

**Fig. 1 (abstract P152).**
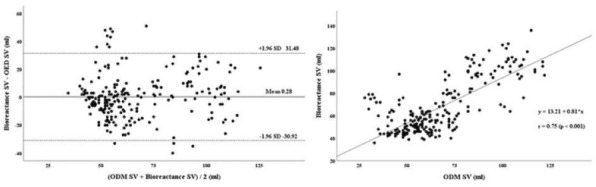
Comparison between oesophageal Doppler and bioreactance technique for stroke volume measurement, Bland and Altman Plot (left panel), regression line and data (right panel). SV = stroke volume, OED = oesophageal Doppler

**Fig. 2 (abstract P152).**
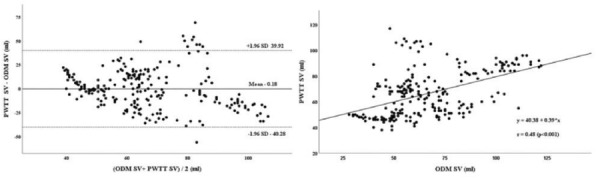
Comparison between oesophageal Doppler and pulse wave transit time for stroke volume measurement, Bland and Altman Plot (left panel), regression line and data (right panel). SV = stroke volume, OED = oesophageal Doppler, PWTT = pulse wave transit time

### P153 Hemorrhage diagnosis using sequential deep learning models of waveform vital sign data

#### F Falck^1^, MR Pinsky^2^, G Clermont^2^, A Dubrawski^1^

##### ^1^Carnegie Mellon University, Auton Lab, School of Computer Science, Pittsburgh, United States; ^2^University of Pittsburgh, Department of Critical Care Medicine, School of Medicine, Pittsburgh, United States

**Introduction:** Hemorrhage is the most common cause of trauma deaths and the most frequent complication of major surgery. It is difficult to identify until profound blood loss has already occurred. We aim at detecting hemorrhage early and reliably using waveform vital sign data routinely collected before, during, and after surgery.

**Methods:** We use waveform vital sign data collected at 250 Hz during a controlled transition from a stable (non-bleeding) to a fixed bleeding state of 93 pigs. These vital signs include airway, arterial, central venous and pulmonary arterial pressures, venous oxygen saturation (SvO2), pulse oximetry pleth and ECG heartrate, continuous CO, and stroke volume variation (LiDCO). We used Gated Recurrent Units (GRU), Long Short-Term Memory (LSTM) and dilated, causal, one-dimensional convolutional neural networks presenting time windows drawn from the raw vital signs as inputs during training.

**Results:** Our GRU model reaches Area Under ROC (AUC) at a clinically relevant False Positive Rate (FPR) <1% of 0.7469 and True Positive Rate of 0.5639, thus achieving an operationally relevant performance for a real-world clinical application (Table 1). However, outside of the very low FPR range (cf. ROCs in Fig. 1 and 2), our models appear inferior to a referenced Random Forest (RF) classifier.

**Conclusions:** Our work demonstrates the applicability of deep learning models to diagnose hemorrhage based on raw, waveform vital signs. Future work will address why the RF classifier can address the greater homogeneity of subjects when they bleed compared to an apparently wide dispersion of their statuses when being stable.

**Reference**

This work is partially supported by NIH GM117622.

**Table 1 (abstract P153).** Experimental results (superslow bleeding subjects)

Model	AUC	AUC@FPR<1%	TPR@FPR=0.1%	TNR@FNR=1%
Support Vector Machine	0.8936	0.5306	0.0132 ± 0.0012	0.0631 ± 0.0038
Logistic Regression	0.8445	0.5132	0.0062 ± 0.0014	0.0484 ± 0.0083
Naive Recurrent Neural Network (nRNN)	0.9015	0.6077	0.0439 ± 0.2558	0.0583 ± 0.2875
RF on statistical features (baseline)	0.9705	0.6386	0.1456 ± 0.3242	0.6386 ± 0.1972
Long Short-Term Memory (LSTM)	0.9263	0.7010	0.3289 ± 0.1357	0.0981 ± 0.3140
Gated Recurrent Unit (GRU)	0.9449	0.7469	0.3832 ± 0.2267	0.2227 ± 0.2881
Dilated, causal convolution	0.9360	0.5390	0.0163 ± 0.3763	0.1564 ± 0.1991

**Fig. 1 (abstract P153).**
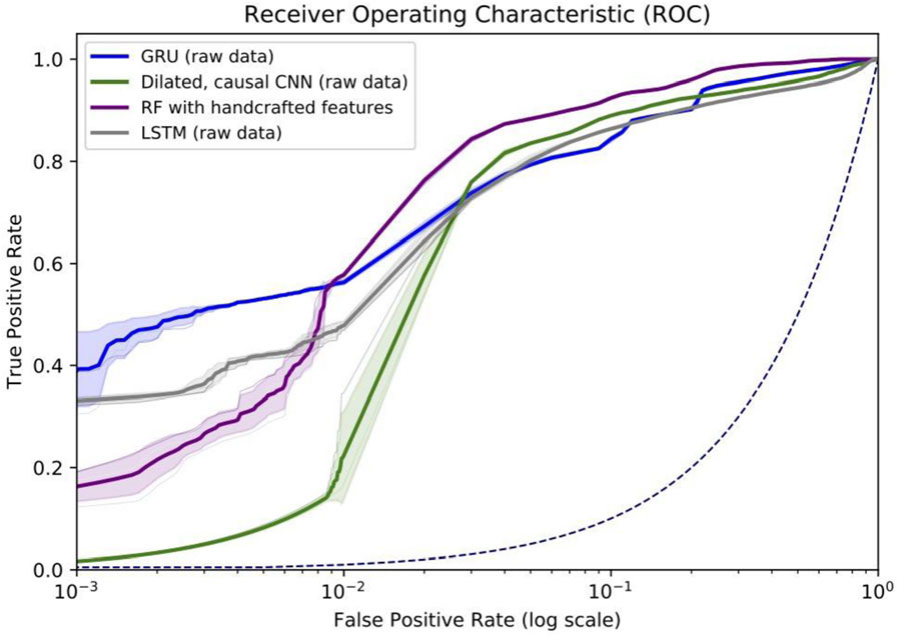
ROC (TPR vs. FPR)

**Fig. 2 (abstract P153).**
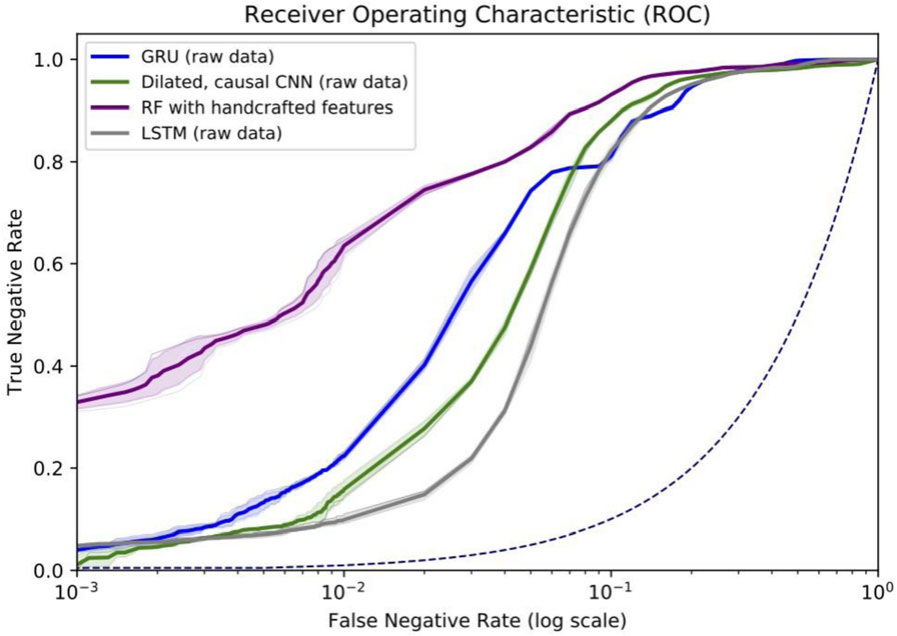
ROC (TNR vs. FNR)

### P154 Can myocardial perfusion imaging with echo contrast help recognise type 1 acute myocardial infarction in the critically ill?

#### S Orde^1^, M Slama^2^, F Pathan^3^, S Huang^4^, A Mclean^4^

##### ^1^Nepean Hospital, Intensive Care Unit, Sydney, NSW, Australia; ^2^Amiens University Hospital, Medical ICU, Amiens, France; ^3^Nepean Hospital, Cardiology Department, Sydney, NSW, Australia; ^4^Nepean Hospital, Sydney, NSW, Australia

**Introduction:** We sought to assess feasibility of myocardial contrast perfusion echo (MCPE) in the critically ill and if MCPE could aid correlation of clinical acumen in detecting type 1 myocardial infarction [acute MI]. Diagnosis of acute MI in ICU is difficult and inappropriate intervention can be harmful.

**Methods:** Single centre, prospective, observational study. Critically ill adult patients with Troponin I >50ng/L and acute MI being considered were included. Exclusion criteria: poor echo windows (2pts), known ischaemic heart disease, contrast contraindications.

MCPE analyses flow in the myocardium. After transient microbubble destruction (‘flash’), combined replenishment rate and plateau intensity estimate myocardial blood flow in ‘region of interest’ (ROI) (Figure 1).

Ischaemia assessed by angiography (22pts), nuclear imaging (1pt), MRI (3pts), CTA (3pts) or normal repeat echo in stress induced cardiomyopathy (14pts). 2 cardiologists and 6 intensivists, blinded to outcome, analysed history, ECG, Troponin and 2D echo images to estimate likelihood of MI.

**Results:** 40 patients (28 female), age 65 years (IQR 44-73) with Troponin 1987 ng/L (400-4384). 6 had acute ischaemia (15%). MCPE analysis was feasible in 415/640 segments (68.8% [IQR 50-81]). No adverse events seen. Regional wall analysis and global longitudinal strain (GLS) were both abnormal, but no significant difference seen in acute MI group vs none. Significant difference was seen in MCPE assessment (3.3 vs 2.4dB/s, p=0.050). A myocardial blood flow 2.8dB/s had 67% sensitivity and 88% specificity vs acute MI. Clinical acumen had mild agreement with outcomes (r2 0.1, p=0.054; AUC 0.74); however, combining clinical acumen with MCPE analysis correlation improved (r2 0.3, p=0.03; AUC 0.87) (Figure 2).

**Conclusions:** MCPE is feasible in the critically ill and showed better correlation with presence of acute MI vs clinical acumen alone.

**Fig. 1 (abstract P154).**
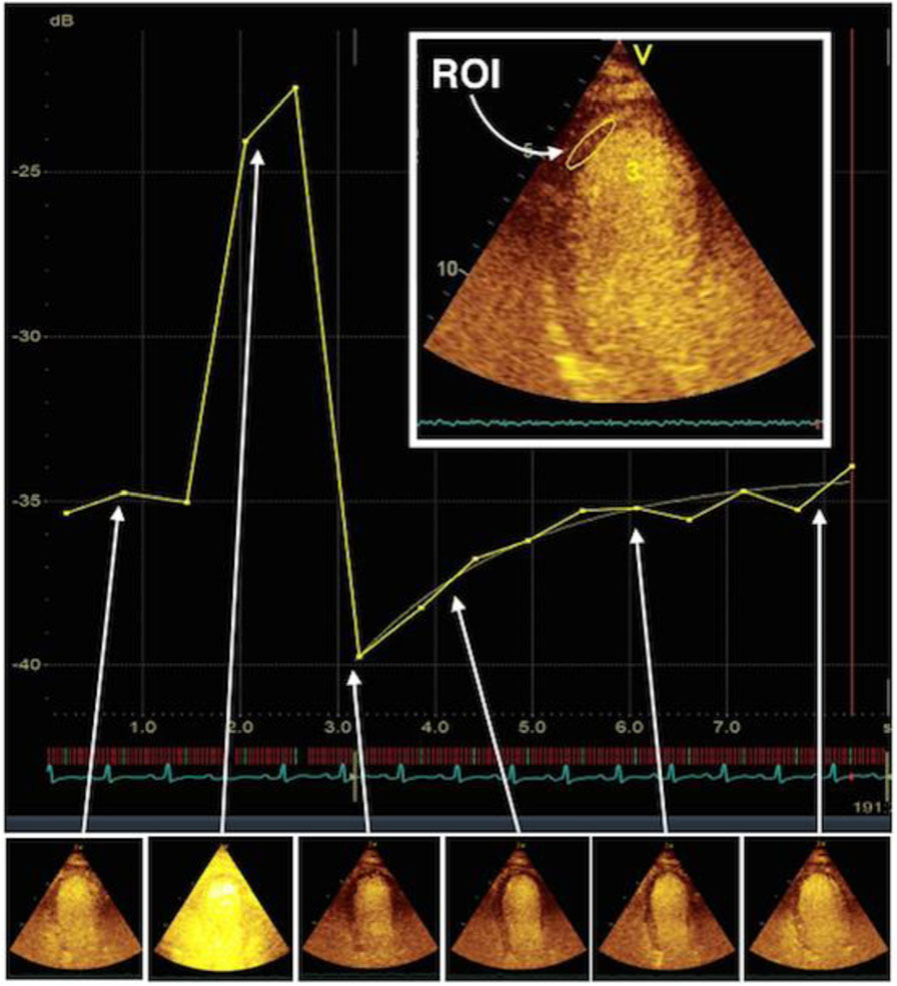
Myocardial contrast perfusion echocardiography (MCPE)

**Fig. 2 (abstract P154).**
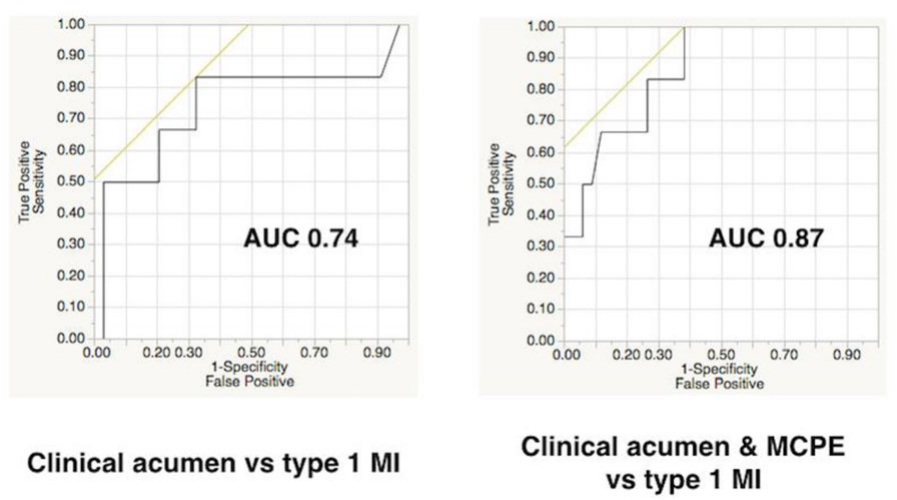
Receiver operating curves for detection of type 1 myocardial infarction

### P155 Non-invasive hemorrhage detection approach using photoplethysmography

#### JH Yoon, Y Chen, MR Pinsky, G Clermont

##### University of Pittsburgh, Critical Care Medicine, Pittsburgh, United States

**Introduction:** Many instances of significant bleeding may not occur in highly monitored environment, contribution in the delay in recognition and intervention. We therefore proposed a non-invasive monitoring for early bleeding detection using photoplethysmography (PPG).

**Methods:** Fifty-two Yorkshire pigs were anesthetized, stabilized and bled to hemorrhagic shock, and their invasive arterial blood pressure (ABP), and PPG data were collected [1]. Time series of vital signs were divided into data frames of 1 minute updated every 30 seconds and beat to beat features were computed. The final feature matrix contained 18 ABP features and 85 PPG features. A supervised machine-learning framework using Least Absolute Shrinkage and Selection Operator regularized logistic regression model was constructed to score the probabilities for hemorrhage of each data frame. Data in stabilization was set as negative and data in bleeding was set as positive. Model performance was evaluated by receiver operating characteristic (ROC) area under the curve (AUC) with leave-one-out cross validation, and its precision was assessed with activity monitoring operative characteristic (AMOC).

**Results:** Two different models were proposed using ABP and PPG features separately. Figure 1 showed the PPG model could classify the hemorrhage with AUC = 0.89, where the AUC of ABP model was 0.91. Figure 2 showed the PPG model could detect the hemorrhage on average 15.5 minutes (equals to 320 ml blood loss) if the false alarm rate of 1/100 was tolerated, whereas the average detection time of ABP model were 12.5 minutes at same threshold of false alarm rate.

**Conclusions:** We proposed a novel non-invasive bleeding detection approach using PPG signals only. This method potentially can improve the identification of hemorrhage with in patients and environments where invasive monitoring is unavailable.

**Reference**

1. Gomez H, et al. J Surg Res 2012.

**Fig. 1 (abstract P155).**
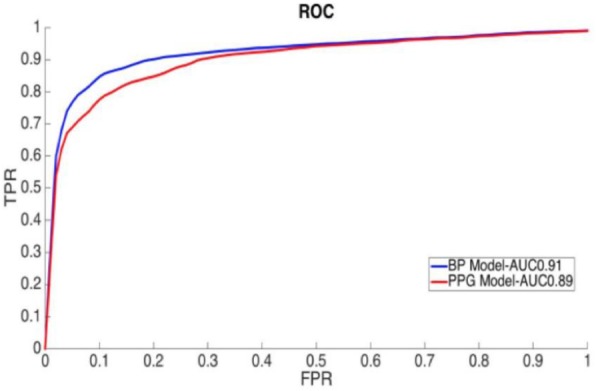
Comparison of model performance with two different vital sign feature sets, on Receiver Operating Characteristic (ROC) curve

**Fig. 2 (abstract P155).**
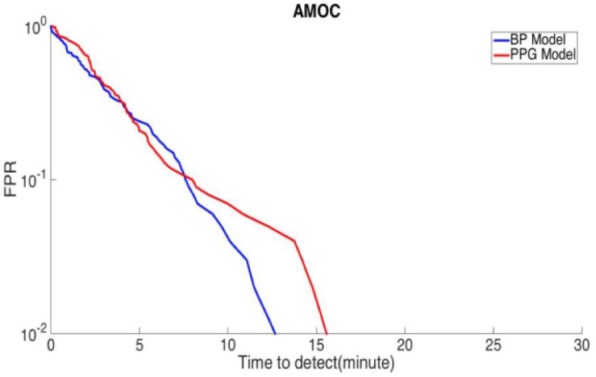
Comparison on precision, on Activity Monitoring Operating Characteristic (AMOC) curve

### P156 Feasibility of ultrasound guided central venous catheterization in critically ill patients without Trendelenburg position

#### A Dumoulin^1^, W Stockman^1^, P Lormans^1^, D Benoit^2^

##### ^1^AZ Delta, Intensive Care medicine & Anaesthesiology, Rumbeke, Belgium; ^2^University Hospital Gent, Intensive Care UZ Gent, Gent, Belgium

**Introduction:** In our experience, most central venous catheters in ICU patients can be placed without using Trendelenburg position (TP) if ultrasound is used before and during the procedure, thereby avoiding unwanted side effects of TP.

**Methods:** We conducted a prospective observational single centre study from 28/02/2018 till 08/06/2018 in 30 consecutive mixed ICU patients who needed central venous catheterization using real-time ultrasound guidance. Ultrasound was used pre-procedural to determine the appropriate location for catheterization and the size of the vessel in various non TP positions. If deemed necessary the physician could adjust the bed position to optimize ultrasound vessel visualization and patient comfort. We registered the angle of the upper body position using a digital protractor.

**Results:** Baseline characteristics are shown in Table 1, catheter and procedure characteristics are shown in Table 2. The median angle of bed position was 26°. No patients were positioned in neutral or TP. All procedures were successful with a mean of 1.3 punctures per patient, and a maximum of 3. The median procedure time was 12.5 minutes. No major complications occurred in any of our patients.

**Conclusions:** Central venous catheterisation in moderate upright position is feasible and can be done safely when using real-time ultrasound by well-trained physicians. We recommend performing clinical assessment and pre-procedural ultrasound to choose the optimal puncture site and position in order to attain an optimal ultrasound visualisation of the vessel and patient comfort.

**Table 1 (abstract P156).** Patient demographics

Hemodynamic support	Yes 53% (16/30)	No 47% (14/30)
Mechanical Ventilation	Yes 47% (14/30)	No 53% (16/30)
Gender	Male 73% (22/30)	Female 27% (8/30)
	Median	Inter quartile range
paO2/FiO2	193	121-264
Age (years)	70.5	63.5-76
SOFA score at the day of the procedure	8	7-11

**Table 2 (abstract P156).** Catheter+ procedure characteristics

Catheter position	internal jugular vein 53% (16/30)	subclavian vein 40% (12/30)
Catheter properties	7french central venous catheter: 60% (18/30)	12french dialysis catheters: 37% (11/30)
	Median	Inter quartile range
Number of punctures	1	1 - 2
Angle of positioning (°)	26°	22°- 35°
Minimal venous diameter (mm)	7.7 mm	5.5mm -11.7mm
Procedure time (minutes)	12.5 min	10 min -15.5min

### P157 A survey of practice for central venous catheter placement confirmation methods

#### K Chen, C Day

##### Royal Devon & Exeter Hospital, Critical Care, Exeter, United Kingdom

**Introduction:** Once a central venous catheter (CVC) has been inserted, its position should be confirmed and complications excluded. Ultrasound is commonly used to aid insertion but the extent to which it is used in confirmation of site and exclusion of complications is not known for the UK.

**Methods:** We conducted a telephone survey contacting all critical care units in England. The nurse in charge or an Advanced Critical Care Practitioner was asked to provide information about what constituted usual practice for most CVC lines inserted in their ICU. If they were busy a call back was arranged. If this failed then a further call was made to that ICU.

**Results:** 147 of the 190 ICU contacted provided information (77%). 94.6% used ultrasound to confirm the position of the guide wire during insertion. This could include exclusion of a wire in the subclavian vein or visualisation as far caudally as possible. 95.2% of units used a chest X-ray. 65.3% used waveform analysis from the line. 42.9% would use ultrasound to look for the catheter tip and/or to exclude pneumothorax. 35.4% used a blood gas taken from the line.

**Conclusions:** There is still a great dependancy on the chest x-ray to confirm position and exclude complications following CVC insertion. USS is used, mostly as an adjunct in confirming position and excluding complications. The use of USS is very dependant on an operator being available. There were several comments that practice during the day was different to those at night. During the day there was more likely to be a skilled operator, able to use USS for confirmation of line site and placement.

**Reference**

American Society of Anaesthesiologists Task Force on Central Venous Access. Anaesthesiology 2012; 116: 539-75;

### P158 Ultrasound-guided oblique right brachiocephalic vein central venous cannulation (CVC) in adult: a novel approach

#### R Ismail, A Osman, A Ahmad, A Azil

##### Hospital Raja Permaisuri Bainun, Emergency & Trauma, Ipoh Perak, Malaysia

**Introduction:** The aim of this study was to evaluate the feasibility right brachiocephalic vein central vein cannulation (CVC) using high frequency linear ultrasound probe via an oblique needle trajectory [-3].

**Methods:** A retrospective analysis of 10 patients presenting to tertiary-care emergency department who required CVC for vasopressor administration was carried out. All central venous cannulation into the right brachiocephalic vein was performed with ultrasound guidance using the high frequency linear probe. Right brachiocephalic vein was visualised in its long axis. The needle was positioned just beside the centre of ultrasound probe 15 degrees below the coronal plane and 10 degrees angle to the ultrasound probe and advanced just behind the clavicle.

**Results:** The mean puncture time taken to perform this procedure, calculated from the needle piercing the skin until to the aspiration of blood from the brachiocephalic vein through the needle, was 23 ± 6.8s. No procedure-related complications were detected.

**Conclusions:** The oblique needle trajectory of right brachiocephalic vein CVC in adult is feasible and able to visualised well the anatomical structure, hence avoid complications.

**References**

1. Breschan, et al. Br JAnaesth 106 (5): 732–7 (2011)

2. Byon HJ, et al. Br J Anaesth 111 (5): 788–92 (2013)

3. Klug W, et al. Saudi J Anaesth. 2016;10(2):143-148.24.

### P159 Identifying risk factors of central venous catheter placement related complications: an observational multicenter study

#### JM Smit^1^, TS Steenvoorden^1^, ME Haaksma^1^, EH Lim^1^, MJ Blans^2^, FH Bosch^2^, M Petjak^3^, B Vermin^3^, HR Touw^1^, AR Girbes^1^, L Heunks^1^, PR Tuinman^1^

##### ^1^Amsterdam UMC, Location de Boelelaan, Intensive Care Medicine, Amsterdam, Netherlands; ^2^Rijnstate Hospital, Intensive Care Medicine, Arnhem, Netherlands; ^3^Groene Hart Ziekenhuis, Intensive Care Medicine, Gouda, Netherlands

**Introduction:** Central venous cannulation, a routine procedure on intensive care units, is associated with a low complication rate. As a consequence, the routine use of chest X-ray (CXR) or ultrasound (US) to assess these complications is under discussion. Our aim was to identify risk factors for central venous catheter (CVC) placement associated complications that can help decide whether or not follow-up using CXR and/or US is indicated.

**Methods:** Multicenter prospective, observational study. Consecutive critically ill adult patients who underwent CVC placement. Either the internal jugular vein or subclavian vein was cannulated. Complication rates were determined. Predicting factors were obtained through a questionnaire filled in by physicians after placing a CVC. If the questionnaire was incomplete or data was missing, analyses were performed using the available data. Patient characteristics were duplicated if a patient recieved more than one CVC. Outcomes were iatrogenic pneumothorax and malposition. Pneumothorax was detected using US, whereas CXR was used to determine CVC malposition.

**Results:** We included 756 CVC insertions in 727 patients. Median age was 68 (IQR: ± 15.0) years and men outnumbered women more than 2:1 (519 vs. 239). Mechanical complications were identified in 4.8 (n=36) of all CVC placements:11 pneumothoraces and 26 malpositions. Clinically relevant complications occurred in 1.2% (n=9) of the cases. Risk factors for CVC-placement related complications are shown in Table 1. US-guidance, insertion site, and setting were predictive for complications.

**Conclusions:** The overall CVC placement associated complication rate is low and multiple risk factors associated with the occurrence complications were identified. A complication rate this low, strongly suggests that routine post-procedural diagnostics is superfluous. Therefore, we suggest, provided that uneventful execution of the procedure is assured, post-procedural diagnostics are only necessary in selected cases with (multiple) risk factors.

**Table 1 (abstract P159).** Peri-procedural factors and the outcomes of CVC placement they correspond with

	No complications (N=719)	Complications (N=37)	Total (N=756)	P value
US-guided vs. Landmark technique (N)	247 (56.0%) vs. 194 (44.0%)	20 (76.9%) vs. 6 (23.1%)	267 (57.2%) vs. 200 (42.8%)	0.036
IJV-cannulation vs. SV-cannulation (N)	691 (96.1%) vs. 28 (3.9%)	33 (89.2%) vs. 4 (10.8%)	724 (95.8%) vs. 32 (4.2%)	0.042
Elective insertion vs. Emerency insertion (N)	477 (66.3%) vs. 242 (33.7%)	15 (40.5%) vs. 22 (59.5%)	492 (65.1%) vs. 264 (34.9%)	0.001

Numbers in between brackets indicate percentages of total amount of CVCs in that category. P values portraying correlation of predictive factors and positive outcomes. If the questionnaire was incomplete or data was missing, analyses were performed with the available data. It was unclear in 289 cases if US-guidance or the landmark technique was used to insert the CVC. CVC: central venous cathteter; US: ultrasound; IJV: internal jugular vein; SV: subclavian vein

### P160 Supraclavicular approach to ultrasound-guided subclavian vein cannulation. A preliminary study

#### Y Aissaoui

##### Avicenna Military Hospital - Caddi Ayyad University, Department of Anesthesiology and Intensive Care, Marrakesh, Morocco

**Introduction:** The use of ultrasound for subclavian vein cannulation (SCV) has developed poorly due to the difficulty of visualizing this vein via the classical infraclavicular approach. We explored the feasibility of ultrasound-guided subclavian vein catheterization via a supraclavicular approach

**Methods:** Prospective study conducted over six-month period in intensive care unit. After approval of the ethics committee, we included patients over 18 years of age and requiring central venous access. Exclusion criteria were: hemostasis disorders, puncture area infections and cervico-thoracic vascular malformations The procedure consisted of catheterization of the VSC with a supraclavicular approach under ultrasound guidance using an ultrasound in plane approach (Fig 1 and 2). Data collection included clinical and ultrasound data: SCV depth, diameter and length, catheterization time, number of needle redirection, cannulation success and complications.

**Results:** Thirty four patients were included. age: 57 ± 15 (Mean ± SD), 60% of whom were male. The success rate of SCV catheterization was 97% (one failure). The depth of the SCV was 11 ± 4.5 mm and its diameter was 11 ± 3.5 mm. The puncturable length of the SCV was 33 ± 7mm and the puncture angle was 36 ± 15 °. The time required to obtain an adequate ultrasound image was 25 ± 9 seconds. The interval between the beginning of the puncture and the insertion of the guidewire into the vein was 42 ± 29 sec. The total catheterization time was 69 ± 33 seconds. The number of needle redirection 0.8 +/- 1.2 redirects. The quality of the ultrasound image was excellent or good in 87.5% of cases. An arterial puncture was observed in two patients

**Conclusions:** This preliminary study demonstrated the feasibility of the subclavian vein cannulation via the supraclavicular approach. More study are required to confirm its safety and to compare this approach to the infraclavicular acces using ultrasound.

**Fig. 1 (abstract P160).**
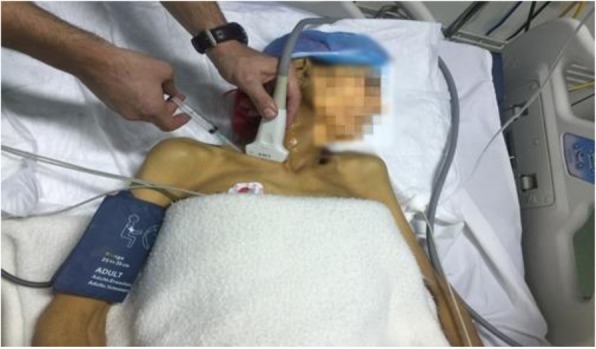
Probe positioning in the supraclavicular fossa during ultrasound guided subclavian vein cannulation via supraclavicular approach

**Fig. 2 (abstract P160).**
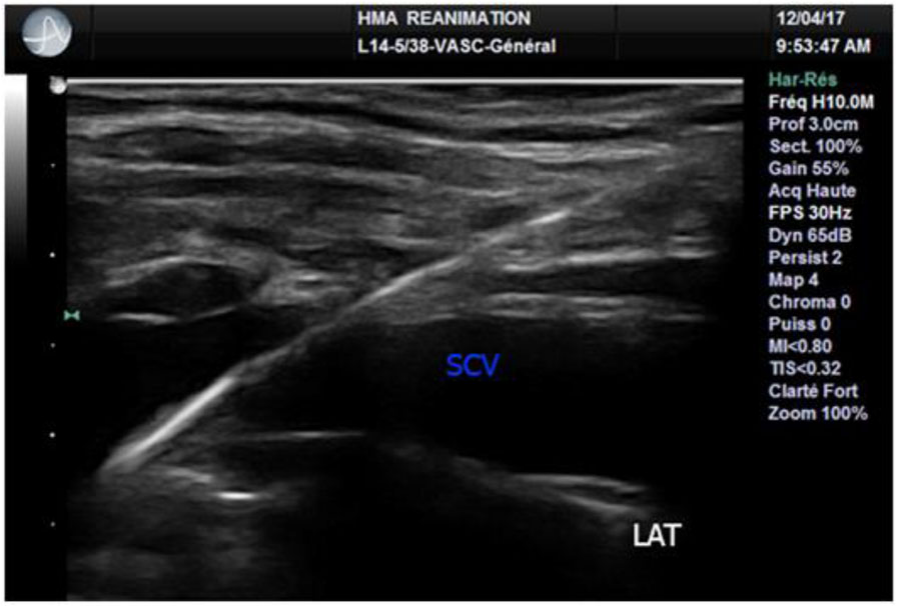
Ultrasound image of the subclavian vein after cannulation and insertion of the guidewire

### P161 Contribution of the ultrasound guidance in the set up of central venous catheters in emergency situations

#### W Sellami, I Ben mrad, Z Hajjej, M Chniti, I Labbene, M Ferjani

##### Department of critical care medicine and anesthesiology Military Hospital, Tunis, Tunisia

**Introduction:** In intensive care unit, we are confronted to place venous catheters in urgent situations. Although the femoral site is preferred, it remains difficult to handle in case of abdominal pelvic surgery or ventral position. In addition it does not allow to develop a diagnostic approach. The purpose of our study was to see if ultrasound guided cannulation of the internal jugular vein could be an alternative to the femoral one.

**Methods:** It was a prospective, monocentric, observational, and comparative study conducted in the anesthesia resuscitation department of the Military Hospital Main Instruction of Tunis over a period of 12 months. There were 118 patients, 58 in the group “guided femoral vein catheterization (FVC)” and 60 in the group “guided internal jugular vein catheterization (IJVC)”. The rate of failure and complications (mechanical, infectious and thrombotic) was compared; the number of punctures and access times too. The threshold of statistical significance was chosen at 0,05.

**Results:** The failure rate was 10.3% for FVC, compared to 8.3% for IJVC (p = 0.47, (p >0.05)). The risk of hematoma was 3.4% for FVC, 1.7% for IJVC (p = 0.5).No case of pneumothorax was noted, 2 malpositions of the IJVC were reported.Catheter-related infection was 6.9% for FVC, 3.3% for IJVC (p = 0.3). Venous thrombosis was 20.7% for FVC, 10% for IJVC, (p = 0.08). The number of attempts and the access time were lower for IJVC (respectively 1.3 ± 0.4 and 204s ± 46.3 vs 1.9 ± 0.7 and 256.5s ± 90.4 for the FVC, p <0.0001).

**Conclusions:** The failure rate and complications were comparable between the 2 groups, but the ultrasound-guided internal jugular catheter appears to be faster to insert and requires fewer punctures, so it could be an alternative to the femoral one in emergency situations.

### P162 Correlation of lung ultrasound B-lines and New York Heart Association(NYHA) functional classification in congestive heart failure

#### CY Cheong, A Osman, AH Ahmad, MH Mohamed Sakan

##### Hospital Raja Permaisuri Bainun, Emergency and Trauma Department, Ipoh, Malaysia

**Introduction:** Lung ultrasound B-lines, a comet-like reverberation artefacts arising from water-thickened interlobular septa, indicate extravascular lung water which is a key variable in heart failure management and prognosis. Aim of this study is to measure the correlation between lung ultrasound B-lines and NYHA functional classification.

**Methods:** This is a 6 months prospective study on congestive heart failure patients conducted in 2 urban emergency departments in Malaysia. Following enrolment, patients had their functional capacity categorised based on NYHA classification, followed by Point of Care Ultrasound (POCUS) lung scan using a 12MHz linear probe. The scanning was performed by trained emergency physicians. The longitudinal scan done at the recommended 6 zones of both left and right lungs and the total number of B-lines identified were summed up as the comet score. Comet Score of 0, 1, 2 and 3 were categorised based on amount of B-lines of less than 5, 5-15, 15-30 and more than 30 B-lines respectively.

**Results:** Hundred and twenty-two patients were analysed (69 males(56.6%) and 53 females(43.4%)) ranging from 24 to 88 years old. Comet Score of 1,2 and 3 were found to be statistically significant with presence of paroxysmal nocturnal dyspnoea, elevated jugular venous pressure, lung crackles, bilateral pitting oedema and chest radiographic findings. A moderate correlation between NYHA classes with Comet Score 1,2 and 3 (rs= 0.77 (P<0.01)) was documented.

**Conclusions:** Our study demonstrated a moderate correlation between NYHA classes and lung ultrasound B-lines. Lung ultrasound may be a potential tool to objectively determine the functional capacity in patients with congestive heart failure and monitor its changes in response to treatment and disease progression.

### P163 The use of online communications as a tool for ultrasound peer review: a survey of users

#### O Olusanya^1^, J Wilkinson^2^, P Parulekar^3^

##### ^1^St Mary´s Hospital, Intensive Care Unit, London, United Kingdom; ^2^Northampton General Hospital, Intensive Care Unit, Northampton, United Kingdom; ^3^East Kent Hospitals University NHS Trust, Intensive Care Unit, Kent, United Kingdom

**Introduction:** Point of Care Ultrasound (POCUS) is a tool of increasing utility in the management of the critically ill patient. Guidelines exist for training and accreditation in POCUS [1, 2] however the widespread use of POCUS has been hampered by a lack of mentors. Online communication with end-to-end security, such as WhatsApp ™ are increasingly used in medicine as a communication aid [3]. Some individuals are using such communications to share POCUS images for review- the overall sentiment around these tools is unknown.

**Methods:** An online survey of POCUS users was conducted via Twitter ™. The question was “in situations where an expert opinion on an ultrasound is not immediately available, is it acceptable to get an expert review via an online medium such as WhatsApp, and would you be happy to be that expert?”

**Results:** 304 votes were received. Voters were a mix of POCUS users from the USA, Europe, and Australia. 58% said the medium was acceptable, and that they would be happy to provide expertise. 34% voted “no”, with 8% voting “other” (Fig 1).

**Conclusions:** In this international survey of POCUS users, 58% were happy to provide and receive mentorship using remote software such as WhatsApp. Distance mentorship for POCUS training should be explored.

**References**

1. Volpicelli G, et al. ICM 2012; 38(4):577-591.

2. Via G et al. J Am Soc Echocardiogr. 2014 Jul;27(7):683.e1-683.e33

3. NHS England.https://t.co/E9fXW9agnD (last checked 27th Nov)

**Fig. 1 (abstract P163).**
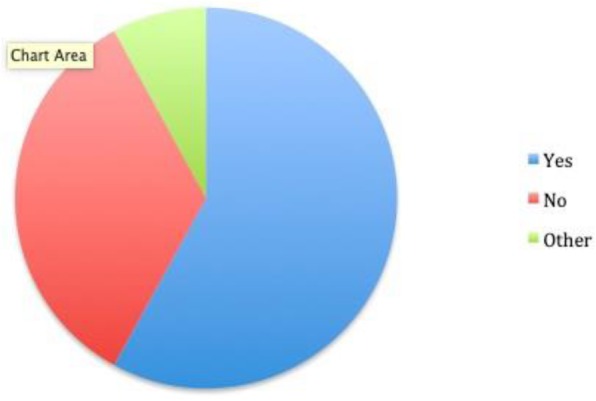
Distribution of votes in Poll

### P164 A modified Delphi process to develop an integrated scheduled inter-professional assessment of critically ill patients with ultrasound. INSIGHT

#### E Corcoran^1^, N Hare^2^, D Hadfield^1^, S Helyar^1^, P Hopkins^3^

##### ^1^Kings College Hospital London - London, Research, London, United Kingdom; ^2^GSTT, Research, London, United Kingdom; ^3^Kings College Hospital London - London, Intensive Care Medicine, London, United Kingdom

**Introduction:** Point of care, focused ultrasound offers safe, non-invasive imaging for immediate evaluation, resuscitation and guidance of therapeutic procedures in the critically ill patient [1]. Our previous research showed all critical care professions within our institution support a clinical effectiveness trial of nurse-led scheduled ultrasound [2]. A description of the development and refinement of INSIGHT - a feasibility and clinical effectiveness randomized controlled trial.

**Methods:** A modified Delphi exercise was used to select the most beneficial ultrasound windows and imaging questions to ask for each window in scheduled inter-professional ultrasound. 260 nurses, 46 doctors and 6 physiotherapists from critical care were given the same information regarding potential utility of each window. The windows and associated questions were individually ranked; each window and question tested against three further criteria; and filtered by ease of training to level 1 standard; clinical usefulness; time of practical delivery and applicability across an inter-professional group.

**Results:** The Modified Delphi exercises and prioritization exercise ranked ease of adoption by training; feasibility within the time frame and clinical usefulness to develop a core INSIGHT scan of 5 domains, each with set binary questions (Tables 1 and 2)

**Conclusions:** We have developed a research intervention that will allow us to test the effectiveness of inter-professional scheduled whole body assessment of critically ill patients by ultrasound. We now plan to conduct a clinical effectiveness trial with an internal pilot to confirm feasibility.

**References**

(1) Beaulieu Y&Marik P. 2005. Chest 128: 1766-81.

(2) Hare N, et al. 2014. Intensive Care Medicine 40 (conference abstract).

**Table 1 (abstract P164).** Results from the first stage of the Delphi exercises: 10 windows across the whole body were found to be useful. These are ranked in order of importance (1-10). A subsequent prioritization exercise by ease of adoption by training showed number of scans required for each window to reach level one standard (ranked 1-10). Time taken to scan each window (ranked 1-10) and clinical usefulness of each window (ranked 1-10). Total added up for each window and sums helped identify 5 key windows for use in the trial

Windows (ranked 1-10)	Number of scans to level 1 standard (1-10)	Time to complete ultrasound window (1-10)	Clinical usefulness (1-10)	Final
Bladder/pelvis 1	1	1	1	4
Sub-costal cardiac 4/ Sub-costal IVC 3	6/3	3/4	4/2	17/12
Central veins 5/Lower limb veins 8	2/8	6/7	3/7	16/30
R hemi-diaphragm 6/L hemi-diaphragm 7	5/4	2/5	5/6	18/22
Cerebral doppler 9	10	8	8	35
Apical Cardiac 2	7	10	9	28
Parasternal cardiac 10	9	9	10	38

**Table 2 (abstract P164).** Final choice of windows (subcostal cardiac/IVC, right hemi-diaphragm/right lower lobe/subphrenic assessment, abdominal ultraosund using bladder as acoustic window, left hemidiaphragm, central veins - internal jugular/subclavian/femoral. The questions that are required to be answered when scanning each window (outcome of Delphi exercises) are identified

Ultrasound window	Questions
1. Sub-costal cardiac and IVC	Heart visible;effusion;RV vs LV size; IVC patency
2. R hemi-diaphragm	effusion;consolidation;abdo fluid;diaphragm moving
3. Abdominal ultrasound	urine in bladder;catheter;free fluid surrounding
4. L hemi-diaphragm	effusion;consolidation;abdo fluid;diaphragm moving
5. Major central veins (L/R IJV/SCV)	Vein visible;depth;AP diameter

### P165 Central venous pressure cannot be reliably estimated by transthoracic echocardiography in critically ill patients

#### M Jozwiak, A Boilève, JL Teboul, C Richard, X Monnet

##### Hôpital Bicêtre, service de médecine intensive réanimation, Le Kremlin-Bicêtre, France

**Introduction:** The central venous pressure (CVP) remains a helpful variable at the bedside for hemodynamic management of critically-ill patients. Moreover, transthoracic echocardiography (TTE) is currently the first-line tool for evaluating the hemodynamic conditions in such patients. We sought to assess the ability of TTE to estimate and to track changes in CVP in critically ill patients.

**Methods:** In 31 patients (25 mechanically ventilated and four with atrial fibrillation), concomitant TTE examination and CVP measurement (internal jugular catheter) were performed before and after a passive leg raising test (n=10) or the infusion of 500-mL of saline (n=21).

**Results:** After pooling all values, CVP was only correlated to the end-expiratory inferior vena cava (IVC) diameter and the respiratory variations of the IVC diameter (r=0.40 and r=-0.26, respectively, p<0.05). At baseline, an end-expiratory IVC diameter ≤14 mm predicted a CVP ≤10 mmHg with a specificity of 100% (95%CI: 81-100%) but a sensitivity of 29% (95%CI: 8-58%). An end-expiratory IVC diameter >26 mm predicted a CVP >10 mmHg with a specificity of 100% (95%CI: 77-100%) but a sensitivity of 0% (95%CI: 0-20%). The respiratory variations of the IVC diameter could predict neither a CVP ≤10 mmHg nor a CVP >10 mmHg (AUC=0.683, p=0.08). Relative changes in CVP were weakly correlated to relative changes in S/D ratio and in velocity-time integral (VTI)-systolic filling fraction of the supra-hepatic vein flow (r=0.44 and r=0.43, respectively, p<0.05). The concordance rate between changes in CVP and S/D ratio or changes in VTI-systolic filling fraction was 48% and 55%, respectively.

**Conclusions:** In critically-ill patients, TTE cannot reliably estimate CVP or track changes in CVP induced by a passive leg raising test or fluid administration. However, an end-expiratory IVC diameter ≤14 mm or >26 mm can predict a CVP ≤10 mmHg or >10 mmHg, respectively, with a specificity of 100%.

### P166 Lung water assessment by lung ultrasonography

#### A Ramos^1^, A Dogliotti^2^, F Acharta^3^, D Latasa^1^, F Gerber^1^, J Robles^1^, C Lovesio^1^

##### ^1^Grupo Oroño, Critical Care, Rosario, Argentina; ^2^Grupo Oroño, Statistics and Epidemiology, Rosario, Argentina; ^3^Sanatorio Parque, Critical Care, Carlos Pellegrini, Argentina

**Introduction:** The objective was to investigate the accuracy of lung ultrasonography in the quantification of lung water in critically ill patients by using transpulmonary thermodilution technique as the gold standard for the determination of extravascular lung water level (ELWI).

**Methods:** Prospective study of 20 consecutive, fully mechanically ventilated patients who required hemodynamic monitoriztion with EV1000, were included. ELWI was determined using the transpulmonary thermodilution technique, a value above 10 ml/kg was considered as the presence of pulmonary water . Semiquantitative ultrasound assessment of lung water was performed by determining the ultrasound B-line, defined as the total number of B-lines detectable in an anterolateral lung ultrasonography examination, ≤ 3 B-lines was considered as absence of pulmonary water and >4 B-lines was considered as the presence of lung water.

**Results:** Good correlations was found between the presence of more than 3 B-line and ELWI; ≤ 3 B-lines, Kappa < 0 (no agreement) (p= 0.06); >3, Kappa 1 (good agreement) (p <0.0001). Sensitivity 58.3% (95% CI: 27 to 84), specificity 100%, positive predictive value 100%, negative predictive value 61.5% (95% CI: 31 to 86), (p= 0.006).

**Conclusions:** More than 3 B-lines by ultrasonography agreed with transpulmonary thermodilution technique. For the entire population: >3 B-lines by ultrasonography has a good positive predicted value. Lung ultrasonography examination may provide a reliable, simple and bedside lung densitometry in the intensive care setting.

### P167 Search for optimal strength and time for capillary refilling time

#### R Kawaguchi^1^, T Nakada^1^, T Oshima^1^, M Shinozaki^2^, T Nakaguchi^2^, H Haneishi^2^, S Oda^1^

##### ^1^Chiba University Graduate School of Medicine, Department of Emergency and Critical Care Medicine, Chiba, Japan; ^2^Chiba University Center for Frontier Medicine Enginieering, Chiba, Japan

**Introduction:** Capillary refilling time (CRT) has been widely used in clinical settings. However, CRT measurement conditions, including pressing strength and time, are not standardized. Search for optimal strength and time for CRT measurement seems to be needed.

**Methods:** We developed a novel device, which can adjust pressing strength and time, and can precisely measure CRT using an electric actuator and strength and color sensor. CRT was in 31 healthy adults using the device under conditions of pressing strength 1, 3, 5 and 7 N, and pressing time 1, 2, 3, 4, 5 and 6 s.

**Results:** There was a significant difference in CRT for pressing strength but not for pressing time (two-way ANOVA: power P<0.001, time P=0.97). There was a significant difference in CRT between pressing strength of 1 and 3 N but not for 3, 5 and 7 N (P=0.16). Thus 3 N seems to be needed for the pressing strength. To search for optimal pressing time, the plots from the color sensor during nail bed compression were analyzed. We found two phases in the color sensor plots. In the initial part of compression, the plots changes rapidly (rapid phase) and then the slope of plots reduces (slow phase). The pressure release during the rapid phase could destabilize the measurement. The longest period of the rapid phase was 1.9999 s among all the study subjects. Thus, a pressing time of 2 s seems to be needed to obtain stable CRT measurements.

**Conclusions:** On our study for the investigation of standard pressing time and strength for CRT measurements, pressing the nail bed with 3-7 N and 2 s appears to be optimal.

### P168 Detection of pancreas ischemia with microdialysis and CO2-sensors in a porcine model

#### K Rydenfelt^1^, R Strand-Amundsen^2^, R Horneland^3^, S Hødnebø^1^, TI Tønnessen^1^, H Haugaa^1^

##### ^1^Oslo university hospital, Department of Anesthesiology, Oslo, Norway; ^2^Oslo university hospital, Department of Clinical and Biomedical Engineering, Oslo, Norway; ^3^Oslo university hospital, Department of Transplantation Medicine, Section of Transplantation Surgery, Oslo, Norway

**Introduction:** Pancreas transplantation is associated with a high rate of early graft thrombosis. Current postoperative monitoring lack tools for early detection of ischemia, which could precipitate a graft-saving intervention. We are currently exploring the possibility of ischemia detection with microdialysis and CO_2_-sensors in the organ tissue or on the surface in a porcine model.

**Methods:** In anesthetized pigs, CO_2_-sensors and microdialysis catheters are inserted into the parenchyma or attached to the surface of the pancreas. PCO_2_ is measured continuously and lactate is sampled with microdialysis every 15 min. Ischemia is induced by sequential arterial and venous occlusions for 45 minutes, with 120 minutes of reperfusion in between.

**Results:** PCO_2_ increased and decreased in response to ischemia and reperfusion within minutes. Lactate increased and decreased with the same pattern, but with a considerable delay as compared to PCO_2_. An example is depicted in Figure 1. The values are presented in Table 1 (n=3).

**Conclusions:** Preliminary data suggest that PCO_2_ increase and decrease within minutes in response to ischemia and reperfusion in the pancreas and seem to correlate well with lactate measured with microdialysis both intraparenchymally and on the surface of the organ.

**Table 1 (abstract P168).** Tissue and surface lactate and PCO2 in response to ischemia and reperfusion, results from 3 pigs

	Parenchymal lactate (mmol/L)	Surface lactate (mmol/L)	Parenchymal PCO_2_ (kPa)	Surface PCO_2_ (kPa)
Baseline	1.1 ± 0.2	2.5±0.6	7.8±4.6	10.2±9.6
Max after arterial occlusion	8.0±0.2	7.8±0.9	18.1±4.5	18.9±13.2
Baseline after reperfusion	1.8±0.6	3.2±0.8	8.7±2.9	10.1±7.0
Max after venous occlusion	4.9±2.5	6.4 ±3.7	15.8±8.2	15.5±7.7

Values shown as mean±SD

**Fig. 1 (abstract P168).**
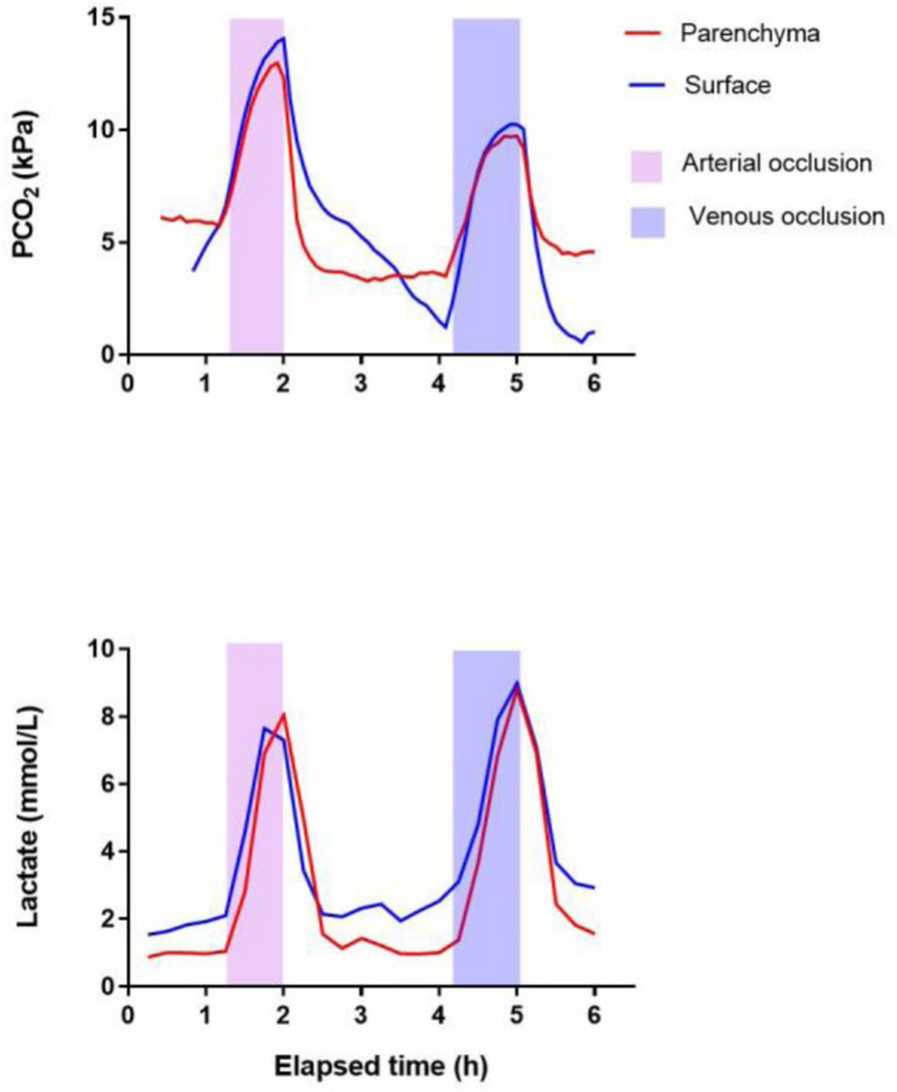
Ischemia detected with CO2-sensors (PCO2) and microdialysis (lactate), in the parenchyma and on the surface of the pancreas. Example from one pig

### P169 Validation of automated software for analysis of the sublingual microcirculation in clinical and experimental settings

#### MP Hilty^1^, P Giaccaglia^1^, S Akin^1^, O Erdem^1^, B Ergin^1^, DM Milstein^2^, F Toraman^3^, Z Uz^1^, G Veenstra^4^, C Ince^1^

##### ^1^Erasmus MC, Department of Intensive Care, Rotterdam, Netherlands; ^2^University Medical Center, Amsterdam, Netherlands; ^3^Acıbadem Mehmet Ali Aydınlar University School of Medicine, Istanbul, Turkey; ^4^Medical Center Leeuwarden, Leeuwarden, Netherlands

**Introduction:** Reliable automated handheld vital microscopy (HVM) image sequence analysis is a prerequisite for use of sublingual microcirculation measurements at the point-of care according to the current consensus statement. We aim to validate a recently developed advanced computer vision algorithm [1] versus manual analysis in a wide spectrum of populations and contexts.

**Methods:** Our collaborators were invited to contribute raw data of published or ongoing institutional review board approved work. Inclusion criteria were use of the Cytocam HVM device, manual analysis with the AVA software, and image quality as independently assessed by Massey score of <10 in >50% of recordings in a random subset of each study. 233 subjects from 11 studies were included, covering clinical and experimental populations, major shock forms and interventions to recruit the microcirculation (Table 1).

**Results:** 2,599,710 red blood cells were tracked by the algorithm across 150,163 frames in 1462 measurements in real time. A good to excellent correlation was found between algorithm-determined and manual capillary density (p<0.0001, r 0.6–0.9, Figure 1). Capillary perfusion was classified using space-time diagram derived red blood cell velocity (RBCv), yielding good correlation with manual analysis for functional capillary density und proportion of perfused vessels. Microcirculatory alterations during disease and interventions were equally detected by the algorithm and manual analysis. Change in flow short of severe abnormality was reflected in absolute RBCv but not microcirculatory flow index.

**Conclusions:** We demonstrate the validity of automated software for HVM image sequence analysis across broad populations, disease conditions and interventions. Thus, microcirculatory assessment at the bedside may finally complement point-of-care evaluation of disease severity and treatment response in critically ill patients and during surgery.

**Reference**

1. Hilty et al, ICMx 2018;6(suppl 2):40;172-173.

**Table 1 (abstract P169).**
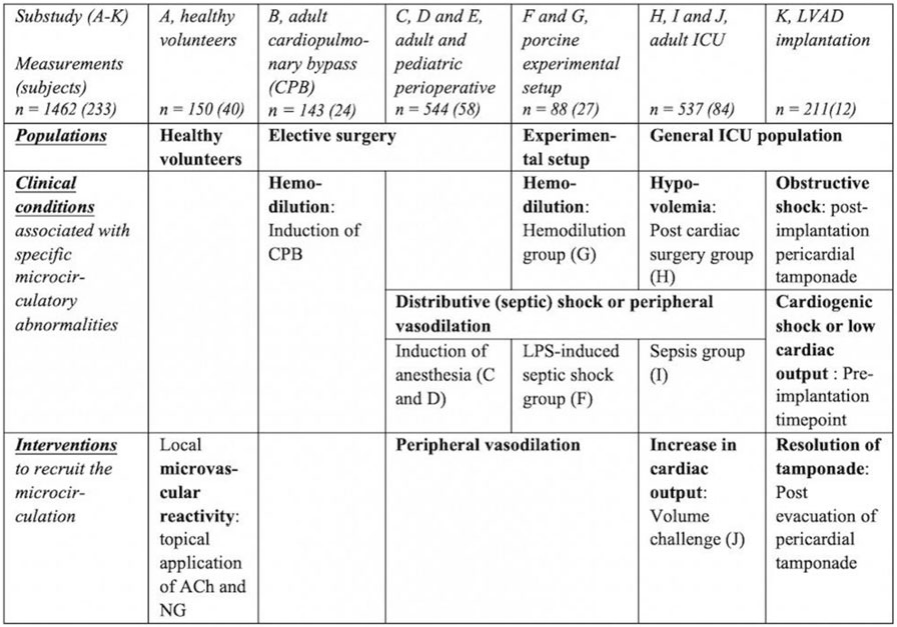
Our study population covers a wide range of major patient populations, clinical conditions associated with specific microcirculatory abnormalities, as well as interventions to recruit the microcirculation

ICU, intensive care unit; LVAD, left ventricular assist device; CPB, cardiopulmonary bypass; LPS, lipopolysaccharide; ACh, acetylcholine; NG, nitroglycerin

**Fig. 1 (abstract P169).**
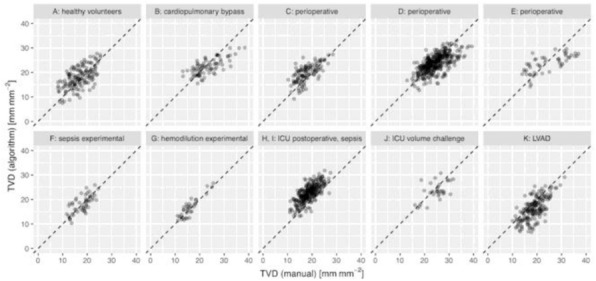
A good correlation was found between algorithm-determined and manually measured capillary total vessel density (TVD). Substudy indices (A-K) correspond to Table 1. Dashed lines represent identity lines. TVD, capillary total vessel density; ICU, intensive care unit; LVAD, left ventricular assist device

### P170 Validating the point of care microcirculation (POEM) score to characterize sublingual microcirculatory flow

#### H Fargaly^1^, A Deitchman^1^, M Gilani^1^, J Assadi^1^, S Hutchings^2^, M McCurdy^1^

##### ^1^University of Maryland, School of Medicine, Pulmonary and critical care, Baltimore, United States,^2^King´s College Hospital, Critical Care, London, United Kingdom

**Introduction:** In 2016, Naumann et al introduced the POEM score as a real-time, point-of-care score to assess sublingual microcirculation [1]. Our study aimed to determine the reproducibility of the POEM score.

**Methods:** Two expert operators used a sidestream darkfield (SDF) videomicroscope (Cytocam, Braedius, Netherlands) to separately acquire four high-quality video clips and assign a POEM score to each image in 20 adult mechanically ventilated patients. Each operator was blinded to the other’s images and analysis. Video clip scores and acquisition times were recorded.

**Results:** Of the 20 patients enrolled in this study, 45% (n=9) required vasopressors. We categorized POEM scores 4-5 as “normal” and POEM scores 1-3 as “impaired.” (Fig 1). With only one instance of interrater disagreement (i.e., a single image scored as 3 versus 4), Cohen’s kappa (0.86) confirmed a strong correlation between interpreters. The mean time to complete a study session was 9 minutes.

**Conclusions:** The present inability to quickly characterize the quality of sublingual microcirculation as either normal or impaired at the point of care limits real-world clinical application of this resuscitative endpoint. The rapidly obtained POEM score appears to be reproducible between bedside interpreters. Future studies should assess the effect of POEM score-guided resuscitation.

**Reference**

1. Naumann DN et al. Crit Care. 2016; 20:310.

**Fig. 1 (abstract P170).**
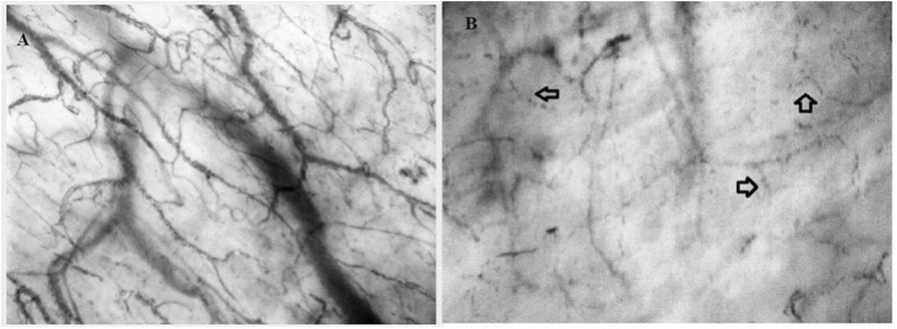
Microcirculatory images for two different subjects

### P171 Microcirculatory response to passive leg raising in recreational marathon runners

#### I Kiudulaite^1^, Z Pranskuniene^2^, J Arstikyte^3^, M Brazaitis^4^, A Pranskunas^5^

##### ^1^Lithuanian university of health sciences, Department of intensive care medicine, Kaunas, Lithuania; ^2^Lithuanian university of health sciences, Department of Drug Technology and Social Pharmacy, Kaunas, Lithuania; ^3^Lithuanian university of health sciences, Institute for Digestive Research, Kaunas, Lithuania; ^4^Lithuanian Sports University, Sports Science and Innovation Institute, Kaunas, Lithuania; ^5^Lithuanian university of health sciences, Department of Intensive Care Medicine, Kaunas, Lithuania

**Introduction:** We hypothesize that marathon runners have different microcirculatory response to passive leg raising (PLR) evaluated before and after marathon.

**Methods:** Eleven healthy marathon runners with a training volume of at least 5 h per week for a minimum of 2 years volunteered for the study. PLR were performed two times: 24 hours prior to their participation in the Kaunas Marathon (distance: 41.2 km) and directly after finishing the marathon. The systemic hemodynamic and microcirculatory measurements were obtained before and after PLR. Systemic hemodynamic parameters were measured using impedance cardiography (Niccomo, Medis, Medizinische Messtechnik GmbH, Germany). Sublingual microcirculatory images were obtained using a Cytocam-IDF device (Braedius Medical, Huizen, The Netherlands) and analyzed using standardized published recommendations.

**Results:** The median age of participants was 32 years. We found no significant difference in proportions of hemodynamic responders before and after marathon (64% vs 55%, p=0.819). Also we did not find differences between PLR induced changes of total vessel density (TVD) and proportion of perfused vessels (PPV) of small vessels before and after marathon. Correlations between changes of sroke volume and changes of TVD or PPV of small vessels during PLR were not significant.

**Conclusions:** Marathon running did not change microcirculatory responsiveness.

### P172 Correlation between perfusion index and lactate level in critically ill patients

#### K Kumwilaisak, J Sereeyotin

##### King Chulalongkorn Memorial Hospital, Anesthesiology Department, Bangkok, Thailand

**Introduction:** Macrocirculation and microcirculation are the key components to determine organ perfusion. Early detection of tissue hypoperfusion would guide us to actively manage and lead to the better outcome. Presently, we commonly use serum lactate as a representative of global tissue perfusion but it needs blood sampling and obtains intermittent data. Perfusion index(PI) based on analysis of pulse oximetry signal is noninvasive continuous monitoring for peripheral tissue perfusion. Previous studies have shown correlations between PI and microcirculation parameters in critically ill patients, however, there is still few data. We aim to investigate the correlation between PI and serum lactate level in critically ill patients

**Methods:** A prospective observational study was performed. All patients who had signs of tissue hypoperfusion and serum lactate level ≥ 2 mmol/L were enrolled and received hemodynamic resuscitation based on standard clinical practice of KCMH. PI was measured by Masimo Radical-7®Pulse CO-Oximeter® and recorded values simultaneously with serum lactate at 0, 2, 6 and 24 hours during resuscitation

**Results:** Of the 42 critically ill patients (Table 1), we found significant correlation between PI and lactate level at 0 and 2 hours (r=-0.397, p=.009 and r=-0.311, p=.045 respectively). The change in PI also significantly correlate with lactate clearance at first 6 hours of resuscitation (r=0.444, p=.003 at 0-2 hour and r=0.370, p=.017 at 2-6 hours) (Fig 1). 24 patients(57%) had lactate clearance ≥ 10% within 2 hours, whereas 18 patients(42.8%) had not (Table 2, Fig 2). The cut-off value of increasing in PI<0.86 predicted patients who were not lactate clearance at 2 hours with sensitivity of 88.9% and specificity of 54.2%.(AUC 0.699, 95%CI 0.54-0.86, p=.029)

**Conclusions:** PI may has value as an adjunct continuous monitoring for peripheral tissue perfusion in early resuscitation period by using concurrently with serum lactate. Increasing of PI< 0.86 within 2 hours after resuscitation prompt us to do further management with our patients.

**Table 1 (abstract P172).** Patients characteristics

Age, mean+/-SD (years)	67.07+/-11.52
Gender	M35 (83.30%), F 7(16.67%)
Baseline SOFA score, median(IQR)	3.5(2-6)
Postoperative care	36(85.71%)
Sepsis	3(7.14%)
Combined	3(7.14%)
Mechanical ventilator, median(IQR)(hrs)	17.5 (13.25-48.75)
ICU length of stay, median (IQR)(day)	2(2-4)

**Table 2 (abstract P172).** Comparison of parameters between lactate clearance group and lactate non-clearance group

Variables	Lactate clearance N= 24 (57%)	Lactate non-clearance N= 18 (42.8%)	P value
Δ PI (0-2 hr), median (IQR)	1.05 (0.07-2.35)	0.17 (-0.38-0.60)	.029*
ScvO2 at 2 hour(%), mean(SD)	71.28 (8.55)	66.85 (10.76)	.213
Pv-aCO2 at 2 hour (mmHg), median(IQR)	6.1 (4.2)	6.2 (7.0)	.806
Baseline SOFA score, mean (SD)	3.83(4.72)	4.72(3.64)	.384

**Fig. 1 (abstract P172).**
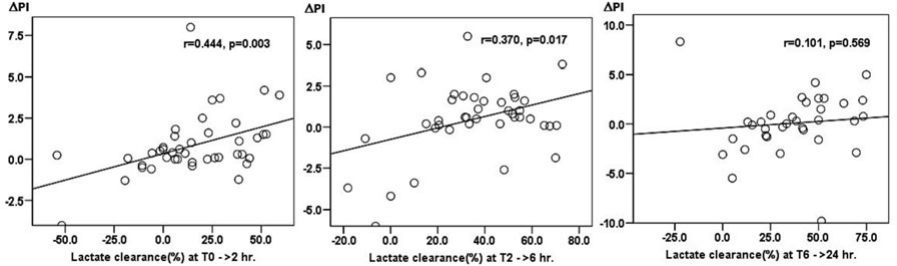
Relationship between change in PI and lactate clearance (%)

**Fig. 2 (abstract P172).**
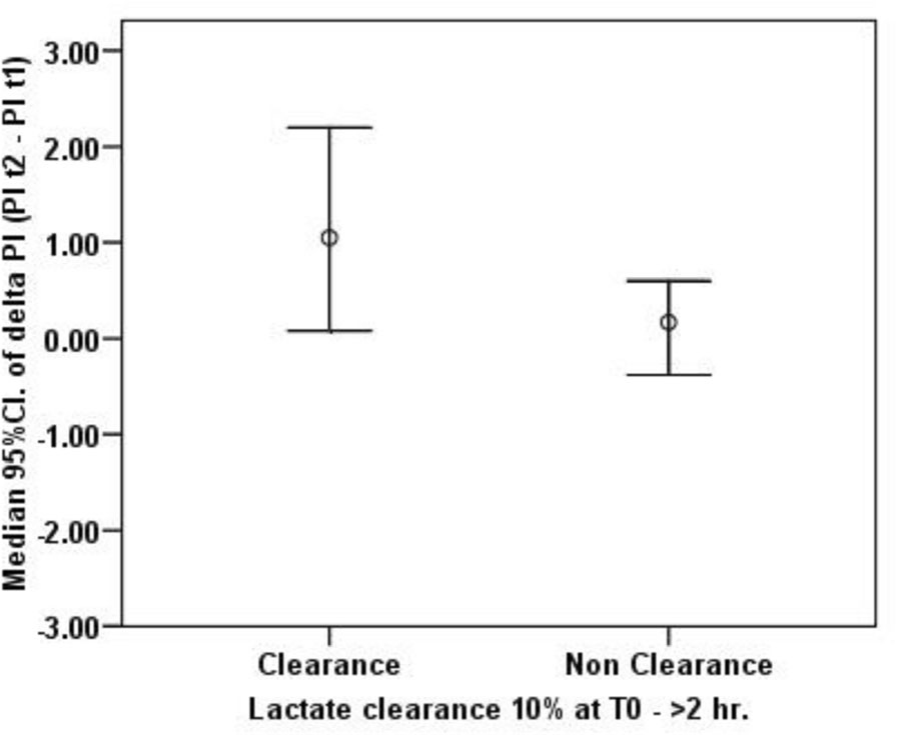
Comparison of the change in PI between lactate clearance group and lactate non-clearance group

### P173 WITHDRAWN

### P174 Acute changes in lactate levels after bolus fluid treatment in critically ill patients

#### C Pierrakos ^1^, T Nguyen^1^, D Velissaris^2^, R Attou^1^, L Kugener^1^, J Devriendt^1^, PM Honore^1^, D De Bels^1^

##### ^1^CHU-Brugmann, Brussels, Intensive Care, Brussels, Belgium; ^2^University Hospital of Patras, Internal Medicine Department, Patras, Greece

**Introduction:** Increased serum lactate is a frequent triggering factor for initiation of bolus fluid treatment (BFT). The aim of this study was to investigate the clinical utility of monitoring acute changes in serum lactate levels for assessing the response to BFT.

**Methods:** This is a prospective observational study in critically ill adult patients, including patients who received either 1 L crystalloids or 500 ml colloids within 30 to 40 min after lactate measurement. Cardiac index (CI) and serum lactate levels were measured before and after BFT. Patients with base-line levels of lactate <2mmol/L (Group 1), and patients with lactate ≥2mmol/L (Group 2) were analyzed separately. Responders to BFT were considered those who had an increase in CI ≥15%.

**Results:** We assessed 69 critically ill patients who received BFT. The patients’ mean age was 69±16 years, and the APACHE II score 22.7±8. Thirty-four patients had a baseline lactate value of <2 mmol/L, and 35 had ≥2mmol/L. Group 1: Fifteen patients (44%) responded to BFT (%dCI:37% ± 14). A statistically significant increase in lactate levels was observed in these patients (from 1.39 mmol/L ±0.3 to 1.65 mmol/L ±0.4, p<0.01) after the end of BFT. Lactate levels remained unchanged in non-responders (1.41 mmol/L ±0.4 versus 1.56 mmol/L±0.5, p=0.11). Group 2: Twelve patients (34%) responded positively to BFT (%dCI: 29% ±8) but had no significant changes in lactate levels (3.6 mmol/L ±0.4 versus 3.8 mmol/L ±0.5, p=0.32). Changes in lactate were also not observed in non-responders (3.9 mmol/L ±0.4 versus 4.1 mmol/L ±0.5, p=0.66).

**Conclusions:** Acute changes in lactate levels cannot be used for assessing responses to BFT. An increase in lactate levels can be observed in BFT responders likely because of a washout phenomenon. We did not observe any significant decrease in lactate concentration secondary to hemodilution effect in non-responders.

### P175 Mitochondrial oxygen availability and regional saturation during CPB

#### E Mik, R Kortlever, M Ter Horst, F Harms

##### Erasmus MC - University Medical Center Rotterdam, Anesthesiology, Rotterdam, Netherlands

**Introduction:** Clinical measurement of mitochondrial oxygen tension (mitoPO_2_) has become available with the COMET system [1]. A question with any novel technique is whether it is feasible to use in clinical practice and provides additional information. In elective cardiac surgery patients we measured cutaneous mitoPO_2_ and tissue oxygenation (StO_2_).

**Methods:** Institutional Research Board approved observational study in patients undergoing cardiopulmonary bypass (CPB). mitoPO_2_ measurements were performed on the left upper arm (COMET, Photonics Healthcare B.V.) by oxygen-dependent delayed fluorescence of 5-aminolevulinic acid (ALA)-induced protoporphyrin IX [2]. Priming of the skin was done with ALA (Alacare, Photonamic GmbH) applied the evening before surgery. StO_2_ measurements (INVOS, Medtronic) were done in close proximity to the COMET sensor.

**Results:** At the time of writing 28 of 40 patients were enrolled and mitoPO_2_ measurements were feasible in this clinical setting. mitoPO_2_ appeared sensitive with a high dynamic range. For example, high-dose vasopressor therapy decreased mitoPO_2_ and blood transfusion increased a low mitoPO_2_ but not a high mitoPO_2_. In the example in Figure 1, mitoPO_2_ is clearly dependent on CPB flow and the restored cardiac circulation is able to maintain good cutaneous oxygenation after CPB even before returning of cellsaver blood. StO_2_ had the tendency to provide relatively stable values within a small bandwidth and little response to even major hemodynamic changes.

**Conclusions:** mitoPO_2_ shows the effect of interventions on mitochondrial oxygenation and provides additional information compared to standard monitoring and StO_2_.

**References**

1. Ubbink R et al. J Clin Mon Comput 31:1143-1150, 2017

2. Mik EG. Anesth Analg 117:834-846, 2013

**Fig. 1 (abstract P175).**
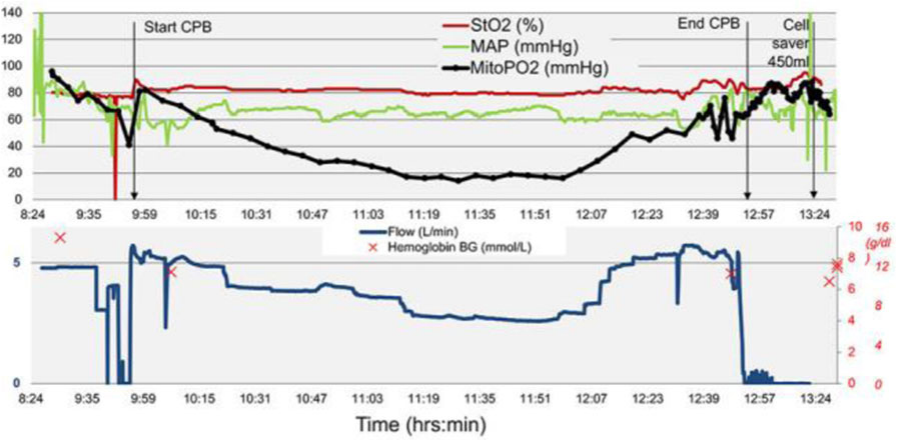
Example of mitoPO2 and StO2 during CPB

### P176 Frailty assesment in cardiopulmonary arrest, is it necessary?

#### N Arriero Fernández, JA Silva Obregón, A Estrella Alonso, Z Eguileor Marin, MA Tirado Fernández, R Viejo Moreno, JE Romo Gonzales, C Marian Crespo

##### Hospital universitario de Guadalajara, Intensive care unit, Guadalajara, Spain

**Introduction:** The aim of this study is to describe the impact of frailty in cardiopulmonary arrest (CPA)

**Methods:** A descriptive, observational and retrospective study was carried out from January 2015 to June 2018. We analyzed interconsultations to ICU for CPA in this period. Age, sex, Clinical Frailty Scale(CFS), causes, limitation of CPR and outcomes were determined.

For statistical analysis of the data, quantitative variables are expressed as means +/-standard deviation; qualitative variables as percentages. The statistic analysis was made by using Chi scuare test. Multivariate analysis was performed using a logistic regression model.

**Results:** We analyze 106 patients, 18 of them were excluded. Frailty divide patients in 2 categories: prefrail and frail (PF-F)(CFS>3) and non-frail (NF)(CFS<4). The univariate analyses revealed no statistically significant difference between groups in age (mean PF-F age=75.98 years +/- 11.28; mean NF age=72.37 years+/-9.58; p=0.15) (Table 1). Most of patients were men (68.2%). Men were significant more likely to be frail (59% PF-F men vs 88,9% NF men OR 5.56 (CI95% 1.51-20.48) p=0.01). Main CPA causes were respiratory 46,6%; coronary heart disease (CHD) 17%; other causes 18,2%; unknow causes 18,2%. Patients suffering from CHD were significant less likely to be frail OR 0.28 (CI95% 0.08-0.97) p=0.04. CPR were stopped in 19.3%, patients who were limited were significant more likely to be frail (27.9% vs 0%; OR 2 (CI95% 1.01-2.63) p=0.04). 94.3% died. Patient that did not survive to discharge were significant more likely to be frail (98.4% vs 85.2% OR 10.43 (CI95% 1.11-98.35) p=0.04). Multivariate analysis included several variables. Only frailty was statistically significant (OR 11.35 (CI95% 1.03-125.52) p=0.04

**Conclusions:** Assessing frailty could predict patient mortality in CPA. It may help clinicians in decision-making

**Table 1 (abstract P176).**
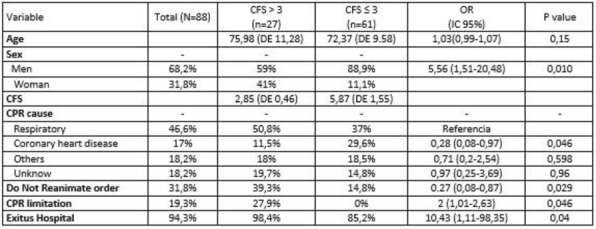
Univariate analysis

### P177 Prehospital ventilation strategies during cardiopulmonary resuscitation

#### G Jansen, N Kappelhoff, K Götz

##### Evangelisches Klinikum Bethel, Anaesthesiology, Intensive Care and Emergency Medicine, Bielefeld, Germany

**Introduction:** While there is increasing evidence for the importance of chest compressions for the outcome of cardiopulmonary resuscitation (CPR), recommendations for ventilation strategies during CPR are rather poor. Although continuous ventilation at 10 breaths per minute and the avoidance of hyperventilation is suggested after intubation, the guidelines provide no information regarding the implementation of differentiated ventilation strategies (DVS) [1]. The present survey was designed to determine the DVS used in the prehospital setting by German emergency physicians (GEP) during CPR

**Methods:** An anonymous web-based questionnaire encompassing 10 questions was sent to GEP during September and October 2018. The EP were asked to specify the ventilator model, their preferred VS during CPR and the initial set of ventilator parameters, i.e.: volume- vs. pressure-controlled ventilation, respiratory rate, tidal volume, maximum and minimum pressure limits.

**Results:** 61 % of the questionnaires were completed (151/247). 95 % of respondents had preclinical access to respirators with the ability to perform DVS. 75 % of the participants chose volume-controlled, 19 % pressure-controlled ventilation modes during CPR. 6 % of participants indicated that they were performing manual ventilation. 53% chose a non-guidance-compliant ventilation with respiratory rates > 12/min. The corresponding group characteristics are shown in Table 1.

**Conclusions:** Despite the possibility of preclinical implementation of differentiated VS, GEP predominantly use volume-controlled ventilation modes. In contrast to the current guidelines, 53 % of GEP hyperventilate the patients under the CPR. Future studies are necessary to evaluate the influence of prehospital DVS on outcome of preclinical CPR.

**Reference**

[1] Perkins GD et al. Resuscitation 123:43-50, 2018

**Table 1 (abstract P177).** Prehospital ventilation strategies during cardiopulmonary resuscitation

		Volume controlled ventilation	Pressure controlled ventilation
Respiratory Rate	< 10 /min	1 (1 %)	-
	10 – 12 /min	52 (46 %)	15 (54 %)
	> 12 /min	61 (53%)	13 (46 %)
Tidal Volume	< 400 ml	10 (9 %)	5 (18 %)
	400 – 600 ml	103 (90 %)	21 (75 %)
	> 600 ml	1 (1 %)	2 (7 %)

### P178 Traumatic asphyxia, focused on presence of cardiac arrest and the therapeutic reactivity

#### S Kikuta, S Ishihara, S Kai, H Nakayama, S Matsuyama, T Kawase, S Nakayama

##### Hyogo Emergency Medical Center, Department of Emergency and Critical Care Medicine, Chuo, Kobe, Hyogo, Japan

**Introduction:** Traumatic asphyxia is a rare condition in which breathing and venous return is impaired due to a strong compression to the upper abdomen or chest region, and induces swelling, purplish red appearance, and petechiae around the face and neck. To our knowledge, there are no reports describing details of traumatic asphyxia including the clinical course and the therapeutic reactivity from cardiac arrest. We focused on cardiac arrest among all traumatic asphyxia patients treated at our hospital, and investigated their clinical features and therapeutic reactivity.

**Methods:** Sixteen cases of traumatic asphyxia involved with our hospital between April 2007 and March 2018 were reviewed by using the pre-hospital activity record, medical record, and Hyogo prefectural inspection record. These patients were divided into three groups. The first group had already cardiac arrest at the time of rescue from the trapped place (Group A; 6 cases). The second group became cardiac arrest after the rescue (Group B; 5 cases). The third group did not experience cardiac arrest (Group C; 5 cases).

**Results:** All cases had abnormal findings in skin or conjunctiva (Table 1). Total mortality rate reached 56%, but among 11 cases of Group A and B who resulted in cardiac arrest, there were 10 cases with Injury Severity Score 16 or more and Abbreviated Injury Scale in the chest 3 or more. They had pneumothorax, flail chest, pericardial hematoma. Seven of them restored spontaneous circulation, and two cases achieved neurologically full recovery.

**Conclusions:** There are some cases of traumatic asphyxia whose therapeutic reactivity is very good even after cardiac arrest, so it is important not to spare efforts for life support in such cases.

**Table 1 (abstract P178).** Patients´ characteristics of each group

	Total (n=16)	A group (n=6)	B group (n=5)	C group (n=5)
Age(y.o.)	43(17-70)	44(36-58)	35(31-59)	43(17-70)
Time for compression (minutes)	5(1-37)	15(3-37)	5(3-12)	4(1-20)
Petechiae/congestion of skin or conjunctiva	16(100%)	6(100%)	5(100%)	5(100%)
Abbreviated Injury Scale of chest	4(0-5)	4(0-5)	5(4-5)	3(0-4)
TRISS-Ps	0.41(0.01-1.00)	0.08(0.01-0.53)	0.15(0.01-0.56)	0.99(0.42-1.00)
Return of spontaneous circulation	7/11(64%)	3(50%)	4(80%)	-
In hospital death	9(56%)	5(83%)	4(80%)	0(0%)

### P179 Physiological derangements are cumulatively associated with poor outcome after cardiac arrest

#### Z Haxhija^1^, A Bilkanovic^1^, L Lucas^2^, J Dziodzio^1^, R R. Riker^1^, T May^1^, N Nielsen^3^, H Friberg^4^, D B. Seder^1^

##### ^1^Maine Medical Center, Department of Critical Care Services and Neuroscience Institute, Portland, United States; ^2^Maine Medical Center, Center for Outcomes Research and Evaluation, Portland, United States; ^3^Helsingborg Hospital, Department of Anesthesia and Intensive Care, Helsingborg, Sweden; ^4^Skåne University Hospital, Department of Anaesthesia and Intensive Care, Lund, Sweden

**Introduction:** Hyperglycemia, hypotension, seizures, dysoxia and dyscarbia may contribute to secondary brain injury and have been identified as independent risk factors for poor outcome after cardiac arrest. It is unknown, however, if these physiological derangements have a cumulative association with outcome, and may represent an underappreciated treatment target.

**Methods:** We performed retrospective analyses of prospectively collected data from the International Cardiac Arrest registry. Demographics, clinical characteristics and physiological parameters from the first 24 hours were abstracted and assessed in bivariate and multivariable analyses, with standard confounders forced in. The primary outcome variable was delayed functional outcome, defined by the Cerebral Performance Status (CPC) dichotomized to good (CPC 1–2) or poor (CPC 3–5) outcome, measured about 6 months after resuscitation. Missing data were assessed by multiple imputation.

**Results:** Among the 2024 patients eligible for analysis, with a median ischemic time of 25 (15–36) minutes, 44% shockable initial heart rhythm and 75% witnessed arrest, five hundred ten (25%) patients had a good functional outcome at 6-months. Physiological derangements were each negatively associated with outcome in bivariate analysis at the p <0.001 level. A summary score of physiological derangements was included with potential confounders in the final regression model, and was independently associated with outcome with the chance of a good outcome decreasing by 46% for each increase of one physiologic derangement (95% CI 0.46–0.63).

**Conclusions:** Uncorrected physiological derangements are independently and cumulatively associated with worse outcome after cardiac arrest. Although causality cannot be established, it is reasonable to consider that the correction of physiological parameters may be an important step in the chain of survival after resuscitation.

### P180 Characteristics and clinical outcomes of cardiac arrest patients admitted at the Intensive Care Unit from 2010-2016 in the Netherlands

#### L Mandigers^1^, F Termorshuizenb^2^, N De Keizerc^3^, D Gommers^1^, D Dos Reis Mirand^1^, W Rietdijk^1^, C Den Uila^1^

##### ^1^Erasmus MC-University Medical Center, Department of Intensive Care Medicine, Rotterdam, Netherlands; ^2^Rivierduinen Psychiatric Institute Leiden, Department of Psychosis research, Leiden, Netherlands; ^3^University of Amsterdam, NICE foundation, Academic Medical Center, Amsterdam, Netherlands

**Introduction:** Worldwide, cardiac arrest remains a major cause of death. Many of the post cardiac arrest patients will be admitted at the Intensive Care Unit, however little is known about these patients. This patient group is relevant, because of the amount of patients and their vulnerability. For this reason, this study was set up to describe the characteristics and clinical outcomes of cardiac arrest patients admitted at the Intensive Care Unit.

**Methods:** We used data from the Dutch National Intensive Care Evaluation registry [1] from 2010-2016 in the Netherlands. All adult patients admitted at the Intensive Care Unit with cardiac arrest as admission diagnosis were included.

**Results:** In this study, characteristics and clinical outcomes of 26,056 cardiac arrest patients are described. Over time, the survival rate of Out-of-hospital cardiac arrest patients significantly decreases, while the survival rate of In-hospital cardiac arrest patients showed no significant relation.

**Conclusions:** This study shows a nationwide overview of the characteristics and outcomes of cardiac arrest patients admitted at the Intensive Care Unit. The decrease in mortality rate of out-of-hospital cardiac arrest patients may point to improvements made in post cardiac arrest care, however future research has to be done to specify the cause of this decrease.

**Reference**

1. NICE. Data in beeld. 2016; https://stichting-nice.nl/datainbeeld/public.

### P181 Outcomes following cardiac arrest at a UK cardiac arrest centre: a retrospective observational study

#### R Pugh^1^, L Pugh^2^, Z Habib^3^

##### ^1^Glan Clwyd Hospital, Anaesthetics/ Intensive Care, Denbighshire, United Kingdom; ^2^Cardiff University, Cardiff University, Cardiff, United Kingdom; ^3^Glan Clwyd Hospital, Critical Care, Denbighshire, United Kingdom

**Introduction:** Glan Clwyd Hospital (GCH) was recently designated one of three Cardiac Arrest Centres for Wales. It has offered a 24/7 Percutaneous coronary angiography (PCI) service to a geographically dispersed North Wales population of approximately 690,000 since June 2017. Prior to this, urgent coronary angiography was available on a more limited basis to patients requiring PCI. The aim of this study was to investigate factors associated with hospital mortality after critical care admission following cardiac arrest.

**Methods:** Retrospective review of the Ward Watcher critical care database at GCH to identify patients who had undergone CPR in the 24 hours prior to critical care admission in 2013-18. Patients likely to have sustained OOHA of cardiac aetiology (OOHA-C) were identified from primary and secondary diagnoses and free text entry. Data were subsequently analysed using Excel and SPSS. The project was registered as a service evaluation with GCH Audit Department.

**Results:** There were 190 cardiac arrest admissions over this period, increasing from 25 in 2013-14 to 69 in 2017-18. Of these 122 were OOHA, of which 103 were considered OOHA-C. Although OOHA-C hospital mortality appeared to decrease over the time period (89%% to 56%), this was not statistically significant (p=0.149). Factors associated with survival to hospital discharge are presented in the Tables below. On logistic regression, only PCI and low pH within the first 24 hours of critical care remained statistically significant (p=0.027 and p<0.001 respectively).

**Conclusions:** Although we have been unable to make a distinction between patients presenting following STEMI and NSTEMI, and appreciating a potential influence of selection bias, the significant association between PCI and survival to hospital discharge supports the introduction of clinical pathways enabling PCI access following OOHA-C [1].

**Reference**

1. Nolan J, et al. Resuscitation.2015; 95: 202-222

**Table 1 (abstract P181).** Factors associated with survival to hospital discharge (categorical)

OOHA-C patients	Survivor (n=41)	Non-survivor (n=62)	All (n=103)	P Value
Sex (male)	31 (76%)	39 (63%)	70 (68%)	0.201
Pre-hospital independence	33 (80%)	42 (68%)	75 (73%)	0.18
PCI	26 (63%)	17 (27%)	43 (42%)	<0.001

**Table 2 (abstract P181).** Factors associated with survival to hospital discharge (continuous, median)

OOHA-C patients	Survivor (n=41)	Non-survivor (n=62)	All (n=103)	P Value
Age (years)	59	65.5	63	0.08
APACHE II	15.3	19	17	0.007
P:F ratio (kPa)	25.9	18.2	22.6	0.008
Low SBP	89	85	86	0.185
Lactate	2.5	3.7	3.1	<0.001
pH	7.29	7.17	7.23	<0.001
Temperature	35.5	34.6	35	<0.001

### P182 Determination of theoretical personalised optimum chest compression point with anteroposterior chest radiography

#### S Chon^1^, S Kim^1^, W Oh^2^, S Cho^1^

##### ^1^CHA Bundang Medical Center, CHA University School of Medicine, Emergency medicine, Seongnam, South Korea; ^2^Kangwon National University School of Medicine, Internal Medicine, Chuncheon, South Korea

**Introduction:** Following the traditional assumption that the point (P_max.LV) beneath which the left ventricle (LV) is at its maximum diameter should be compressed to maximise stroke volume, we reported how to locate personalised P_max.LV using posteroanterior chest radiography. [1] Here, we aimed to derive and validate rules to estimate P_max.LV using anteroposterior chest radiography (chest_AP), which is performed for critically-ill patients urgently needing determination of personalised P_max.LV.

**Methods:** A retrospective, cross-sectional study was performed with non-cardiac arrest adults who underwent chest_AP and computed tomography (CT) within 1 h (derivation:validation=3:2). On chest_AP, we defined CD (cardiac diameter), RB (distance from right cardiac border to midline) and CH (cardiac height, from carina to uppermost point of left hemi-diaphragm) (Fig 1, 2). [1] Setting P_zero (0, 0) at the midpoint of xiphisternal joint and designating leftward and upward directions as positive on x and y axes, we located P_max.LV (x_max.LV, y_max.LV). The coefficients of the following mathematically-inferred rules were sought: x_max.LV=a0*CD-RB; y_max.LV=ß0*CH+γ . (a0: mean of (x_max.LV+RB)/CD; ß0, γ : representative coefficient and constant of linear regression model, respectively). [1]

**Results:** Among 360 cases (52.0±18.3 y, 102 females), we derived: x_max.LV=0.643*CD-RB and y_max.LV=55-0.390*CH (Table 1, Fig 2). This estimated P_max.LV (19±11) was as close as the averaged P_max.LV (19±11, P=0.13) and closer than three equidistant points representing the current guidelines (67±13, 56±10, and 77±17; all P<0.001) to the reference identified on CT, and thus was validated (all units: millimetre) (Table 2).

**Conclusions:** Personalised P_max.LV can be estimated with chest_AP. Further studies with actual cardiac arrest victims are needed to verify the safety and effectiveness of the rule.

**Reference**

1. Cho S et al. Resuscitation 2018;128:97-105.

**Table 1 (abstract P182).** The constants derived from the derivation set and those among total cases including both the derivation and validation set

Derived constants	Derivation (n=237)	Total (n=360)
a	0.643 ± 0.073	0.643 ± 0.080
ß (95% CI)	-0.425 (-0.556, -0.294)	-0.390 (-0.498, -0.282)
γ (95% CI)	60 (45, 75)	55 (43, 68)

**Table 2 (abstract P182).** The differences among estimated P_max.LV (x_max.LV, y_max.LV) using posteroanterior chest radiography, those of the reference P_max.LV measured on CT, and the three points along the lower sternal half with (top) at its top, (middle) at its middle and (bottom) representing the sites of chest compression recommended by the current CPR guidelines

Distance from the reference coordinate/point to (m	Validation (n=123)	p	Total (n=360)
x_max.LV (estimated)	1±13	N/A	0±12
y_max.LV (estimated)	1±19	N/A	0±18
P_max.LV (estimated)	20±11	N/A	19±11
P_max.LV (averaged)	19±11	0.13	19±11
P_guideline (top)	67±13	<0.001	67±13
P_guideline (middle	56±11	<0.001	56±10
P_guideline (bottom)	76±18	<0.001	77±17

**Fig. 1 (abstract P182).**
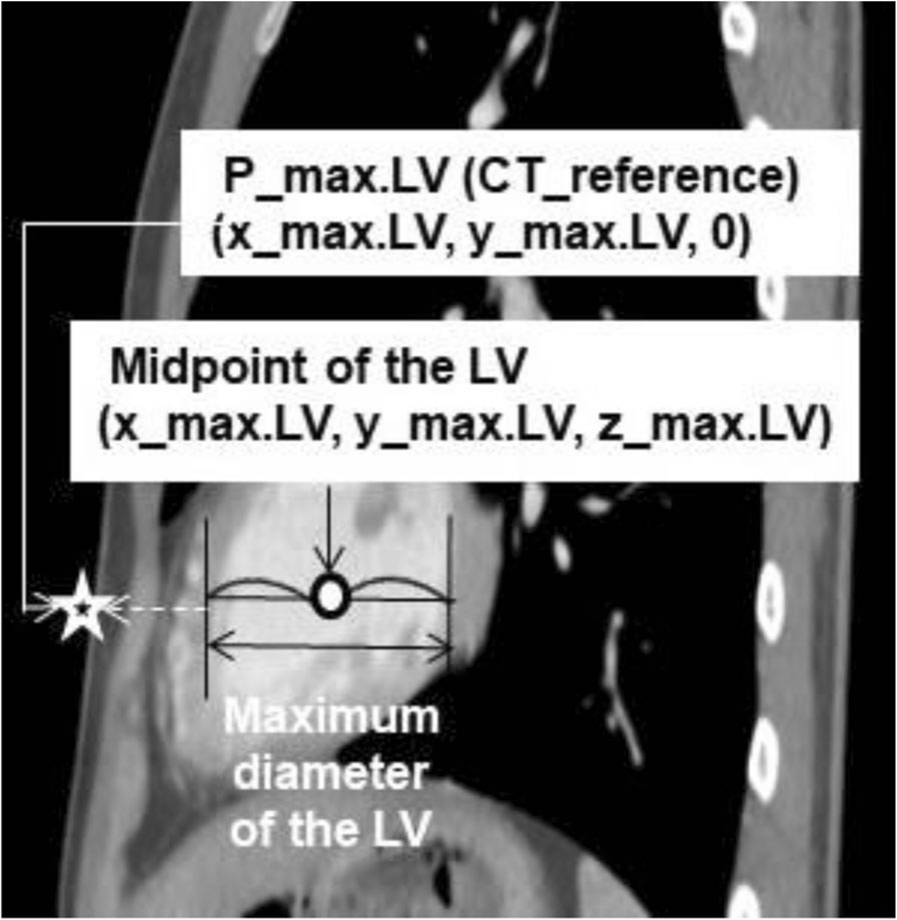
Identification of the theoretical optimum chest compression point (modified from a figure of reference 1)

**Fig. 2 (abstract P182).**
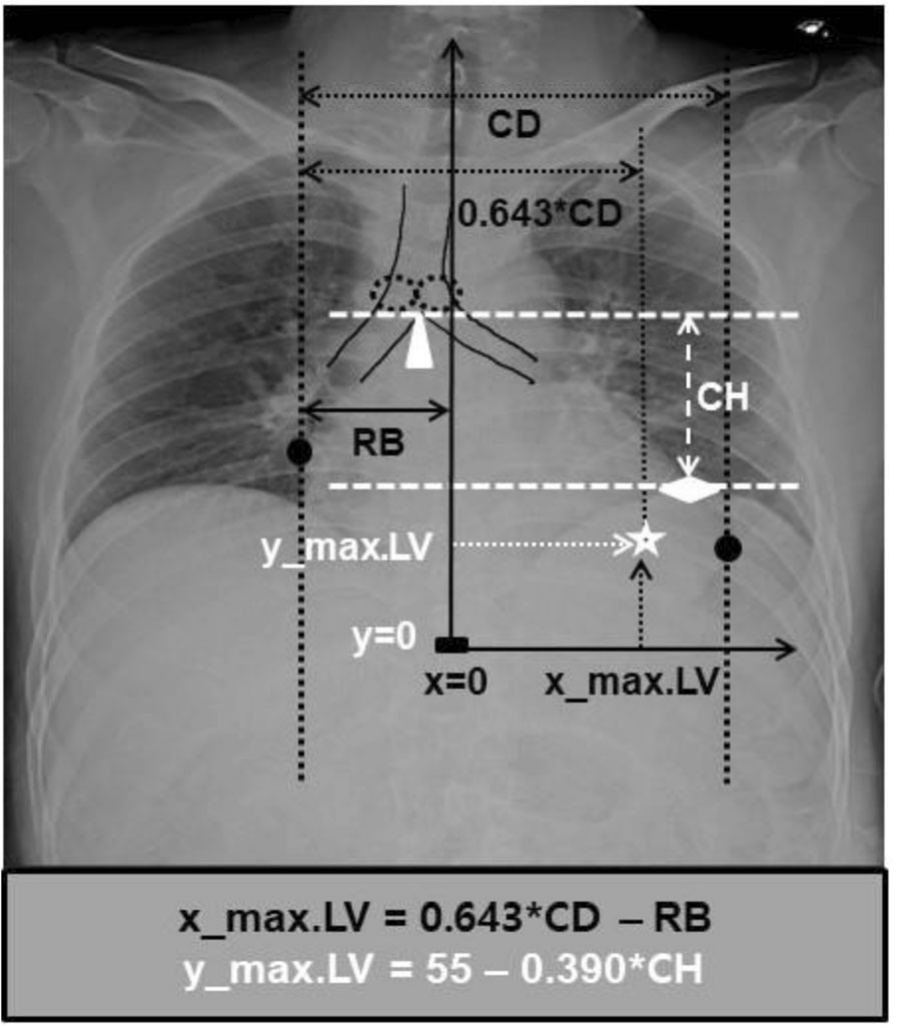
Estimation of the theoretical optimum chest compression point from anteroposterior chest radiography (modified from a figure of reference 1)

### P183 WITHDRAWN

### P184 Correlation between end-tidal CO2 and degree of compression of heart cavities measured by transthoracic echocardiography during cardiopulmonary resuscitation for out-of-hospital cardiac arrest

#### R Skulec, P Vojtisek, V Cerny

##### Masaryk hospital Usti nad Labem, Dept. of Anesthesiology, Perioperative Medicine and Intensive Care, Usti nad Labem, Czech Republic

**Introduction:** We realized clinical study to evaluate whether changes of the left ventricular (LV), right ventricular (RV) and inferior caval vein (IVC) diameter induced by chest compressions during cardiopulmonary resuscitation (CPR) for out-of-hospital cardiac arrest (OHCA) evaluated by transthoracic echocardiography correlate with end-tidal CO2 (EtCO2) levels.

**Methods:** 20 consecutive patients resuscitated for OHCA were included to the study. Transthoracic echocardiography was performed from subcostal view during ongoing chest compressions in all of them. This was repeated three times during CPR in each patient and EtCO2 levels were registered. From each investigation, video loop was recorded. Later on, maximal and minimal diameter of LV, RV and IVC were obtained from the recorded loops and compression index of LV (LVCI), RV (RVCI) and IVC (IVCCI) was calculated as *(maximal-minimal/maximal diameter)x100*. CImax defined as the value of LVCI or RVCI, whichever was greater was also assessed. Correlations between EtCO2 and LVCI, RVCI, IVCI and CImax were expressed as Spearman´s correlation coefficient (r).

**Results:** Evaluable echocardiographic records were found in 13 patients and a total of 39 measurements of all parameters were obtained. Chest compressions induced significant compressions of all observed cardiac cavities (LVCI=21.58±13.25%, RVCI=38.23±22.46%, IVCCI=62.26±30.84%, CImax=40.55±20.53%). We identified positive correlation between EtCO2 and LVCI (r=0.579, p<0.001), RVCI (r=0.752, p<0.001) and IVCCI (r=0.628, p<0.001). The most significant positive correlation has been found between EtCO2 and CImax (r=0.805, p<0.001) (Fig 1).

**Conclusions:** Evaluable echocardiographic records were reached in most of the patients. EtCO2 positively correlated with all parameters under consideration, while the strongest correlation was found between CImax and EtCO2. Therefore, CImax is a candidate parameter for real-time monitoring of haemodynamic efficacy of chest compressions during CPR.

**Fig. 1 (abstract P184).**
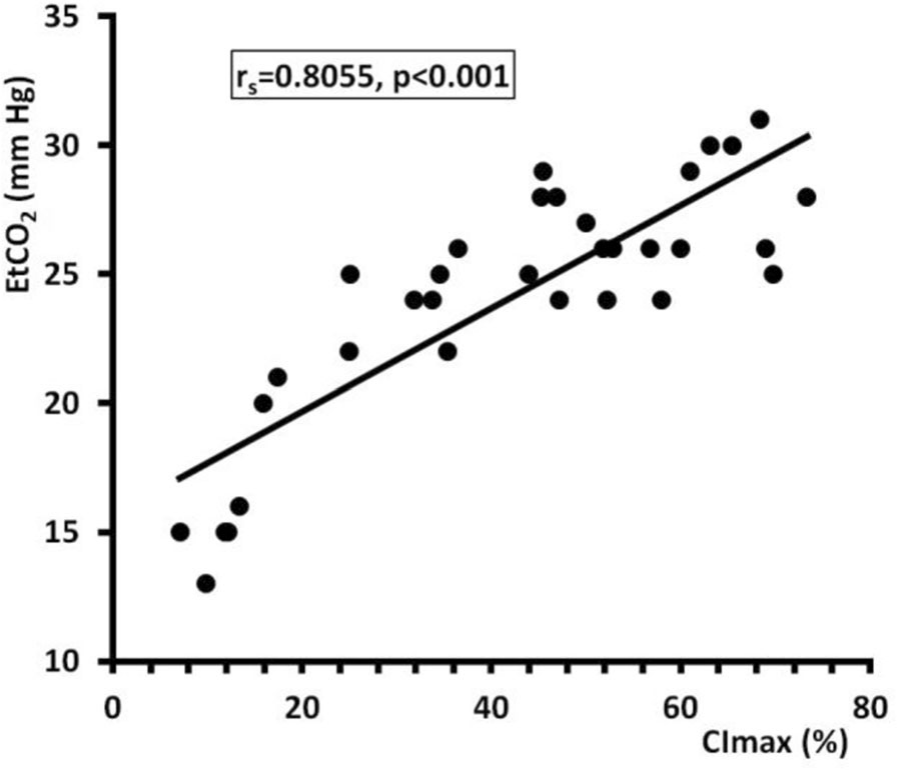
Correåation between CImax and EtCO2

### P185 Table cut-off values of time interval between witness and first CPR for neurologically favourable 1-m survival in CC-only and CC+MMV first CPR groups

#### H Inaba, H Kurosaki, K Takada, A Yamashita, Y Tanaka

##### Kanazawa University Graduate School of Medicine, Department of Circulatory Emergency and Emergency Medical Science, Kanazawa, Japan

**Introduction:** Time interval between witness and first CPR (Bystander- or EMS-performed CPR, whichever performed earlier) is known to be one of critical time factors associated with survival from out-of-hospital cardiac arrest (OHCA). This study aimed to compare cut-off values of this time interval for neurologically favourable one-month survival between bystander-witnessed OHCA groups receiving chest compression-only (CC-only) and chest compression + ventilation (CC+V )first CPR.

**Methods:** From nationwide OHCA database during 2014-2016, we extracted 82,888 bystander-witnessed OHCA cases. The cut-off values for neurologically favourable one-month survival were determined using Yoden index in the two groups receiving CC-only and CC+V first CPR before and after propensity-score matching including characteristics of OHCA associated with bystander CPR.

**Results:** The matching procedure did not alter the cut-off values (Table 1). The values were 3 and 7 min in CC-only and CC+V first CPR groups, respectively. When analysed for subgroups, the difference in cut-off value was largest in subgroup of presumed non-cardiac aetiology: 3 vs. 8 min. A hazard model disclosed that effect of first CPR on survival was more largely diminished by delay in start of time first CPR in CC-only group than a in CC+V group: hazard ratio (95% CI), 1.89 (1.84-1.94). The overall survival rates (8.9% before matching and 8.6% after matching) in CC-only group were higher than those in CC+V group (5.6% and 6.2%). However odds ratio (OR) was small when adjusted by other factors associated with survival: adjusted OR (95% CI), 1.23 (1.15-1.32) before matching, 1.13 (1.09-1.22) after matching.

**Conclusions:** The effect of CC-only CPR on survival after OHCA was more largely diminished by delay in initiation than that of CC+ V CPR.

**Table 1 (abstract P185).**
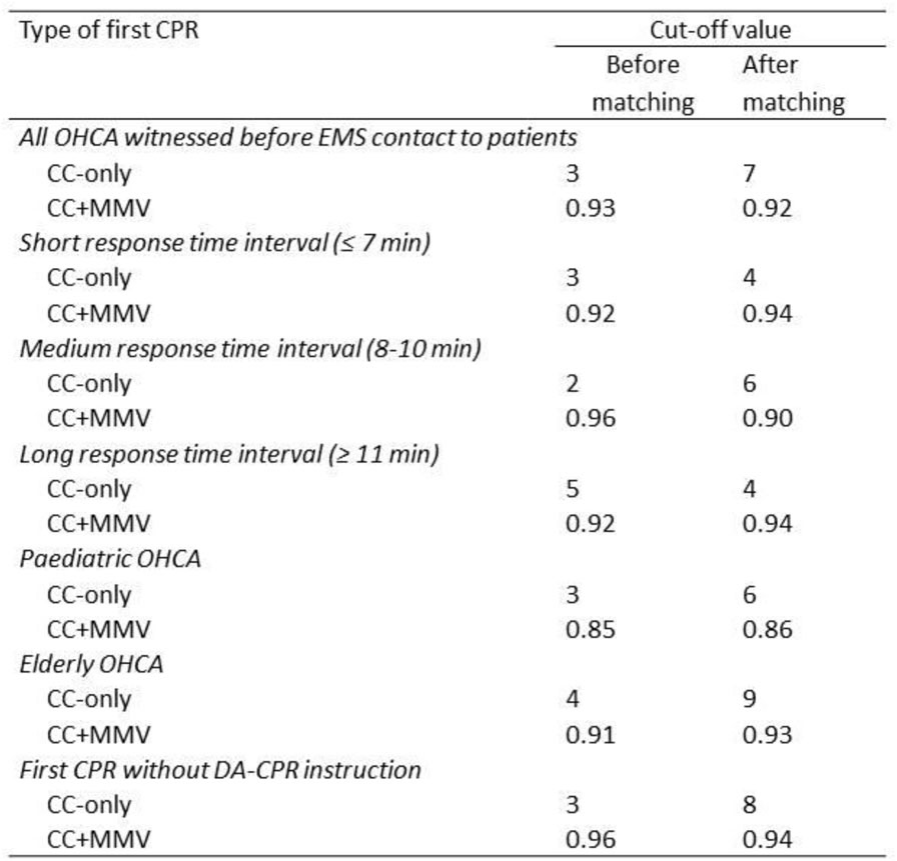
Cut-off values of time interval between witness and first CPR for neurologically favourable 1-M survival in CC-only and CC+MMV first CPR groups

### P186 Post cardiac arrest management

#### S Chaudhry, M Ranganathan, M Bishop

##### South Warwickshire NHS Foundation Trust, Anaesthetics and Intensive Care Unit, Warwick, United Kingdom

**Introduction:** The UK Resuscitation Council has set out guidelines for management of patients post cardiac arrest [1]. This is in line with European Resuscitation Council guideline. We set out to find if we are following the guideline.

**Methods:** We did a retrospective audit over the course of 2 years looking at the data of 24 patients who had in hospital and/or out of hospital cardiac arrest and after the return of spontaneous circulation were admitted to the Intensive Care Unit (ICU). We focused on whether the care they received was as per the standards set by the UK Resuscitation Council.

**Results:** We had 11 in the hospital and 13 out of hospital cardiac arrests; 11 patients had less than 10 minutes of CPR, 9 had more than 10 minutes CPR and 4 patients the data was not recorded; 16 patients needed more than 61 minutes to reach from the site of arrest to the ICU. The partial pressure of carbon dioxide was >5.5 Kpa in 11 patients at two or more occasions. Target MAP was not documented in 9 patients; blood sugar target was not documented in 15 patients and was not maintained within limits in 5 patients. Target temperature was not documented in 10 patients. The withdrawal of treatment was not delayed for 72 hours in 1 patient out of 24. In 4 patients neurological tests were not documented. Multimodal assessment tools were not used in 1 patient. Electroencephalography and serum Neuron Specific Enolase were not used to diagnose brain deaths as they were not available at our trust. 11 patients were discharged, 12 died in the ICU and 1 died in hospital after discharge from ICU.

**Conclusions:** The audit reflected our local practice and showed that our mortality was in line with the acceptable limits; poor documentation of plan of care which posed problems in analyzing the care that these patients received; some of the parameters were not being maintained as set by UK Resuscitation guideline.

**Reference**

1. Nolan JP et al. Advance life support resuscitation council UK seventh edition 2016. Post-resuscitation care; 13: p 153 - 162.

### P187 Chest compression quality depending on provider position during intra-hospital transportation

#### G Jansen^1^, K Kipker^2^, E Latka^2^, D Marx^3^, R Borgstedt^1^, S Rehberg^1^

##### ^1^Evangelisches Klinikum Bethel, Anaesthesiology, Intensive Care and Emergency Medicine, Bielefeld, Germany; ^2^Studieninstitut Ostwestfalen-Lippe, Fachbereich Medizin und Rettungswesen, Bielefeld, Germany; ^3^Medi-Learn GbR, Kiel, Germany

**Introduction:** High-quality chest compressions (CC) with minimized interruptions are one of the most essential prerequisites for an optimal outcome of resuscitation. Therapy of reversible causes of cardiac arrest often requires intra-hospital transportation (IHT) during ongoing CPR. The present study investigated CC quality during transportation depending on the position of the provider.

**Methods:** 20 paramedics were enrolled into a manikin study with four groups: a reference group with the provider kneeling beside manikin on the floor (group 1), and 3 groups performing CC during a simulated IHT of 100 meters: walking next to the bed (group 2), kneeling beside the patient in bed (group 3, Fig. 1) or squatting above the patient in bed (group 4, Figure 2). Indicators of CC quality were measured as defined in the ERC Guidelines 2015 (pressure point and depth, compression frequency, complete relief, sufficient pressure depth) [1]. All 20 paramedics performed CC during each scenario (group 1-4).

**Results:** There were no statistical differences in quality of CC between groups 1, 3 and 4. Notably, group 2 performed significantly worse in respect to the proportion of CC with correct pressure point (p = 0.044 vs group 1), correct CC depth (p=0.004 vs. group 1, p=0.035 vs. group 3, p=0.006 vs. group 4). The results are shown in Table 1.

**Conclusions:** Carrying out guideline-compliant CC [1] during IHT is feasible with multiple provider positions. Based on the present results, kneeling or squatting position next to the patient (Figure 1 and 2) is recommended, whereas “walking next to the bed” while performing CC should be avoided.

**Reference**

[1] Perkins GD et al. Resuscitation 95:81-99

**Table 1 (abstract P187).** Indicators of chest compression quality

Group	1	2	3	4
Correct pressure point (%)	99±0.6 #	79±30	85±29	86±32
Compression depth (mm)	57±11#	46± 10	54±6#	56±5#
Frequency of compressions (/min)	114±7	120±12	116±9	116±9
Compressions with complete relief (%)	86±23	68±31	74±31	70±38

# p < 0.05 vs. group 2

**Fig. 1 (abstract P187).**
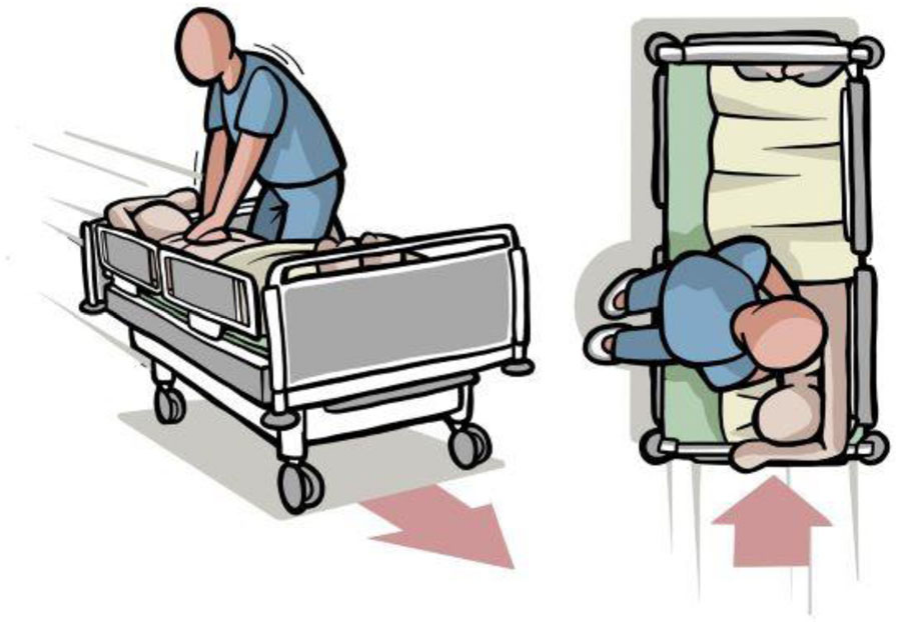
Chest compressions in kneeling position while intra-hospital transportation

**Fig. 2 (abstract P187).**
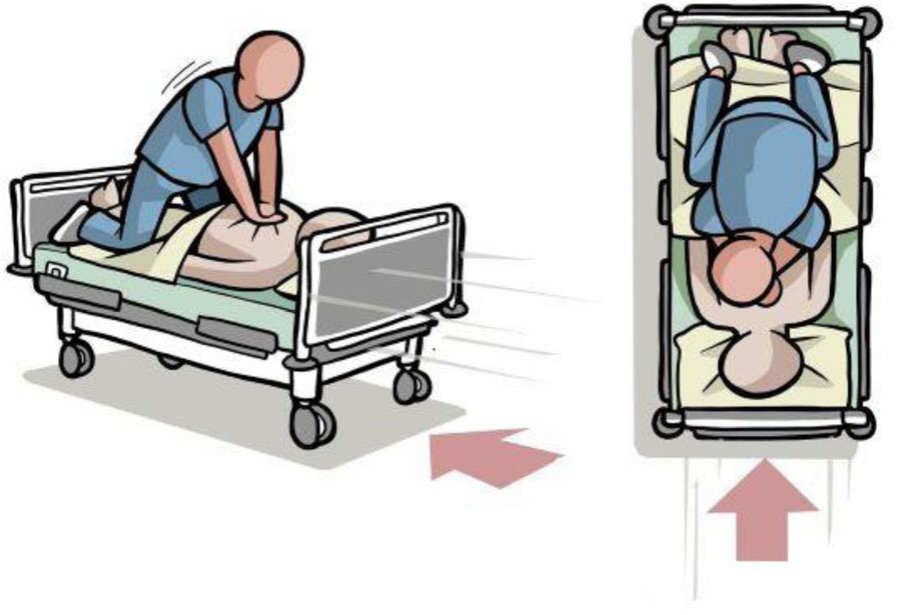
Chest compressions in squatting position while intra-hospital transportation

### P188 Ineffective ventricular and aortic compression during chest compression in CPR: resuscitative transesophageal echocardiography (TEE) findings

#### A Osman^1^, M Mohamed Sakan^2^, A Ahmad^1^, C Yen^1^, A Abdullah^3^

##### ^1^Hospital Raja Permaisuri Bainun, Emergency Department, Perak, Malaysia; ^2^Hospital Raja Permaisuri Bainun, Emergency Medicine, Perak, Malaysia; ^3^Hospital Serdang, Cardiology Department, Selangor, Malaysia

**Introduction:** In cardiac arrest, resuscitative transesophageal echocardiography (TEE) provides continuous unobstructed visualisation of the heart during each compression in real-time fashion enabling instant feedbacks to improve the quality of subsequent chest compressions. The 2015 American Heart Association only regarded resuscitative TEE as an adjunct to find treatable causes and guide treatment decisions [1].

**Methods:** We presented a case series of cardiac arrest situations incorporating the use of resuscitative TEE to review the effectiveness of high quality chest compression following 2015 AHA recommendations.

**Results:** Four cardiac arrest patients underwent resuscitative TEE during CPR. Hands were placed at lower half of sternum and compressed to the depth of between 5 to 6cm. Mid esophageal four chamber view (to visualise left ventricular compression) and mid esophageal long axis view (to visualise aortic compression) were reviewed. In 3 out of 4 cases, chest compressions led to aortic outflow tract (LVOT) obstruction necessitating corrective hand relocations.

**Conclusions:** Chest compression is a blind intervention and external chest landmarks do not always correlate with the location of left ventricle [2]. Maximal compression at the recommended lower half of sternum might result in compression of the LVOT. Resuscitative TEE is a robust tool that should be equipped in centres that strive for better outcomes among cardiac arrest patients.

**References**

1. Brooks SC et al, Circulation. 2015;132:S436-S443

2. Qvigstad E et al, Resuscitation 2013. https://doi.org/10.1016/j.resuscitation.2013.03.010

### P189 Abnormal movements but not agonal breathing is a concomitant sign associated with shockable initial rhythm and better outcomes in “sudden” cardiac arrest

#### H Inaba, H Kurosaki, K Takada, A Yamashita, Y Tanaka

##### Kanazawa University Grauate School of Medicine, Department of Circulatory Emergency and Emergency Medical Science, Kanazawa, Japan

**Introduction:** Emergency medical service (EMS) personnel may detect sudden onset of concomitant signs in EMS-witnessed out-of-hospital cardiac arrest (OHCA). This study aimed to investigate the incidences of concomitant signs and their associations with characteristics and outcomes of EMS-witnessed “sudden” OHCAs.

**Methods:** In addition to standard recommendation, EMS prospectively recorded concomitant signs which lead to start of advance life support in 385 cases with EMS-witnessed out-of-hospital cardiac arrest (OHCA) during the period of April 2012 to March 2018. Detailed database was completed after additional interview to EMS personnel. After excluding 268 cases with impending cardiac arrest, 117 cases were analysed.

**Results:** In addition to common and standard signs for cardiac arrest, the following signs were recorded as a concomitant signs which lead to start¡¡of advanced life support: abnormal movements of extremities and jaw which mimic tonic convulsion (17.9%), abnormal eye movement or position (3.4%), shout or groan (5.1%) and vomiting (5.1%). Agonal breathing was recorded in 29.1% (34/117) of all EMS-witnessed “sudden” OHCA cases and after cease of abnormal movements in 17.4% (4/22) of cases with abnormal movements. While abnormal movements were significantly associated with shockable initial rhythm (unadjusted OR; 95% CI, 4.39;1.56-12.3) and better neurologically favourable one-year survival (5.11;1.87¨C14.0), agonal breathing was not associated with shockable initial rhythm (0.59;0.22-1.63) or neurologically favourable survival (0.45;0.14-1.44). Multivariable analysis including all concomitant signs and agonal breathing confirmed the results of univariate analyses: adjusted OR (95% CI) of abnormal movement, 5.45 (1.82-16.7) for shockable rhythm, 4.16 (1.40-12.3).

**Conclusions:** Abnormal movements mimicking tonic convulsion are likely to be a concomitant sign associated with shockable initial rhythm and better outcomes in “sudden” cardiac arrest.

### P190 Introduction of a treatment bundle improves post-cardiac arrest care by reducing variation in practice

#### C Platt, P Hampshire

##### Royal Liverpool University Hospital, Critical Care Department, Liverpool, United Kingdom

**Introduction:** This retrospective audit aimed to assess adherence to the post-cardiac arrest care bundle utilised by our intensive care unit (ICU). Introduced in 2017, following publication of International Liaison Committee on Resuscitation (ILCOR) guidance[1], the care bundle was designed to standardise care for this patient cohort which previously often varied according to the responsible clinician.

**Methods:** A retrospective review of clinical notes was undertaken for patients admitted to ICU following return of spontaneous circulation but whom remained comatose. This audit encompassed three-month periods before and after introduction of the care bundle in October 2017. Audit standards were assigned from target parameters documented in the bundle and reflected guidance from the Cheshire and Merseyside Critical Care Network.

**Results:** 39 patients were included in our audit; 15 admitted prior to and 24 admitted following implementation of the care bundle. In patients whom targeted temperature management was indicated, improved adherence to thermoregulation between 34-36°C was observed (50 vs 71%). Significant improvements were since in the observance to target values for oxygen saturation (0 vs 70.8%, p=0.0023) and mean arterial pressure (20 vs 95.8%, p<0.0001) following the introduction of the care bundle. Improved observance of ventilation targets was also seen; maintenance of P_a_CO_2_>4.5 kPa (40 vs 75%, p=0.0441) and tidal volumes <8 ml/kg ideal body weight (0 to 37.5%, p=0.0069).

**Conclusions:** The introduction of a post-cardiac arrest care bundle in our ICU has improved care by providing discrete physiological targets to guide nursing staff and standardising management between clinicians. Variations in care are associated with poorer patient outcomes [2] and introduction of this bundle has reduced disparities in practice.

**References**

1. Nolan JP et al. Resuscitation 95:202-222, 2015.

2. Peterson ED et al. JAMA 295:1912-1920, 2006.

### P191 Cardiac causes and outcome of out-of-hospital cardiac arrest – single centre experience

#### P Pekic^1^, M Bura^2^, N Maric^3^

##### ^1^University Hospital “Sveti Duh”, Department for cardiovascular disease - Cardiac intensive care and arrhythmology unit, Zagreb, Croatia; ^2^Neuropsychiatric hospital “dr. Ivan Barbot”, Internal Medicine, Popovača, Croatia; ^3^University Hospital “Sveti Duh”, Intensive Care Unit, Zagreb, Croatia

**Introduction:** Out-of-hospital cardiac arrest (OHCA) is a major health problem worldwide with an annual incidence of approximately 9,000 in Croatia. Cardiac causes of OHCA can be attributable to a wide array of cardiac diseases and reported survival rate is low in spite of advances in resuscitation and EMS services.

**Methods:** Single-centre retrospective study analyzed outcomes of 42 OHCA patients admitted to cardiac ICU between 2010.-2015. We studied demographic data, initial rhythm, type of CPR, comorbidities and various post admission diagnostic findings in order to identify their impact on survival.

**Results:** OHCA comprised 0,5% of all admissions. Mean LOS was 8.24 days (0-64). Mean age was 65,4y (29-90), M: F ratio 29:13 and bystander CPR was performed in only 19% OHCA patients. The most common initial rhythm was VF (45.2%), followed by VT (16.6%), PEA was found in 2,9% and asystole in 1.9% of pt More than half of pt received adrenalin (55%) and defibrillation (57%) and only 17% required a temporary pacemaker. 38% of pt had an ECG consistent with MI after ROSC, 19% underwent coronary angiography resulting in PCI in 75% of cases. In 5 pt (12%) therapeutic hypothermia protocol was performed. Most OHCA pt had hypertension (60%) and hyperlipidaemia (69%) as the most common risk factors followed by cardiomyopathy (33%), diabetes (29%) and CAD (21%). Only 9% had a preexisting significant valvular disease and the rest were extracardial comorbidities: chronic renal disease (17%), COPD (9%) and cerebrovascular disease (9%). 14 patients survived (33%) and GCS on admission was the only significant impact factor on survival along with comorbidities (mean GSC was 6 in survivors vs. 3 in deceased). Interestingly, age, initial rhythm, troponin I level, pH and therapeutic hypothermia had no impact on survival.

**Conclusions:** Our data demonstrate the importance of early on-site resuscitation as the most important factor of neuroprotection and outcome and puts an emphasis on the importance of CPR education for layman population.

### P192 Prediction of acute coronary ischaemia and angiographic findings in patients with out-of-hospital cardiac arrest

#### J Higny^1^, A Guédès^2^, C Hanet^2^, V Dangoisse^2^, L Gabriel^2^, J Jamart^3^, C De Meester De Ravenstein^4^, E Schroeder^2^

##### ^1^CHU UCL NAMUR, Pathologie Cardiovasculaire, Soins Intensifs, Yvoir, Belgium; ^2^CHU UCL NAMUR, Pathologie Cardiovasculaire, Yvoir, Belgium; ^3^CHU UCL NAMUR, Unité de Support Scientifique, Yvoir, Belgium; ^4^Pôle de Recherche Cardiovasculaire (CARD), Institut de Recherche Expérimentale et Clinique (IREC), Cliniques Universitaires Saint-Luc, Bruxelles, Belgium

**Introduction:** Coronary artery disease (CAD) is the leading cause of out-of-hospital cardiac arrest (OHCA). However, diagnosis of acute coronary ischaemia (ACI) remains challenging, particularly in patients without ST-segment elevation on the post-resuscitation ECG. In this regard, a consensus statement recommends the implementation of a work-up strategy in the emergency room (ER) to exclude non-coronary causes of collapse within 2 hours.

**Methods:** Retrospective single-centre study performed on 64 consecutive patients with resuscitated OHCA who underwent a diagnostic coronary angiography (CA). We present data on coronary angiograms for patients who underwent cardiac catheterization after resuscitation. Afterwards, we sought to identify parameters associated with ACI.

**Results:** ST-segment elevation was noted in 29 patients (45%). ST-segment depression or T-wave abnormalities were noted in 35 patients (55%). Invasive coronary strategy allowed to identify an acute culprit lesion in 46 cases (72%). 29 patients with ST-segment elevation underwent an immediate angioplasty for an acute coronary occlusion. 17 patients without ST-segment elevation underwent an ad hoc percutaneous coronary intervention for a critical lesion. Stable CAD was found in 9 cases (14%) and a normal angiogram was found in only 9 cases (14%) (Figure 1). The independent predictors of ACI were convertible rhythm (OR 16.02; 95% CI 4.48-57.29), personal history of CAD (OR 15.12; 95% CI 4.19-54.53) and presence of at least 2 cardiovascular risk factors (CVRF) (OR 10.68; 95% CI 2.55-44.74) (Table 1).

**Conclusions:** ACI was the leading precipitant of collapse. ST-segment elevation was highly predictive of coronary occlusion. In addition, a culprit coronary lesion was identified in nearly 50% of patients undergoing CA despite the lack of ST-segment elevation. Finally, our findings suggest that the identification of risk criteria may help to improve the recognition of ACI after OHCA.

**Table 1 (abstract P192).** Univariate and multivariate analysis for the prediction of ACI in patients with OHCA

Variables	Univariate analysis	P-value	Multivariate analysis	P-value
	OR (CI 95%)		OR (CI 95%)	
Convertible rhythm	9.22 (3.64-23.31)	<0.001	16.02 (4.48-57.29)	<0.001
Haemodynamic instability	0.7 (0.31-1.58)	0.39		
Daytime cardiac arrest	1.56 (0.73-3.32)	0.25		
Personal history of CAD	3.47 (1.61-7.47)	0.001	15.12 (4.19-54.53)	<0.001
Age > 65 years	1.07 (0.51-2.23)	0.87		
Male gender	1.94 (0.84-4.51)	0.12		
Diabetes	2.57 (1.22-5.45)	0.014		
Hypertension	1.7 (0.81-3.57)	0.16		
Dyslipidaemia	2.94 (1.37-6.28)	0.005		
Smoking	0.97 (0.47-2.02)	0.94		
Family history of CAD	1.5 (0.64-3.5)	0.35		
≥ 2 CVRF	14.04 (3.17-62.26)	0.001	10.68 (2.55-44.74)	<0.001

**Fig. 1 (abstract P192).**
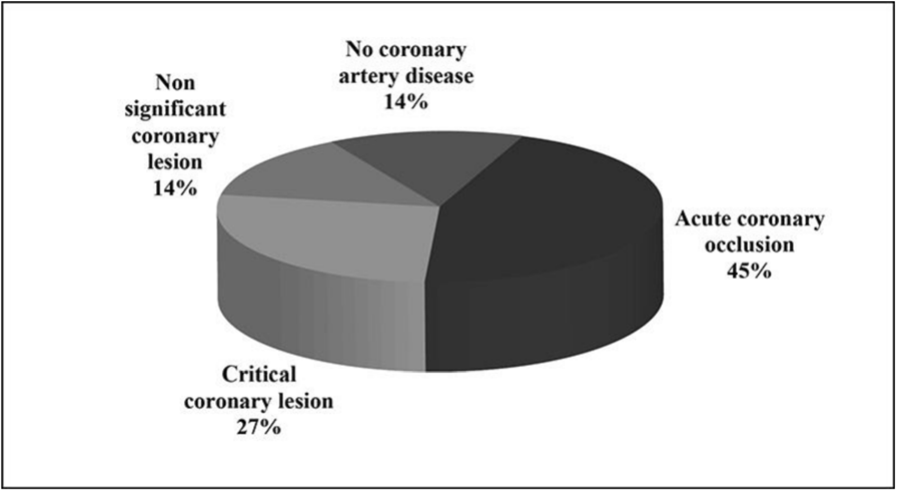
Angiographic findings in patients in patients undergoing cardiac catheterization after OHCA (n = 64)

### P193 The prediction of outcome for in-hospital cardiac arrest (PIHCA) score

#### E Piscator^1^, K Göransson^1^, S Forsberg^1^, M Bottai^2^, M Ebell^3^, J Herlitz^4^, T Djärv^1^

##### ^1^Karolinska Institutet, Center for Resuscitation Science, Department of Medicine Solna, Stockholm, Sweden; ^2^Karolinska Institutet, Unit of Biostatistics, Department of Environmental Medicine (IMM), Stockholm, Sweden; ^3^College of Public Health, University of Georgia, Department of Epidemiology and Biostatistics, Athens, United States; ^4^University of Borås and Sahlgrenska Academy, University of Gothenburg, Center of Prehospital Research, Faculty of Caring Science, Work-life and Welfare, University of Borås and Department of Molecular and Clinical Medicine, Sahlgrenska Academy, University of Gothenburg, Borås and Gothenburg, Sweden

**Introduction:** A do-not-attempt-resuscitation (DNAR) order may be issued when it is against the wishes of the patient to receive cardiopulmonary resuscitation (CPR), or when CPR is considered medically futile; that is when the chances of good outcome are minimal. A prearrest prediction tool can aid clinicians in consolidating objective findings with clinical judgement in the decision process for DNAR orders. Our aim was prediction model update of the Good Outcome Following Attempted Resuscitation score for favorable neurologic survival after in-hospital cardiac arrest (IHCA).

**Methods:** The prediction model update was based on a retrospective cohort of 717 adult IHCAs in Stockholm County 2013-2014 identified through the Swedish Cardiopulmonary Resuscitation Registry. It included redefinition and reduction of predictor variables, and addition of the predictor variable chronic co-morbidity, assessed as Charlson Comorbidity Index, to create a full model consisting of 9 predictors. The outcome was favorable neurologic survival defined as Cerebral Category Score ≤2 at discharge. The full model was recalibrated based on 1000-bootstrap resampling calibration. Model performance was evaluated with the area under the receiver operating curve (AUROC), calibration and classification accuracy with a cutoff of 3% likelihood of favorable neurologic survival.

**Results:** We call the updated prediction model the Prediction of outcome for In-Hospital Cardiac Arrest (PIHCA) score. It had an AUROC of 0.81 (95% CI:0.81 to 0.81) and showed evidence of good calibration, see calibration plot in Figure. Predictive value for classification into <3% likelihood of favorable neurologic survival was 97.4%. False classification into < 3% likelihood of favorable neurologic survival was 0.57%.

**Conclusions:** The PHICA score has potential to be used as an aid for objective prearrest assessment of the chance of favorable neurologic survival after IHCA, as part of decision making for a DNAR order.

**Fig. 1 (abstract P193).**
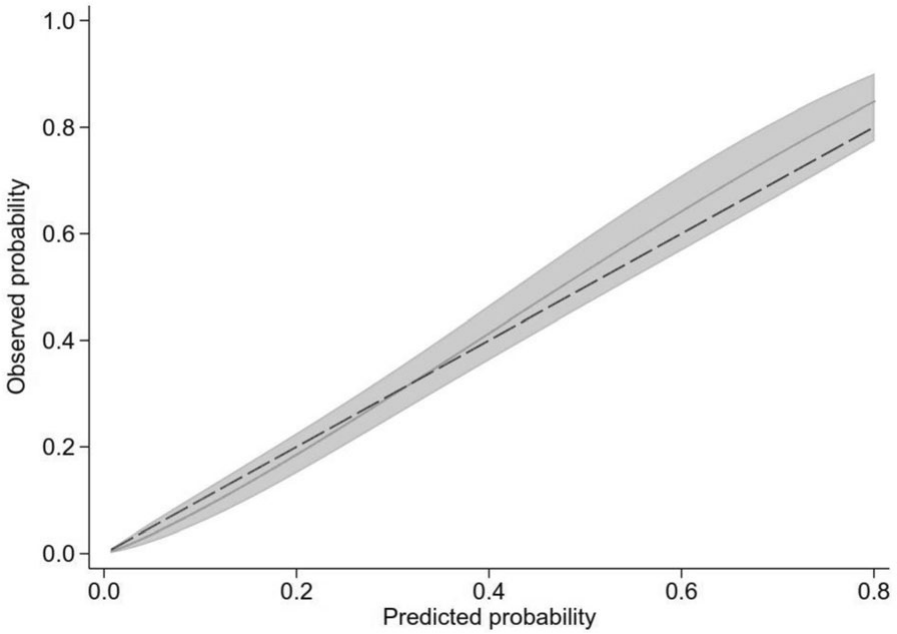
Calibration plot for the Prediction of outcome for In-Hospital Cardiac Arrest (PIHCA) score

### P194 Extracorporeal membrane oxygenation and cytokine adsorption in post-resuscitation disease

#### P Morimont, T Desaive, T Amand, A Rego, J Koch, M Lagny, S Habran, B Lambermont

##### University Hospital of Liege, Medical Intensive Care, Liege, Belgium

**Introduction:** Prognosis of survival in patients with cardiac arrest remains poor. During and after cardiopulmonary resuscitation, pathophysiological disturbances in relation with a cytokine storm, are described as “post-resuscitation” disease like a combination of cardiogenic and vasodilatory shocks. Veno-arterial extracorporeal membrane oxygenation (VA ECMO) allows to restore adequate perfusion but little is known about its effect on left ventricular (LV) function and about the role of cytokines.

**Methods:** This study was performed in an experimental model of cardiac arrest performed in 3 groups of 3 anesthetized and mechanically ventilated pigs. Cardiac arrest was obtained by application of electrical current to epicardium inducing ventricular fibrillation. After a no-flow period of 5 minutes, medical resuscitation with catecholamines and vasopressors was performed in “CONTROL” group while VA ECMO was started in “ECMO” group and VA ECMO in combination with CYTOSORB (extracorporeal blood purification therapy designed to reduce excessive levels of inflammatory mediators such as cytokines) was started in “ECMO-CYTO” group. LV function was assessed with transthoracic echocardiography and arterial pressure with aortic pressure catheter.

**Results:** Hemodynamic stability was obtained after 22 ± 6 and 24 ± 7 minutes in ECMO and ECMO-CYTO groups, respectively. No return of spontaneous circulation was observed in CONTROL group. At 15 minutes following cardiac arrest, LV area fractional change on short axis was normalized in ECMO and ECMO-CYTO groups (31± 3 and 34 ± 4 %, respectively). Vasopressor requirements were significantly lower in ECMO-CYTO group than in ECMO group.

**Conclusions:** After cardiac arrest (no-flow) of 5 minutes duration, VA ECMO allowed complete LV recovery and hemodynamic stability within 30 minutes of “post-resuscitation” disease. CYTOSORB added to VA ECMO could contribute to reduce post-resuscitation vasodilatation.

### P195 Impact of rapid response car system on ECMO in out-of-hospital cardiac arrest: a retrospective cohort study

#### M Nasu^1^, R Sato^2^, K Takahashi^1^, Y Kitai^1^, Y Kitahara^1^, T Hozumi^1^, H Fukui^1^, T Yonemori^1^

##### ^1^Urasoe General Hospital, Emergency and Critical Care, Urasoe, Japan; ^2^Department of Internal Medicine, John A. Burns School of Medicine, University of Hawaii at Manoa, Department of Internal Medicine, Hawaii, United States

**Introduction:** Extracorporeal life support (ECLS) has been reported to be more effective than conventional cardio-pulmonary resuscitation (CPR). In ECLS, a shorter time from arrival to implantation of extracorporeal membrane oxygenation (ECMO; door-to-ECMO) time has been reported to be associated with better survival rates. This study aimed to examine the impact of the physician-based emergency medical services (P-EMS) using a rapid response car (RRC) on door-to-ECMO time in patients with out-of-hospital cardiac arrest (OHCA).

**Methods:** In this retrospective cohort study, adult patients with OHCA who were admitted to a Japanese tertiary care hospital from April 2012 to November 2018 and underwent veno-arterial ECMO were included. Patients were either transferred by emergency medical service (EMS only group) or with physician using RRC (RRC group). Primary outcome was door-to-ECMO time. Secondary outcome was onset-to-ECMO time, 24-hour survival, 30-day survival and neurologically favorable outcome. Wilcoxon rank-sum test was used to compare the outcome between the two groups.

**Results:** A total of 46 patients were included in this study, and outcome data were available for all patients. The door-to-ECMO time was significantly shorter in the RRC group than in the EMS only group (median, 20 min vs. 33 min; P =0.004). Additionally, the onset-to-ECMO time was significantly shorter (median, 50 min vs. 60 min; P =0.005). There was no significant difference in the 24-hour survival, 30-day survival rates and neurologically favorable outcome.

**Conclusions:** The physician-based RRC system was associated with a shorter door-to-ECMO time. Combination of the RRC system with ECLS may lead to better outcomes in patients with OHCA.

### P196 The evaluation of ICU outcome of extracorporeal cardiopulmonary resuscitation (ECPR) for adult patients with out-of-hospital cardiac arrest (OHCA)

#### R Hokama, S Miyakawa, M Koie, M Nakashio, J Maruyama, Y Irie, K Hoshino, H Ishikura

##### Fukuoka university hospital, Department of Emergency and Critical Care Medicine, Fukuoka city, Japan

**Introduction:** Although there have been some reports to examine the indication and prognostic factors of ECPR, the indication for ECPR has not been established in patients with OHCA. The purpose of this study was to establish prognostic factors for ECPR in adult patients with OHCA.

**Methods:** This retrospective, observational study was conducted on OHCA patients aged over 18 who were delivered to the ER from January 2001 to December 2017. And we enrolled the patients who were performed ECPR immediately after arrival and survived 24 hours or more. These patients were divided into ICU survival groups (S) and non-survival groups (NS) and evaluated of ICU outcome of ECPR. We reviewed medical records collected at the time of admission and compared demographic data and experimental data between the two groups.

**Results:** In this study, 70 patients were included, 22 patients (31.4%) were S (median age 55 years [IQR, 42-64 years]) and 48 patients were NS (median age 59 years [IQR, 53-65 years]). There was a significant difference between S and NS with the ratio of witnesses [S 21(95.5%) vs non-survivor 37(77.1%), p=0.013], the ratio of abnormal pupil diameter (5mm or more, 2mm or less)[S 7(31.8%) vs NS 36(75.0%), p<0.001] and a presence of pupillary light reflex[S 9(40.9%) vs NS 3(6.3%), p<0.001]. Multivariable logistic regression analysis performed for 5 explanatory variables (pupillary light reflex, dilated pupils (5mm or more), normal pupil diameter (3-4mm), the witness, and ventricular fibrillation), indicated pupillary light reflex (OR, 6.1; 95% CI, 1.14-32.50, p=0.03) and dilated pupils (OR, 0.06; 95%CI, 0.004-0.889, p=0.04) to be an independent prognostic factor.

**Conclusions:** In this study, we suggested that pupil findings on hospital arrival might become predictive factors of ECPR for adult patients with OHCA.

### P197 Spontaneous CPR simulations in the ICU

#### N Tabak, Y Polishuk, E Kishinevsky, G Bregman

##### Kaplan Medical Center, ICU, Rehovot, Israel

**Introduction:** Quick identification and professional treatment of conditions requiring CPR must be practiced by all staff, with refresher simulations providing updated knowledge. Spontaneous CPR simulations lead to faster staff response times, improves team work, and increases the effectiveness of treatment during real CPR situations. Providing refresher resuscitation courses are not enough, and lack the advantages of spontaneous simulations, specifically practicing performing CPR.

**Methods:** The simulations all based on American Heart Association protocols and based on clinical situations of typical ICU patients. The simulations take place in a room with specialized equipment with feedback given immediately to participants. The participants of the simulations are Nursing staff and Doctors that are currently on shift, plus trainees and medical students. The simulations are run by experienced staff members. The simulations are held once a week covering all shifts. Judgement of staff performance uses a tool based on AHA protocols and checks effectiveness of CPR management, specifically response time, correct usage of equipment and devices, performance of chest compressions, intubation, drug management and team work.

**Results:** During the period between 03/17 - 10/18 47 spontaneous CPR simulations were performed in the ICU, the majority on the morning shift. During this period at least 57% of Nursing staff and 66% of Medical staff practiced CPR simulations at least twice, with an overall improvement in performance of 25%, improved identification of conditions requiring CPR, quicker response times, use of appropriate equipment and increased effective team work.

**Conclusions:** Improving resuscitation skills needs practice and team work, a continuous process which needs to include all Nursing and Medical staff and involves repeat CPR simulations. There is a need to expand simulations to cover all shifts. Staff responded positively to this process and reported increased confidence when managing real life CPR situations.

### P198 Optimized infrastructure and regular training reduces time-to-extracorporeal life support for in hospital cardiac arrest

#### M Suverein^1^, A Bruekers^1^, T Delnoij^1^, R Lorusso^2^, M Bol^1^, J Sels^1^, P Weerwind^2^, P Roekaerts^1^, J Maessen^2^, M Van de Poll^1^

##### ^1^Maastricht UMC, Intensive Care, Maastricht, Netherlands; ^2^Maastricht UMC, Cardiothoracic Surgery, Maastricht, Netherlands

**Introduction:** ECPR requires rapid activation of a multidisciplinary team and a well-organized ECPR infrastructure [1]. In May 2017, we started optimising our ECPR infrastructure for out-of-hospital cardiac arrest by introducing a dedicated ECPR cart, regular training sessions and simulation cases, and by evaluating all procedures. This infrastructure is also used for in-hospital cardiac arrest (IHCA). The objective of this retrospective analysis was to determine whether these changes had an effect on the time to extracorporeal life support (ECLS) in IHCA as well.

**Methods:** A single-centre, retrospective review was conducted a year after the start of training. All adult patients (≥18 years) with a witnessed, refractory IHCA who received ECPR from 2013 to 2018 were included. The primary outcome was time-to-ECLS, defined as time from start of arrest to adequate flow on ECLS. Data are presented as median [IQR]. Statistical analysis was performed using a Mann-Whitney U test.

**Results:** In the first period (2013 – April 2017), 13 patients received ECPR with a median age of 62 [60-67], 69.2% were male and with a Charlson Comorbidity Index (CCI) of 2.5 [2.0-3.0]. In the second period (May 2017 – May 2018), 4 patients received ECPR, with a median age of 53 [33-60], 50% were male and with a CCI of 1.5 [0.5-1.5]. The time-to-ECLS was significantly reduced from 40 minutes [25-45] to 27 minutes [22-33] (p = 0.019) (Fig 1). Successful weaning of mechanical circulatory support remained similar for both time periods (1st 46.2 % vs. 2nd 50%).

**Conclusions:** In conclusion, optimisation of the ECPR infrastructure significantly reduced time-to-ECLS, which may ultimately improve clinical outcome after ECPR for IHCA.

**Reference**

[1] Swol, J., et al. (2016). Perfusion 31(3): 182-188.

**Fig. 1 (abstract P198).**
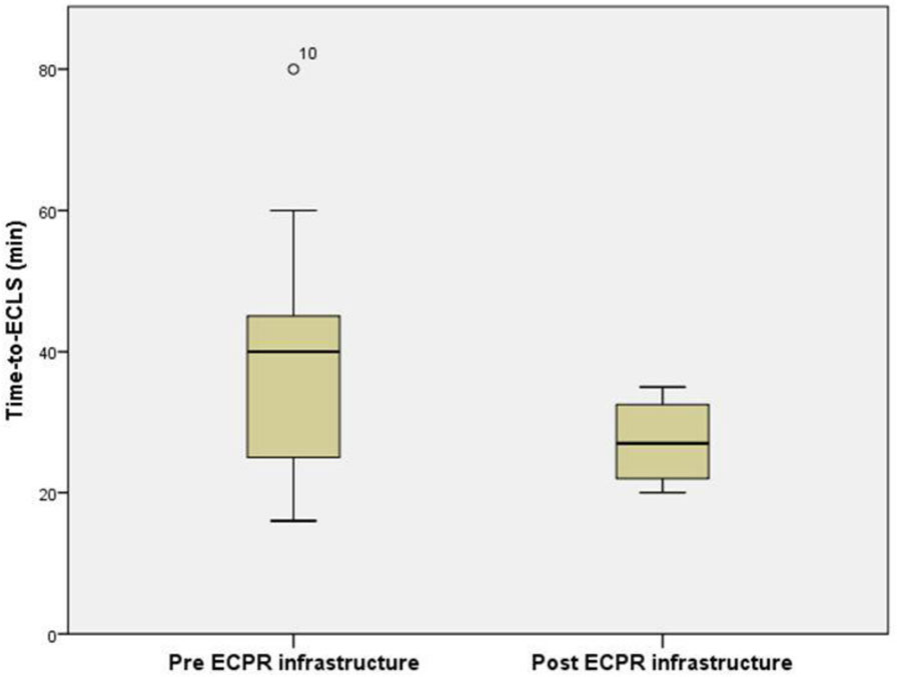
Boxplot of time-to-ECLS (min) in the first and second period (p = 0,019)

### P199 Interest of the “high fidelity” medical simulation as a pedagogical tool in training of medical residents

#### W Sellami, I Ben mrad, Z Hajjej, M Chniti, I Labbene, M Ferjani

##### Department of critical care medicine and anesthesiology Military Hospital, Tunis, Tunisia

**Introduction:** Medical simulation has become an essential teaching method for all health professionals. It is effective in terms of learning and improves skills. However, most studies have focused only on immediate benefits and short-term retention of skills. Main objective: To study the interest and the educational contribution in the short and medium term of medical simulation compared to a classical training.

**Methods:** Cohort, prospective, observational, single-center, randomized study with control group including 30 residents (20 in anesthesia resuscitation and 10 in emergency medicine). All benefited from a theoretical training with a reminder of the latest recommendations on the management of cardiac arrest and anaphylactic shock. They were randomized into 2 groups and received practical training on a high-fidelity simulator for the management of either cardiac arrest (ACC group) or anaphylactic shock (CA group). Each group was evaluated at 6 weeks (T0) and at 6 months on two scenarios: refractory ventricular fibrillation (FV) scored on 20 points and grade 3 anaphylactic reaction (RA3) scored on 30 points. Each group served as the control group for the pathology in which they did not receive specific simulator training. The results are expressed on average with their standard deviations with “p” <0.05.

**Results:** 30 residents were included and randomized into 2 groups of 15. The ACC and CA groups scored significantly higher in their respective 2-time scenarios: T0 (FV: ACC = 18.90 [17.8-20] vs CA = 9.35 [6.66-12.04]; RA3: CA = 26.5 [23.00-30] vs ACC = 26.28 [9.56-23]), T1 (FV: 18.0 [16.01-20] vs 15.03 [12,33-17,73] RA3: 28.50 [27-30] vs 21.3 [15.5-27.06]).

**Conclusions:** Simulator-trained participants have significantly better results than untrained ones, at both 6 weeks and 6 months.

### P200 Benefits of simulation as a teaching tool in postgraduate education in emergency medicine “cardiac arrest scenario”

#### M Khrouf, H Sandid, K Hraiech, F Boukadida, Y Bel haj, Z Mezgar, M Methamem

##### Hopitall Farhat Hached 4000 Rue Ibn AL Jazzar, Sousse, Tunisia

**Introduction:** Simulation is a tool for improving the quality and safety of care, and its recognized as an essential method of evidence-based education. Emergency Medicine is a discipline in which there is a constant concern for the safety of patients. The emergency physician is often called upon to take charge of critical situations that use knowledge, know-how and knowledge as skills that must be mastered and whose theoretical learning alone is insufficient.

**Methods:** It´s a prospective study including residents in emergency medicine performing their specialty courses in emergency services and emergency medical assistance in the region of Sousse from January to June 2018. They were randomized into two groups: the one benefiting from a traditional education and the other from an education based on simulation sessions. The chosen scenario was the management of a cardiac arrest. A pre-test and a post-test were performed in both groups.

**Results:** We included 30 emergency residents who did not receive specialized training in the management of cardiac arrest, there was a female predominance with an average age of 27, there was no significant difference regarding the pretest between the two groups with 10.08 There was no significant difference with respect to the pre-test score between the two groups 10.08 ± 2.7 / 20 for the control group versus 10.34 ± 3.3 / 20 for the simulation group. There was a significant progression after the course with an average post-test score of 13.87 ± 1.8 in the simulation group while this score was 11.94 ± 2.3 in the control group with a statistically significant difference (p <0.001).

**Conclusions:** Simulation learning has led to a better acquisition of cognitive knowledge by learners. The simulation is not intended to replace bed-based teaching, nor theoretical or faculty teaching, but it is an essential complement . In Tunisia, the simulation must continue its current integration in the initial and continuous training of doctors.

### P201 mTOR mediates neuronal death following transient global cerebral ischemia in the striatum of chronic high-fat diet-induced obese gerbils

#### JK Seo^1^, HJ Lim^1^, JB Moon^1^, TG Ohk^1^, MC Shin^1^, KE Kim^1^, MH Won^2^, JH Cho^3^

##### ^1^Kangwon National University, Emergency Medicine, Chuncheonsi, South Korea; ^2^Kangwon National University, Neurobiology, Chuncheonsi, South Korea; ^3^Kangwon National University, Department of Emergency Medicine, Chuncheonsi, South Korea

**Introduction:** Recent studies have shown that obesity and its related metabolic dysfunction exacerbates outcomes of ischemic brain injuries in some brain areas, such as the hippocampus and cerebral cortex when subjected to transient global cerebral ischemia (tGCI). However, the impact of obesity in the striatum after tGCI has not yet been addressed. The objective of this study was to investigate the effects of obesity on tGCI-induced neuronal damage and inflammation in the striatum and to examine the role of mTOR which is involved in the pathogenesis of metabolic and neurological diseases.

**Methods:** Gerbils were fed with a normal diet (ND) or high-fat diet (HFD) for 12 weeks and then subjected to 5 min of tGCI. HFD-fed gerbils showed the significant increase in body weight, blood glucose level, serum triglycerides, total cholesterol, and low-density lipoprotein cholesterol without affecting food intake.

**Results:** In HFD-fed gerbils, neuronal loss occurred in the dorsolateral striatum 2 days after tGCI and increased neuronal loss were observed cholesterol days after tGCI; however, no neuronal loss was the in ND-fed gerbils after tGCI, as assessed by neuronal nuclear antigen immunohistochemistry and Fluoro-Jade B histofluorescence staining. The HFD-fed gerbils also showed severe activated microglia and further increased immunoreactivities and protein levels of tumor necrosis factor-alpha, interukin-1beta, mammalian target of rapamycin (mTOR) and phosphorylated-mTOR in the striatum during pre- and post-ischemic conditions compared with the ND-fed gerbils. In addition, we found that treatment with rapamycin, a mTOR inhibitor, in the HFD-fed gerbils significantly attenuated HFD-induced striatal neuronal death without changing physiological parameters.

**Conclusions:** These findings reveal that chronic HFD-induced obesity results in severe neuroinflammation and significant increase of mTOR activation, which could contribute to neuronal death in the stratum following tGCI. Abnormal mTOR activation might play a key role.

### P202 Associations between partial pressure of oxygen and neurological outcome in out-of-hospital cardiac arrest patients

#### F Ebner^1^, S Ullén^2^, A Åneman^3^, T Cronberg^4^, N Mattsson^4^, H Friberg^1^, C Hassager^5^, J Kjærgaard^5^, M Kuiper^6^, P Pelosi^7^, J Undén^1^, M Wise^8^, J Wetterslev^9^, N Nielsen^1^

##### ^1^Lund University, Helsingborg Hospital, Department of Clinical Sciences Lund, Anesthesia and Intensive Care, Helsingborg, Sweden; ^2^Skane University Hospital, Clinical Studies Sweden, Lund, Sweden; ^3^Liverpool Hospital, Department of Intensive Care, Sydney, Australia; ^4^Lund University; Skane University Hospital, Department of Clinical Sciences Lund, Neurology, Lund, Sweden; ^5^University of Copenhagen, Department of Cardiology and Department of Clinical Medicine, Rigshospitalet, Copenhagen, Denmark; ^6^Leeuwarden Medical Centrum, Intensive Care Unit, Leeuwarden, Netherlands; ^7^University of Genoa, Department of Anesthesia and Intensive Care, IRCCS San Martino Policlinico Hospital, Genoa, Italy; ^8^University Hospital of Wales, Adult Critical Care, Cardiff, United Kingdom; ^9^University of Copenhagen, Copenhagen Trial Unit, Centre for Clinical Intervention Research, Copenhagen, Denmark

**Introduction:** Exposure to hyperoxemia and hypoxemia is common in out-of-hospital cardiac arrest (OHCA) patients following return of spontaneous circulation (ROSC) but its effects on neurological outcome are uncertain and study results are inconsistent.

**Methods:** Exploratory post-hoc substudy of the Target Temperature Management (TTM) trial [1], including 939 patients after OHCA with ROSC. The association between serial arterial partial pressures of oxygen (PaO_2_) during 37 hours following ROSC and neurological outcome at 6 months, evaluated by Cerebral Performance Category (CPC), dichotomized to good (CPC 1-2) and poor (CPC 3-5), was investigated. In our analyses, we tested the association of hyperoxemia PaO_2_ > 40 kPa and hypoxemia PaO_2_ < 8 kPa, time weighted mean PaO_2_, (TWM-PaO_2_) (Fig 1), maximum PaO_2_ difference (Δ PaO_2_) and gradually increasing PaO_2_ levels (13.3 - 53.3 kPa) with poor neurological outcome. A subsequent analysis investigated the association between PaO_2_ and a biomarker of brain injury, peak serum Tau levels.

**Results:** 869 patients were eligible for analysis. 300 patients (35%) were exposed to hyperoxemia or hypoxemia after ROSC (Table 1). Our analyses did not reveal a significant association between hyperoxemia, hypoxemia, TWM-PaO_2_ exposure or Δ PaO_2_ and poor neurological outcome at 6-month follow-up after correction for co-variates (all analyses p= 0.146 - 0.847) (Fig 2). We were not able to define a PaO_2_ level associated with the onset of poor neurological outcome. Peak serum Tau levels at either 48 or 72 hours after ROSC were not associated with PaO_2_.

**Conclusions:** Hyperoxemia or hypoxemia exposure occurred in one third of the patients during the first 37 hours of hospitalization and was not significantly associated with poor neurological outcome after 6 months or with the peak s-Tau levels at either 48 or 72 hours after ROSC.

**Reference**

1. Nielsen et al. N Engl J Med. 2013;369:2197-206.

**Table 1 (abstract P202).** Outcome according to exposure groups in primary and secondary analyses

Multivariable model	OR	95% CI	p-value
Hypoxemia vs no hypoxemia*	1.13	0.66 - 1.91	0.647
Hypoxemia vs normoxemia*	1.06	0.60 - 1.85	0.847
Hyperoxemia vs no hyperoxemia*	1.28	0.86 - 1.91	0.219
Hyperoxemia vs normoxemia*	1.24	0.81 - 1.89	0.314
Δ PaO_2_**	1.01	0.99 - 1.02	0.146
PaO_2_-TWM T-1 to T36**	1.03	0.97 - 1.09	0.375
PaO_2_-TWM T-1 to T12**	1.02	0.98 - 1.05	0.288

PaO2 = arterial partial pressure of oxygen. CI = confidence Interval. TWM = time weighted mean. Δ PaO2 = maximum PaO2 difference. T = measuring time point in hours after inclusion into the TTM-trial. T -1 = first blood gas analysis after admission but before inclusion. OR < 1 indicates better outcome. *Categorical data. **Continuous data

**Fig. 1 (abstract P202).**
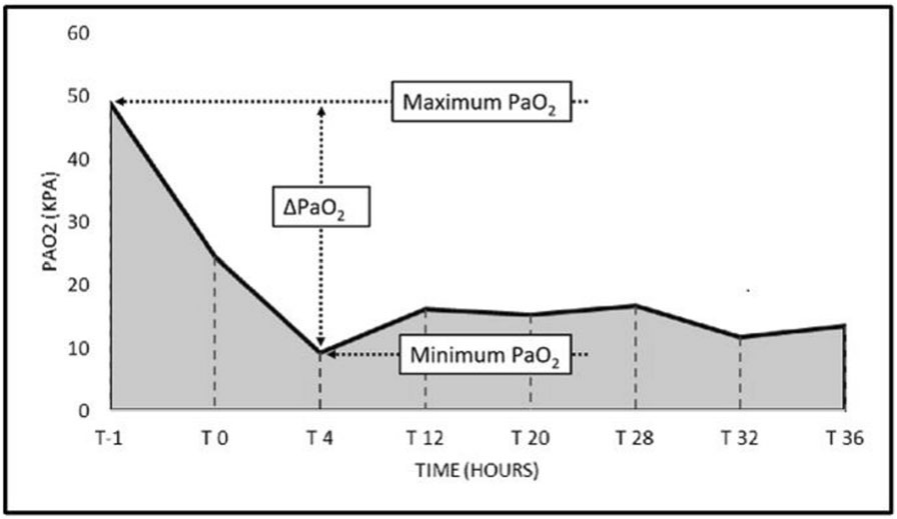
Explanatory illustration depicting the primary and secondary analyses. The grey area under the chart represents the area from which PaO2-TWM was calculated. PaO2 = partial pressure of oxygen. kPa = kilopascal. T -1 = first blood gas analysis after admission but before inclusion into the TTM-trial. T0 to T36 = time points (hours) for protocolized blood gas sampling

**Fig. 2 (abstract P202).**
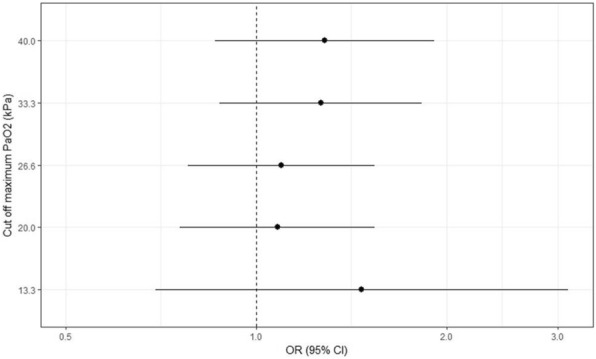
Adjusted OR´s (bullet points) with 95% CI´s (horizontal lines) for poor neurological outcome according to Cerebral Performance Category (CPC) across stepwise increasing PaO2 threshold values. OR below 1.0 indicates better outcome above the PaO2 threshold

### P203 Clinical predictors of early and late mortality after cardiac arrest: a retrospective multicenter cohort study

#### P Kurtz^1^, C Storm^2^, M Soares^3^, F Bozza^3^, U Melo^4^, D Siefeld Araya^5^, B Mazza^6^, M S. Santino^7^, JL Salluh^3^

##### ^1^Paulo Niemeyer State Brain Institute, Neuro ICU, Rio de Janeiro, Brazil; ^2^Charité – Universitätsmedizin, Berlin, Germany; ^3^D´Or Institute for Research and Education, Rio de Janeiro, Brazil; ^4^Hosp Estadual Alberto Torres, São Gonçalo, Brazil; ^5^Hospital Santa Paula, São Paulo, Brazil; ^6^Hospital Samaritano, São Paulo, Brazil; ^7^Hospital Barra d’Or, Rio de Janeiro, Brazil

**Introduction:** Data on outcome of survivors of cardiac arrest patients in low and middle-income countries is scarce. The purpose of this study was to investigate clinical predictors of hospital mortality in patients resuscitated after cardiac arrest in a large sample of Brazilian ICUs.

**Methods:** We performed a retrospective cohort study of survivors from cardiac arrest in 57 hospitals in Brazil, during 2014 and 2015. We retrieved patients’ clinical and outcome data from an electronic ICU quality registry. We used multivariable logistic regression analysis to identify factors associated with hospital mortality.

**Results:** A total of 2295 patients were included. Both patients with primary admission diagnosis of cardiac arrest (n = 800, 35%) and those that arrested on admission to the ICU (65%) were included. Median age was 67 (IQR 54 – 79) and 53% were male. Median SAPS 3 was 70 (57 – 83), SOFA was 10 (7 – 13) and hospital mortality was 83%. Among nonsurvivors, 47% died in the first 48h of ICU admission (early mortality). Only 1% received therapeutic hypothermia and 6% underwent withhold/withdrawal of life support. After adjusting for SAPS 3 and SOFA, early mortality was associated with hemodynamic compromise (systolic blood pressure <100 mmHg, lactate >4 mmol/L and use of vasopressors) and hypo or hyperthermia. Late mortality was independently associated with delayed admission to the ICU (>48h) and palliative care.

**Conclusions:** This large cohort of post cardiac arrest survivors demonstrates extremely high mortality rates and negligible implementation of hypothermia in Brazil, with half of nonsurvivors dying within 48 hours of admission with severe hemodynamic compromise and organ dysfunction. Late mortality was related to delayed admission to ICU and withdrawal/withholding measures.

### P204 Cerebral oxygenation after out-of-hospital cardiac arrest: association with neurological outcome

#### P Jakkula^1^, J Hästbacka^1^, M Reinikainen^2^, V Pettilä^1^, P Loisa^3^, M Tiainen^4^, M Bäcklund^1^, S Bendel^5^, T Birkelund^6^, R Laru-Sompa^7^, M Skrifvars^8^

##### ^1^University of Helsinki and Helsinki University Hospital, Perioperative, Intensive Care and Pain Medicine, Helsinki, Finland; ^2^North Karelia Central Hospital, Joensuu, Finland; ^3^Päijät-Häme Central Hospital, Lahti, Finland; ^4^University of Helsinki and Helsinki University Hospital, Department of Neurology, Helsinki, Finland; ^5^Kuopio University Hospital, Kuopio, Finland; ^6^Aarhus University Hospital, Aarhus, Denmark; ^7^Central Finland Central Hospital, Jyväskylä, Finland; ^8^University of Helsinki and Helsinki University Hospital, Department of Emergency Medicine and Services, Helsinki, Finland

**Introduction:** Cerebral hypoperfusion may aggravate the developing neurological damage after cardiac arrest. Near-infrared spectroscopy (NIRS) provides information on cerebral oxygenation but its clinical relevance during post-resuscitation care is undefined. We wanted to assess the possible association between cerebral oxygenation and clinical outcome after out-of-hospital cardiac arrest (OHCA).

**Methods:** We performed a post hoc analysis of a randomised clinical trial (COMACARE) where both moderate hyperoxia and high-normal arterial carbon dioxide tension (PaCO_2_) increased regional cerebral oxygen saturation (rSO_2_) as compared with normoxia and low-normal PaCO_2_, respectively. RSO_2_ was measured from 118 OHCA patients with NIRS during the first 36 h of intensive care and neurological outcome was assessed using the Cerebral Performance Category (CPC) scale at 6 months after cardiac arrest. We calculated the median rSO_2_ for patients with good (CPC 1-2) and poor (CPC 3-5) outcome and compared the results using the Mann-Whitney U test. We compared the rSO_2_ over time with outcome using a generalised mixed model. Finally, we added median rSO_2_ to a binary logistic regression model to control for the effects of possible confounding factors.

**Results:** The median (interquartile range [IQR]) rSO_2_ during the first 36 h of intensive care was 70.0% (63.5-77.0%) in patients with good outcome compared to 71.8% (63.3-74.0%) in patients with poor outcome, p = 0.943. We did not find significant association between rSO_2_ over time and neurological outcome (Figure 1). In the binary logistic regression model rSO_2_ was not a statistically significant predictor of good outcome (OR 0.99, 95% CI 0.94-1.04, p = 0.635).

**Conclusions:** We did not find any association between cerebral oxygenation during the first 36 h of post-resuscitation intensive care and neurological outcome at 6 months after cardiac arrest.

**Fig. 1 (abstract P204).**
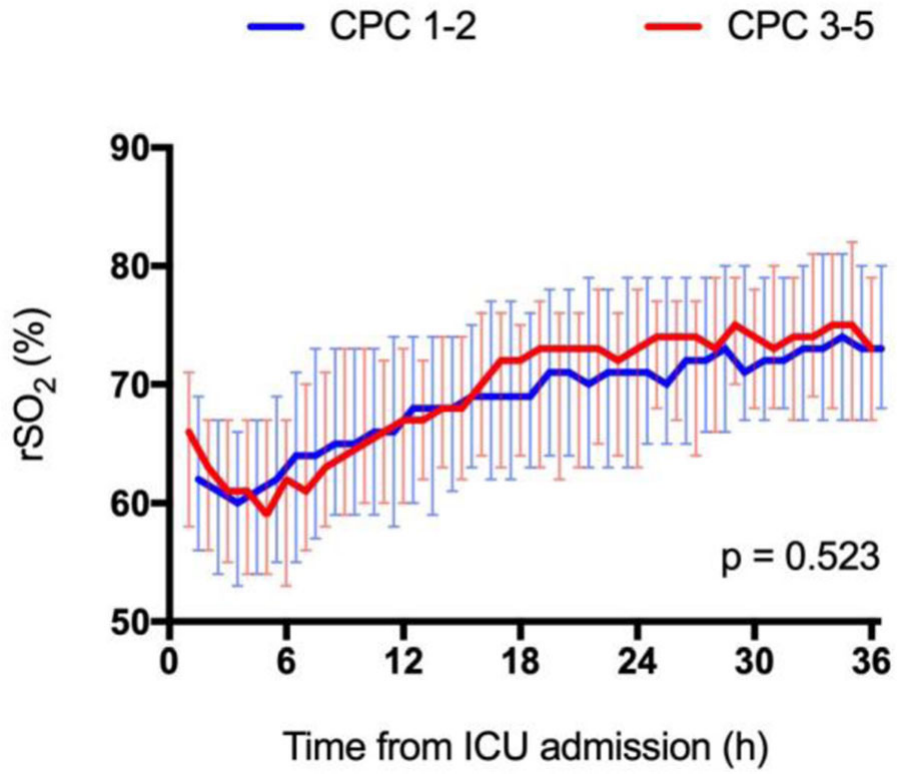
Median (inter-quartile range) regional cerebral oxygen saturation (rSO2) in patients with good (Cerebral Performance Category [CPC] 1-2) and poor (CPC 3-5) neurological outcome during the first 36 h after intensive care unit (ICU) admission

### P205 Cerebral oxygenation after out-of-hospital cardiac arrest: association with neuron-specific enolase at 48 h

#### P Jakkula^1^, M Reinikainen^2^, J Hästbacka^1^, V Pettilä^1^, M Lähde^3^, M Tiainen^4^, E Wilkman^1^, S Bendel^5^, T Birkelund^6^, A Pulkkinen^7^, M Skrifvars^8^

##### ^1^University of Helsinki and Helsinki University Hospital, Perioperative, Intensive Care and Pain Medicine, Helsinki, Finland; ^2^North Karelia Central Hospital, Joensuu, Finland; ^3^Päijät-Häme Central Hospital, Lahti, Finland; ^4^University of Helsinki and Helsinki University Hospital, Department of Neurology, Helsinki, Finland; ^5^Kuopio University Hospital, Kuopio, Finland; ^6^Aarhus University Hospital, Aarhus, Denmark; ^7^Central Finland Central Hospital, Jyväskylä, Finland; ^8^University of Helsinki and Helsinki University Hospital, Department of Emergency Medicine and Services, Helsinki, Finland

**Introduction:** Near-infrared spectroscopy (NIRS) provides a non-invasive means to assess cerebral oxygenation during post-resuscitation care but its clinical value is unclear. We determined the possible association between cerebral oxygenation and the magnitude of brain injury assessed with neuron-specific enolase (NSE) serum concentration at 48 h after out-of-hospital cardiac arrest (OHCA).

**Methods:** We performed a post hoc analysis of a randomised clinical trial (COMACARE) comparing two different levels of carbon dioxide, oxygen and arterial pressure after OHCA and successful resuscitation. We measured rSO_2_ continuously with NIRS from 118 patients during the first 36 h of intensive care. We determined the NSE concentrations at 48 h after cardiac arrest from serum samples using an electrochemiluminescent immunoassay kit. The samples were tested for haemolysis and all samples with a haemolysis index > 500 mg of free haemoglobin per litre (n = 2) were excluded from the analyses. We calculated the median rSO_2_ for all patients and used a scatterplot and Spearman’s rank-order correlation to assess the possible relationship between median rSO_2_ and NSE at 48 h. In addition, we compared the NSE concentrations at 48 h after cardiac arrest in patients with good (Cerebral Performance Category Scale [CPC] 1-2) and poor (CPC 3-5) neurological outcome at 6 months using the Mann-Whitney U test.

**Results:** We did not find significant correlation between median rSO_2_ and serum NSE concentration at 48 h after cardiac arrest, rs = -0.08, p = 0.392 (Figure 1). The median (IQR) NSE concentration at 48 h was 17.5 (13.4-25.0) μg/l and 35.2 (22.6-95.8) μg/l in patients with good and poor outcome, respectively, p < 0.001.

**Conclusions:** We did not find any association between cerebral oxygenation during the first 36 h of post-resuscitation intensive care and NSE serum concentrations at 48 h after cardiac arrest.

**Fig. 1 (abstract P205).**
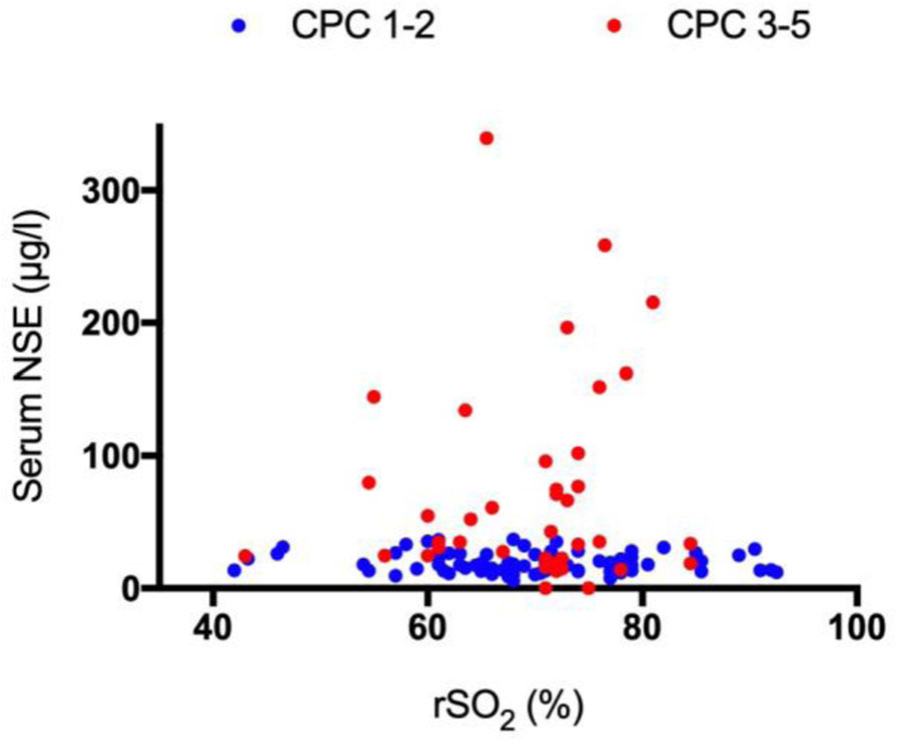
Scatter plots of serum neuron-specific enolase (NSE) concentration at 48 h after cardiac arrest vs. median regional cerebral oxygen saturation (rSO2) during the first 36 h in intensive care unit in patients with good (Cerebral Performance Category [CPC] 1-2) and poor (CPC 3-5) neurological outcome

### P206 The association between lactate, cerebral oxygenation and brain damage in post-cardiac arrest patients

#### P Nuyens^1^, S Janssens^1^, J Dens^2^, C De Deyne^2^, B Ferdinande^2^, M Dupont^2^, PJ Palmers^2^, K Ameloot^2^

##### ^1^University Hospitals Leuven, Cardiology, Leuven, Belgium; ^2^Ziekenhuis Oost-Limburg, Cardiology, Genk, Belgium

**Introduction:** patients admitted to the intensive care unit (ICU) after being successfully resuscitated from a cardiac arrest (CA) have a large cerebral penumbra at risk for secondary ischemic damage in case of suboptimal brain oxygenation. Therefore, resuscitation during ICU stay should be guided by parameters that adequately predict cerebral hypoxia. The value of lactate as resuscitation parameter may be questioned in post-CA patients since the brain critically depends on aerobic metabolism. We aimed to investigate the relationship between arterial lactate, cerebral cortex tissue oxygenation (SctO2) by near infrared spectroscopy (Foresight) and unfavorable neurological outcome at 180days (CPC score 3-5)

**Methods:** Subanalysis from the Neuroprotect post-CA trial. Lactate values and SctO2 were recorded hourly in 102 post-CA patients during 24hours TTM33 and subsequent rewarming.

**Results:** In total 3290 paired lactate/ SctO2 measurements were analysed. We found no correlation between paired lactate and SctO² measurements (R²= 0.01) (Fig.1). Moreover, temporary trends in lactate did not correlate with corresponding trends in SctO2 during the same one-hour time interval (R²=0.003) (Fig 2). If lactate values above 2.0 mmol/l are considered to be abnormal, lactate could not adequately detect clinical important brain ischemia (SctO2 < 60%): sensitivity 62% and specificity 53% (Table 1, 2). Nevertheless, time weighted lactate at 6h (OR 1.38; p 0.01), 12h (OR 1.38, p 0.01), 24h (OR 1.53; p 0.003) and 36h (OR 1.60; p 0.005) were inversely correlated with unfavorable neurological outcome at 180days (Fig 1, 2).

**Conclusions:** Although lactate was a marker of prognosis in post-CA patients, it should not be used to guide resuscitation since lactate values were not correlated with SctO2 and changes in lactate do not correspond with changes in SctO2 during the same time interval.

**Table 1 (abstract P206).** Logistic regression analysis of average lactate as predictor of poor neurological outcome (CPC 3–5) at 6 months

Average lactate	OR	95% CI	p value
Δ h	1.38	1.07-1.77	0.013
Δ h	1.38	1.07-1.77	0.013
Δ h	1.53	1.16-2.03	0.003
Δ h	1.60	1.16-2.22	0.005

**Table 2 (abstract P206).** Sensitivity and specificity of lactate values > 2 mmol/L to detect SctO² < 60%

	Estimated values	95% CI
Sensitivity	61.82%	55.79% - 67.59%
Specificity	52.57%	50.77% - 54.37%
PPV	10.63%	9.71% - 11.61%
NPV	93.79%	92.83% - 94.63%
LR+	1.3	1.18 - 1.44
LR-	0.73	0.62 - 0.85

**Fig. 1 (abstract P206).**
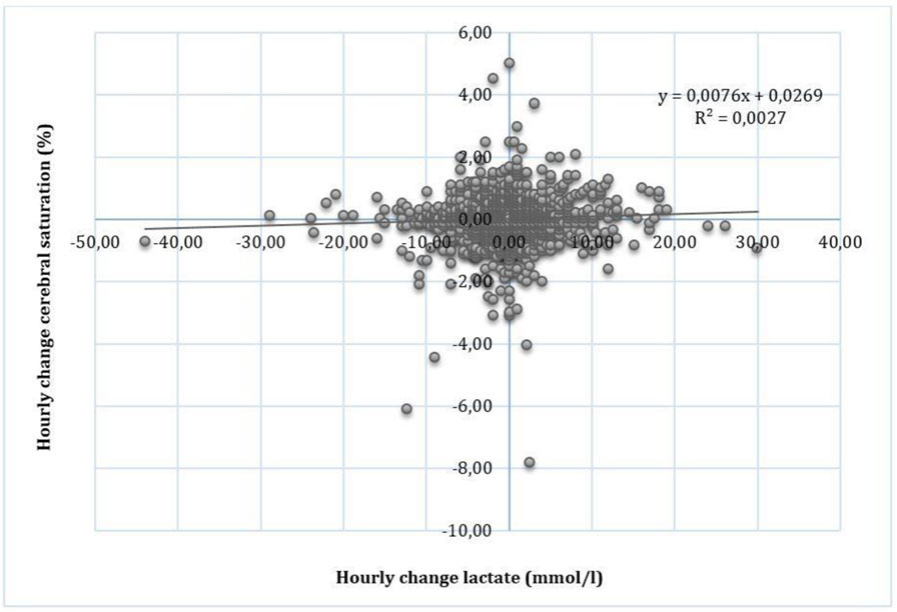
Linear correlation between paired lactate and SctO² measurements

**Fig. 2 (abstract P206).**
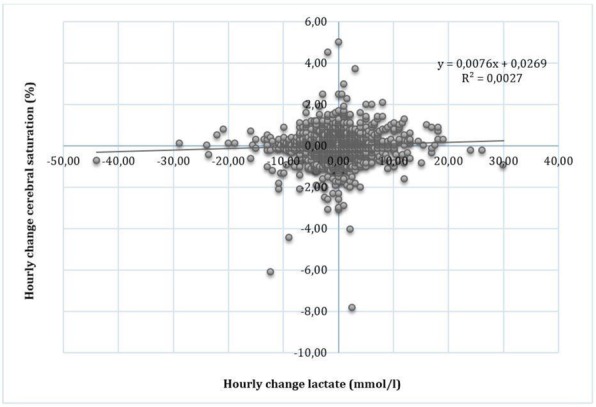
Linear correlation between paired, hourly lactate and SctO² measurements

### P207 Simplified monitoring and interpretation of continuous EEG after cardiac arrest - a useful bedside tool for the ICU physician

#### A Lybeck^1^, T Cronberg^2^, O Borgquist^1^, J During^3^, G Mattiasson^1^, D Piros^3^, S Backman^4^, H Friberg^3^, E Westhall^4^

##### ^1^Skane Univeristy Hospital, Dept of Anaesthetics and Intensive Care, Lund, Sweden; ^2^Skane Univeristy Hospital, Dept of Neurology, Lund, Sweden; ^3^Skane University Hospital, Dept of Anaesthetics and Intensive Care, Malmo, Sweden; ^4^Skane Univeristy Hospital, Dept of Clinical Neurophysiology, Lund, Sweden

**Introduction:** The aim of the study was to investigate whether simplified continuous EEG monitoring (cEEG) [1] post-cardiac arrest can be reliably interpreted by ICU physicians after a short structured training, and whether acceptable interrater agreement compared to an EEG-expert can be achieved.

**Methods:** Five ICU physicians received training in interpretation of simplified cEEG (Fig 1) consisting of lectures, hands-on cEEG-interpretation, and a video tutorial - total training duration 1 day. The ICU physicians then interpreted 71 simplified cEEG recordings. Basic EEG background patterns and presence of epileptiform discharges or seizure activity were assessed on 5-grade rank-ordered scales based on a standardized EEG terminology [2]. An experienced EEG-expert was used as reference.

**Results:** There was substantial agreement (κ 0.69) for EEG background patterns and moderate agreement (κ 0.43) for epileptiform discharges between ICU physicians and the EEG-expert. Sensitivity for detecting seizure activity by the ICU physicians was limited (50%), but with high specificity (87%). Among ICU physicians interrater agreement was substantial (κ 0.63) for EEG background pattern and moderate (κ 0.54) for epileptiform discharges.

**Conclusions:** After a one-day educational effort clinically relevant agreement was achieved for basic EEG background patterns after cardiac arrest. Assessment of epileptiform patterns was less reliable, but bedside screening by the ICU physician may still be clinically useful for early detection of seizures. Interpretation of simplified cEEG requires awareness of its limitations and support from an EEG-expert when clinically indicated.

**References**

1 Friberg H, et al. Crit Care 17:233-242, 2013

2 Hirsch LJ, et al. J Clin Neurophysiol 30:1-27, 2013

**Fig. 1 (abstract P207).**
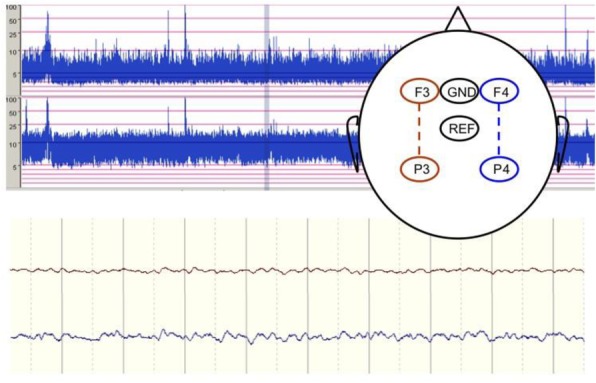
1 cEEG montage and example of simplified continuous EEG recording: F3, P3, F4, P4, reference Cz, ground Fz. The upper curves display the left and the right time-compressed aEEG, the y-axis displays the semilogarithmic uV scale. The shaded aEEG marks the time span of the aEEG corresponding to the original EEG below. The lower part displays the original EEG recording from the left and the right hemispheres; each division represents 1 sec on the x-axis

### P208 Neurocardiac risk stratification 6 hours after resuscitation from cardiac arrest

#### A Bilkanovic^1^, Z Haxhija^1^, L Lucas^2^, J Dziodzio^1^, R R. Riker^1^, T May^1^, H Friberg^3^, D B. Seder^1^

##### ^1^Maine Medical Center, Department of Critical Care Services and Neuroscience Institute, Portland, United States; ^2^Maine Medical Center, Center for Outcomes Research and Evaluation, Portland, United States; ^3^Skåne University Hospital, Department of Anaesthesia and Intensive Care, Lund, Sweden

**Introduction:** A tool to distinguish between the competing risks of neurologic- and circulatory-etiology death very early after return of spontaneuos circulation (ROSC) would facilitate triage decisions to extracorporeal membrane oxygenation, cardiac revascularization, and experimental neuroprotective therapies. We combined the electroencephalographic suppression ratio (SR) and CREST score (Coronary artery disease, initial non-shockable Rhythm, low Ejection fraction, admission Shock and Time to ROSC>25 minutes), a validated model to predict risk of circulatory-etiology death (CED), to create new neurocardiac risk classes.

**Methods:** Prospectively collected registry data included demographics, clinical data, the SR recorded at 6 hours after ROSC (SR6), CREST score, and outcomes. Utility of SR6 to predict neurological-etiology death (NED) was evaluated with a ROC curve. Clinically appropriate cutoffs to describe risk levels for each tool were selected, and patients stratified into 6 groups to demonstrate competing risks of CED and NED.

**Results:** Of 543 patients, 227 (42%) survived to hospital discharge, 239 (44%) had NED, 77 (14%) had CED. SR6 values predicted NED with AUC 0.89, and clinical thresholds were set at SR6 <37% (low risk of NED; 3-11%), SR6 37-82% (intermediate risk; 23-60%) and SR >83% (high risk of NED; 71-89%). CED occurred in 11-13% in low CREST (0-2) groups and 18-28% in the high CREST (3-5) groups. No patient with both high neurological and circulatory risk survived, and the lowest risk group had 87% survival. Relative risk of NED:CED in patients with lowest SR6 was low (1:4.3); in patients with highest SR6, NED:CED was high (5.2:1).

**Conclusions:** A neurocardiac risk model using early SR and CREST score allows for risk stratification at 6 hours after ROSC, and could be used to optimize the effectiveness of post-resuscitation care pathways and interventions.

### P209 Interobserver variability in the interpretation of early head computed tomography following out of hospital cardiac arrest

#### A Caraganis^1^, RR Kempainen^2^, M Mulder^3^, R Brown^4^, M Oswood^2^, ME Prekker^2^

##### ^1^University of Minnesota, Division of Pulmonary, Allergy and Critical Care, Minneapolis, United States; ^2^Hennepin Healthcare, Minneapolis, United States; ^3^Abbott Northwestern Hospital, Minneapolis, United States; ^4^University of Minnesota, Minneapolis, United States

**Introduction:** Hypoxic-ischemic injury on head computed tomography (CT), which manifests with varying degrees of cerebral edema and loss of gray-white matter differentiation, is a poor prognostic sign after resuscitated out-of-hospital cardiac arrest that may influence early clinical decision-making. Agreement among physicians on the presence of hypoxic-ischemic injury on early head CT is unknown.

**Methods:** We recruited 10 faculty physician participants (2 emergency medicine, 3 critical care, 3 neurocritical care, and 2 general radiology; average 8.2 years of practice) across 3 academic medical centers each with >100 admissions for resuscitated out-of-hospital cardiac arrest each year. Participants, blinded to clinical context, reviewed 20 unique head CTs obtained within 2 hours of cardiac arrest that were randomly selected from a local registry. A blinded neuroradiologist also reviewed all scans (gold standard). Participants determined if hypoxic-ischemic injury was present on each CT, and agreement was determined using multi- and dual-rater kappa statistics with 95% confidence intervals.

**Results:** Overall agreement among physicians regarding the presence of hypoxic-ischemic injury on head CT was fair (kappa 0.34; 95% CI, 0.19-0.49) with agreement consistent across most specialties (Table 1). When compared to the neuroradiologist, individual physician agreement ranged widely, from poor (kappa 0.12) to substantial (kappa 0.68), with 6 of 10 physicians having fair or worse agreement compared to the gold standard interpretation.

**Conclusions:** The finding of hypoxic-ischemic injury on early head CT after cardiac arrest had high interobserver variability as interpreted by acute care physicians and general radiologists. Pending the development of objective diagnostic criteria, clinicians should bear in mind the subjectivity and subtlety of cerebral edema or loss of gray-white matter differentiation soon after return of spontaneous circulation in these patients.

**Table 1 (abstract P209).** Interobserver agreement on the presence of hypoxic-ischemic injury on early head computed tomography after cardiac arrest

Medical Specialty	Number of Physicians	Multi-Rater Kappa Statistic (95% CI)
Emergency Medicine	2	0.61 (0.30-0.90)
Critical Care	3	0.28 (-0.01-0.54)
Neurocritical Care	3	0.29 (0.0-0.55)
General Radiology	2	0.39 (-0.03-0.80)
All Physicians	10	0.34 (0.19-0.49)

### P210 Testing before stopping: a 6-year audit of neuro-prognostication tests after cardiac arrest

#### P Eiben, S Blakey, J Van Griethuysen, D Noakes, F Caetano, E Poimenidi, A Gupta, A Pineau Mitchell, G Patel, R Maharaj, S Vlachos

##### King´s College Hospital NHS Foundation Trust, Critical Care, London, United Kingdom

**Introduction:** We assessed institutional adherence of neuro-prognostication test use before Withdrawal of Life Sustaining Treatment (WLST) to international guidance. We also assessed the change in practice over the six-year time frame and compared the time periods before and after publication of the 2015 ERC-ESICM guidelines [1].

**Methods:** Retrospective analysis of adult comatose Out of Hospital Cardiac Arrest (OHCA) survivors, admitted to a tertiary referral Intensive Care Unit (ICU) between January 2012 and December 2017. Blinded data collection from electronic and paper records followed the Utstein template. Patients who were not intubated, had a non-cardiac arrest or stayed in ICU ²72h were excluded since their neuro-prognostication differed. We used Fisher´s exact tests for categorical variables, Wilcoxon tests for continuous variables and non-parametric tests for trend.

**Results:** We included 287 patients (Figure 1). Baseline characteristics and differences between the WLST and no-WLST groups are shown in Table 1. Utilization of neuro-prognostication tests is shown in Table 2. While CT and EEG were commonly employed, SSEP and MRI were used less frequently. Basic multimodal neuro-prognostication (arbitrarily defined as at least one CT or MRI, plus EEG, plus SSEP) was performed only in 34.1% of all patients undergoing WLST but the rate increased significantly over six years (p<0.001) and was higher in the time period after 2015, compared to the one prior to 2015 (Figure 2). This association remained significant after adjustment for confounders such as age, arrest rhythm, downtime, targeted temperature management, APACHE II score and organ failure in a logistic regression model (p=0.004).

**Conclusions:** In an institution with access to a wide range of imaging and neurophysiology tests, MRI and SSEP remained under-utilized but the rate of basic multimodal neuro-prognostication increased significantly over the study period, especially in the period after 2015.

**Reference**

1. Nolan JP, et al. Resuscitation. 2015;95:202-22.

**Table 1 (abstract P210).** Baseline characteristics of patients by category of withdrawal of life support (WLST)

Variable [n (%) or median (IQR)]	WLST (N=85)	No WLS (N=202)	p-value
Age	67 (56.5-75)	60 (49-69)	0.002
Female sex	22 (26.9)	44 (21.8)	0.447
Initial rhythm VF or VT	49 (63.6)	148 (81.8)	0.002
Downtime (minutes)	25.3 (19.5-40)	18.5 (10-27)	0.002
Targeted Temperature Management	39 (58.2)	94 (61.4)	0.656
APACHE II score	26 (21-30)	20 (16-26)	<0.001
Any organ support	72 (84.7)	168 (83.2)	0.862

**Table 2 (abstract P210).** Neuro-prognostication tests performed in patients after OHCA

Variable (n, %)	WLST (N=85)	No WLS (N=202)	p-value
Computed Tomography (CT)	74 (87.1)	150 (74.3)	0.019
Magnetic Resonance Imaging (MRI)	16 (18.8)	29 (14.4)	0.375
Electroencephalography (EEG)	68 (80.0)	74 (36.6)	<0.001
Somatosensory Evoked Potentials (SSEP)	29 (34.1)	28 (13.9)	<0.001
Basic multimodal neuro-prognostication (n, %)	29 (34.1)	28 (13.9)	<0.001

**Fig. 1 (abstract P210).**
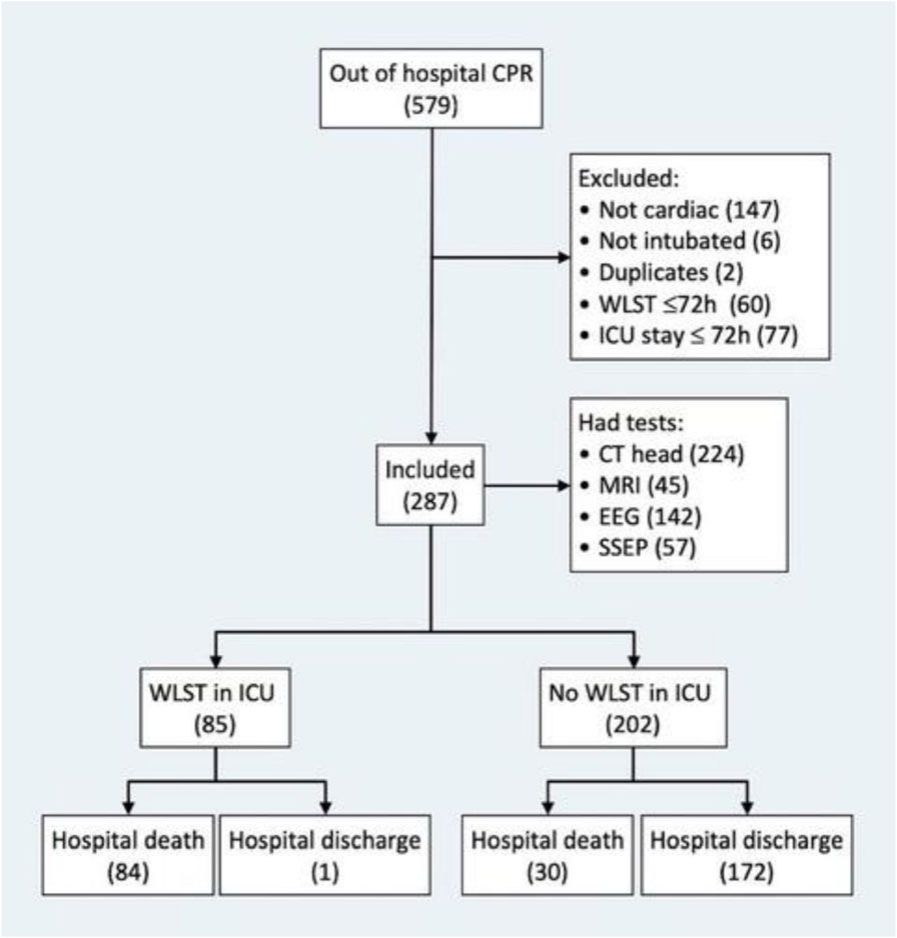
Patient flow during the study period

**Fig. 2 (abstract P210).**
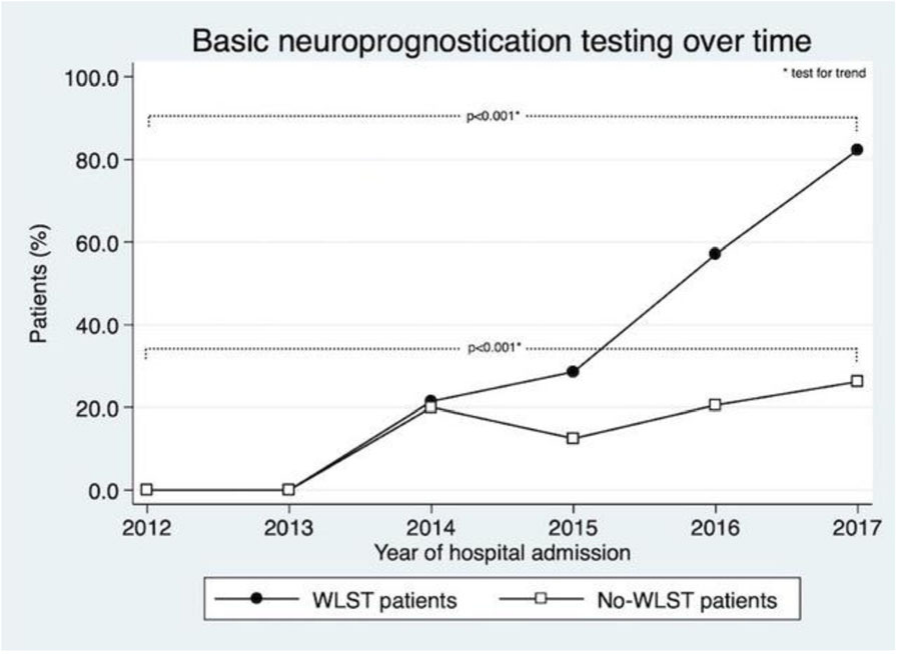
Proportion of patients receiving basic multimodal neuro-prognostication over the study period

### P211 Melatonin protects autophagy-like cell death cerebellar Purkinje cells following asphyxial cardiac arrest through attenuation of oxidative stress via MT2 receptor

#### JK Seo^1^, HJ Lim^1^, JB Moon^1^, TG Ohk^1^, MC Shin^1^, KE Kim^1^, MH Won^2^, JH Cho^3^

##### ^1^Kangwon National University, Emergency Medicine, Chuncheonsi, South Korea; ^2^Kangwon National University, Neurobiology, Chuncheonsi, South Korea; ^3^Kangwon National University, Department of Emergency Medicine, Chuncheonsi, South Korea

**Introduction:** Although multiple reports using animal models have confirmed that melatonin appears to promote neuroprotective effects following ischemia/reperfusion-induced brain injury, the relationship between its protective effects and the activation of autophagy in cerebellar Purkinje cells following the asphyxial cardiac arrest and cardiopulmonary resuscitation (CA/CPR) remains unclear.

**Methods:** Rats used in this study were randomly assigned to 6 groups as follows; vehicle-treated sham-operated group, vehicle-treated asphyxial CA/CPR-operated group, melatonin-treated sham-operated group, melatonin-treated asphyxial CA/CPR-operated group, melatonin plus (+) 4P-PDOT (the MT2 melatonin receptor antagonist)-treated sham-operated group and melatonin+4P-PDOT-treated asphyxial CA/CPR-operated group.

**Results:** Our results demonstrate that melatonin (20 mg/kg, IP, 1 time before CA and 4 times after CA) significantly improved the survival rates and neurological deficits compared with the vehicle-treated asphyxial CA/CPR rats (survival rates ≥ 40% vs 10%). We also demonstrate that melatonin exhibited the protective effect against asphyxial CA/CPR-induced Purkinje cell death. The protective effect of melatonin in the Purkinje cell death following asphyxial CA/CPR paralleled a dramatic reduction in superoxide anion radical (O2·-), intense enhancements of CuZn superoxide dismutase (SOD1) and MnSOD (SOD2) expressions, as well as a remarkable attenuation of autophagic activation (LC3 and Beclin-1), which is MT2 melatonin receptor-associated. Furthermore, the protective effect of melatonin was notably reversed by treatment with 4P-PDOT.

**Conclusions:** This study shows that melatonin conferred neuroprotection against asphyxial CA/CPR-induced cerebellar Purkinje cell death by inhibiting autophagic activation by reducing expressions of ROS, while increasing of antioxidative enzymes, and suggests that MT2 is involved in the neuroprotective effect of melatonin in cerebellar Purkinje cell death induced by asphyxial CA/CPR.

### P212 Pretreated fucoidan confers neuroprotection against transient global cerebral ischemic injury in the gerbil hippocampal CA1 area via reducing of glial cell activation and oxidative stress

#### HJ Lim^1^, JK Seo^1^, JB Moon^1^, TG Ohk^1^, MC Shin^1^, KE Kim^1^, MH Won^2^, JH Cho^3^

##### ^1^Kangwon National University, Emergency Medicine, Chuncheonsi, South Korea; ^2^Kangwon National University, Neurobiology, Chuncheonsi, South Korea; ^3^Kangwon National University, Department of Emergency Medicine, Chuncheonsi, South Korea

**Introduction:** Fucoidan is a sulfated polysaccharide derived from brown algae and possesses various beneficial activities, such as anti-inflammatory and antioxidant properties. Previous studies have shown that fucoidan displays protective effect against ischemia-reperfusion injury in some organs. However, few studies have been reported regarding the protective effect of fucoidan against cerebral ischemic injury and its related mechanisms.

**Methods:** Therefore, in this study, we examined the neuroprotective effect of fucoidan against cerebral ischemic injury, as well as underlying mechanisms using a gerbil model of transient global cerebral ischemia (tGCI) which shows loss of pyramidal neurons in the hippocampal cornu ammonis 1 (CA1) area. Fucoidan (25 and 50 mg/kg) was intraperitoneally administered once daily for 3 days before tGCI.

**Results:** Pretreatment with 50 mg/kg of fucoidan, not 25 mg/kg fucoidan, attenuated tGCI-induced hyperactivity and protected CA1 pyramidal neurons from ischemic injury following tGCI. In addition, pretreatment with 50 mg/kg of fucoidan inhibited activations of resident astrocytes and microglia in the ischemic CA1 area. Furthermore, pretreatment with 50 mg/kg of fucoidan significantly reduced the increased 4-hydroxy-2-noneal and superoxide anion radical production in the ischemic CA1 area after tGCI and significantly increased expressions of superoxide dismutase 1 (SOD1) and SOD2 in the CA1 pyramidal neurons compared with the vehicle-treated-group. We found that treatment with diethyldithiocarbamate (an inhibitor of SODs) to the fucoidan-treated-group notably abolished the fucoidan-mediated neuroprotection in the ischemic CA1 area following tGCI.

**Conclusions:** These results indicate that fucoidan can effectively protect neurons from tGCI-induced ischemic injury through attenuation of activated resident glial cells and reduction of oxidative stress following increasing SODs. Thus, we strongly suggest that fucoidan can be used as a useful preventive agent in cerebral ischemia.

### P213 The effects of cold fluids for induction of therapeutic hypothermia on reaching target temperature and complications– a sub-study of the TTH48 study

#### A Holm^1^, M Skrifvars^2^, FS Taccone^3^, E Søreide^4^, A Grejs^5^, C Duez^5^, A Jeppesen^5^, H Kirkegaard^5^

##### ^1^University of Helsinki, Faculty of Medicine, Helsinki, Finland; ^2^Dept. of Emergency Care and Services, Helsinki University Hospital, Finland, Helsinki, Finland; ^3^Dept. of Intensive Care, Erasme Hospital, Belgium, Brussels, Belgium; ^4^Dept. of Anesthesiology and Intensive Care, Stavanger University Hospital, Stavanger, Norway; ^5^Dept. of Anesthesiology and Intensive Care Medicine, Aarhus University Hospital, Aarhus, Denmark

**Introduction:** Induction of hypothermia with cold fluids does not improve outcome in out- of- hospital cardiac arrest (OHCA) [1]. We hypothesized that this may be due to ineffective cooling and side effects.

**Methods:** A post hoc analysis of the randomised TTH48 trial (NCT01689077) comparing cooling for 48 or 24 hours after OHCA. Data collection included cardiac arrest factors, adverse effects, cooling methods and continuous core temperature measurements. The primary outcome endpoint was time to target temperature (TTT, <34°C) and prevalence of abnormal electrolyte levels and oxygen saturation within the first 24 hours. TTT was considered significantly reduced if it was at least 1 hour shorter with i.v. fluid cooling than with no or only surface pre-ICU cooling. We compared electrolyte and circulatory side effects between groups.

**Results:** Intervention group given pre-hospital cold fluids contained 110 patients and the control group 242 patients. The pre-ICU cold fluid cooling increased TTT (347min vs. 268min, p=0.010). Temperatures over time varied based on whether pre-ICU i.v. fluids were given (Figure 1). With linear multiple regression in a model including age, basic CPR, shockable or non-shockable rhythm, whether the CA was witnessed or not, time to ROSC, the use of cold fluids was positively associated with longer time to target temperature (coefficient 1.22, 95% CI 4.9 and 71.7, p=0.025). There was no difference in paO2 on ICU admission (mean 15.5 kPa compared to 16 kPa, p=0.787). Cold fluid use did not increase electrolyte abnormalities (Na+<130mmol 1.8% vs. 2.9% p=0.540, K+<3.0mmol 1.8% vs. 4.5% p=0.202).

**Conclusions:** Contrary to our hypothesis, the initiation of targeted temperature management with cold fluids before ICU arrival may increase TTT but does not cause other side effects.

**Reference**

1. Francis K et al. JAMA 311:45-52, 2014

**Fig. 1 (abstract P213).**
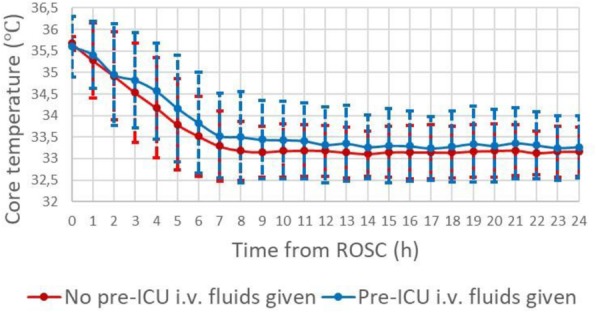
Temperature of patients given and not given pre-ICU fluids

### P214 Targeted temperature management during extracorporeal life support after CPR

#### A Mecklenburg^1^, J Stamm^2^, F Angriman^3^, G Söffker^2^, H Reichenspurner^3^, L Del Sorbo^1^, E Fan^1^, S Kluge^2^, S Braune^4^

##### ^1^Interdepartmental Division of Critical Care, University of Toronto, University of Toronto, Toronto, Canada; ^2^Department of Critical Care, University Medical Center Hamburg-Eppendorf, Hamburg, Germany; ^3^Department of Cardiac Surgery, University Heart Center Hamburg, Hamburg, Germany; ^4^Department of Medical Intensive Care and Emergency Medicine, St. Franziskus-Hospital, Münster, Germany

**Introduction:** Extracorporeal life support (ECLS) is increasingly used after cardiopulmonary resuscitation. However, bleeding complications are common and challenging. Targeted temperature management (TTM) improves neurologic outcome after CPR but may compromise coagulation. The purpose of this study was to examine whether TTM in patients on ECLS post CPR increases risk for major bleeding.

**Methods:** Retrospective single-center study of patients on ECLS (vaECMO) post CPR±TTM from Jan 2009 to Dec 2015, using inverse probability weighting of a marginal structural modified Poisson regression model to estimate TTM effect. Bleeding was recorded within 36hrs post CPR (early observation) and between 36 to 72hrs post CPR (late observation) using the BARC score [1]. Secondary outcomes: ICU survival, 28d-mortality, organ dysfunction.

**Results:** 36 out of 78 patients received TTM in addition to ECLS after CPR. They were younger (48 [38.5-57] vs. 55.5 [48.3-65.5] yrs), had higher illness severity (SAPS2 66.5 [57.8-74.3] vs. 56 [43.3-66.3]), and, higher incidence of lung injury (83.3% vs. 23.8%), brain injury (58.3% vs. 28.6%) and multi-organ failure (88.9% vs. 64,3%). ICU survival and 28-d mortality did not differ. Overall bleeding frequency (BARC 1-5) was similar in both groups (TTM 86.1% vs. noTTM 92.9%). There was no difference in early bleeding incidences (Fig 1). During late observation, TTM patients had fewer minor bleeding (55.2% vs. 100%) and more intracranial bleeding (23.1% vs. 0%; Fig 2). Adjusted calculated risk ratio for major bleeding (including intracranial) for TTM was 0.24 (95%CI 0.10-0.54) at baseline and 1.45 (95%CI 1.18-1.77) over time.

**Conclusions:** Bleeding complications were common. Although the risk ratio for major bleeding increased over time in TTM patients, residual and unmeasured confounding in addition to selection and detection bias may limit the clinical relevance of this finding.

**Reference**

1) Mehran Ret al., Circulation, 123:2736–2747, 2011

**Fig. 1 (abstract P214).**
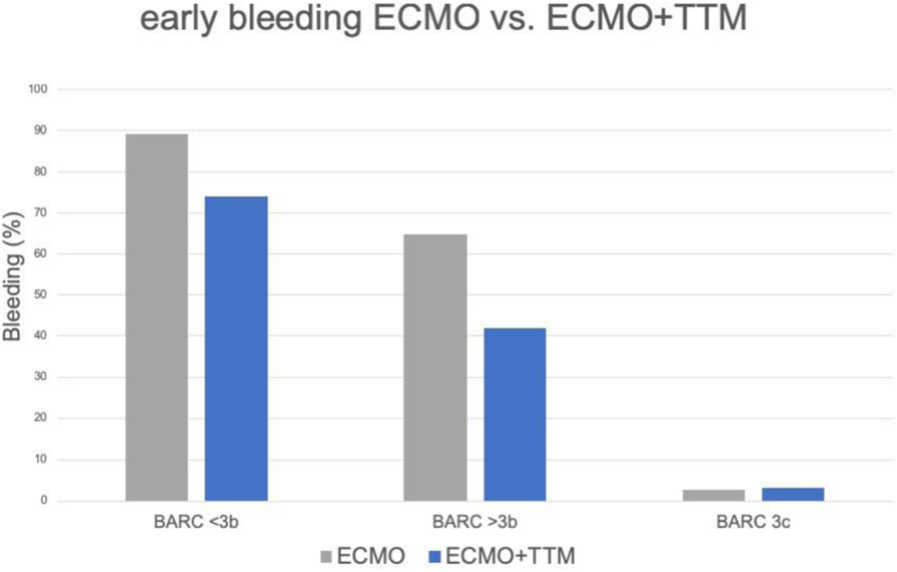
Bleeding frequencies in early observation period, ECMO vs. ECMO+TTM

**Fig. 2 (abstract P214).**
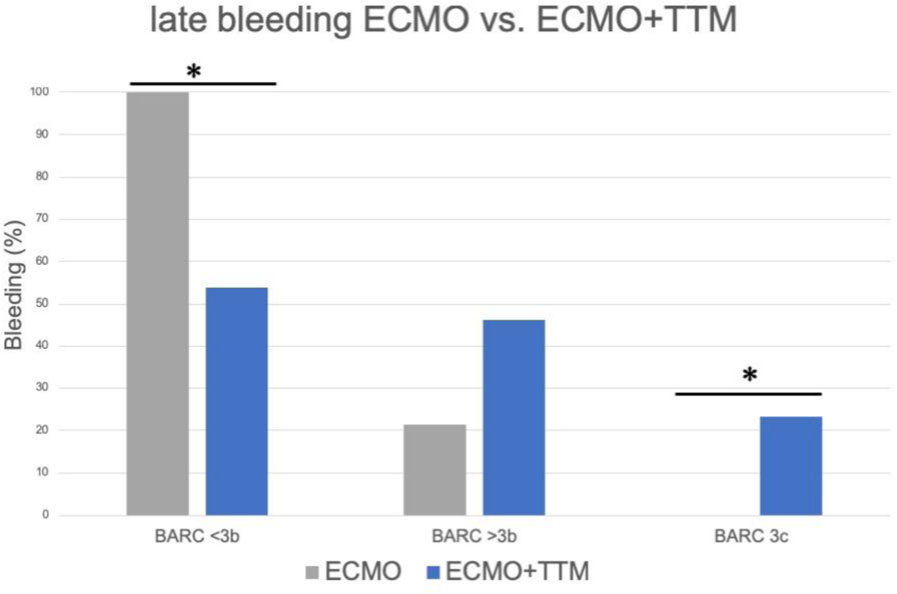
Bleeding frequencies in late observation period, ECMO vs. ECMO+TTM, p=0.05

### P215 Features of cerebral thermal balance in the chronically critically ill patients

#### M Petrova^1^, O Shevelev^2^, S Shavkat^1^, R Mohan^2^

##### ^1^Federal scientific and clinical center of resuscitation and rehabilitation, Moscow, Russia; ^2^Peoples´ Friendship University of Russia, Moscow, Russia

**Introduction:** In acute period of different cerebral events, often, there is fever & focal cerebral hyperthermia which can worsen the condition. Supposedly, the change in brain thermal balance (TB) can also develop in critical conditions (coma, vegetative & minimally conscious state) but there is inadequate information about variations in cerebral temperature (t°) thus, research was done to study the cerebral TB in the chronically critically ill (CCI) in comparison with healthy people (HP) & patients in acute period of ischemic stroke (IS).

**Methods:** Patients with neurological deficit > 10 by NHISS were included. The t° of the brain was recorded non-invasively using radiothermometer RTM-01-RES (Russia). We measured t° in 18 symmetric regions of left & right hemispheres, calculated the average t° of brain, Δ°C between warmest & coolest brain regions, made the correlative analysis of t° variation in symmetric regions (Pearson correlation) in HP (n=120), IS (n=65) & CCI (n=65).

**Results:** In HP, disposition of cold & warm regions doesn’t have typical localization, it was different in every individual. In IS, the region of focal hyperthermia & the location of penumbra coincided in 85% of cases (Table 1).

**Conclusions:** Observed moderate brain t° heterogenecity in HP, marked increase brain t° heterogenecity in IS & sharp decline of t° heterogenecity in CCI. Supposedly, correcting the impairment of cerebral TB (increase or decrease t°) through physical (selective cerebral hypothermia, magnetic stimulation etc.) or pharmacological (sedation) can contribute to positive therapeutic results in IS & CCI. Nonivasive radiothermometry of the brain can be an objective method of patients’ condition evaluation & their rehabilitation potential.

**Table 1 (abstract P215).** Results

Patients	Average cerebral t	Δ °	Correlation coefficient
HP	36.64±0.37°C	1-2°C	0.494±0.09 - 0.747±0.07 *
IS	37.24±0.37°C	2.5-4°C	0.370±0.09 - 0.848±0.05 *
CCI	36.98±0.18°C	<1.5°C	0.914±0.05 - 0.974±0.04**

* strong reliable relationship, medium strength connection

** reliable relationship, strong connection

### P216 Angio-oedema or pseudobulbar palsy? The masquerades of basilar artery stroke

#### J Clarke, V Della Torre

##### West Suffolk Hospital NHS Foundation Trust, Department of Anaesthesia and Intensive Care, Bury St Edmunds, United Kingdom

**Introduction:** Basilar artery stroke has a multitude of different presentations and may not be captured on plain Computed Tomography (CT). It can progress to severe disability, locked in syndrome and death [1]. With the advent of thrombolytic and endovascular therapies, prompt diagnosis can change the outcome. We present a case of basilar artery stroke, which was heralded by tongue spasticity and dysarthria, indicative of pseudobulbar palsy.

**Methods:** Case reviewed with consent. A literature search was conducted using PubMed and Medline.

**Results:** A 53-year-old presented with pulmonary oedema and hypertension. He was transferred to our intensive care unit for treatment of a suspected anaphylaxis. His marked lingual swelling was associated with dysarthria. Glyceryl-trinitrate and labetalol infusions were started for hypertension. He developed left sided weakness and deteriorated over several days to the point that he could only move his right foot (Table 1). Magnetic Resonance Imaging (MRI) showed midbrain ischaemia and angiogram showed no flow in the basilar artery (Fig 1,2).

**Conclusions:** Common presenting features of basilar artery occlusion include dysarthria, vertigo, vomiting, headache and motor defects; these may evolve gradually or be intermittent [1,2]. Presentation with pseudobulbar palsy is described in early literature [2]. Delayed recognition of the stroke led to aggressive treatment of hypertension, potentially compromising perfusion to the penumbral area [2,3]. This case highlights the need for a wide index of suspicion with posterior strokes.

**Consent:** Informed consent to publish has been obtained from the patient

**References**

1 Ausman JI et al. Surg Neurol Int. 9:106, 2018

2 Silversides JL. Proc R Soc Med. 47:290-3, 1954

3 Jauch EC et al. Stroke. 44:889-891, 2013

**Table 1 (abstract P216).** Trends in blood pressure, neurological signs and imaging results

Day / time	Blood Pressure	Anti-hypertensives	Neurological Progression	Significant Imaging Results
Day 1 08:00	195/145	Amlodipine	GCS 15	
Day 1 12:00	250/121	GTN and labetalol infusion	GCS 14: E4 V4 M6, dysarthria, lingual swelling	
Day 1 19:00	168/98	GTN and labetalol infusion	GCS 15: E4 V5 M6, left sided hemiparesis	CT: possible distal right MCA thrombus
Day 2 13:00	157/71	GTN and labetalol infusion	GCS 15: E4 V5 M6	
Day 3 23:00	166/81	Labetalol infusion, bisoprolol, ramipril	GCS 12: E2 V2 M5, right upper limb weakness	MRI: basilar artery occlusion, midbrain ischaemia
Day 4 9:00	172 /74	Bisoprolol, ramipril, doxazosin	GCS 8-9: E3 M3-4 V2, unresponsive episodes	CT: basilar artery occlusion, midbrain ischaemia
Day 8 09:00	164/75	Bisoprolol, ramipril, doxazosin	GCS 10: E3 V1 M6, right lower limb weakness	

**Fig. 1 (abstract P216).**
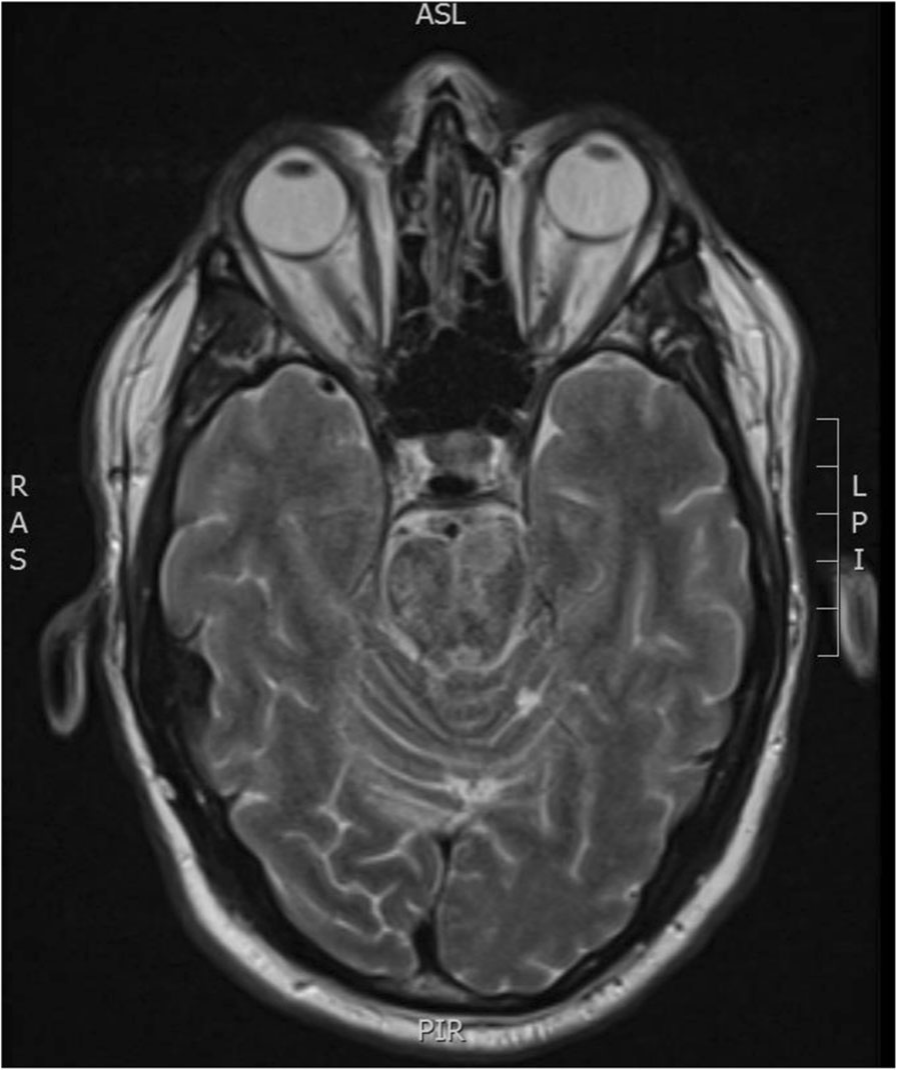
MRI brain showing bilateral midbrain infarcts

**Fig. 2 (abstract P216).**
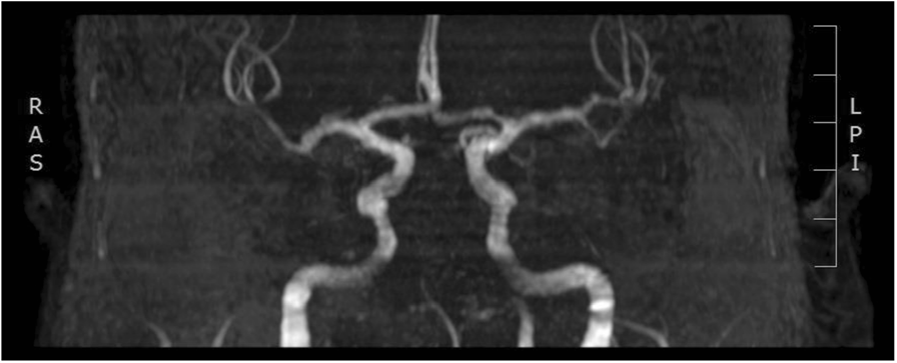
MRI Angiogram showing absence of flow in the basilar artery

### P217 Burns intensive care (ICU) survivors show altered functional connectivity between sub-regions of the default mode network compared to healthy volunteers

#### EJ Watson^1^, S O´Connor^1^, ME Finnegan^2^, M Grech-Sollars^3^, CE Richards^4^, K Nenadlova^5^, L Honeyfield^2^, R Quest^2^, TL Edginton^6^, AD Waldman^7^, MP Vizcaychipi^1^

##### ^1^Chelsea and Westminster Hospital NHS Foundation Trust, Magill Department of Anaesthesia, Intensive Care Medicine and Pain Management, London, United Kingdom; ^2^Imperial College Healthcare NHS Trust, Department of Imaging, London, United Kingdom; ^3^Imperial College London, Department of Surgery & Cancer, London, United Kingdom; ^4^Morriston Hospital, Department of Medicine, Swansea, United Kingdom; ^5^University of Westminster, Department of Psychology, London, United Kingdom; ^6^City University, Department of Psychology, London, United Kingdom; ^7^University of Edinburgh, Centre for Clinical Brain Sciences, Edinburgh, United Kingdom

**Introduction:** Up to two thirds of patients discharged from ICU experience long-term cognitive impairment (LTCI) and this can have a significant impact on quality of life. Little is currently known about its neurobiological basis. One area of interest is the Default Mode Network (DMN), a well-defined resting state functional network that is disrupted in pathologies including Alzheimer’s disease and Traumatic Brain Injury.

**Methods:** 15 patients, who had previously sustained a significant burns injury and had been admitted to burns ICU for invasive ventilation, and 15 volunteers, underwent resting state functional MRI (fMRI) scans. Data analysis was performed using the CONN toolbox in SPM8. Regions of Interest (ROIs) were the four major subdivisions of the DMN (anterior, posterior, l-TPJ and r-TPJ). The average BOLD time series for each ROI was correlated with all other ROIs. The correlation coefficients were compared between patients and volunteers using ANCOVA and correlated with cognitive performance (linear regression). Age and gender adjustment and multiple comparisons correction were undertaken.

**Results:** Reciprocal functional connectivity between r-TPJ and Posterior-DMN (F(1)(26) = 7.62, p=0.03) (Figure 1) and between r-TPJ and l-TPJ (F(1)(26) = 9.07, p=0.03) was lower in patients compared to volunteers. Functional connectivity between r-TPJ and Posterior-DMN positively correlated with cognitive performance across the cohort (r2=0.36, p=0.001), even after adjusting for predicted premorbid IQ levels (r2=0.37, p=0.002).

**Conclusions:** To our knowledge this is the first study demonstrating resting state network disruption in ICU patients. Since functional connectivity correlates with cognitive performance, further focussed research in this area may allow for targeted prevention or treatment of ICU related LTCI.

**Fig. 1 (abstract P217).**
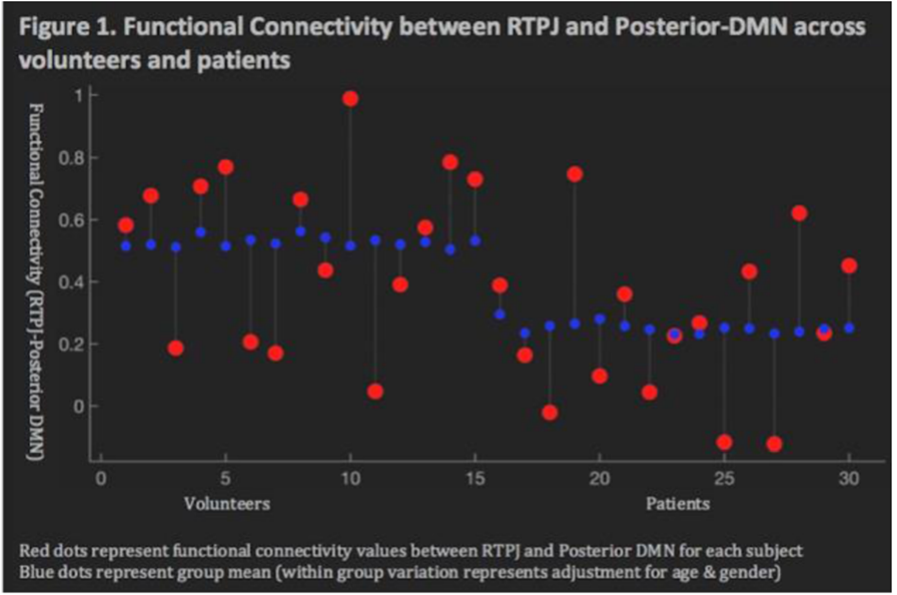
Functional connectivity between RTPJ and posterior-DMN across volunteers and patients

### P218 Ischemic stroke predictors in an Italian ICU

#### A Marudi^1^, G Bettelli^1^, G Branchetti^2^, E Marinangeli^2^, R Sandulli^2^, E Bertellini^1^

##### ^1^Azienda Ospedaliero Universitaria di Modena, Struttura Complessa di Anestesia e Rianimazione, Modena, Italy; ^2^Azienda Ospedaliero Universitaria di Modena, Università degli Studi di Modena e Reggio Emilia, Modena, Italy

**Introduction:** Ischemic stroke is a rapidly evolving condition that may lead to death. The development of acute stroke network has facilitated recognition, diagnosis and rapid treatment improving outcome of ischemic stroke. The aim of this study is to highlight any predicting factors that facilitate to outline the prognosis of acute ischemic stroke.

**Methods:** We made a retrospective analysis of the patients admitted to Ospedale Civile of Modena’s NICU (Neurosciences Intensive Care Unit) with diagnosis of ischemic stroke. We selected a period of 22 months, from January 2017 to October 2018. At the admission we collected information about: Glasgow Coma Scale (GCS), worst GCS in the first 24 hours, SOFA score, SAPS II. Furthermore we analysed the following data: complications, surgical and non surgical procedures, GCS < or = 8 or mortality at discharge from NICU. We considered the last two parameters indicating the worst prognosis.

**Results:** 49 patients were studied, with average age of 70.8 sd ± 11.9. 34 (69.4%) patients were male. Although GCS on admission of 16 patients was missed, median of GCS was 8 iq 8. Median of the worst GCS in the first 24 hours was 9 iq 7. Median SOFA score and median SAPS II were respectively 5 iq 2.5 and 37 iq 22. During the recovery 21 patients (42.9%) developed neurological complications, of which 10 (47.6%) had malignant cerebral edema. Decompressive craniectomy was applied in 7 patients (14.3%). 33 patients (67.4%) underwent neuroradiology procedures. At discharge from NICU 19 patients had a GCS < or = 8 and were died (38.8%). The worst prognosis is related to GCS < or = 8 on admission (P = 0.003) and to malignant cerebral edema (P = 0.004).

**Conclusions:** Our study has shown some predictive factors closely related to mortality and morbidity in patients with acute ischemic stroke. GCS at admittance < or = 8 and onset of malignant cerebral edema lead to a worst prognosis at discharge from NICU.

### P219 Coherence analysis of cerebral oxygenation using multichannel functional near-infrared spectroscopy evaluates cerebral perfusion in hemodynamic stroke

#### TJ Kim^1^, JM Kim^2^, SH Park^1^, HB Jeong^1^, HM Bae^2^, SB Ko^1^

##### ^1^Seoul National University Hospital, Neurology, Seoul, South Korea; ^2^Korea Advanced Institute of Science and Technology (KAIST), Electrical Engineering, Daejeon, South Korea

**Introduction:** The functional near infrared spectroscopy (fNIRS) could evaluate brain function based on measuring changes in oxygenated and deoxygenated hemoglobin concentrations. The wavelet phase coherence (WPCO) can reveal the relationship of two signals between oscillations. We aimed to evaluate prefrontal functional connectivity using WPCO analyses of cerebral oxyhemoglobin (OxyHb) in patients with hemodynamic stroke.

**Methods:** A 35 consecutive patients with anterior circulation ischemic strokes and control patients (n=34) were included for analysis. The cerebral OxyHb data were collected using multichannel fNIRS (NIRSIT, OBELAB Inc., Republic of Korea). The coherences between eight segments of prefrontal OxyHb oscillations in five frequency intervals (I, 0.6–2Hz; II, 0.145–0.6Hz; III, 0.052–0.145Hz, IV, 0.021–0.052Hz, and V, 0.005–0.0095 Hz) were analyzed using wavelet coherence analysis. The patients were categorized into three groups with control groups, patients with perfusion defect and patients without perfusion defect. We compared the result of coherence analysis of OxyHb between three groups.

**Results:** Among the included 35 patients (age, 63.8; and male, 62.9%), 21 patients (60.0%) had perfusion defect in anterior circulation stroke. In patients with perfusion defect, phase coherence was significantly higher compared to patients without perfusion defect and control group, especially in interval III under the myogenic mechanism of cerebral autoregulation (0.70±0.17 vs. 0.57±0.20 vs. 0.58±0.15, P = 0.021) (Table 1). In addition, severe stroke patients were more likely to have higher phase coherence in interval III (P =0.078).

**Conclusions:** Our results demonstrated that the higher phase coherence of OxyHb in myogenic signal, which was originated locally from smooth muscle cells in brain was related to impaired cerebral perfusion. This suggests that monitoring cerebral oxygenation using fNIRS could be a useful noninvasive measuring tool for evaluating impaired cerebral autoregulation in stroke patients.

**Table 1 (abstract P219).** WPCO value according to perfusion

	No territorial lesion (n= 34, 49.3%)	Impaired perfusion (n = 21, 30.4%)	Normal perfusion (n = 14, 20.3%)	P-value
Frequency interval				
I (0.6-2), Cardiac activity	0.36 ± 0.16	0.42 ± 0.17	0.36 ± 0.20	0.333
II (0.15-0.6), Respiration	0.33 ± 0.16	0.38 ± 0.21	0.33 ± 0.20	0.666
III (0.05-0.15) Myogenic activity	0.58 ± 0.15	0.70 ± 0.17	0.57± 0.20	0.021
IV (0.02-0.05), Neurogenic activity	0.68± 0.17	0.74 ± 0.15	0.62 ± 0.14	0.102
V (0.0095-0.02), Endothelial metabolic activity	0.81± 0.13	0.78 ± 0.13	0.75 ± 0.17	0.500

### P220 Is esmolol associated with worse outcome at the acute phase of ischemic stroke that receives thrombolysis?

#### P Papamichalis^1^, E Neou^1^, O Triantafillou^1^, S Karagiannis^1^, E Dardiotis^2^, D Papadopoulos^1^, T Zafeiridis^1^, D Babalis^1^, A Pappa^1^, A Skoura^1^, N Ntafoulis^1^, A Komnos^1^

##### ^1^General Hospital of Larissa, Department of Intensive Care, Larissa, Greece; ^2^University Hospital of Larissa, Department of Neurology, Larissa, Greece

**Introduction:** The aim of our study was to examine the hypothesis that esmolol has neuroprotective action at brain ischemia and test this assumption at strokes that receive Intravenous Thrombolysis (I.T).

**Methods:** Retrospective study from our department¢s thrombolysis database.We compared 36 patients [mean National Institutes of Health Stroke Scale (NIHSS) 11.5/range 3-23] who received I.T with alteplase for acute ischemic stroke and esmolol for rhythm / rate control within the first 24 hours from the stroke with 113 patients [mean NIHSS 11/range 2-28] who received alteplase but not esmolol. Severity scores, predisposing factors, complications, mortality / functional outcome at 3 months of the 2 groups were statistically analysed.

**Results:** Length of stay, 7 days NIHSS, complication rate and functional outcome at 3 months were worse at esmolol group. The esmolol group had higher Symplified Acute Physiology Score (SAPS) II and at higher rate atrial fibrillation (Table 1).

**Conclusions:** Our study failed to detect any neuroprotective effect of esmolol on ischemic brain. On the contrary esmolol was correlated with worse outcome and more complications. Further studies are needed to determine if this is a direct negative effect of esmolol or if it is just the result of greater severity index and more frequent presence of atrial fibrillation resulting at more severe strokes at patients receiving esmolol.

**Table 1 (abstract P220).** Comparison of the 2 groups: esmolol (n=36) versus control group (n=113)

	CONTROL GROUP	ESMOLOL GROUP	p VALUE
LENGTH OF STAY (DAYS) (m. ± SE)	4.1 ± 0.6	15 ± 3	< 0.001^a d^
NIHSS (7 DAYS) mean (range)	2 (0 - 28)	11 (0 - 25)	< 0.001^a d^
COMPLICATIONNO (%)YES (%)	110 (97.3) 3 (2.7)	32 (88.9) 4 (11.1)	0.05c ^d^
FUNCTIONAL OUTCOME(mRS ≤ 2) (%)(mRS > 2) (%)	80 (70.8) 33 (29.2)	16 (44.4) 20 (55.6)	0.004b ^d^
SAPS II (n= 99) mean (range)	24 (6 - 59)	28 (13 - 61)	0.026a ^d^
ATRIAL FIBRILLATIONNO (%)YES (%)	81 (71.7) 32 (28.3)	14 (38.9) 22 (61.1)	< 0.001b ^d^

^a^:Mann-Whitney U test. ^b^:x2 after Yates correction. ^c^:Fisher¢s exact test. mRS: modified Rankin Scale, NIHSS: National Institutes of Health Stroke Scale, SAPS II: Simplified Acute Physiology Score II, ^d^:Statistically significant

### P221 Neutrophil-to-lymphocyte ratio is associated with early neurological deterioration in acute ischemic stroke

#### TJ Kim, KW Nam, SHP ark, HB Jeong, SB Ko

##### Seoul National University Hospital, Neurology, Seoul, South Korea

**Introduction:** Ischemic stroke patients experienced frequent early neurological deterioration (END) events. Since ischemic stroke has also been shown as inflammatory disease, the neutrophil-to-lymphocyte ratio (NLR) may associated with END events. However, the direct study regarding this association has not been addressed. We evaluated the association between NLR and END in ischemic stroke patients.

**Methods:** We included ischemic stroke patients between 2010 and 2015. END was defined as an increase ≥ 2 on the total National Institutes of Health Stroke Scale (NIHSS) score or ≥ 1 on the motor NIHSS score within 72 hours of admission. The NLR was calculated as the ratio of the absolute neutrophil count to the absolute lymphocyte count on admission.

**Results:** A total of 1,152 patients were included. Among them, END occurred in 154 (13%) patients and the median NLR value was 2.47 [1.59-3.97]. In multivariate analysis, NLR remained an independent predictor of END [adjusted odds ratio (aOR) = 1.071, 95% confidence interval (CI) = 1.025 to 1.119, P = 0.002] (Table 1). These results were more prominent in large artery atherosclerosis group (aOR = 1.107, 95% CI = 1.031 to 1.188, P = 0.005), while stroke patients with small vessel occlusion (aOR = 1.114, 95% CI = 0.895 to 1.387, P = 0.335) or cardioembolic (aOR = 1.028, 95% CI = 0.912 to 1.159, P = 0.649) mechanisms did not show statistical significance.

**Conclusions:** A high NLR level was associated with END in ischemic stroke patients, especially in large artery atherosclerosis patients. The NLR may hep to identify high-risk patients in time and provide clues for further studies about inflammatory pathophysiology of ischemic stroke.

**Table 1 (abstract P221).** Multivariable analysis of possible predictors of early neurological deterioration

	Crude OR (95% CI)	P value	Adjusted OR (95% CI)	P value
Age, y	1.021 [1.006-1.036]	0.007	1.021 [1.004-1.038]	0.016
Visit time, h	0.983 [0.973-0.993]	0.001	0.991 [0.981-1.001]	0.090
Atrial fibrillation, n (%)	1.288 [0.877-1.892]	0.197	0.649 [0.403-1.044]	0.075
Initial NIHSS score	1.069 [1.044-1.094]	< 0.001	1.024 [0.992-1.058]	0.147
Thrombolysis therapy, n (%)	1.788 [1.499-2.133]	< 0.001	1.616 [1.276-2.046]	< 0.001
Fasting blood sugar, mg/dL	1.008 [1.005-1.012]	< 0.001	1.006 [1.002-1.011]	0.003
NLR	1.078 [1.036-1.122]	< 0.001	1.071 [1.025-1.119]	0.002

### P222 The role of the transcranial Doppler in the neuromonitorization of the critically ill patient

#### J Higuera Lucas, S Gallego, G Narvaéz, A Caballero, D Cabestrero, C Soriano, R De Pablo

##### Hospital Universitario Ramón y Cajal, Medicina Intensiva, Madrid, Spain

**Introduction:** Hemodynamic, renal, hepatic or cardiac monitoring is widely described in the critically ill patient, however, the knowledge about neuromonitoring is scarcer. Especially in patients who are sedated and on mechanical ventilation. Our aim is to describe the role of the transcranial Doppler in this process.

**Methods:** A prospective, observational study was performed with all the patients admitted to an Intensive Care Service of a Tertiary, University Hospital. All patients analyzed required mechanical ventilation to support their pathology. All consecutive patients admitted to the unit under mechanical ventilation, noradrenaline dose <0.06 mcg/kg/min, were included, with the expectation of remaining under mechanical ventilation for at least 24 more hours after data collection. The severity indexe and, mortality rates were collected. As well, pulsatibility indexes of the middle cerebral artery were obtained through the left or right temporal window.

**Results:** A total of 30 patients were included. Mean age 63±14.3 years, SOFA 12.3±12.8 SAPS II 56±17.6; APACHE II 25.5±9.7; Total days on M.V. 7.5±13 days; Length of stay 29.5±32.5 days; Mean vasoactive drugs 0.003 mcg/kg/min. The average values of pulsatibility indexes in the group of survivors was 1.07, being 1.5 in the group of non-survivors (P=0.02). Values above 1.2 were associated in a statistically significant way with mortality (P = 0.013). The mortality rate in the group of patients which values were higher than 1.2 was 66.7%. On the other hand, patients with fewer values than 1.2 had a mortality rate of 20%. Patients with pulsatibility indexes greater than 1.2 presented higher severity indices. (P=0.02).

**Conclusions:** Transcranial Doppler is a useful tool in the neuromonitoring of critically ill patients. In our study, the pulsatibility indexes of the middle cerebral artery were associated in a statistically significant way with mortality. Patients with higher severity indexes presented altered pulsatility values.

### P223 WITHDRAWN

### P224 Predictors of mortality after subarachnoid hemorrhage: a restrospective multicenter cohort study

#### P Kurtz^1^, FS Taccone^2^, B Gonçalves^3^, M Soares^3^, F Bozza^3^, M Medeiros Machado^4^, M Maia^5^, M Ferez^6^, C Nassif^7^, C Shinotsuka^8^, JL Salluh^3^

##### ^1^Paulo Niemeyer State Brain Institute, Neuro ICU, Rio de Janeiro, Brazil; ^2^Free University of Brussels, Brussels, Belgium; ^3^D´Or Institute for Research and Education, Rio de Janeiro, Brazil; ^4^Hospital Agenor Paiva, Salvador, Brazil; ^5^Hospital Santa Luzia, Viana do Castelo, Portugal; ^6^Hospital Sao Francisco, Brasília, Brazil; ^7^Hospital Nove de Julho, São Paulo, Brazil; ^8^Paulo Niemeyer State Brain Institute, Rio de Janeiro, Brazil

**Introduction:** Aneurysmal subarachnoid haemorrhage (SAH) is an acute, and often catastrophic, cerebrovascular event, with high mortality and morbidity. Data on predictors of mortality in low and middle-income countries is scarce. The purpose of this study was to investigate clinical predictors of hospital mortality in patients admitted with subarachnoid hemorrhage in a large sample of Brazilian ICUs.

**Methods:** We performed a retrospective cohort study of patients admitted with spontaneous (SAH) to 57 hospitals in Brazil, during 2014 and 2015. We retrieved patients’ clinical and outcome data from an electronic ICU quality registry. SAPS 3 non-Neuro and SOFA non-Neuro scores were calculated subtracting the Glasgow Coma Scale contribution from the original score values. We used mixed multivariable logistic regression analysis to identify factors associated with hospital mortality.

**Results:** A total of 1114 patients were included. Fifty five percent (n=610) of patients were female and 71% were admitted from the emergency room, while 18% were transfered from another hospital. Median age was 57 (interquartile range 45 – 72) and median ICU length of stay was 5 (IQR 2 – 10). Median SAPS 3 non-Neuro score was 46 (38 – 55) and SOFA non-Neuro score was 2 (0 – 5). A total of 446 (30%) patients presented with poor grade SAH (World Federation of Neurosurgeons grading scale IV and V) and hospital mortality was 35%. In univariate comparisons, nonsurvivors were older and had higher SAPS3 non-Neuro and SOFA non-Neuro scores (all P<0.001). Poor grade SAH, use of vasopressors, mechanical ventilation, intracranial pressure monitoring, external ventricular drainage, blood transfusions and renal replacement therapy were all more frequent among nonsurvivors (all P<0.001). Mortality was also higher with initial lactate above 2 mmol/L, in those admitted to public hospitals and when admission to ICU was delayed more than 24 hours after ictus. After adjusting for common predictors (age, gender and WFNS) SAPS 3 non-Neuro, SOFA non-Neuro, early vasopressor use and admission to a public hospital were independently associated with hospital mortality. Moreover, the area under the curve for prediction of mortality with SAPS3, SOFA and WFNS was 0.86 (Figure 1).

**Conclusions:** Mortality is elevated and highly variable in this large sample of SAH patients. Age, severity of clinical presentation, both systemic and neurological, as well as the presence of organ dysfunction were associated with mortality.

**Fig. 1 (abstract P224).**
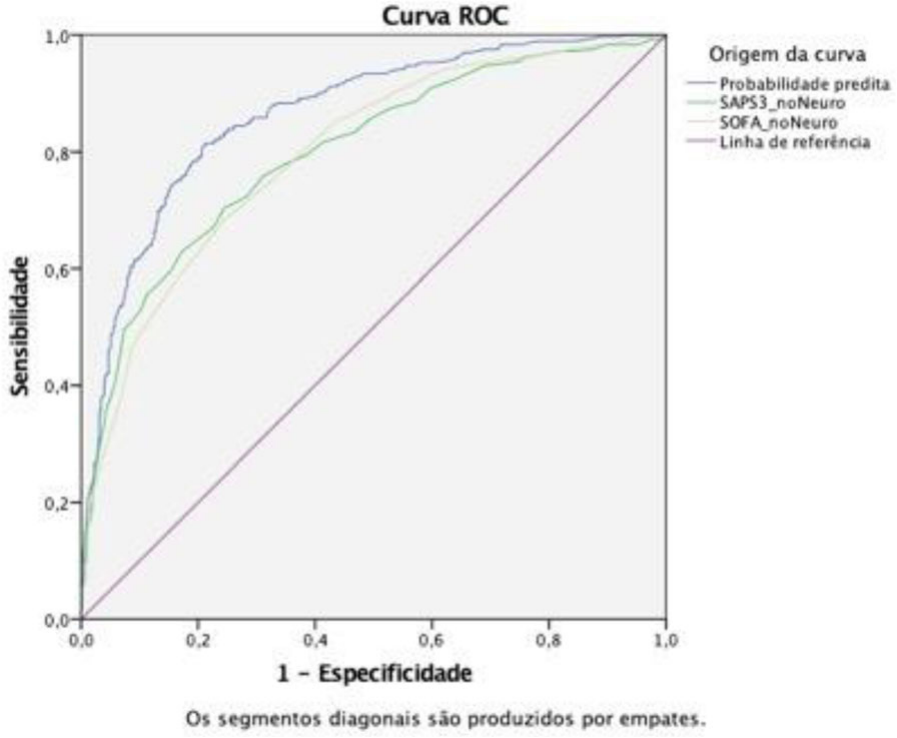
Area under the curve (AUC) for hospital mortality for variables: SAP 3 non-Neuro; SOFA non-Neuro and the combination of both plus WFNS Poor Grade

### P225 Incidence, risk factors & outcome from acute kidney injury in non-traumatic subarachnoid haemorrhage patients on the neuro-intensive care unit

#### S Muldoon^1^, G Bose^2^, V Luoma^1^, G Bird^3^

##### ^1^The National Hospital for Neurology & Neurosurgery, London, United Kingdom; ^2^University Hospital North Midlands, Neuroanaesthesia, Stoke-On-Trent, United Kingdom; ^3^The National Hospital for Neurology & Neurosurgery, Neurocritical Care, London, United Kingdom

**Introduction:** The incidence of acute kidney injury (AKI) after non-traumatic subarachnoid haemorrhage (ntSAH) is poorly quantified, purportedly between 5-27% depending on diagnostic criteria used [1,2]. This observational study aimed to; quantify incidence, qualify risk factors and outcome following AKI in patients with ntSAH on the neuro-intensive care unit (NICU).

**Methods:** A retrospective electronic case-note review of patients admitted with ntSAH to NICU between 2014-17. Exclusion criteria: NICU stay <48 hours & >4 days ictus to NICU admission. AKI was defined by KDIGO criteria [3]. Data analysed included: demographics, pre-morbid risk factors for AKI, WFNS SAH grade, treatment, radio-contrast load, development of delayed cerebral ischaemia (DCI) (defined as decision to induce hypertension to treat new neurological deficit) and infection (defined as decision to prescribe antibiotics), NICU mortality, NICU and hospital length of stay (LOS).

**Results:** 204 patients included (Table 1), incidence of AKI was 26.5%. The majority were stage 1, with 4.4% [n=9] developing stage 2&3 AKI; only 2 required renal replacement therapy. The median [range] time from ictus to AKI was 4 [0-23] days. AKI was associated with poor grade ntSAH [P=0.0035], diabetes [p=0.0040], vasopressors [P=0.0001], infection [P=0.0001] & DCI [P=0.0253]. Hypertension & total contrast load were not associated with AKI. AKI was associated with increased NICU mortality [P=0.0008], but not LOS except for patients with stage 2 & 3 [p=0.018]. A trend towards increased incidence of AKI in males and patients treated by clipping was observed.

**Conclusions:** Incidence of AKI in our study was comparable with published data [2] and associated with poor-grade ntSAH, DCI and NICU mortality. Further work will focus on the cause of AKI in these patients, where early occurrence of AKI may be a consequence of early brain injury.

**References**

1. Kamar C et al. Ulus Travma Acil Cerrahi Derg. 23:39-45.2017

2. Bercker S et al. PLoS ONE 13,2018

3. KDIGO. Kidney Int. Suppl 2:1–138,2012

**Table 1 (abstract P225.):**
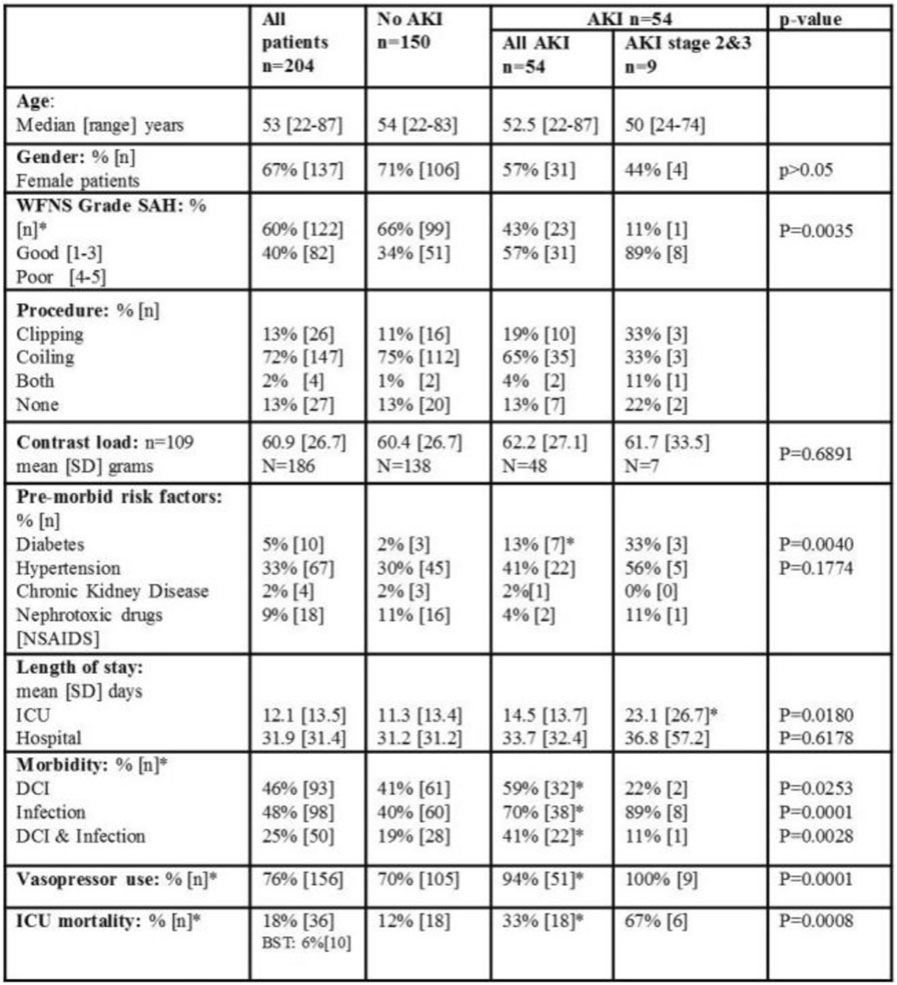
Comparison of groups

### P226 An acute hemodynamic effect of intra-arterial nimodipine injection for symptomatic vasospasm in patients with aneurysmal subarchnoid hemorrhage

#### SH Park, TJ Kim, HB Jeong, SB Ko

##### Seoul National University Hospital, Department of Neurology and Critical care, Seoul, South Korea

**Introduction:** Intra-arterial (IA) nimodipine injection has been used in the treatment of refractory vasospasm after subarachnoid hemorrhage (SAH). IA nimodipine promotes dilatation of cerebral vessels and improves cerebral blood flow (CBF). However, its effect of duration when injected intra-arterially still remained to be elucidated.

**Methods:** A 76-year-old woman with SAH (H-H scale 4, mFisher 3, 3mm X 3.5mm, ruptured left middle cerebral artery [MCA] bifurcation aneurysm) was admitted to the neuroICU. Aneurysm was secured with coil embolization. On hospital day 6, she became drowsy and developed new right hemiparesis (GCS decreased from 15 to 13). Mean flow velocity in the left MCA on Transcranial Doppler ultrasonography was increased to 182cm/sec compared to baseline (60cm/sec). Continuous CBF flow monitoring was monitored using cerebral flow index (CFI) in C-FLOW. This patient was treated with IA nimodipine infusion under refractory vasospasm.

**Results:** Before initiating IA nimodipine, hemodynamic parameters were as follows (mean arterial pressure [MAP], 110mmHg; heart rate [HR], 64/min; right CFI, 48; left CFI, 32). Two minutes after IA nimodipine (5mg/hr), MAP decreased 85mmHg, and CFI was increased (Rt, 54; Lt, 50) while HR did not change (62/min). After 15 minutes of IA nimodipine, MAP dropped to 80mmHg, and CFI started to decrease on the left (46) while right CFI did not change. The IA nimodipine rate was decreased to 1mg/hr and norepinephrine was increased to 10mcg/minute. IA nimodipine was infused over 75 minutes. After the procedure, MAP was 92mmHg, HR was 66/minute, CFI was 54 on the right, and 50 on the left. Neurological status was improved and patients became more alert. 1 hour after stopping IA nimodipine, CFI returned to baseline (Right, 44; Left, 33) (Figure 1)

**Conclusions:** Acute hemodynamic effect of IA nimodipine can be assessed using C-FLOW. The positive effect of IA nimodipine on CBF lasted only 1 hour on this observation. More studies are needed to confirm this observation.

**Consent:** Informed consent to publish has been obtained from the patient

**Fig. 1 (abstract P226).**
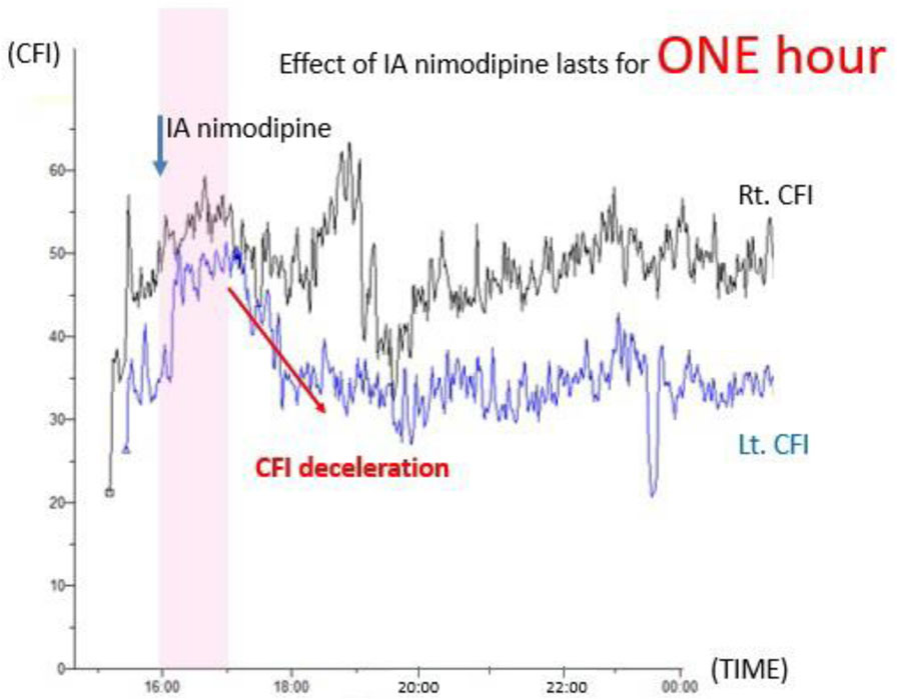
Variation of CFI after IA nimodipine injection

### P227 Association of duration and intensity of intracranial hypertension insults with outcome in subarachnoid hemorrhage: an observational study of two cities

#### G Carra^1^, F Elli^2^, L Huber^3^, F Güiza^4^, B Ianosi^3^, V Rass^5^, B Depreitere^4^, G Meyfroidt^4^, G Citerio^2^, R Helbok^5^

##### ^1^KU Leuven, Department and Laboratory of Intensive Care Medicine, Leuven, Belgium; ^2^University of Milan-Bicocca, Monza, Italy; ^3^University of Health Sciences, Medical Informatics and Technology, Hall in Tirol, Austria; ^4^KU Leuven, Leuven, Belgium; ^5^Medical University of Innsbruck, Innsbruck, Austria

**Introduction:** Some patients with severe-grade subarachnoid hemorrhage (SAH) require intracranial pressure (ICP) monitoring. Current ICP treatment thresholds in SAH are derived from studies in patients with traumatic brain injury (TBI). The purpose of the study was to assess the association of intensity and duration of episodes of intracranial hypertension with neurological outcome in adult patients with SAH.

**Methods:** Retrospective analysis of 2 prospectively collected datasets, including time series of ICP, from 52 patients at the San Gerardo University Hospital, Italy and from 46 patients at the Innsbruck University Hospital, Austria. The association of intensity and duration of intracranial hypertension episodes with 12-month Glasgow Outcome Score (GOS) was visualized using the methodology introduced by Güiza et al. [1].

**Results:** In both cohorts, it could be demonstrated that the combination of duration and intensity defined the tolerance to intracranial hypertension, and that a semi-exponential curve separated episodes associated with better outcomes from those associated with worse outcomes. The association with worse outcomes occurred at a lower pressure-time burden than what has been previously observed in patients with TBI. Nevertheless, the percentage of monitoring time spent by every patient in the zone associated with poor GOS was independently associated with worse 12-month neurological outcome, even after correcting for age and Fisher score (p-values of 0.001 and 0.02 in Monza and Innsbruck respectively). The pressure-time burden curve for the Monza patients was shifted to the left compared to Innsbruck, which could only partially be explained by differences in baseline characteristics between the cohorts.

**Conclusions:** In two cohorts of adult patients with SAH, an independent association of the pressure and time burden of ICP, and worse clinical outcomes, could be demonstrated. The association occurs at lower ICP to what was observed in TBI.

**Reference**

1. Güiza et al. Intensive Care Med 41:6, 2015

### P228 Predisposing factors of failed apnea test during brain death determination in potential organ donor

#### JJ Kim, EY Kim

##### Seoul St. Mary´s hospital, Department of General Surgery, Division of Surgical Critical Care and Trauma, Seoul, South Korea

**Introduction:** Apnea test is an essential component in the clinical determination of brain death, but it may incur a significant risk of complications such as hypotension, hypoxia and even cardiac arrest [1]. We analyzed the risk factors associated with failed apnea test during brain death assessment in order to predict and avoid these adverse events.

**Methods:** Medical records of apnea tests performed for brain-dead donor between January 2009 and January 2016 in our institution, were reviewed retrospectively. Age, gender, etiology of brain death, use of catecholamine and results of arterial bleed gas analysis (ABGA), systolic/diastolic blood pressure (SBP/DBP), mean arterial pressure (MAP) and central venous pressure (CVP) prior to apnea test initiation were collected as variables. A-a gradient and PaO2/FiO2 were calculated for more precise assessment of the respiratory system. In total, 267 cases were divided into a group which was completed apnea test and the other which was failed the test.

**Results:** 13 cases failed the apnea test and the majority of reasons were severe hypotension (SBP < 60mmHg). In terms of hemodynamic state, SBP was significantly higher in the completed test group than the failed group (126.5 ± 23.9 vs. 103 ± 15.2, respectively; p = 0.001). In ABGA, the completed test group showed significantly higher PaO2/FiO2 (313.6 ± 229.8 vs. 141.5 ± 131.0, respectively; p = 0.008) and lower A-a gradient (278.2 ± 209.5 vs. 506.1 ± 173.1, respectively; p = 0.000). In multivariable analysis, low SBP (p = 0.040) and high A-a gradient (p = 0.002) were independent risk factors associated with failed apnea test.

**Conclusions:** Although the unexpected adverse events during apnea test for brain death determination do not occur frequently, they could be fatal. If a brain-dead patient shows low SBP and high A-a gradient, clinicians should pay more attentions and preparations prior to apnea test.

**Reference**

Murthy T. Medical Journal, Armed Forces India. 2009;65:155-160.

### P229 Neuroinvasive West Nile disease (NIWND): a case series of the 2018 Tunisia outbreak

#### D Ben Braiek, K Meddeb, N Fraj, W Ammar, C El Marzougui, W Zarrougui, M Boussarssar

##### Farhat Hached University Hospital, Medical Intensive Care Unit, Sousse, Tunisia

**Introduction:** Tunisia has already suffered recurrent outbreaks since 1997. 2018 outbreak started relatively earlier this year. We were interpellated by the frequency of neuroinvasive presentation of the disease.

**Methods:** We report a case series of 11 patients presented to ICU with NIWND.

**Results:** We report 11 cases of NIWND with different severe presentations overlapping neurological manifestation including encephalitis (n=8/11), meningitis (n=10/11) and flaccid paralysis (n=8/11). Almost all patients live in the locality of Sousse. Six patients presented a long course of isolated fever before developing neurological signs. Cerebrospinal fluid was consistent with encephalitis within the 11 patients. Cerebromedullar MRI identified brain lesions (n=8/10), myelitis (n=1/10) and polyradiculoneuritis (n=1/10).Three patients had electromyography for flaccid paralysis showed diffuse axonal polyneuropathy with motoneuron involvement. Ten cases had a positive WNV IgM antibody and nine had a positive WNV IgG antibody in serum. Urine polymerase chain reaction was positive for WNV in 8/10 patients. Ten patients were mechanically ventilated. All patients were managed symptomatically. Two received high doses of methylprednisolone for 3 days, one patient received polyclonal immunoglobulin intravenous and one patient had plasmapheresis. Two patients died consecutive to brainstem lesions. Two patients recovered significantly and discharged with no complications. Five other patients evolved to persistent flaccid paralysis with a minimal consciousness state and weaning difficulties requiring tracheostomy. The last remaining patient is still evolving.

**Conclusions:** Modification of the regional climatic conditions accounted probably for the early 2018 outbreak of NIWND. This initial case series displays the severity and the poor outcomes of NIWND with higher incidence compared to past epidemics.

### P230 Noninvasive estimation of intracranial pressure with transcranial Doppler: a prospective multicenter validation study

#### C Robba^1^, C Iaquaniello^2^, A Mazeraud^3^, M Czosnyka^4^, D Savo^5^, M Saini^6^, G Citerio^7^

##### ^1^Policlinico San Martino IRCCS for Oncology, Anaesthesia and Intensive Care, Genoa, Italy; ^2^Università degli Studi “Milano Bicocca”, Scuola di specializzazione in Anestesia, Rianimazione, Terapia Intensiva e del Dolore, Milano, Italy; ^3^Tertiary Hospital Européen Georges Pompidou, Paris, France; ^4^University of Cambridge, Department of Clinical Neurosciences, Cambridge, United Kingdom; ^5^ ASST-Monza, NeuroIntensive Care Unit, Monza, Italy; ^6^ ASST-Monza, Neurointensive Care Unit, Monza, Italy; ^7^Università degli Studi “Milano Bicocca”, Milano, Italy

**Introduction:** Invasive intracranial pressure (ICP) monitoring through an intraventricular or parenchymal catheter is crucial in neurological critically ill patients. Non-invasive bedside ICP measurement techniques have been developed but results are questionable. Transcranial Doppler (TCD) Ultrasonography has shown promising results, as variation in the flow velocity waveform may reflect ICP changes. The aim of our study is to assess if a published formula [1] based on the TCD diastolic flow velocity correlates with invasive ICP in a cohort of brain injured patients.

**Methods:** We designed a prospective multicenter observational study of patients admitted in two tertiary neurocritical care units (Monza, Italy and Addenbrookes Hospital, Cambridge, UK) with a diagnosis of acute brain injury that required invasive ICP (ICPi) monitoring. Non-invasive ICP (ICPtcd) values were derived from the flow velocities measured by the TCD of the middle cerebral artery (MCA): MCA pulsatility index (PIa) and an estimator based on diastolic flow velocity (FVd). We applied the Bland-Altman method, and assessed sensitivity and specificity of the method through the ROC and the AUC analyses.

**Results:** 115 patients were enrolled, 314 paired ICPi and ICPtcd measure were performed. 25 patients (29%) had at least one episode of intracranial hypertension (ICP>20 mmHg). In the Bland-Altman approach [Fig. 1], mean bias was -3.24 mmHg (limits of agreement are ± 2 SD 24.6 mmHg). 7.5% measures were outside the limit of agreement in the overall population. However, when ICP was high, 43% of measures were out of the limit of agreement. The AUC [Fig. 2] was 0.344 and 0.362 for ICPtcd and for PIa respectively, with a mean bias of -3.24 mmHg (SD 12.28 mmHg with limits of agreement -27.8 - 21.32 mmHg).

**Conclusions:** According to our results, the method is not enough reliable to be used in clinical practice for substituting invasive ICP monitoring. Further studies are needed to confirm the hypothesis.

**Reference**

[1] Czosnyka M et al. J. Neurosurg., 88:802–808, 1998

**Fig. 1 (abstract P230).**
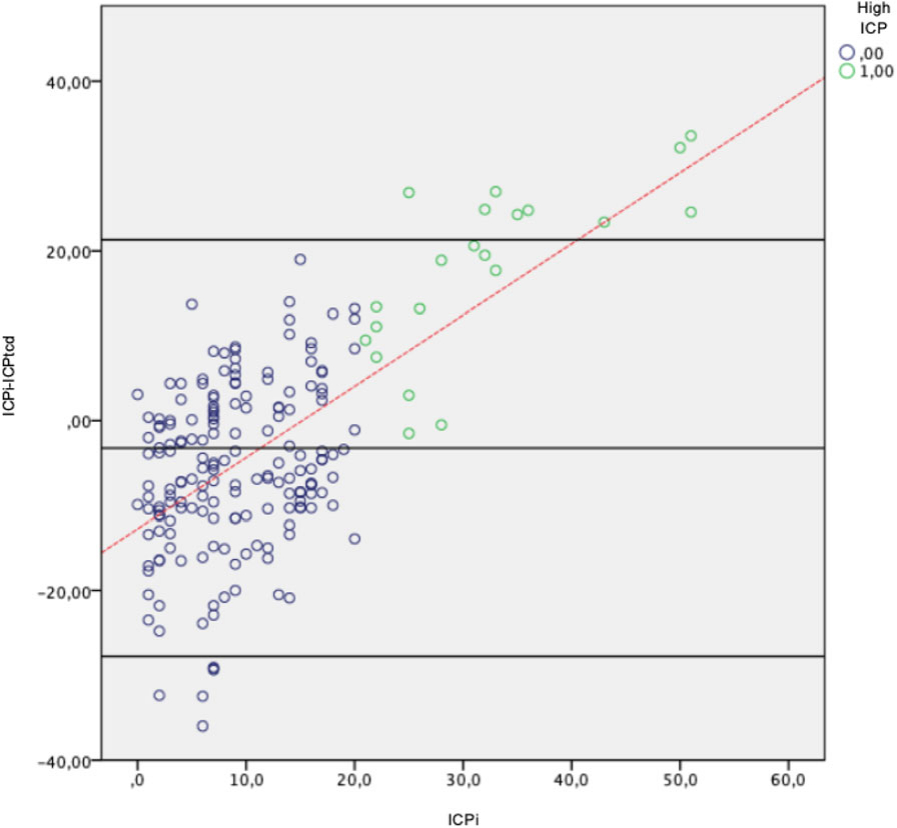
Bland-Altman analysis

**Fig. 2 (abstract P230).**
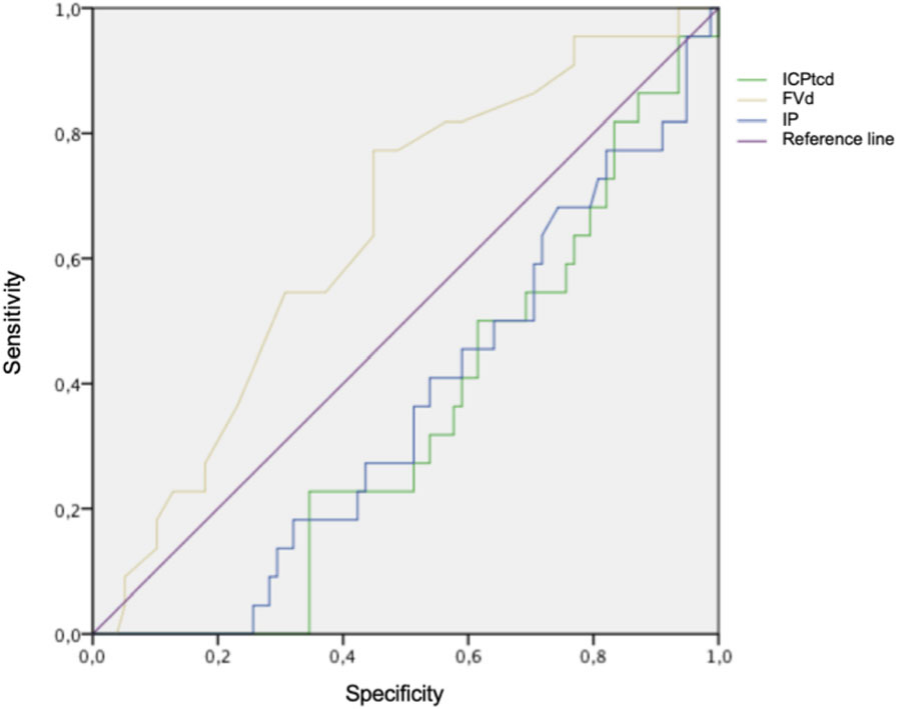
ROC curve

### P231 Non-invasive intracranial pressure monitoring for ICU management of cardiovascular patients

#### P Travassos^1^, R Vale^1^, W Costa^1^, C Hayashi^1^, G Vilela^2^, V Veiga^1^, J Souza^3^, S Rojas^1^

##### ^1^Hospital BP - A Beneficência Portuguesa de São Paulo, Neurological ICU, Sao Paulo, Brazil; ^2^Braincare Desenvolvimento e Inovacao Tecnologica S.A., Research Consultant, Sao Paulo, Brazil; ^3^Hospital BP - A Beneficência Portuguesa de São Paulo, Cardiovascular Surgery, Sao Paulo, Brazil

**Introduction:** Surgical treatment of aortic aneurysm needs extracorporeal circulation (ECC), aorta clamp and hypothermia, and it is often related to poor systemic perfusion and blood flow velocity. One of the main concerns of intensive care team is to prevent secondary neurological injury after long time without blood flow pulsatility, such as brain edema and seizure. The most common parameters for neuromonitoring would be intracranial pressure and EEG, however, for non-neurological patients this information is unusual and prevents optimal management.

**Methods:** We aimed to assess brain compliance and neurological condition of ICU patients on immediate post-operative recovery of Bentall-De Bono procedure and/or other aortic aneurysm surgical treatment using a novel non-invasive intracranial pressure (ICP) device. This device uses mechanical displacement sensor capturing extracranial continuous volumetric variation of the skull and this information proportionally reflects intracranial dynamic[1].

**Results:** Twenty patients were included in this study. ECC mean time was 111 minutes for 19 patients and only one did not need it. Eleven presented altered ICP curves with poor brain compliance (P2/P1 ratio > 1.0) assessed by ICP curve morphology analysis. Volemic optimization and neuroprotective measures were taken based on this ICP information for acute case management. Among these patients with altered ICP curves, eight were discharged from ICU with good clinical condition and Glasgow Coma Scale of 15. Overall mortality rate was six out of twenty (30%) and three of these had altered ICP curves.

**Conclusions:** Brain monitoring of cardiovascular post-operative patients is important to prevent secondary neurological complications and can be a helpful tool for neuroprotective acute management on ICU.

**Reference**

[1] Cabella et al. Acta Neurochirurgica Supplement 122:93-6, 2016.

### P232 Functional electrical stimulation in the critically ill – what can we learn from healthy volunteers?

#### P Turton^1^, B Johnston^1^, S AlAidarous^2^, I Welters^1^

##### ^1^Institute of Aging and Chronic Disease, Liverpool, United Kingdom; ^2^Institute of Global Health, Liverpool, United Kingdom

**Introduction:** Muscle loss is common in critical illness. Functional Electrical Stimulation (FES) is a potential method of preventing this. The technique supplies electrical current to muscle, combined with passive cycling. Prior to a clinical trial, we first investigated the effects of one session of FES in healthy volunteers.

**Methods:** Healthy male volunteers (n=15) were recruited. The participants had their postural sway assessed on a pressure sensitive board, and measurement of Maximal Inspiratory Pressure (MIP). Ultrasounds were taken assessing thickness of the quadriceps and rectus abdominis. They performed 20 minutes of supine passive cycling, with FES supplying the lower limbs and abdomen. After a 1 minute rest, the tests were repeated. A further 14 participants performed just the initial baseline tests, to help assess muscular factors affecting balance and sway.

**Results:** The current needed for palpable contraction was significantly correlated to weight in the abdomen (r=0.79, p<0.001) and quadriceps (r=0.82, p<0.001). Current required to stimulate the abdominal muscles was also correlated to depth of the subcutaneous fat layer (r=0.85, p<0.001) and echogenicity of the muscle (r=0.65, p=0.012). Pre-cycling, left and right vastus lateralis thickness inversely correlated to postural sway in the antero-posterior (r=-0.638, p<0.001) plane. Compared to pre-cycling, postural sway in the antero-posterior and lateral planes increased significantly after cycling. There was a significant decrease in MIP after cycling and greater reductions in MIP were found in participants who had thinner rectus abdomni.

**Conclusions:** Sway at baseline is related to quadriceps thickness, which atrophies during critical illness, and could worsen balance. MIP is reduced during FES and the severity of reduction is related to the thickness of the abdominal wall muscles at baseline, suggesting that FES can fatigue the diaphragm and abdominal muscles. In awake healthy volunteers, FES is a safe, comfortable technique.

### P233 The prevalence of postoperative cognitive dysfunction after off-pump cardiac surgery

#### V Sharipova, A Valihanov, A Alimov

##### Republican Research Centre of Emergency Medicine, Uzbekistan, Anesthesiology, Tashkent, Uzbekistan

**Introduction:** In most cases postoperative cognitive dysfunction (POCD) is transient, but still some patients suffer from persistent cognitive impairment which is associated with increased length of hospital stay, early withdrawal from labor market and higher mortality. Available data on the prevalence of POCD after cardiac surgery is very diverse from 20% to 90% upon discharge and up 20% 3 months after surgery. We aimed to investigate the prevalence of short-term and long-term POCD after off-pump coronary artery bypass grafting (CABG) surgery.

**Methods:** Psychometric testing was performed in 230 (mean age 63.5±8.2) patients before, 10 days and 6 months after the surgery. We used following tests to assess cognitive capacity: auditory verbal learning test (AVLT), digit span test (DST), digit-letter substitution test (DLST), Stroop’s test and trail making test (TMT). A decline in comparison to preoperative test results for 20% or more in two or more tests was declared as POCD.

**Results:** The prevalence of POCD after 10 days was 31.7% (73 patients) and 9.1% (21 patients) after 6 months. When comparing patients who developed POCD with those who did not we found the former were older (69.2±8.7 vs 61.1±10.3 years; p<0.001), had lower education level (13.7±2.1 vs 10.6± 2.4 years; p<0.05) and had longer surgery duration (250.4±12.2 vs 232.1±15.9 minutes; p<0.05). The most affected cognitive domains were long term memory (AVLT) and executive function (TMT) and least affected – working memory (DST) and selective attention (Stroop’s test).

**Conclusions:** In our prospective study the prevalence of long-term POCD after cardiac surgery was slightly less (9.1%) in comparison to available data (from 9% to 20%). It might be due differences in psychometric testing and interpretation of its results among authors. Advanced age, low cognitive reserve and long duration surgeries are linked with higher incidences of POCD.

### P234 Hospital length of stay increases postoperative cognitive dysfunction

#### V Sharipova, A Valihanov, A Alimov

##### Republican Research Centre of Emergency Medicine, Uzbekistan, Anesthesiology, Tashkent, Uzbekistan

**Introduction:** Postoperative cognitive dysfunction (POCD) is a common and widely described phenomenon in surgical patients. Advanced age, major surgery, certain general anesthetics, genetic factors, sleep deprivation and other factors were described as contributing factors to POCD. The hospital stay itself is a major ‘social’ trauma for patients; social isolation, sleep deprivation and changes in daily regimen may effect neurocognitive behavior of patients. In this trial we tried to assess the link between POCD and the length of hospital stay in cardiac surgery patients.

**Methods:** 94 patients who underwent ‘off-pump’ coronary artery bypass grafting (CABG) surgery selected for this trial. Neuropsychological testing was performed prior to the operation and upon discharge. We used auditory verbal learning test (AVLT), digit span test (DST), digit-letter substitution test (DLST), Stroop test and trail making test (TMT). A 20% or more decline in two or more tests in comparison to preoperative test results was declared as POCD. Patients were allocated into two groups according to the length of hospital stay: the SHORT-STAY group (group 1) included patients (n=36) who were discharged on the 8th day after surgery or earlier and the LONG-STAY (group 2) group consisted of patients (n=58) who were discharged on the 9th day after surgery or later. Patients received similar anesthesia, postoperative care and were operated by the same surgical team. Reasons for prolonged duration of hospital stay were mainly surgical.

**Results:** 11 patients (30.6%) in group 1 and 21 patients (36.2%) in group 2 had POCD upon discharge (p<0.05). Mean length of hospital stay were 7±1.2 and 10±1.4 days in group 1 and group 2 patients respectively (p<0.05).

**Conclusions:** Prolonged length of hospital stay increased the prevalence of POCD in our trial. Studies with various types of surgical procedures and larger patient populations needed to further understand the effect of length of hospital stay to POCD.

### P235 The influence of multiple trauma with head trauma on posttraumatic meningitis: a nation-wide study with hospital-based trauma registry in Japan

#### Y Katayama^1^, T Kitamura^2^, T Hirose^1^, Y Nakagawa^1^, T Shimazu^1^

##### ^1^Osaka University Graduate School of Medicine, Department of traumatology and acute critical medicine, SUITA, Japan; ^2^Osaka University Graduate School of Medicine, Division of Environmental Medicine and Population Sciences, Department of Social and Environmental Medicine, SUITA, Japan

**Introduction:** Posttraumatic meningitis is one of severe complications and results in increased mortality and longer hospital stay among head trauma patients. However, it remains unclear whether there is a difference in the incidence of post-traumatic meningitis due to single traumatic brain injury (TBI) and multiple trauma including head injury.

**Methods:** This study was a retrospective observational study during 12 years We included trauma patients registered in Japanese Trauma Data Bank whose head AIS score was >3 in this study. Multivariable logistic regression analysis was used to assess potential factors associated with posttraumatic meningitis such as CSF fistula, skull base fracture, type of injury that divided into single TBI and multiple trauma.

**Results:** Among 60,390 patients with severe head injury, 9415 (15.6%) patients were multiple trauma patients and 50,975 (85.4%) were single TBI patients (Table 1). The incidence of posttraumatic meningitis was 0.6% (61/9,415) in multiple trauma patients and 0.4% (223/50,975) in single TBI patients, respectively. Multiple trauma was associated with posttraumatic meningitis compared with single TBI (adjusted odds ratio [OR] 1.415 [95% confidence interval {CI}; 1.055-1.900]) (Table 2).

**Conclusions:** In this population, multiple trauma patients were associated with posttraumatic meningitis among trauma patients.

**References**

Sonig A, et al. Neurosurg Focus. 2012 ;32: E4

Adepoju A, Adamo MA. J Neurosurg Pediatr. 2017; 20: 598-603

**Table 1 (abstract P235).** Patient´s characteristics

	Multiple trauma (N=9415	Single head injury (N=50975)	P value
Age, median(IQR)	59(34-74)	69(34-77)	<0.001
Male sex, n(%)	6430(68.3%)	34904(68.5%)	0.736
Skull fracture, n(%)	1983(21.1%)	12102(23.7%)	<0.001
Skull base fracture, n(%)	1535(16.3%)	6853(13.4%)	<0.001
CSF fistula, n(%)	410(4.4%)	1623(3.2%)	<0.001
Craterizartion at ED, n(%)	350(3.7%)	1118(2.2%)	<0.001
Re-op within 48hrs, n(%)	152(1.6%)	776(1.5%)	0.496

**Table 2 (abstract P235).** Result

Variables	Posttraumatic meningitis %(n/N)	Adjusted odds ratio	95% confidence interval	P value
Male	0.3(63/19056)	1.482	(1.111-1.978)	0.007
Skull fracture	0.8(108/14085)	1.164	(0.900-1.505)	0.247
Skull base fracture	1.2(99/8388)	1.676	(1.198-2.345)	0.003
CSF fistula	2.7(54/2033)	3.328	(2.206-5.019)	<0.001
Craterization at ED	3.3(49/1468)	2.150	(1.396-3.311)	0.001
Re-op within 48hrs	2.6(7/267)	3.170	(2.125-4.729)	<0.001
Multiple trauma	0.6(61/9415)	1.415	(1.055-1.900)	0.021

### P236 Determination of NIRS and BIS values in traumatic braine injury patiens

#### C Balci^1^, E Haftaci^1^, H Aytuluk^1^, H Durmus^1^, R Sivaci^2^

##### ^1^Kocaeli Derince Traning Hospital, Intensice Care Unit, Kocaeli, Turkey; ^2^AfyonKocatepe Universty, afyon, Turkey

**Introduction:** The aim of this study was to determine if regional cerebral oxygenation (rScO2) can be used as an indicator of tissue perfusion in ICU patients with TBI [1, 2], and to determine the prognostic value of cerebral oxygenation rScO2 in survival prediction.

**Methods:** Patients were enrolled retrospectively from January 2012 through July 2018 in the ICU of Derince Kocaeli Training Hospital. 250 patients with trauma patients and traumatic braine injury patients who were admitted to the ICU from the emergency room were included in the study. The sedation levels of the patients were followed up with BIS. The rScO2, BIS was taken as well as blood lactate level, mean arterial blood pressure and cardiac output at baseline time, 6, 12, 24, 48 and 72 hours.

**Results:** No significant difference was also detected between the value of rScO2 in all patients . It was Average ScO2 (right) 60.54 ±4.8 and Average rScO2 (left) 55.63± 5.4.

**Conclusions:** Cerebral regional oxygen saturation might be helpful as one of the perfusion parameters in patients with TBI but it could have no prognostic value in mortality prediction. However, further studies with larger sample size are still needed to validate these results.

**References**

1. Dunham CMet al. J Trauma. 52:40-46, 2002.

2. Balci C, et al. J Int Med Res 46:1130-1137, 2018.

### P237 Traumatic brain injury in elderly: impact of frailty on outcome

#### L Zacchetti^1^, S Aresi^1^, R Zangari^2^, G Cavalleri^1^, L Fagnani^1^, L Longhi^1^, P Gritti^1^, F Ferri^1^, L Lorini^1^

##### ^1^Ospedale Papa Giovanni XXIII, Dipartimento di Emergenza Urgenza e Area Critica, Bergamo, Italy; ^2^FROM, Fondazione per la Ricerca Ospedale di Bergamo, Bergamo, Italy

**Introduction:** TBI in elderly is an increasingly cause of admission in ICU. Data regarding management and prognosis of these patients are lacking. Validated prognostic models refer to younger patients and do not adequately consider the influence of pre-injury functional status, which often compromises with aging. Frailty has been defined as a state age-related of increased vulnerability and decline in autonomy of daily life activity. Aim of the study is to evaluate the impact of frailty on outcome in TBI elderly patients.

**Methods:** moderate and severe TBI patients >65years, admitted in NeuroICU from January 2017 to May 2018, were prospectively enrolled. Data of age, comorbidity, Glasgow Coma Scale (GCS), pupils’ reactivity, CT scan characteristics, neurosurgical intervention and GOSE (Extended Glasgow Outcome Scale) at 6-months were collected. Frailty status was measured by Clinical Frailty Scale (CFS) [1] and patients were divided as frail (CFS>4) and not frail (CFS<4). Bad outcome was defined as GOSE<4.

**Results:** 22(37%) of the 60 studied patients were frail. Frailty was not related to age. Frail patients had more comorbidities and worse pupils’ reactivity at admission (Table 1). Other variables did not differ between groups. In univariate analysis neurological diseases, GCS, tSAH (traumatic subarachnoid haemorrhage), compressed/absent basal cisterns, non-reactive pupils and CFS were significantly associated to bad outcome. In multivariate analysis only GCS and CFS remained associated to bad outcome (Table 2).

**Conclusions:** pre-injury frailty is strongly associated to outcome in TBI elderly patients.

**Reference**

1. Rockwood et al. CMAJ 173:489-95, 2005

**Table 1 (abstract P237).** Characteristics at admission

	FRAIL (CFS >4) (n=22)	NOT FRAIL (CFS <4) (n=37)	p value
Age, mean (SD)	76 (6)	75 (6)	-
Neurodegenerative disease, n(%)	11(50)	6(16)	0.008
GCS, median(IQR)	7 (5-10)	8(7-12)	-
Pupils non reactive, n(%)	13(59)	10(27)	0.03
tSAH n(%)	17(77)	25(68)	-
Basal cisterns compressed/absent, n(%)	15 (68)	21(57)	-
Shift, median(IQR)	7(3-12)	4(0-9)	-

Fisher and Mann-Whitney tests are used. Only significant p value (<0.05) are reported

**Table 2 (abstract P237).** Multivariate analysis

	OR(95% CI)	p value
Neurological diseases	1.89(0.26-13.91)	-
GCS <8	6.84(1.78-26.25)	0.005
tSAH	2.43(0.52-11.25)	-
Compressed/absent basal cisterns	0.90(0.11-7.17)	-
Non-reactive pupils	0.37(0.37-3.62)	-
CFS >4	9.98(1.81-54.98)	0.008

Only significant p value (<0.05) are reported

### P238 Efficiency of nerve block anesthesia of the scalp with low-volume local anesthetic as a component of anesthetic management of craniotomies

#### D Markevich^1^, A Marochkov^2^, V Sedin^1^, Y Zhloba^1^, N Raikova^1^, A Borozna^1^

##### ^1^Mogilev city emergency hospital, department of anesthesiology and intensive care, Mogilev, Belarus; ^2^Mogilev Regional Hospital, Department of Anesthesiology and Intensive Care, Mogilev, Belarus

**Introduction:** Efficacy evaluation of the scalp nerve blocks with low-volume ropivacaine as a component of combined anesthesia for perioperative analgesia in patients with neurosurgical interventions

**Methods:** A randomized prospective study was conducted. The efficiency of 275 blockages of the peripheral nerves of the scalp in 54 patients with neurosurgical interventions on the head was evaluated. The age of the patients was 48.4±15.6 years. Patients were operated on for intracranial traumatic (39 cases) and non-traumatic hematomas (5), brain tumors (4) and the need for plastic of postoperative skull defects (6). General endotracheal total intravenous anesthesia with fentanyl, propofol, rocuronium, or tracrium was used. After tracheal intubation, 4-12 nerves were blocked (e.g., supraorbital, supratrochlear, zygomaticotemporal, auriculotemporal, great auricular, greater and lesser occipital nerves), depending on the surgical site. 0.75–1.0% ropivacaine was used. For blockade of one nerve used 0.5-2.0 ml of local anesthetic. Fentanyl was applied on section of a periosteum, dura matter and at inefficiency of blockade of nerves. Anesthesiology monitoring included HR, ECG, SpO2, NIB, respiratory parameters, EEG (CSI), body temperature, blood glucose and lactate levels. In 2 and 10-12 hours post-surgery, the intensity of pain was ranked by alert patients using VAS.

**Results:** the volume of local anesthetic for blockade in one patient was 7.7± 1.7 ml. In 5 (9.3%) from 54 patients, an additional fentanyl injection was required to skin incision due to an increase in blood pressure and heart rate by 25% of the baseline values, and an increase in CSI until 80 un. Patients available to productive contact in 2 hours post-surgery ranked the pain by VAS at 1 (0;2) point, and in 10-12 hours post-surgery ranked it at 2 (1;3) p.

**Conclusions:** At patients with craniotomies scalpe-block with low-volumes of a ropivacaine showed high efficiency (90.7%).

### P239 Comprehension of oxidative stress in ICU patients

#### D Markopoulou^1^, A Karagianni^2^, K Venetsanou^3^, E Papadaki^1^, K Vlachos^2^, I Alamanos^1^

##### ^1^KAT General Hospital, B ICU, Kifisia, Greece; ^2^KAT General Hospital, Neurosurgery, Kifisia, Greece; ^3^KAT General Hospital, Research Unit B ICU, Kifisia, Greece

**Introduction:** The oxidative-antioxidative balance (REDOX is critical in Intensive Care Unit (ICU) patients Oxidative stress disease development strongly depends on major REDOX molecules Malondialdehyde (MDA) and Superoxide Dismutase (SOD) which are activated under inflammatory conditions. Total antioxidant capacity TAC is the sum of all antioxidant circulating molecule. The aim of this study is to investigate early REDOX markers in ICU patients.

**Methods:** Forty two patients hospitalized in ICU enrolled in the study, 28 neurosurgical patients with CNST and 14 with traumatic brain injury (TBI). A single peripheral blood sample was collected from each patient before surgical intervention or upon admission to ICU. Major REDOX molecules, Malondialdehyde (MDA) and Superoxide Dismutase (SOD), as well as Total Antioxidant Capacity (TAC) were determined by spectrophotometry.

**Results:** TAC levels were lower in CNST patients when compared with the TBI group (p<0.05). On the opposite, MDA was found to be significantly increased in the neurosurgical group when compared with that of injured subjects (p<0.05). Although no significant differences found in SOD levels, distinctive alterations were revealed by studying SOD/MDA ratio.

**Conclusions:** REDOX markers, TAC and MDA were associated with patient diagnosis and were reversed between TBI and CNST individuals. Redox balance could be evaluated for ICU patient’s management.

### P240 The microbiology of central nervous system infections of neurosurgical patients treated in the intensive care unit

#### G Bouboulis^1^, E Drosos^2^, M Nepka^3^, I Karaminas^1^, C Vrettou^4^

##### ^1^Evangelismos Hospital, First Critical Care Department, School of Medicine, University of Athens, Athens, Greece; ^2^Evangelismos Hospital, First Department of Neurosurgery, School of Medicine, University of Athens, Athens, Greece; ^3^Evangelismos Hospital, Microbiology Department, Athens, Greece; ^4^Evangelismos Hospital, First Department of Intensive Care, Athens, Greece

**Introduction:** Our purpose is to identify the species and antibiotic resistance of the microorganisms that cause hospital acquired central nervous system (CNS) infections in neurosurgical patients treated in the intensive care unit. We also examine how often the CNS pathogens were previously isolated in samples other than the cerebrospinal fluid (CSF).

**Methods:** This is a retrospective epidemiological study. From the electronic medical files we retrieved the demographic clinical and microbiology data of the neurosurgical patients treated in our department from 1/1/2013 to 31/12/2017. The hospital’s ethics committee approved the study protocol.

**Results:** We found 42 positive CSF cultures from 31 neurosurgical patients with suspected CNS infection. The reasons for ICU admission and the presence of foreign bodies in the CNS are shown in Table 1. Figure 1 shows the isolated microorganisms. Resistant to Carbapenems were 77% of the gram negative pathogens and 44% of the gram positives. Resistant to Colistin were 30% of the gram negatives and there was one case of Vancomycine resistant enterococcus. In 50% of the cases the CSF isolates were previously cultured in the same patient, either in brochial secretions (37%), or in blood cultures (13%).

**Conclusions:** Our results are in agreement with recent published literature pointing that Acinetobacter and Klebsiella sps. are becoming more frequent pathogens of hospital acquired CNS infections than coagulase negative Staphylococci [1, 2]. The increasing emergence of Carbapenem resistance among these gram negatives complicates the empirical and definitive antibiotic treatment. The importance of previous infection and/or colonization with resistant pathogens in neurosurgical patients warrants further study.

**References**

1. O´Horo JC, et al. Neurocrit Care. 2017;27:458-467.

2. Tunkel AR et al. Clin Infect Dis 2017;00:1–32

**Table 1 (abstract P240).** Admission diagnosis and CNS foreign bodies for the study population

Admission Diagnosis	Number of patients	CNS foreign body	Number of patients
Intraparenchymal haemorrhage	7	Ventriculostomy catheter	14
Subarachnoid haemorrhage	7	Lumbar drain	5
Brain neoplasm	9	Ventriculoperitoneal shunt	3
Traumatic brain injury	7	No foreign body	9
Hydrocephalus of other cause	1		

**Fig. 1 (abstract P240).**
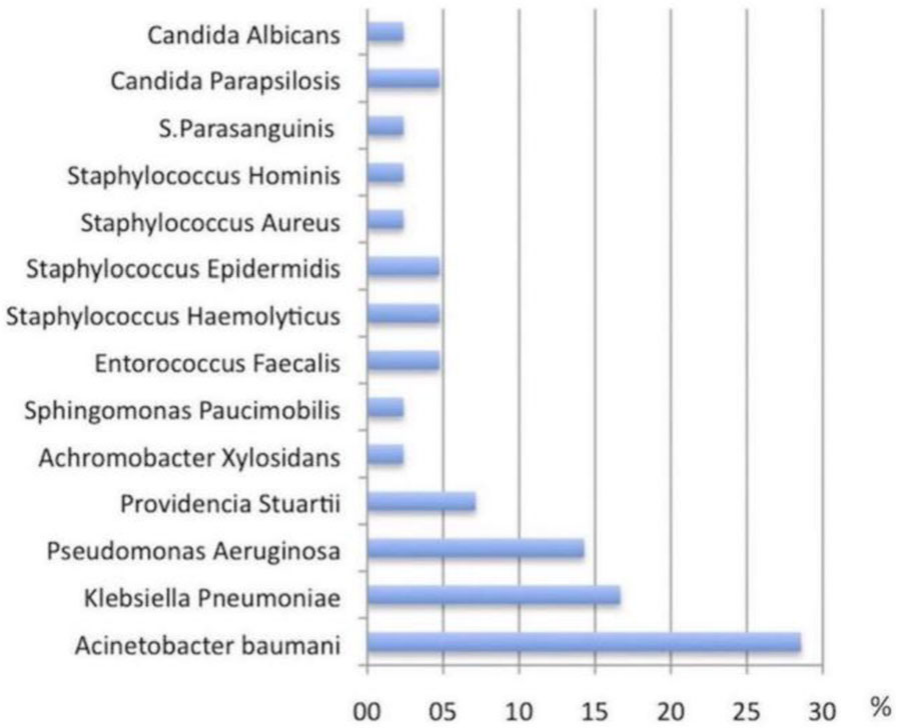
Microorganisms cultured from the cerebrospinal fluid in the study population

### P241 Association of electrolytic disturbances with increased mortality in the intensive care unit

#### J Gomez, MRRamos, A Tognon, R Gradaschi

##### Hospital São Vicente de Paulo, CTI Central, Passo Fundo - Rs, Brazil

**Introduction:** To assess the prevalence of electrolyte disorders at Intensive Care Unit (ICU) admission and its relation with mortality as an independent risk factor [1].

**Methods:** We performed a retrospective observational study including all patients >12 years-old admitted for more than 48 hours at the ICU of Hospital São Vicente de Paulo from January to August 2017. Data was entered using Microsoft Excel and the statistical analysis was performed using IBM SPSS Statistics version 22.0 for Windows. Mortality risk factors were identified using binary logistic regression models. Laboratory variables that significantly improved model’s predictive capacity compared to the model that considered only chronic kidney disease history and diagnosis of acute renal failure at admission were kept in the final model. Predictive capacity change was tested using likelihood test and was considered significant when P-value was < 0.15.

**Results:** 419 patients were admitted to the ICU, 405 were included in the study population and 14 (3.3%) were excluded due to missing data. The mean age was 53.7 ± 19.3 years and 237 (58.2%) of them male, chronic renal disease 23 (5.7%) and acute renal failure 63 (15.6%). Table 1 describes laboratory baseline characteristics of the study population. Regarding the outcome of ICU patients: 153 (37.8%) died, 197 (48.6%) were transferred to hospital ward or 53 (13.1%) to the center of intensive nursing care; 2 (0.5%) went to the surgical recovery room. Acute renal failure, hypernatremia and hyperphosphatemia were independent predictors of mortality as described in Table 2.

**Conclusions:** Hypernatremia and hyperphosphatemia were independent predictors of mortality in critically ill patients.

**Reference**

Bucley MS et al. Crit Care Med 2010; 38[Suppl.]:S253–S264

**Table 1 (abstract P241).** Serum electrolytes on ICU admission

	Na	K	Cl	P
Normal	141.8 ± 6.9	3.8 ± 0.8	112.3 ± 9.2	4.4 ± 2.0
Hypo	48 (11.9%)	126 (31.1%)	3 (0.7%)	55 (13.6%)
Hyper	98 (24.2%)	15 (3.7%)	247 (61.0%)	147 (36.3%)

**Table 2 (abstract P241).** Mortality predictors at ICU (n = 405)

AKI	HypoNa	HyperNa	HypoP	HyperP
3.11 (1.71 – 5.66)	1.90 (0.98 – 3.67)	2.56 (1.55 – 4.23)	0.52 (0.24 – 1.10)	1.82 (1.13 – 2.92)
<0.001	0.056	< 0.001	0.085	0.014

### P242 Relation between hydroelectrolytic disturbances and residence time in neurocritical patients

#### P Travassos, R Vale, W Geres, V Veiga, S Rojas

##### Hospital BP - A Beneficência Portuguesa De São Paulo, Neurocritical Care Unit, Sao Paulo, Brazil

**Introduction:** The objective of this study is to evaluate the relationship between electrolytic disturbances and length of stay in intensive care units in neurocritical patients.

**Methods:** All hospitalized patients were evaluated in a 3-month period in a large hospital neurological intensive care unit, considering sodium, potassium, magnesium and calcium analysis. During the period, 307 patients were admitted, of which 170 were female (55.4%), with a mean age of 59.85 years, 59.6% of which were surgical and the mean length of stay was 8.05 days. Statistical analysis was performed, using Cox regression, with time-dependent covariables, being considered a significance of 0.05.

**Results:** In the analyzed sample, in relation to sodium variations, RR was found to be 0.63, with p = 0.009 for hyponatremia and RR of 0.57, with p = 0.083. In the evaluation of magnesium, a RR of 0.54 (p = 0.06) was found for hypomagnesemia and RR of 1.51 (p = 0.415) for hypermagnesemia. In the potassium variations, RR of 0.26 (p = 0.001) was observed for hypokalemia and RR of 1.50 (p = 0.33) in hyperkalemia. In hypocalcemia, OR 0.85 was observed with p = 0.44 and for hypercalcemia, OR 0.611 (with p = 0.206).

**Conclusions:** In the analyzed sample, hyponatremia and hypokalemia were the electrolyte changes that correlated with increased residence time in ICU

### P243 Strong ion difference assessment: point-of-care or central laboratory?

#### T Langer^1^, S Brusatori^2^, P Brambilla^2^, C Ferraris Fusarini^2^, R Maiavacca^2^, G Giudici^1^, A Zanella^1^, G Grasselli^2^, F Ceriotti^2^, A Pesenti^1^

##### ^1^Università degli Studi di Milano, Critical Care and Emergency, Milan, Italy; ^2^Fondazione IRCCS Ca´ Granda Ospedale Maggiore Policlinico, Anesthesia, Critical Care and Emergency, Milan, Italy

**Introduction:** The Strong Ion Difference (SID) is essential for the assessment of acid-base equilibrium, thus requiring an accurate measurement of plasma electrolytes. Currently there is no gold standard for electrolyte measurements and SID computation. Differences in electrolyte values obtained with point-of-care (PoC) and central laboratory (Lab) analyzers have been reported [1, 2]. In previous studies [3, 4] we have shown that changes in PCO2 induce electrolyte shifts from red blood cells to plasma (and vice versa), yielding variations in SID. Aim of the present in-vitro study was to induce SID changes through acute changes in PCO2 and compare values of electrolytes and SID obtained with PoC and Lab techniques.

**Methods:** Blood samples from 10 healthy volunteers were tonometered (Equilibrator, RNA Medical) with 3 gas mixtures at fractions of CO2 (FCO2) of 2, 12, and 20%. Electrolytes were measured quasi-simultaneously with a PoC analyzer (ABL800 FLEX, Radiometer) and a routine Lab method (COBAS 8000 ISE, Roche). For both techniques a simplified SID was computed as sodium + potassium – chloride.

**Results:** Bland-Altman analysis of SID calculated with PoC and Lab showed a proportional bias (slope = 0.64, r2 = 0.55, p <0.0001), indicating a variable agreement between methods according to the average SID value (Fig.1). SID values measured with PoC and Lab at different FCO_2_ differed significantly (p<0.001, Fig.2). A similar discrepancy was observed for chloride (p <0.001, Fig.2), while sodium (p=0.439) and potassium (p=0.086) were similar.

**Conclusions:** SID measured with PoC and Lab differed significantly, mainly due to a variable discrepancy in chloride. Our findings suggest that our POC analyzer is superior to the Lab in measuring electrolytes and thus compute SID.

**References**

1. Morimatsu H et al. Anesthesiology 98:1077-1084, 2003;

2. Jain A et al. Int J Emerg Med 2:117-120, 2009;

3. Langer T et al. Crit Care 22(suppl 1):82, 2018;

4. Langer T et al. J Crit Care 30:2-6, 2015.

**Fig. 1 (abstract P243).**
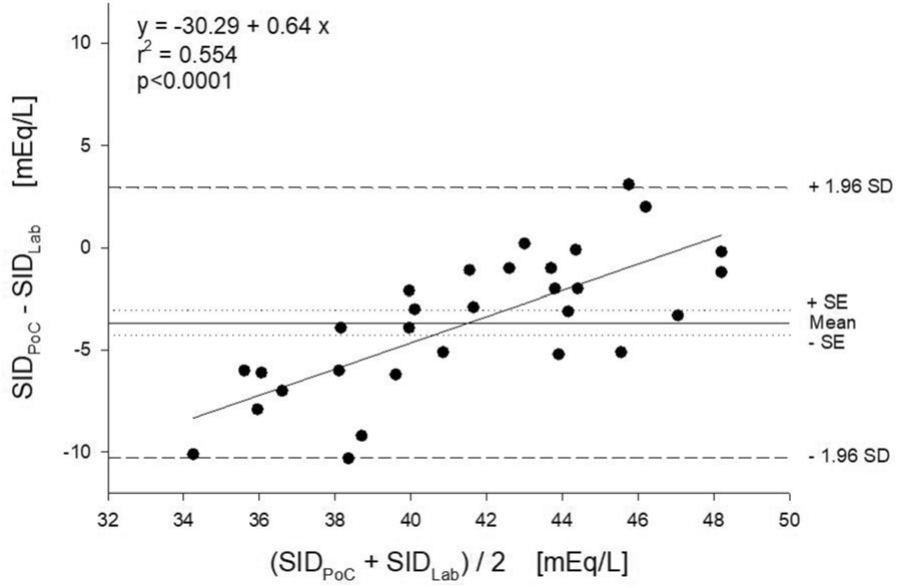
Bland-Altman analysis of simplified Strong Ion Difference (SID) calculated with point-of-care (PoC) and central laboratory (Lab) techniques. X-axis represents the mean of the two measurements, while Y-axis represents their difference. Bias is represented as the horizontal solid line (-3.66 mEq/L); standard errors of the bias are represented as horizontal dotted lines; limits of agreement (±1.96 SD) are represented as horizontal dashed lines (-10.26 and 2.94 mEq/L)

**Fig. 2 (abstract P243).**
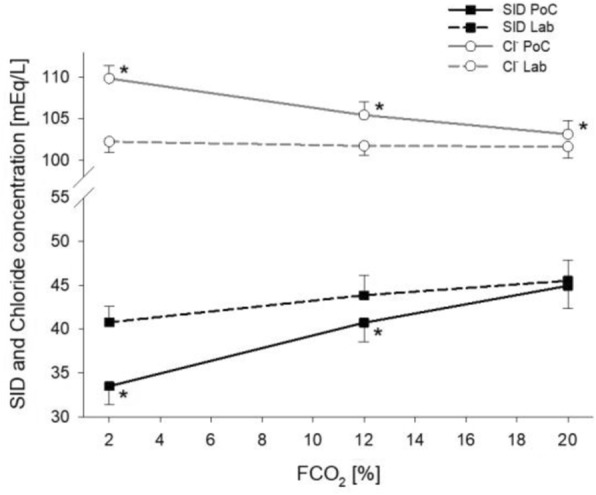
Variations in simplified SID and chloride concentration at incremental fractions of CO2 (FCO_2_) in gas mixture used for tonometry. Point-of-care (PoC) and central laboratory (Lab) data were compared via Two Way Repeated Measures ANOVA. * = p<0.05 as compared to Lab

### P244 Comparison of two insulin regimens for the treatment of hyperkalemia in critically ill patients with renal insufficiency

#### J DeCleene, T Lam, J Jancik

##### Hennepin County Medical Center, Pharmacy, Minneapolis, United States

**Introduction:** This study evaluated the safety of half dose insulin (HDI) versus standard dose insulin (SDI) for the treatment of hyperkalemia in a medical intensive care unit (MICU) population with renal insufficiency. Recent emergency medicine data demonstrated a lower incidence of hypoglycemia in patients with renal insufficiency when HDI was used for the treatment of hyperkalemia [1]. There is limited data describing the safety of HDI in a MICU population with renal insufficiency.

**Methods:** This was a retrospective, chart review of patients admitted to the MICU with a diagnosis of AKI and/or CKD stage 3-5 with a serum potassium ≥ 5.8 mEq/L from January 2013 to September 2018. SDI is defined as 10 units of regular IV insulin and HDI as 5 units. The primary outcome was the incidence of hypoglycemia within 12 hours of insulin administration. Secondary outcomes included severe hypoglycemia and change of serum potassium after insulin administration.

**Results:** A total of 265 patients were screened and 98 were included for analysis. The incidence of hypoglycemia occurred in 6/45 patients (13.3%) and 6/53 patients (11.3%) who received SDI and HDI, respectively. One patient in the SDI group and two patients in the HDI group developed severe hypoglycemia. The mean decrease in serum potassium after insulin administration was 1.0 mEq/L in both groups. Patients in the HDI group who were re-dosed with 5 units of regular insulin did not have any hypoglycemic events.

**Conclusions:** In a MICU population with renal insufficiency, SDI and HDI regimens appear safe and effective for the treatment of hyperkalemia.

**Reference**

Mcnicholas BA, et al. Kidney Int Rep. 3:328-336, 2018.

### P245 Hypernatremia in critically ill patients

#### EH Mestrom^1^, AJ Bindels^1^, JG Van der Hoeven^2^

##### ^1^Catharina Hospital, Intensive Care Unit, Eindhoven, Netherlands; ^2^Radboud University Medical Center, Intensive Care Unit, Nijmegen, Netherlands

**Introduction:** This study aims to provide new insight into predictive variables in the development of hypernatremia in critically ill patients. Current literature lacks large scale retrospective studies or studies including more variables which are considered relevant in a patient with hypernatremia [1].

**Methods:** A retrospective case control analysis was performed using prospectively collected data in the Intensive Care Unit of the Catharina Hospital in Eindhoven, Netherlands. The dataset includes demographic details, clinical data such as fluid balances and laboratory parameters, medication prescriptions and ICU and hospital outcomes. Primary outcome was the development of mild (>145 mmol/L) or severe (>149 mmol/L) hypernatremia. Case-control matching was performed for age, length of stay and APACHE IV scores.

**Results:** A total number of 5836 ICU records were included for analysis. 1159 (19.9%) patients developed hypernatremia during their ICU stay, and 543 of these patients (9.3%) developed severe hypernatremia (Table 1). After case control matching, 910 matched patients were included in the final analysis. The multivariate analysis found no strong predictors for the development of mild or severe hypernatremia (Table 2). After case control matching, multivariate analysis for mortality in case of development of hypernatremia above 145mmol/L or 149mmol/L resulted in OR 2.84 (CI 1.51-5.33) and OR 2.2 (p 0.010, 95% CI 1.21-3.91) respectively.

**Conclusions:** Despite including more patients and more relevant variables than before, this study was not able to identify strong predictors for the development of hypernatremia. Nevertheless, the results showed both mild and severe hypernatremia during ICU admission as independent predictors of mortality.

**Reference**

Waite et al. J Crit Care. 2013;28(4):405-12.

**Table 1 (abstract P245).** Baseline characteristics

	Normonatremia	Hypernatremia >145mmol/L	Mild hypernatremia 146-149mmol/L	Severe hypernatremia >149mmol/L
Number of cases (% total)	4677 (80.1)	1159 (19.9)	616 (10.6)	543 (9.3)
Median age in years (IQR)	68 (17)	68 (16)	68 (17)	68 (16)
Median length of stay ICU in days (IQR)	2 (2)	7 (10)	5 (6)	10 (13)
Median day of highest sodium (IQR)	2 (1)	4 (5)	3 (3)	6 (5)
Mortality ICU (%)	182 (3,9)	229 (19,8)	82 (13,3)	147 (27,1)
Mortality post ICU (%)	110 (2,4)	66 (5,7)	27 (4,4)	39 (7,2)
Median APACHE IV (IQR)	42 (21)	63 (32)	57 (29)	70 (33)

**Table 2 (abstract P245).** Results of multivariate analysis after case control matching

	OR (95% CI)	p value	AUC
Mild hypernatremia			0.65
Lactate	1.1 (1.05-1.18)	<0.01	
Fever	2.0 (1.20-3.47)	<0.01	
Chloride	1.1 (1.01-1.10)	0.03	
Severe hypernatremia			0.70
Chloride	1.1 (1.09-1.20)	<0.01	

### P246 Hypernatremia in septic patients: epidemiology and prognostic impact

#### M Shirazy^1^, K Bousselmi^1^, D Abduljabar^2^

##### ^1^King Hamad University Hospital, Critical Care Department, Muharraq, Bahrain; ^2^King Hamad University Hospital, Internal Medicine Department, Muharraq, Bahrain

**Introduction:** Sepsis and septic shock are common causes of admission in the Intensive Care Unit with a high mortality rate [1,2]. Hence, electrolyte disturbances are common in this group of patients. Acute hypernatremia is one of the multiple features of homeostasis disturbances and available data in the literature suggest that its incidence can reach 47% [3,4].

**Methods:** A retrospective observational study from August 2017 to November 2018 on the patients with a primary diagnosis of sepsis admitted to the ICU in King Hamad University Hospital within 48 h after the onset of the disease. The database contained age, sex, diagnosis, initial APACHE II, initial and daily SOFA scores, Na levels, outcome, and ICU and hospital length of stay. All statistical tests were two-tailed, and the significance level was set at P<0.05. Data were analyzed with SPSS.

**Results:** Among 159 patient, 89 patients fulfilled the inclusion criteria. 32 of the included patients (35.9%) developed hypernatremia during the study period. Mortality rate was higher among the hypernatremic patients 22 (68.75%) compared to the eunatremic patients 11 (32.35%) (Fig 1, 2). The main source of sepsis was pneumonia with 37 affected patients (41.57%).

**Conclusions:** Hypernatremia is significantly associated with higher mortality in septic patients.

**References**

1. Levy MM et al. Intensive Care Med 2018;44:925-928.

2. Andre SF et al. Applied Soft Computing 2016 (42); 194-203.

3. Ni HB et al. Am J Med Sci 2016; 351; 601-605.

4. Bataille S et al. BMC Nephrol 2014; 15: 2-9

**Fig. 1 (abstract P246).**
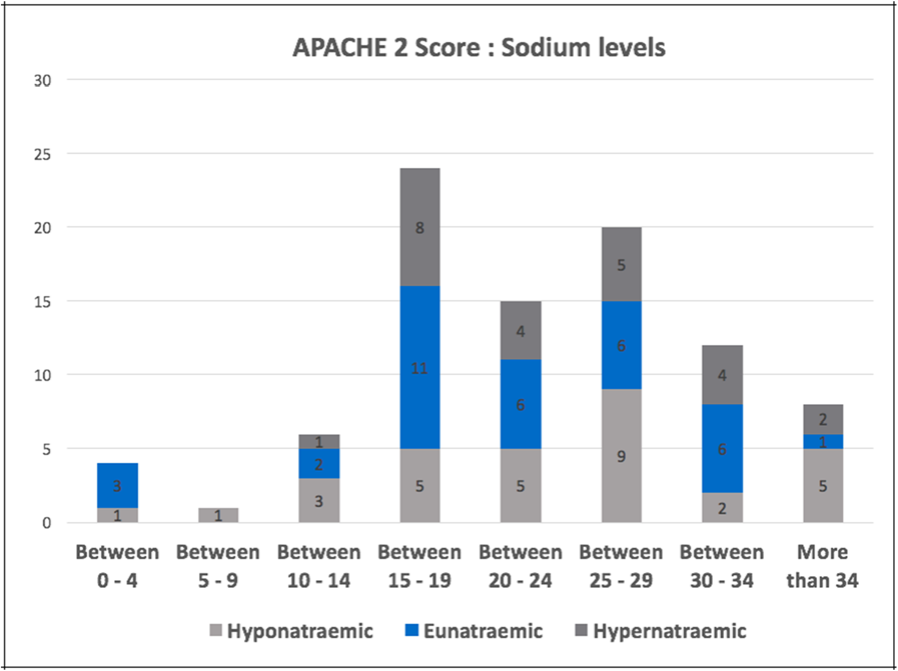
The risk of hypernatremic patient´s mortality associated with specific APACHE II scores

**Fig. 2 (abstract P246).**
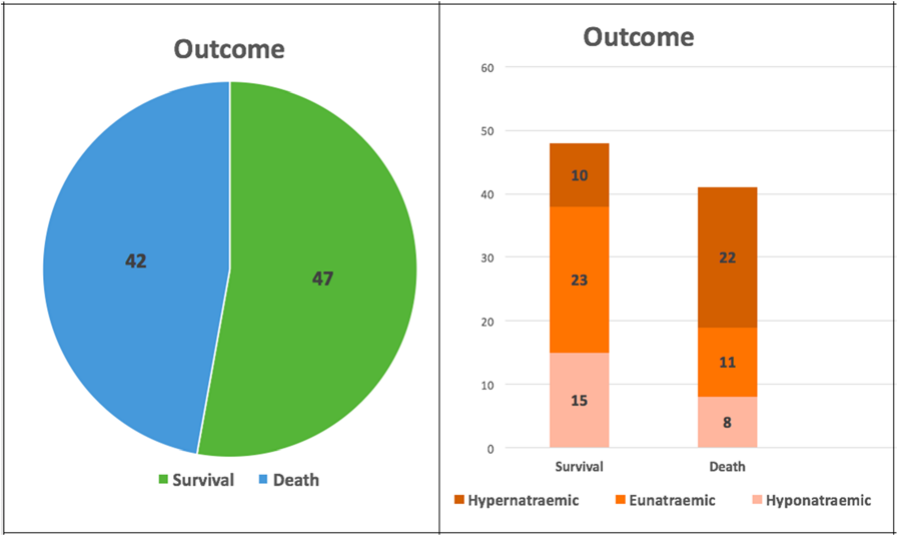
The outcome versus the sodium levels

### P247 Calcium disorders in medical intensive care: a Moroccan experience, epidemiologic data and prognosis

#### H Ezzouine, A Karchi, A Raja, K Mediouni

##### Faculty of Medicine and Pharmacy; University Hassan II, Medical Intensive Care, Casablanca, Morocco

**Introduction:** Calcium disorders are frequent among intensive care patients. Our study aimed to determine the epidemiological and clinical profile of the patients who developed hypocalcemia or hypercalcemia during their hospitalization in our unit.

**Methods:** We conducted a retrospective study in the medical intensive care unit of the university teaching hospital Ibn Rushd of Casablanca-Morocco for one year.The epidemiological, clinical and therapeutic data were collected and analyzed as was the clinical evolution.

**Results:** The incidence of hypocalcemia was 24.12%,96 patients were identified, their average age was 42.15 years old and 55.21% were female.Their mean Charlson comorbidity index was 1.65, APACHE II 10.96, SAPSII 29.06 and OSF 3.94.Neurological pathology was the main reason for admission 36.46%. 83.33% developed hypocalcemia before the 5th day from admission.48.96% had mechanical ventilation, blood transfusion was necessary for 37.50% of the patients.The mortality rate was 31.25%. The incidence of hypercalcemia was 4.27%., 17 patients were identified, their mean age was 53.82 years old and 35.29% were female .The mean charlson comorbidity index was 4.41, APACHE II was at 10.05, SAPS II 30.05 and OSF 3.88. Metabolic diseases were the main reason of admission 47.06%. 100% of these patients had hypercalcemia in their admission .35.29% were intubated and ventilated, 58.82% received diuretics, 47.05% received biphosphonates.The mortality rate was 52.94%. The factors associated with mortality were, for hypocalcemic patients, APACHE II, SAPS II, early hypocalcemia, mechanical ventilation, vasoactives drugs, blood transfusion and length of hospitalization and for hypercalcemic patients the prognosis factors were APACHE II, low serum albumin and sedation.

**Conclusions:** Calcium disorders are associated with a high mortality risk. The main prognosis factors for these patients are APACHE II, mechanical ventilation, albumin rate and its timing.

### P248 Prognostic value of serum phosphorus level in patients with multiple organ dysfunction syndrome after abdominal surgery

#### SA Tachyla, AL Lipnitski, AV Marachkou

##### Mogilev Regional Hospital, Department of Anesthesiology and Intensive Care, Mogilev, Belarus

**Introduction:** The aim of this study was to determine the prognostic significance of the dynamic measurement of serum phosphorus levels in patients with multiple organ dysfunction syndrome (MODS) after abdominal surgery.

**Methods:** 33 patients in the department of anesthesiology and intensive care unit with MODS after abdominal surgery were included in a prospective cohort study (20 men and 13 women, age 55.1±16.4 years, weight 78.9±18.4 kg.). All patients were divided into two groups according to hospital outcome: survivors (group 1, n=23) and nonsurvivors (group 2, n=10). Patients in the groups did not have statistical differences by sex, body weight and height.

**Results:** Mortality was 30.3%. In the group 2 the age of patients was higher 64.3±15.1 years vs 52.1±15.8 years (p=0.037). Apache III score was significantly higher in the group 2 – 55.4±19.4 vs 38.7±21.9 (p=0.01). The frequency of use of vasoactive drugs was significantly higher in the group 2 – 80% vs 39.1% (p=0.031). There were no significant differences between the groups in length of stay in the ICU. In group 1, there was an increase of serum phosphorus level and in the group 2 – the tendency to decrease. However, statistically significant differences were obtained only on the 2nd day after surgery 1.74±0.84 mmol/l (group 1) vs 0.88±0.26 mmol/l (group 2) (p=0.01). The ROC curve was constructed to assess the predictive significance of serum phosphorus levels (Fig. 1). AUC was 0.687; 95% CI 0.574-0.799; p=0.002; sensitivity 55.2%, specificity 83.9%. The Kaplan-Meier survival analysis (Fig. 2) showed that the survival rate was significantly higher in patients with serum phosphorus content >0.81 mmol/l (Gehan-Wilcoxon test, statistics=-3.92, p<0.001).

**Conclusions:** Constantly decreased levels of serum phosphorus indicate a poor prognosis in patients with multiple organ dysfunction syndrome after abdominal surgery.

**Fig. 1 (abstract P248).**
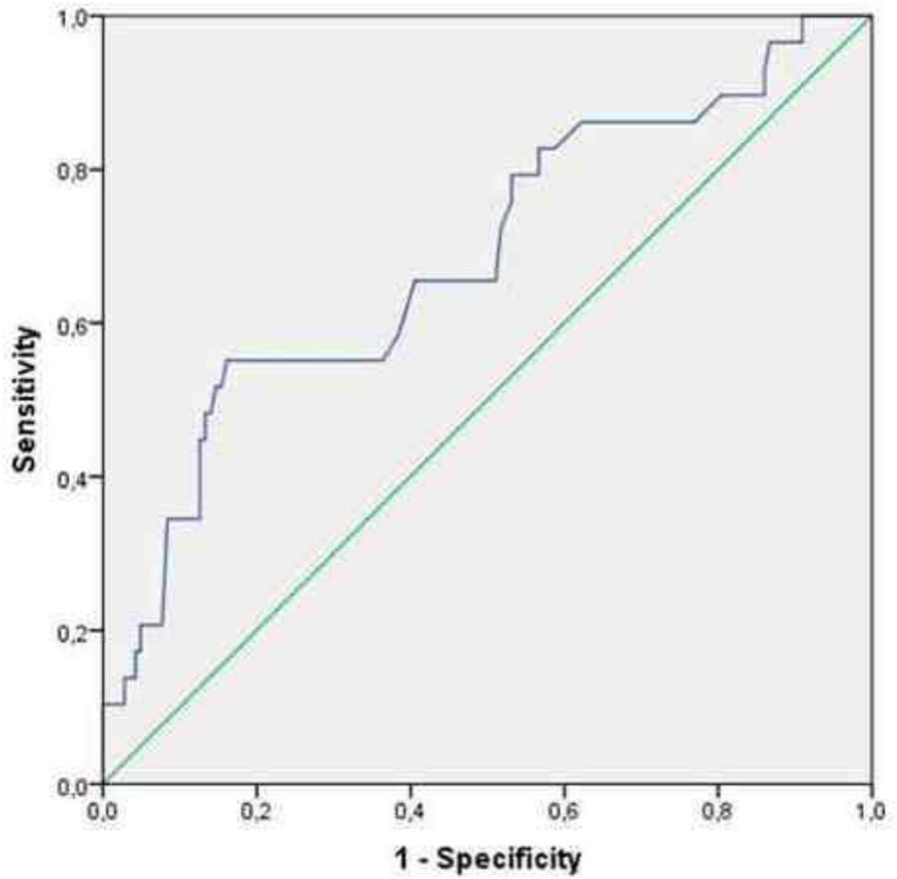
ROC-curve for serum phosphorus levels

**Fig. 2 (abstract P248).**
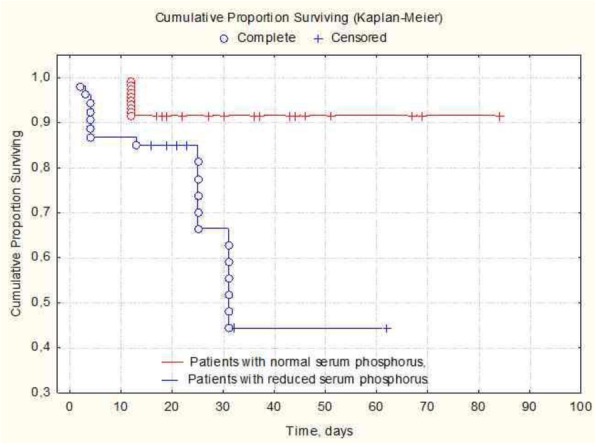
Kaplan-Meier survival analysis

### P249 Obesity is not a risk factor for extubation failure in intensive care unit

#### A De Jong^1^, HQ Quintard^2^, RC Cinotti^3^, KA Asehnoune^3^, JM Arnal^4^, CG Guitton^5^, CP Paugam-Burtz^6^, PA Abback^6^, A Mekontso-Dessap^7^, KL Lakhal^8^, SL Lasocki^9^, GP Plantefeve^10^, BC Claud^11^, JP Pottecher^12^, PC Corne^13^, CI Ichai^14^, ZH Hajjej^15^, NM Molinari^16^, GC Chanques^17^, LP Papazian^18^, EA Azoulay^19^, SJ Jaber^1^

##### ^1^PhyMed Exp University of Montpellier, Department of Anesthesiology and Intensive Care, Montpellier, France; ^2^Université Cote d’Azur, CNRS U7275, CHU de Nice, Service réanimation polyvalente et U 7275, IPMC, Nice, France; ^3^University of Nantes, Hotel-Dieu Hospital, Intensive Care & Anesthesiology Department, Nantes, France; ^4^Sainte Musse Hospital, Intensive Care Department, Toulon, France; ^5^Hopital Le Mans, Le Mans, France; ^6^Univ Paris Diderot and AP-HP, Hôpital Beaujon, Intensive Care & Anesthesiology Department, Paris, France; ^7^Hôpitaux Universitaires Henri Mondor, Assistance Publique-Hôpitaux de Paris, Service de Réanimation Médicale, Créteil, France; ^8^University of Nantes, Intensive Care & Anesthesiology Department, Nantes, France; ^9^CHU Angers, Département Anesthésie Réanimation, Angers, France; ^10^General Hospital Centre, Medical-Surgical Intensive Care Unit, Argenteuil, France; ^11^General Hospital Centre, Medical-Surgical Intensive Care Unit, Le Puy en Velay, France; ^12^Hôpitaux Universitaires de Strasbourg, Hôpital de Hautepierre, Pôle Anesthésie Réanimation Chirurgicale SAMU, Strasbourg, France; ^13^Montpellier University Hospital, Medical Intensive Care Unit, Montpellier, France; ^14^Université Cote d’Azur, CNRS U7275, CHU de Nice, Service réanimation polyvalente et U 7275, IPMC, Nice, France; ^15^Tunis military hospital, Tunis, Tunisia; ^16^IMAG, CNRS, Univ Montpellier, CHU Montpellier, Montpellier, France; ^17^PhyMed Exp University of Montpellier, Montpellier, France; ^18^APHM, URMITE UMR CNRS 7278, Hôpital Nord, Aix-Marseille Univ, Réanimation des Détresses Respiratoires et Infections Sévères, Marseille, France; ^19^Univ Paris Diderot, Saint Louis Hospital, Medical Intensive Care Unit, Paris, France

**Introduction:** The rate of extubation failure might be higher in obese patients than in non-obese patients. Effect of obesity on mortality is controversial [1,2] (obesity paradox). Several pathophysiological changes contribute to an increase of respiratory complications [1]. We sought to identify incidence of extubation failure in obese and non-obese patients.

**Methods:** The primary endpoint of this post-hoc analysis of a prospective, observational, multicenter study [3] performed in 26 intensive care units was extubation failure, defined as the need for reintubation within 48 hours following extubation. Only patients with body mass index (BMI) recorded were included.

**Results:** Between December 1, 2013 and May 1, 2015, among the 1427 patients with BMI available undergoing extubation, 301 obese patients (21%) and 1126 non-obese patients (79%) were enrolled. Extubation-failure rate was 7.6% (23/301) in obese patients, and 11.0% (124/1126) in non-obese patients (p=0.09). Delay of reintubation did not differ between obese and non-obese patients (Figure 1). Length of intubation > 8 days was significantly more frequent in obese patients (84/301, 29%) than in non-obese patients (195/1126, 18%, p<0.0001). Precautions to anticipate extubation failure were more often taken in obese patients (153/301, 51%) than in non-obese patients (426/700, 38%, p<0.0001). Spontaneous Breathing Trial (SBT) characteristics differed between obese and non-obese patients (Table 1). Physiotherapy was more often used in obese patients (173/301, 57%) than in non-obese patients (556/1126, 49%, p=0.01).

**Conclusions:** Incidence of extubation failure did not differ between obese and non-obese patients. In obese patients, clinicians anticipate more a possible extubation failure, delaying the moment of extubation, performing more physiotherapy and providing an optimal SBT.

**References**

1. De Jong A et al. Crit Care 2017

2. De Jong A et al. Crit Care Med 2018

3. Jaber S et al. Crit Care 2018

**Table 1 (abstract P249).** Characteristics of spontaneous breathing trial type according to obesity status

Characteristic	Obese patients (n = 301)	Non-obese patients (n = 1126)	p value
SBT before extubation	250 (83)	869 (77)	0.03
SBT type			
T-tube	102 (34)	407 (36)	0.47
PS-PEEP	99 (33)	344 (31)	0.44
PS-ZEEP	54 (18)	145 (13)	0.02
PEEP	6 (2)	13 (1)	0.26

**Fig. 1 (abstract P249).**
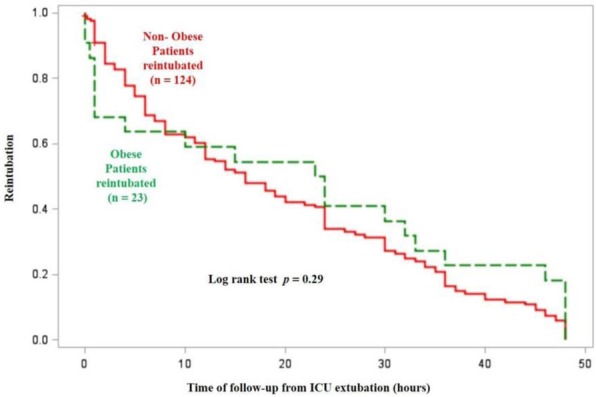
Delay of reintubation at 48 hours in obese and non-obese patients

### P250 The growth hormone axis in relation to muscle weakness in the ICU: effect of early macronutrient deficit

#### L Van Dyck, I Derese, S Vander Perre, PJ Wouters, MP Casaer, G Hermans, G Van den Berghe, I Vanhorebeek

##### KU Leuven, Laboratory of Intensive Care Medicine, Leuven, Belgium

**Introduction:** In the acute phase of critical illness, growth hormone (GH) resistance develops, reflected by increased GH and decreased insulin-like growth factor-I (IGF-I), mimicking fasting in health. The EPaNIC RCT observed fewer complications such as muscle weakness and faster recovery with accepting a macronutrient deficit in the first ICU week, as compared with early full feeding [1,2]. We characterized its impact on the GH axis in relation to the risk of acquiring muscle weakness.

**Methods:** In this EPaNIC RCT sub-analysis, for 564 matched patients per group, and all patients assessed for muscle weakness (n=600), serum GH, IGF-I, IGF binding protein 3 (IGFBP3) and IGFBP1 were measured upon ICU admission and at day 4 or the last ICU day for patients with shorter ICU stay (d4/LD). For 10 matched patients per group, GH was quantified every 10 min between 9 PM and 6 AM, and deconvolved to estimate GH secretion. Groups were compared with Wilcoxon test or repeated-measures ANOVA. Associations between changes from baseline to d4/LD and muscle weakness were assessed with logistic regression analysis, adjusted for baseline risk factors, baseline hormone concentrations and randomization.

**Results:** In the fully fed group GH, IGF-I and IGFBP3 increased, whereas IGFBP1 decreased from admission to d4/LD (all p<0.001). Accepting an early macronutrient deficit prevented the rise in GH and IGF-I and the decrease in IGFBP1 (all p<0.005) but did not affect IGFBP3, whereas basal, but not pulsatile, GH secretion was lowered (p=0.005). A stronger rise in GH and IGF-I was independently associated with a lower risk of acquiring muscle weakness (OR (95%CI) per ng/ml change 0.88 (0.81-0.96) for GH; 0.98 (0.97-0.99) for IGF-I).

**Conclusions:** Accepting an early macronutrient deficit suppressed basal GH secretion and reduced IGF-I bioavailability during critical illness, which may counteract its protection against muscle weakness.

**References**

1. Casaer MP et al. N Engl J Med 365:506-17, 2011;

2. Hermans G et al. Lancet Respir Med 1:621-9, 2013

### P251 Length of stay in intensive care unit related with incorrect choice of treatment of diabetic ketoacidosis

#### D Adukauskiene^1^, L Jazokaite^1^, R Verkauskiene^2^

##### ^1^Hospital Kaunas Clinics of Lithuanian University of Health Sciences, Intensive Care Clinic, Kaunas, Lithuania; ^2^Hospital Kaunas Clinics of Lithuanian University of Health Sciences, Endocrinology Clinic, Kaunas, Lithuania

**Introduction:** Aim of the study was to relate hypokalemia (hypoK) and hypoglycemia as diabetic ketoacidosis (DKA) treatment complications and precocious insulin interruption also use of sodium bicarbonate with length of stay (LOS) in intensive care unit (ICU).

**Methods:** Analysis of retrospective cohort study data of 100 patient (pt) treated for DKA at ICU of Hospital Kaunas Clinics of Lithuanian University of Health Sciences during 2012-2018 has been carried out. Serum kalemia, glycaemia; rate of episodes of hypoK, hypoglycaemia and precocious insulin interruption; use of sodium bicarbonate, in relation with LOS in ICU were analysed. SPSS 23.0 was used for statistic calculations. Traits evaluated as significant at p<0.05.

**Results:** At the beginning of DKA treatment hypoK (3.1±0.3 mmol/l) was recorded in 53/100 (53%) pt. Due to disregarding of blood pH (6.8 - 7.3 (7.1 ± 0.1) kalemia was falsely misinterpreted as “normo-“ or “hyperkalemia” 3.5 - 6.1 (4.8 ± 0.8mmol/l) in 49 of 63 (78%) pt, as normo– and hyperkalemia thus not treated and complicated by hypoK additionally in 20/49 (41%) pt. In hypoK LOS in ICU was 52.2 ± 31.3 vs 31.7 ± 18.2 h, p<0.05. Insulin use has caused hypoglycaemia (1.2 - 3.3 (2.5 ± 0.7 mmol/l)) in 20/100 (20%) pt, LOS in ICU 62.1 ± 40.3 vs 37.7 ± 21.4 h, p<0.05. Insulin use was interrupted in case of normo - and hypoglycaemia with still persisting ketoacidosis in 34/100 (34%) pt, LOS in ICU was found to be 56.2 ± 32.1 vs 35.5 ± 22.5 h, p<0.05. Sodium bicarbonate was given for symptomatic treatment of acidosis during the first 10 h of DKA in 21/100 (21%) pt with stable hemodynamic: HCO3 ^-^ buffer has increased (4.2 ± 3.1-7.5 ± 3.0 mmol/l), p<0.05, but ketoacidosis has still persisted, LOS in ICU was 54.8 ± 30.0 vs 39.3 ± 26.5 h, p<0.05.

**Conclusions:** HypoK (53%), hypoglycemia (21%), precocious interruption of insulin use (34%) have prolonged LOS in ICU almost twice. Symptomatic treatment of ketoacidosis with sodium bicarbonate (1/5 pt) didn’t control it and has prolonged LOS in ICU.

### P252 Glucocorticoid dimerization deficient mice display an aberrant metabolic response to endotoxic shock

#### T Merz^1^, O McCook^2^, N Denoix^3^, U Wachter^1^, J Tuckermann^4^, P Radermacher^1^, S Vettorazzi^4^, M Wepler^1^

##### ^1^Ulm University Medical Center, Institute for Anesthesiological Pathophysiology and Process Engineering, Ulm, Germany; ^2^Ulm University Medical Center, Ulm, Germany; ^3^Ulm University Medical Center, Clinic for Psychosomatic Medicine and Psychotherapy, Ulm, Germany; ^4^Ulm University, Institute for Comparative Molecular Endocrinology, Ulm, Germany

**Introduction:** Cystathionine-γ -lyase (CSE), a regulator of glucocorticoid (GC)-induced gluconeogenesis[1], correlates with endogenous glucose production in septic shock[2]. The hyperglycemic stress response to noradrenaline (NoA) is mediated by the kidney[3] and less pronounced with low CSE[4]. GC receptor (GR)-mediated gene expression is differentially regulated: the GR monomer is considered to repress inflammation, and GC side effects are attributed to the GR dimer; recent reports challenge this view[5]. GC-induced gluconeogenic gene expression is reduced in GR dimerization deficient (GRdim) mice[6]. The aim of this study is to investigate renal CSE expression and systemic metabolism in GRdim and GRwt mice in a resuscitated model of LPS-induced endotoxic shock.

**Methods:** Anesthetized GRdim (n=10) and GRwt (n=9) mice were surgically instrumented, monitored, resuscitated and challenged with LPS. NoA was administered to maintain MAP and ^13^C_6_ glucose was continuously infused. 6h after LPS, CSE expression was determined via immunohistochemistry of formalin-fixed paraffin sections (n=8 p.gr.).

**Results:** GRdim required 2.5-fold more NoA than GRwt and had 1.5-fold higher glucose and 1.9-fold higher lactate 6h after LPS. This was concomitant with elevated endogenous glucose production (2-fold), 10% lower glucose oxidation and 1.8-fold higher renal CSE expression in GRdim.

**Conclusions:** Increased CSE expression together with higher glucose production (confirming[2,4]) and glucose levels in GRdim mice suggest an association that may link CSE to GC signaling. The higher NoA administration in GRdim mice could contribute to these effects. Funding: CRC1149

**References**

1: Untereiner AA et al. Antioxid Redox Signal. 24:129-140,2016.

2: Merz T et al. ICMx. 5:30,2017.

3: Gerich JE et al. Diabetes Care. 24:382-91,2011.

4: Merz T et al. ICMx. 6:43,2018.

5: Vandevyver et al. Endocrinology. 154:993-1007,2013.

6: Frijters et al. BMC Genomics. 11:359,2010.

### P253 Automated blood glucose management in critically ill patients

#### J Mader^1^, M Motschnig^2^, V Schwetz^2^, K Eibel-Reisz^3^, A Reisinger^2^, B Lackner^4^, T Augustin^4^, P Eller^2^, C Mirth^3^

##### ^1^Medical University of Graz, Internal Medicine / Endocrinology and Diabetology, Graz, Austria; ^2^Medical University of Graz, Graz, Austria; ^3^Karl Landsteiner Privatuniversität (KPU), Universitätsklinikum St. Pölten, t. Pölten, Austria; ^4^Joanneum Research GmbH, HEALTH - Institute for Biomedicine and Health Sciences, Graz, Austria

**Introduction:** To achieve safe glycemic control in critically ill patients frequent blood glucose (BG) measurements and according titration of insulin infusion rates are required. Automated systems can help to reduce increased workload associated with diabetes management. This bi-centric pilot study combined for the first time an intraarterial glucose sensor with a decision support system for insulin dosing (SGCplus system) in critically ill patients with hyperglycemia.

**Methods:** Twenty-two patients (3 females, 19 males, 12 with pre-existing diabetes mellitus, age 65.7 ± 12.7 years, BMI 27.3 ± 3.5 kg/m2, creatinine level 1.4 ± 1.2 mg/dl, SAPS (Simplified Acute Physiology Score) 64.6 ± 18.3, TISS-28 (Therapeutic Intervention Scoring System) 39.2 ± 5.5 who were equipped with an arterial line and required iv insulin therapy were managed by the SGCplus system during their medical treatment at the intensive care unit.

**Results:** 1845 SGCplus-based BG determinations were performed and 0.8 ± 0.8 sensor calibrations per day were required. Sensor glucose readings correlated well with reference BG (Figure 1). Mean treatment duration was 6.0 ± 3.8 days. Time to target was 100 ± 178 min (80-150 mg/dl) and 135 ± 267 min (100-160 mg/dl). Mean blood glucose was 142 ± 32 mg/dl with seven blood glucose values <70mg/dl. Mean daily insulin dose was 62 ± 38 U and mean daily carbohydrate intake 148 ± 50 g /day (enteral nutrition) and 114 ± 50 g/day (parenteral nutrition). Acceptance of SGCplus suggestions was high (>90%).

**Conclusions:** The novel intraarterial glucose sensor demonstrated to be highly accurate. The SGCplus system can be safely applied in critically ill patients with hyperglycemia and enables good glycemic control.

**Fig. 1 (abstract P253).**
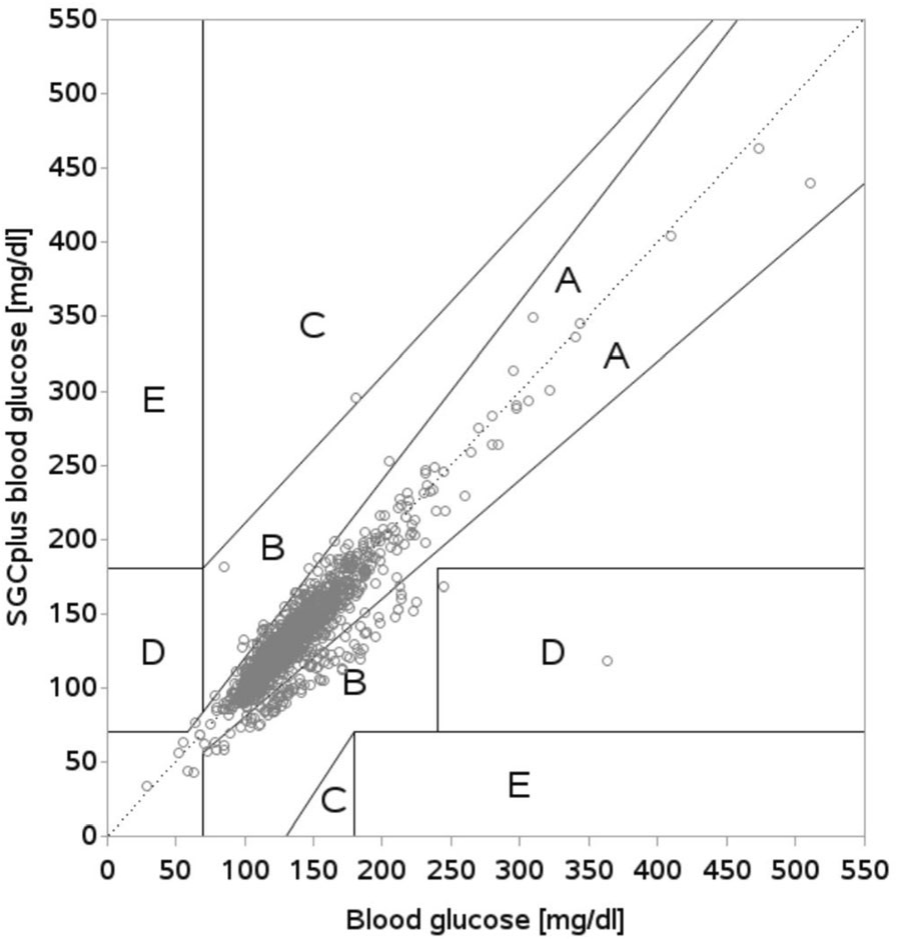
Clarke Error Grid for SGCplus Blood Glucose Sensor. 99.8% of values were located in the clinically benign zones A and B. One value was located in zone B and two values in zone D

### P254 Posthepatectomy critical care

#### N Rapti, P Vernikos, K Tsakalis, I Floros

##### General Hospital of Athens LAIKO, ICU, Athens, Greece

**Introduction:** Post hepatectomy care remains crucial for successful outcome due to the metabolic and functional changes that follow this procedure. The aim of this study was to evaluate the risk factors that could affect the outcome.

**Methods:** From 01/2017 to 10/2018, 47 patients (26 females–21 males), aged between 43 and 83 years underwent major resections (>3 segments) due to cancer (primary or metastatic). All patients had normal liver parenchyma or cirrhosis Pugh – Child’s A. Pre and post-operative liver biochemistry and coagulation markers were analyzed. We evaluated the presence of hypothermia and shock, lactic acid values, the number of transfusions and the outcome.

**Results:** At the time of ICU admission the majority of patients were hypothermic, no one received vasopressors and 8 of them remained intubated. No significant coagulation abnormalities pre- or post-hepatectomy were observed (Table 1). Twenty-eight patients did not receive any transfusion (Group A), the rest 19 patients (Group B) were transfused intraoperatively (average 2.5 RBC). Postoperatively the transfused patients had higher levels of LDH, bilirubin, serum lactate and needed more time to normalize the lactate value (Table 2). Posthepatectomy mortality was 2.1%.

**Conclusions:** Advanced age and primary or metastatic disease do not affect the morbidity and mortality. The presence of elevated conjunctive enzymes preoperatively and the need for transfusion intraoperatively seem to be associated with delayed extubation, higher lactate values and lower reduction rate for lactate.

**Table 1 (abstract P254).** Pre-operative parameters

Pre-operatively	Group A	Group B
Age (years)	64	65.5
INR	1.05	1.18
SGOT/SGPT	28.7/29.7	40.3/42.3
LDH	235.56	186.16
γ GT/ALP	61.7/87.5	189/186.2
TBil	0.5	1.8

**Table 2 (abstract P254).** Post-operative parameters

Post-operatively	Group A	Group B
Intubated in ICU	3/28	5/19
Lactate/Reduction to normal	2.74/8.92h	3.46/ 15.72h
TBil	1.43	2.72
LDH	559.2	991.7
Days in ICU	2.71	2.94
PO2/FiO2	341.8	368.6

### P255 Comparison of risk scores and shock index for hemodynamically stable patients presenting emergency department with non-variceal upper gastrointestinal bleeding

#### B Ko^1^, DH Jung^2^, WY Kim^2^, YJ Kim^2^

##### ^1^College of Medicine, Hanyang University, Department of Emergency Medicine, Seoul, South Korea; ^2^Asan Medical Center, University of Ulsan College of Medicine, Seoul, South Korea

**Introduction:** Risk assessment in upper gastrointestinal bleeding (UGIB) is recommended and various scoring systems have been developed and validated [1]. However, there is few studies that compares the performance of scores in hemodynamically stable patients in emergency department

**Methods:** Single center prospective observational study was conducted, consecutive non-variceal UGIB patients presented with normotension (systolic blood pressure ≥90 mmHg) to the emergency department were included. We compared the area under the curves (AUC) of Glasgow Blatchford score, pre-endoscopy Rockall score, AIMS65, shock index, modified shock index in regard to composite outcome (endoscopic treatment, embolization, surgery, ICU admission, rebleeding, in-hospital mortality)

**Results:** A total of 1,233 patients were included, and the composite outcome occurred in 493 patients (40%) and in-hospital mortality was 1.2%. The AUC of Glasgow Blatchford score for composite outcome was higher than those of shock index, however there was no significant difference (0.644 vs 0.593, p = 0.43) (Table 1). The AUC of modified shock index, AIMS65 and pre-endoscopy Rockall score have shown 0.58, 0.552 and 0.52, respectively. Rule in cutoff value of Glasgow Blatchford score (> 8) was associated with 81% sensitivity and 41% specificity for predicting composite outcome (Table 2).

**Conclusions:** Pre-existing risk score have shown suboptimal level of predictive ability for adverse event in normotensive patients with non-variceal UGIB. Glasgow Blatchford score (>8) might be useful to rule in patients of adverse event, however further studies with risk scores or new scores are needed given relatively low accuracy of those scores.

**Reference**

1. Blatchford O, et al. BMJ 315, 510-514 (1997).

**Table 1 (abstract P255).** Diagnostic performance of scoring systems

Scoring system	AUC (95% CI)
Composite outcome	
Glasgow Blatchford	0.644 (0.613–0.675)
Pre endoscopy Rockall score	0.52 (0.488–0.553)
AIMS65	0.552 (0.519–0.584)
Shock index	0.593 (0.561–0.626)
Modified shock index	0.58 (0.547–0.612)

**Table 2 (abstract P255).** Cutoff value of Glasgow Blatchford score, AIMS65, and pre-endoscopy Rockall score in prediction of composite outcome

Scores	Sensitivity	Specificity	PPV	NPV
Glasgow Blatchford >8	81%	41%	48%	76%
Pre-endoscopy Rockall score >1	82%	22%	41%	65%
AIMS65 >1	63%	51%	43%	64%
Shock index >0.8	53%	61%	48%	66%
Modified shock index >1.2	3%	78%	50%	64%

### P256 Impact of frailty on critical care and hospital mortality in critically-ill patients with decompensated alcoholic liver disease

#### A Pedder^1^, R Harrold^1^, A Cruikshanks^1^, A Tridente^2^, A Raithatha^1^

##### ^1^Sheffield Teaching Hospitals, Critical Care, Sheffield, United Kingdom; ^2^St Helens & Knowsley Hospitals Trust, Critical Care, St Helens, United Kingdom

**Introduction:** We aimed to assess the effect of frailty as assessed by Clinical Frailty Scale (CFS) and Karnofsky performance score (KPS) on critical care (CC) and hospital mortality in this group at a non-specialist tertiary critical care unit.

**Methods:** Patients admitted to critical care were identified from our electronic database by screening for liver disease or cirrhosis in the admission diagnoses. Those with an aetiology of liver disease other than alcoholic liver disease (ALD) were excluded. Data was collected on patient demographics, length of stay, status at discharge from critical care and hospital and CFS. KPS was also calculated where sufficient in-formation was available in the medical record. Data was analysed using logistic regression multivariate analysis with Stata 14 software.

**Results:** A total of 146 patients were identified from our database between December 2011 and Feb 2017. The median (IQR) age was 51 (43-59), and 83 (56%) were male. Mean length of CC stay was 4.9 days and hospital length of stay was 21 days. Overall critical care mortality was 40.4% and hospital mortality was 55.5%. SOFA and increasing frailty as assessed by KPS correlates with increasing critical care mortality, Odds ratio (OR) 0.8, CI 0.70-0.93 p=0.003 for SOFA and OR 1.04, CI 1.01-1.08 p=0.009 for KPS and also for hospital mortality, OR 0.81 CI 0.70-0.93 p value=0.004 for SOFA and OR 1.04CI 1.01-1.08, p=0.011 KPS respectively.

**Conclusions:** SOFA and increasing frailty, as assessed by KPS, in patients with decompensated ALD correlates with an increase in critical care and hospital mortality.

### P257 Tokyo guidelines 2018: updated Tokyo guidelines for the management of acute cholangitis/acute cholecystitis

#### T Mayumi^1^, T Takada^2^, M Yoshida^3^

##### ^1^School of Medicine, University of Occupational and Environmental Health, Department of Emergency Medicine, KitaKyushu, Japan; ^2^Teikyo University, Department of Surgery, Tokyo, Japan; ^3^Ichikawa Hospital, International University of Health and Welfare, Department of Hemodialysis and Surgery, Ichikawa, Japan

**Introduction:** We proposed Tokyo Guidelines 2007, first international practice guidelines for the management of acute cholangitis and acute cholecystitis and revised them (TG13) in 2013 from clinical feedback.

**Methods:** After then, modern clinical practice and new evidence made us re-revised the guidelines as Tokyo Guidelines 2018 (TG18) using GRADE system [1].

**Results:** TG13 diagnosis criteria and severity grading criteria for acute cholangitis and acute cholecystitis were judged from numerous validation studies as useful indicators in clinical practice and adopted as TG18 diagnostic criteria and severity grading without any modification. Provide initial treatment, such as sufficient fluid replacement, electrolyte compensation, and intravenous administration of analgesics and full-dose antimicrobial agents, as soon as a diagnosis has been made. In new flowchart for the treatment of acute cholecystitis (AC) in the TG18, Grade III AC was indicated for gallbladder drainage, but some Grade III AC can be treated by laparoscopic cholecystectomy (Lap-C) at advanced centers with specialized surgeons experienced in this procedure and for patients that satisfy certain strict criteria. We also redefine the management bundles for acute cholangitis and cholecystitis.

**Conclusions:** TG 18 provide evidence-base management of acute cholangitis and acute cholecystitis

**Reference**

1. Free full articles and mobile app of TG18 are available at: http://www.jshbps.jp/modules/en/index.php?content_id=47.

### P258 ^13^CO_2_-breath-test to estimate gastric emptying as a marker of gut functioning under critical illness.

#### J Vogt^1^, L Bernhard^2^, M Georgieff^3^, P Radermacher^1^, E Barth^3^

##### ^1^University Medical Center, Institute APV, Ulm, Germany; ^2^University Medical Center, Ulm, Germany; ^3^University Medical Center, Department of Anesthesia, Ulm, Germany

**Introduction:** ^13^C-acetate breath tests provide a non-invasive assessment of gastric emptying [1] and could, hence, be used to judge tolerance to enteral nutrition. Result values like t50 (time for 50% absorption) correlate with scintigraphic measurements. The data evaluation is based on model equations like the β -exponential function (BEX) [1]. It considers a mono-phasic breath gas response. This may not be the case during critical illness, which could reduce precision too low for a reliable personalized assessment [2].

**Methods:** We recently developed an evaluation of irregular gastric emptying patterns, which separates absorption from post-absorptive distribution and retention of tracer and from the terminal respiratory release of the oxidized tracer [3]. Using breath test data of 31 ICU patients (mean SAPS2 36 +/- 14) the precision of this approach was compared with a BEX analysis to explore how often an extended analysis is warranted and whether it improves the reliability of estimates.

**Results:** 15 patients had a release profile consisting of series of peaks with a periodicity of 60-90 min. A first dominant peak carries about 95% of the released moiety, as reported [4] for controls. For these patients the precision in t50 for the BEX approach was 70 +/- 30% of that observed for the new approach. For the other patients, the secondary peaks had a similar periodicity but were more pronounced, indicating persisting peristaltis, which has been linked to tolerance to enteral nutrition [4]. The BEX approach achieved a precision of 40 +/- 27 % relative to the new one, challenging its applicability for these patients.

**Conclusions:** Irregular release patterns for about 50 % of the patients can be handled with the new evaluation which enhances the applicability of ^13^C-breath tests.

**References**

1: Bluck LCJ et al. Physiol Meas. 27:279-289, 2006.

2: Deane A et al. Clinical Nutr. 29:682-6,2010.

3: Vogt J et al. J Breath Res. 11 (2017).

4: Moore FA et al. J Trauma 51:1075-82, 2001.

### P259 Gastrointestinal failure as a predictor of the development of infections in patients with acute pancreatitis

#### A Ramos^1^, A Dogliotti^2^, C Misto^3^, A Ugolini^4^, M Perezlindo^1^, F Acharta^5^, C Lovesio^1^

##### ^1^Grupo Oroño, Critical Care, ROSARIO, Argentina; ^2^Grupo Oroño, Statistics and Epidemiology, ROSARIO, Argentina; ^3^Grupo Oroño, Bacteriology, ROSARIO, Argentina; ^4^Grupo Oroño, Infectology, ROSARIO, Argentina; ^5^Sanatorio Parque, Critical Care, Carlos Pellegrini, Argentina

**Introduction:** Infections in acute pancreatitis are a frequent complication and lead to an increase in morbidity and mortality. Intestinal barrier dysfunction increases the risk of bacterial translocation and the consequent development of infections. The objective of this study was to determine if gastrointestinal failure is associated with an increase in infections in patients with acute pancreatitis.

**Methods:** We retrospectively analyzed the cases admitted to a 3rd level center for acute pancreatitis, from January 2013 to April 2018. The definition of gastrointestinal failure developed by the European group was used (WGAP-ESICM). The same was recorded during the first 48 hours of admission and the persistence of it on the 5th day. A logistic regression model was used to determine if the development of gastrointestinal failure was independently associated with the development of infections.

**Results:** Forty-six patients were included. Twelve (26%) of them developed infections during their evolution. In the multivariate analysis, the development of gastrointestinal failure during the first 5 days of hospitalization was independently associated with the development of infections (p=0.01, OR 32.5, 95% CI 1.99-529) (AUC ROC 0.95 95% CI 0.84-0.99).

**Conclusions:** A relation was established between the presence of gastrointestinal failure during the first 5 days of acute pancreatitis and the development of infections. However, this must be studied in a larger cohort of patients.

### P260 Patients with acute pancreatitis who have SIRS and hypoxemia at presentation have evidence of severe systemic and pancreatic inflammation

#### C Bruen^1^, J Miller^2^, C Mackey^3^, M Dunn^4^, K Stauderman^4^, S Hebbar^4^

##### ^1^HealthPartners Regions, St. Paul, United States; ^2^Henry Ford Health Systems, Detroit, United States; ^3^Riverside Methodist Hospital, Columbus, United States; ^4^CalciMedica, La Jolla, United States

**Introduction:** Clinical scoring systems used to prognosticate the severity of acute pancreatitis (AP), such as APACHE II, are cumbersome and usually require 24 hours or more after presentation to become accurate, at which time the window for early therapeutic intervention has likely passed. SIRS at presentation is sensitive but poorly specific for severe AP. We postulated that SIRS and accompanying hypoxemia would specify at presentation patients with AP who have severe inflammation and are at risk for clinically severe disease.

**Methods:** Patients with AP who had SIRS and hypoxemia at presentation were enrolled in an open-label study evaluating the safety and efficacy of CM4620-IE, a Calcium Release-Activated Calcium (CRAC) channel inhibitor (NCT03401190). Hypoxemia was defined as an estimated PaO2 <75 mm Hg calculated using a log-linear equation and the SpO2 on room air at the time of presentation. A contrast-enhanced computed tomography (CECT) was performed at presentation and a CBC with differential, D-dimer and CRP were analyzed daily. The CECT was read by a blinded central reader who assessed the degree of inflammation using the Balthazar scoring system (Table 1).

**Results:** 13 patients, seven men and six women, have been randomized in the study. The mean estimated PaO2 at presentation was 74 mm Hg. 10 patients had 2 SIRS criteria present and the other 3 patients had 3 SIRS criteria present. The median value for age was 53.5 (IQR 48-56), initial neutrophil-lymphocyte ratio (NLR) 10.6 (6.3-13.1), D-Dimer at 24 hours 3670 ng/mL (1440-4840), and CRP at 48 hours 230 mg/L (93-367). In 7 of 13 patients the Balthazar score was 4.

**Conclusions:** In patients with AP who have SIRS and hypoxemia at presentation, there was evidence of severe systemic inflammation as assessed by NLR, D-Dimer and CRP. The majority of patients also had CECT evidence of severe pancreatic inflammation. SIRS and SpO2 do not have the limitations of clinical scoring systems and identify AP patients at presentation who would benefit from early intervention.

**Table 1 (abstract P260).** Balthazar Score

Pancreatic Inflammation	Score
Normal Pancreas	0
Enlargement of Pancreas	1
Inflammatory changes in pancreas	2
Ill-defined single peripancreatic fluid collection	3
Two or more peripancreatic fluid collections	4

### P261 Circulating immunological profiles as early biomarkers in predicting persistent organ failure in patients with acute pancreatitis

#### J Liu

##### Department of Critical Care Medicine, Suzhou Municipal Hospital, The Affiliated Suzhou Hospital of Nanjing Medical University, Suzhou, China

**Introduction:** To investigate whether circulating immune profiles were able to serve as early biomarkers in predicting persistent organ failure (POF

**Methods:** Thirty-nine patients with predicted severe acute pancreatitis (pSAP) and 9 healthy control subjects were prospectively enrolled in our study. We measured the expression of monocytic human leukocyte antigen-DR (mHLA-DR), the proportions of dendritic cells (DC) and its subtypes (including myeloid dendritic cell (mDC) and plasmacytoid dendritic cell (pDC)), the different cytokine-producing CD4+ T helper (Th) cells and regular T (Treg) cells. Plasma CRP and several inflammatory mediators levels were measured by ELISA.

**Results:** Compared with healthy controls, there is a significant decrease in the expression of mHLA-DR, the frequencies of total circulating DCs and its subsets, and percentage of Th1 cells in patients with pSAP. However, we found significantly higher frequencies of Th17 cells, higher proportion of Treg cells than healthy subjects. Of interest, we observed that there was a significant decrease in the positive percentage and mean fluorescence intensity (MFI) of mHLA-DR, the proportions of total DCs and pDC, and Th1 cells in patients with POF compared with transient organ failure (TOF). Besides, there is a significantly higher frequency of Th17 cells in POF than those in TOF. Area under the receiver-operating characteristic curve analysis showed that disease severity scores had a moderate discriminative power for predicting POF in patients with pSAP. More importantly, the expression of mHLA-DR and the percentage of DCs and pDC had a significantly higher AUROC and thus, better predictive ability than disease severity in patients with pSAP.

**Conclusions:** Circulating immune profile show multiple aberrations in patients with pSAP who have developed POF. Both the expression of mHLA-DR and the percentage of total DC and pDC may be early good biomarkers for predicting risk of POF in patients with pSAP.

### P262 Early detection of pancreatic fistula after pancreaticoduodenectomy (Whipple’s procedure) with microdialysis catheters

#### G Bergmann, H Haugaa, S Pischke, E Lindholm, T Tønnessen

##### Oslo University Hospital, Division of Emergencies and Critical Care & Faculty of Medicine, University of Oslo, Oslo, Norway

**Introduction:** Pancreatic fistula (POPF) due to anastomosis insufficiency is a common (12-30%) complication after pancreaticoduodenectomy and often discovered with delay, causing severe morbidity, ICU stay and deaths. Microdialysis (MD) catheters have been shown to detect inflammation and ischemia in several postoperative conditions and organs. The aim was to investigate if MD catheter monitoring could facilitate earlier detection of POPF than current standard of care.

**Methods:** In a prospective, observational study 35 patients (44 to 88 years) were investigated. A MD catheter was fixed to the pancreaticojejunal anastomosis. Samples for analysis of glucose, lactate, pyruvate and glycerol were acquired hourly during the first 24 hours, then every 2-4 hours to discharge. POPF was defined according to the International Study Group of Pancreatic Fistula 2016 update definition.

**Results:** Patients who developed POPF (N=7) had significantly higher glycerol levels (P<0.01) in microdialysate than did patients without POPF (N=28) during the first 24 h. Thereafter, the difference diminished. A glycerol concentration >400 μmol/L during the first 12 h detected patients who later developed POPF with a sensitivity of 100 % and a specificity of 92%. Lactate and lactate to pyruvate ratio were significantly higher (P<0.05) and glucose was significantly lower (P<0.05) in patients with POPF from about 24 h. Fig. 1 shows microdialysis measurements in patients with (red lines) and without (blue lines) POPF.

**Conclusions:** A high level of glycerol in microdialysate is an early (first 12 hours) indicator of POPF. Glucose, lactate and lactate to pyruvate ratio are indicators of peritonitis caused by the leakage. Thus, MD monitoring detects POPF several days earlier than current methods and may play an important clinical tool in the future. We are currently conducting a RCT to explore if MD monitoring will improve prognosis in these patients

**Fig. 1 (abstract P262).**
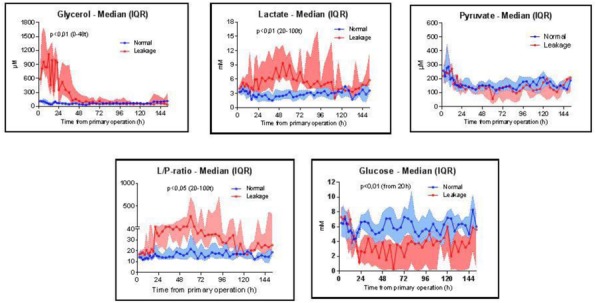
Postoperative microdialysis measurements in samples from the pancreatojejunal anastomosis catheter in patients with pancreatic fistula after pancreatoduodenectomy and in patients with no complications (reference cohort). Glycerol, lactate, pyruvate, lactate-pyruvate ratio, and glucose values during the postoperative course. Data are presented as median (line) and interquartile range (shadow)

### P263 The phenomenon of total impaired of metabolic activity of gut microbiota in critically ill septic patients

#### N Beloborodova, E Chernevskaya, A Pautova, A Sergeev

##### Federal Research and Clinical Center of Intensive Care Medicine and Rehabilitology, Laboratory of Metabolism in Critical State, Moscow, Russia

**Introduction:** During a critical condition, dramatic disturbances occur not only in the change of species diversity, but in gut microbiota metabolism as well, that might lead to nonreversible breakdowns of host homeostasis and death [1]. Metabolic activity of microbes can be assessed by the measurement of the levels of aromatic microbial metabolite (AMM) in blood serum, which are associated with the severity and mortality of ICU patients. Critically ill patients are characterized by the totally different SFS profile than in healthy people, particularly by the absence of PhPA; but dominated by p-HPhAA and p-HPhLA [2]. The purpose of our study is to assess the gut metabolic activity via AMM in sepsis.

**Methods:** In this study 40 simultaneously serum and fecal samples (SFS) were taken from ICU patients: 14 - with sepsis, 21- chronic critical ill (CCI) patients and control – 5 SFS from healthy people. After liquid-liquid extraction from serum and fecal samples, 9 phenylcarboxylic acids (AMM) were measured using GC/MS (Thermo Scientific).

**Results:** The sum of the level of 9 most relevant AMM in serum samples were higher in patients with sepsis (median - 4.7 μM) than in CCI patients (1.1 μM) and healthy people (1.3 μM). At the same time the opposite pattern was observed in the fecal samples – 2.4, 31.9 and 68.7 μM, respectively. The ratios of sums AMM gut/serum were higher in healthy people than ICU patients (Fig. 1). In group of CCI patients this ratio was higher in patients without infection compared with infection – 25.2 vs 16.1 μM, respectively.

**Conclusions:** Low ratio of sums of AMM gut/serum may reflect the phenomenon of total impaired metabolic activity of the gut microbiota in critically ill patients. Further studies may confirm the clinical significance of this indicator.

**References**

1. Chernevskaya E. et al. General Reanimatology 14: 96-119, 2018.

2. Beloborodova N.et al. Crit. Care. 22(Suppl 1): P82, 2018.

**Fig. 1 (abstract P263).**
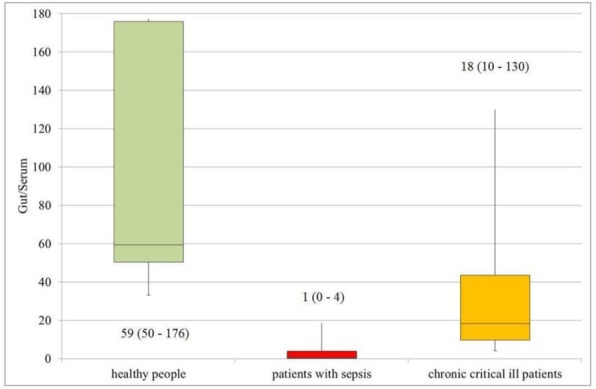
The ratio of sums of AMM gut/serum. Data presented as median and interquartile range

### P264 Evaluation of muscular and nutritional status detected by bioelectric impedance vector analysis and muscular ultrasound in critically ill patients: preliminary data

#### P Formenti^1^, M Umbrello^1^, S Coppola^1^, S Froio^1^, M Gotti^1^, L Bolgiaghi^1^, D Chiumello^2^

##### ^1^Asst Santi Paolo E Carlo-Ospedale San Paolo-Polo Universitario, Sc Anestesia E Rianimazione, Milano, Italy; ^2^Asst Santi Paolo E Carlo-Ospedale San Paolo-Polo Universitario, Dipartimento di Scienze della Salute, Università degli Studi di Milano, Milan, Italy, Milano, Italy

**Introduction:** The aim of this study is to describe the characteristic of Bioelectric Impedance Vector Analysis (BIVA) and muscular ultrasound during the first week after admission in the ICU, and their correlation with indices of metabolic support. BIVA is a commonly used approach for body composition measurements [1]. Muscular ultrasound represents a valid tool to provide qualitative and quantitative details about muscle disease [2].

**Methods:** Consecutive patients admitted to ICU and expected to require mechanical ventilation for at least 72 hours were enrolled in the study. Within the first 24 hours of ICU admission (T1), patients were evaluated with muscular ultrasonography comprehensive of diaphragm thickness (DTH) and rectus femoris cross-sectional area (CSA). At the same time, BIVA and biochemical analysis. All the same measures were repeated at day 3 (T3) and 7 (T7) (Figure 1).

**Results:** 50 patients were enrolled in the study. PA (3 [2.2-4] vs 2.6 [2.1-3.4], p<0.001), DTH (0.25 [0.2-0.32] vs 0.18 [0.14-0.21]p<0.001, CSA (2.6 [2-3.6] vs 1.6 [1.3-2.1], p<0.001) were significantly reduced after 7 days (Table 1). Dividing the patients in two groups based on prealbumine changes (T3 vs T1: increase, anabolic vs decrease, catabolic), those in which prealbumine increased had a higher reduction in muscle mass (Figure 2).

**Conclusions:** This study showed how the PA tends to be reduced in the first week of ICU stay. It is correlated with a concomitant reduction in CSA and D TH, and associated with the degree of catabolism developed in the first week of ICU stay

**References**

1. Kyle UG, et al (2004) Clin Nutr, 23(6):1430-1453

2. Connolly BA, et al. Crit Care. 2013;17:R229.

**Table 1 (abstract P264).** Oneway ANOVA for repeated measures between T2 and T3. Bonferroni´s method for multiple comparisons. The Table shows the principal results of the study

Variables	T1	T2	T3	T1 VS T3
Arm circumference	30.3 [26.5 - 34.0]	30.3 [26.0 - 34.5]	31.0 [27.8 - 36.0]	<0.001
PA	3.0 [2.2 - 4.0]	2.6 [2.1 - 3.4]	2.0 [0.8 - 3.0]	NS
D TH	0.25 [0.20 - 0.32]	0.21 [0.17 - 0.23]	0.18 [0.14 - 0.21]	<0.001
CSA	2.6 [2.0 - 3.6]	2.2 [1.8 - 2.8]	1.6 [1.3 - 2.1]	<0.001
BUN	32.5 [20.0 - 39.0]	26.0 [21.0 - 47.0]	35.0 [25.5 - 57.8]	<0.001
Azotemic Nalance	-7.4 [-26.4 ; +4.0]	-5.3 [-9.5 ; +1.9]	-1.3 [-9.7 ; +7.5]	<0.001
Prealbumine	9.2 [6.3 - 11.9]	10.1 [6.5 - 13.2]	15.2 [8.3 - 19.2]	<0.001

CSA= cross sectional area rectus femoris; D thickness= diaphragm thickness; Pa= phase angle

**Fig. 1 (abstract P264).**
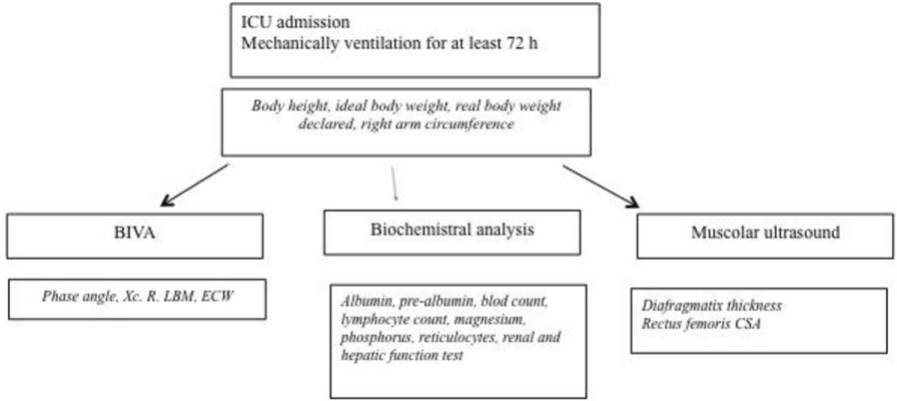
Within the first 24 hours of ICU admission (T1), patients will be evaluated with muscular ultrasonography comprehensive of diaphragm thickness and rectus femoris (medial vastus) cross-sectional area. At the same time, anthropometric measure will be collected (such as body height, ideal body weight, real body weight declared, right arm circumference) as well as BIVA measure (Xc, R, PA, lean body weight and % of extracellular body weight) and biochemical analysis (inclusive albumin, pre-albumin, blood count, lymphocyte count, magnesium, phosphorus, reticulocytes, renal and hepatic function test). The day after, the fluid balance will be calculated as well as the nitrogen balance. All the same measures will be repeated at day 3 (T3) and 7 days (T7)

**Fig. 2 (abstract P264).**
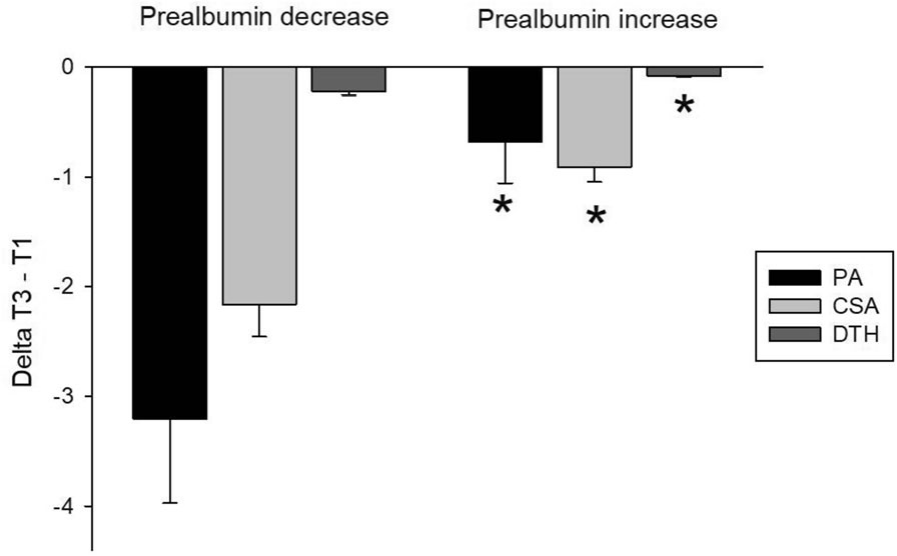
The figure shows the changes in DTH, CSA and PA between T3 and T1 in the two groups of patients divided by changes in prealbumine (decreased or increased); * <0.05

### P265 The 28-day mortality and modified NUTRIC score in Thai medical intensive care patients

#### T Petnak^1^, P Panusitthikorn^2^, A Trisataya^1^, N Thanintara-arj^3^, V Saifah^3^, V Tangsujaritvijit^1^, B Chindavijak^3^, P Dilokpattanamongkol^3^

##### ^1^Ramathibodi Hospital, Mahidol University, Department of Medicine, Bangkok, Thailand; ^2^Siriraj Hospital, Mahidol University, Bangkok, Thailand; ^3^Faculty of Pharmacy, Mahidol University, Department of Pharmacy, Bangkok, Thailand

**Introduction:** The modified Nutrition Risk in Critically ill (mNUTRIC) has been developed in order to identify critically ill patients who may receive benefit from nutrition support [1]. Several evidences showed the association between the mNUTRIC score and clinical outcomes [2, 3], however there are no data in Thai critically ill patients. The purpose of this study was to find the association between mNUTRIC score and 28-day mortality in medical intensive care unit (ICU) patients, Ramathibodi hospital.

**Methods:** We retrospectively reviewed the medical patient records from June 2016 to January 2017. A mNUTRIC score of each patient was calculated to evaluate the risk of malnutrition. Statistical analysis of the association between mNUTRIC score and 28-day mortality, length of stay in ICU and hospital were performed.

**Results:** A total of 78 critically ill patients were included in the study. The 28-day mortality was 53.06% in patients with high mNUTRIC score (5-9) and 13.79% in patients with low mNUTRIC score (0-4). Modified NUTRIC score was significantly correlated with 28 day mortality (r = 0.390, p<0.001), length of stay in ICU (r = 0.394, p<0.001) and length of stay in hospital(r = 0.414, p<0.001). In the receiver operating characteristic (ROC) curve analysis, the AUC of mNUTRIC score and 28-day mortality was 0.788 (95% confidence interval (CI), 0.686-0.889) (Fig 1). Optimal cut-off value of 6 showed sensitivity of 66.7% and specificity of 77.1% in mortality prediction (Youden’s index, 0.438). Additionally, patients who received adequate nutrition supplement within 7 days was 87.20% for calorie and 64.10% for protein. There was no association between nutrition support and 28-day mortality.

**Conclusions:** In Thai medical intensive care population, the mNUTRIC score was associated with 28-day mortality in critically ill patients.

**References**

1. Rahman A, et al. Clin Nutr 35:158-62, 2016

2. Mukhopadhyay A, et al. Clin Nutr 36:1143-8, 2017

3. Mendes, R., et al. J Crit Care 37:45-9, 2017

**Fig. 1 (abstract P265).**
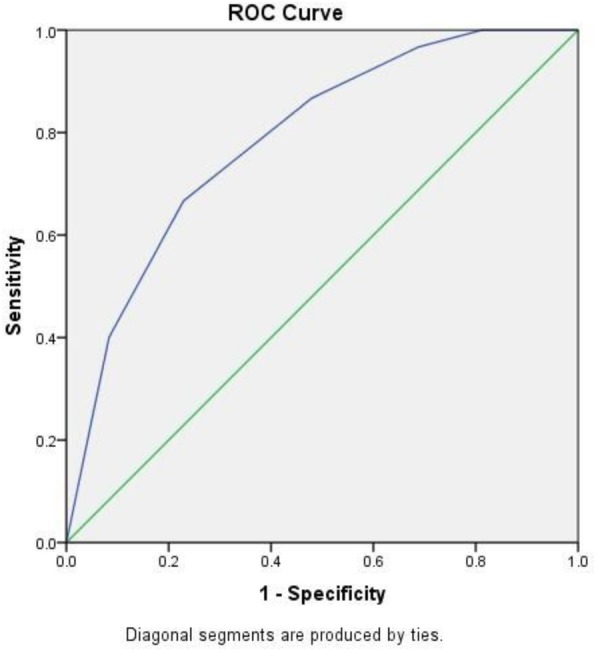
mNUTRIC in predicting 28-day mortality

### P266 Use of indirect calorimetry to determine caloric requirements of critically ill mechanically ventilated patients in an Asian surgical intensive care unit

#### N Lim^1^, I Kerner^2^

##### ^1^Changi General Hospital, Anaesthesia & Surgical Intensive Care, Singapore, Singapore; ^2^Changi General Hospital, Dietetics, Singapore, Singapore

**Introduction:** Estimation of caloric requirements in critically ill patients is an important part of nutrition assessment in the intensive care unit (ICU). In the absence of indirect calorimetry (IC), which is regarded as the gold standard as it measures resting energy expenditure (REE) [1], clinicians rely on predictive equations. However, many of these equations correlate poorly with IC measurements and validation studies in our Asian population are lacking.

**Methods:** We performed up to five IC measurements each on 57 mechanically ventilated patients (mean age 62 +/- 16 years; mean BMI 24 +/- 5.5 kg/m2; mean APACHE II score 18 +/- 8.45, mean SOFA score on admission 6.5 +/- 3.2). We also calculated REE using different predictive equations (Harris-Benedict (HBE), Schofield, Penn State University (PSU), Ireton Jones 1998 & 1992 (IJE), Mifflin-St-Jeor (MSJ), 25 kCal/kg of actual and adjusted body weight). These were compared to the IC measurements. The equation was considered accurate if the percentage difference fell within 10% of the measured value. Statistical analysis was performed using SPSS, version 19.0.

**Results:** Mean measured REE was 1940 +/- 540 kCal (range 1100-3390 kCal). All the equations were out of the acceptable range from 45% for HBE to 75% for PSU. Exclusion of obese and underweight patients did not improve the performance of the equations. Subgroup analysis of the neurosurgical patients (n=24) showed a similar trend. The highest incidence of underestimation was found for the 25 kCal/kg calculation when using adjusted body weight; however it also showed the lowest incidence of overestimation.

**Conclusions:** Overall accuracy of predictive equations was 39%. Both under- and over-estimations were observed. IC is a valuable tool to guide nutrition therapy in the acute phase of critical illness. The impact on patient outcome will have to be determined by further studies

**Reference**

1. McClave SA, Martindale RG, Kiraly L. Curr Opin Clin Nutr Metab Care 2013; 16:202-8

### P267 The condition of orally fed patients in ICU: an observational study

#### X Mathy^1^, L Rougier^1^, M Fadeur^2^, AM Verbrugge^2^, N Paquot^2^, JC Preiser^3^, AF Rousseau^4^

##### ^1^Dept of Anesthesiology, University Hospital, University of Liège, Liège, Belgium; ^2^Multidisciplinary Nutrition Team, University Hospital, University of Liège, Liège, Belgium; ^3^Dept of Intensive Care, Erasme University Hospital, Brussels, Belgium; ^4^Dept of Intensive Care and Burn Center, University of Liège, Liège, Belgium

**Introduction:** Malnutrition during intensive care unit (ICU) stay is associated with poor outcomes. Oral nutrition is rarely considered in ICU related nutritional guidelines and trials. Aim of this observational study was to quantify nutritional intakes in patients who were exclusively orally fed (OFP), compared to artificially fed patients, and to determine if major outcomes could have been affected.

**Methods:** Study was conducted during 17 weeks in 2016 and 2017 in a mixed tertiary ICU. Adults with ICU length of stay (LOS) >3d were included. Energy (E) and protein (P) requirements were determined according to age, body mass index and clinical parameters. Artificial or oral nutritional intakes (quartile method), and non-nutritional caloric intakes were noted on a daily basis. Intakes of first and last ICU days were not considered. Outcomes included mortality, hospital LOS and cumulated duration of antibiotic therapy. Data were expressed as median (min-max) or percentages.

**Results:** We enrolled 289 patients (59% male). Age was 67 (18-89)y, BMI was 25.7 (11.7-60.5)kg/m2 and SAPS II score was 36 (6-104). All fed patients (88%) received significantly less E and P than predicted requirements. First intakes occurred 1 (0-10)d after admission. Deficits were more pronounced in OFP (n=126): E and P intakes were respectively 9.7 (0.6-36.8) kcal/kg/d and 0.35 (0.1-1.38) g/kg/d. In OFP with ICU LOS ≥ 7d (n=37), 51% and 94% never received at least 80% of E and P requirements, respectively. Would they have eaten 100% of the meals when not fasting, they would have received 112% but only 67% of predicted respectively E and P requirements. Outcomes were not worse in OFP with ICU LOS ≥ 7d.

**Conclusions:** Present generalizable study is an alert on nutritional condition of OFP in ICU. Their P intakes were dramatically low. Oral nutrition requires as much attention as any other support. Intakes should be closely monitored; supplements prescriptions should be individualized on a daily basis.

### P268 Ultrasound (US) assessment of gastric content, volume and nasal/ oral gastric tube placement in general intensive care unit (GICU) (preliminary results)

#### E Brotfain^1^, A Erblat^2^, P Luft^2^, A Elir^2^, L Koyfman^2^, M Friger^3^, I Livshiz-Riven^4^, L Israeli^2^, J Nagorny^2^, A Grivnev^2^, A Zlotnik^2^, M Klein^2^

##### ^1^Ben Gurion University of the Negev, Department of Anesthesiology and Critical Care, Beer Sheva, Israel; ^2^Ben Gurion University of the Negev, Department of Anesthesiology and Critical Care, General Intensive Care Unit, Soroka Medical Center, Ben-Gurion University of the Negev, Beer Sheva, Israel; ^3^Ben Gurion University of the Negev, Department of Public Health, Faculty of Health Sciences, Ben-Gurion University of the Negev, Beer Sheva, Israel; ^4^Ben Gurion University of the Negev, Infection Control Unit, Soroka Medical Center. Faculty of Health Science, Ben-Gurion University of the Negev, Beer Sheva, Israel

**Introduction:** Ultrasonography is an essential imaging modality in critical care to diagnose and guide for therapeutic management of shock, multiple organ failure, etc. Enteral tube feed intolerance occurs frequently in hospitalized patients and more so in critically ill patients. In present study, we consider that nursing staff may be able to use bedside ultrasound as an alternative to standard aspiration protocol or radiographic studies to assess gastric volume and nasogastric (NG) tube in patients with enteral feed intolerance.

**Methods:** In present prospective, single-center study, we performed ultrasound residual stomach volume and NG tube placement assessments of adult critically ill patients (Figure 1) compared to standard protocol of stomach volume assessment (routine daily shift 60-ml syringe aspirations) and NG (nasogastric) tube placement verified by abdominal X ray. We used an abdominal (Linear Ultrasound Transducer) probe (4-10 MHz). The residual volume was calculated according to formula: GV (ml) = 27+14.6 x right-lateral CSA-1.28 x age).

**Results:** Hundred simultaneous double (ten critically ill patients) ultrasound measurements sessions were performed by nursing staff of our intensive care (ICU) (Fig 1). Double simultaneous measurements of the ultrasound assessments were compared to standard nurse ICU protocol for assessment of residual volume of stomach. The new ultrasound assessment method demonstrated excellent intra-class reliability (ICC- 0.96 (0.95-0.98, p<0.001) and strong correlation with standard residual volume assessment method (ICC-0.82 (0.7-0.89, p<0.001). NG tube placement was successfully verified by ultrasound measurements in all ten critically ill patients and, thereafter, confirmed by abdominal X-rays.

**Conclusions:** Preliminary results of our study demonstrated good correlations between both methods of NG tube placement and residual stomach volume: standard ICU nurse protocol and ultrasound assessment.

**Fig. 1 (abstract P268).**
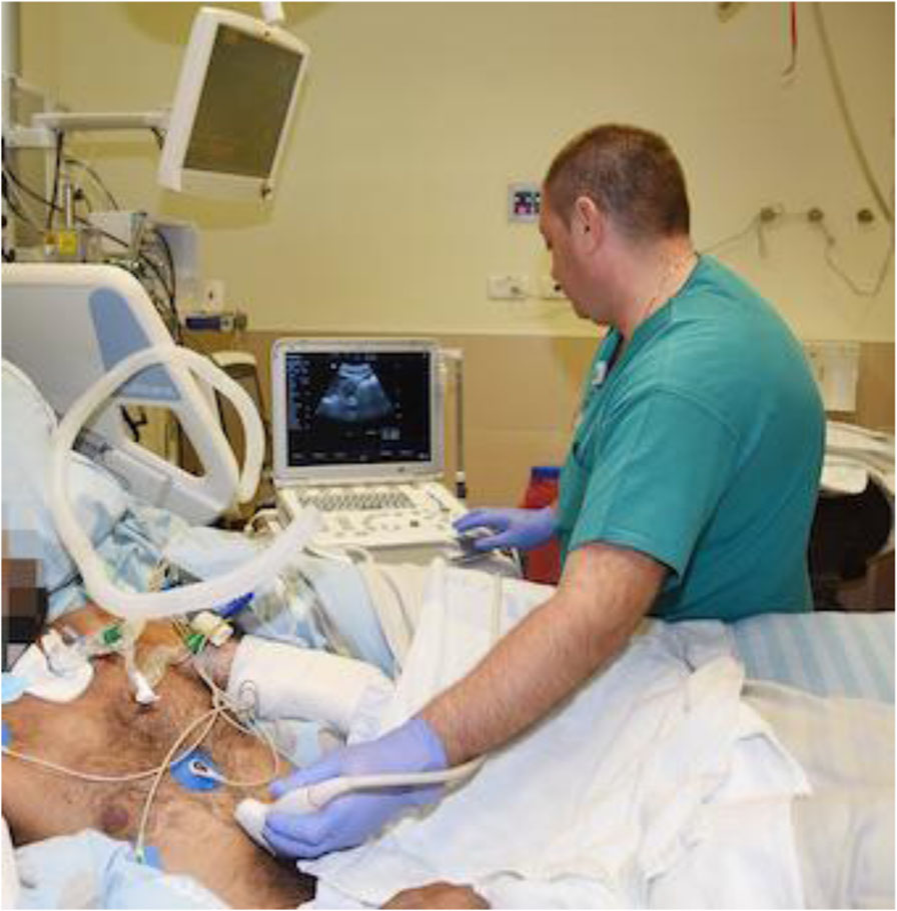
Ultrasound-guided residual stomach volume and nasogastric (NG) tube placement assessments performed by ICU nurse staff

### P269 Evaluating the documentation of nasogastric tube insertion and adherence to safety checking

#### L Roberts^1^, D Hepburn^2^, J Pugh^1^, D Penney^1^

##### ^1^Cardiff University, School of Medicine, Cardiff, United Kingdom; ^2^Aneurin Bevan Health Board, Intensive Care Unit, Newport, United Kingdom

**Introduction:** Enteral feeding into a misplaced nasogastric (NG) tube is recognised by the National Patient Safety Agency as a Never Event. NG tubes are commonly indicated in level 2/3 patients, thus we set out to evaluate current practice in critical care. The aim was to evaluate: documentation of insertion, adherence to safety guidance pertaining to checking safe use, chest x-ray interpretation.

**Methods:** This prospective cohort study was based on inpatients in critical care who had insertion of NG tubes over four weeks; there were 57 insertions. Data was analysed from patients’ medical notes and the hospital’s imaging system.

**Results:** 65% of insertions were documented using proformas. 97.1% of proforma documentations included 4 or more details: type of tube, tube length at the nostril, NEX measurement, aspirate adequacy, chest x-ray adequacy, whether it was safe to feed. Only 13.6% of hand-written documentations included 4 or more details.

51% of initial aspirates were obtained on insertion, of these, 57% had an appropriate pH between 1 and 5.5. This led to 74% of patients having chest x-rays to confirm initial placement of the NG tube. Only 54% of chest x-rays adequately satisfied the four criteria. Written documentation in medical notes stating if it was safe to feed was completed in 67% of cases.

**Conclusions:** We found that proformas ensure a higher level of detail and uniformity in the documentation of NG tube insertions. There was a high incidence of chest x-rays performed to confirm correct placement of tubes due to difficulties in obtaining aspirates and failure to follow guidelines. A need for a uniform, ward-specific proforma on NG tube insertion has been identified, as well as a teaching session on chest x-ray interpretation and on techniques to aid obtaining aspirates. We have established critical care’s shortcomings in NG tube insertion documentation and tube safety checking.

### P270 Investigation of the efficiency of curcumin and fish oil during the formation of pressure ulcer in mice

#### S Tultak^1^, E Karakoç^2^, B Yelken^3^, E Dündar^3^

##### ^1^Şemdinli State Hospital, Hakkari, Turkey; ^2^Eskisehir Osmangazi University Faculty of Medicine, Anaesthesiology and Reanimation Intensive Care Unit, Eskisehir, Turkey; ^3^Eskisehir Osmangazi University Faculty of Medicine, Pathology, Eskişehir, Turkey

**Introduction:** Pressure ulcers(PU) are considered as important types of public health problems, due to high mortality and cost. We aimed to investigate the efficiency of curcumin and fish oil on prevention and treatment of PU using a feasible mice model.

**Methods:** 28 mice were randomly divided into Control(Group 1), Curcumin(Group 2), Fish oil(Group 3), Curcumin and Fish oil(Group 4) groups. 5mm skin bridge between two 1000 gauss magnets was formed on the back of mice, followed by 3 ischemia reperfusion cycles as 12 hours of rest after 12 hours of magnet placement [1]. A single dose of curcumin and fish oil was injected intraperitoneally. Tissue samples had taken 10th day of first compression, rates of PU, inflammation, reepithelisation, neovascularisation and granulation were examined histopathologically. The data analyzed by Pearson chi-square test.

**Results:** Third degree PU were observed in all groups.There was no significant difference between groups in terms of inflammation.The formation of reepithelisation showed a significant difference between groups.Partial reepithelisation ratios in group 3 and group 4 was elevated.There was significant difference between groups in terms of neovascularisation, the highest rate as 75% was observed in group 4.Formation of granulation was observed at maximum rate as 46.2% at group 3.

**Conclusions:** Depending on positive results of curcumin, fish oil, curcumin+fish oil on wound healing it may be advised to use them in treatment of acute PU.After similar rate of PU with control group we consider that it should be beneficial to evaluate the effect of these therapies with more studies by changing the mode of administration, time of initiation and duration of therapy.

**Reference**

Stadler I et al. J Invest Surg 2004 17;221-7

### P271 Does inflammation influence the response to nutritional therapy in patients with disease-related malnutrition? - Secondary analysis of a randomized multicenter trial

#### M Merker^1^, M Felder^2^, L Gueissaz^2^

##### ^1^Kantonsspital Aarau, Allgemeine Innere Medizin, Aarau, Switzerland; ^2^University, Medical student, Basel, Switzerland

**Introduction:** Inflammation is a key driver of malnutrition during acute illness and has different metabolic effects including insulin resistance and reduction of appetite. Whether inflammation influences the response to nutritional therapy in patients with disease-related malnutrition remains undefined. We examined whether the effect of nutritional support on the risk of mortality differs based on the inflammatory status of patients.

**Methods:** This is a secondary analysis of a multicentre trial in eight Swiss hospitals, where patients with a nutritional risk score (NRS) of ≥3 upon hospital admission were randomly assigned to receive protocol-guided individualized nutritional support according to nutrition guidelines (intervention group) or a control group. The inflammatory status was defined based on admission CRP levels as low inflammation (CPR <10 mg/dl), moderate inflammation (CRP 10-100 mg/dl) and high inflammation (CRP >100 mg/dl).

**Results:** We included a total of 1,950 patients of which 27.33%, 45.85% and 26.82% had low, moderate and high inflammation levels on admission. While overall there was a significant reduction in 30-day mortality associated with nutritional support (adjusted OR in the overall cohort 0.65, 95%CI 0.46 - 0.91), the subgroup of patients with high inflammation did not show reduced mortality (adjusted OR 1.36, 95%CI 0.76 - 2.41, p for interaction = 0.005). There was no difference in other secondary endpoints when stratified based on inflammation. Nutritional support did not affect CRP levels over time (kinetics).

**Conclusions:** This secondary analysis of a multicentre randomized trial provides evidence, that the inflammatory status of patients influences their response to nutritional support. These findings may help to better individualize nutritional therapy based on patients initial presentation.

### P272 Clinical indicators of low plasma glutamine on admission to ICU

#### R Blaauw^1^, N Van Wyk^1^, D Nel^2^, G Scheicher^3^

##### ^1^Stellenbosch University, Human Nutrition, Cape Town, South Africa; ^2^Stellenbosch University, Centre for Statistical Consultation, Cape Town, South Africa; ^3^Wits Donald Gordon Medical Centre ICU, Parktown, South Africa

**Introduction:** Low plasma glutamine levels have been associated with unfavourable outcomes in critically ill patients. This study aimed to measure plasma glutamine levels in critically ill patients and to correlate glutamine levels with biomarkers and severity of illness.

**Methods:** We enrolled critically ill patients admitted to three ICUs in South Africa, excluding those receiving glutamine supplementation prior to admission. We collected clinical, biochemical and dietary data. Plasma glutamine levels were determined within 24 hours of admission, using Liquid Chromatography Mass Spectrometry and categorized as low (<420 μmol/L), normal (420-700 μmol/L) and high (>700 μmol/L).

**Results:** Of the 330 patients (average age 47.42±16.56 years, 56% male), 68% were mechanically ventilated, with a mean APACHE II score of 18.57±8.55 and a mean SOFA score of 7.05±3.78. Plasma glutamine levels were low in 58.5% (median plasma glutamine of 382.15 μmol/l). Baseline plasma glutamine correlated inversely with CRP (r=-0.287, p<0.001) and serum urea (r=-0.124, p<0.026), and positively with serum bilirubin (r=0.262, p<0.001) and serum ALT (r=0.119, p=0.032). Significantly more patients with low admission glutamine levels required mechanical ventilation (Chi2=12.65, p<0.001) and had higher APACHE scores (p=0.003), higher SOFA scores (p=0.003), higher CRP values (p<0.001), higher serum urea (p=0.008), higher serum creatinine (p=0.023), lower serum albumin (p<0.001) and lower bilirubin levels (p=0.054). Using multiple logistic regression analysis, APACHE score (odds ratio, [OR] 1.032, p=0.018), SOFA score (OR 1.077, p=0.016) and CRP (OR 1.006, p<0.001) were significant predictors of low plasma glutamine levels. ROC curve analysis revealed a CRP threshold value of 87.95mg/L to be indicative of low plasma glutamine levels (AUC 0.7, p<0.001).

**Conclusions:** 58.5% of critically ill patients had low plasma glutamine levels on admission to ICU. This was associated with increased disease severity and higher CRP.

### P273 A quality improvement project of the TPN Branch of the Magnificent 7 Care Bundle – Cycle Two

#### A Webb^1^, P Bishop^2^

##### ^1^Colchester Hospital, Intensive Care, Colchester, United Kingdom; ^2^Colchester Hospital, Anaesthetics and Intensive Care, Colchester, United Kingdom

**Introduction:** The East of England deanery Operational Delivery Network in the United Kingdom came together as a group of Intensive Care Units to comply an evidence-based care bundle. One of the branches of this care bundle is on parenteral nutrition and states: ‘Parenteral nutrition should not be given to adequately nourished, critically ill patients in the first seven days of an ICU stay.’ This is based on evidence [1-3] that showed that ‘In patients who are adequately nourished prior to ICU admission, parental nutrition initiated within the first seven days has been associated with harm, or at best no benefit, in terms of survival and length of stay in ICU.´ The objective of this second cycle was to assess whether or not we are adhering to the guidelines, last year we were failing to hit targets and after some action I reassessed how we performed in the year 2017 compared to 2016.

**Methods:** A retrospective audit of the whole year of 2017 for all patients admitted to ICU who had parenteral nutrition started at any point during their stay.

**Results:** There is a significant improvement in the percentage of patients who are being started incorrectly on TPN before 7 days (36% compared to 69%) (Fig 1, 2). I also found a total reduction in the number of patients prescribed TPN, a reduction in the number of bags being used and a reduction in length of hospital stays.

**Conclusions:** As we have recently switched over to an electronic ICU programme for all documentation and prescriptions, as part of our plan and act in the PDSA cycle we are organising for several things to be put in place on the new system on prescription: pharmacy authorisation, links to guidelines and alert/justification boxes. I will do a further cycle in another year.

**References**

1) Casaer MP, Mesotten D et al. N Engl J Med. 2011; 365: 506-17

2) Guidelines for nutrition support therapy in the adult critically ill patient. Crit Care Med 2009; 37: 1757-61

3) Buzby GP. World J Surg 1993;17: 173-7

**Fig. 1 (abstract P273).**
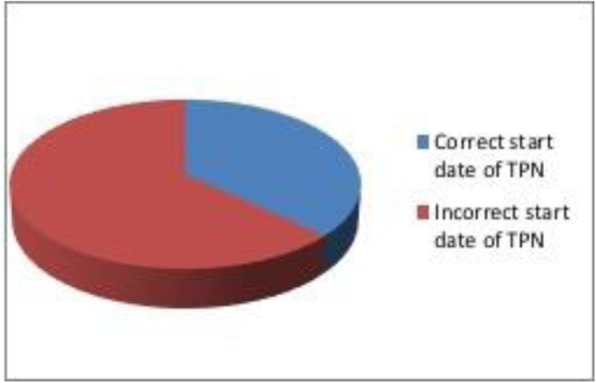
2016 appropriate vs inappropriate TPN prescriptions

**Fig. 2 (abstract P273).**
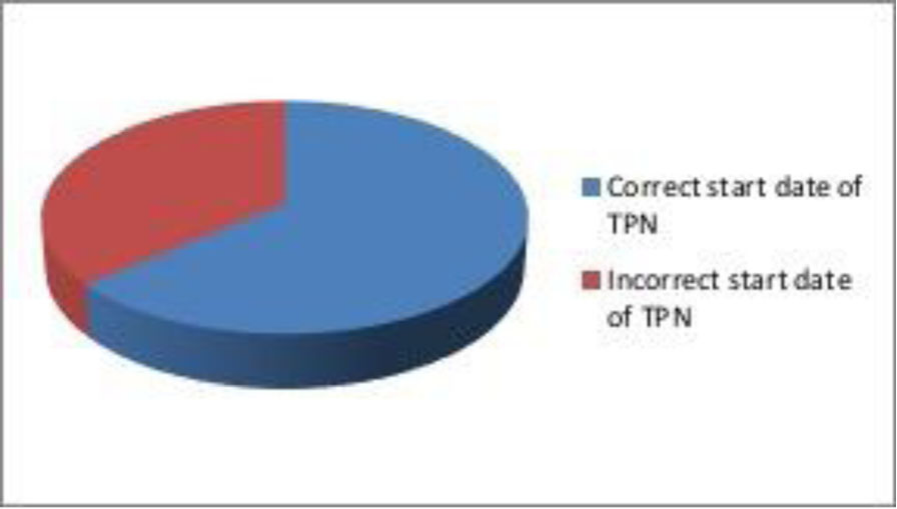
2017 appropriate vs inappropriate TPN prescriptions

### P274 Designing a fasting-mimicking diet for the prolonged critically ill: the pilot ICU-FM-1 study

#### L Van Dyck, I Vanhorebeek, A Wilmer, A Schrijvers, I Derese, PJ Wouters, G Van den Berghe, J Gunst, MP Casaer

##### KU Leuven, Laboratory of Intensive Care Medicine, Leuven, Belgium

JG and MPC contributed equally.

**Introduction:** Recent RCTs revealed clinical benefit of early macronutrient restriction in critical illness, which may be explained by enhanced autophagy, an evolutionary conserved process for intracellular damage elimination [1]. However, in the absence of specific and safe autophagy-activating drugs, enhancing autophagy through prolonged starvation may produce harmful side effects. A fasting-mimicking diet (FMD) may activate autophagy while avoiding harm of prolonged starvation, which also improved biomarkers of age-related diseases in an experimental study [2]. We evaluated if short-term interruption of continuous feeding can induce a metabolic fasting response in prolonged critically ill patients.

**Methods:** In a randomized cross-over design, 70 prolonged critically ill patients receiving artificial feeding were randomized to be fasted for 12 hours, followed by 12 hours full enteral and/or parenteral feeding, or vice versa. Patients were included at day 8 in ICU and blood glucose was maintained in the normal range. At the start and after 12 and 24 hours, we quantified total bilirubin, urea, insulin-like growth factor-I (IGF-I) and beta-hydroxybutyrate (BOH) in arterial blood. Insulin requirements were extracted from patient files. Changes over time were analyzed by repeated-measures ANOVA after square root transformation.

**Results:** As compared to 12 hours of full feeding, 12 hours of fasting decreased bilirubin (-0.23±0.06mg/dl; p=0.001) and IGF-I (-13.94±1.62ng/ml; p<0.0001), and increased BOH (+0.47±0.07mmol/l; p<0.0001), without affecting urea concentrations (Fig 1). Fasting reduced insulin requirements (-1.16±0.14IU/hour; p<0.0001).

**Conclusions:** Short-term fasting induces a metabolic fasting response in prolonged critically ill patients, which provides perspectives for the design of a FMD, aimed at activating autophagy and ultimately at improving outcome of critically ill patients.

**References**

1. Casaer MP et al. N Engl J Med 370:1227-36, 2014;

2. Brandhorst S et al. Cell Metab 22:86-99, 2015.

**Fig. 1 (abstract P274).**
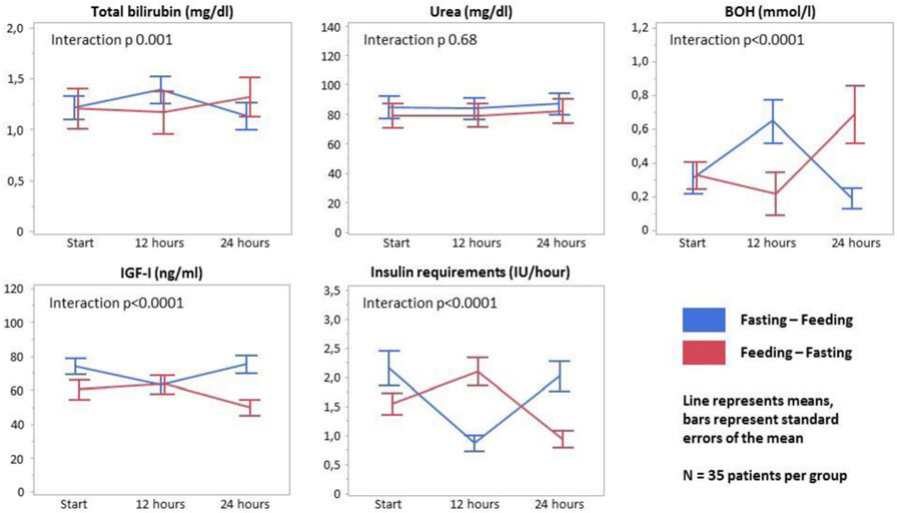
Changes in metabolic markers of fasting over time for both randomization groups

### P275 Current practice and variability in micronutrient monitoring and administration: results of the Vita-Trace survey

#### W Vankrunkelsven^1^, J Gunst^1^, K Amrein^2^, DE Bear^3^, MM Berger^4^, KB Christopher^5^, V Fuhrmann^6^, MJ Hiesmayr^7^, MJ Hiesmayr^7^, C Ichai^8^, SM Jakob^9^, S Lasocki^10^, JC Montejo^11^, HM Oudemans-van Straeten^12^, JC Preiser^13^, A Reintam Blaser^14^, AF Rousseau^15^, P Singer^16^, J Starkopf^17^, AR Van Zanten^18^, S Weber-Carstens^19^, J Wernerman^20^, A Wilmer^21^, MP Casaer^1^

##### ^1^KU Leuven, Laboratory of intensive care medicine, Leuven, Belgium; ^2^Medical University of Graz, Division of Endocrinology and Diabetology/ department of Internal Medicine, Graz, Austria; ^3^Guy´s and St Thomas´ NHS Foundation Trust, Department of Critical Care And Department of Nutrition and Dietetics, London, United Kingdom; ^4^University of Lausanne Hospital - CHUV, Service of Intensive Care Medicine & Burns, Lausanne, Switzerland; ^5^Brigham and Women´s Hospital, Division of Renal Medicine, Boston, United States; ^6^University Medical Center Hamburg-Eppendorf, Department for Intensive Care Medicine, Hamburg, Germany; ^7^Medical University of Vienna, Department of Anesthesia, General Intensive, Vienna, Austria; ^8^University Côte d´Azur, CHU de Nice, Hôpital Pasteur 2, Department of Anesthesiology and Critical Care Medicine, Nice, France; ^9^Inselspital - Bern University Hospital - University of Bern, Department of Intensive Care Medicine, Bern, Switzerland; ^10^Centre hospitalier universitaire d´Angers, Département Anesthésie-Réanimation, Angers, France; ^11^Hospital Universitario 12 de Octubre, Intensive Care Medicine Department, Madrid, Spain; ^12^Amsterdam Medical Centers, Department Adult Intensive Care, Amsterdam, Netherlands; ^13^Erasme University Hospital - Université Libre de Bruxelles, Department of Intensive Care, Brussels, Belgium; ^14^Lucerne Cantonal Hospital, Department of Intensive Care Medicine, Lucerne, Switzerland; ^15^University Hospital of Liège, Intensive Care Department and Burn Centre, Liège, Belgium; ^16^Rabin Medical Center, Tel Aviv University, General Intensive Care Department and Institute for Nutrition Research, Tel Aviv, Israel; ^17^University of Tartu - Tartu University Hospital, Department of anaesthesiology and intensive care, Tartu, Estonia; ^18^Gelderse Vallei Hospital, Department of Intensive Care, RP Ede, Netherlands; ^19^Charité - Universitätsmedizin Berlin, Department of Anesthesiology and operative intensive care medicine, Berlin, Germany; ^20^Karolinska University Hospital Huddinge - Karolinska Institutet, Intensive Care Medicine, Stockholm, Sweden; ^21^KU Leuven, Medical Intensive Care, Leuven, Belgium

**Introduction:** Recent evidence has led to changed feeding guidelines for critically ill patients, with a shift towards lower feeding targets during the acute phase [1]. When micronutrients are not provided separately, prolonged hypocaloric feeding could induce micronutrient deficiencies and increase risk of refeeding syndrome once full feeding is restarted, which are both potentially lethal complications [2]. Since there is limited evidence how to optimize micronutrient provision in order to avoid deficiencies, we hypothesized that there is a great variation in current practice.

**Methods:** Within the MEN section of the European Society of Intensive Care Medicine (ESICM), we designed a questionnaire to gain insight in the current practice of micronutrient administration. In 3 email blasts, invitations were sent to all ESICM members, with currently more than 300 respondents. The survey will be closed at December 10, 2018.

**Results:** First, we will describe demographic characteristics of the respondents, including geographical location, ICU and hospital type, and function. Second, we will describe some aspects of the current practice of micronutrient administration. We will identify the proportion of respondents having a protocol, on which evidence such protocol is based and whether it takes into account the stability and daylight sensitivity of micronutrients. Next, bearing refeeding syndrome in mind, we will identify whether there are respondents who never measure and/or separately administer micronutrients and phosphate. Finally, we will make a top 5 of the most measured and most supplemented micronutrients.

**Conclusions:** This survey will deliver more insight in the current practice of micronutrient provision across different types of ICUs and may identify areas for future research. Furthermore, we will evaluate whether there is need to increase awareness for refeeding syndrome.

**References**

1. Singer P et al. Clin Nutr (2018). doi: 10.1016/j.clnu.2018.08.037

2. Doig GS et al. Lancet Respir Med 3, 943-952 (2015).

### P276 Machine-learning based prediction and stratification of patients with enteral feeding intolerance and quantification of clinical outcomes

#### S Mataraso^1^, S Panchavati ^1^, C Almansa^2^, M Subramanyam^2^, G Dukes^2^, C Barton^1^, J Hoffman^1^, R Das^1^

##### ^1^Hessian Pharmaceuticals, Inc., Hayward, United States; ^2^Takeda Pharmaceuticals, Inc., Cambridge, United States

**Introduction:** Large gastric residual volumes (GRVs) have been used as surrogate markers of delayed gastric motility to define enteral feeding intolerance (EFI). Recent studies have challenged the definition of EFI. Study objectives: 1) investigate the potential relationship between GRVs and clinically outcomes, 2) develop an algorithm for early identification of patients at increased risk of mortality due to EFI.

**Methods:** A retrospective study of inpatient encounters from electronic health record charts within the Dascena Clinical Database. 4,018 patients were included in the study; 295 patients had EFI. Eight vital signs (diastolic/systolic BP, heart rate, temperature, respiratory rate, GRV, Glasgow Coma Scale, and feeding rate) and their trends were input to the classifier. Machine learning classifiers were created using the XGBoost gradient boosted tree method with 3-fold cross validation.

**Results:** Rate of change in GRV (Δ GRV) was measured over a 5-day period, beginning at the time of EFI onset (Figure 1a). Figure 1b shows a high likelihood of mortality for patients with none or modest GRV reduction. Patients with an increase in GRV over the five-day period after EFI onset had the highest mortality likelihood. A stratification algorithm was developed to identify EFI patients who died in-hospital despite GRV reduction at 1, 12, and 24 hours in advance of EFI onset. Area under the receiver operating characteristic (AUROC) curves demonstrated high sensitivity and specificity of algorithm predictions of in-hospital death up to 24 hours in advance of EFI onset (Table 1).

**Conclusions:** The analysis suggests an association between GRV and mortality, especially in patients with persistent GRV increase over the 5-day period after EFI onset and the potential of algorithmic models to predict EFI development. Prospective validation of these algorithms may assist in clinical trial design to develop treatments for patients at highest risk of experiencing serious outcomes due to EFI.

**Table 1 (abstract P276).** Comparison of performance metrics of machine learning stratification algorithms for patient death despite GRV reduction at various prediction time windows in advance of EFI onset

	1 hr	12 hr	24 hr
AUROC (± CI)	0.977 (± .002)	0.951 (± .015)	0.930 (± .015)
Sensitivity	0.90	0.81	0.85
Specificity	0.96	0.93	0.93
DOR	424.33	68.33	175.23

AUROC, area under the receiver operator characteristic curve; CI, confidence interval; DOR, diagnostic odds ratio

**Fig. 1 (abstract P276).**
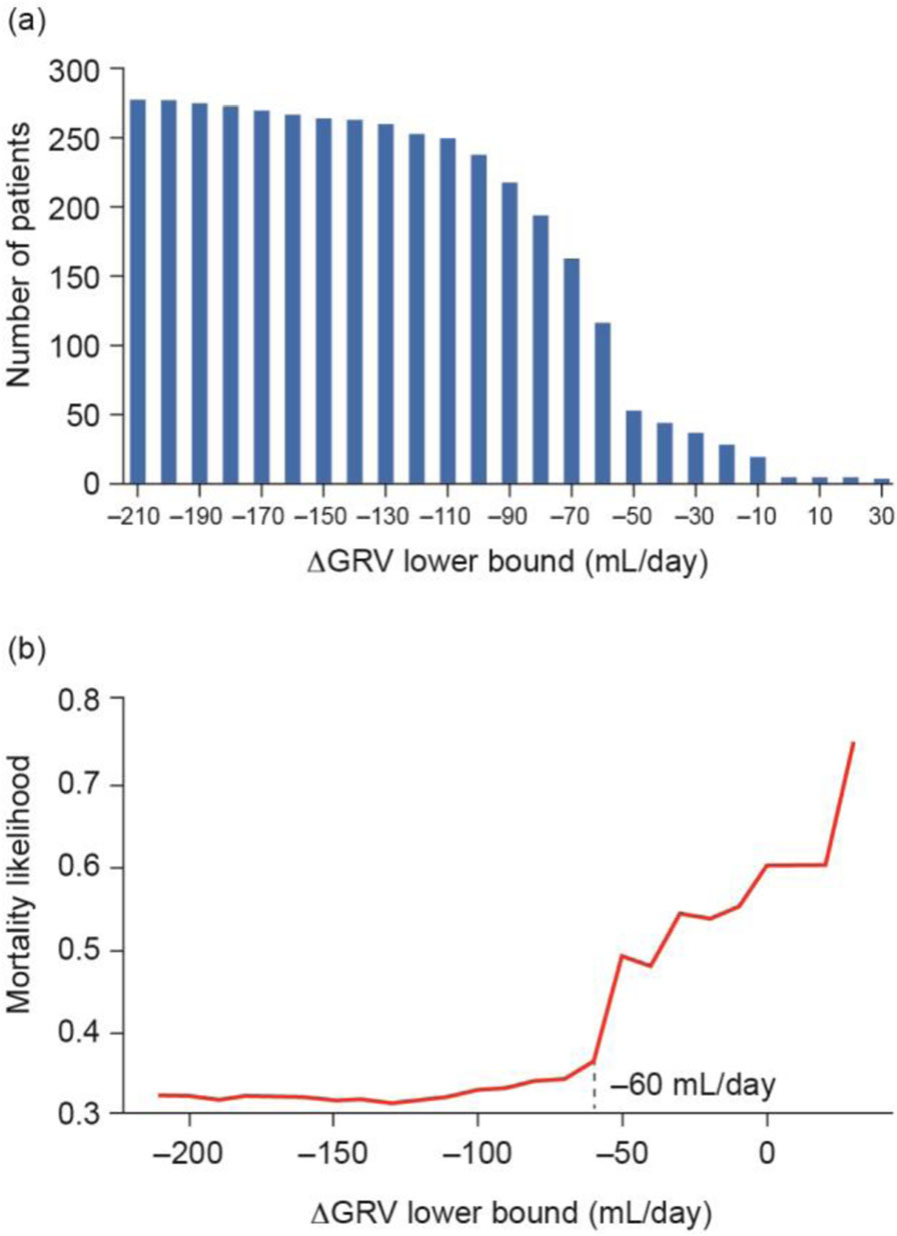
Distribution of patients with high ∆GRV (Panel a) and mortality likelihood as a function of ∆GRV (Panel b). For each ∆GRV value, the bar represents the number of people with a ∆GRV no smaller than the specified value (Panel a). The data in Panel b is obtained from Panel a by taking the proportion of each bar’s deceased patients

### P277 A quality improvement project to improve the daily calorific target delivery via the enteral route in critically ill patients in a mixed surgical and medical intensive care unit (ICU)

#### B Johnston, D Long, R Wenstone

##### Royal Liverpool and Broadgreen University Hospital Trust, Critical Care, Liverpool, United Kingdom

**Introduction:** ‘Iatrogenic underfeeding’ is widespread with the CALORIES study reporting only 10%-30% of prescribed daily kCal was actually delivered to patients [1]. In the present project, quality improvement methodology was utilised with the aim of delivering greater calories by implementing 24-hour volume-based feeding and allowing increased feeding rates for, ‘catch up’ of missed daily feed volume.

**Methods:** Baseline data assessing the percentage of daily kCal delivered to ventilated patients was collected in September 2017. Data was presented and new intervention guidelines agreed based upon the PEPuP protocol [2]. Nurse champions were identified and were responsible for cascade training of the PEPuP protocol. Educational tools to help determine daily calorific requirement and volume of feed required were provided. Repeat data was collected at 6 months (cycle 2) after PEPuP implementation.

**Results:** Ten patients were included in cycle 1. During cycle 1 the percentage of kCal achieved via enteral feeding was 45%. Following intervention this increased to 82% (p<0.001) during cycle 2. This increased further to 95.3% of daily kCal when calories obtained from Propofol were included.

**Conclusions:** A 24-hour volume-based feeding regimen is a simple and cost-effective method of improving enteral feeding targets. Through the use of quality improvement methodology, we demonstrated that this approach is achievable. The success of this project has led to the adoption of the protocol in other ICU units in a regional critical care network.

**References**

[1] Harvey SE et al. N Engl J Med. 371:1673–1684, 2014

[2] Heyland D et al. J Parenter Enter Nutr 39, 698–706, 2015

### P278 Effect of non-nutritional calories on the calory/protein ratio in ICU patients

#### S Jakob, J Takala

##### University Hospital Bern, Dept of Intensive Care Medicine, Bern, Switzerland

**Introduction:** Nutritional diets are composed to match the needs of critically ill patients. While effective calory needs can be measured or calculated, the needs of proteins are more controversial. We aimed to calculate non-nutritional calories and assess how they influence the ratio of calories to protein delivered to the patients.

**Methods:** In this retrospective analysis, nutritional and non-nutritional calories and protein delivery were calculated in 220 consecutive ICU patients receiving enteral nutrition in 2016.

**Results:** 154 male and 66 female patients with a mean SAPS II score of 60 (range 14-107), age of 63 years (14-89 years), and weight of 82 kg (35-187 kg) were included. 191 were emergency and 29 elective patients. They spent 8 days (0.3-65 days) in the ICU. According to Harris Benedict formula, their basal metabolic rate was 1595 KCal (961-3113 KCal). Dependent on their ICU length of stay they received on average 10 (<5 d), 15 (≥5 d), and 18 (≥10 d) KCal/kg/day, respectively, and 0.3, 0.7, and 0.8 g/protein/kg/d. Non-nutritional calories, derived from glucose (average 61%) and propofol infusions (39%), respectively, represented 14% (0-91%) of totally administered calories. The calory/protein ratio increased from 21+/-2 (mean+/-SD) to 27+/-18 when the administration of non-nutritional calories were taken into account (p<0.001; paired t-Test).

**Conclusions:** In critically ill patients, significant proportions of calories are administered with glucose and propofol infusions. Since non-nutritional calories are derived from carbohydrates or lipids, their administration decrease the relative contribution of proteins to delivered calories. This should be considered when enteral nutrition is prescribed.

**Acknowledgment**

This study was partly supported by a grant from Nestle Health Science.

### P279 Optimizing protein balance is associated with outcome in neurointensive care unit

#### TJ Kim^1^, SH Park^1^, HB Jeong^1^, EJ Ha^2^, WS Cho^2^, HS Kang^2^, JE Kim^2^, SB Ko^1^

##### ^1^Seoul National University Hospital, Neurology, Seoul, South Korea; ^2^Seoul National University Hospital, Neurosurgery, Seoul, South Korea

**Introduction:** Marked protein catabolism is common in neurocritical patients. Optimal nutritional monitoring and protein nutritional adequacy could be associated with outcome in neurointensive care unit (NCU) patients. We aimed to evaluate the impact of monitoring and optimal support of protein using nitrogen balance on outcome in neurocritical patients.

**Methods:** A consecutive 69 patients who were admitted to NCU were included between July 2017 and February 2018. Nitrogen balance was calculated using excreted urine urea nitrogen during ICU admission. Follow-up nitrogen balance monitoring was performed in 33 patients. We divided patients into two groups based on the results of nitrogen balance (positive balance and negative balance). Moreover, we evaluated improvement of nitrogen balance in 33 patients. We assessed the outcome as length of stay in hospital, length of stay in NCU, and in-hospital mortality. We compared the clinical characteristics and outcome according to nitrogen balance.

**Results:** Among the included patients (age, 58.1; and male. 59.4%), 53 (76.8%) patients had negative nitrogen balance. The negative balance group was more likely to have lower Glasgow Coma Scale (GCS), longer length of stay in hospital, and longer length of stay in NCU. In 33 patients with follow-up nitrogen balance monitoring, improvement of nitrogen balance group had lower in-hospital mortality (10.5% vs. 50.0%, P = 0.019), and received adequate protein intake (1.8 g/Kg/day vs. 1.1 g/Kg/day, P = 0.001) compared to no change group (Table 1). There was no significant difference in baseline nitrogen balance, baseline body mass index, and GCS between two groups.

**Conclusions:** This study demonstrated that critical illness patients in NCU are underfeeding using nitrogen balance, however, adequate provision of protein was associated improvement of nitrogen balance and outcome. This suggests that adequate nutrition monitoring and support could be an important factor for prognosis in neurocritical patients.

**Table 1 (abstract P279).** Clinical characteristics according to improvement of nitrogen balance

	Improvement of Nitrogen balance(n=1	No improvement of nitrogen balance (n=14, 42.4%)	p-value
BMI, mean (SD)	22.1 ± 4.7	22.8 ± 4.0	0.655
Initial GCS, median (IQR)	7 (5-12)	7.5 (3-13)	0.760
Baseline Nitrogen balance, mean (SD)	-8.8 ± 7.1	-7.0 ± 7.1	0.476
Initial Protein g/Kg	0.69 ± 0.59	0.64 ± 0.49	0.666
Baseline negative Nitrogen balance, n (%)	17 (89.5)	12 (85.7)	1.000
Changed Protein g/Kg	1.81 ± 0.58	1.12 ± 0.54	0.001
In hospital mortality, n (%)	2 (10.5)	7 (50.0)	0.019

### P280 Increased protein delivery within a hypocaloric protocol may be associated with lower 30-day mortality in critically ill patients

#### J Ochoa Gautier^1^, R Hussein^1^, A Berger^2^

##### ^1^Geisinger Health System, Critical Care, Scranton, United States; ^2^Geisinger Health System, Henry Hood Research Center, Scranton, United States

**Introduction:** To test the hypothesis, using real world evidence that increasing protein delivery and decreasing carbohydrates (CHO) may improve clinical outcomes.

**Methods:** Retrospective analysis of existing electronic medical records (EMR) of patients admitted to the intensive care units (ICU) at the Geisinger Health System. Logistic regression analysis was used to determine correlation between protein delivered (which was proportional to the concentration of protein in the formula utilized) and clinical outcomes.

**Results:** 2000 medical encounters for a total number of 12,321 ICU days were collected and analyzed. Average age was 62.2 years (55.2% male) and 68.1% were obese and overweight. Primary diagnoses included sepsis or septic shock, acute and/or chronic respiratory failure (or illness), cardiovascular diseases, stroke and cerebrovascular diseases among others. Median hospital LOS was 13.6 days, 6.9 days in the ICU, median days of invasive mechanical ventilation of 4. 30-day readmission rate among patients discharged alive was 19.3%. Patients in the High protein group received lower amounts of CHOs (data not shown). Unadjusted 30-day post-discharge mortality was inversely proportional to the amount of protein delivered (Table 1).

**Conclusions:** A significant improvement in mortality is observed with increased protein delivery while decreasing carbohydrate loads. Prospective randomized trials are warranted to establish causality.

**Table 1 (abstract P280).** Outcomes

Protein (P) (delivered/formula)	Low P	Intermediate P	High P	P-Value
Inpatient Death	81 (7.6%)	48 (7.4%)	20 (7.0%)	0.9250
30 Day Post Discharge Mortality	123 (12.6%)	62 (10.3%)	11 (4.1%)	0.0007
Composite Mortality Inpatient + 30-Day Post	204 (19.2%)	110 (16.9%)	31 (10.8%)	0.0040

### P281 Low tidal volume ventilation during cardiovascular surgery reduces the incidence of postoperative AKI: a retrospective cohort study

#### K Tojo, T Mihara, T Goto

##### Yokohama City Univerisy, Department of Anesthesiology and Critical Care Medicine, Yokohama, Japan

**Introduction:** Acute kidney injury (AKI) is a major complication after cardiovascular surgery. Although several risk factors have been reported, most of them are not easily modifiable. It has been reported that intraoperative low tidal volume (TV) ventilation can reduce postoperative respiratory complications. Moreover, low TV ventilation is known to attenuate systemic inflammatory responses. However, the effects of intraoperative low TV ventilation on remote organs other than lungs are not clear. We performed a retrospective cohort study to evaluate whether intraoperative low TV ventilation reduces the incidence of AKI after cardiovascular surgery.

**Methods:** Records of patients who undergone cardiovascular surgery between January 2009 and March 2017 were reviewed. The primary outcome was AKI diagnosed by the perioperative changes of serum creatinine values. Intraoperative mean TV relative to predicted body weight (PBW) was calculated. We divided patients into four groups: those who received mean TV ≤ 7, 7-8, 8-9, and >9 mL/kg PBW, and evaluated the incidence of AKI. Thereafter, we compared the incidence of AKI in patients who received TV ≤ 7 or >7mL/kg PBW using inverse probability of treatment weighting (IPTW) using the propensity score.

**Results:** Of the 340 patients included in the analysis, 106 patients developed AKI (31.2 [95% CI: 26.5-36.3] %). The incidence of AKI in the patients who received mean TV < 7, 7-8, 8-9, and >9 mL/kg PBW were 12.5 [5.9-24.7], 31.0 [23.7-39.4], 38.1 [29.4-47.6], and 34.5 [23.6-47.3] %, respectively. The results of IPTW analysis revealed that the risk of AKI in the patients who received mean TV < 7mL/kg PBW was significantly lower than those who received mean TV >7mL/kg PBW (odds ratio: 0.14 [0.05-0.43], p < 0.001).

**Conclusions:** This study suggests that intraoperative low TV ventilation during cardiovascular surgery seems to have a protective effect on kidney, a remote organ other than lungs, and may reduce the incidence of postoperative AKI.

### P282 Cumulative fluid balance is a risk factor for AKI development and progression

#### J Zhang^1^, N Seylanova^2^, S Crichton^3^, A Dixon^4^, ZY Peng^5^, M Ostermann^4^

##### ^1^Zhongnan hospital of Wuhan University, Department of Critical Care, Wuhan, China; ^2^Sechenov First Moscow State Medical University, Moscow, Russia; ^3^University College London, Medical Research Council Clinical Trials Unit, London, United Kingdom; ^4^Guy’s and St Thomas’ NHS Foundation Trust, London, United Kingdom; ^5^Zhongnan hospital of Wuhan university, Wuhan, China

**Introduction:** Acute kidney injury (AKI) is associated with high mortality. The risk increases with severity of AKI. Our aim was to identify risk factors for development and subsequent progression of AKI in critically ill patients.

**Methods:** We analysed 2525 patients without end-stage renal disease who were admitted to the ICU in a tertiary care centre between January 2014 to December 2016 and did not have AKI on admission. We identified risk

factors for development and non-recovery of AKI as defined by the KDIGO criteria.

**Results:** The incidence of new AKI in 7 days was 33% (AKI I 42%, AKI II 35%, AKI III 23%). Multivariate analysis revealed BMI, SOFA score, chronic kidney disease (CKD) and cumulative fluid balance as independent risk factors for development of AKI. Among patients who developed AKI in ICU, 69% had full renal recovery, 8% partial recovery and 23% had no recovery of renal function by day 7. AKI patients without renal recovery in 7 days had significantly higher hospital mortality (60%) compared to the other groups. Independent risk factors for non-recovery of renal function were CKD, mechanical ventilation, diuretic use and extreme fluid balance before and after first day of AKI. (Table 1) The association between cumulative fluid balance before AKI and 48 hours after AKI with risk of AKI non-recovery are shown in Figure 1 and 2.

**Conclusions:** AKI is common and mortality is highest in those who do not recover renal function. Cumulative fluid accumulation impacts chances of AKI development and progression.

**Table 1 (abstract P282).** Multivariable analysis: Risk factors for AKI development and non-recovery of AKI

Variables	Model 1 AKI development	P-value	Model 2 AKI nonrecovery ^1,2^	P-value
	OR (95% CI)		OR (95% CI)	
BMI	1.06(1.04,1.07)	<0.01		
SOFA score before AKI			1.00(0.91-1.09)	0.97
SOFA score after AKI			1.11(1.00-1.24)	0.06
Lowest MAP after AKI			0.99(0.96-1.02)	0.99
CKD in AKI development model	1.81(1.34,2.45)	<0.01		
CKD in before AKI model			1.98(1.08-3.64)	0.03
CKD in after AKI model			4.39(1.36-5.75)	0.01
Mechanical ventilation before AKI			2.30(1.20-4.44)	0.01
Mechanical ventilation after AKI			1.63(2.07-9.30)	<0.01
Norepinephrine use before AKI			1.44(0.73-2.83)	0.29
Norepinephrine use after AKI			1.67(0.61-4.56)	0.32
Diuretic use before AKI			1.90(1.18-3.06)	0.01

^1^also adjusted for the non-linear association between FB and non-recovery (p<0.001). See Fig 1 for estimated predicted probability of non-recovery by FB. ^2^also adjusted for the non-linear association between net FB and non-recovery (p=0.004). See Fig 2 for estimated predicted probability of non-recovery by net FB

**Fig. 1 (abstract P282).**
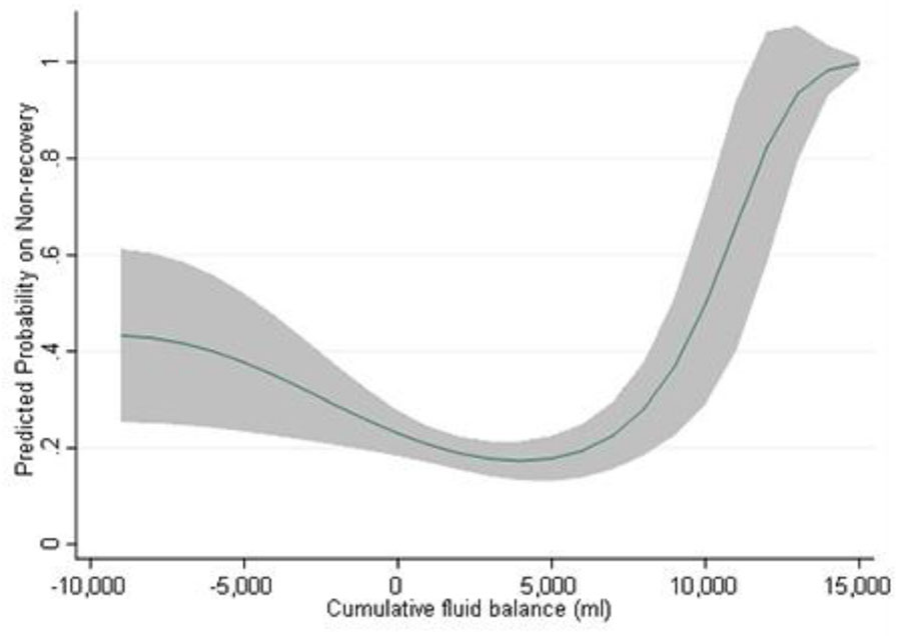
Association between cumulative fluid balance before AKI and risk of AKI non-recovery

**Fig. 2 (abstract P282).**
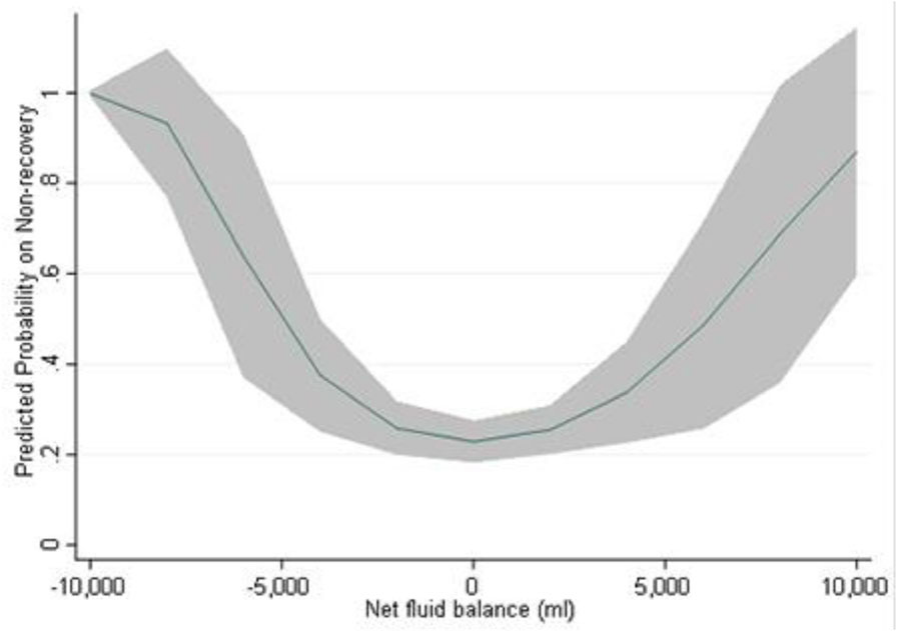
Association between cumulative fluid balance in 48 hours after AKI and risk of AKI non-recovery

### P283 Rhabdomyolysis and acute kidney injury: incidence and relationship to creatine kinase

#### TM Lee^1^, CJ Kirwan^2^, JR Prowle^3^

##### ^1^Barts Health NHS Trust, Adult Critical Care Unit, London, United Kingdom; ^2^Barts and The London School of Medicine and Dentistry, Queen Mary University of London, Adult Critical Care Unit, London, United Kingdom; ^3^Barts and The London School of Medicine and Dentistry, Queen Mary University of London, London, United Kingdom

**Introduction:** Rhabdomyolysis (RM) is likely if serum Creatine Kinase (CK)>5000U/L. RM can cause Acute Kidney Injury (AKI), however in critically ill patients RM and AKI are often multifactorial. Limiting myglobin nephrotoxicity is the goal of intervention. Our ICU has a high urine output AKI prevention protocol for patients with a CK>5000. We review the incidence of RM, initiation of protocol, and its association with AKI.

**Methods:** Retrospective audit of critically ill patients with biochemical evidence of significant RM (CK>5000), over a 6 month period. CK was measured daily. The incidence of AKI by KDIGO criteria, and adherence to RM treatment protocol was recorded.

**Results:** 84 patients had at least 1 CK>5000. 7 were excluded as they were palliated. 21 (28%) had CK>5000 for only 1 day (R1) and 53 (72%) for 2 or more days (R2) (Table 1). Most were Trauma patients (47%). AKI was present in 2/21 in the R1 group with 1 requiring CRRT. The other was not treated on RM protocol. Both had clear additional reasons to RM for developing AKI. The incidence of AKI in the R2 group was significantly higher 26/53 (49%) (p<0.001). 15 were treated on RM protocol. 11 received CRRT. In the 27 who did not develop AKI, 3 required CRRT for pre-existing CKD and 1 was not treated on RM protocol. 31/74 patients had CK>10000 (Table 2). All were in R2. 5/8 (63%) of those with an admission CK>10000 had AKI 2 or 3. All 12 (15%) patients who required CRRT for AKI associated with RM were at risk for AKI regardless of initial CK: vascular surgery (4/12), multi-organ dysfunction (7/12), and/or pre-existing renal disease (3/12).

**Conclusions:** Raised CK is common in ICU but its cause is multi factorial thus an isolated measure >5000 does not require immediate high output treatment for RM AKI. AKI is more common in patients who have more than 1 CK>5000 on 2 sequential days or those whose first CK was >10000 as RM may be contributing. A single CK>5000 in patients with a clear reason to develop RM should also start treatment.

**Table 1 (abstract P283).** Patients with CK>5000 in ACCU

	R1 No AKI (n=19)	R1 with AKI 1-3 (n=2)	R2 No AKI (n=27)	R2 with AKI 1-3 (n=26)
Male	14 (74%)	2 (100%)	21 (78%)	20 (77%)
Trauma	8 (42%)	0	17 (63%)	8 (31%)
Emergency Surgery	1 (5%)	2 (100%)	5 (19%)	4 (15%)
Peak CK (median, range)	6292 (5018 - 9725)	8156 (7559 - 8754)	10232 (6039 - 64750)	12266 (5216 - 94686)
Treated on protocol	5 (26%)	0	23 (85%)	15 (58%)
CRRT	0	1 (CKD 2)	3 (11%)	8 (31%)
Mortality	0	0	1 (4%) CRRT	2 (12%) 2CRRT

**Table 2 (abstract P283).** Patients with CK>10000, their preceding CK measurement(s) and AKI

	Admission CK >10000	Preceding CK <5000	Preceding CK 5000-9999. 1 Day	Preceding CK 5000-9999. ≥2 Days
No AKI (n=15)	3	5	4	3
AKI 1 (n=6)	0	4	1	1
AKI 2 (n=5)	1	2	2	0
AKI 3 (n=5)	4	0	1	0
Total	8	11	8	4

### P284 Surgical outcomes of end-stage kidney disease patients who underwent major surgery

#### P Petchmak^1^, Y Wongmahisorn^1^, K Trongtrakul^2^

##### ^1^Faculty of Medicine Vajira Hospital, Surgery, Bangkok, Thailand; ^2^Faculty of Medicine Vajira Hospital, Internal Medicine, Bangkok, Thailand

**Introduction:** End-stage kidney disease (ESKD) is a major public health problem worldwide, including in Thailand. Patients with ESKD are at significant risk for developing complications during the perioperative period. This study aimed to evaluate ESKD patients admitted to the surgical intensive care unit (SICU) after major surgical procedures and their association with mortality risk at Faculty of Medicine Vajira Hospital, Navamindradhiraj University, Bangkok, Thailand.

**Methods:** A matched cohort retrospective study was conducted of 122 patients who underwent major surgical procedures and who were subsequently admitted to surgical ICU from 2013 to 2016. Sixty-one ESKD patients who requiring long-term dialysis (hemodialysis or peritoneal dialysis) were compared with 61 non-ESKD patients who were matched for sex, age interval, type of operation, and year of procedure. Risk of ICU admission and hospital mortality were compared between the two groups.

**Results:** The clinical characteristics of the ESKD and non-ESKD groups were similar, including sex, age, and underlying disease. The most prevalent co-morbidity during the ICU admission of all patients was severe sepsis/septic shock (22%). The ICU mortality rates were significantly higher in patients with ESKD than non-ESKD (23% versus 5%, P-value 0.004), as were hospital mortality rates (34% versus 17%, P-value 0.001). In addition, patients with ESKD had longer ICU and hospital length of stay than those without ESKD (12 days versus 5.4 days, P-value 0.32, and 42.4 days versus 27.1 days, P-value 0.41, respectively).

**Conclusions:** Patients with pre-existing ESKD requiring long-term dialysis who had major surgery with subsequent intensive admission had a greater risk of mortality than patients without E SKD. With careful monitoring during the perioperative period, major surgical procedures can safely be performed on patients with end-stage kidney disease.

### P285 Perioperative risk factors for acute kidney injury after open aortic aneurysm repair

#### F Pedrosa^1^, S Soares^1^, C Candeias^2^, JM Ribeiro^2^

##### ^1^Centro Hospitalar Universitário Lisboa Norte, Anesthesiology Department, Lisboa, Portugal; ^2^Centro Hospitalar Universitário Lisboa Norte, Intensive Care Medicine Department, Lisboa, Portugal

**Introduction:** Occurrence of acute kidney injury (AKI) in patients submitted to open aortic aneurysm repair (OAAR) is frequent and generally considered to be associated with conventional risk factors. The aim of this study is to characterise this surgical subgroup of patients and identify perioperative risk factors for AKI.

**Methods:** A retrospective non-interventional study was conducted, in a 22-bed level III ICU, tertiary university-affiliated urban referral hospital, including all patients admitted to the ICU after OAAR, from 2015 to 2017. A protocol-driven collection of data from medical records was taken, including the pre and peri-operative periods until 24h after ICU admission. Statistical analysis was performed using SPSS IBM software version 24. A level of significance was considered for a p-value below 0.05.

**Results:** Eighty-eight patients were included in this analysis. Sixty-six percent of patients developed post-operative AKI. Mean age was 70.9 years and the majority of patients were male. Most patients had a history of smoking (66%) and arterial hypertension (83%), but fewer patients had a diagnosis of chronic renal disease (18%), diabetes (13%) or peripheral artery disease (22%). Amongst the variables studied, the following reached a level of statistical significance: emergent surgery (OR=6.08), need for vasopressor therapy (OR=6.47), SAPS II value (OR=1.06), suprarenal aortic cross-clamping duration (OR=1.06), need for blood transfusion during the procedure (OR=1.46), serum lactate level (OR=1.04) and pre-operative glomerular filtration rate (OR=0.95) were associated with the development of postoperative AKI.

**Conclusions:** This study revealed that perioperative factors have a role in risk stratification of AKI in this population. An association between AKI and conventional risk factors like diabetes and hypertension was not found. Identifying additional risk factors might help on establishing better strategies to prevent AKI in these patients and start early treatment strategies.

### P286 Acute kidney injury in critically ill cancer patients

#### N Seylanova^1^, J Zhang^2^, S Crichton^3^, M Ostermann^4^

##### ^1^Sechenov First Moscow State Medical University, Department of Pathophysiology, Moscow, Russia; ^2^Zhongnan Hospital of Wuhan University, Department of Critical Care Medicine, Wuhan, China; ^3^University College London, Medical Research Council Clinical Trials Unit, London, United Kingdom; ^4^Guy’s and St Thomas’ NHS Foundation Trust, Department of Critical Care, London, United Kingdom

**Introduction:** Acute kidney injury (AKI) is common in critically ill patients and associated with increased mortality. The prognosis of AKI is affected by the underlying acute illness and comorbidities. Our aims were to explore the risk factors for AKI in cancer patients admitted to the Intensive Care Unit (ICU) and to investigate whether AKI was associated with ICU and 28-day mortality.

**Methods:** We identified all patients with a haematological malignancy (HM) or solid tumour (ST) who required an emergency admission to ICU in a tertiary care centre between January 2004 and July 2012. AKI was defined according to the serum creatinine criteria of the KDIGO classification. We differentiated between AKI on admission to ICU versus AKI that developed after 24 hours in ICU.

**Results:** 430 patients were included (94 patients with HM, 336 patients with ST). The prevalence of AKI in HM patients was 82% (76% AKI on admission; 6% during stay in ICU) and 62% in ST patients (50% on admission, 12% during stay) (Table 1). ICU and 28-day survival were 67% and 52% in patients with HM, and 78% and 68%, respectively, in the ST cohort. Multivariable analysis showed a borderline association between severe AKI and ICU survival (p=0.051) in HM patients. The odds of survival were significantly lower in those who developed severe AKI during ICU stay compared to patients without AKI (OR=0.029, 95% CI 0.001-0.611). In ST patients, AKI was a risk factor for ICU and 28-day mortality. Independent risk factors for developing AKI during stay in the ICU were APACHE II (OR=1.082, 95% CI 1.010-1.158; p=0.024) and SOFA scores (OR=1.242, 95% CI 1.051-1.468; p=0.011).

**Conclusions:** AKI is common in cancer patients admitted to the ICU. In HM and ST patients, the development of AKI during stay in the ICU was independently associated with higher ICU mortality, and a higher 28-day mortality in the ST cohort. More work is necessary to identify strategies to prevent the development of AKI in high risk cancer patients.

**Table 1 (abstract P286).** Demographics

Parameter	ST patients (n=336)	HM patients (n=94)	P-value
Age, median (IQR)	65 (56-73)	57 (42-64)	<0,001
Male gender, n (%)	202 (60)	62 (66)	0,304
Sepsis on admission, n (%)	233 (69)	77 (82)	0,018
AKI on admission, n (%)	167 (50)	71 (76)	<0,001
AKI during stay, n (%)	42 (13)	6 (6)	
Inotropes on admission, n (%)	74 (22)	36 (38)	0,001
SOFA score on admission, median (IQR)	4 (3-6)	8 (6-10)	<0,001

### P287 Does the length of targeted temperature management after out-of-hospital cardiac arrest have an impact on acute kidney injury?

#### K Strand^1^, E Søreide^1^, H Kirkegaard^2^, A Grejs^2^, CH Duez^2^, AN Jeppesen^2^, FS Taccone^3^, M Skrifvars^4^

##### ^1^Stavanger University Hospital, Dept. Anesthesiology and Intensive Care, Stavanger, Norway; ^2^Aarhus University Hospital, Dept. Anesthesiology and Intensive Care Medicine, Aarhus, Denmark; ^3^Erasme Hospital, Dept. of Intensive Care, Brussels, Belgium; ^4^Helsinki University Hospital, Dept. of Emergency Care and Services, Helsinki, Finland

**Introduction:** Acute kidney injury (AKI) occurs in more than 40% of successfully resuscitated out-of-hospital cardiac arrest patients treated with targeted temperature management (TTM) [1]. The effect of the duration of cooling on AKI has not been well studied. In this post-hoc analysis of the TTH48 randomized controlled trial that compared 24 vs 48-hours of TTM (33°C) after cardiac arrest [2], we studied the impact of TTM length on the development of AKI.

**Methods:** We analyzed out-of-hospital cardiac arrest patients included in the TTH48 trial for the development of AKI during ICU stay with a follow-up time of maximum 7 days. Daily creatinine and urine output were used to categorize AKI according to the KDIGO guidelines. Main outcome was the development of AKI stage 1-3 during the first 7 days of the ICU stay. The impact of duration of TTM on creatinine values was analyzed with a mixed linear model

**Results:** Out of 249 patients included in the analysis, 159 patients (45.5%) developed AKI during ICU stay. The duration of TTM did not affect the incidence of AKI as 78 (44.3%) patients in the 24-hour group developed AKI versus 81 (46.8%) in the 48-hour group, p=0.64. Among the patients with AKI, the length of TTM did not affect the time to development of AKI. Time to AKI was 1.5 (1.3-1.7) days in the 24-hour group and 1.8 (1.5-2.1) days in the 48-hour group, p=0.66. Cumulative number of patients with AKI during ICU stay is shown in Fig. 1. Duration of TTM had a significant impact on the development of creatinine values during the first 7 days in the ICU, p<0.05. This was primarily driven by an increase in creatinine during rewarming on day 2 for the 24-hour and day 3 for the 48-hour group (Fig 2).

**Conclusions:** In a trial of 24 vs 48 hours of TTM after out-of-hospital cardiac arrest, the length of TTM did not affect the incidence of AKI.

**References**

1. Sandroni C et al. Minerva Anestesiologica. 82:989-999, 2016

2. Kirkegaard H et al. JAMA. 318:341-35, 2017

**Fig. 1 (abstract P287).**
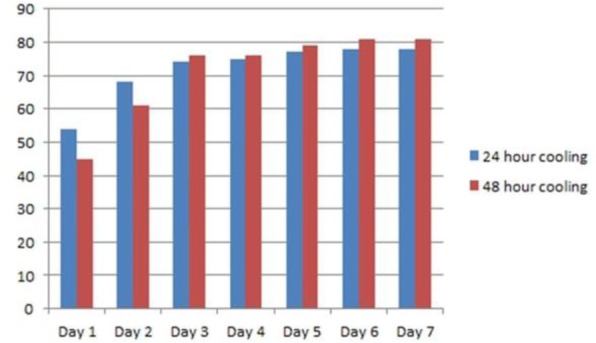
Cumulative number of patients with AKI in the two cooling groups

**Fig. 2 (abstract P287).**
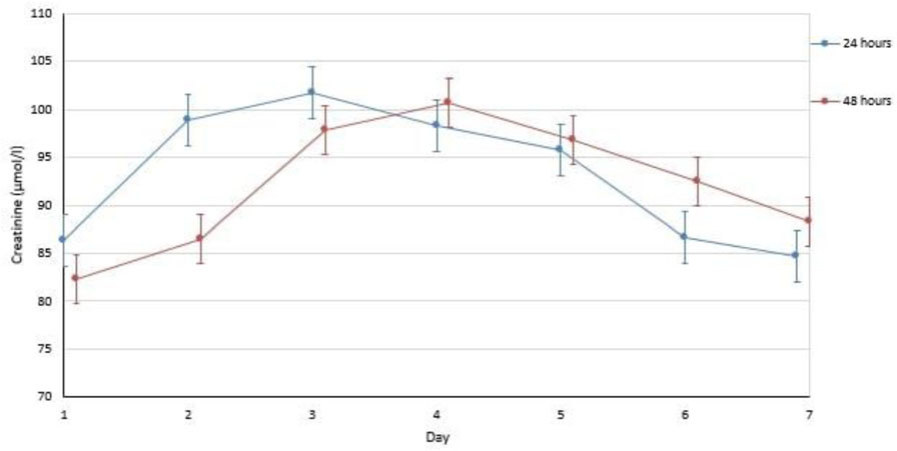
Creatinine over time

### P288 Renal follow up post ECMO

#### G Hutson^1^, R Wright^1^, K Lee^1^, G Vlachopanos^2^, J Kusic^3^, S Crichton^4^, E Ahmadnia^1^, M Ostermann^5^

##### ^1^Guy´s & St Thomas´ Hospital, London, United Kingdom; ^2^Rethymno General Hospital, Rethymno, Greece; ^3^Department of Nephrology, Belgrade, Serbia; ^4^Clinical Trials Unit UCL, London, United Kingdom; ^5^Guy´s & St Thomas´ Hospital, Dept of Critical Care & Nephrology, London, United Kingdom

**Introduction:** Acute kidney injury (AKI) is associated with short- and long-term complications, including chronic kidney disease (CKD). AKI is common in patients receiving extracorporeal membrane oxygenation (ECMO). Our objective was to determine long-term renal function in ECMO survivors.

**Methods:** We retrospectively analysed the data of all patients who were admitted to an adult tertiary care ECMO centre in the UK between 2010 – 2016 and left hospital alive. Using routinely available data, we collected demographics, baseline serum creatinine, presence of AKI during ICU admission, and renal function at hospital discharge, 6 months and 1 year, including treatment with renal replacement therapy (RRT).

**Results:** 350 patients had ECMO between 2010-2016 of whom 268 (78%) had AKI during stay in ICU. 262 patients (75%) left hospital alive. At 6 months, 237 patients (68%) were still alive (6-month survival status was unknown for 14 patients). Among patients alive at 6 months, only 35% had a serum creatinine result available, and at 12 months, only 21% had a creatinine result available. (Table 1). At 6 months, RRT status was known for 167 patients of whom 10 (6%) were receiving regular RRT. At 12 months, RRT was known for 65 patients of whom one (2%) was on RRT. In patients with a creatinine result available at 6 months, the values at 6 months were higher than at hospital discharge and the correlation between both values was low (r=0.11, p=0.331). Conversely, in 36 patients with data at 6 and 12 months, there was a strong correlation between the measures (r=0.75, p<0.001).

**Conclusions:** Renal follow up of ECMO survivors is not very common and more awareness is necessary.

**Table 1 (abstract P288).** Renal follow up data in ECMO survivors

	Number of patients with data	Median creatinine value	Interquartile range
Serum creatinine [micromol/L]			
at hospital discharge	261	72	49-148
at 6 months	82	83	63-106
at 12 months	50	80	63-101

### P289 Longer term renal function post ECMO

#### R Wright^1^, G Hutson^1^, K Lee^1^, E Ahmadnia^2^, M Ostermann^1^

##### ^1^Guy´s and St Thomas´ NHS Foundation Trust, London, United Kingdom; ^2^Guy´s and St Thomas´ NHS Foundation Trust, Critical Care, London, United Kingdom

**Introduction:** Patients receiving extracorporeal membrane oxygenation (ECMO) support often have concurrent acute kidney injury (AKI). Previous research has described short-term outcomes in ECMO patients [1], but there are no published data on longer-term renal outcomes in adult patients. The purpose of this study was to assess longer-term trends in serum creatinine in this cohort.

**Methods:** A retrospective study was conducted of all patients admitted to an adult regional referral centre for ECMO at a UK university hospital between 2010 and 2016. Those who survived for >12 months were included. Demographics, baseline serum creatinine, presence of AKI during ICU admission, and serum creatinine at hospital discharge were determined. Serum creatinine and dependence on renal replacement therapy (RRT) were assessed at 6 and 12 months post ECMO.

**Results:** 13 patients had a complete (or near-complete) data-set available. The mean age was 45.5 years, 38% of whom were male. 8/13 had AKI during their critical care admission. None were dependent on RRT at 6 or 12 months post ECMO. Most patients had lower serum creatinine results at hospital discharge compared to their pre-hospitalisation baseline, but creatinine concentrations at 6 and 12 months post ECMO tended to be higher than at hospital discharge (Figure 1).

**Conclusions:** In this cohort of ECMO patients who were discharged from hospital alive, serum creatinine tended to be lower at hospital discharge compared to baseline and rose again in the following 12 months. Decreased creatinine production due to deconditioning and muscle wasting may offer a biological rationale for the lower creatinine results at hospital discharge [2]. Therefore, caution should be exercised in the use of serum creatinine at hospital discharge to assess renal dysfunction - further research is warranted.

**References**

[1] Antonucci E et al. Artificial Organs 40:746-54, 2016.

[2] Forni LG et al. Intensive Care Med 43:855–866, 2017.

**Fig. 1 (abstract P289).**
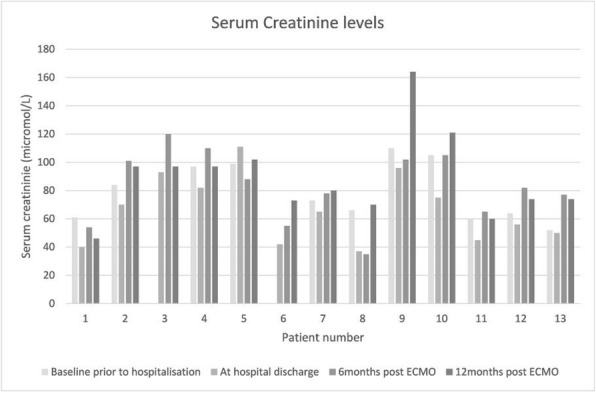
Serial creatinine results pre-and post ECMO

### P290A Epidemiology of acute kidney injury in ICU patients: an observational one-year single-center study

#### S Soundoulounaki^1^, D Marouli^1^, A Spartinou^2^, E Pediaditis^1^, I Papakitsou^1^, N Xirouhaki^1^, D Georgopoulos^1^

##### ^1^University Hospital Heraklion, Department of Intensive Care, Heraklion, Greece; ^2^University Hospital Heraklion, Department of Anesthesiology, Heraklion, Greece

**Introduction:** AKI complicates more than half of ICU admissions [1,2] and is associated with development of chronic kidney disease (CKD), need for renal replacement therapy (RRT) and increased mortality [3]. We prospectively evaluated all ICU admissions during a one-year period in order to determine incidence, etiology and timing of AKI as well relevant clinical outcomes.

**Methods:** Prospective observational study of all patients admitted from Jan to Dec 2017 to a multidisciplinary ICU in Greece. Patients with end-stage renal disease and anticipated ICU stay less than 24 hrs were excluded. AKI diagnosis and classification was based on KDIGO criteria [4]. Lowest creatinine level within 3 months before admission or first creatinine after ICU admission served as reference. Outcomes measured at ICU and hospital discharge included mortality, serum creatinine, estimated glomerular filtration rate (eGFR) and need for RRT.

**Results:** 355 patients with a median SAPS II score of 33 were included. 219/355 (61.7%) were diagnosed with AKI, 162 on admission and 57 during ICU stay. 46.2% were classified as AKI KDIGO stage 1, while 14% and 39.8% developed stage 2 and stage 3 respectively. On admission AKI patients had a higher incidence of septic shock, a higher SOFA score (9.1 vs 6.6) and a lower eGFR (59±40 vs 104±43 ml/min) compared to non-AKI patients. 76 AKI patients (34.7%) received RRT. AKI was associated with longer ICU stay (13 vs 6 days), significantly higher ICU and hospital mortality (OR: 5.68 and 5.97 respectively) and lower eGFR at hospital discharge (69±46 vs 99±57 ml/min).

**Conclusions:** AKI occurred in more than half of ICU admissions, being associated with increased ICU and hospital mortality as well as worse kidney function at hospital discharge.

**References**

1. Hoste E et al. ICM; 41: 1411-23, 2015

2. Piccini P et al. Minerva Anestesiol; 77: 1072-83, 2011

3. Chawla L et al. NEJM; 371: 58-66, 2014

4. Acute Kidney Injury Work Group: Kidney Intern; 2: 1-138, 2012

### P290B Electronic AKI alert implementation, precision and accuracy

#### M Cuartero^1^, L Dono^1^, T Bakonyi^1^, S Tahir^2^, T Christodoulopoulou^2^

##### ^1^St Mary´s Hospital. Imperial College Healthcare NHS Trust, Intensive Care Department, London, United Kingdom; ^2^Charing Cross Hospital. Imperial College Healthcare NHS Trust, Intensive Care Department, London, United Kingdom

**Introduction:** AKI has a high prevalence in ICU. The aim of this study is to audit the compliance of AKI NICE guidelines [1], and assess the precision and accuracy of National Health Service England electronic AKI alert (e-alert) [2]

**Methods:** We conducted a prospective audit against the latest NICE and KDIGO guidelines in two ICU within Imperial College Healthcare NHS Trust. Data was collected from 01/05/18 to 15/06/18. We looked at AKI risk factors, e-alert and actual AKI diagnoses by ICU team. E-alert compares actual serum creatinine during admission with patients baseline

**Results:** The sample included 104 patients, mean age 62-15 years, length of ICU stay 8.5-10.7 days. 56 were male. 14.4% required level 1 care, 49.0% level 2 and 36.5% level 3 care (defined by degree of organ support). 34.6% were admitted from Emergency, and 38.5% from theatres/ recovery. 39 patients (37.5%) had previous chronic kidney disease (CKD); 22 of 39 (56%) were CKD 1 or 2. AKI incidence was 39.4% (41 patients): 20 patients had AKIN 1, 8 AKIN 2 and 13 AKIN 3 (Table 1). 12 patients required renal replacement during ICU stay. Eight patients were referred to nephro-urology: 6 for specific treatment, 1 hemodialysis and 1 for nephrostomy. The incidence of AKI recovery during ICU admission was 80.5%. AKI alert was positive for 34 of 41 (83%) patients. It showed a sensitivity 73.1% (95% CI 58.1%-84.3%), specificity of 93.7% (95CI 84.8%-97.5%), likelihood ratio +11.5 (95%CI 4.4–30) to diagnose AKI. However, only 21 of 41 patients were diagnosed by ICU medical team: sensitivity 51.2% (95%CI 36.5-65.6%) and specificity 100% (95% CI 94.3% - 100%) (Fig 1).

**Conclusions:** Although AKI alert does not include urine output criterion or AKI risk factors, it remains a helpful tool to point out patients with AKI. Education and diagnostic algorithms are still needed to early diagnose and treat AKI patients.

**References**

1. NICE guideline for AKI: prevention, detection and management

2. NHS England AKI alert: NHS/PSA7D/2014/010

**Table 1 (abstract P290B).** Population characteristics and risk factors for AKI

Total n=104	No AKI	AKIN 1	AKIN 2	AKIN 3
N	63 (60.6%)	20 (19.2%)	8 (7.7%)	8 (7.7%)
CKD	12 (19%)*	8 (40%)	2 (25%)	8 (61.5%)
Medical admission	25 (39.7%)	8 (40%)	7 (87.5%)	6 (46.1%)
Surgical admission	38 (60.3%)	12 (60%)	1 (12.5%)	7 (54.8%)
Sepsis	10 (15.8%)**	8 (40%)	4 (50%)	5 (38.4%)
Hypotension	13 (20.6%)***	5 (25%)	6 (75%)	7 (54.8%)
Nephrotoxics	12 (19%)	10 (50%)	2 (25%)	4 (30.8%)

Values expressed as % per column. Abbreviations in alphabetical order: AKI acute kidney injury; AKIN acute kidney injury network definition; CKD chronic kidney disease. There were statistical differences between subgroups with and without AKI for the subgroups of patients with previous CKD (p = 0.010*), sepsis at admission (p = 0.031**), hypotension (p= 0.003***)

**Fig. 1 (abstract P290B).**
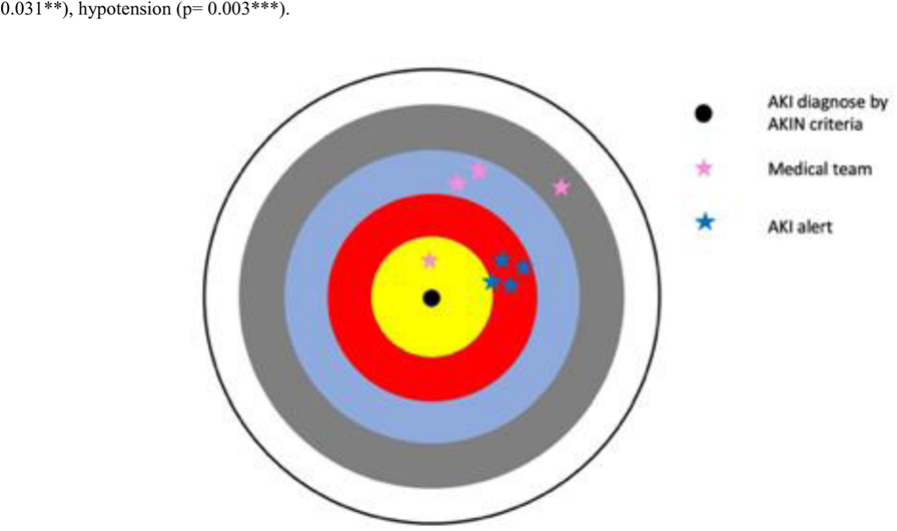
Target comparing accuracy and precision of AKI alert and actual AKI diagnoses

### P291 Influence of severity of illness on urinary neutrophil gelatinase-associated lipocalin in critically ill patients: a prospective observational study

#### C Mitaka, C Ishibashi, I Kawagoe, D Satoh, E Inada

##### Untendo University, Anesthesiology and Pain Medicine, Tokyo, Japan

**Introduction:** Neutrophil gelatinase-associated lipocalin (NGAL) is a diagnostic marker for acute kidney injury (AKI). NGAL expression is highly induced not only in kidney injury, but also in epithelial inflammation of intestine, bacterial infection, and cancer. However, the relationship between uNGAL and severity of critically ill patients has not been well understood. The purpose of this study was to elucidate whether uNGAL is associated with severity of illness and organ failure in critically ill patients.

**Methods:** We prospectively enrolled patients with sepsis (n=34) and patients who underwent esophagectomy with gastric reconstruction for esophageal cancer (n=39). Sepsis was defined according to Sepsis-3. uNGAL levels were measured on ICU day 1, 2, 3, 4 and 7. uNGAL levels and AKI rate in patients with sepsis were compared with those in patients who underwent esophagectomy. AKI was defined according to KDIGO. Acute physiology and chronic health evaluation (APACHE) II score and sequential organ failure assessment (SOFA) score were calculated.

**Results:** Median uNGAL level (413 ng/mg creatinine) was significantly higher in patients with sepsis than that (26 ng/mg creatinine) in patients who underwent esophagectomy on day 1. Median APACHE II score and median SOFA score in patients with sepsis were significantly higher than those in patients who underwent esophagectomy. Four patients with sepsis developed AKI, and 3 out of them underwent continuous renal replacement therapy, whereas no patients who underwent esophagectomy developed AKI. uNGAL levels were positively correlated with APACHE II score and SOFA score in patients with sepsis. uNGAL levels were remarkably elevated (> 4000 ng/mg creatinine) in urinary tract infection (n=3), loops enteritis (n=1), and obstructive jaundice due to cholangiocarcinoma (n=1).

**Conclusions:** These findings suggest that uNGAL level is associated with severity of illness and organ failure in patients with sepsis. uNGAL levels might be influenced by severity of illness and inflammation.

### P292 “FARIUS”-A structured training concept for kidney sonography in a follow-up examination

#### S Kloesel

##### GPR, Department of Anesthesiology, Intensive-Care Medicine and Pain Therapy, Ruesselsheim, Germany

**Introduction:** Structured training concepts are essential for a high-quality medical education [1]. Small groups and hands-on lessons are the basis of these concepts. Can the structured ultrasound (US) training concept “FARIUS” (focused on acute renal injury with ultrasound) enable high-quality examination results even for a longer period after completion of the course?

**Methods:** From 2015-2017, we educated 60 physicians with “FARIUS” in kidney US (ethics: FF108/2014). In addition to theoretical knowledge for acute kidney failure and US technique, a variety of US examinations were conducted under supervision during the course. In addition, over 100 US images and findings were demonstrated and discussed. To assess the quality of the course 15 US renal images had to be evaluated in “post-renal obstruction” (p-ro) or “no p-ro”. The rate of correctness (RoC_Farius_) was determined. In 2018 we, once again, contacted the students to attend a web-based online “follow-up”. This online survey was created with “Google Formular”. 15 new and unknown US images were presented and rated in “p-ro” or “no p-ro” (RoC_FUP_). Data collections, calculation of the median and graphical representation were carried out with EXCEL 2013™. The CHI^2^-test (SPSS13™) was applied to compare the groups, significance level p <0.05.

**Results:** FARIUS-Results: Participant (n) = 60. RoC_FARIUS_: 90.5% (796 out of 880), FOLLOW-UP-Results: n = 28. Frametime (median): 15 months [6-35]. RoC_FUP_: 81.4% (342/420). The CHI^2^ group comparison RoC showed a significant difference (p <0.05).

**Conclusions:** Although the “follow-up” shows a significant decline in RoC, the Follow-up value (81,4%) is still excellent. Hence the structured ultrasound training concept “FARIUS” enables participants that completed the course over a year ago, to still carry out an assessment of kidney US images to a high quality standard.

**Reference**

1. Harden RM et al. BMJ. 1975;1:447–51.

### P293 Renal resistive index changes in a murin sepsis model

#### B Deniau^1^, P Kounde^1^, P Bonnin^2^, J Samuel^3^, A Mebazaa^1^, A Blet^1^

##### ^1^GH Lariboisière Saint Louis, Anesthesiology and Intensive care, Paris, France; ^2^Lariboisière Hospital, Explorations fonctionnelles, Paris, France; ^3^Lariboisière Hospital, Inserm U942, Paris, France

**Introduction:** Septic-induced kidney injury worsen the patient’s prognosis [1]. Renal resistance index (RRI) is correlated with an increased mortality in septic patients [2]. The aim of this study was to describe the evolution of RRI in a rat sepsis model.

**Methods:** The local ethics committee approved the study (APAFIS#9385-2016113016181432). Sepsis was induced in 3-month-old male rats by caecal ligation and puncture (CLP) [3]. The RRI was assessed before and 24h after CLP by pulse Doppler on the left renal artery (RRI=(peak systolic velocity – end diastolic peak)/ peak systolic velocity) (Fig 1). RRI were compared by a paired Wilcoxon test (R software v.3.3.4). A p value < 0.05 was considered significant.

**Results:** 14 rats were included. 24 hours after sepsis induction, all rats were in septic shock with cardiac dysfunction. The RRI increased after sepsis induction compared to baseline (0.61 ± 0.03 vs 0.66 ± 0.02, p<0.05) and mean renal artery velocity decreased (11.8 ± 1.41 vs 7.7 ± 0.94, p<0.05) (Fig 2). Systolic and diastolic peaks velocity of the renal artery were unchanged.

**Conclusions:** Sepsis induced changes in RRI and mean velocity on the left renal artery whereas no changes in systolic or diastolic velocities were seen. These results are consistent with available clinical datas. The RRI could be an additional tool to assess renal failure in septic rats. Further studies are needed to confirm the validity of this marker during sepsis. Kidney failure is one of the most common organ dysfunction during sepsis. The RRI could be an additional tool in small animals to assess the effects of potential therapeutic targets on renal function induced by sepsis.

**References**

1. Singer M et al. JAMA 315 :801-10;2016

2. Lerolle N et al. Intensive Care Med, 32:1553–1559;2006

3. Rittirsch et al. Nat Protoc, 4:31-36, 2009

**Fig. 1 (abstract P293).**
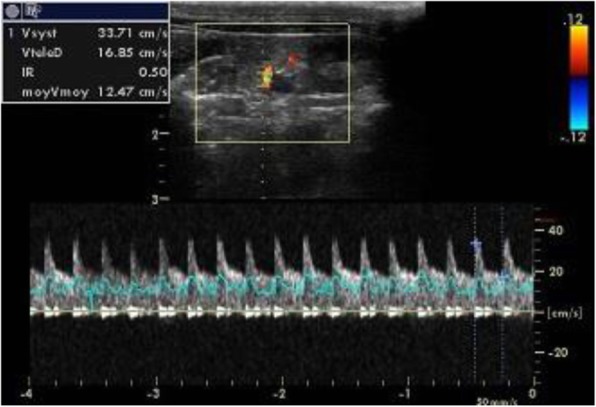
Assessment of RRI by pulse Doppler of the left renal artery in a rat

**Fig. 2 (abstract P293).**
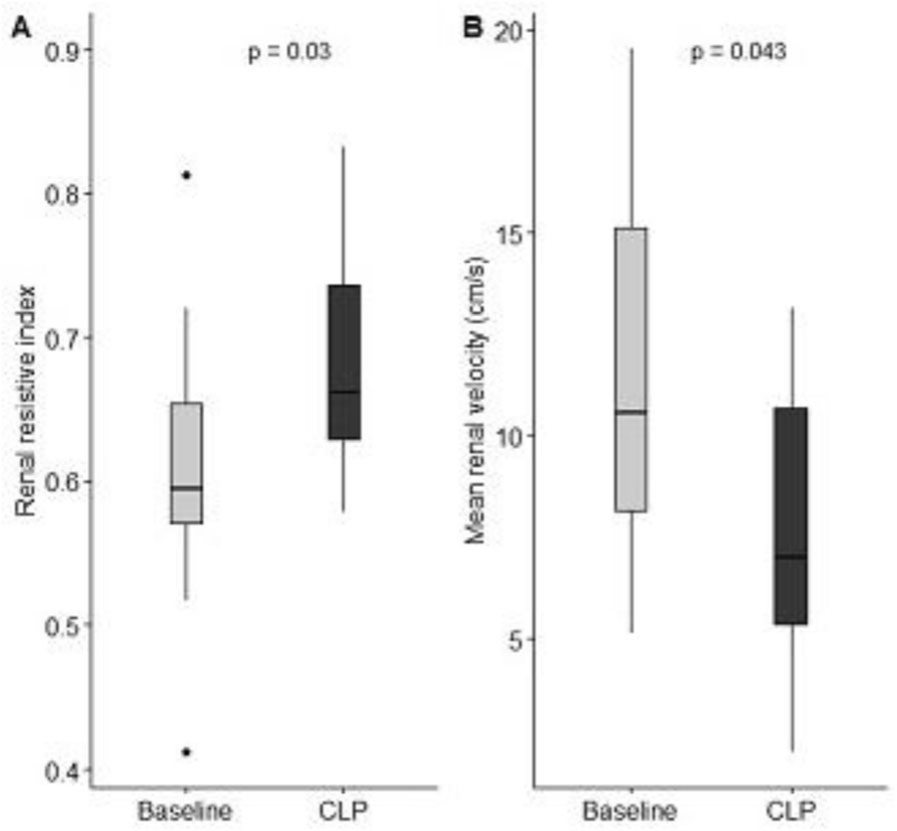
Left artery RRI and mean velocity before and after sepsis induction

### P294 Do simple or sequential renal resistive index (RRI) measures predict renal function evolution in critically ill patients with acute kidney injury (AKI)?

#### P Wiesen, P Damas

##### CHU Liège, Intensive Care, Liège, Belgium

**Introduction:** Renal failure is, in critically ill patients, associated with bad prognosis. It seems to be no benefit to the early use of Renal Replacement Therapy (RRT) [1], but a positive fluid balance (owing to delayed RRT) is associated with bad prognosis. The place of Doppler derived RRI as a potential renal function predictor remains controversial.

**Methods:** In this observational study, 93 single or sequential RRI measurements were obtained in 55 patients meeting at least stage 2 AKI according to KDIGO criteria. Risk factors (diabetes, vascular surgery, cardiac dysfunction, arterial hypertension), SOFA score, presumed etiology of renal failure (hypoperfusion, sepsis, congestion, vascular glomerular dysregulation) and clinical and biological data were recorded. Patients were classified according to their RRI (cut off value 0.70) and their characteristics as well as the ultimate RRT requirement were compared between them.

**Results:** 23 patients present at least one RRI < 0.7 (28 RRI measurements). Among them, no patients required RRT during the 28 following days; except one who died unexpectedly of septic shock. Of the 32 patients who had only RRI > 0.7 (single or sequential measurements), 9 patients finally required RRT. We found no relationship between RRI and creatinine evolution nor between RRI and presumed AKI etiology. Patients with RRI > 0.7 had however significantly more risk factors for kidney injury in particular diabetes (p=0.0003).

**Conclusions:** RRI was not associated with evolution of biological AKI or with AKI etiology, even when sequential measurements were obtained. However, low RRI were associated with the absence of RRT requirement, while high RRI correlated with patient’s risk factors for AKI.

**Reference**

1: Gaudry et al. NEJM 375;2: 122-133, 2016

### P295 Incidence of alkalosis during CRRT on the Nikkiso Aquarius platform when using different anticoagulation strategies

#### C Kirwan, S Lucena-Amaro, S Schwarze, J Prowle

##### Royal London Hospital, Adult Critical Care Unit, London, United Kingdom

**Introduction:** Regional citrate anticoagulation (RCA) has been available on the Nikkiso Aquarius for ~3y. The bicarbonate dose in ACCUSOL 35 Ca2+ containing replacement fluid raises concern that adding di-sodium citrate will cause problematic alkalosis. We evaluate the incidence of alkalosis (pH>7.5/HCO3^-^ >40 mmol/L) in critically ill patients receiving CRRT this way

**Methods:** A retrospective audit. Protocol offers RCA (CVVHF post dilution), CVVHDF & heparin or CVVH with 100% pre-dilution +/- heparin, at pre-set doses of 20, 25 or 35mLs/kg/h. Contraindications to RCA are lactic acidosis (>5mmol/L), acute liver failure & pH>7.5. If pH>7.5 during therapy protocol suggests reducing dose or cease RCA

**Results:** 231 filters in 52 patients (median 2 [1-27]). Median filter life censored for reasons other than clotting was 31h [1-71] with RCA versus non-RCA at 37 v 29h respectively (p=0.047). 34 (65%) patients started with RCA and 15 only used 1 filter. None were stopped due to alkalosis. Of the remaining 216 filters 92 (43%) used RCA; 26 filters were started when the pH was already >7.5, though 11 had a pH<7.5 at the end of treatment. Only 1 was prescribed 20mL/kg/min & 5 were prescribed RCA, which is against protocol. 23 started with pH<7.5 but had a pH>7.5 during treatment; 16 received RCA with: median starting pH 7.45, filter life 22h & delivered dose of 26mL/kg; compared to 7.47, 5h (p=0.02) & 23mL/kg for non-RCA respectively. 7 in the RCA group stopped for alkalosis. Only 6/23 patients had a dose reduction during therapy as the pH rose. Median (range) serum bicarbonate 26.2 [6.7-39.5]. No reported adverse events associated with citrate accumulation / alkalosis

**Conclusions:** RCA on the Nikkiso Aquarius is successful & safe. Alkalosis is important & influenced by improved filter life & increased delivered dose associated with RCA. Alkalosis is often predictable & thus vigilance, individualisation of CRRT prescription & improved protocol compliance is likely to reduce its incidence further

### P296 Cost and haemodynamic model comparison for heparin vs citrate renal replacement therapy

#### R Gould^1^, J Cheong^2^, M Mallick^2^

##### ^1^Leeds Teaching Hospitals, Department of Adult Critical Care, Leeds, United Kingdom; ^2^Hull and East Yorkshire Hospitals, Department of Anaesthesia and Critical Care, Hull, United Kingdom

**Introduction:** Renal Replacement Therapy (RRT) is the focus of new technological development within critical care. There has been a trend towards regional citrate anticoagulation replacing heparin for continuous RRT. Little has been published on the actual monitoring costs associated with a citrate therapy. In addition to reviewing changes in eGFR as a marker of efficiency and the impact of interruptions on patient haemodynamics. Our study aims to identify the monitoring costs of RRT, compare the efficacy of the two systems and explore the relationship between interruptions and inotropic requirements.

**Methods:** Retrospective observational study of 80 patients receiving RRT over two time periods to cover the local change from heparin to citrate. The data collected included type and frequency of blood sampling, biochemical results, hemofiltration parameters and inotrope infusion rates over the first 24 hour period of treatment.

**Results:** A total of 62 patients analysed. Monitoring costs were £45.72 with the heparin group, and £36.73 with citrate (Fig 2). The eGFR improved more with the heparin group (53% vs 48%; p=0.21) (Fig 1). Interruptions of the filter circuit were as expected less with the citrate group (144mins vs 45mins; p=0.17). Finally, inotropic requirements increased following therapy interruptions, more so with patients receiving citrate (7.2% vs 14.5%; p=0.4).

**Conclusions:** Our analysis suggests that using citrate anticoagulation for RRT results in a monitoring cost saving of approximately £9 per 24 hours, alongside the other conferred savings previously reported. Furthermore, results demonstrate the efficacies of both systems are similar in the initial 24 hours, although there is a suggestion that heparin systems improves renal parameters more quickly. Finally, interruptions and ‘filter downtime’ caused an increase in the patient’s inotropic requirements, however results suggestive that this is greater in the citrate group.

**Fig. 1 (abstract P296).**
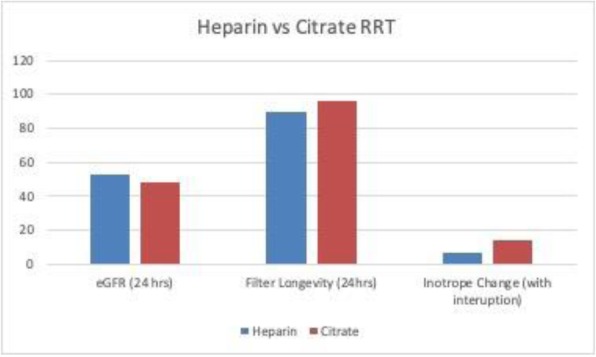
Bar chart comparing eGFR, filter longevity, inotrope changes

**Fig. 2 (abstract P296).**
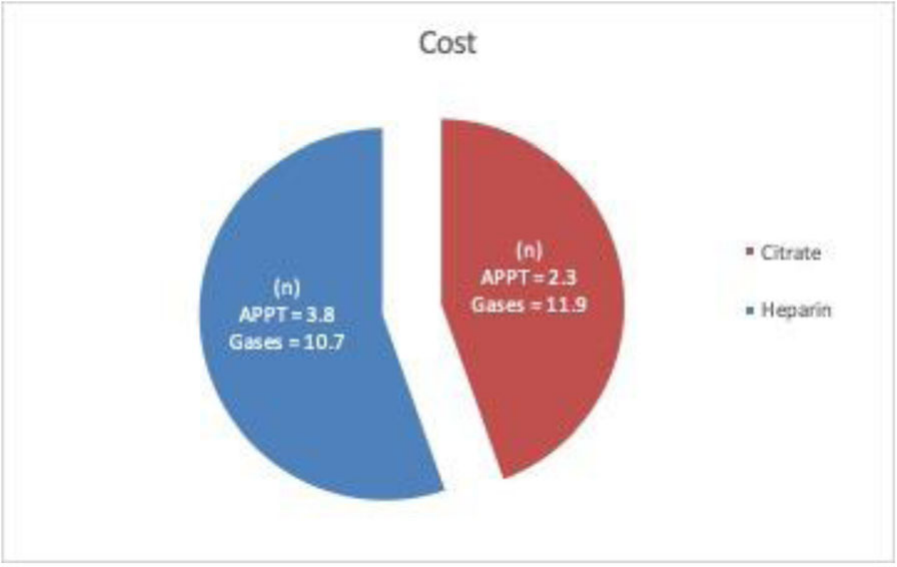
Pie chart of blood tests and costs

### P297 Citrate anticoagulation in pediatric CRRT patients

#### E Sevketoglu^1^, N Akcay^2^, U Kocoglu Barlas^1^, M Talip Petmezci^1^, G Ozcelik^1^, K Boydag Guvenc^1^

##### ^1^University of Health Sciences, Bakirkoy Dr Sadi Konuk Training and Research Center, Istanbul, Turkey; ^2^University of Health Sciences, Bakirkoy Dr Sadi Konuk Training and Research Center, Pediatric Intensive Care Unit, Department of Pediatrics, Istanbul, Turkey

**Introduction:** Citrate is preferred to heparin for anticoagulation in adult continuous renal replacement therapy (CRRT). However, its potential adverse effects and data on use in CRRT in infants and children are limited. We conducted a prospective study on using citrate in CRRT in pediatric patients

**Methods:** Patients who underwent CRRT in our PİCU included to this prospective observational study. Patients with severe hepatic impairment and less than 5 kilograms were excluded. PRISM, OFI, age, sex, diagnosis, RRT form, citrate flow rate, calcium flow rate, blood flow rate, total RRT time, PICU length of stay, number of circuits used, dialysis flow rate, complications were recorded. We used 4% Sodium citrate solution (136 Mmol / L). The prepared calcium solution was set to infuse at 1.7 Mmol/L after the filter. Post filter ionized calcium (PfiCa), patient ionized calcium (PiCa), blood gas, total Ca / PiCa values were taken hourly in the first two hours, if normal twice for every 3 hours, and than every 6 hours. The results were evaluated according to the pre-formed flow chart and the necessary adjustments were made (Table 1,2).

**Results:** Fourteen patients (% 64 M, % 36 F) were included in the analysis. Results are presented as the mean values. The mean age of children at the beginning of the procedure was 4.2 years. During 1728 h CRRT procedure 20 filters were used in total. Also the mean circuit life was 3.6 days. The mean OFI score was 2.9, PRISM score was 19.3. Circuits were started with the default setting for the citrate dose and calcium dose 3 Mmol/L and 1.7 Mmol/L respectively. Demographic characteristics of the study group and the main parameters of the procedure were presented in Fig 1.

**Conclusions:** Regional citrate is a safe and effective anticoagulation method for CRRT in children, when it is applied following a protocol. It significantly prolongs circuit survival time and thereby should increase CRRT efficiency. We did not find any serious adverse effects of regional citrate anticoagulation.

**Table 1 (abstract P297).** Complication follow-up chart

PiCa < 0.8 mmol/L or >1.5 mmol/L	Calcium infusion Increase / decrease
Total Ca > 3 mmol/L	Decrease calcium infusion CL!!
Na^+^ < 130 or >150 mmol/L	Correction with medical treatment
HCO3^-^ > 35mmol/L	Decrease citrate infusion
pH < 7.3 or pH > 7.5	Stop/Decrease citrate infusion
Base Excess < - 5	Stop/Decrease citrate infusion
Total Ca (mmol/L) / PiCa (mmol/lt) > 2.25	CL!! Stop/Decrease citrate infusion, İncrease dialysate rate and blood flow rate
Patient Anion Gap > 8mmol/L	Stop/Decrease citrate infusion
Hypomagnesemia, hypophosphatemia	Correction with medical treatment

PiCa: patient ionized calcium, CL: citrate lock

**Table 2 (abstract P297).** Calcium compensation and citrate dose modification according to laboratory findings

PFiCa mmol/L	citrate infusion rate mmol/L	PiCa mmol/L	calcium infusion rate mmol/L
<0.30	0.2 mmol/L Decrease	<1.0	0.4 mmol/ L İncrease
0.30-0.35	0.1 mmol/L Decrease	1.0-1.10	0.2 mmol /L İncrease
0.35-0.45	Don't touch	1.10-1.30	Don't touch
0.45-0.50	0.1 mmol/L İncrease	1.30-1.35	0.2 mmol /L Decrease
>0.50	0.2 mmol/L İncrease	>1.35	0.4 mmol/ L Decrease

PFiCa: Post filter ionized calcium, PiCa: patient ionized calcium

**Fig. 1 (abstract P297).**
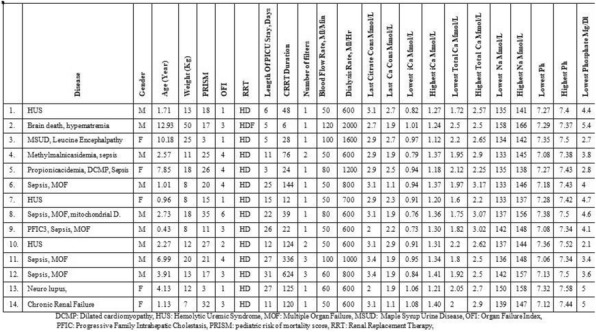
Characteristics of the pediatric patients enrolled in the study

### P298 CRRT after orthotopic liver transplantation

#### G Pavone^1^, G Martucci^1^, S Li Petri^2^, G Panarello^1^, F Tuzzolino^3^, C Piacente^1^, A Papeo^1^, E Bonicolini^1^, C Spina^1^, G Burgio^1^, R Volpes^4^, G Vizzini^4^, S Gruttadauria^2^, A Arcadipane^1^

##### ^1^IRCCS ISMETT - Istituto Mediterraneo per i Trapianti e Terapie ad Alta Specializzazione, Anesthesia and Critical Care Medicine, Palermo, Italy; ^2^IRCCS ISMETT - Istituto Mediterraneo per i Trapianti e Terapie ad Alta Specializzazione, Abdominal surgery and transplantation unit, Palermo, Italy; ^3^IRCCS ISMETT - Istituto Mediterraneo per i Trapianti e Terapie ad Alta Specializzazione, Palermo, Italy; ^4^IRCCS ISMETT - Istituto Mediterraneo per i Trapianti e Terapie ad Alta Specializzazione, Hepatology and Gastroenterology, Palermo, Italy

**Introduction:** In liver transplantation (LT) recipients, a multifactorial acute kidney injury (AKI) has been documented in 5-35% and together with CRRT are significant risk factors for in-hospital mortality. We aim at describing the kidney function and the use of CRRT in the first week after LT and the impact of CRRT on ICU and hospital length of stay (LOS) and mortality with one year follow up.

**Methods:** Retrospective observational study. Explorative analysis of AKI and CRRT use in adult LT recipients during first 7 days post operative in a LT center in southern Italy, from 2006 to 2016. Results are presented as median value and IQR or count and percentage, the association of CRRT and clinical factors was evalutated by logistic regression.

**Results:** 479 conseutive patients underwent LT during the study period with the standard technique: age 55 years (48-61), female n=128 (26.7%), BMI 26 (24-30), MELD 22 (20-23), hepatocarcinoma n=193 (40.3%), surgery duration 6 hours (5-7), intraoperative bypass use n=89 (18.6%), ICU LOS 2 days (1-5), deceased at 1 year n=67 (14%). The MDRD trend is more indicative than creatinine of decline of renal function in the post operative period (Fig 1). CRRT was used in 14.4% (69 pts) and was associated to a greater LOS and mortality (Fig 2). Preoperative bilirubin, BUN and creatinine are among the greatest risk factors for its use (Table 1). Intraoperatively, main risk factors were: the use of vasopressin (OR 25, 95%CI 2.7-228, p<0.01), prolonged hypotension episodes defined as more than 20 min with median arterial pressure <50 mmHg (OR 2.5, 95%CI 1.3-4.8, p<0.01) and transfusions of PRBC or plasma or platelets (p<0.01). Diuresis and CRRT dose in first 7 postoperative days are summarized in Table 2. At 1 year follow up n=6 pts (1.5%) were on hemodialysis.

**Conclusions:** AKI requiring CRRT in after LT is associated with higher mortality and LOS. Identify patients at risk and adopt preventive strategies in the perioperative period is mandatory.

**Table 1 (abstract P298).** See text for description

	CRRT group (n=69 - 14.4%)	NO CRRT group (n=410-85.6%)	p value
Bilirubin pre LT (mg/dl)	5.52 (2.45-15.4)	2.24 (1.1-4.99)	<0.001
BUN pre LT (mg/dl)	64 (28-115)	34 (25-45)	<0.001
Creatinine pre LT (mg/dl)	1.3 (1-2.7)	0.9 (0.8-1.2)	<0.001
MELD	25 (21-33)	22 (19-22)	<0.001
Survival rate at 30/90/360 days	80%/67%/51%	99%/96%/93%	<0.001
HD at 30/90/360 days	40%/22.8%/11.1%	3%/1.6%/1.4%	<0.001
ICU LOS/Hospital LOS	19.45±33/59.48±58.6	11.3±3.5/29.6±23.7	<0.001

**Table 2 (abstract P298).** Diuresis and CRRT dose in first 7 postoperative days

	Diuresis (ml)	Fluid balance (ml)	Dose CRRT (ml/kg/h)
Day 1	1350 (880-1890)	3179 (1258-5965)	26.5 (18-34)
Day 2	1610 (1205-2050)	1096 (194-1900)	30 (22-35)
Day 3	1560 (1130-2120)	420 (-461-1290)	28 (24.5-35)
Day 4	1500 (930-2190)	-324.5 (-1425-477)	28 (24-35)
Day 5	1380 (800-2020)	-672.5 ( -1606-65.5)	28 (25-35)
Day 6	1350 (730-2050)	-837.5 (-1735- -50)	29 (22-35)
Day 7	1400 (700-2020)	-1050 (-1850- -160)	30 (25-35)

**Fig. 1 (abstract P298).**
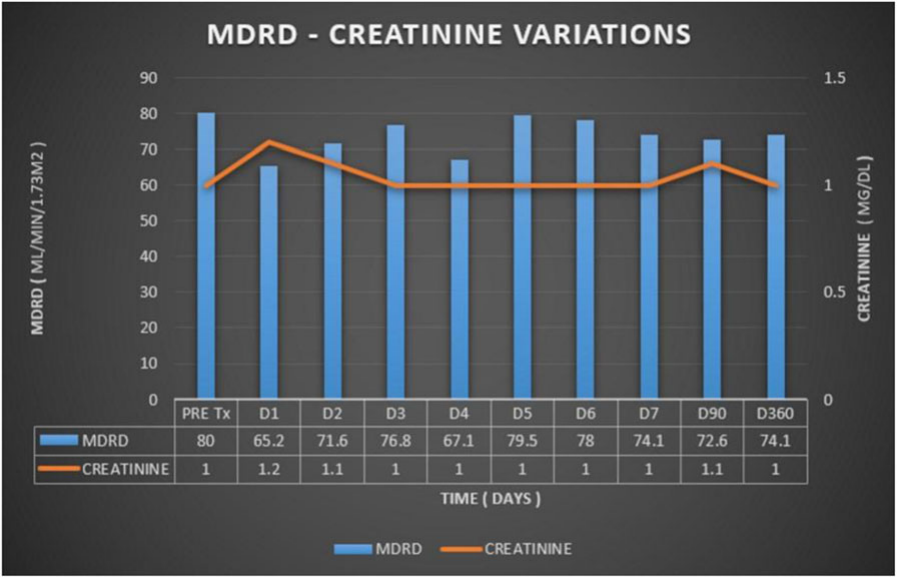
See text for description

**Fig. 2 (abstract P298).**
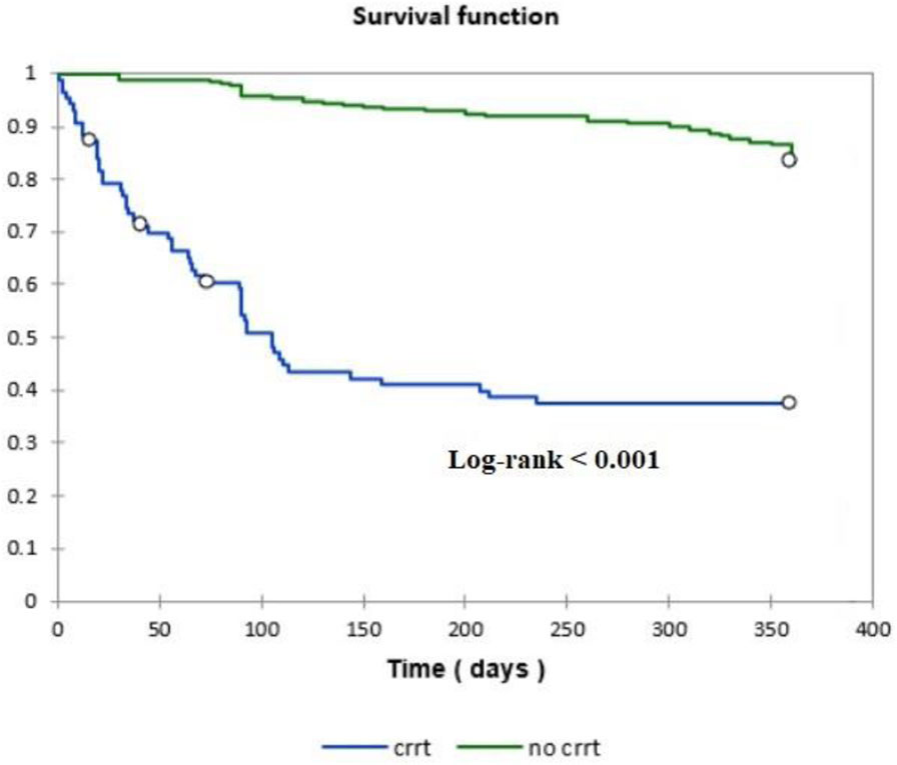
See text for description

### P299 Continuous renal replacement therapy in acute liver failure: new perspectives

#### T Isidoro Duarte^1^, C Vieira^2^, N Germano^1^

##### ^1^Hospital de Curry Cabral, Unidade de Cuidados Intensivos Polivalente 7, Lisboa, Portugal; ^2^Hospital de Santo António dos Capuchos, Serviço de Medicina 2.4, Lisboa, Portugal

**Introduction:** Acute liver failure (ALF) is a life-threatening condition. It activates immune cells response, which promotes the production of proinflammatory cytokines and perpetuates the apoptosis and necrosis cascade, leading to hemodynamic instability. The use of continuous renal replacement therapy (CRRT) using blood purification adsorption devices may have an important role in improving patient outcomes by removing the excessive circulating inflammatory mediators.

**Methods:** A retrospective analysis of five patients with ALF and without concurrent bacterial infection which initiated CRRT with oXiris®. Acid-base balance, lactate levels and vasopressor dose were recorded every 6 hours during the first 24 hours.

**Results:** Five patients (80% male), mean age of 36.4±6.7 years. Admission APACHE score 32.6±5.6. ALF etiologies were primary graft dysfunction (60%), acetaminophen intoxication (20%) and herpes simplex virus type 1 infection (20%). Acid-base balance before CRRT showed pH 7.25±0.07 and HCO3^-^ 13.15±6.15mmol/L. All patients were under vasopressor support with noradrenaline (NA) with a mean dose of 1.9±2.7mcgr/kg/min and blood lactate of 11.2±0.14mmol/L. Mean effluent rate 42.5±3.5mL/kg/h. During the first 24 hours of CRRT there was a rise in the mean pH (7.37±0.03) and HCO3^-^ (23.1±0.28mmol/L) values, a progressive decrease in the vasopressor perfusion dose (0.30 mcgr/kg/min) and blood lactate (2.3±0.0mmol/L) without worsening of the hemodynamic status. Differences in acid-base and blood lactate values before and after 24 hours of CRRT with oXiris® appears to be statistically significative (p-value <0.05). Hospital mortality rate of 40%.

**Conclusions:** The use of adsorbent membranes may play a part in acute liver failure patients by reducing circulating proinflammatory mediators responsible for hemodynamic instability, thus improving survival and outcomes.

### P300 The in vivo experiment of extracorporeal lung and renal assist device

#### N Takahashi^1^, T Nakada^1^, T Sakai^2^, Y Kato^2^, K Moriyama^3^, O Nishida^2^, S Oda^1^

##### ^1^Chiba University Graduate School of Medicine, Department of Emergency and Critical Care Medicine, Chiba, Japan; ^2^Fujita Health University School of Medicine, Department of Anesthesiology and Critical Care Medicine, Aichi, Japan; ^3^Fujita Health University School of Medicine, Laboratory for Immune Response and Regulatory Medicine, Aichi, Japan

**Introduction:** We developed a new CO2 removal system, which has a high efficiency of CO2 removal at a low blood flow. To evaluate this system, we conducted in vivo studies using experimental swine model.

**Methods:** Six anesthetized and mechanically ventilated healthy swine were connected to the new system which is comprised of acid infusion, membrane lung, continuous hemodiafiltration and alkaline infusion. In vivo experiments consist of four protocols of one hour; Baseline= hemodiafiltration only (no O2 gas flow of membrane lung); Membrane lung = “Baseline” plus O2 gas flow of membrane lung; “Acid infusion” = “Membrane lung” plus continuous acid infusion; “Final protocol” = “Acid infusion” plus continuous alkaline infusion. We provided an interval period of one hour between each protocol. We changed the respiratory rate of the mechanical ventilation to maintain PCO2 at 50-55 mmHg during the experiment.

**Results:** The amount of CO2 eliminated by the membrane lung (VCO2ML) significantly increased by 1.6 times in the acid infusion protocol and our final protocol compared to the conventional membrane lung protocol, while there was statistically no significant difference observed in the levels of pH, HCO3-, and base excess between each study protocol. Minute ventilation in the “Final protocol” significantly decreased by 0.5 times compared with the hemodiafiltration only protocol (P <0.0001), the membrane lung (P=0.0006) and acid infusion protocol (P=0.0017).

**Conclusions:** We developed a novel ECCO2R system which efficiently removed CO2 and is easy-to-setup to permit clinical application. This new system significantly reduced minute ventilation, while maintaining acid-base balance within the normal range. Further studies are needed for the clinical application of this easy setup system comprising of the materials typically used in a clinical setting.

### P301 Potential adverse hemodynamic effects of higher intensity continuous renal replacement therapy

#### YP Kelly, SS Mothi, SS Waikar

##### Brigham and Women´s Hospital, Nephrology, Boston, United States

**Introduction:** Higher intensity continuous renal replacement therapy (CRRT) has been studied as a potential therapeutic advance for the treatment of severe acute kidney injury (AKI). We hypothesized that compared to standard intensity CRRT, higher intensity CRRT leads to greater hemodynamic instability due to the unregulated removal of small solutes, including phosphate, amino acids, trace elements and vasopressor medications

**Methods:** Using detailed hemodynamic data recorded during the Acute Renal Failure Trial Network (ATN) trial, we assessed the incidence of hemodynamic instability in those randomized to higher (35 ml/kg/hour) versus lower intensity (20 ml/kg/hour) CRRT treatment.

**Results:** Of 1124 individuals enrolled in the ATN Trial, 711 were managed solely with CRRT (N = 355 higher intensity and 356 lower intensity therapy). 138/356 (38.8%) patients in the low intensity group experienced 403 hypotensive events over 2554 CRRT treatments and 159/355 (44.8%) patients in the high intensity group experienced 537 hypotensive events over 2605 CRRT treatments (p = 0.007). Using a Poisson regression model, the unadjusted odds ratio for hypotension in the high intensity CRRT group compared to the low intensity group was 1.19 (95% confidence limits 1.05 to 1.35; p = 0.007). However, this relationship was no longer significant once ultrafiltration rate, CVP, arterial pH, ionized calcium and glucocorticoid dose were added to the model; with an adjusted odds ratio of 1.33 (95% confidence limits 0.7 to 2.5; p = 0.38). Only arterial pH was significantly independently associated with the risk of hypotensive events (p = 0.01).

**Conclusions:** Higher intensity CRRT is associated with a significantly greater incidence of hypotensive events; however when adjusted for arterial pH, there is no longer a significant relationship between CRRT treatment dose and the risk of hypotension.

### P302 Circulatory effects of CRRT for AN69ST and a polysulfone membrane in patients with septic shock: a retrospective observational study

#### M Shibata, K Miyamoto, S Kato

##### Wakayama Medical University, The Department of Emergency and Critical Care Medicine, Wakayama, Japan

**Introduction:** The acrylonitrile-co-methallyl sulfonate surface-treated (AN69ST) membrane is expected to improve hemodynamics in patients with sepsis by cytokine adsorption. However, the clinical literature on AN69ST membranes is scarce.

**Methods:** This retrospective observational study compared the circulatory effects of continuous renal replacement therapy (CRRT) between patients with AN69ST and polysulfone (PS) membranes. We enrolled 38 patients with septic shock, defined by Sepsis-3 criteria who required CRRT from April 2013 until May 2018. We excluded patients who died within 24 hours after starting CRRT and received a polymyxin B immobilized fiber column direct hemoperfusion, extracorporeal membrane oxygenation, and CRRT using other membranes. The primary outcome was the vasopressor dependency index during the 12 hours after the start of CRRT. The vasopressor dependency index was calculated as (inotropic score)/(mean arterial pressure).

**Results:** Of the 38 patients who were analyzed, 16 underwent CRRT with an AN69ST membrane and 22 with a PS membrane. The median patient age was 68 years, and the median acute physiology and chronic health evaluation II score at ICU admission was 29.5. Intraabdominal infection was more common in the PS group than in the AN69ST group. The vasopressor dependency index decreased significantly during the 12 hours after the start of CRRT in both groups (AN69ST: from 0.50±0.43 to 0.33±0.27 (*P*<0.05), PS: from 0.34±0.30 to 0.21±0.22 (*P*<0.05)). The vasopressor dependency index during the 12 hours did not differ between the two groups (*P*=0.11). The hospital mortality rate did not differ between the groups (10/16 (63%) and 8/22 (36%); *P*=0.19).

**Conclusions:** The vasopressor dependency index decreased significantly after the start of CRRT with both AN69ST and PS membranes in patients with septic shock. The time course of the vasopressor dependency index did not differ between the groups.

### P303 Feasibility of ultrafiltration in patients treated with the ADVOS/HepaWash-device under haemodynamic monitoring with transpulmonary thermodilution (TPTD) and pulse contour analysis (PCA): the HAEMADVOS-III-study

#### W Huber, M Leinfelder, T Lahmer, G Batres-Baires, S Rasch, S Schreiber, R Schmid

##### II. Medizinische Klinik, Station 2/11, Munich, Germany

**Introduction:** Due to the high prevalence of multi-organ failure in critically ill patients, multi-organ support provided by one single device is intriguing. The “advanced organ support” (ADVOS) procedure combines liver support, renal replacement, CO2-elimination and acid-base modulation. Furthermore, ADVOS allows for high volume fluid removal by ultrafiltration.

Objective: The HAEMADVOS-I and –II studies (see ESICM 2018) investigated haemodynamic effects of connection and disconnection of ADVOS. HAEMADVOS-III investigates feasibility and effects of ultrafiltration during ADVOS-therapy in patients with PiCCO-monitoring.

**Methods:** Thermodilution (PiCCO; Pulsion; Germany) was performed immediately before and after connection (T1, T2; prefilled tubings/“acute connection”) to the ADVOS-device and disconnection (T3; T4). Immediately after T1 and before connection to the ADVOS-device the treating physician defined an ultrafiltration goal.

**Results:** 36 ADVOS-treatments in 13 patients; SOFA 12 (8-17). Vasopressors 25/36 (69%), mechanical ventilation 33/36 (92%). Pre-defined ultrafiltration goal and final ultrafiltration correlated (r=0.577; p<0.001) and were not significantly different (1553±905 vs. 1563±1039mL; p=0.860). Noradrenaline dosage was comparable at T3 vs. T1 (429±377 vs. 373±396 micro_g/h; p=0.222). Ultrafiltration rate was lower than 80% of the pre-defined goal in 10/36 (28%) treatments. In ROC-analysis none of the baseline haemodynamic parameters predicted this event. By contrast this event was predicted by decreases in global end-diastolic volume index GEDVI (ROC-AUC 0.737; p=0.048), cardiac index CI (AUC 0.737; p=0.048) and stroke volume index SVI (AUC 0-753; p=0.035) during connection (T2 vs. T1). Changes in CVP, heart rate or MAP were not predictive.

**Conclusions:** Ultrafiltration with ADVOS was well tolerated in the majority of patients. Failure to achieve the preset filtration-goals was best predicted by decreases in GEDVI, CI and SVI after the connection to ADVOS.

### P304 Quality outcome data following the introduction of slow low efficiency daily dialysis (SLEDD) in critical care

#### L O´Connor, D Elliot, H Blackman, C Wilson, S Ahmed

##### Sunderland Royal Hospital, Anaesthesia & Intensive Care Medicine, Sunderland, United Kingdom

**Introduction:** SLEDD is a renal replacement therapy (RRT) that offers the stability of a continuous modality with the efficiency of intermittent haemodialysis(IHD). In 2016, Sunderland Royal Hospital integrated critical care unit (ICCU) transitioned from IHD by the renal staff to SLEDD delivery by the ICCU nurses. During the implementation phase quality indicators were identified to assess the process, organisation & outcome of therapy.

**Methods:** SLEDD episodes were assessed for success in meeting the SLEDD prescription targets, delivery by the critical care team, premature termination of SLEDD, safety & dose (urea reduction ratio – URR). The standard prescription was a low flux filter with blood & dialysate flows of 150 & 300ml/min for 6hrs using a Fresenius 4008 dialysis machine. During the study period standard anticoagulation practice changed from unfractionated heparin (UFH) to citrate containing dialysate (C).

**Results:** Between June & Dec 2017, 173 sessions were delivered to 40 patients, daily for a median of 3 sessions (1-25). 68% of prescriptions were the standard with deviations in therapy duration, & 92% were timely. Anticoagulation was avoided in 10%, 35% received C, 46% UFH & 9% both. SLEDD targets were achieved in 74% cases using UFH & 67% C anticoagulation. Clotting of the circuit occurred in 23% of session using UFH & 37% using C, occurring disproportionately in a few patients. The median dose URR was 0.55+ with midpoint URR showing there was minor filter fatigue in second half of therapy.

**Conclusions:** The SLEDD implementation has been successful with 98.7% of sessions delivered by ICCU staff, 73.4% met SLEDD prescription & at an optimal dose irrespective of unplanned interruption. Future challenges are improving the lifespan of the circuit avoiding unplanned interruptions.

### P305 Clinical presentation of hypertensive crises in emergency medical services

#### G Ciobanu, A Oglinda

##### State University of Medicine and Pharmacy “Nicolae Testemițanu”, Chisinau, Moldova

**Introduction:** According to the 2003 JNC 7 and to the ACC/AHA Guideline 2017 Hypertensive crisis is defined as an acute elevation of systolic blood pressure >180 mmHg or diastolic blood pressure >120 mm Hg associated with evidence of new or worsening target organ damage. However, the absolute level of blood pressure may not be as important as the rate of increase.

**Methods:** The study was conducted between January and May 2017 and included 630 subjects of both sexes, aged 28-92 with a diagnosis of hypertensive crises. All subjects were divided into two groups: hypertensive urgencies (492 subjects) and hypertensive emergencies (138 subjects).

**Results:** The total sample of 630 subjects was 57.9% female and 42.1% male. The largest number of subjects belonged to the age group of 60-69 (36.4%) years of age: 28.8% with hypertensive urgency and 38.6% with hypertensive emergency. The average blood pressure in subjects with hypertensive crisis was 216.46/122.16 mmHg. The most common symptoms of hypertensive urgency were (42.2%), epistaxis (16.4%), faintness (8%), and psychomotor agitation (10%) while the most common symptoms of hypertensive emergency were chest pain (30.4%), dyspnea (28.6%) and neurological deficit (29%). Clinical manifestations of hypertensive emergency were cerebral infarction (26.4%), acute pulmonary edema (24.8%), hypertensive encephalopathy (20.6%), acute coronary syndromes (20.4%), cerebral hemorrhage (4,.5%), congestive heart failure (12%), aortic dissection (0.8%), preeclampsia and eclampsia (2.6%).

**Conclusions:** Hypertensive urgencies were significantly more common than emergencies (78.1% vs. 21.9%, p<0.0001). There was no statistically significant difference in the number of patients with hypertensive urgency and emergency in relation to age, gender, duration of hypertension, except for the 60-69 age group, where urgency was statistically significantly higher (p=0.0204).

### P306 Severity scores APACHE II, SAPS II compared to APACHE III, SAPS III in a Moroccan medical intensive care unit

#### H Ezzouine, F Ettaya, A Raja, K Mediouni

##### Faculty of medicine and pharmacy;University Hassan II, Medical Intensive care, Casablanca, Morocco

**Introduction:** Despite the many critics they have faced, the use of gravity indices remains the only means to evaluate intensive care unit performance .The objective of this study is to compare the efficacy of different generation of the general severity of illness scoring systems .

**Methods:** Retrospective study covering a period of 6 months (1 January 2016 to 30 June 2016), conducted in the medical intensive care unit, Ibn Rushd hospital in Casablanca .The epidemiological, clinical and therapeutic data were collected studying this category of patients specificity and clinical evolution in ICU .

**Results:** 175 patients were hospitalized during the period of the study. 55% of the patients were female.The average age was 44 years .The neurological pathology was the main reason of hospitalization with 29.63% of cases.The length of stay in ICU varied between 2 and 103 days, with an average of 9.67 days. The mean severity scores was as follows APACHE II 17.79 +/-5.95; SAPS II 23.18 +/-14.01; APACHE III 46.88 +/-2.40 and SAPS III 65.92. The biological assessment at admission had anemia in 40.57% of cases, leukocytosis 56.57%, renal insuffisiency 48.57%, a hepatic cytolysis 25.14%, hydrelectrolytic disorders in 45.72%. 51.42%of patients were intubated and ventilated .72% had received corticotherapy. 38.85% of the patients had received a blood transfusion.The use of vasoactive drugs was necessary in 42.86%of cases. The outcome was favorable in 63.42%.In univariate analysis, the variables predicting mortality are the age, APACHE II, SAPS II, intubation and artificial ventilation.

**Conclusions:** The mortality remains high compared to that predicted by the scores of gravity, proving the low specificity of the indices of gravity in our context. The search for new evaluation tools adapted to our methods of work is required.

### P307 Emergency department to ICU time is associated with in-hospital mortality: a Netherlands intensive care evaluation (NICE) analysis of 14,787 university hospital patients

#### C Groenland^1^, F Termorshuizen^2^, W Rietdijk^1^, J Van den Brule^3^, D Dongelmans^4^, E De Jonge^5^, D De Lange^6^, A De Smet^7^, N De Keizer^4^, J Weigel^1^, L Jewbali^8^, E Boersma^9^, C Den Uil^8^

##### ^1^Erasmus MC, Intensive Care Medicine, Rotterdam, Netherlands; ^2^Amsterdam University Medical Center, Medical Informatics, Amsterdam, Netherlands; ^3^Radboud University Medical Center, Intensive Care Medicine, Nijmegen, Netherlands; ^4^Amsterdam University Medical Center, Intensive Care Medicine, Amsterdam, Netherlands; ^5^Leiden University Medical Center, Intensive Care Medicine, Leiden, Netherlands; ^6^University Medical Center Utrecht, Intensive Care Medicine, Utrecht, Netherlands; ^7^University Medical Center Groningen, Intensive Care Medicine, Groningen, Netherlands; ^8^Erasmus MC, Cardiology and Intensive Care Medicine, Rotterdam, Netherlands; ^9^Erasmus MC, Cardiology, Rotterdam, Netherlands

**Introduction:** Emergency Department (ED) crowding is a major public health concern. It delays treatment and possible ICU admission, which can negatively affect patient outcomes. The aim of this study was to investigate whether ED to ICU time (ED-ICU time) is associated with ICU and hospital mortality.

**Methods:** We conducted an observational cohort study using data from the Dutch NICE registry. Adult patients admitted to the ICU directly from the ED in 6 academic centers, between 2009 and 2016, were eligible for inclusion. For these patients NICE data were retrospectively extended with ED admission date and time. ED-ICU time was divided in quintiles. The data were analyzed using a logistic regression model. We estimated crude and adjusted (for disease severity; Apache IV probability) odds ratios of mortality for ED-ICU time. In addition, we assessed whether the Apache IV probability (divided into quartiles) modified the effect of ED-ICU time on mortality.

**Results:** A total of 14,787 patients were included. Baseline characteristics are shown in Table 1. The median ED-ICU time was 2.0 [IQR 1.3-3.3] hours. ICU and hospital mortality were 18.1 and 22.2%, respectively. The crude data showed that an increased ED-ICU time was associated with a decreased ICU and hospital mortality (both p<0.001, Figure 1A). However, after adjustment for disease severity, an increased ED-ICU time was independently associated with increased hospital mortality (p<0.002, Figure 1B). Figure 2 shows that only in the sickest patients (Apache IV probability >60.9%), the association between increased ED-ICU time and hospital mortality was significant (p=0.019, Figure 2D). We found similar results with respect to ICU mortality.

**Conclusions:** This study shows that a prolonged ED-ICU time is associated with increased ICU and hospital mortality in patients with higher Apache IV probabilities. Strategies aiming at rapid identification and transfer of the sickest patients to the ICU might reduce in-hospital mortality.

**Table 1 (abstract P307).** Baseline characteristics

Characteristics	All patients (n=14,787)	
Age, years, median[IQR]	59	[45-71]
Male, gender, n.(%)	9,179	(62%)
Apache IV predicted mortality, median[IQR]	0.16	[0.05-0.50]
ED-ICU time, hours, median[IQR]	2.0	[1.3-3.3]
ICU LOS, days, median[IQR]	1.7	[0.7-4.3]
ICU mortality, n (%)	2,682	(18.1%)
Hospital mortality, n (%)	3,284	(22.2%)

IQR, interquartile range; ED-ICU time, Emergency Department to ICU time; ICU, Intensive Care Unit; LOS, length of stay

**Fig. 1 (abstract P307).**
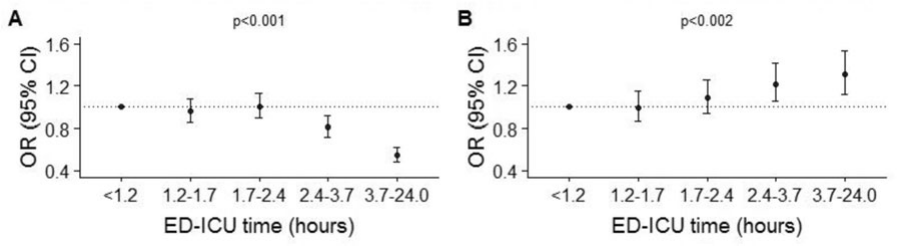
Odds Ratios for hospital mortality per lenght of stay in the Emergency Department. (1A) Crude (1B) Adjusted for Apache IV probability

**Fig. 2 (abstract P307).**
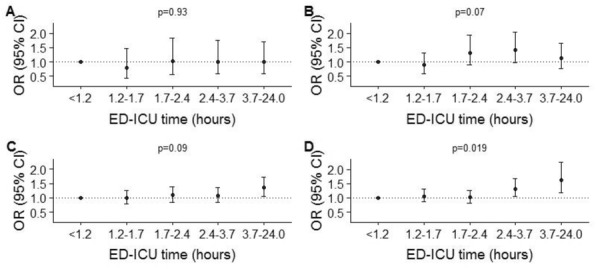
Odds Ratios for hospital mortality per lenght of stay in the Emergency Department. (2A) Association between ED-ICU time and Apache IV probability <10.5% (2B) Association between ED-ICU time and Apache IV probability 10.5-25.6% (2C) Association between ED-ICU time and Apache IV probability 25.7-60.9% (2D) Association between ED-ICU time and Apache IV probability >60.9%

### P308 Reliability and validity of the SALOMON algorithm: 5-year experience of nurse telephone triage for out-of-hours primary care calls

#### E Brasseur, A Gilbert, A Ghuysen, V D´Orio

##### CHU Liege, Emergency Departement, Liège, Belgium

**Introduction:** Due to the persistent primary care physicians (PCP) shortage and their substantial increased workload, the organization of PCP calls during out-of-hours periods has been under debate. The SALOMON (Système Algorithmique Liégeois d’Orientation pour la Médecine Omnipraticienne Nocturne) algorithm is an original nursing telephone triage tool allowing to dispatch patients to the best level of care according to their conditions [1]. We aimed to test its reliability and validity under real life conditions.

**Methods:** This was a 5-year retrospective study. Out-of-hours PC calls were triaged into 4 categories according to the level of care needed: Emergency Medical Services (AMU), Emergency Department visit (MAPH), Urgent PCP visit (UPCP), Delayed PCP visit (DPCP). Data recorded included patients’ triage category, resources and potential redirections. More precisely, patients included into the UPCP + DPCP cohort were classified under-triaged if they had to be redirected to an Emergency Department. Patients from the AMU+MAPH cohort were considered over-triaged if they did not spend at least 3 resources, 1 emergency specific treatment or any hospitalization.

**Results:** 10207 calls were actually triaged using the SALOMON tool, of which 19.1% were classified as AMU, 15.7% as MAPH, 62.8% as UPCP and 2.1% as DPCP (Fig 1). As concerns the AMU+MAPH cohort, the triage was appropriate in 85.3% of the calls, with an over-triage rate of 14.7%. As concerns the UPCP + DPCP cohort, 97.1% of the calls were accurately triaged and only 2.9% were under-triaged. SALOMON sensitivity reached 93.9% and its specificity 92.5%.

**Conclusions:** These results indicate that SALOMON algorithm is a reliable and valid nurse telephone triage tool that has the potential to improve the organization of PCP out-of-hours work.

**Reference**

1. Brasseur E et al. Crit Care 2015, 19(Suppl 1):P406.

**Fig. 1 (abstract P308).**
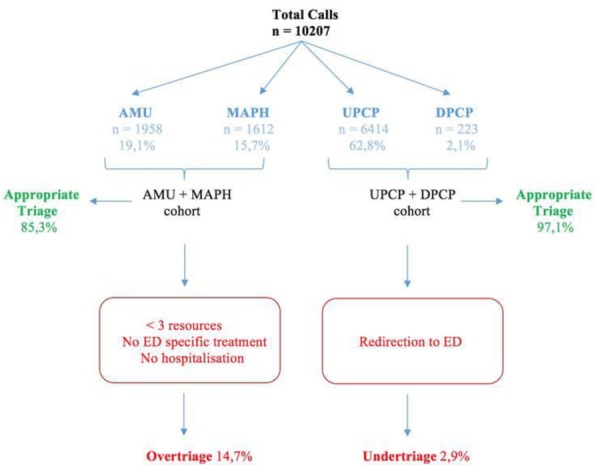
Distribution of different calls, their triage using the SALOMON algorithm and the inappropriate triages (over and undertriages) based on the preselected criteria

### P309 Advanced triage for self-referrals in the emergency department: the PERSEE algorithm

#### E Brasseur, A Gilbert, M Petit, A Ghuysen, V D´Orio

##### CHU Liege, Emergency Department, Liège, Belgium

**Introduction:** Inappropriate visits to the Emergency Department (ED), such as patients manageable by a primary care physician (PCP), have been reported to play some role in the ED crowding [1]. Indeed, non-urgent patients directly managed by PCPs could reduce ED workload [2]. Triage and diversion to alternative care facilities, eventually co-located within the ED, could offer a solution [3] provided the availability of a reliable triage tool for their early identification. We created a new triage algorithm, PERSEE (Protocoles d’Evaluation pour la Réorientation vers un Service Efficient Extrahospitalier) and tested its feasibility, performance and safety.

**Methods:** After initial evaluation with a 5-level ED triage scale [4], ambulatory self-referred patients classified as level 3 or below benefited from a simulated triage with PERSEE identifying 2 categories of patients: ED Ambulatory patients and primary care (PC) treatable patients. We collected patients data and resources. Patients requiring less than 3 resources, no specific emergency treatment and no hospitalization were considered as manageable in a PC facility.

**Results:** 1999 patients were included in the study of whom 66.9% were self-referred (Fig 1). Among those self-referrals, 58.6% were triaged as level 3 or below. 38.9% patients were triaged as Ambulatory patients of whom 10% were as PC treatable. We noted a redirection rate of 10% of the global visits or 15% of the self-referrals, an error rate of 7%, a sensitivity of 24.6% and specificity of 97.6%.

**Conclusions:** Using advanced ED triage algorithm in addition to classical ED triage might offer interesting perspectives to safely divert self-referrals to PC facilities and, potentially, reduce ED workload.

**References**

1. Van den Heede K et al. Health Policy 120:1337-49, 2016.

2. Van den Heede K et al. Health Policy 121:339-45, 2017.

3. Van Gils-Van Rooij ESJ et al. J Am Board Fam Med 28:807-15, 2015.

4. Jobe J et al. EMJ 31:115-20, 2014.

**Fig. 1 (abstract P309).**
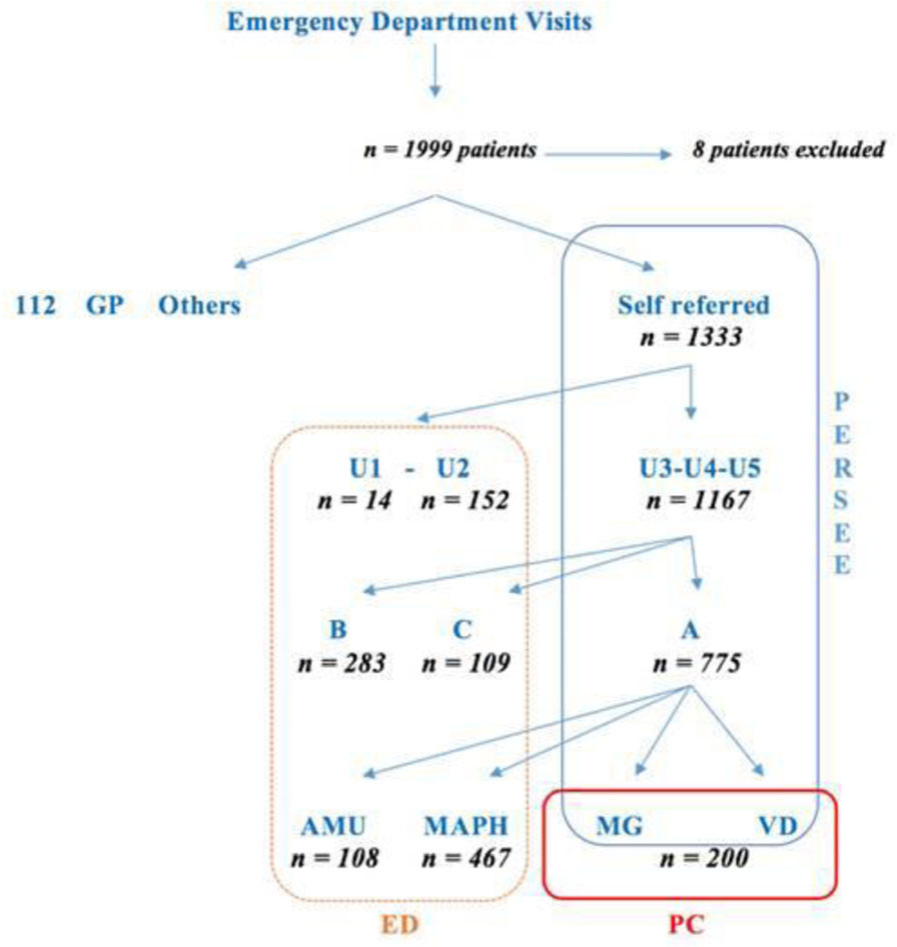
Distribution of the self-referred cohort using the PERSEE Algorithm

### P310 Analysis of relationship between number of medical procedure and staying time in the prehospital care scene. Research in Japanese air ambulance (Doctor-Heli)

#### N Yamada, T Yoshida, S Nachi, T Fukuta, R Yasuda, Y Kitagawa, N Yoshiyama, T Doi, S Nagaya, K Suzuki, M Nakano, M Tachi, T Miyake, S Ogura

##### Gifu University Hospital, Advanced Critical Care Center, Gifu, Japan

**Introduction:** Generally, prehospital medical provider should minimize staying prehospital scene to reach the patient to definitive care as soon as possible in prehospital medical activity. In addition, Some textbook and report saids that medical provider minimize the number of procedure or limit minimum requirement procedure because unnecessary procedure may extend the staying time in prehospital scene. However, there are few studies evaluating this hypothesis and that this “extension is significant or not. Therefore, we perform this study.

**Methods:** We evaluated the operated air ambulance(Doctor-Heli) case from 1st April 2015 to 31st March 2018, in Gifu University Hospital using our mission record. We evaluated about time from landing to ready for taking off(activity time), operation doctor, mission category (i.e. trauma), number of procedure in the each activity and work load. We only focused on prehospital care and exclude transportation from hospital to hospital . In addition, we exclude the case which are not suitable for analysis.

**Results:** 1299 cases were operated in these period. 511 cases were suitable for analysis. Average activity time in prehospital scene was14.32±6.09. There was weak correlation between the number of procedure and activity time. (r=0.3452) The length of the activity time did not depend on mission category. If the doctor perform 6 and over procedures, staying time was 7minutes longer, this was significantly longer than that of under 5 and under procedures.

**Conclusions:** We confirmed that we have to minimize the number of procedure or limit minimum requirement procedure in prehospital scene. And our result suggest we may have to limit appropriate number of procedures.

### P311 Prevalence of organ failure and mortality among patients in the emergency department

#### PB Pedersen^1^, DP Henriksen^2^, M Brabrand^3^, AT Lassen^1^

##### ^1^Institute of Clinical Research, Odense University Hospital, Department of Emergency Medicine, Odense C, Denmark; ^2^Odense University Hospital, Department of Emergency Medicine & Department of Respiratory Medicine, Odense C, Denmark; ^3^Odense University Hospital & Hospital of South West Jutland, Department of Emergency Medicine, Odense C & Esbjerg, Denmark

**Introduction:** Organ failure is a critical condition, but the prevalence is largely unknown among unselected emergency department (ED) patients. Knowledge of demographics and risk factors could improve identification, quality of treatment, and thereby improve the prognosis. The aim was to describe prevalence and all-cause mortality of organ failure upon arrival to the ED.

**Methods:** This was a cohort-study at the ED at Odense University Hospital, Denmark, from April 1, 2012 to March 31, 2015. We included all adult patients, except minor trauma. Organ failure was defined as a modified SOFA-score > 2 within six possible organ systems: Cerebral, Circulatory, Renal, Respiratory, Hepatic, and Coagulation. The first recorded vital, and laboratory values were extracted from the electronic patient files. Primary outcome was prevalence of organ failure; secondary outcomes were 0-7-day and 8-365-day mortality.

**Results:** Of 70,399 contacts 52.1% were female and median age 62 (IQR 42-77) years. The prevalence of new organ failure was 11.8%, individual organ failures; respiratory 4.9%, circulatory 3.0%, cerebral 2.3%, renal 1.7%, hepatic 1.4%, and coagulation 0.6%. The 0-7-day and 8-365-day all-cause mortality was 11.7% (95% CI: 10.9-12.6) and 19.3% (95% CI: 18.2-20.4), respectively, if the patient had new organ failures at first contact in the observation period, compared to 1.4% (95% CI: 1.3-1.5) and 6.6% (95% CI: 6.4-6.9) for patients without. Seven-day mortality ranged from hepatic failure, 5.6% (95% CI: 4.0-7.5) to cerebral failure, 33.3% (95% CI: 30.4-36.2), and the 8-365-day mortality from cerebral failure, 13.2% (95% CI: 11.2-15.4 to renal failure, 26.7% (95% CI: 23.6-29.9).

**Conclusions:** New organ failure is frequent and serious, with a prevalence of 11.8% and a one-year mortality of 31% with wide variation according to type of organ failure.

### P312 Epidemiological profil of patient with acute abdominal pain in the emergency department

#### M Khrouf, M Khaldi, M Ben abdelaziz, A Garrouch, A Saada, Z Mezgar, M Methamem

##### Hopitall Farhat Hached 4000 Rue Ibn AL Jazzar, SOUSSE, Tunisia

**Introduction:** Abdominal pain is the most common reason for a visit to the emergency department (ED), accounting for 8 million (7%) of the 119 million ED visits in 2006

**Methods:** The aim of our study is to determine the epidemiological and clinical feature of patients consulting the emergency department for abdominal pain .

**Results:** We proceeded to a descriptive study that showed that 39% of patients were male and 62% of them were female with a sex ratio of 0.62.The average age of patients was 34 years old and ranged between 15 and 90 years old.We found that 59 patients of our population had medical background, dominated by diabetes in 12 cases, high blood pressure in 8 cases and asthma in 6 cases.The results also showed that 29.5% of patients had a history of abdominal surgery while 13% of them had history of other types of surgery.The patients were oriented according to their severity level as following: 21% care unit of emergency department, 1.5% close monitoring room .The VASPI score was ranged between 1 and 10 with an average of 4±2. It was higher than 5 in 32.5% of cases.The results of physical examination found an isolated pain in 67,5% of cases, a reactionnal pain syndrom in15% of cases, a peritoneal syndrome in 12% of cases and an occlusive syndrome in 7% of cases.The final diagnosis was mostly represented by the following causes: 45.5% of gastroenteritis 11.5% of constipation and 9% of ulcer disease.The final orientation of patients according to the diagnosis led to hospitalization in 21% of cases and to outpatient clinic in13% of cases while 66% of them did not need any more care.

**Conclusions:** Appropriate diagnostic evaluation and decision for or against hospitalization is a challenge in the patient who comes to the emergency department with acute abdominal pain it need an adequate evaluation and management.

### P313 Self-administrated automated history-taking device in emergency department: a feasibility study

#### J Servotte^1^, M Buchet^2^, A Ghuysen^2^

##### ^1^University of Liège, Département des Sciences de la Santé publique, Liège, Belgium; ^2^University of Liège, Department of Public Health Sciences, Liège, Belgium

**Introduction:** We assessed patients’ impressions of a self-administrated automated history-taking device (tablet) to gather information concerning emergency department (ED) patients prior to physicians’ contact. The quality of communication was compared with the traditional history-taking.

**Methods:** The algorithm content was developed by two emergency physicians and two emergency nurses through an iterative process. Item-Content Validity Index (I-CVI) was measured by five experts rating the relevance of each item (from 1: not relevant to 4: highly relevant) [1]. Next, quality control was realized by research team. To assess the feasibility, we used a computerized randomization. Low acuity, ambulatory adult patients presenting to the ED were assigned either to a control group (CG, n=65) beneficiating form a traditional history-taking process or to the experimental group (EG, n=66) assigned to use the tablet with further history-taking by the ED physician. Communication was analyzed by the Health Communication Assessment Tool [2] and satisfaction assessed by questionnaires.

**Results:** After two rounds, validity was excellent for each item (I-CVI > 0.8). The Universal agreement method was of 0.9. Refusals (n=11) to participate were analyzed: they fear using an electronic device or the experimentation. Content satisfaction revealed that 93% of patients understood the questions. 94% of patients indicated that the device was easy to hold and use. Medical communication was not affected by the device (p=0.15). We noticed that, among the subsections, physicians significantly introduced themselves better in the EG (p=0.04).

**Conclusions:** In this feasibility study, patients were highly satisfied. The use of a self-administrated automated history-taking device does not generate miscommunications and allow physicians better introduce themselves.

**References**

1. Polit et al. Research in Nursing & Health 30:489-497, 2007

2. Pagano et al. Clinical Simulation in Nursing 11:402-410, 2015

### P314 Experience treat ovarian cysts in urgent gynecology

#### M Khusanhodzhaeva

##### Health Ministry of the Republic of Uzbekistan Republic Research Center of Emergency Medicine of Uzbekistan, Gynecology, Tashkent, Uzbekistan

**Introduction:** The proportion of ovarian cysts, over the past 10 years, has increased from 11% to 19-25% in the structure of all genital neoplasms. The continuing high incidence of organ-resecting surgical procedures among women of reproductive age in urgent gynecology (torsion and rupture of cysts), put forward the issue in a number of topical. Objective: To analyze the results of implementation sparing treatment strategy of ovarian cysts in emergency gynecology.

**Methods:** 1340 patients with urgent complications of ovarian cysts were for the period from 2015 to 2018. Mean age of the patients were 22.1 ± 0.7 (15 to 42) years.

**Results:** Structure of complications ovarian cysts: retention cysts rupture in 726 women, retention cysts torsion - in 390, hemorrhage into the cavity of the retention cyst - in 84, rupture of endometrioma - in 140. The volume and nature of emergency provided: transvaginal cyst aspiration in 258 (19.3), ovary resection in 549 (41.0), unilateral salpingo-oophorectomy in 135 (10.0), conservative therapy in 398 (29.7). A positive point we have established is the possibility for the detorsion of a twisted retention ovarian cyst after its transvaginal aspiration. We used this method only in cases when the onset of torsion did not exceed 4 hours. 28.5% of all emergency conditions associated with retention cysts were recurred by conservative therapy, and 50.4% of patients with the retention cysts rupture were successfully treated in this way. Conservative management is possible in the case of a small loss of blood (up to 150.0-200.0 ml), hemodynamic stability and the absence of signs of continuing bleeding. The detorsion and resection of the cyst when torsion is not more than 180 ° and even longer than 4 hours, in most cases did not reveal necrosis in the appendages.

**Conclusions:** Improvement of organs of preservation and reproduction in women.

### P315 WITHDRAWN

### P316 Criteria for admission to an intensive care unit of a tertiary hospital: analysis of the decisions of the outreach intensivist and 28 day in-hospital mortality

#### A Catarino, M Simões, P Casanova, P Martins

##### CHUC - Centro Hospitalar e Universitário de Coimbra, Serviço Medicina Intensiva, Coimbra, Portugal

**Introduction:** The aim of this study was the analysis of ICU admission criteria and evaluation of in-hospital mortality of patients assessed by our Critical care outreach team. Criteria for admission to the ICU should be defined to identify the patients most likely to benefit from ICU admission. This triage process is complex, associated with several factors, including clinical characteristics of the patients, but also subjective factors because it depends on the judgment of the intensivist who decides whether to admit or not the patient and is obviously conditioned to the structure and size of the ICU.

**Methods:** The outreach intensivist records the patient observation in a form with 5 questions (reversibility of acute illness, objective of admission in ICU, comorbidities, functional reserve and intuitive prognosis of the doctor). Analysis of 6 months (January 1 through June 30, 2018) of admission decisions in ICU, mean delay, ICU mortality, and 28 day in-hospital mortality (28HM).

**Results:** The intervention of the intensivist in “outreach” was requested on 855 occasions. The main places of observation were the emergency room (40.7%) and the wards (40.4%). The 28HM increased with the degree of comorbidity decompensation. Functional reserve also influenced 28HM, reaching 42.2% in partially dependent patients and 63.4% in totally dependent patients. There was agreement between the mortality and the physician´s intuitive prognosis in 71% of the cases.

**Conclusions:** A larger sample is needed to draw sustainable conclusions, however, the evaluation algorithm correlated well with hospital mortality. Decompensated comorbidities and low functional reserve have a negative impact on prognosis, regardless of acute disease. There was agreement between mortality and the physician´s intuitive prognosis.

### P317 Electrochemical methods for diagnosing the severity of patients with multiple trauma

#### A Evseev^1^, I Goroncharovskaya^1^, A Shabanov^2^, A Kuzovlev^3^, A Goloubev^4^, S Petrikov^1^

##### ^1^N.V. Sklifosofsky research institute of emergency medicine, Moscow, Russia; ^2^Federal Research and Clinical Center of Intensive Care Medicine and Rehabilitology, N.V. Sklifosofsky research institute of emergency medicine, Moscow, Russia, Moscow, Russia; ^3^Federal Research and Clinical Center of Intensive Care Medicine and Rehabilitology, Moscow, Russia; ^4^Federal Research and Clinical Center of Intensive Care Medicine and Rehabilitology, People`s friendship university of Russia, Moscow, Russia

**Introduction:** Multiple trauma is one of the leading causes of death worldwide [1]. Timely diagnosis and treatment is crucial in this state. One of the promising areas is the use of new electrochemical methods – they are simple, flexible, efficient and of low cost. Among these methods, attention is paid to the measurement of open circuit potential (OCP) of the platinum electrode and cyclic voltammetry (CVA). The OCP is a reflection of the balance of pro- and antioxidants in the body, and the amount of electricity (Q) determined by CVA is proportional to the antioxidant activity of the biological environment.

**Methods:** A total of 51 patients with severe multiple trauma (38.7±13.5 y.o., 33 men and 18 women) were enrolled; APACHEII 17.4±7.2; ISS 40.9±8.3; blood loss 2356±997 ml. Blood plasma was collected from patients. Measurement of the OCP was carried out according to [2], CVA analysis - according to the original method on a platinum working electrode.

**Results:** A shift in the OCP towards more positive potential values (Fig. 1), while the antioxidant activity of blood plasma decreased (Fig. 2). A more significant change of OCP, as compared to the Q values, may indicate not only a deficiency in the components of the antioxidant defense system of the body, but also an increase in the concentration of prooxidants (e.g., reactive oxygen species), which are involved in oxidative stress.

**Conclusions:** A complex electrochemical approach (open circuit potential of the platinum electrode and cyclic voltammetry) for the assessment of body pro- and antioxidant systems is an effective tool for diagnosing the state of oxidative stress in severe multiple injury.

**References**

1. Pfeifer R et al. PLoS ONE 11:e0148844, 2015

2. Khubutiya MSh et al. Russ J Electrochem 46:537-541, 2010

**Fig. 1 (abstract P317).**
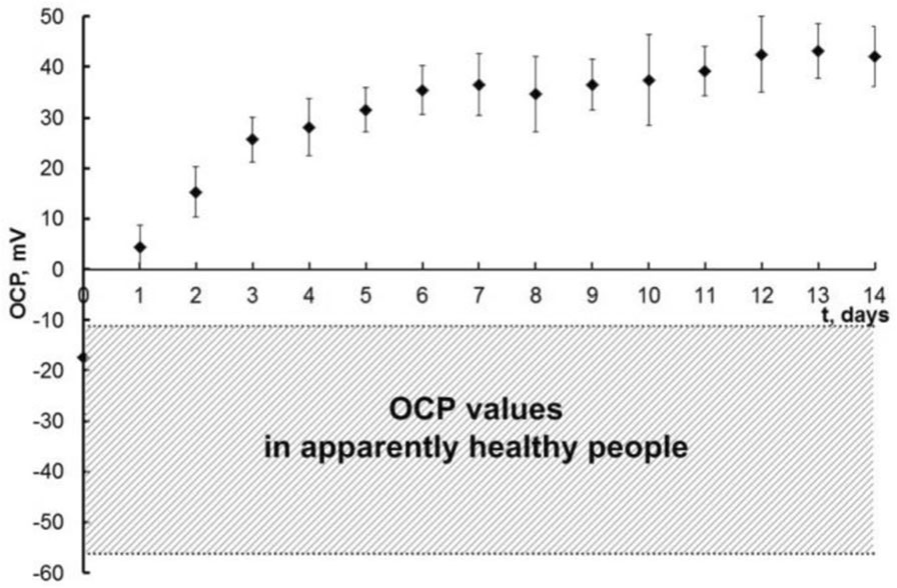
Dynamics of open circuit potential (OCP) of blood plasma of patients (in gray area - normal values)

**Fig. 2 (abstract P317).**
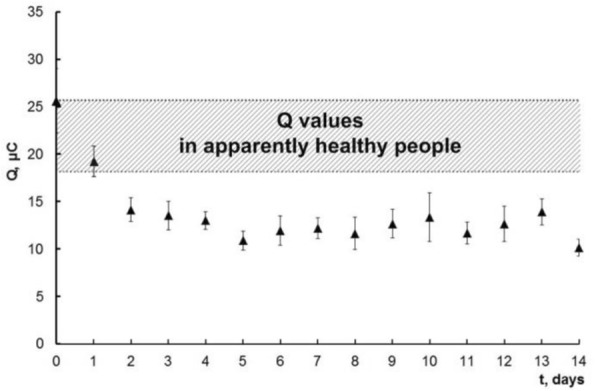
Dynamics of antioxidant activity of blood plasma of patients (in gray area - normal values)

### P318 WITHDRAWN

### P319 Outcomes following surgical rib fixation in critical care patients admitted with multiple rib fractures

#### C Achary^1^, C Pang^1^, R McCartney^1^, P Zolfaghari^2^

##### ^1^Bartshealth NHS Trust, Royal London Hospital Adult Critical Care Unit, London, United Kingdom; ^2^Bartshealth NHS Trust, Consultant Anaesthetics and Intensive Care, Royal London Hospital Adult Critical Care Unit, London, United Kingdom

**Introduction:** Patients with severe thoracic injuries are more likely to require ventilation and to suffer from pulmonary complications, but surgical fixation may provide some benefit. Since August 2014, rib fixation has been considered in these critical care patients admitted to The Royal London Hospital (RLH ACCU). We present differences in outcomes between patients undergoing surgical and non-surgical treatment.

**Methods:** Retrospective cohort analysis of adult patients admitted to the RLH ACCU with severe rib fractures (AIS T3-6) between 2012-2018. Patients were divided in three groups; Group 1 (admitted prior to 1/8/2014), Group 2 (admitted after 1/8/2014), and Group 3 (those who underwent surgical fixation). Information was collected from TARN, ICNARC and surgical team databases. Our primary outcome was ITU resource utilisation (ITU LOS and mechanical ventilation days). Our secondary outcomes were morbidity and mortality (Hospital LOS, infection burden, inotrope use and death before discharge). Data was collected and analysed in Microsoft Excel and R.

**Results:** 691 patients were included (Group 1 = 246, Group 2 = 414, Group 3 = 31). Mortality was significantly higher when comparing the post 2014 groups undergoing conservative (12%, 51/414) vs. surgical fixation (0%, 0/31), p-value = 0.026. Regarding potential temporal changes, there was no significant difference in mortality between the non surgical groups; pre-2014 (Group 1: 28/218) and post 2014 (Group 2), p-value 0.683. Group 3 patients did spend more time mechanically ventilated (p-value 0.019) and used more antimicrobials (p-value 0.032) (Table 1).

**Conclusions:** Patients undergoing surgical rib fixation at the RLH had significantly improved mortality with more days spent mechanically ventilated.

**Table 1 (abstract P319).** Comparison of resource utilisation between surgical fixation and conservative treatment in patients admitted with severe thoracic injuries

	Pre- August 2014	Post-August 2014	Surgical Fixation	
	(median ± IQR)			p-value
ITU LOS (days)	9±12	9±11	11.5±18.25	0.1042
Mechanical Ventilation days	0±1	1±8	3±10.5	0.0193
Hospital LOS (days)	21±30	21±25	29±26.5	0.3583
Inotrope requirement days	0±1	0±1.5	0±1	0.9843
Number of antimicrobials used	0±1	0±1	1±0	0.0321

### P320 Pilot study on ultrasound evaluation of epiglottis thickness in normal adult

#### A Osman^1^, A Ahmad^1^, A Azil^1^, MH Sakan^1^, MH Sakan^1^, R Idris^2^

##### ^1^Hospital Raja Permaisuri Bainun, Emergency Department, Ipoh, Malaysia; ^2^Hospital Tengku Ampuan Afzan, Emergency Department, Kuantan, Malaysia

**Introduction:** As the prevalence of epiglottitis is decreasing due to immunization, the difficulty in early detection remained. The aim of this study is to determine the thickness of epiglottis in normal adult with the utilization of bedside ultrasound.

**Methods:** This was a prospective observational study of convenience selection among healthy staff in Emergency Department, University Malaya Medical Centre. The identification and measurement of epiglottis were performed using a 10MHz linear transducer by trained emergency physicians and registrars in EM. Subjects were scanned in either standing or upright seated position with the neck neutral or mildly extended. The epiglottis, thyroid cartilage and vocal cord were visualized and the epiglottis anteroposterior(AP) diameter was measured. Difference in categorical parameters were analyzed by independent-sample t-test. The relationship between height, weight and epiglottic size was analyzed using Pearson’s correlation.

**Results:** Fifty-six subjects were analyzed with 25 males and 31 females age ranging from 18 to 50 years old. The epiglottis AP diameter ranged from 0.15cm to 0.23cm, with average of 0.18cm. There was significant difference in epiglottic AP diameter between male (M=0.19cm, SD=0.19) and female (M=0.17cm, SD=0.12; t(54)=5.27, p=<0.001, two-tailed). Moderate positive correlation between height and epiglottic AP diameter (R=0.49) and weight (R=0.33) was documented.

**Conclusions:** Our study demonstrated the identification and visualization of epiglottis was feasible and easy with the use of bedside upper airway ultrasonography. There was a little variation in the AP diameter of epiglottis in adults.

### P321 Indoor vs. outdoor occurrence in mortality of accidental hypothermia in Japan

#### Y Fujimoto^1^, T Matsuyama^2^, K Takashina^1^, T Takegami^1^

##### ^1^Japanese Red Cross Kyoto Daiichi Hospital, Emergency service, Kyoto, Japan; ^2^Kyoto Prefectual University of Medicine, Emergency medicine, Kyoto, Japan

**Introduction:** The impact of location of accidental hypothermia (AH) occurrence has not been sufficiently investigated so far. Thus we aimed to evaluate the differences between indoor and outdoor occurrence about baselines, occurrence place, mortality, and length of ICU stay and hospital stay.

**Methods:** This was a multicenter retrospective study of patients with a body temperature ≤35 °C taken to the emergency department of 12 hospitals in Japan between April 2011 and March 2016. We divided the included patients into the following two group according to the location of occurrence of AH (Indoor versus Outdoor). The primary outcome of this study was in-hospital death. Secondary outcomes were the length of ICU stay, and hospital stay.

**Results:** A total of 537 patients were enrolled in our hypothermia database. There were 119 and 418 patients with the outdoor and indoor occurrence. The indoor group was older (71 versus 81.5 years-old, p<0.001) and worse in ADL than the outdoor group. The proportion of in-hospital death was higher in the indoor group than the outdoor group (28.2% [118/418] versus 10.9% [13/119], p<0.001). The multivariable logistic regression analysis demonstrated that Adjusted Odds Ratio of the indoor group over the outdoor group was 2.48 (95%CI; 1.18 to 5.17) (Table 1). As for secondary outcomes, both of the length of ICU stay and hospital stay in survivors were longer in the indoor group than the outdoor group.

**Conclusions:** Our multicenter study indicated that Indoor occurrence hypothermia accounts for about 78% of the total in this study, and the proportion of in-hospital death was higher in the indoor group. We have to raise an alert over the indoor onset accidental hypothermia and need to take countermeasures for prevention and early recognition of AH in indoor location.

**Table 1 (abstract P321).** Outcomes

Characteristics	Outdoor	Indoor	P values
	(n=119)	(n=418)	
Primary Outcome			
In-hospital death	13(10.9%)	118(28.2%)	<0.001
Adjusted Odds Ratio (95%CI)	Reference	2.48(1.18-5.17)	
Secondary Outcomes			
hospital stay	5(2-15.5)	15(4-32.25)	<0.001
ICU stay	2(1-4)	3(2-6)	0.02

### P322 Hyperlactatemia during acute asthma attack

#### P Tedchai^1^, Y Sutherasan^2^, T Kaewamatawong^3^, S Trakulsrichai^4^, P Theerawit^5^

##### ^1^Faculty of medicine Ramathibodi hospital, Department of Medicine Ramathibodi Hospital, Mahidol University, Bangkok, Thailand; ^2^Faculty of medicine Ramathibodi hospital, Medicine, Bangkok, Thailand; ^3^Faculty of medicine Ramathibodi hospital, Division of Pulmonary and Pulmonary Critical Care Medicine, Department of Medicine Ramathibodi Hospital, Mahidol University, Bangkok, Thailand; ^4^Faculty of medicine Ramathibodi hospital, Department of Emergency Medicine, Faculty of Medicine, Ramathibodi Hospital, Mahidol University, Bangkok, Bangkok, Thailand; ^5^Faculty of medicine Ramathibodi hospital, Division of Critical Care Medicine, Department of Medicine Ramathibodi Hospital, Mahidol University, Bangkok, Thailand

**Introduction:** Hyperlactatemia has been reported in acute asthmatic attack patients. Possible causes of hyperlactatemia in asthma include hypoxemia, increase in work of breathing and beta-2 agonist therapy. Our objectives are to evaluate arterial lactate levels during treatment of acute asthma.

**Methods:** We performed a prospective study at Emergency Department(ED) of Ramathibodi hospital in patient with acute asthma who treated with either salbutamol or ipratropium bromide and fenoterol nebulizer solutions at 30-minute intervals for 4 times. Arterial lactate concentrations and physiologic parameters were measured at ED arrival and 2 hr after treatment.

**Results:** A total of 29 subjects were enrolled. The mean age was 55.55±20.30 years. Baseline systolic pressure (SBP), diastolic pressure (DBP) and SpO2(%) were 145.55±26.14 mmHg, 80.41±13.93mmHg and 95.24±3.09 respectively. The initial lactate was 2.27±1.67 mmol/L. At 2 hr after treatment with 4 doses of bronchodilator, the medication significantly decreased SBP from 145.55±26.14 to 133.48±15.24 mmHg (P=0.003), DBP from 80.41±13.93 to 74.86±9.75 mmHg (P=0.03) and RR from 24.41±5.36 to 21.55±1.76 breaths/min (P=0.004). The SpO2 (%) was increased (95.24±3.09 vs. 97.38±2.12; P<0.001). The lactate slightly increased from baseline, but no statistical significance (2.27±1.67 vs. 2.56±1.67 mmol/L; P=0.115). However, an increasing of lactate was found in sixteen subjects (55.2%). Subgroup analysis of lactate increase found significant rising of lactate from baseline after bronchodilator therapy (1.83±1.63 vs. 2.72±1.76 mmol/L; P<0.001). The Pearson correlation showed only significant relationship between the Δ lactate with Δ SpO2 (r=0.552; P=0.027), but not with ΔSBP and Δ DBP.

**Conclusions:** During acute asthmatic attack, arterial hyperlactatemia is frequently present at ED arrival. Nevertheless, the plasma lactate level was no significant difference between ED admission and 2 hr after treatment.

### P323 The results of the treatment of limb bone fractures in children with combined injuries

#### T Musaev, F Masharipov, N Tolipov, O Ganiev

##### Health Ministry of the Republic of Uzbekistan Republic Research Center of Emergency Medicine of Uzbekistan, Traumatology and Orthopedics, Tashkent, Uzbekistan

**Introduction:** In the treatment of limb bone fractures in children with severe concomitant injury, the timing and the choice of fracture treatment methods remain. Some authors advocate early surgical treatment of bone damage, while others support conservative treatment methods.

**Methods:** From 2005 to 2017, 1,330 children with combined injuries of the musculoskeletal system (MSS) of varying severity were treated in the Department of Pediatric Traumatology of the Republican Scientific Center for Emergency Medicine of the Ministry of Health of the Republic of Uzbekistan. After the accident, 998 (75%) were treated, 201 (15.1%) children fell from a height. The remaining children - 131 (9.8%) of combined damage to the musculoskeletal system received as a result of household, street and sports injuries. Conservative treatment for MSS was performed in 559 (42%) of the injured children with associated injuries, operative in 771 (58%).

**Results:** In terms of MSS operations, 571 (42.8%) were patients who underwent early surgical intervention (in the first two days), including 275 (48%) used external fixation devices, and 296 (52%) traditional surgical methods of osteosynthesis, mainly intramedullary fixation. 200 (15.1%) patients underwent delayed surgery (later than two days after the injury). So in 52 cases, osteosynthesis was carried out in a delayed manner due to unstable hemodynamics or low hemogram values during surgery for damage to internal organs, and in 148 cases after preliminary treatment of damage to the ODE using skeletal traction. Studying the immediate results of surgical treatment of children with combined injuries of MSS showed that good results were obtained in 651 (84.4%), satisfactory results in 102 (13.2%), and unsatisfactory results in 18 (2.4%).

**Conclusions:** Early surgical stabilization of injuries of the skeletal system in severe concomitant injury in children in 84.4% of cases resulted in positive treatment results.

### P324 Point of care transesophageal echocardiography (TEE) in early diagnosis of traumatic aortic injury (TAI) at emergency department

#### A Osman^1^, M Mohamed Sakan^2^, P Chan^1^, A Azil^1^, A Abdullah^3^

##### ^1^Hospital raja permaisuri bainun, Emergency and Trauma Department, perak, Malaysia; ^2^Hospital raja permaisuri bainun, Emergency Medicine, perak, Malaysia; ^3^Hospital Serdang, Cardiology Department, Selangor, Malaysia

**Introduction:** This is a case series of traumatic aortic injury (TAI) which was diagnosed by transesophageal echocardiography (TEE) in the emergency department. The number of patients with blunt thoracic aorta injury arriving at emergency department is on the rise and survival rate is time-dependent on early diagnosis. TEE offers several advantages over transthorasic echocardiography (TTE) including reliability, continuous image acquisition and superior image quality.

**Methods:** All trauma patients who presented to emergency department from 1st January 2017 until 30th November 2018 at Hospital Raja Permaisuri Bainun, Perak, Malaysia with suspected TAI were evaluated with transesophageal echocardiography. Over the 2 years period, TEE was performed in 30 patients. 6 patients had positive findings suggestive of TAI.

**Results:** The first case was an old lady who presented after a deceleration injury in a car accident. TEE was performed due to hemodynamic instability and found an intimal flap along the ascending aorta. The second case, a Stanford Type A (Figure 1), was complicated with pericardial tamponade. The intimal flap was visualised from the aortic arch extending to the descending aorta by TEE. The third case was a case of intramural haematoma involving distal aortic arch extending to the descending aorta which survived until corrective surgery. In the fourth case, TEE revealed a motion artefact which mimicked an intimal flap in the ascending aorta. In the fifth case, TEE showed intimal flap at aortic isthmus which was not detected by TTE. In the last case, a traumatic aortic dissection was complicated by aortic regurgitation (Figure 2).

**Conclusions:** TEE can be a useful point of care tool use by emergency and critical care physicians for early diagnosis of blunt traumatic aorta injury.

**Fig. 1 (abstract P324).**
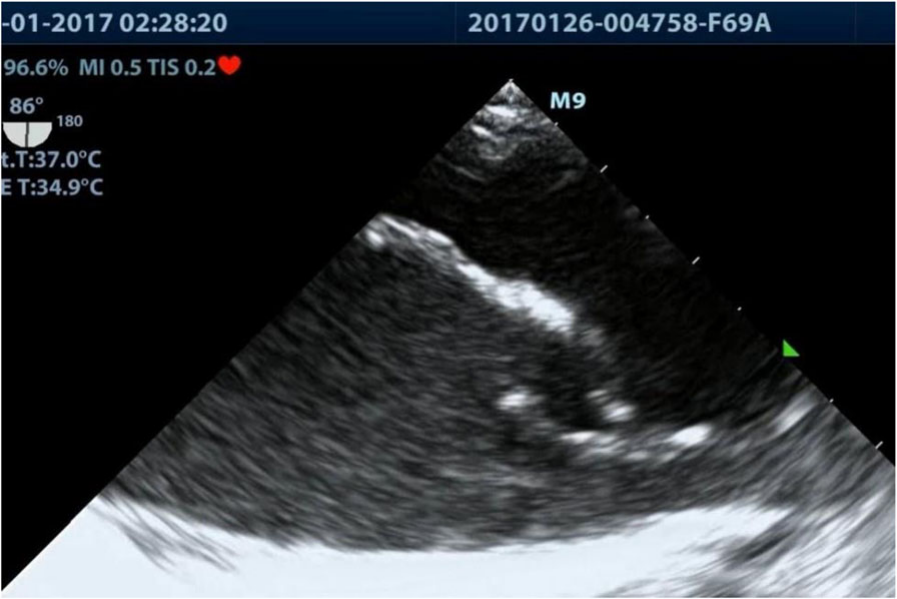
Intimal flap at descending aorta

**Fig. 2 (abstract P324).**
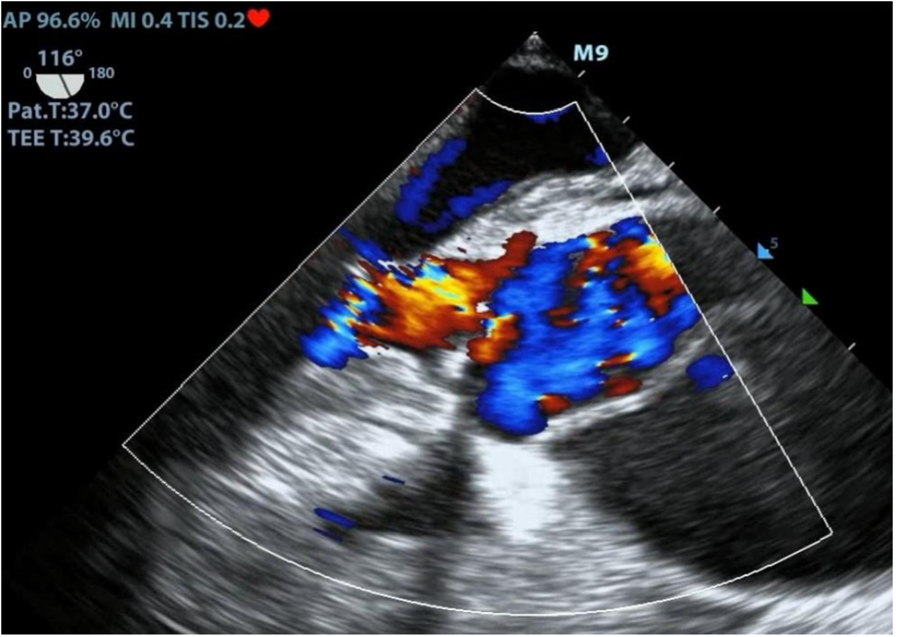
Ascending aortic dissection complicated by aortic regurgitation

### P325 Resuscitative endovascular balloon occlusion of the aorta (REBOA) in the haemodynamically unstable trauma patient: a systematic review

#### J Scanlon^1^, J Wilkinson^2^, R Pugh^1^

##### ^1^Glan Clwyd Hospital, Department of Anaesthesia, Bodelwyddan, United Kingdom; ^2^Countess of Chester Hospital, Department of Intensive Care, Chester, United Kingdom

**Introduction:** REBOA is an endovascular intervention intended to preserve central perfusion in the context of shock due to non-compressible torso haemorrhage. More so, it is less invasive than the traditional approach of resuscitative thoracotomy (RT) and aortic cross-clamping. Though its use dates back to the Korean War, it has not been widely adopted in trauma management, as evidence demonstrating clear benefit compared with conventional RT is lacking [1]. We aimed to evaluate feasibility, outcomes and complications after REBOA for haemorrhagic shock and traumatic cardiac arrest.

**Methods:** We performed a systematic literature review, searching SCOPUS and PUBMED databases using relevant terms (July 2018). We included studies enrolling patients with haemorrhagic shock or cardiac arrest after civilian trauma who had undergone REBOA and reported hospital mortality (our primary outcome). Abstract-only studies and single-patient case reports were excluded. We collated and analysed data using Review Manager v5.3. The Newcastle-Ottawa scale was used to assess risk of bias.

**Results:** Sixteen in-hospital studies met inclusion criteria (n=2034). Ten were case series and six were cohort studies comparing REBOA outcomes with those of RT. There were wide differences between studies’ inclusion criteria, case-mix (including cardiac arrest), injury severity, insertion details, and reported outcomes. Overall hospital mortality post-REBOA was 61.4%. Meta-analysis of cohort studies indicated notably lower mortality in patients undergoing REBOA (OR 0.41, 0.32-0.54) than RT with low statistical heterogeneity between studies (I^2^ = 0%), shown in Fig 1.

**Conclusions:** Whilst our findings are limited by methodological differences and biases in the included studies, almost 40% of patients undergoing REBOA for haemorrhagic shock and/or cardiac arrest survived to discharge. Furthermore, REBOA appeared to offer a consistent mortality benefit compared with RT.

**Reference**

1. Brenner et al. Trauma Surg Acute Care Open 2018;3:1–3.

**Fig. 1 (abstract P325).**
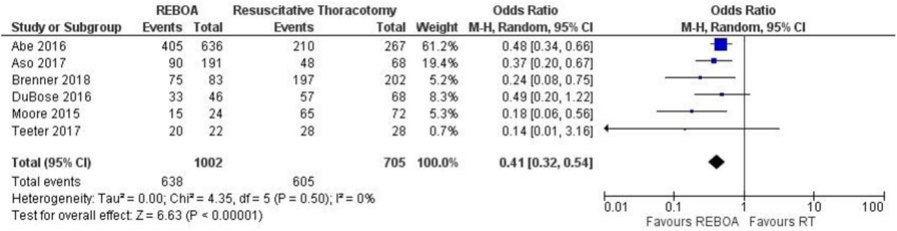
Forest plot: hospital mortality REBOA vs RT

### P326 Pre-hospital use of fibrinogen concentrate in traumatic bleeding patients

#### B Ziegler^1^, H Haberfellner^2^, C Niederwanger^3^, M Bachler^4^, B Schenk^4^, B Treichl^4^, H Schöchl^5^, W Völkl^5^, W Martini^6^, U Martinowitz^7^, D Fries^8^

##### ^1^Perioperative Medicine and General Intensive Care, Paracelsus Medical University, Department of Anaesthesiology, Salzburg, Austria; ^2^Medical University, Department of Anaesthesiology, Innsbruck, Austria; ^3^Medical University, Department of Pediatrics, Innsbruck, Austria; ^4^Department of General and Surgical Critical Care Medicine, Innsbruck, Austria; ^5^AUVA Trauma Centre, Department of Anaesthesiology and Intensive Care, Salzburg, Austria; ^6^Fort Sam Houston, US Army Institute of Surgical Research, San Antonio, United States; ^7^The Chaim Sheba Medical Centre, Institute of Thrombosis and Haemostasis and the National Haemophilia Centre, Tel Hashomer, Israel; ^8^Department of General and Surgical Critical Care Medicine, Medical University Innsbruck, Innsbruck, Austria

**Introduction:** Trauma related coagulopathy remains a primary contributor to mortality on battlefields and in civilian trauma centres. Fibrinogen is considered to be the first to drop below critical level and correspondingly compromised coagulation process. However, it is unclear if fibrinogen concentrate at a very early stage is feasible and effective to prevent from coagulopathy.

**Methods:** A total of 66 acutely injured patients in Austria, Germany and Czech Republic were screened and enrolled in this controlled, prospective randomized placebo controlled double blinded multicentre and multinational trial. Upon the completion of randomization, fibrinogen concentrate (50 mg/kg, FGTW©, LFB France) or placebo was reconstituted and given to the patients at the scene or during helicopter transportation from the scene to nearby hospitals. Blood samples were taken at baseline (scene of accident before study drug administration), at the emergency room, three hours, nine hours and twenty-four hours after admission to the hospital as well as after three and seven days after admission, for measurements of blood gases and coagulation, together with clinical data and outcome records.

**Results:** The demographic and injury characteristics and the estimated blood loss, ISS, and GCS at the scene were similar in both groups. In the placebo group, fibrinogen concentration dropped from 200 mg/dL at injury site to 160 mg/dL () at ER admission and clot stability reduced from 13.5 mm (4, 21 mm) to 10 mm (p=0.013) (Fig 1). Fibrinogen concentrate administration prevented the drop of fibrinogen level (baseline of 170 mg/dL to 210 mg/dL and improved clot stability from 13 mm at baseline to 16 mm at ER.

**Conclusions:** Pre-hospital administration of fibrinogen concentrate in traumatic bleeding patients is feasible and effective in preventing the development of coagulopathy. Data from this study support the use of fibrinogen to prevent trauma related coagulopathy.

**Fig. 1 (abstract P326).**
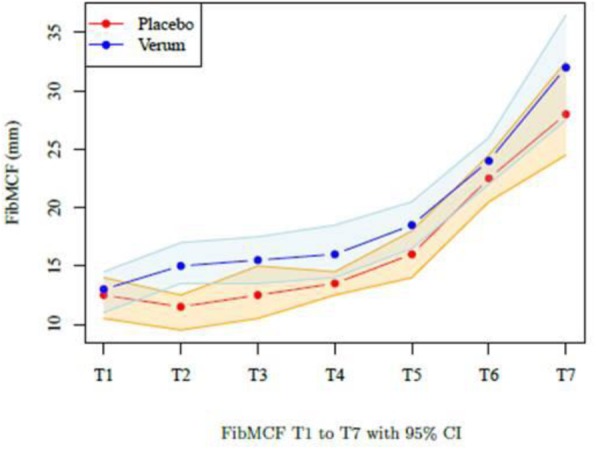
Fib MCF T1 to T7 with 95% CI

### P327 Fibrinogen concentrate vs cryoprecipitate in pseudomyxoma peritonei surgery: results from a prospective, randomised, controlled phase 2 study

#### A Roy^1^, N Sargant^1^, S Rangarajan^1^, S Alves^1^, J Bell^1^, S Stanford^1^, C Solomon^2^, I Kruzhkova^2^, S Knaub^2^, F Mohamed^1^

##### ^1^Basingstoke and North Hampshire Hospital, Basingstoke, United Kingdom; ^2^Octapharma AG, Lachen, Switzerland

**Introduction:** Maintaining adequate plasma fibrinogen levels during cytoreductive surgery for pseudomyxoma peritonei (PMP) may help control haemostasis. FORMA-05 compared the efficacy and safety of cryoprecipitate (cryo) with a new highly purified, double virus-inactivated human fibrinogen concentrate (HFC; *Octafibrin*, Octapharma) in patients (pts) with acquired fibrinogen deficiency undergoing surgery for PMP.

**Methods:** FORMA-05 was a prospective, single centre, randomised, controlled phase 2 study. Pts undergoing surgery for PMP with predicted intraoperative blood loss ≥2 L preemptively received HFC (4 g) or cryo (2 pools of 5 units). The composite primary endpoint was intraoperative (assessed by surgeon/anaesthesiologist) and postoperative efficacy (assessed by haematologist), graded using objective 4-point scales and adjudicated by an Independent Data Monitoring & Endpoint Adjudication Committee.

**Results:** The per-protocol set included 43 pts (HFC, n=21; cryo, n=22). The mean total intraoperative dose of HFC was 6.5 g vs 4.1 pools of cryo (containing approx 8.8 g of fibrinogen). Median duration of surgery was 7.7 h. Overall haemostatic efficacy of HFC was non-inferior to cryo and was rated excellent or good for 100% of pts receiving HFC and cryo, with similar blood loss. Intraoperatively, only red blood cells were transfused (median: 1 unit). Intraoperative efficacy is shown in Table 1. Infusions were initiated 0.4 h earlier with HFC than cryo due to faster product availability. Preemptive HFC led to a greater mean increase vs cryo in FIBTEM A20 (Figure 1) and plasma fibrinogen (Figure 2). There were 6 serious adverse events (SAEs) in the HFC group and 17 in the cryo group, including 7 thromboembolic events (TEEs; 2 deep vein thromboses, 5 pulmonary embolisms). No AEs or SAEs were deemed related to the study drug.

**Conclusions:** HFC was efficacious for treatment of bleeding in pts undergoing surgery for PMP. No related AEs and no TEEs occurred in pts treated with HFC.

**Table 1 (abstract P327).** Intraoperative haemostatic efficacy ratings (n=43)

4-point Efficacy Scale	Assessed by the Surgeon and Anaesthesiologist	Assessed by the Surgeon and Anaesthesiologist	Assessed by the IDMEAC	Assessed by the IDMEAC
	HFC N (%)	Cryoprecipitate N (%)	HFC N (%)	Cryooprecipitate N (%)
Excellent	13 (61.9)	12 (54.5)	13 (61.9)	11 (50)
Good	7 (33.3)	6 (27.3)	7 (33.3	5 (22.7)
Moderate	1 (4.8)	4 (18.2)	1 (4.8)	6 (27.3)
None	0.0	0.0	0.0	0.0
Missing	0.0	0.0	0.0	0.0
Total	21 (100)	22 (100)	21 (100)	22 (100)

**Fig. 1 (abstract P327).**
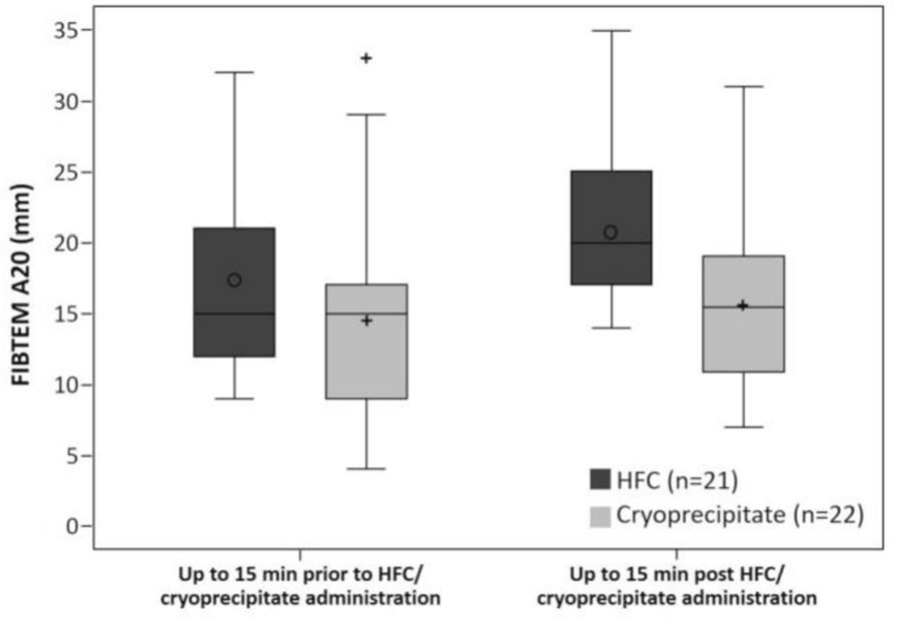
FIBTEM A20 prior to and following the preemptive dose of HFC/cryoprecipitate

**Fig. 2 (abstract P327).**
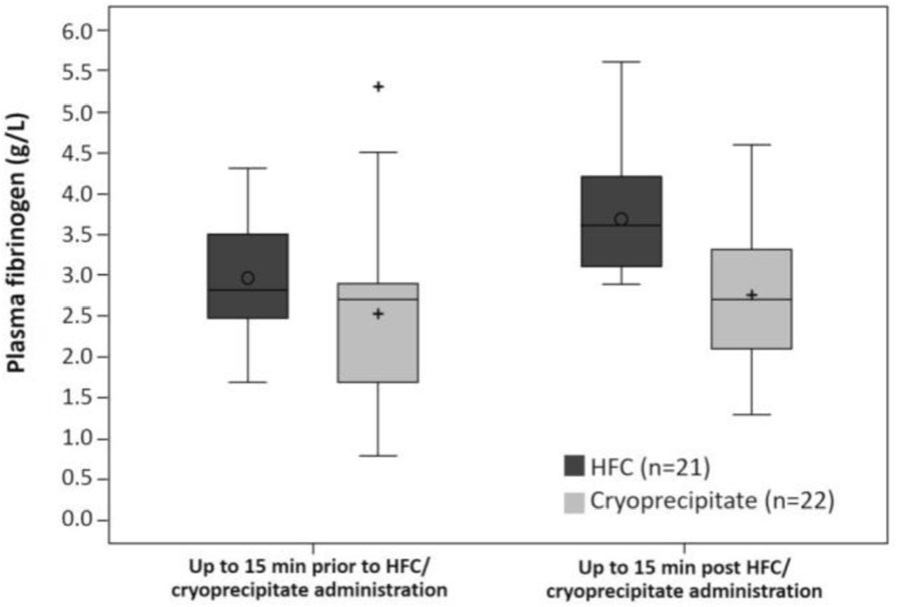
Fibrinogen levels prior to and following the preemptive dose of HFC/cryoprecipitate

### P328 A novel microchip flow-chamber system for evaluating comprehensive thrombogenic activity in thrombocytopenic patients

#### B Atari^1^, T Ito^2^, T Nagasato^3^, K Hosokawa^3^, T Yasuda^2^, Y Kakihana^2^

##### ^1^Kagoshima University Hospital, Emergency and Critical Care Center, Kagoshima, Japan; ^2^Kagoshima University Hospital, Kagoshima, Japan; ^3^Fujimori Kogyo Co., Ltd., Yokohama, Japan

**Introduction:** Patients in the intensive care unit often suffer from thrombocytopenia. In dealing with this problem, we need to figure out not only the cause of thrombocytopenia but also the risk of bleeding. However, there is no reliable method for evaluating bleeding risk.

**Methods:** In this preliminary study, four thrombocytopenic patients who required platelet transfusion before undergoing invasive procedure were enrolled. Written informed consent was obtained from all patients for participation in the study. Bleeding was graded using the WHO bleeding scale. Thrombogenic activity was evaluated using total thrombus-formation analysis system (T-TAS), rotational thromboelastometry (ROTEM), and multiplate impedance aggregometry. For T-TAS analysis, we prepared a novel microchip, named HD chip, which is suited for analyzing low platelet samples rather than those with normal platelet counts.

**Results:** Two patients showed no or minor bleeding (WHO bleeding grade 0-1) while other two patients showed significant bleeding (grade 2-3). Platelet counts (25-43 vs 27-58 x 10^3^ cells/μ L), maximum clot firmness in EXTEM (27-45 vs 34-40 mm), and multiplate aggregation in response to the collagen stimulation (101-119 vs 94-114 U) were not different between the minor bleeding group and the significant bleeding group. By contrast, the occlusion time in the T-TAS HD chip was prolonged in the significant bleeding group (longer than 30 minutes in both cases) compared to the minor bleeding group (17.4-20.3 minutes).

**Conclusions:** The T-TAS HD chip might be useful in evaluating bleeding tendency.

### P329 Fresh frozen plasma in acute haemorrhage: use, wastage and appropriateness of transfusion. A baseline service evaluation of practice in a large UK district general hospital

#### A Garland, C Pritchett, J Cheung

##### Royal Cornwall Hospital, Department of Anaesthesia and Critical Care, TR5 0SL, United Kingdom

**Introduction:** We investigated wastage of Fresh Frozen Plasma (FFP), key patient groups in which it was wasted and the use of standard laboratory tests (SLTs) to guide its use. The purpose was to assess the potential benefit a point of care viscoelastic haemostatic assay (VHA) could have on FFP transfusion and waste. The National Blood Transfusion Committee and NHS Blood and Transplant committee have published data showing that up to 25% of FFP is transfused inappropriately [1].

**Methods:** Blood bank data was obtained evaluating haemorrhaging patients in whom FFP was requested across a nine-month period in 2018. Patient bleeds were categorised by speciality. The mean time FFP dispensed and wasted was recorded, as were timings of SLT requests. Where available, the INR result was recorded.

**Results:** 66 patients were identified. 74 transfusions were requested. Table 1 shows that the highest transfusion requirements are for acute medical emergencies and major trauma. 70% of transfusion were surgical specialities, it would be expected that these patients would have anaesthetic or critical care input. 242 units were wasted. Acute medical emergencies wasted the highest amount of FFP (82 units). Table 2 demonstrates that 29.7% of transfusions had an INR available one hour prior to FFP being dispensed.

**Conclusions:** We conclude that use of SLTs to guide FFP transfusion is low. This suggests transfusion decisions are being made clinically. A point of care VHA could give treating physicians better access to timely haemostatic data.

**Reference**

1. Sherliker et al National Blood Transfusion Committee, NHS Blood and Transplant, 2015 Survey of Patient Blood Management. 2015.

**Table 1 (abstract P329).** Showing who bleeds, FFP units dispensed, and FFP wasted (n= 66)

Speciality	Total Units Used	Wasted FFP Units	Mean FFP given (n)	Mean FFP wasted (n)
Vascular N= 14	11	49	0.79	3.5
Obstetric N=11	1	55	0.09	5
Acute Medical Emergency N=20*	16	82	0.8	4.1
Urology N=8	2	7	0.66	2
Major Trauma N=10	22	15	2.75	1.86
General Surgery N=2	7	32	0.7	3.2
Orthopaedics N=1	0	2	0	2

*Acute medical emergency encompasses medical emergencies on the intensive care unit, acute medical unit and the emergency department

**Table 2 (abstract P329).** Showing INR result time prior to FFP dispensation. (N=74)

Time prior to transfusion (Minutes)	Number =	Percentage (%)
0-30	15	20.2
31-60	7	9.5
>60	14	19
Unable to quantify	4	5.4
Post transfusion Coagulation Result	34	45.9

### P330 Out-of-hospital blood transfusion is as safe as conventional blood transfusion in the hospital?

#### C Carriedo Scher^1^, M Madrigal Sanchez^2^, L Mifsut Rodriguez^3^, A Pacheco Rodriguez^4^, R Estevez Montes^4^, A Cortina Nieto^2^

##### ^1^GUETS - Gerencia de Urgencias, Emergencias y Tte. Sanitario, Department of Medecine, Toledo, Spain; ^2^Hospital General Universitario de Ciudad Real, Center of Transfusions, HGUCR, Ciudad Real, Spain; ^3^GUETS - Gerencia de Urgencias, Emergencias y Tte. Sanitario, Department of Medecine, Babcock International, Toledo, Spain; ^4^GUETS - Gerencia de Urgencias, Emergencias y Tte. Sanitario, Gigante 2 - HEMS GUETS-SESCAM, Toledo, Spain

**Introduction:** We developed the process for the out-of-hospital packed red blood cells (pRBC) transfusion in the HEMS of Castilla-La Mancha CLM according to criteria of medical indications, security, monitoring and tracking. Haemorrhage is a preventable cause of death among population suffering accidents or bleeding injuries in regions with low population density where health services should reach people in remote areas. HEMS of CLM is the first out-of-hospital Emergency Service in Spain that provides pRBC transfusion there where the accident takes place. This program has been developed jointly between hematologists of the Center for Transfusions CT and the HEMS team.

**Methods:** Observational retrospective study with data collected from June 2014 to August 2018. The medical helicopter was provided with two pRBC O Rh(D) negative (Fig 1). Shock Index was selected as indication for transfusion. To achieve feasibility and preservation of the pRBC it was established a prospective monitoring and microbiological culture for both groups: case group for the pRBC kept in the HEMS and control group in the Hospital (Fig 2). Controls and comparison of hematologic analysis were performed immediately and 35 days after collection. Statistics used SPSS 23.0 (signification p<0.05).

**Results:** 138 pRBC were evaluated, 67 case -71 control. Analyses were tested days 1 and 35 after collection. Hemolysis was not observed. All cultures were negative. Results obtained of the pRBC after 35 days transported in the HEMS related to monitoring parameters were not different than those observed on pRBC conserved in the CT. 35 pRBC were transfused to 28 patients in out-of-hospital assistance. Neither post-transfusional reactions or undesirable events have been registered. pRBC units are changed every 30 days.

**Conclusions:** The process designed (collection, conservation, tracking and tests) to make pRBC available in the medical helicopter has demonstrated to keep the standard conditions and properties to be transfused in critically ill patients out-of-hospital.

**Fig. 1 (abstract P330).**
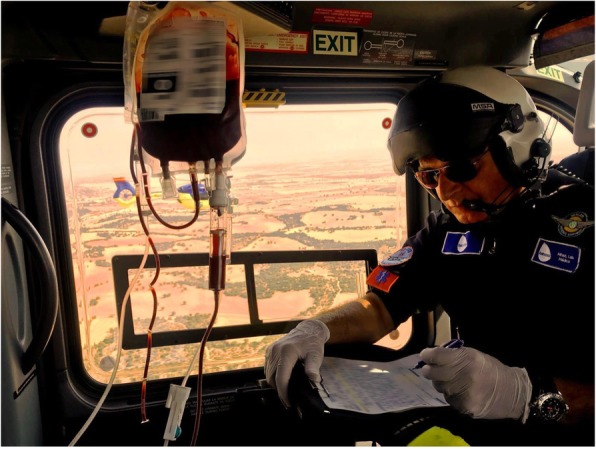
In-flight pRBC transfusion. Picture reproduced with subject’s permission

**Fig. 2 (abstract P330).**
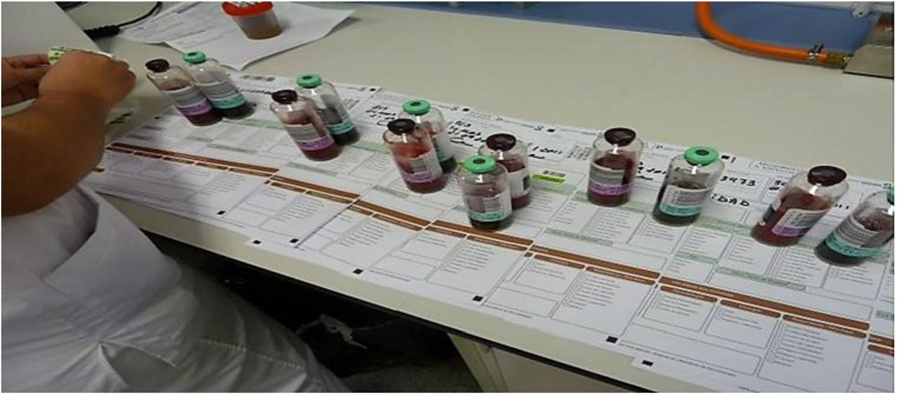
Blood lab controls

### P331 Outcomes in patients with a haematological malignancy admitted to a general intensive care unit

#### A Corner

##### East Sussex Healthcare NHS Trust, Intensive Care, Eastbourne, United Kingdom

**Introduction:** Recent published data have challenged the view that critically ill patients with a haematological malignancy have a poor prognosis [1]. Reports have largely originated from tertiary centres. The aim of this audit was to evaluate the intensive care unit (ICU), in hospital and one year mortality for a cohort of patients admitted to a mixed medical and surgical ICU in a district general hospital.

**Methods:** Details were obtained for all patients with a haematological malignancy admitted to Eastbourne and Hastings ICU between March 2012 and August 2017. Patient characteristics, type of malignancy, reason for admission, degree of organ support and survival rates at ICU discharge, hospital discharge and 1 year post-admission were collected.

**Results:** 60 patients, 65% male, were identified. Median (interquartile range, IQR) age was 68 (60-73) years. 55% had neutropenia. The commonest malignancies were acute leukaemia 43%, lymphoma 27% and myeloma 17%. Reasons for admission were respiratory 48%, cardiac 18% and renal 10%. Organ supports used were noradrenaline 68%, intubation and mechanical ventilation 42%, renal replacement therapy (RRT) 32% and dobutamine 10%. Overall survival rates are shown in Figure 1. 7 patients were discharged from hospital following a period of mechanical ventilation. For these patients, median (range) age was 64 (26-72) years. All were male. Median (IQR) time in hospital prior to admission was 0 (0-2) days, 7/7 patients required vasoactive support, 3/7 required RRT, median ICU length of stay was 12 (3-25) days. 3/7 were admitted following surgery for an unrelated condition. To date, only 1/7 patient has survived 5 years post ICU admission.

**Conclusions:** Although survival rates were disappointing, particularly in those patients requiring mechanical ventilation, selected patients have the potential for a good outcome. These results outcomes have been presented to our Haematology Department to aid patient counselling.

**Reference**

1. Bird GT et al. British Journal of Anaesthesia 108:452-9,2012

**Fig. 1 (abstract P331).**
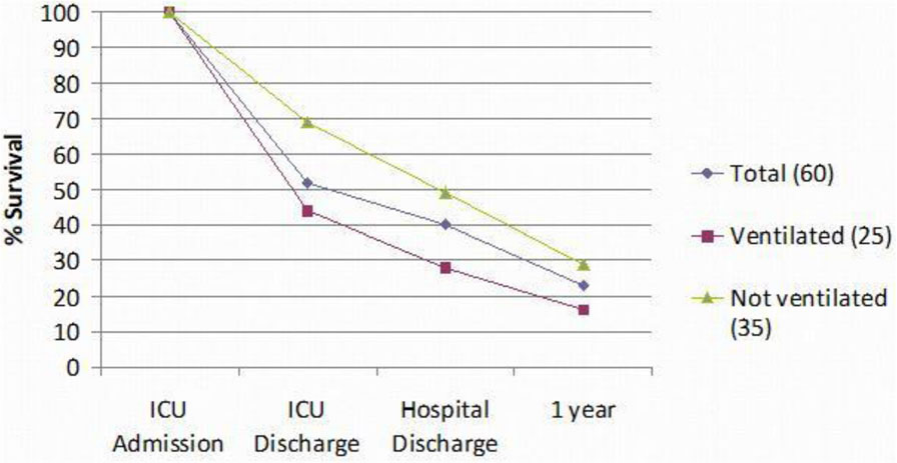
Percentage survival at ICU discharge, hospital discharge and 1 year

### P332 Routine coagulation tests on admission are associated with mortality and morbidity in general ICU-patients: an observational study

#### S Benediktsson, C Hansen, A Frigyesi, T Kander

##### Skåne University Hospital, Intensive and Perioperative Care, Lund, Sweden

**Introduction:** It is well known that low platelet count on admission to intensive care units (ICU) is associated with increased mortality. However, it is unknown whether prothrombin time (PT-INR) and activated partial thromboplastin time (APTT) on admission correlate with mortality and organ failure. Therefore the aim of this study was to investigate if PT-INR and APTT at admission can predict outcome in the critically ill patient after adjusting for severity of illness measured with Simplified Acute Physiology Score 3 (SAPS 3).

**Methods:** Data was collected from our electronic journal systems. SAPS 3, PT-INR and APTT were independent variables in all regression analyses. Cox regression was used for the survival analysis. Organ failure was defined as the occurrence of renal failure based on Acute Kidney Injury Network (AKIN)-creatinine or need for; vasopressors, invasive ventilation or continuous renal replacement therapy (CRRT) the first 28 days after admission. Length of stay was only analysed in survivors.

**Results:** The study included 3585 unique patients. Prolonged APTT was associated with mortality with a 95% confidence interval (CI) of hazard ratio 1.001-1.010. Prolonged APTT correlated also with the occurrence of renal failure and the need for vasopressor and CRRT with 95% CI of odds ratio (OR) 1.009-1.028, 1.009-1.033 and 1.009-1.029 (Fig 2). Increased PT-INR was associated with the need for vasopressors and invasive ventilation with 95% CI of OR 1.138-2.056 and 1.151-1.871. Both APTT and PT-INR correlated with length of stay with 95% CI of OR 1.007-1.020 and 1.034-1.438.

**Conclusions:** Activated partial thromboplastin time on admission to the ICU is independently associated with mortality. Both APTT and PT-INR are independently associated with length of stay and the need of organ support. All regression models were adjusted for SAPS 3 score which means that APTT prolongation and PT-INR increase on admission represent morbidity that is not accounted for in SAPS 3.

**Fig. 1 (abstract P332).**
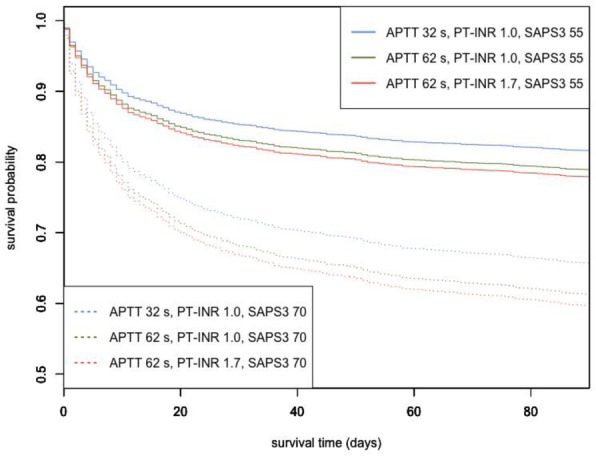
Survival probability of two fictive patients with SAPS 3 score 55 and 70, respectively. The mean SAPS 3 score was 61.0 points and standard deviation 16.1. APTT 32 s and PT-INR 1.0 are within the normal ranges. APTT 62 s is the 99th percentile and PT-INR 1.7 is the 95th percentile

**Fig. 2 (abstract P332).**
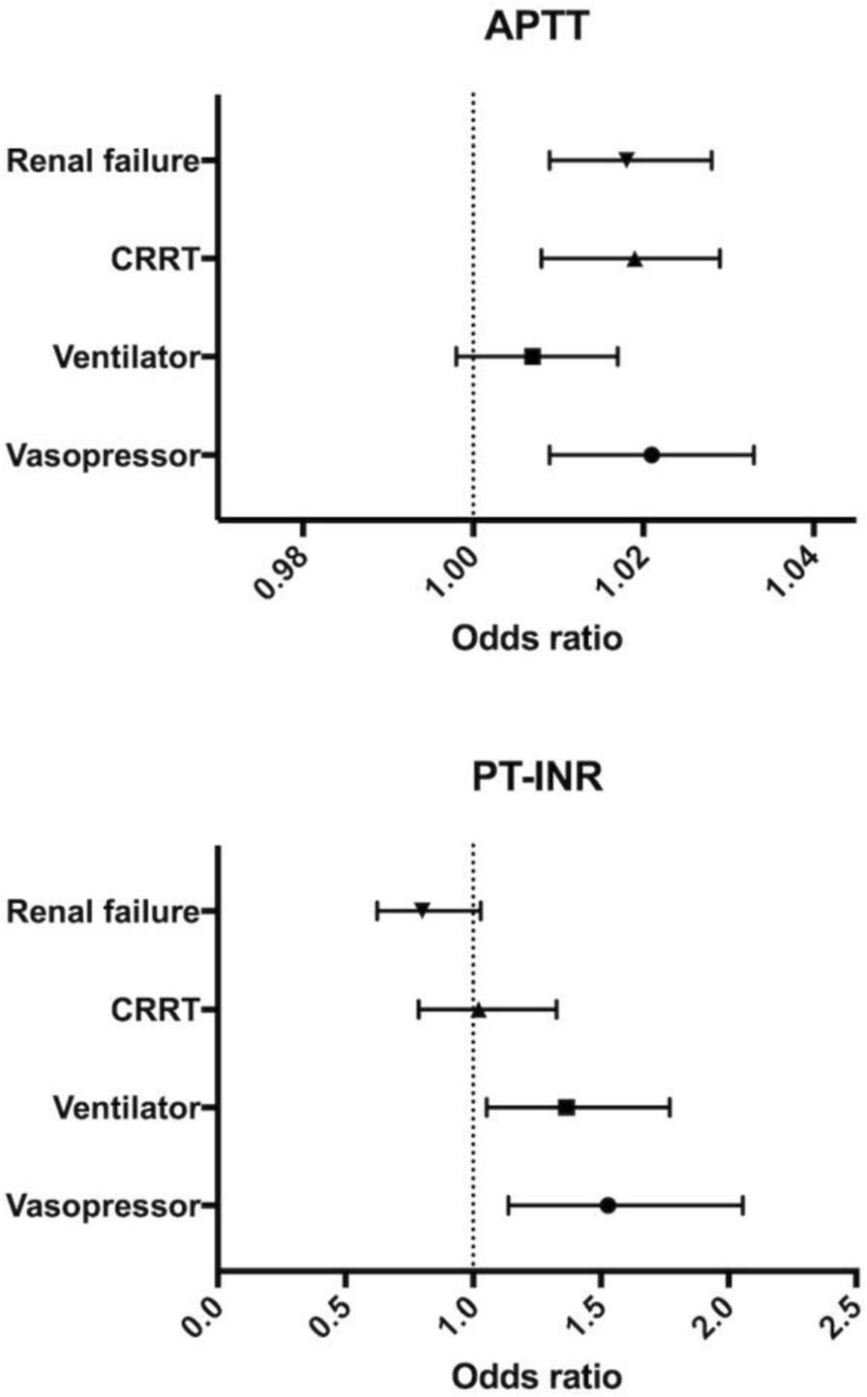
Odds ratio with 95% confidence intervals for APTT and PK-INR. All outcomes (Renal failure, CRRT, Ventilator and Vasopressor) were analyzed in separate multivariable regression models using APTT, PK-INR and SAPS3 as independent variables. Outcomes were measured the first 28 days after admission and were defined as: Renal failure: Occurrence of Acute Kidney Injury Network (AKIN)-creatinine class 1-3; CRRT (Continuous Renal Replacement Therapy; Ventilator: Days alive and free (DAF) of invasive ventilation; Vasopressor: DAF of vasopressors

### P333 A quality improvement project on daily venous thromboembolism risk assessment within critical care

#### E Castren, S Hutchinson

##### Norfolk & Norwich University Hospital, Critical Care Complex, Norwich, United Kingdom

**Introduction:** The goal was to assess if daily venous thromboembolism (VTE) assessment was being done in our critical care (CC) unit, and if not, what changes could be made. A mortality review showed the need for a dynamic VTE assessment in CC patients, who are subject to daily changes influencing VTE risk. A daily risk assessment was introduced, and a ‘tab’ on our clinical information system, Metavision(r)(MV) was created. Recently published National Institute for Health and Care Excellence guidelines on VTE risk assessment in CC provided us cause to assess our compliance [1].

**Methods:** Data was collected from MV. Review of daily VTE assessment was made and a percentage completion of daily VTEassessments was calculated per patient.Interventions were done using standard improvement methods through PDSA cycles.

**Results:** Baseline data, of 53 patients, was collected in July,2018.Compliance with daily VTE assessment was 51%. The results were presented at the clinical governance forum(CGF), and posters were displayed in CC. The second cycle, of 32 patients, was collected in October. Compliance had increased to 68%.Following discussion from presenting results at the CGF, the VTE tool was appropriately modified.The responsibility of VTE assessment was also shifted to becoming more shared, including all clinical staff, rather than mainly consultants. The third cycle, of 30 patients, was collected in November. Compliance had increased to 80%.Introducing a nursing care bundle with VTE is in progress.

**Conclusions:** Despite the identification of a risk in our clinical practice and the development of an appropriate IT tool to facilitate improved practice, the advent of new national guidance revealed poor compliance with agreed standards. This shows the difficulties with achieving practice change in complex multiprofessional clinical environments. A sustained effort is required focusing on dissemination and engagement across the whole team.

**Reference**

1. NICE (2018) Venous thromboembolism in over 16s (NICE Guideline 89)

### P334 Venous thromboembolism prophylactic dose Tinzaparin rarely achieves significant anti-Xa activity in critically ill patients but does affect thrombin generation

#### K Pates^1^, M O´Connor^1^, S Platton^2^, S Forbes^2^, J Prowle^1^, C Kirwan^1^

##### ^1^Royal London Hospital, Critical Care Unit, London, United Kingdom; ^2^Royal London Hospital, London, United Kingdom

**Introduction:** We describe the changes in anti factor Xa (aFXa) activity, thrombin generation and thromboelastography (TEG) in critically ill patients with and without acute kidney injury (AKI) following routine administration of Tinzaparin as part of venous thromboembolism (VTE) prophylaxis.

**Methods:** Pilot prospective observational study. 18 patients divided into those with and without AKI were administered Tinzaparin by subcutaneous injection as per established local guidelines. 6 patients who did not receive Tinzaparin were recruited as a ‘control’. Plasma aFXa activity and thrombin generation were measured at intervals over a 24 hour period. TEG parameters were collected at t0 and t24.

**Results:** aFXa activity: Results are shown in Figure 1. 3/18 patients failed to achieve a prophylactic aFXa level of >0.2 at any point. 14/18 patients achieved a level of >0.2 however in all cases this was at the lower end of the prophylactic range and was achieved for only a short time (median 1.5 hours). 1/18 achieved a level of >0.2 for the whole 24h period. There was no difference between the AKI and no AKI groups. Endogenous thrombin generation: there is no significant difference in thrombin generation between the AKI and no AKI groups. There is a significant decrease in thrombin generation between 0h and 5h (p<0.01) and a significant increase between 5h and 24h (p<0.01) (Figure 2). There is no significant difference between 0h and 24h (p=0.90). TEG: all TEG parameters for all patients were within normal range

**Conclusions:** Standard VTE prophylactic dose Tinzaparin rarely achieves an aFXa range that has been suggested for VTE prophylaxis. However, as assessed by thrombin generation, a hypo-coagulable state is generated in response to LMWH. There is no difference between critically ill patients with or without AKI that would suggest the need for dose reduction in this context.

**Fig. 1 (abstract P334).**
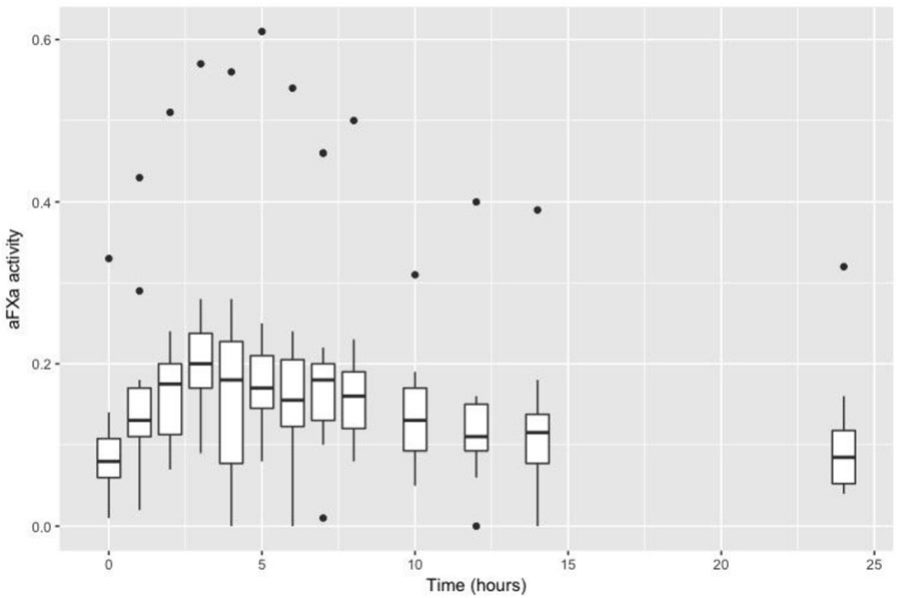
Box plot showing median aFXa activity throughout the study period of 24 hours for all 18 patients (AKI and no AKI). Time 0 = time of Tinzaparin administration, with the sample taken just prior to administration. Black circles represent outliers identified in analysis

**Fig. 2 (abstract P334).**
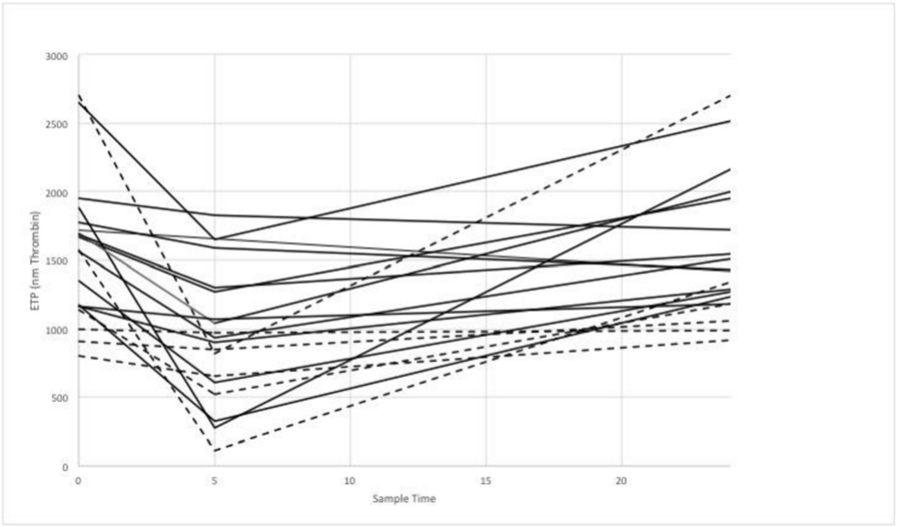
Thrombin generation at 0h, 5h and 24h. t0 = time of Tinzaparin administration, with the sample taken just prior to administration. Patients from AKI group shown with dotted line and from no AKI shown with solid line

### P335 Investigation of protein C and fibrin-monomer levels in children after cardiac surgery with cardiopulmonary bypass

#### V Lastovka, R Tepaev, O Gordeeva

##### National Medical Research Center for Children´s Health, Moscow, Russia

**Introduction:** Cardiopulmonary bypass (CPB) is assotiated witn thrombotic complications(TC). Occurrence of thrombosis after CPB is 12% which takes the third place between CPB-associated complications . Current data demonstrates the importance of researching of changes in haemostatic system in paediatric patiens after CPB. Provided below data is an intermediate result of our research.

**Methods:** 39 patients in age up to 11 mohth 29 days (median age – 5,5 months, youngest age – 2 days after birth, oldest – 11 months 29 days), who underwent cardiac surgery with CPB to treat congenital heart diseases, were enrolled in this study. All patients were divided into two groups: 1st – without TC, 2nd – with TC. Protein C (PC) and fibrin-monomer (FM) plasma levels were assessed in there points: before surgery, 24-hours and 72 hours after surgery. Thrombotic cases were provided by Doppler ultrasound or MRI.

**Results:** Thrombotic complications were diagnosed in 7 chidren (18%). Between all TC ischemic strokes were diagnosed in 57% (4 cases), arterial thrombosis in 29% (2 cases), intracardiac thrombus in 14 % (1 cases). In group with TC FM-mean values in points 1,2 and 3 respectively were 9.62; 37 and 108 mcg/ml, meamwhile in group without thrombosis – 7.5; 36.04 and 9.25 mcg/ml .PC-mean value in 1st group – were 50; 56 and 76%, in the 2nd group – 49; 52 and 39% respectively in the points 1, 2 and 3. Statistically significant differences between groups in 3rd point (p<0.05) and correlation between PC and FM (r=-0.93; p<0.05) were detected.

**Conclusions:** CPB causes hypercoagulation with increasing of PC consumtion and FM level. Moreover, CP associated with a high risk of TC on the 3rd day after cardiac surgery. Further studies to investigate prognostic values of FM and PC in thrombosis are required. These studies would help to asses FM and PC as markers of TC and possibility of PC-prescribing for prevention and treatment of these complications.

### P336 Sepsis-associated coagulopathy and the association of platelet count with mortality

#### A Waite^1^, Y Alhamdi^2^, R Iqbal^1^, N Venugopal^1^, C Toh^2^, S Abrams^2^, I Welters^3^

##### ^1^Royal Liverpool University Hospital, Liverpool, United Kingdom; ^2^University of Liverpool, Liverpool, United Kingdom; ^3^Royal Liverpool University Hospital, Intensive Care Unit, Liverpool, United Kingdom

**Introduction:** Thrombocytopenia is a common condition in critically ill patients and an independent predictor of mortality. The relevance of a supranormal platelet count remains unclear. Septic patients with Disseminated Intravascular Coagulation (DIC) are also known to have a high mortality, but the influence of sepsis on mortality rates in coagulopathic patients is less well characterised. Our objectives were to:

1. Evaluate mortality amongst patients with sepsis and non-sepsis associated DIC.
2. Assess incidence of DIC during the first 7 days of admission.
3. Assess the relationship between platelet count and mortality.

**Methods:** Records of 935 adult critical care patients admitted to the Royal Liverpool University Hospital between 2008-2014 were retrospectively reviewed. The presence of sepsis (using the definition of SIRS with infection), coagulopathy, degree of thrombocytopenia and 28 day mortality were noted. Modified ISTH DIC score was used to define DIC.

**Results:** The overall mortality rate was 19%. 522 patients were identified as having sepsis (56%) and 413 non septic patients (44%). Mortality rates of patients with sepsis were significantly higher than without sepsis (71% vs 29% respectively, p<0.0001). In patients with DIC, their DIC scores tended to be ‘positive’ for the first 4 days of admission. Fibrin-related markers were often not available for DIC scoring. Mortality rates amongst patients with sepsis-associated DIC were greater than patients with non-sepsis related DIC. Thrombocytopenia severity was associated with mortality, and patients with platelets above the upper limit of normal had lower mortality rates (11% when platelets > 400x10^9/L, 30% when platelets <50x10^9/L).

**Conclusions:** Sepsis-associated coagulopathy is associated with a higher mortality rate than non-sepsis associated coagulopathy. Supranormal platelet counts may be associated with a mortality benefit.

### P337 Comparison of hemostatic potential and analgesia methods of elderly patients who underwent major urological surgery during their stay in ICU

#### O Tarabrin, O Suslov, R Sukhonos, I Basenko, D Volodychev, P Tarabrin

##### Odessa National Medical University, Anaesthesiology and Intensive Care, Odessa, Ukraine

**Introduction:** Deep vein thrombosis (DVT) is a major problem in ICU and affects overall lethality. DVT is widespread complication in ICU, especially in elderly patients, when early activisation may not be achieved. Aim of this study is comparison of haemostatic potential and analgesia methods of elderly patients who underwent major urological surgery during their stay in ICU.

**Methods:** A cross-sectional study was employed. Participants were ≥70 y.o., underwent major urological surgery, have had normal initial hemocoagulation data (thromboelastography was performed to all of them), had received analgesia with epidural catheter or IV by opioids use and were treated in ICU >3 days due to non-coagulopathy states, were included. Data were collected from October 2017 till October 2018. The patients were examined with thromboelastograph “Mednord” for thromboelastogramm (TEG) and with eSaote USG for thrombi occurrence in lower limb deep veins. The anticoagulants were prescribed under the ESA guidelines 2017.

**Results:** Participants (n=30) were divided in two groups - non-opioid analgesia with epidural catheter (n=12) and opioid analgesia (n=18). We received moderate decrease in anticoagulants dosage to the patients with epidural analgesia with the same TEG goals compared to the patients with opioid analgesia. Other factors as comorbidities may provoke DVT events, but was not evaluated in this study. The DVT events were monitored by expert with the use of USG to locate thrombi in the vein.

**Conclusions:** Use of epidural catheter analgesia provides moderate decrease of anticoagulants dosage compared to opioid analgesia patients; however strict control of TEG data must be presented. Comorbidity need to be monitored for early detection and prevention of DVT events.

### P338 Utility of low- frequency piezoelectric thromboelastography (LPTEG) for comparison of mono- and combined antithrombotic therapy results in patients with morbid obesity

#### O Tarabrin^1^, R Sukhonos^1^, O Suslov^1^, D Volodychev^1^, S Vorotyntsev^2^, P Tarabrin^1^

##### ^1^Odessa National Medical University, Anaesthesiology and Intensive Care, Odessa, Ukraine; ^2^Zaporizhzhya State Medical University, Anaesthesiology and Intensive Care, Zaporizhi, Ukraine

**Introduction:** Patients with morbid obesity (MO) have a high risk of thromboembolic events. In patients with a BMI >35, the hypercoagulable state is due to impairment of all parts of the blood coagulation as well as anticoagulation mechanisms by obesity.

**Methods:** The hemostasis system was studied in 100 patients with a BMI> 35 kg/m2 with various pathologies that were admitted to ICU. All patients were divided into 2 groups depending on the type of therapy: 1 group (n=50) received monotherapy with Enoxaparin sodium 0.1% 0.2 ml SC 2 times a day every 12 h; group 2 (n=50) received combination therapy with Enoxaparin sodium 0.1% 0.2 ml SC 2 times a day every 12 h and Pentoxifylline 100 mg 2 times a day every 12 h. To study the hemostasis system, we used LPTEG immediately after hospitalization, on 1, 3, 5 days.

**Results:** In both groups, prior to treatment: Contact Coagulation Intensity (ICC) was increased by 23.57%, Intensity of coagulation drive (ICD) - by more than 32.68%, clot maximum density (MA) - by 74.52%, index of retraction and clot lysis (IRCL) - 91.18% above normal. Patients of the 1st group: ICC increased by 12.62%, ICD was close to normal values, MA increased by 18.63%, IRCL was increased by 31.17%. Patients of the 2nd group on the 5th day: ICC decreased by 15.22% compared with the norm; the coagulation and fibrinolysis parameters were close to normal values and the decrease in fibrinolysis activity reaches to normal.

**Conclusions:** Combined therapy of thromboembolic complications in patients with obesity Sodium Enoxaparin sodium and Pentoxifylline is more effective than Enoxaparin sodium monotherapy because it affects all parts of the hemostatic system.

### P339 Role of point-of-care ultrasound (POCUS) airway in blunt neck trauma

#### A Azma Haryaty, O Adi, R Ismail

##### Hospital Raja Permaisuri Bainun, Emergency & Trauma, Ipoh Perak, Malaysia

**Introduction:** A laryngeal injury secondary to blunt neck trauma can lead to life-threatening upper airway obstruction [1,2]. Ultrasound enables us to identify important sonoanatomy of the upper airway [3]. The purpose of this report is to discuss role of POCUS airway in blunt neck trauma and to determine airway management based on standard Schaefer 5 subgroups classification.

**Methods:** Three cases of blunt neck trauma presented to our centre with either subtle or significant clinical signs and symptoms. Standard airway management was performed prior to POCUS airway using 15MHz linear transducer and it findings were later compared to flexible fibreoptic laryngoscopy and Computed Tomography (CT).

**Results:** POCUS airway had identified one out of 3 cases to have Schaefer 2 and the remaining as Schaefer 3. All POCUS airway findings were confirmed with flexible fibreoptic laryngoscopy and CT scan (Figs 1, 2). Based on Schaefer, supportive care and early steroid administration are advisable for group 1 and 2. For groups 3 to 5, immediate open surgical repair is deemed necessary due to extension of injuries.All cases were intubated using Glidescope.All including those presented with Schaefer 3 were managed conservatively and discharge well with proper follow-up.

**Conclusions:** Upper airway ultrasound is a valuable, non-invasive and portable for evaluation of airway management even in anatomy distorted by pathology or trauma. An organised approach using POCUS airway as an adjunct can expedite care and prevent early and long term complications in facilities without flexible laryngoscope and CT.

**References**

1. Atkins BZ et al. J Trauma 56:185Y190,2004.

2. Schaefer SD. Arch Otolaryngol Head Neck Surg.1992 118:598-604.

3. Osman A and Sum KM. J Intensive Care 2016;4:52

**Fig. 1 (abstract P339).**
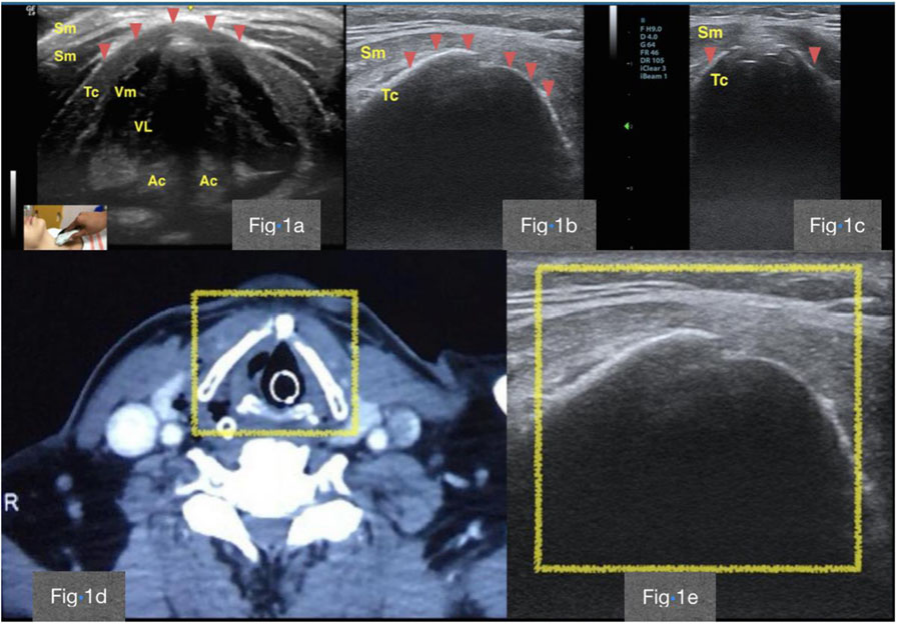
Figure 1a: Normal sonoanatomy in transverse scan. Figure 1b: Discontinuity of anterior cortex of thyroid cartilage with surrounding tissue oedema in transverse plane. Figure 1c: POCUS image of tranverse section of airway showing air mucosa disruption with a displaced thyroid cartilage. Sm-sternocleidomastoid muscle; Tc-thyroid cartilage; Vm-vocalis muscle; VL-vocalis ligament; AC- arytenoid cartilage. Figure 1d: A Computerised Tomography (CT) scan image shown defect in posterolateral wall of trachea with fracture of right anterior lamina of thyroid cartilage and superior cornu of left thyroid cartilage. Figure 1e: Figure of POCUS airway of thyroid cartilage in relation to the CT scan image

**Fig. 2 (abstract P339).**
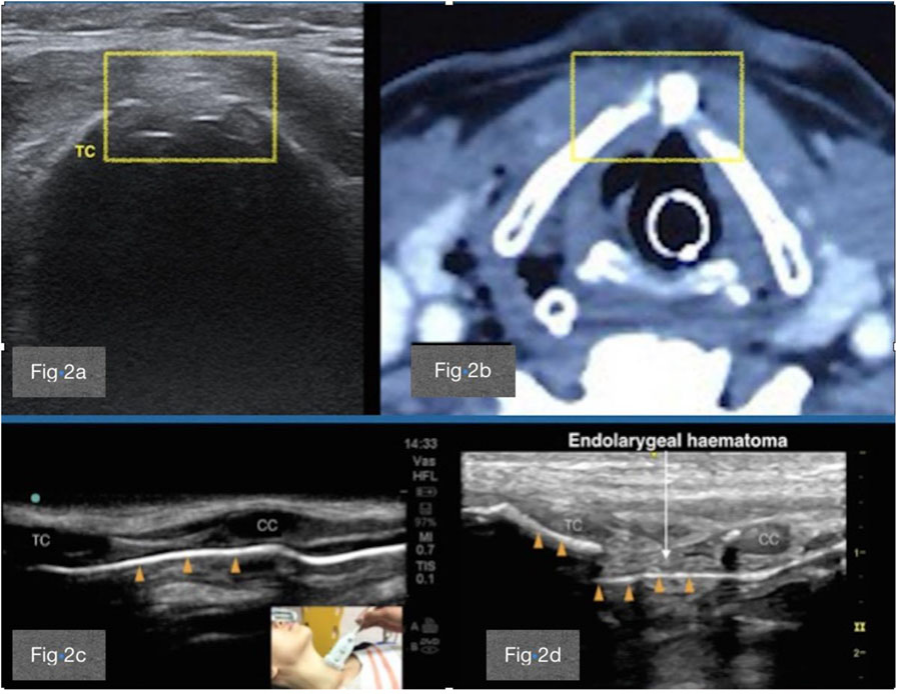
Figure 2a: Figure of POCUS airway of displaced thyroid cartilage fracture and disruption of air mucosal. Figure 2b: A Computerised Tomography (CT) scan image shown defect in posterolateral wall of trachea with fracture of right anterior lamina of thyroid cartilage and superior cornu of left thyroid cartilage in relation to the image in POCUS. TC- thyroid cartilage. Figure 2c – Normal sonoanatomy in longitudinal scan showing continuous intact air mucosal as shown by yellow arrowhead. Figure 2d- POCUS airway in longitudinal scan showing disruption of air mucosal interface and displaced thyroid cartilage as shown by yellow arrowhead and formation of endolaryngeal haematoma. TC – thyroid cartilage; CC- cricoid cartilage

### P340 Preliminary report: a randomized controlled trial comparing helmet continuous positive airway pressure (CPAP) vs high flow nasal cannula (HFNC) for treatment of acute cardiogenic pulmonary oedema in the emergency department

#### O Adi^1^, S Kai Fei^1^, A Azma Haryaty^1^, M Mohamed Sakan^2^

##### ^1^Hospital Raja Permaisuri Bainun, Emergency & Trauma, Perak, Malaysia; ^2^Hospital Raja Permaisuri Bainun, Emergency Medicine, Perak, Malaysia

**Introduction:** High Flow Nasal cannula(HFNC) is a new modality in respiratory failure management [1]. This study objectively held to compare the physiological outcomes in the non-invasive ventilation(NIV) treatment of cardiogenic acute pulmonary oedema(APO) patient in the emergency department(ED) delivered by helmet CPAP(hCPAP) and HFNC.

**Methods:** Single-centre randomized controlled trial on patients presenting with cardiogenic APO. Primary endpoint was a heart rate reduction.Secondary endpoints included: improvement in subjective dyspnoea scales, respiratory rate, blood oxygenation, intubation rate and 28 days mortality rate.

**Results:** 188 patients were enrolled and randomized (94 patients to hCPAP; 94 to HFNC) (Table 1). Both intervention reduced heart rate (from 111.80±14.87 to 91.93±13.67 bpm and from 110.45±13.84 to 95.27±13.52 bpm), respiratory rate (from 34.93±5.86 to 23.00±3.61 brpm and from 33.36±5.59 to 24.51±3.69 brpm) and improved dyspnoea measure by both Visual Analog Scale(VAS) (from 20.43±11.91 to 60.53±18.97 and from 21.70±10.64 to 55.43±16.95) and Likert scale (Table 2). Both NIV methods improved arterial oxygenation, PaO2 (from 87.26±21.36 mmHg to 176.52±41.11 mmHg in hCPAP and from 91.37±36.54 to 163.10±44.18 mmHg in HFNC), PaO2:FiO2 ratio (from 145.43±35.60 to 294.19±68.52 and from 152.27±60.89 to 271.83±73.63). Intubation rate was lower in hCPAP (6.9% for hCPAP versus 10.48% for HFNC) and 28 days mortality rate is lower in hCPAP (9.6% for hCPAP versus 14.9% for HFNC).

**Conclusions:** Both hCPAP and HFNC significantly improved patient condition in patient presenting to the ED with cardiogenic APO. However, hCPAP was better than HFNC in improving physiology outcomes, lower intubation rate and mortality rate in patient

**Reference**

Patel BK et al. JAMA 2016, 315:2435-2441.

**Table 1 (abstract P340).**
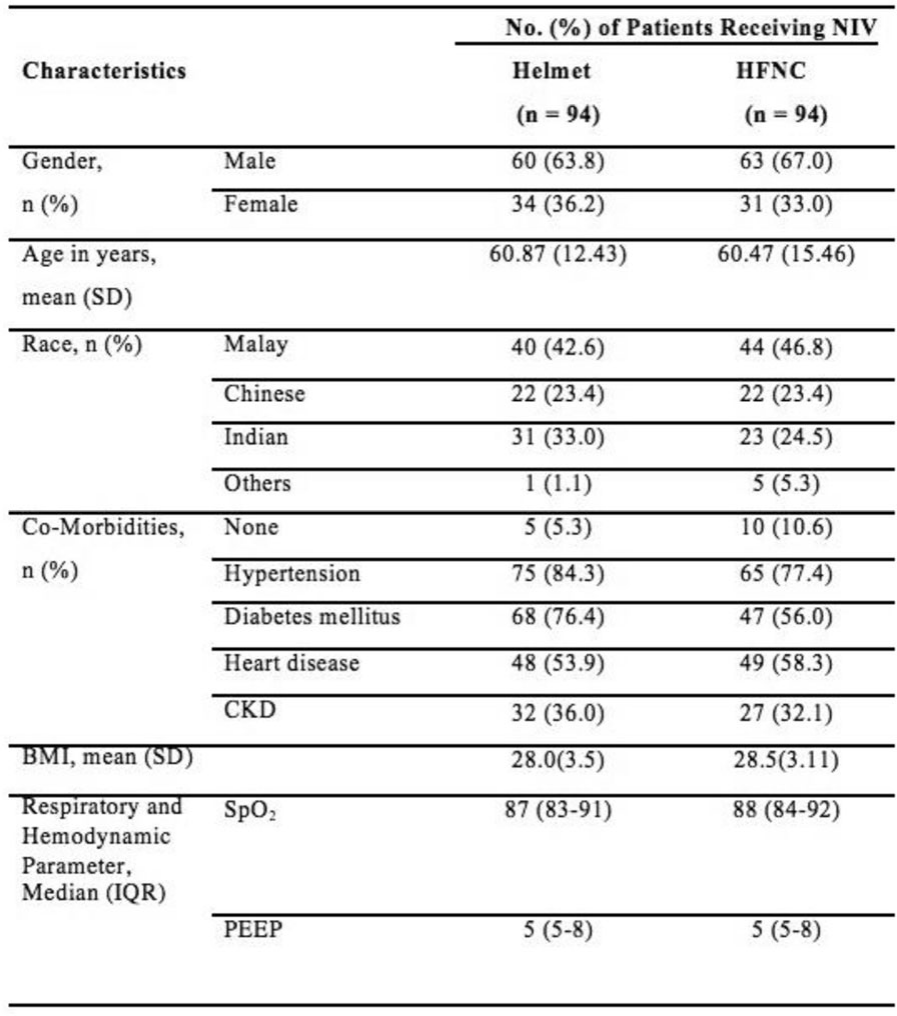
Characteristics of Patients at Baseline

**Table 2 (abstract P340).**
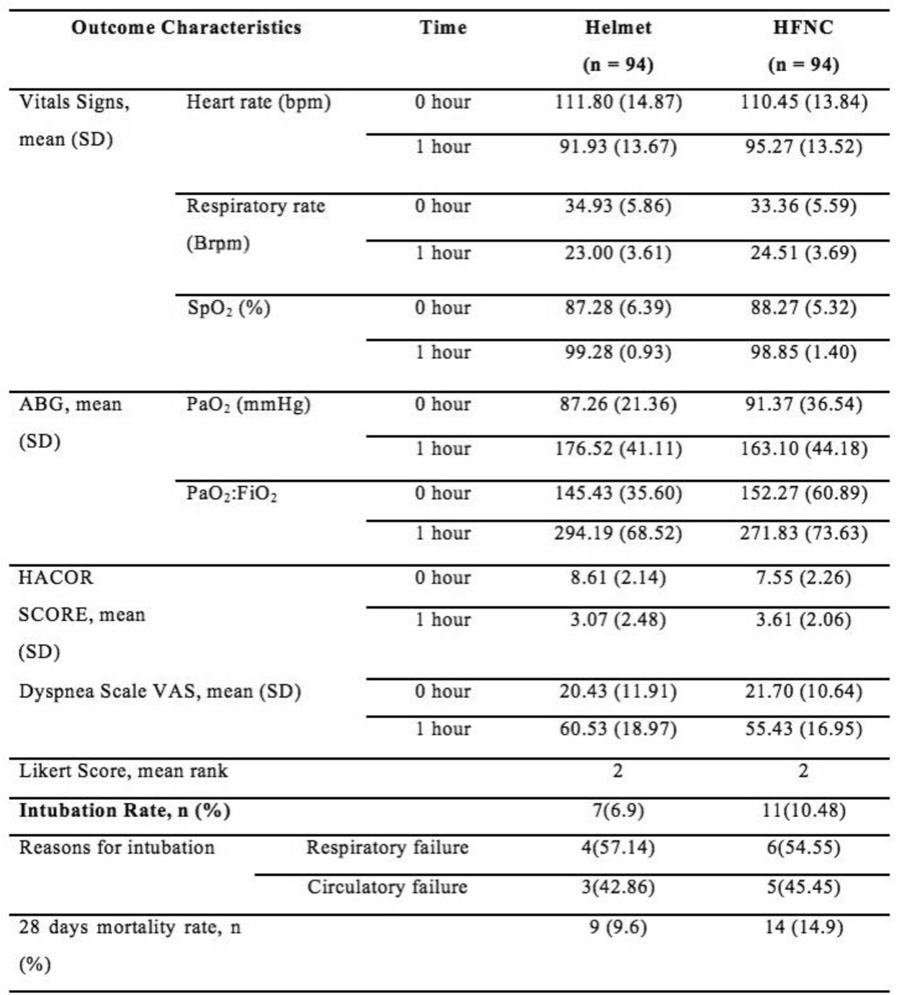
Primary and secondary outcome

### P341 Helmet non-invasive ventilation versus high flow nasal oxygen in acute hypoxemic respiratory failure: physiological effects

#### LS Menga, V Raggi, F Bongiovanni, DL Grieco, D Eleuteri, G Bello, S D´Arrigo, G Mercurio, E Tanzarella, GM Anzellotti, MA Pennisi, M Antonelli

##### Fondazione Policlinico Universitario A. Gemelli IRCCS, Department of Anesthesiology and Intensive Care Medicine, Rome, Italy

**Introduction:** High-flow nasal oxygen (HFNO) and helmet non-invasive ventilation (hNIV) are increasingly used for the early management of acute hypoxemic respiratory failure (AHRF). We compared the physiological effects of HFNO and hNIV during AHRF.

**Methods:** In this randomized cross-over study, we enrolled patients with acute-onset (<7 days), non-cardiogenic respiratory distress (respiratory rate>25/min), pulmonary infiltrates at the chest-x-ray and hypoxemia (SpO2<90% while breathing on room air). All patients received hNIV (PEEP 10 cmH2O, pressure support adjusted to achieve a peak inspiratory flow of 100 L/min) and HFNO (flow 50 L/min) for one hour each, in a randomized cross-over manner. At the end of each period, arterial blood gases, inspiratory effort (esophageal pressure) and respiratory rate were recorded. Self-assessment of dyspnea and device-related discomfort (visual analog scale [VAS], ranging from 0 to 10) were performed. Results are expressed as medians [interquartile range].

**Results:** Ten patients were included: median age was 70 years [61-77], 4 (40%) women, median SOFA 7 [3-10]. Baseline PaO2/FiO2 ratio during VenturiMask oxygen therapy was 140 [105-186] mmHg. PaO2/FiO2 was significantly higher during hNIV than during HFNO (264 mmHg [217-298] vs.149 mmHg [109-174]; mean difference 108 mmHg [95%CI, 72 to 144]; p=0.005). During hNIV, patients had lower inspiratory effort (esophageal swing 11 cmH2O [8-19] vs. 7 cmH2O [3-11], mean difference -6 cmH2O [CI 95%, -9 to -3]; p=0.005) and lower dyspnea (VAS 8 [5-10] vs. VAS 4 [2-6], mean difference VAS -3 [-5.4 – 0.7]; p = 0.02). No significant differences between treatments were observed in PaCO2 (31 mmHg [28-35] vs. 31 mmHg [28-39]), respiratory rate (27/min [23-31] vs. 23/min [21-30]) and discomfort (VAS 6 [3-9] vs VAS 6 [3-7]).

**Conclusions:** As compared to HFNO among critically ill patients with AHRF, hNIV ameliorates oxygenation, limits inspiratory effort and relieves dyspnea, without affecting PaCO2, respiratory rate and comfort.

### P342 The intubation first pass success for patients with hypoxemia in emergency department

#### N Miyauchi^1^, H Okamoto^1^, T Goto^2^, Y Hagiwara^3^, H Watase^4^, K Hasegawa^5^

##### ^1^Kurashiki Central Hospital, Emergency medicine, Okayama, Japan; ^2^University of Fukui, School of Medical Sciences, Fukui, Japan; ^3^Tokyo Metropolitan Children´s Medical Center, Pediatric Emergency and Critical Care Medicine, Tokyo, Japan; ^4^University of Washington, Surgery, Seattle, Washington, United States; ^5^Massachusetts General Hospital, Emergency Medicine, Boston, Massachusetts, United States

**Introduction:** Pre-intubation hypoxemia is a predictor of negative patient outcomes including in-hospital mortality. While successful first intubation attempt is also an important factor of patient outcomes, little is known about whether physicians achieve successful first intubation attempt for the hypoxemic patients in the emergency department (ED). The aim of this study is to investigate the first-pass success for patients with pre-intubation hypoxemia in the ED.

**Methods:** This is an analysis of the data from the second Japanese Emergency Airway Network study (JEAN-2 study) – a multicenter, prospective, observational study of 15 EDs in Japan. We included all patients who underwent intubation in the ED from 2012 through 2018. We excluded patients 1) aged <18 years and 2) patients who underwent intubation for cardiac arrest. We grouped pre-intubation hypoxemia as follows: non-hypoxemia (oxygen saturation [SpO2], ≥97%), moderate-hypoxemia (SpO2, 96%-90%), and severe-hypoxemia (SpO2, <90%). Primary outcome was the first-pass success rate. To demonstrate the association between pre-intubation hypoxemia and the first-pass success in the real-world setting, we fit two unadjusted logistic regression models 1) using grouped pre-intubation hypoxemia as a categorical variable and 2) using the pre-intubation SpO2 as a continuous variable.

**Results:** Among 10,144 patients who underwent intubation in the ED (capture rate, 97%), 5,463 patients were eligible for the analysis. Compared to the non-hypoxemia, the first-pass success rate was low in moderate-hypoxemia (72% vs 67%; OR=0.77 [95%CI, 0.67-0.89]) and severe-hypoxemia (72% vs 69%, OR=0.85 [95%CI, 0.72-0.99]). Additionally, there was a linear association between pre-SpO2 and lower first-pass success rate (OR for the success, per one pre-SpO2 decrease, 0.99 [95%CI, 0.98-0.99]).

**Conclusions:** Based on the large, multicenter data, the first-pass success rate was low in hypoxemic patients compared to non-hypoxemic patients in the ED.

### P343 Introduction of rapid-sequence induction guideline to reduce drug-associated hypotension in critically unwell patients

#### C Flanders^1^, O Francis^2^, B Clarke^2^, J Service^1^, CA Walker^1^

##### ^1^NHS Lothian, Intensive Care Unit, Livingston, United Kingdom; ^2^NHS Lothian, Emergency Department, Livingston, United Kingdom

**Introduction:** The aim of this project was to assess whether the introduction of a Rapid Sequence Induction (RSI) agent guideline changed drug choice and the incidence of peri-intubation vasopressor use at St John’s Hospital, Livingston. It is well documented that emergency airway management in the critically ill can be a source of significant morbidity and mortality [1, 2] and the choice of induction agent matters [3].

**Methods:** An RSI agent guideline was instituted for all critically ill patients being intubated in ICU and the ED [Figure 1]. Following this, we set up an intubation registry to collect data from all intubation events. This data was then compared to a previous audit of intubations completed in 2012.

**Results:** The choice of agent used pre- and post-intervention are summarized in Figure 2. Forty-five intubation events were included in the initial audit in 2012, of which, 7 (16%) required vasopressor support immediately following intubation. Of the 41 intubation events following the guideline’s introduction, 2 (5%) required vasopressors. Ketamine use changed from 0% to 51%, Propofol use from 43% to 29% and Midazolam from 14% to 0%. Thirty-eight of these intubation events (93%) were compliant with the guideline.

**Conclusions:** The introduction of the RSI guideline dramatically affected the choice of induction agent and reduced the incidence of significant hypotension requiring vasopressors (5% versus 16%). Overall compliance with the guideline was excellent (93%).

**References**

1. Simpson GD et al, British Journal of Anaesthesia, 108: 792-9, 2012

2. Cook TM et al, British Journal of Anaesthesia, 106: 617-631, 2011

3. Stollings JL et al, Annals of Pharmacotherapy 48: 62-76, 2013

**Fig. 1 (abstract P343).**
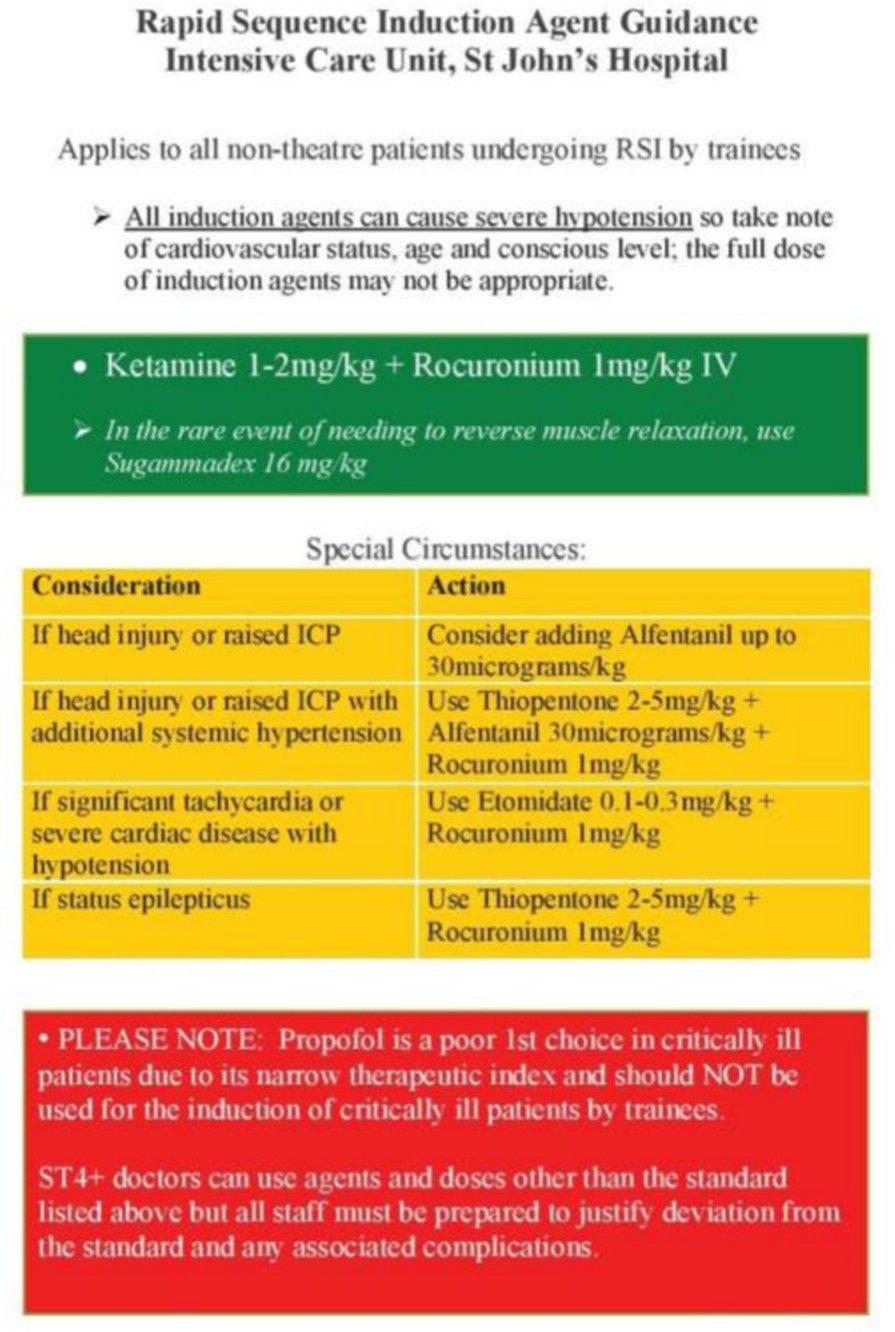
RSI Agent Guideline

**Fig. 2 (abstract P343).**
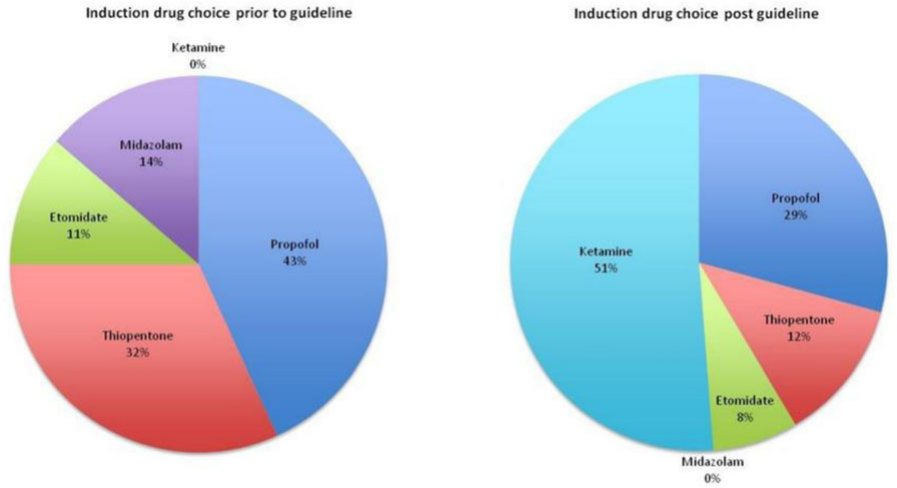
Induction agents used

### P344 Use of I-Gel® facilitates percutaneous dilatational tracheostomy in the ICU. A preliminary report

#### MJ Sucre, A De Nicola

##### Dept Anaesthesia and Intensive Care, Castellammare di Stabia, Italy

**Introduction:** The purpose is to test the feasibility of using the I-Gel® device for airway maintenance during bronchoscopic-guided percutaneous dilatational tracheostomy (PDT). Usually PDT is accomplished via the tracheal tube. Failure to position the endotracheal tube correctly can result in further complications during the procedure. The alternative implies extubation and reinsertion of an I-Gel® airway device.

**Methods:** The PDT was performed using the Blue Dolphin method in 5 patients in intensive care unit. Before undertaking bronchoscopic-guided percutaneous dilatational tracheostomy (PDT), the patient’s tracheal tube (ET) was exchanged for I-Gel®, as a ventilatory device for airway maintenance. The insertion of the I-Gel®, the quality of ventilation, the blood gas values, the view of the tracheal puncture site, and the view of the balloon dilatation were rated as follows: very good (1), good (2), barely acceptable (3), poor (4), and very poor (5) [1].

**Results:** The I-Gel® successfully maintained the airway and allowed adequate ventilation during percutaneous tracheostomy in all patients. The ratings were 1 or 2 in 100% of cases with regards to ventilation and to blood gas analysis, for identification of relevant structures and tracheal puncture site, and for the view inside the trachea during PDT.

**Conclusions:** The I-Gel® successfully maintained the airway and allowed adequate ventilation during percutaneous tracheostomy in all patients. The ratings were 1 or 2 in 100% of cases with regards to ventilation and to blood gas analysis, for identification of relevant structures and tracheal puncture site, and for the view inside the trachea during PDT. No damages to the bronchoscope, reports of gastric aspiration or technical problems were detected. The bronchoscopic view obtained via an I-Gel® seems to be better than that obtained through an endotracheal tube (ET) or through traditional laryngeal mask [1].

**Reference**

1) De Nicola A et al. Crit Care. 2013; 17(Suppl 2): P169

### P345 Comparison of the efficiency of oral airway and nasal airway inserted in the oral airway during mask ventilation

#### B Kim, S Park, J Kim, Y Park

##### Seoul National University Bundang Hospital, Department of Anesthesiology and Pain Medicine, Gyeonggi-do, South Korea

**Introduction:** The purpose of this study was to investigate the efficiency of nasal airway inserted in the oral airway (ON airway) in securing the airway patency during mask ventilation [1] (Fig 1).

**Methods:** Fifty eight patients undergoing general anesthesia were randomly assigned to either oral airway group (group O) or ON airway group (group N). In both group, 2 mg/kg of propofol was infused intravenously and mask ventilation was performed in the sniffing position without head extension or jaw thrust. The patients were ventilated with a volume-controlled ventilator with O2 flow of 10 l/min, tidal volume of 10 ml/kg (IBW), and respiratory rate of 10 /min. Before the start of mask ventilation, airway was placed in the oral cavity. Oral airway was used in group O and ON airway was used in group N. Peak inspiratory pressure (PIP), tidal volume and EtCO2 were compared between the two groups. The location of airway tip was graded by fiberoptic bronchoscope as; 0: airway obstructed by tongue, 1: epiglottis visible, 2: airway touches epiglottis tip, 3: airway passes beyond epiglottis tip [2].

**Results:** Compared with group O, group N significantly decreased the PIP (25.0 [18.0 – 29.0] vs. 18.0 [16.0 – 19.0], P < 0.001, Group O and N, respectively) and increased tidal volume and EtCO2 during mask. In the bronchoscopic findings, airway obstruction was more frequent in group N (P< 0.001).

**Conclusions:** Compared with oral airway, nasal airway inserted in the oral airway facilitates the mask ventilation by promoting the patency of airway.

**References**

1. Liang Y et al (2008) Anesthesiology 108: 998-1003.

2. Kim S et al (2014). Anaesthesia 69: 53-57.

**Fig. 1 (abstract P345).**
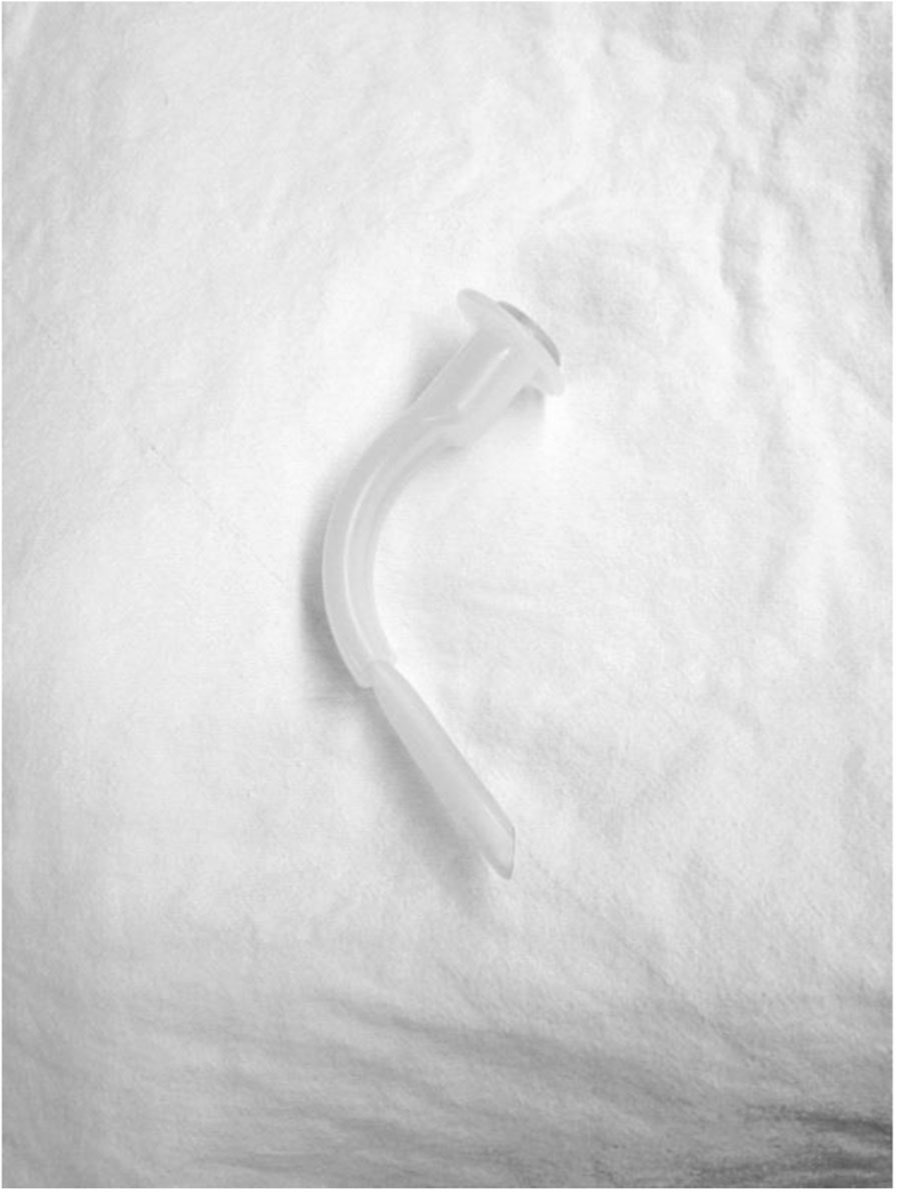
Nasal airway inserted in the oral airway

### P346 Outcome improving in patients with spinal muscle atrophy

#### V Artemenko, E Plotnaya

##### MEDICAP, Anesthethia&ICU, Odessa, Ukraine

**Introduction:** In Ukraine, 150 pts with Spinal Muscle Atrophy(SMA-pts.) are registered. The weakness of respiratory muscles leads to their ICU admission, intubation, followed with tracheostomy. They can’t leave ICU. ICU LOS is about 823 days. Other option is extubation according to the Dr J R. Bach protocol (Dr. Bach pr). Our purpose: to evaluate the efficacy and possibility to implement Dr. Bach pr. in Ukraine.

**Methods:** A prospective uncontrolled observational study in 2017-18 in 4 Ukrainian hospitals. 10 SMA-pts from 6-18 mo were involved. All pts. ready for extubation: afebrile, no infiltrations on chest x-ray, normal WBC. However, each SMA-pts. failed SBT (T-tube or PSV). We evaluated: extubation success (no reintubation in 48 hours), ICU LOS, one year survival. Three pts. were excluded: two pts. by staff decision, 1 family have choosen tracheostomy. 7 SMA-pts. included. A cuff leakage test performed - with a negative, dexamethazone 1mg IV was administered. After extubation NIV was started by Ventilogik LS in ST mode via nasal mask Giraffe. The EPAP and IPAP settings were titrated to reach the chest excursion and target levels of SpO2 (92-96%) and EtCO2 (40-45 mmHg). A sputum was draining by mechanical insufflation-excuflation (MIE) and aspirator

**Results:** All pts, were extubated successful. The mean ICU LOS was 8.5 days (7-10 days), one year survival rate was 100%, respiratory failure fully compensated by NIV, there was no ICU admission. Every SMA-pts. are in good condition, gaining weight

**Conclusions**

1. Dr. Bach pr. allowed successful extubation in 100% of SMA-pts.
2. Dr. Bach pr implementation in the country could reduced ICU LOS.
3. Furure implementation of method is necessary in SMA-pts in Ukraine

### P347 Aerosol delivery during simulated high flow nasal therapy supplied by a mechanical ventilator

#### G Bennett, P Brady, M Joyce, R MacLoughlin

##### Aerogen, Science, Galway, Ireland

**Introduction:** Aerosol delivery has previously been assessed during simulated adult HFNT, delivered by various stand-alone humidification systems [1]. The objective of this study was to evaluate aerosol delivery during simulated HFNT delivered by a mechanical ventilator, across three clinically relevant gas flow rates.

**Methods:** 2ml of 2 mg/ml salbutamol was nebulised using an Aerogen Solo nebuliser (Aerogen, Ireland). An adult head model was connected to a breathing simulator (ASL5000, Ingmar, US), Vt 500 ml, BPM 15 and I: E, 1:1 (Fig 1). HFNT was supplied via the Servo-U ventilator (Maquet, Getinge, Sweden), using the integrated nebulisation option. Tracheal dose was recorded at two nebuliser positions; A (after the humidification chamber) or B (before of the cannula), at three gas flow rates (10LPM, 30LPM and 60LPM) (n=3). The mass of drug captured on a filter placed distal to the trachea (tracheal dose) was quantified using UV spectroscopy at 276 nm.

**Results:** Presented in Table 1.

**Conclusions:** To our knowledge, this is the first study to successfully demonstrate aerosol delivery during simulated HFNT, delivered by a mechanical ventilator. Increasing gas flow rate was associated with a reduced tracheal dose (p= <0.0001). At 10LPM, a significantly greater tracheal dose was observed when the nebuliser was positioned before the nasal cannula (p= <0.0001). At 60LPM, a greater tracheal dose was yielded when the nebuliser was positioned after the humidifier (p= <0.0001).

**Reference**

[1] Reminiac et al. JAMPDD. 2016; 29(2):134-41.

**Fig. 1 (abstract P347).**
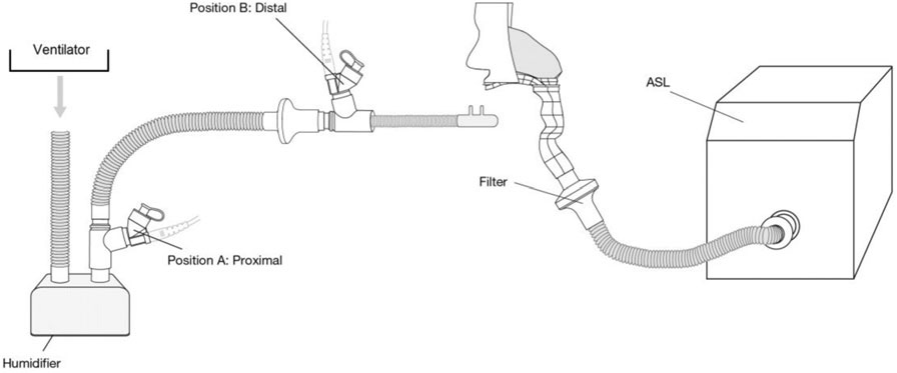
Experimental set-up

**Table 1 (abstract P347).** Mean ± standard deviation values of tracheal dose (%)

Flow rate	Tracheal dose (%) - A	Tracheal dose (%) - B	P-value
10LPM	16.80 ± 1.16	31.40 ± 0.75	<0.0001
30LPM	5.60 ± 0.77	5.72 ± 0.53	0.8309
60LPM	2.19 ± 0.04	0.91 ± 0.08	<0.0001
P-value	<0.0001	<0.0001	N/A

### P348 Effect of percutaneous and surgical tracheotomy on thyroid hormone levels

#### G Koukoulitsios^1^, K Tsikritsaki^1^, S Antonopoulou^1^, C Mandila^1^, E Papadimitriou^1^, D Toumpanakis^1^, D Belesiotis^1^, I Tsoni^1^, S Bakouli^1^, M Anifanti^1^, A Marinou^2^, AC Sampani^3^, D Papageorgiou^3^, A Kalogeromitros^1^

##### ^1^G. Gennimatas General Hospital, ICU, Athens, Greece; ^2^G. Gennimatas General Hospital, ENT, Athens, Greece; ^3^University of west Attica, ICU, Athens, Greece

**Introduction:** Tracheotomies are often performed in critically ill patients who are in need of prolonged mechanical ventilation and respiratory care. Our aim was to evaluate the possible effect of percutaneous and surgical tracheotomies on thyroid hormone levels.

**Methods:** Eighty seven adult patients were included in our study from January 2017 to September 2018. Patients were in need of prolonged mechanical ventilation and tracheotomies were performed after consent was taken. We have excluded patients with preexisting thyroid diseases. Forty five patients were undergone percutaneous tracheotomies and forty two patients were undergone for surgical. Thirty eight female patients and forty nine male, age range 18-85. We studied TSH, T3 and FT4 serum levels using Chemiluminescence Immunoassay method before either procedure and 2 hours post each procedure.: Statistical analysis was performed using SPSS 15. Significance was estimated at the level of p< 0.005

**Results:** TSH levels were increased in surgical group compared to percutaneous group at 2 hours post procedure but the difference was not found statistically significant (p> 0.005). The rise in post operative levels of T3 compared to preoperative was found statistically significant for surgical tracheotomy group (p< 0.005).Elevated FT4 levels for both groups have shown statistically significant difference between preoperative and postoperative period for the surgical tracheotomy group (p< 0.005)

**Conclusions:** We analyzed the effect of surgical versus percutaneous tracheotomy on thyroid hormones and it was found that both procedures may affect the level of thyroid hormones, being statistical significant in the surgical group.

### P349 Early cuff deflation in tracheostomised patients requiring ventilatory support

#### J Callon^1^, C Lamont^1^, S Dyson^1^, L Poole^1^, I Welters^2^

##### ^1^Royal Liverpool University Hospital, Royal Liverpool University Hospital, Liverpool, United Kingdom; ^2^Royal Liverpool University Hospital, Intensive Care Unit, Liverpool, United Kingdom

**Introduction:** Tracheostomies performed during critical illness have a significant impact on patient comfort and care, as verbal communication is impaired until cuff deflation is feasible and speaking devices can be introduced. This 12-month retrospective audit aimed to determine whether trials of cuff deflation as early as possible decrease tracheostomy days and lead to timelier decannulation.

**Methods:** Tracheostomised patients admitted to the Royal Liverpool Intensive Care Unit between 26/10/17 and 26/10/18 were identified and their clinical notes audited for details of admission, tracheostomy indication, insertion, first cuff deflation and decannulation. Trials of early cuff deflation started on 26/04/18 and followed new local guidance for the use of speaking valves. Early cuff deflation was performed in patients who would not have been considered before due to ongoing ventilatory support. Patients with long term tracheostomies or those who were not decannulated after their ICU stay were excluded. Patients with tracheostomy insertion dates prior to the initiation of early trials of cuff deflation on 26/4/18 were deemed ‘pre-trial’ patients, and served for comparison with patients in whom early cuff deflation was performed.

**Results:** 78 tracheostomised patients were identified over the 12-month audit period. 30 patients were included (13 in the pre-trial group and 17 in the trial group). Between the two groups there was a significant decrease in the mean number of days between tracheostomy insertion and cuff deflation (p= 0.043), a significant decrease in the mean number of tracheostomy days (38±30 vs 16±9, p=0.015) and a significant decrease in the mean length of Intensive Care Unit (ICU) stay (45±28 vs 28±11, p=0.028).

**Conclusions:** Early cuff deflation may facilitate weaning from ventilatory support, is safe to perform, leads to earlier decannulation and decreases tracheostomy days and length of stay on ICU. The results of this small cohort study need to be followed up in larger multi-centre trials.

### P350 Surgical tracheostomy: experience of the procedure of choice in an intensive care unit

#### JF Martins^1^, I Coutinho^2^, E Sousa^1^, P Martins^1^

##### ^1^Centro Hospitar e Universitário de Coimbra, Intensive Care, Coimbra, Portugal; ^2^Centro Hospitar e Universitário de Coimbra, immunoallergology, Coimbra, Portugal

**Introduction:** Surgical tracheostomy (ST) plays an important role in clinical management of critically ill patients. Although percutaneous tracheostomy (PT) is the most frequent procedure, actually, there is no significant advantage between the two techniques.

**Methods:** Retrospective, descriptive and inferential trial of 116 patients, evaluating: age; sex; co-morbid conditions; reason for ICU admission; SAPS II, APACHE II and SOFA score; Postoperative (PO) ventilator-associated pneumonia (VAP); sedation, vasopressor support and mechanical ventilation (MV) duration; indication, timing and location of ST; ICU length of stay (LOS); in-hospital and 6-month mortality.

**Results:** A total of 116 patients, 69.8% (n=81) male, with mean age of 62.62 ± 16.18 years. The kind of admission was medical in 44.8% (n= 52), trauma in 24.1 % (n=28), Neurosurgical in 22.4 % (n=26) and PO in 8.6% (n=10). Acute respiratory failure (48.3%), Neurosurgery (22.4%) and Neurologic (17.2%) were the most frequent reasons for ICU admission. Time from beginning MV to perform ST was < 7 days in 15.5% (n=18), 7 to 21 days in 58.6% (n=68), 22 to 30 days in 21,6% (n= 25), > 30 days in 4.3% (n=5). The incidence of VAP was 28.4% (n=33) and the most common agent identified was Pseudomonas aeroginosa (n=8). ICU LOS was 26.5 days and in-hospital mortality was 52.0 days and 30.2%, respectively. There was a statistically significant relationship between duration of sedation and vasopressor support with timing for ST (p <0.001). Time from beginning MV to perform ST superior to 15 days was associated with higher in-hospital mortality (p=0.014).

**Conclusions:** We found that early tracheostomies could lead to shorter duration of sedation, vasopressor support and reduction of in-hospital mortality. Despite our practice, the approach of choice, ST or PT techniques, require adequate training, and should always be performed in the right patients and in the right time.

### P351 Impact of protocolised weaning on patients with a tracheostomy in a large teaching hospital intensive care unit

#### E Mackay, G Cornell, A Cruikshanks, AJ Glossop

##### Northern General Hospital, Intensive Care, Sheffield, United Kingdom

**Introduction:** Insertion of a tracheostomy for weaning purposes is associated with prolonged critical length of stay (LOS) and several adverse patient outcomes [1]. Previous work has suggested that protocolised weaning may reduce weaning times [2]. We aimed to assess the impact of protocolised weaning on LOS following introduction of a standardised weaning protocol in 2017.

**Methods:** Data was collected on critical care patients who had a tracheostomy inserted, before and after the introduction of a ventilator weaning protocol, for seven months in 2016 and 2017 respectively. The protocol prescribed twice daily periods of spontaneous breathing trials (SBT) of increasing duration. Data was collected on demographics (patient age, diagnosis, pre-tracheostomy ventilator management and indication for tracheostomy), post tracheostomy weaning practice (time to first SBT and first cuff down, percentage of days with ≥2 SBT) and clinical outcomes (total ventilator days, duration of tracheostomy and critical care LOS).

**Results:** A total of 15 patients in 2016 and 19 patients in 2017 were eligible for analysis. The mean age was 60 in both groups. The mean number of days ventilated prior to tracheostomy was 15 in 2016 and 16 in 2017. The mean number of extubation attempts was 0.47 in 2016 and 0.63 in 2017. Following introduction of a weaning protocol in 2017, a reduction in mean duration of tracheostomy (24 vs 18 days) and critical care length of stay (44 vs 38 days) were seen. There was also a greater proportion of tracheostomy ventilator days with ≥2 SBT (19% vs 44%), a reduction in days to first SBT (5.6 vs 3.4 days) and days to first cuff down (20.3 vs 11.2 days) seen.

**Conclusions:** Introduction of a standardised weaning protocol for patients with a tracheostomy in our unit has had a beneficial effect on several patient outcomes, notably duration of weaning and length of critical care admission.

**References**

1. Young D et al. JAMA 309: 2121–9, 2013

2. Blackwood B et al. BMJ 342: c7237, 2011

### P352 The effect of early extubation on postoperative delirium in liver transplantation recipients: a propensity score matching analysis

#### JY Park^1^, YJ Choi^2^

##### ^1^ Pusan National University Yangsan Hospital, Department of Anesthesia and Pain Medicine, Yangsan, South Korea; ^2^Ansan Hospital, Korea University College of Medicine, Department of Anesthesiology and Pain Medicine, Ansan, South Korea

**Introduction:** Delirium is a relatively frequent neurologic complication in liver transplantation (LT) recipients, which is an important cause of increased morbidity, mortality, extended ICU stay, and increased cost of medical care. Extubation of the endotracheal tube at an appropriate timing is an essential part of intensive care after LT, suggested to improve graft perfusion and systemic oxygenation, and thus decrease intensive care unit (ICU) stay and positively affect prognosis. The aim of this study was to compare the incidence of delirium between early and late extubation groups after LT.

**Methods:** Medical records from 247 patients who received LT from January 2010 to July 2017 in a single university hospital were retrospectively reviewed. Patients were divided into 2 groups: Those who underwent early extubation after LT (group E, n = 52) and those who underwent extubation within few hours of ICU admission after surgery (group C, n = 195). The data of patients´ demographics, perioperative management, and postoperative complications were collected. Early extubation was defined as performing extubation in the operating room after LT. A propensity score matching analysis was performed to minimize the effects of selection bias.

**Results:** Postoperative delirium occurred in 4/52 (7.69%) in group E and 30/195 (15.38%) in group C, respectively (P = 0.15). After propensity score matching, there was no difference in ICU stay (P = 0.96), time to discharge after surgery (P = 0.12), and incidence of delirium between groups (P = 1.00).

**Conclusions:** Although this study is retrospective in nature, limited by small sample size, early extubation did not affect the incidence of delirium after LT. Further prospective studies on this area are required.

### P353 Weight estimation and its impact on mechanical ventilation settings in Queen Elizabeth hospital intensive care unit

#### A Nasr, A Iasniuk, A Roshdy

##### Queen Elizabeth Hospital, ICU, London, United Kingdom

**Introduction:** Documented weight in the intensive care unit (ICU) can be the total, ideal, adjusted or predicted body weight (PBW). Lung protective ventilation depends on tidal volume (VT) delivery which is based on accurate calculation of patients´ weight [1]. The weight is most probably documented on admission to the ICU using estimation or one of many available equations. The aim of this study is to assess the documented versus the PBW and its impact on tidal volume delivery for mechanically ventilated patients in Queen Elizabeth Hospital ICU.

**Methods:** Data was collected prospectively from all ventilated patients over a period of 2 weeks in June 2018. VT delivered in the first hour was calculated for each patient. Documented body weight and height of each patient was obtained from the nursing chart. PBW was calculated and compared with the documented weight. The difference in VT attributable to the difference in weight has been subsequently calculated.

**Results:** 29 ventilated patients were included (17 males). The mean tidal volume delivered according to the documented body weight was 7.16 ml/kg versus 8.28 ml/kg based on PBW. VT more than 8 ml/kg was delivered in 31% of patients based on documented weight versus 48% when correcting the weight according to the PBW equation.

**Conclusions:** Inaccuracy in documenting weight on patients´ admission to the ICU is a potential cause of delivering unsafe tidal volume [2]. The harm can extend to drug dosage, nutrition provision and renal replacement therapy.

**References**

1. Fan E et al. Am J Respir Crit Care Med. 2017; 195:1253-1263.

2. Bloomfield R et al. Crit Care Med. 2006;34:2153-2157.

### P354 Lung ultrasonography in the diagnosis of ventilator-associated pneumonia

#### V Stoilov, G Pavlov, A Prodanov, E Mitkovski, D Vasilev, D Kazakov, C Stefanov

##### Medical University Plovdiv, Department of Anesthesiology, Emergency and Intensive Care Medicine, Plovdiv, Bulgaria

**Introduction:** Ventilator-associated pneumonia (VAP) is the leading cause of death among mechanically ventilated critically ill patients [1]. Chest radiography (CXR) is essential in the diagnosis of VAP. In the past decade lung ultrasonography has proven to be a valuable tool in the diagnosis and monitoring of lung diseases. The aim of the study is to assess sensitivity and correlation between CXR, lung ultrasound and Clinical Pulmonary Infection Score (CPIS).

**Methods:** In this retrospective, non-randomized study seven patients with proved VAP were enrolled. In all patients CPIS and Lung Ultrasound Score (LUS) [2] were assessed. Comparison of patients that had LUS≥18 and CPIS≥6 points was performed. The correlation between LUS and CXR was done using the Pearson model.

**Results:** We found significant difference between positive CXR patients with LUS≥18 and CPIS≥6 (100% vs 57%, p<0.05). There is a very high correlation between CXR and LUS. These results render lung ultrasound as a highly sensitive tool in the diagnosis of VAP.

**Conclusions:** Our study shows that lung ultrasonography could be used as a reliable supplementary method in the diagnosis of VAP. The benefits of lung ultrasound include the ability to perform it at the patient´s bed without need for transportation, no radiation exposure and repeatability. The high correlation between CXR and lung ultrasound makes echography a valuable adjunct in the diagnosis of VAP.

**References**

1. Vincent JL et al (2009) JAMA 302 :2323.

2. Bouhemad B et al (2015). Anesthesiology 122, 437–447.

### P355 Color Doppler imaging to differentiate between pneumonia and atelectasis in intensive care patients: a prospective observational study

#### M Haaksma^1^, E Broeders^1^, J Nooitgedacht^1^, J Smit^1^, AR Girbes^1^, L Pisani^2^, L Heunks^1^, P Elbers^1^, PR Tuinman^1^

##### ^1^Amsterdam UMC, Vrije Universiteit^,^ Intensive Care, Amsterdam, Netherlands; ^2^Amsterdam UMC, Universiteit van Amsterdam, Amsterdam, Netherlands

**Introduction:** It is difficult to differentiate between pneumonia and atelectasis as cause of lung consolidation in Intensive Care Unit patients. Tools like the Clinical Pulmonary Infection score are of little help (Sensitivity 60% and Specificity 59% for detecting pneumonia) [1]. The objective of this study was to determine the accuracy of ultrasound assessed vascular flow within the consolidation to distinguish these causes.

**Methods:** Adult patients with pulmonary symptoms and lung consolidation on lung ultrasound that were scheduled for Chest-CT were included. Vascular flow was analyzed with color Doppler imaging (flow velocity scale was chosen at 0.25m/sec.). The final diagnosis made by the treating physician was regarded as the gold standard.

**Results:** 20 patients were included of which nine (45%) were diagnosed with pneumonia. Vascular flow in the consolidation was present in seven (78%) out of nine patients with pneumonia, compared to three out of 11 (27%) patients with atelectasis (p = 0.07). The diagnostic accuracy in differentiating between pneumonia and atelectasis was 75%. The sensitivity and specificity were 78% and 73% respectively. The positive predictive value was 70% while the negative predictive value was 80%.

**Conclusions:** Vascular flow in lung consolidations assessed by lung ultrasound in ICU patients aids in differentiating between pneumonia and atelectasis. It outperforms the frequently used Clinical Pulmonary Infection Score.

**Reference**

1. Fartoukh M et al. Am J Respir Crit Care Med. 2003, 168:173-9.

### P356 Transesophageal echocardiography in identifying posterior basal lung atelectasis and monitoring recruitment manouvres

#### O Adi, A Azma Haryaty, T Wan Chuan, R Ismail

##### Hospital Raja Permaisuri Bainun, Emergency & Trauma, Ipoh Perak, Malaysia

**Introduction:** Posterior Basal Lung (PBL) atelectasis is difficult to identify from conventional lung ultrasound in intubated patient with a supine position. These case series evaluate the role of transesophageal echocardiography (TEE) in identifying PBL atelectasis in intubated patients and providing direct visualization of the success of recruitment manoeuvres [1, 2].

**Methods:** Three intubated patients for various causes of respiratory distress undergoing mechanical ventilation were subjected to TEE. At the level of mid-esophagus, the descending aorta short-axis view (0°) the imaging plane is directed through the transverse axis of the descending aorta. Sector depth was increased to image the left pleural space beneath the aorta. For the right lung, the TEE is rotated to the right at the level of atria until lung is seen or until the image of the liver is seen and the probe was withdrawn until the right lung is seen. Recruitment manoeuvres were performed after identifying PBL atelectasis. Atelectatic lungs were visually observed to open up during and after the recruitment manoeuvres.

**Results:** The time to acquire the image of PBL atelectasis from the time of insertion by TEE is short. The images of posterior lung and the effect of lung recruitments is successfully viewed (Fig 1). No immediate complication seen.

**Conclusions:** TEE provides an excellent view of PBL atelectasis and able to directly monitor the success and failures of recruitment manoeuvres.

**References**

1. Mitchell MM. Anesthesiology 1994;81:1546-7.

2. Bouhemad B et al. Am J Respir Crit Care Med. 2011;183:341-7.

**Fig. 1 (abstract P356).**
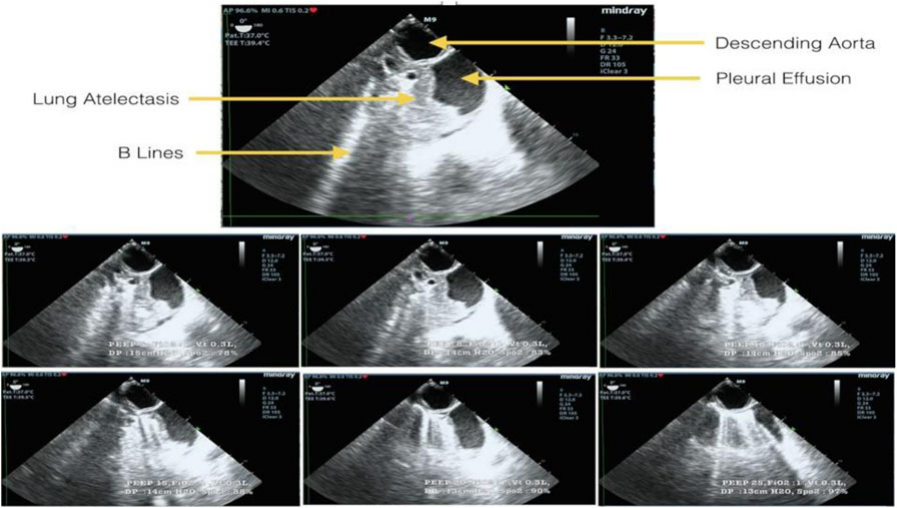
Transesophageal echocardiography (TEE) showing a posterior basal lung (PBL) atelectasis and disappearance of lung consolidation (hepartization or tissue-like appearance) .A formation of B-lines during lung recruitment maneuver (using incremental PEEP method), indicating a part of recruited lung

### P357 Airway pressures and mortality in mechanically ventilated patients

#### W Zarrougui, MA Boujelbèn, N Fraj, I El Meknessi, S Rouis, M Zghidi, A Khedher, I Ben Saida, A Azouzi, K Meddeb, M Boussarsar

##### Farhat Hached University Hospital, Medical Intensive Care Unit, Sousse, Tunisia

**Introduction:** The aim was to identify the discriminative properties of airway pressures (Paw) to predict mortality in mechanically ventilated (MV) patients.

**Methods:** A chart reviews of consecutive MV patients admitted to our MICU from November 2015 to February 2018 was performed. Patients´ characteristics at admission, Paw (at admission and at day 4), high pressure ratio (HPR= number of days spent with high pressures: peak ≥40 cmH2O and/or plateau ≥30 cmH2O; and/or driving pressure ≥14 cmH2O; and/or auto-PEEP ≥6 cmH2O; divided by LOS) and outcomes were recorded. Univariate and multivariate regression analyses were performed to identify factors independently associated to mortality.

**Results:** Were included 304 MV patients. Their main characteristics were: mean age, 56±18years; mean SAPSII, 35±14; pH, 7.3±0.1; pCO2, 50±23mmHg ; PaO2/FiO2, 204±101mmHg; AE/COPD, 105(34.5%); ARDS, 25(8.2%); median MV duration, 6[3;14] days; LOS, 13[6; 21]days; Tracheostomy, 44(14.5%) and mortality, 173(56.9%).

Mean Paw were respectively for peak, plateau, driving and auto-PEEP at admission: 32.3±9.2, 20.4±6, 13.4±5, 4.4±4.2 cmH2O and at day 4: 32.6±10, 20.9±6.6, 13.8±5.3, 6.5±4.4 cmH2O. Median HPR was 0.15[0-0.6]. Univariate analysis showed respectively for the deaths and survivals: peak at day 4 (36.13±10.1 vs 29.13±8.17 cmH2O, p=0.000), plateau at day 4 (23.69±6.93 vs 17.76±4.62 cmH2O, p=0.000), driving at day 4 (15.32±6.38 vs 12.03±3.95 cmH2O, p=0.001) and HPR (0.37[0-1] vs 0.00[0-0.27], p=0.000). Multivariate logistic regression analysis identified o 2 variables as independently associated with ICU mortality: plateau at day 4 (OR, 1.15; 95%CI, [1.01- 1.33]; p=0.43) and HPR (OR, 11.72; 95%CI, [2.65- 51.71]; p=0.01). The ROC-AUCs were respectively for plateau at day 4 and HPR: 0.76 and 0.68.

**Conclusions:** In this non selected consecutive MV patients, an elevated plateau pressure and a length exposure to elevated Paw accounted at least in part for mortality genesis.

### P358 Transpulmonary and respiratory driving pressure changes during proportional assist ventilation

#### E Akoumianaki, V Stamatopoulou, E Pediaditis, K Vaporidi, S Soundoulounaki, E Kondili, D Georgopoulos

##### University Hospital of Heraklion, Intensive Care Unit, Heraklion, Greece

**Introduction:** High respiratory driving pressure (Δ PRS) is strongly associated with increased risk of lung injury and increased mortality during mechanical ventilation. Δ PRS consists of the pressure required to distend the lung the transpulmonary driving pressure (Δ PL) and the pressure required to distend the chest wall. Δ PL is the pressure that increases the risk of lung injury. Data on Δ PL is limited because its measurement requires an esophageal catheter. We aimed to assess changes in Δ PRS and Δ PL during proportional assist ventilation (PAV+) at different experimental conditions.

**Methods:** We retrospectively analyzed patients ventilated with PAV+ who had esophageal pressure measurements before and after dead space or chest load addition. We calculated end-inspiratory plateau pressure (Pplateau), Δ PRS, respiratory system compliance (CRS) and Δ PL during occluded breaths in PAV+ (Figure 1). Data were compared with Wilcoxon signed rank test and p value<0.05 was considered significant.

**Results:** 16 patients were analyzed. Dead space increase (9 patients) did not affect the studied parameters. Chest load (7 patients) significantly increased Pplateau (p=0.03) and Δ PRS (p=0.02) and decreased CRS (p=0.02) but Δ PL remained the same (p=0.4). Median (IQR) changes were 9.5 ml/cmH2O (7.7-13.2) for CRS, 2.9 cmH2O (2.4-5.1) for Pplateau, 3.5 cmH2O (3.2-7.2) for Δ PRS and 0.6 cmH2O (-1.0-1.2) for Δ PL. The reason for Pplateau and Δ PRS increase was the lower chest wall compliance as indicated by the lack of change in Δ PL.

**Conclusions:** In patients ventilated with PAV+, despite significant increases in Δ PRS and Pplateau following chest wall compliance deterioration, Δ PL remained stable. Measurement of Δ PL may help clinicians to identify whether an increase in Δ PRS and Pplateau is associated with increased risk of lung injury during both controlled and assisted mechanical ventilation with PAV+.

**Fig. 1 (abstract P358).**
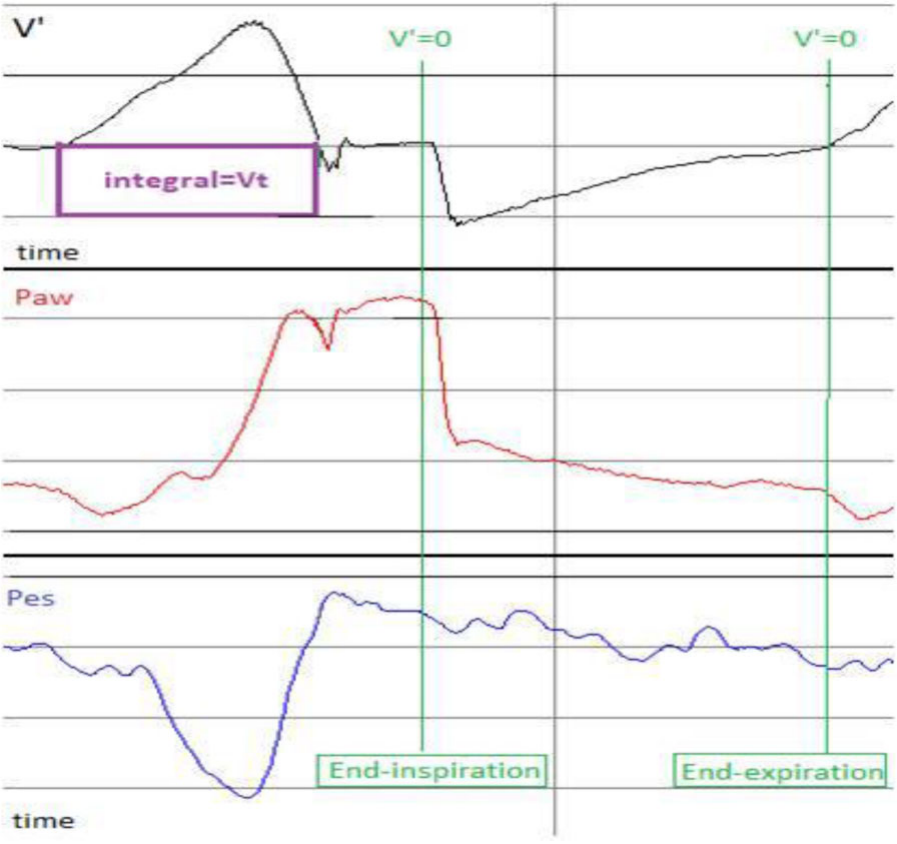
Flow (V´), airway pressure (Paw) and esophageal pressure (Pes) recordings during an occluded breath (V´ is 0) in a patient ventilated with proportional assist ventilation. At endinspiration and endexpiration, respiratory driving pressure (ΔPRS) and transpulmonary driving pressure (ΔPL) were calculated as follows: ΔPRS = endinspiratory Paw - endexpiratory Paw. ΔPL = (endinspiratory Paw-endinspiratory Pes) - (endexpiratory Paw - endexpiratory Pes)

### P359 Different particle flow in exhaled air in mechanically ventilated patients with cardiac failure following cardiac surgery

#### S Hyllen^1^, S Lindstedt^2^

##### ^1^Skane University Hospital and Lund University, Cardiothoracic Aneasthesia and Intensive Care, Lund, Sweden; ^2^Skane University Hospital and Lund University, Cardiothoracic Surgery and Transplantation, Lund, Sweden

**Introduction:** Particle flow in exhaled air from mechanically ventilated patient’s mirrors the opening and closing of small airways and can be detect by optical particle counter [1]. We hypothesized that this particle flow is affected by cardiac function.

**Methods:** Exhaled air from 20 mechanically ventilated patients was analyzed using a customized optical particle counter PExA, Figure 1. Control group had a normal cardiac function (n=10) and received invasive ventilation as a part of the routine postoperative care following cardiac surgery. The other group (n=10) had preoperative left ventricle ejection fraction less than 30% or other evidence of cardiac failure. Particles in range 0.41–4.55 μ m were measured.

**Results:** Median particle flow rate (PFR) in patients with cardiac failure was higher than PFR in those with normal cardiac function, 4855 particles/hour, interquartile range 1955-11676 particles/hour respectively 919 particles/hour, interquartile range 582-931 particles/hour, p<0.001. Particles and their size distribution are presented in Figure 2.

**Conclusions:** PFR from the airways in patients with cardiac failure was significantly higher compare to PFR in patients with normal cardiac function. Our findings indicate that measurement of PFR might have a potential as a diagnostic tool for different respiratory and cardiac conditions during mechanical ventilation.

**Reference**

1. Broberg E et al. Intensive Care Med Exp 6:18, 2018

**Fig. 1 (abstract P359).**
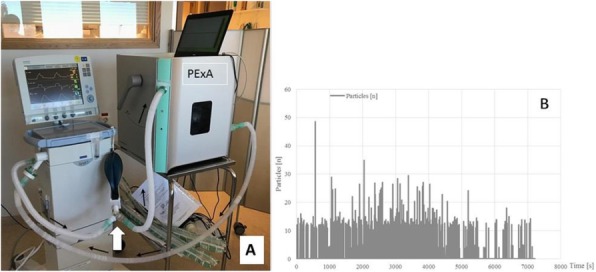
A. The respiratory circuit with optical particle counter PExA. The white arrow shows non-rebreathing valves. The black arrows show the direction of the air. Notice how the PExA is connected on the outflow from the black balloon, which represents a patient, and then back into the respirator again. B. Real-time on-line particle flow measurement from one of the studied patients in the cardiac failure cohort

**Fig. 2 (abstract P359).**
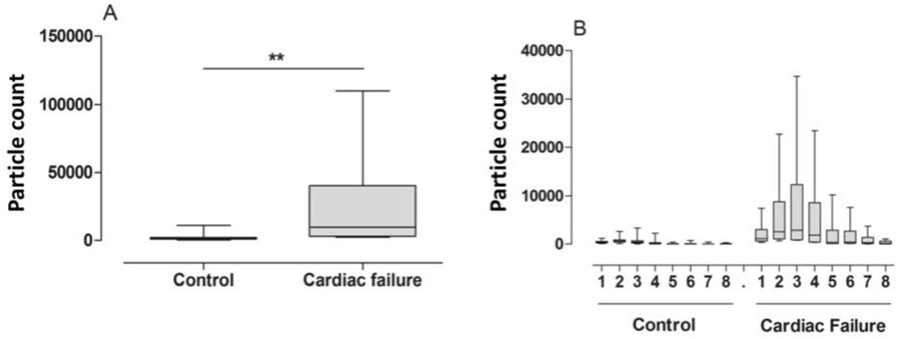
The distribution of the total particle count (A) and according to the mean diameter of particles (B) during two-hour measurement. The line inside the box represents the median value. The means of the particle’s diameter in groups 1-8 are: 0.48 μm, 0.59 μm, 0.75 μm, 0.98 μm, 1.22 μm, 1.67 μm, 2.52 μm, and 3.37 μm. ** indicates p < 0.01. Error bars represent confidence intervals

### P360 Accuracy of stress index, driving pressure and mechanical power to identify injurious ventilation

#### F Alessandri^1^, E Piervincenzi^2^, D Alunni Fegatelli^3^, V Fanelli^4^, G Tellan^2^, F Pugliese^5^, P Terragni^6^, L Mascia^7^, M Ranieri^8^

##### ^1^Policlinico Umberto I, Rome, Department of Anesthesia and Critical Care Medicine, Roma, Italy; ^2^Sapienza University of Rome, Department of Anesthesia and Critical Care Medicine, Rome, Italy; ^3^Sapienza University of Rome, Department of Infectious Disease, Medical Statistics and Public Health, Rome, Italy; ^4^University of Turin, Department of Anesthesia and Intensive Care Medicine, Turin, Italy; ^5^Sapienza University of Rome, department of Anesthesia and Critical Care Medicine, Rome, Italy; ^6^University of Sassari, Department of Surgical Sciences, Sassari, Italy; ^7^Sapienza University of Rome, Department of Biotechnological and Medical and Surgical Sciences, Rome, Italy; ^8^Alma Mater Studiorum University of Bologna, department of Anesthesia and Critical Care Medicine, Bologna, Italy

**Introduction:** We assessed the diagnostic accuracy of Mechanical Power (MP) and Driving Pressure (DP) alone and combined with Stress Index (SI) to identify ventilator settings likely to produce ventilator induced lung injury caused by tidal hyperinflation [1-3].

**Methods:** Secondary analysis of a previous database of ARDS patients [1]. Computerized tomography markers of tidal hyperinflation (were used as a “reference standard”. Analysis of the area under the receiver-operating characteristics curve (AUC) was used using a two-fold cross-validation.

**Results:** In a cluster of 44 patients, a “training set” of 28 not hyperinflated patients was compared with a “validation set” of 14 hyperinflated patients. The AUC for SI was higher than for MP and DP [0.776 (95% CI 0.610 - 0.941) vs. 0.679 (95%, CI 0.509 - 0.848) vs. DP 0.388, (95% CI 0.219 - 0.556), respectively]. Combination of SI with MP and DP was not significantly improved [SI+MP: 0.689 (95% CI, 0.520 - 0.858) vs. SI+DP: 0.709 (95% CI, 0.530 - 0.888), respectively] (Figure 1-2).

**Conclusions:** SI seems to be more accurate than MP and DP in identifying tidal hyperinflation in patients with ARDS. Specificity and sensibility were not improved combining SI with MP or DP.

**References**

1) Terragni PP et al. Anesthesiology 2013; 119: 880-9.

2) Gattinoni L, et al. Intensive Care Med. 2016; 42: 1567-1575.

3) Amato MB, et al. N Engl J Med. 2015; 372: 747-55.

**Fig. 1 (abstract P360).**
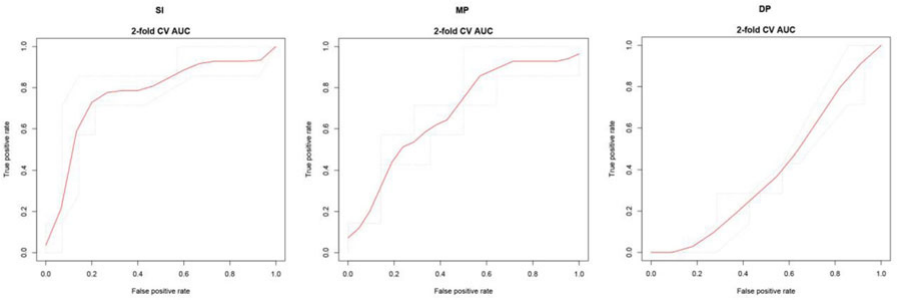
ROC SI, MP and DP

**Fig. 2 (abstract P360).**
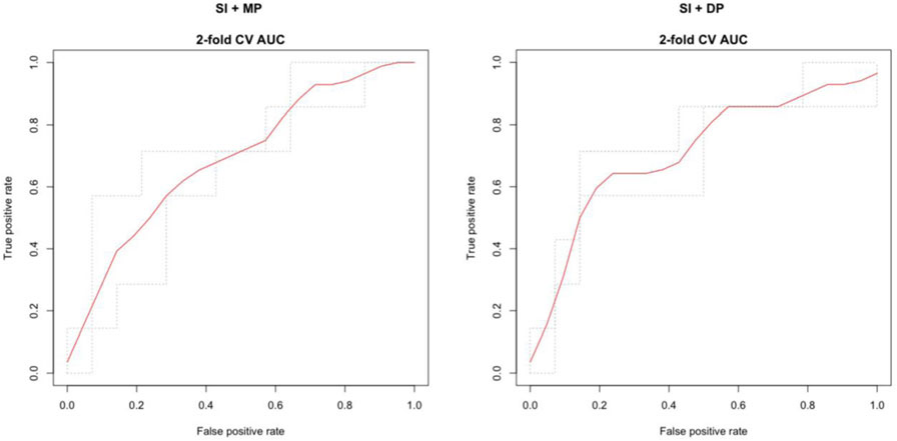
ROC SI+MP and SI+DP

### P361 The effect of FiO_2_ on the PaO_2_ /FiO_2_ ratio in mechancally ventilated intensive care patients

#### S Coenen, H De Vries, F Kingma, A Jonkman, B Smit, P Tuinman, L Heunks, A Spoelstra-de Man

##### Amsterdam University Medical Centers, VU Medical Center, Department of Intensive Care, Amsterdam, Netherlands

**Introduction:** The PaO_2_/FiO_2_ (P/F) ratio is widely used to assess the severity of lung injury. Conceptually, the P/F ratio should be independent of the FiO_2_ and solely depend on the pulmonary condition. However, effect of FiO_2_ modulation on the P/F ratio has not been well characterized in ventilated intensive care (ICU) patients. The purpose of the present study was to investigate the relationship between FiO_2_ and the P/F ratio in ICU patients on mechanical ventilation.

**Methods:** In a prospective, interventional study 10 patients with a Swan Ganz catheter in situ were included. The P/F ratio was calculated at FiO_2_ levels ranging from 0.21 to 1.0 with 10 minute intervals. During the study other ventilator settings were not modulated. To understand the physiological effects of FiO_2_ modulation on gas exchange and hemodynamics, mixed venous oxygen saturation and cardiac output were assessed. Shunt fraction was calculated as described by West [1].

**Results:** Patient characteristics and ventilator settings are reported in Table 1. All patients were admitted to the ICU after elective cardiac surgery. Modulation of FiO_2_ did have a significant effect on the P/F ratio, following a U-shaped pattern (P < 0.05) (Figure 1). The shunt fraction varied with altering FiO_2_ levels, also exhibiting a U-shaped pattern (P < 0.05) (Figure 2). Cardiac output was not affected by FiO_2_.

**Conclusions:** In contrast to current thinking, the P/F ratio varied substantially with altering FiO_2_ levels in mechanically ventilated ICU patients. This is an important novel physiological observation. In addition, it demonstrates that the assessment of the severity of respiratory failure by using the P/F ratio should be standardized to a fixed FiO_2_ level.

**Reference**

1. West J. Respiratory Physiology: The Essentials, 2005, 7th ed, Page 169

**Table 1 (abstract P361).** Patient characteristics and ventilator settings

Age, years	57 [50-66]
Male	9
Ventilator Settings	
Pressure Support	2
Pressure Control	8
PEEP, cmH2O	5 [5-6]
PaO2, mmHg	111 [89.63-127.88]
PaO2/FiO2 ratio, mmHg	234.9 [209.1-319.7]

PEEP: positive end expiratory pressure. PaO_2:_ arterial partial pressure of O_2_ . FiO_2_: fraction of inspired O_2_ . Hb: hemoglobin. Data are presented as median [Interquartile range] or number

**Fig. 1 (abstract P361).**
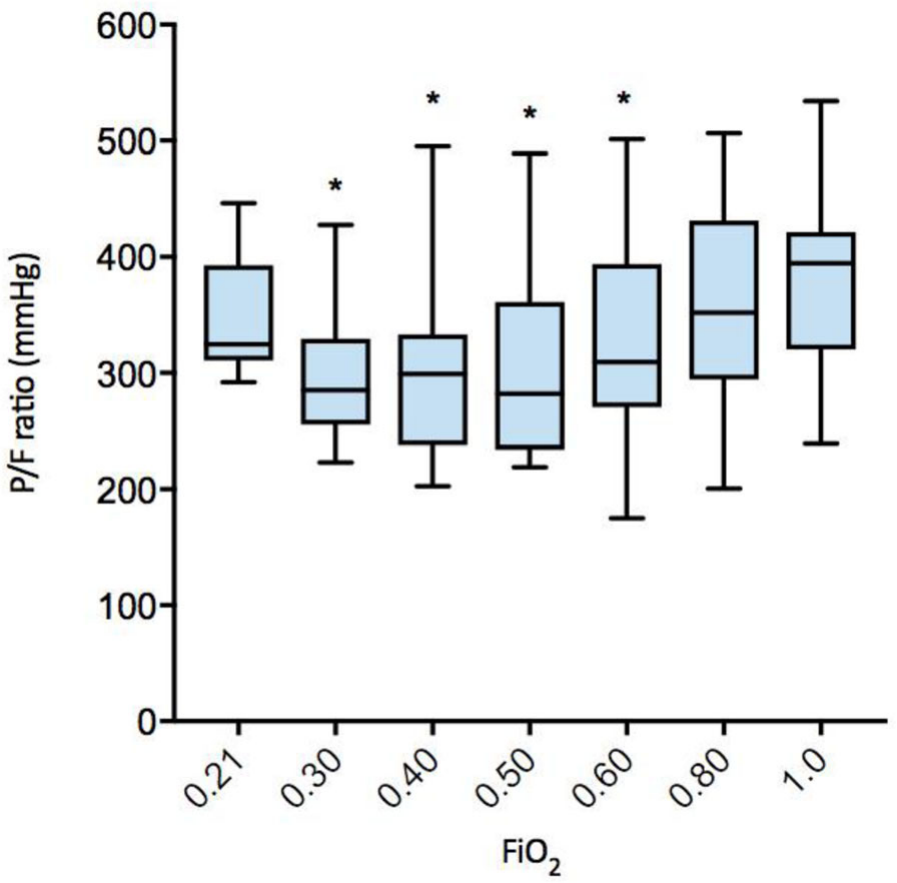
PaO_2_/FiO_2_ ratio as a function of FiO_2_ for all patients. At FiO_2_ 0.21 measurements were performed in 7 patients to ensure adequate saturation. At FiO_2_ ≥ 0.3 all patients were measured. Median, interquartile range and minimum/maximum are shown for every FiO_2_. Asterisks denote significant differences (P < 0.05) between the denoted FiO_2_ level and FiO_2_ of 0.8 and 1.0

**Fig. 2 (abstract P361).**
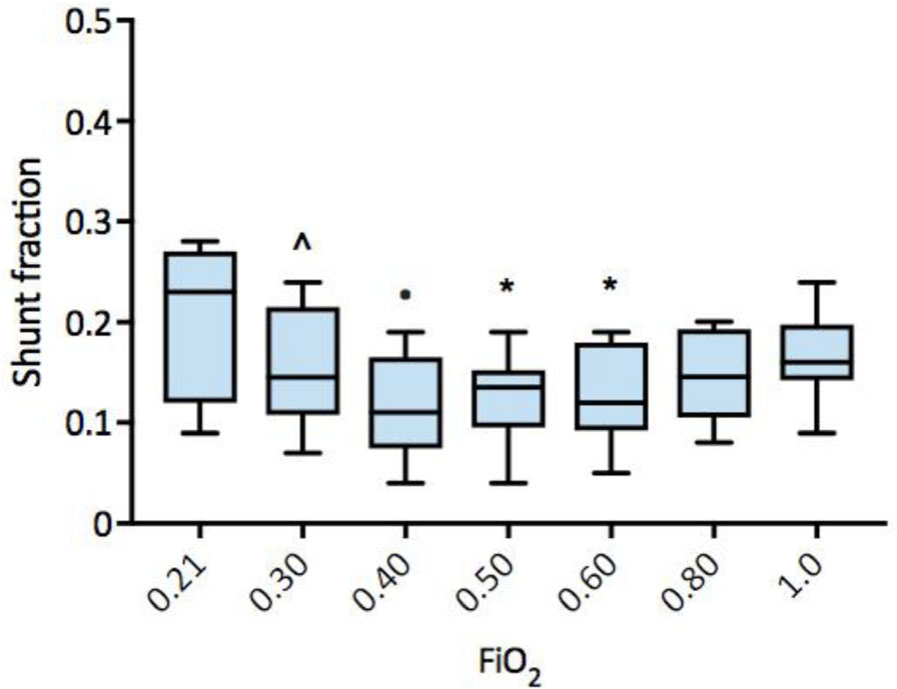
Shunt as a function of FiO_2_ for all patients. At FiO_2_ 0.21 measurements were performed in 7 patients to ensure adequate saturation. At FiO_2_ ≥ 0.3 all patients were measured. Median, interquartile range and minimum/maximum are shown for every FiO_2_. Significant differences (P < 0.05) between FiO_2_ levels are denoted as following: caret (^) denotes the significant difference between FiO_2_ 0.3 compared to 0.4 and 0.6, bullet (•) denotes the significant difference between FiO_2_ 0.4 to 0.21, 0.3 and 1.0 and finally, asterisks (*) denote the significant differences between FiO_2_ 0.5 and 0.6 compared to 1.0

### P362 Diaphragmatic dysfunction in brain injured patients: a pilot study

#### G Cammarota^1^, E De Sanso^2^, I Sguazzotti^1^, M Costa^2^, F Grossi^1^, A Bellantoni^2^, F Carfagna^2^, P Navalesi^3^, F Della Corte^2^, R Vaschetto^2^

##### ^1^AOU Maggiore della Carità Hospital, Anesthesiology and Intensive Care, Novara, Italy; ^2^Università del Piemonte Orientale, Dipartimento di Medicina Traslazionale, Novara, Italy; ^3^Università “Magna Graecia”, Dipartimento di Scienze Mediche e Chirurgiche, Catanzaro, Italy

**Introduction:** In brain injured patients extubation failure occurs frequently, therefore, with this study we aim to investigate whether diaphragmatic dysfunction measured by ultrasound represents one of the major determinants of extubation failure in intubated patients or failure of disconnection from the ventilator in tracheostomized patients.

**Methods:** Patients entering ICU with the following major inclusion criteria were considered eligible: 1) diagnosis of intraparenchymal hemorrhage, subarachnoid hemorrhage, or traumatic brain injury, 2) absence of pneumothorax. In all patients, excursion and thickening fraction (TFdi) were performed, by ultrasound, at the beginning of ventilation (T0), after 48 hours (T48), during the spontaneous breathing trial (SBT) and after extubation or disconnection from the ventilator.

**Results:** The 74 patients included, presented, on average, an age of 62(15) years, a SAPSII of 48(14) and a mechanical ventilation duration of 14(8) days. On T0 and T48, TFdi and excursion were similar i.e., 26%(15) vs. 25%(16) and 1.08(0.46) cm vs. 1.18(0.53) cm, respectively and reduced compared to normal physiologic values. Of the 38 patients attempting SBT, 24 patients succeeded and 14 failed. Patients who failed presented lower but non-significant TFdi compared to those who succeeded both during SBT i.e., 24%(14) vs. 30%(24) and after extubation or disconnection from the ventilator i.e., 22%(13) vs. 33%(29). Among patients who were weaned off, excursion was 1.23(0.57) cm and 1.55(0.92) cm during the SBT and after extubation or disconnection, while in those who failed was 2.04(2.27) cm and 1.24(0.47) cm. Among the predictors of successful SBT i.e., rapid shallow breathing index (RSBi), diaphragmatic RSBi, TFdi or excursion at SBT, the RSBi performed better than the others with an AUC of 0.75.

**Conclusions:** Despite diaphragmatic dysfunction seems to be very common in brain injured patients, it is not a major determinant of extubation or disconnection failure.

### P363 Diaphragm strength and electrodes efficiency after implantation of a pacing stimulation system in high spinal cord injury patients

#### S Gianni^1^, F Curto^2^, M Giacomini^2^, R Pinciroli^1^, M Favarato^1^, G Stagni^2^, D Facchetti^2^, R Fumagalli^1^, A Chieregato^2^

##### ^1^University of Milan-Bicocca Medical School, Monza, Italy; ^2^ ASST Grande Ospedale Metropolitano Niguarda, P.zzaOspedale Maggiore 3, Neuro ICU, 20162 Milan, Italy

**Introduction:** Implantation of a Diaphragmatic Pacing Stimulation (DPS) system is a useful aid to mechanical ventilation in properly selected spinal cord injury (SCI) patients [1]. The aim of this study is to evaluate diaphragm strength, endurance and 4-electrodes stimulation efficiency in ventilator-dependent SCI patients after DPS implantation.

**Methods:** Six consecutive ventilator-dependent SCI patients underwent laparoscopic DPS implant. Phrenic compound Motor Action Potential (cMAP) and trans-diaphragmatic pressure (Pdi-twitch) were measured before (T0) and 6-months after DPS implant (T1). Were also registered the Tidal Volume (TV) and Pressure-time Product (PTP) of the trans-diaphragmatic pressure (PTPdi), stimulating first all 4 electrodes simultaneously, then each single electrode one-by-one.

**Results:** Phrenic nerve cMAP latency did not significatively differ between T0 and T1. An increased cMAP amplitude at T1 was found for left phrenic nerve stimulation (p. 0.046). No difference could be detected between Pdi-twitch values at T0 vs. T1 for both hemidiaphragms. At 6-months, from 24-hours ventilator dependency at T0, patients were spending an average 7.67 ± 6.15 hours a day free from positive-pressure ventilation. (Tab. 1) The evaluation of DPS efficiency showed important differences and heterogeneity in terms of the relative contribution of each electrode to the TV and Pdi being subsequently developed by patients.

**Conclusions:** DPS implantation led to an increased diaphragmatic endurance, with high SCI patients transitioning to complete ventilator-dependency to several daily hours of DPS-triggered breaths. Conversely, diaphragm strength (Pdi-twitch) did not increase at T1. The contribution of each electrode Pdi and TV is variable between subjects and should be evaluated in order to optimize DPS system.

**Reference**

1. Glenn WW et al. Pacing Clin Electrophysiol 1986;9:780–4.

**Table 1 (abstract P363).** Study population

ID	Age	Lesion level	Etiology	Hours of DPS during the day
1	8	C2	Car crash	4
2	18	C0	Arnold-Chiari syndrome	16
3	57	C1-C2	Car crash	2
4	30	C2	Diving trauma	4
5	25	C2-C3	Car crash	5
6	36	C2	Car crash	15

### P364 Utility of successful weaning predictors in patients submitted to prolonged mechanical ventilation

#### JF Martínez Carmona, JE Barrueco Fanccioni, M Delgado Amaya

##### Regional University Hospital of Málaga, Intensive Care Department, Málaga, Spain

**Introduction:** To assess the possibility of successful weaning in patients undergoing mechanical ventilation, we have a set of tools to be used during a Spontaneous Breathing Test (SBT), being useful when making the decision to extubate the patient. It has been shown to decrease the risk of weaning failure as well as associated morbidity and mortality. However, not all the tools will be useful in all patients, it will depend on multiple factors, which highlights the pathology that required mechanical ventilation (MV), the severity of the patient and the duration of MV.

**Methods:** Prospective study that included 15 patients admitted to the ICU due to various pathologies. All patients receive prolonged MV > 7 days. Once the cause of MV was solved, a SBT was performed in Support Pressure 10 cmsH2O with PEEP 5 cmsH2O. Measurements: MIP, P0.1, Rapid Shallow Breathing Index (RSBI), Diaphragmatic Excursion (DE) and Thickness Diaphragm Index (TDI) and the Richmond Agitation Sedation Scale (RASS), secretion management and effective cough are valued.

**Results:** The mean age was 55.6 ± 14. 66.7% males. Reasons admission: TBI (20%), AHS (20%). 73.33% weaning failure. ICU mortality 23.7%. Large part of the patients who presented weaning failure presented good values in the usual predictors during SBT. In our sample, only P0.1 correlated adequately. The diaphragmatic assessment showed a better correlation with weaning failure (DE <19.5 mm / TDI <41% presented a higher risk of weaning failure). The factors that best correlated with weaning failure were: RASS <-1 /> +2, poor secretion management and ineffective cough.

**Conclusions:** In patients undergoing prolonged mechanical ventilation, we must take into account all the factors that may affect our patients. The assessment of diaphragmatic dysfunction is key to preventing weaning failure. An optimal level of consciousness as well as a good management of secretions are key to a successful weaning.

### P365 Prognostic value of the minute ventilation to CO_2_ production ratio as a marker of ventilatory inefficiency in the ICU

#### R Lopez^1^, R Pérez^1^, Á Salazar^1^, I Caviedes^2^, J Graf^1^

##### ^1^Clínica Alemana de Santiago, Departamento de Paciente Crítico, Santiago, Chile; ^2^Clínica Alemana de Santiago, Unidad de Enfermedades Respiratorias, Santiago, Chile

**Introduction:** Ventilatory inefficiency for CO2 clearance may provide better severity stratification in acute respiratory failure than oxygenation [1]. Ventilatory inefficiency (VI) is best assessed by the Bohr-Enghoff physiological dead space [2]. We recently reported that the minute ventilation to CO2 production ratio (VE/VCO2), a simplified VI index from exercise testing that obviates the PaCO2 measurement, correlates better than other VI indices to physiological dead space in mechanically ventilated patients [3]. Here we report the prognostic performance of this index using a survival analysis.

**Methods:** The steady state VE/VCO2 ratio was prospectively recorded within 24 hours of intubation in patients ventilated for more than 48 hours. A 28-day survival record was performed. For a dichotomic analysis of the VE/VCO2 ratio a cutoff value was determined from a univariate mortality analysis. The odds ratio (OR) for death was determined and survival analysis at 28 days was performed. Our local institutional review board approved anonymized ICU patient data collection.

**Results:** A cohort of 79 patients with a global 28-day mortality of 10.1% was enrolled. Median [IQR] VE/VCO2 was 42.7 [37.2-47.9]. Mean±SEM VE/VCO2 was higher in patients who died than those who survived (58±8 vs 43±1, p<0.001, Figure 1). We found a VE/VCO2 cutoff value of 45. Mortality was higher in patients with High-VE/VCO2 (≥45) as compared to those with Low-VE/VCO2 (21% vs 4%, p=0.021) with an Odds ratio of 6.7 [95%-CI 1.2-35.8]. Cumulative mortality was higher in the High-VE/VCO2 than in the Low-VE/VCO2 group (Log-rank p=0.015, Figure 2).

**Conclusions:** In this unselected cohort of mechanically ventilated patients an early high VE/VCO2 ratio was associated to 28-days mortality. The VE/VCO2 ratio may be a simple and non-invasive VI index with prognostic value in this population.

**References**

1. Nuckton TJ. N Engl J Med 2002;346:1281 1286.

2. Sinha P. Intensive Care Med 2011; 37:735-746

3. López R. Intensive Care Med 2017;43:1542-1543.

**Fig. 1 (abstract P365).**
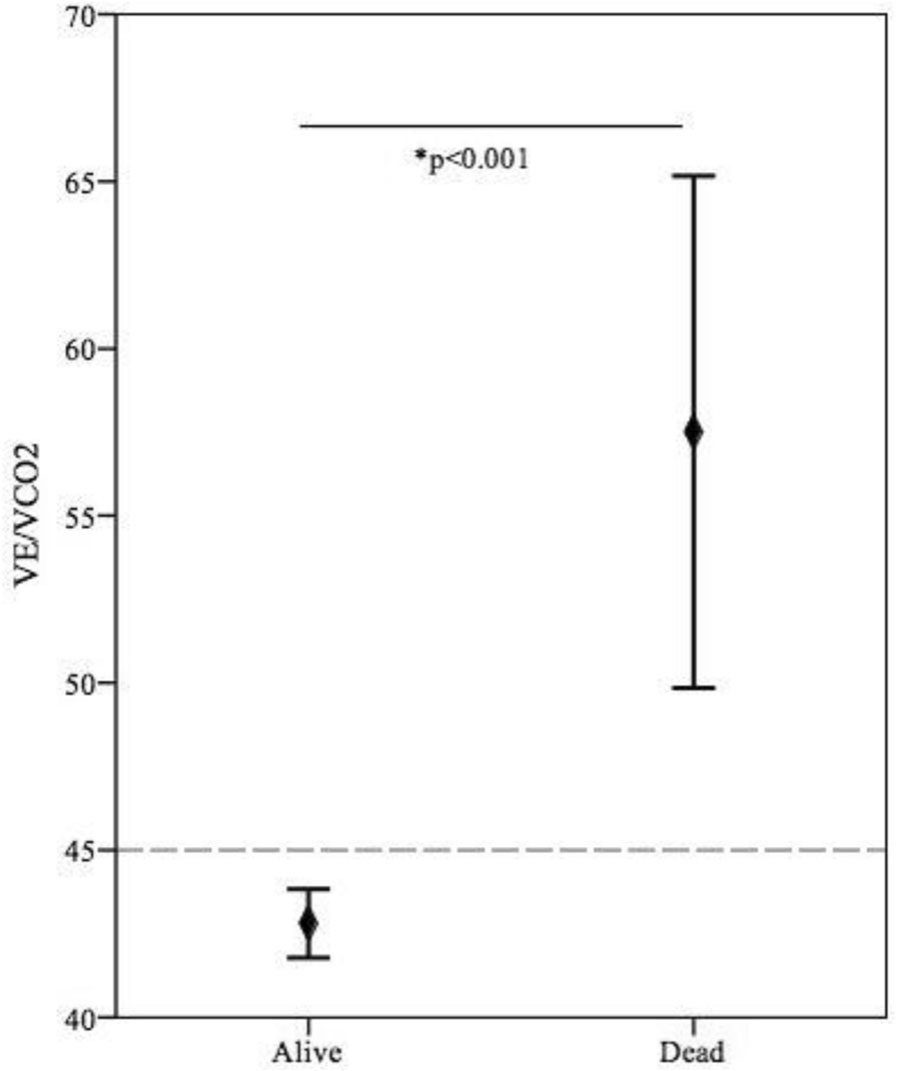
VE/VCO2 ratio obtained within 24 hours of intubation according to 28-days survival stratification (mean±SEM). Those who died had a significantly higher VE/VCO2 ratio. The VE/VCO2 cutoff value from univariate mortality analysis is shown as a gray dashed line

**Fig. 2 (abstract P365).**
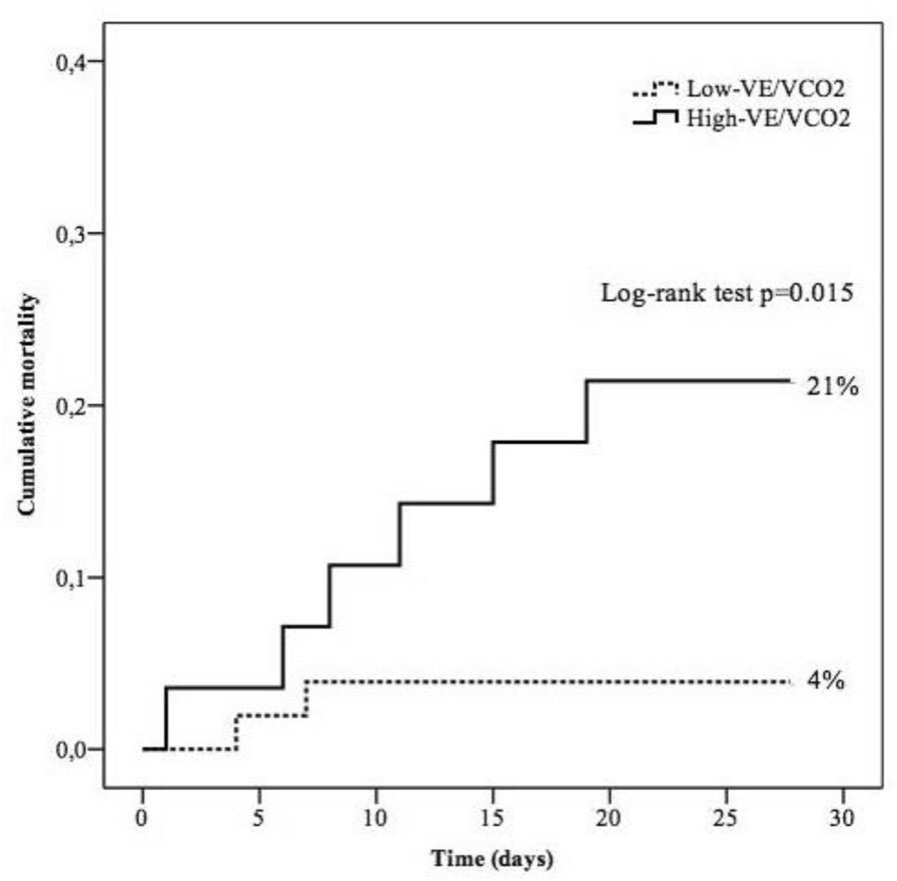
Kaplan-Meier cumulative mortality chart according to dichotomic VE/VCO2 ratio cohort stratification. The “Low-VE/VCO2” category includes patients with a VE/VCO2 ratio <45 within 24 hours of intubation and the “High-VE/VCO2” those with a VE/VCO2 ratio ≥45

### P366 Sodium thiosulfate protects swine with pre-existing coronary artery disease against hemorrhagic shock-induced acute lung injury

#### M Wepler^1^, T Merz^1^, A Schmid^1^, F Kohn^1^, U Wachter^1^, J Vogt^1^, A Hoffmann^1^, M Gröger^1^, C Hartmann^1^, T Schulz^1^, B Stahl^1^, A Seifritz^1^, E Calzia^1^, M Georgieff^2^, F Zink^1^, O McCook^1^, B Nussbaum^2^, P Radermacher^3^, T Datzmann^1^

##### ^1^Institute for Anesthesiological Pathophysiology and Process Engineering, Ulm, Germany; ^2^Department of Anesthesiology, University Hospital, Ulm, Germany; ^3^Institute of Pathophysiology and Process Engineering, ICU, Ulm, Germany

**Introduction:** Sodium thiosulfate (STS) is a clinically relevant and safe hydrogen sulfide donor that improved acute lung injury (ALI) and brain ischemia/reperfusion injury in previous studies [1,2].

**Methods:** In a prospective, controlled, randomized, and double-blinded trial, twenty adult, anesthetized, mechanically ventilated and surgically instrumented swine with preexisting coronary artery disease [3] underwent 3 h of hemorrhagic shock (HS; removal of 30% of the calculated blood volume and subsequent titration of mean arterial pressure to 40mmHg). Post-shock resuscitation (72h) comprised re-transfusion of shed blood, crystalloids, and norepinephrine. Animals were randomly assigned to “placebo” or “STS” (0.1g·kg^-1^·h^-1^ for 24h). Before, at the end of and every 24h after shock, hemodynamics, blood gases, and lung function were recorded.

**Results:** Survival rates did not differ between groups. STS-infusion attenuated the HS-induced impairment of lung mechanics and pulmonary gas exchange (Table 1,2), resulting in a significantly higher Horovitz/PEEP-ratio (Figure 1).

**Conclusions:** STS during acute resuscitation from HS may protect co-morbid swine against HS-induced ALI.

**Acknowledgement**

Supported by the DFG (CRC1149, GEROK to M. Wepler) and Köhler Chemie, Germany

**References**

1. Sakaguchi et al, Anesthesiology 2014;121:1248

2. Marutani et al, J Am Heart Assoc 2015;4:e0021253

3. Hartmann et al, Crit Care Med 2017;45:e1270

**Table 1 (abstract P366).** Measurements in sodium thiosulfate (STS) and placebo-treated swine at baseline, at the end of 3h of hemorrhagic shock (HS), and during resuscitation from HS (24, 48, and 72h)

Parameter	Treatment	Baseline	Schock	72h
pH	Placebo	7.46 (7.45; 7.49)	7.30 (7.25; 7.41)*	7.51 (7.48; 7.55)
	Thiosulfate	7.45 (7.42; 7.47)	7.26 (7.24; 7.38)*	7.52 (7.52; 7.55)
Base excess (mmol·L^-1^)	Placebo	3.6 (2.5; 6.0)	-2.6 (-5.4; 1.8)	8.7 (7.8; 10.7)*
	Thiosulfate	4.9 (0.9; 5.4)	-3.5 (-8.2; -0.1)*	9.4 (7.9; 10.0)*
Lactate (mmol·L^-1^)	Placebo	3.1 (2.4; 3.8)	6.0 (4.5; 8.0)	0.8 (0.7; 1.2)*
	Thiosulfate	2.4 (2.3; 3.8)	7.5 (5.8; 8.8)*	0.8 (0.6; 1.1)

*P<0.05 vs. baseline in the same treatment group. Pplat=plateau pressure. Data is presented as median and interquartile range

**Table 2 (abstract P366).** Measurements in sodium thiosulfate (STS) and placebo-treated swine at baseline, at the end of 3h of hemorrhagic shock (HS), and during resuscitation from HS (24, 48, and 72h)

Parameter	Treatment	Baseline	Schock	72h
Horovitz Index (mmHg)	Placebo	400 (338; 448)	376 (322; 431)	289 (106; 323)*
	Thiosulfate	351 (328; 427)	352 (283; 405)	337 (300; 387)
Pplat (mbar)	Placebo	12 (10; 13)	13 (12; 14)	29 (25; 34)*
	Thiosulfate	11 (9; 13)	13 (10; 14)	23 (21; 24)*#
Effective compliance (ml·mbar^-1^)	Placebo	42 (37; 49)	40 (33; 43)	32 (22; 40)*
	Thiosulfate	44 (42; 50)	39 (38; 42)	36 (33; 47)

*P<0.05 vs. baseline in the same treatment group. #P<0.05 vs. placebo at the same time point. Pplat=plateau pressure. Data is presented as median and interquartile range

**Fig. 1 (abstract P366).**
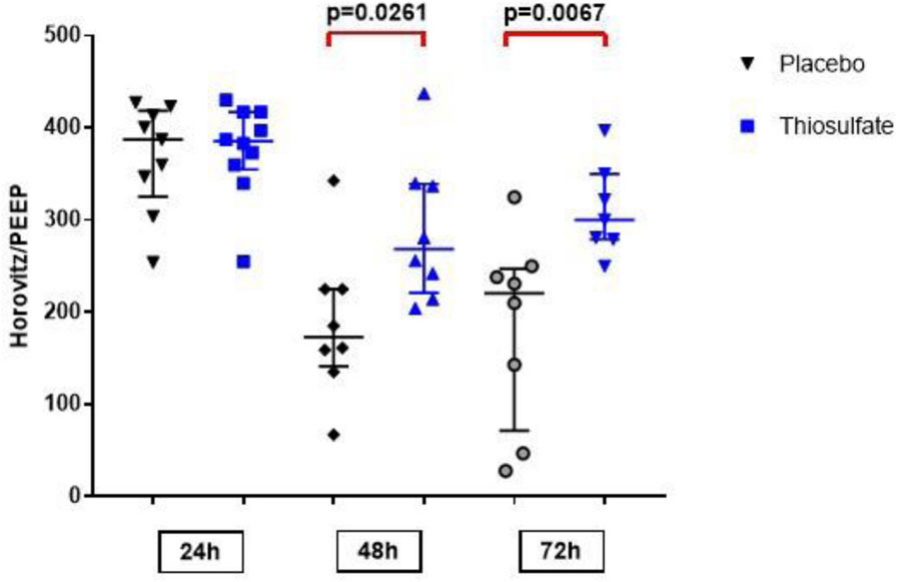
Horovitz/PEEP-ratio in sodium thiosulfate (blue) and placebo-treated (black/gray) swine during resuscitation after 3h of hemorrhagic shock (24, 48, and 72h). Data is presented as median and interquartile range

### P367 Necrosis, rather than apoptosis, is the dominant type of alveolar epithelial cell death in LPS-induced experimental ARDS

#### N Tamada, K Tojo, T Goto

##### Yokohama City University Graduate School of Medicine, Anesthesiology and Critical Care Medicine, Yokohama, Japan

**Introduction:** Alveolar epithelial cell (AEC) death is a main mechanism of severe respiratory failure in acute respiratory distress syndrome (ARDS). Classically, cell death is classified into necrosis or apoptosis. Recent studies have reported that not only apoptosis but also certain types of necrosis are molecularly regulated and that these regulated necrosis can be therapeutic targets for various diseases. However, the relative contribution of necrosis and apoptosis to AEC death in ARDS has not been elucidated. Our study aimed to elucidate which type of cell death is dominant in AEC death and to evaluate whether the regulated necrosis is involved in LPS-induced experimental ARDS.

**Methods:** We established ARDS model by instilling 25μ g of LPS intratracheally to mice. To estimate the relative proportion of apoptosis and necrosis in AEC death, we measured cytokeratin18 M65 level (total cell death marker) and M30 level (apoptosis maker) in bronchoalveolar lavage fluid (BALF) by ELISA, and quantified propidium iodide-positive necrotic cells and TUNEL-positive apoptotic cells in the lung sections. Moreover, we performed pathway enrichment analysis of gene expression data from PCR array to evaluate whether regulated necrosis pathway is associated with the ARDS model.

**Results:** Both M65 and M30 levels were increased in the ARDS mice. The M30/M65 ratio (an indicator of the proportion of apoptosis to total cell death) in the ARDS mice was significantly lower than that of healthy controls. Moreover, the number of propidium iodide-positive necrotic cells was significantly higher than that of TUNEL-positive apoptotic cells in ARDS mice. In the pathway enrichment analysis, the necroptosis pathway, a regulated necrosis pathway, was associated with LPS-induced experimental ARDS.

**Conclusions:** AEC necrosis is more dominant than apoptosis in LPS-induced ARDS model. Moreover, necroptosis may contribute to ARDS pathogenesis. AEC necrosis including necroptosis is a potential therapeutic target for ARDS.

### P368 Clinical ARDS diagnosis is not associated with a unique circulating neutrophil cell surface phenotype

#### T Craven^1^, S Duncan^1^, S Johnston^2^, C Haslett^1^, K Dhaliwal^1^, T Walsh^3^

##### ^1^EPSRC Proteus IRC, Centre for Inflammation Research, Edinburgh, United Kingdom; ^2^University of Edinburgh, Centre for Inflammation Research, University of Edinburgh, Edinburgh, United Kingdom; ^3^Royal Infirmary of Edinburgh, Department of Anaesthesia, Intensive Care, and Pain Medicine, Edinburgh, United Kingdom

**Introduction:** Acute respiratory distress syndrome (ARDS) is a form of non-cardiogenic oedema due to alveolar injury secondary to an inflammatory process. The clinical diagnosis is defined by the Berlin Criteria but this may not reflect the underlying biological process. The activated neutrophil is central to the pathogenesis of ARDS, characterised by altered cell surface markers.

**Methods:** Three cohorts of seven participants were recruited. The first cohort suffered from mild, moderate or severe ARDS as defined by the Berlin Criteria [1]. The second cohort was composed of ventilated patients on the intensive care unit with acute inflammatory lung disease (diagnosis of clinical suspicion) but did not meet the Berlin Criteria for ARDS. A third cohort was composed of age and sex matched healthy volunteers. Procurement of human tissue was approved by a regional Ethics Committee (14/SS/1074 or 08/S1103/38 or AMREC: 15-HV-013) and with the informed consent of the participant or their personal legal representative. Patients were excluded if aged under 16 or over 80 years of age, were expected to survive for less than 24 hours, if the attending physician refused, due to the absence of suitable indwelling vascular catheter, if the haemoglobin concentration was below 6.5 g/dL, or if the patient was enrolled in a trial of novel anti-inflammatory agent. Whole blood (lysed erytocytes) underwent flow cytometry to determine CD11b, 63, 66b, 88, 62L and 16.

**Results:** A description of the enrolled cohorts can be found in Table 1. There were no significant differences between the mechanically ventilated, critically ill cohorts for any cell surface molecule in the multiplicity adjusted p values (Fig 1).

**Conclusions:** The results support the conjecture that clinical diagnostic criteria should not be used as a surrogate to stratify patients according to biological changes, with implications for the testing of biological therapies.

**Reference**

Ranieri VM et al (2012) JAMA 307:2526–33

**Table 1 (abstract P368).** A description of enrolled cohorts

	Control n=6*	ARDS n=7	nonARDS n=7	p
Age Median (±IQR)	60.5 (45.5-68.0)	65 (47-67)	60 (46-74)	0.9240 ^Φ^
Gender M:F (%)	4:2	5:2	4:3	0.8503 ^θ^
APACHE II Median (±IQR)	N/A	21 (15-24)	24 (17-29)	0.2984 ^a^
SOFA Median (±IQR)	N/A	10.5 (6.75-13.25)	11 (9-12)	0.6503 ^a^
Survived to hosp.dis. (%)	N/A	4 (57.1)	4 (57.1)	1.000 ^Β^
PF ratio Median (±IQR)	N/A	17.44 (11.21-21.73)	18.58 (17.48 – 31.28)	0.2564 ^a^

Φ Kruskal-Wallis, θ Chi-squared, a Mann-Whitney U, Β Fisher’s exact *Flow cytometry data from one healthy volunteer did not meet internal quality standards. APACHE II: Acute physiology and chronic health evaluation II, SOFA: Sequential organ failure assessment, PF ratio: PaO2:FiO2 ratio

**Fig. 1 (abstract P368).**
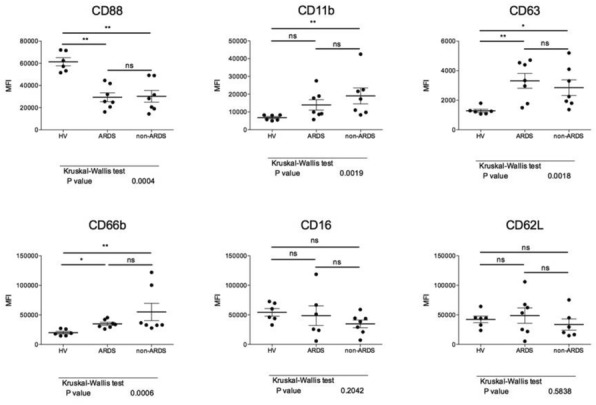
Neutrophil cell surface parameters. Overall Kruskal-Wallis values given with each graph. Multiplicity adjusted p values for individual comparisons shown with each parameter. ns: not significant, *: p<0.05, **: p<0.005

### P369 Global and regional diagnostic accuracy of lung ultrasound compared to computed tomography in patients with ARDS

#### M Umbrello^1^, GF Sferrazza Papa^2^, A Angileri^3^, M Gurgitano^4^, P Formenti^1^, S Coppola^1^, S Froio^1^, G Carrafiello^2^, D Chiumello^2^

##### ^1^Ospedale San Paolo - Polo Universitario, SC Anestesia e Rianimazione, Milano, Italy; ^2^Università degli Studi di Milano, Dipartimento di Scienze della Salute, Milano, Italy; ^3^Ospedale San Paolo - Polo Universitario, UO Radiologia Diagnostica e Interventistica, Milano, Italy; ^4^Università degli Studi di Milano, Scuola di Specializzazione in Radiodiagnostica, Milano, Italy

**Introduction:** Aim of the present study was to compare the global and regional diagnostic accuracy of Lung Ultrasound (LUS) compared to lung computed tomography (CT) scan in patients with the Acute Respiratory Distress Syndrome (ARDS). ARDS is characterized by a diffuse, inhomogeneous, inflammatory pulmonary edema. Lung CT scan is the reference imaging technique, but requires transportation outside the intensive care and exposes patients to X-rays. Lung ultrasound (LUS) is a promising, inexpensive, radiation-free, tool for bedside imaging.

**Methods:** Lung CT scan and LUS were performed at PEEP 5 cmH2O. LUS was performed using a standardized assessment of 6 regions per hemithorax: superior and inferior; anterior, lateral and posterior. Each region was classified for the presence of normally aerated, alveolar-interstitial syndrome, consolidation regions and pleural effusion. Agreement between the two techniques was calculated, and diagnostic parameters were assessed for LUS using lung CT as a reference. Both a global and a regional analysis were performed.

**Results:** Thirty-two sedated and paralyzed ARDS patients (age 65±14 years, BMI 25.9±6.5 Kg/m2 and PaO2/FiO2 139±47) were enrolled. Global agreement between LUS and CT was 0.811±0.032. The overall sensitivity and specificity of LUS are shown in Table 1. Similar results were found with regional analysis (anterior/lateral/posterior lung regions). The diagnostic accuracy of LUS was significantly higher when radiologic patterns assessed by CT, with a non-pleural extension were excluded (area under the ROC curve [95% CI]: alveolar-interstitial syndrome 0.854 [0.821 – 0.887] vs. 0.903 [0.852 – 0.954], p=0.049, and consolidation 0.851 [0.818 – 0.884] vs. 0.896 [0.862 – 0.929], p=0.044).

**Conclusions:** LUS is a reproducible, sensitive and specific tool which allows for bedside detections of the morphologic patterns in ARDS. The presence of deep lung alterations may impact the diagnostic performance of this technique

**Table 1 (abstract P369).** Global diagnostic accuracy of lung ultrasound

CT Scan Pattern	Sensitivity % [95% CI]	Specificity % [95% CI]	PPV % [95% CI]	NPV % [95% CI]
Normally areated	88.6 [75.4 – 96.2]	97.9 [95.8 – 99.2]	84.8 [71.1 – 93.7]	98.5 [96.6 – 99.5]
Alveolar-Interstitial	83.2 [74.6 – 90.9]	90.3 [87.9 – 93.0]	65.7 [54.8 – 77.1]	97.4 [95.0 – 98.9]
Consolidated	82.7 [78.4 – 86.8]	90.2 [88.9 – 93.2]	90.4 [86.0 – 95.2]	86.9 [81.7 – 91.0]
Pleural Effusion	92.3 [85.4 – 96.6]	98.6 [96.4 – 99.6]	96.0 [90.1 – 98.9]	97.2 [94.5 – 98.8]

### P370 Diagnostic specificity of PVPI in major abdominal and lung surgery

#### A Rieß, A Johannsen, P Friederich

##### München Klinik Bogenhausen, Department of Anaesthesiology, Critical Care and Pain Therapy, München, Germany

**Introduction:** Extravascular Lung Water Index (EVLWI) as well as increased Pulmonary Vascular Permeability Index (PVPI) have been claimed to be of diagnostic value in acute respiratory distress syndrome (ARDS) and acute lung injury (ALI). Their normal range values have been claimed to be 3-7 ml/kgPBW (EVLWI) [1] and < 3 (PVPI) although this has not been evaluated.

**Methods:** We investigated patients who either underwent major abdominal (n=71) or lung surgery (n=50) after induction of anaesthesia before beginning of surgery. Using the PiCCO2 device (Maquet Getinge Group, Rastatt, Germany) we obtained EVLWI and PVPI. Additionally, we performed ROC analysis against a cohort of critically ill patients treated on our ICU that suffered ARDS (n=20) diagnosed by pathological CT and PaO2/FiO2<300. Data presented as median [95% IQR].

**Results:** In the abdominal surgery group without ARDS EVLWI was 8 [7; 9] ml/kgPBW and PVPI was 1.6 [1.4; 1.8]. In the lung surgery group without ARDS EVLWI was 8 [7; 9] ml/kgPBW and PVPI was 1.9 [1.5; 2.2]. ROC analysis yielded specificity of 100% for PVPI > 3.3 in abdominal surgery patients and PVPI > 4.05 in lung surgery patients. Further analysis showed 100% specificity for EVLWI > 15 ml/kgPBW in abdominal surgery and EVLWI > 21 ml/kgPBW in lung surgery.

**Conclusions:** The previously claimed normal ranges for EVLWI of 3-7 ml/kgPBW were not met in the investigated patients. In patients undergoing major abdominal surgery a PVPI > 3.3 and in patients undergoing lung surgery a PVPI > 4.05 showed a specificity of 100% against a cohort of critically ill patients independently diagnosed with ARDS. Therefore, the high diagnostic specificity of PVPI for ARDS and ALI in patients undergoing major surgery warrants further investigation.

**Reference**

[1] Sibbald WJ et al. Chest 1983;83:725-31

### P371 Effect of neuromuscolar blocking agents on end expiratory lung impedance in moderate-severe ARDS

#### A Grassi^1^, C Giovannoni^1^, A Borgo^1^, D Albiero^2^, G Bellani^1^, G Foti^2^

##### ^1^University of Milan-Bicocca, Medicine and Surgery, Monza, Italy; ^2^San Gerado Hospital, Department of Anesthesiology and Intensive Care Medicine, Monza, Italy

**Introduction:** The use of Neuromuscular Blocking Agents (NMBA) in severe ARDS patients was shown to improve outcome [1]. The reasons of this finding are still under study. Recent observations on our patients suggest that NMBA might have a role in contrasting the reduction in End Expiratory Lung Volume (EELV) due to the activation of expiratory muscle. Objective of this study is to verify the effect of NMBA on End Expiratory Lung Impedance (EELI), used as surrogate of EELV, in moderate-severe ARDS

**Methods:** Patients affected by moderate-severe ARDS and undergoing controlled mechanical ventilation with muscle paralysis were enrolled. Exclusion criteria were pregnancy and contraindications to EIT belt positioning. Minimizing the dose of NMBA by daily interruption is a common practice in our ICU. During the interruption EIT belt was positioned. When the presence of spontaneous breathing activity was evident by clinical assessment and ventilator traces analysis, NMBA were administered to reach full paralysis, in accordance with the treating physician. EIT tracing were analyzed offline and the change in EELI after NMBA bolus, as compared to before NMBA administration, was measured. Respiratory mechanics and arterial blood gas (ABG) data were collected

**Results:** We enrolled 5 ARDS patients, undergoing controlled mechanical ventilation with muscle paralysis. Baseline respiratory mechanics and ABG data are shown in Table 1. In 4 out of 5 patient the bolus of NMBA led to an increase of EELI. In 1 case, the NMB administration led to no changes in EELI. The mean change in EELI was 159±152ml

**Conclusions:** In our small population of ARDS patients, the administration of a bolus of NMBA after the regain of spontaneous breathing activity led to an increase in EELI in 4 out of 5 patients. Further study are needed to 1) correlate this increase to global and regional respiratory system compliance and 2) correlate this increase to the time needed to wean the patient from NMBA

**Reference**

[1] Papazian L et al. NEJM 363:1107-16, 2010

**Table 1 (abstract P371).** Respiratory mechanics and arterial blood gas in the enrolled patient population

Vt (ml/kg)	5.2 ± 2.1
PEEP (cmH2O)	15.8 ± 2
Compliancers (ml/cmH2O)	49.7 ± 17,3
Driving Pressure (cmH2O)	8.5 ± 1.6
RR (bpm)	22.6 ± 8.1
PaO2/FiO2 ratio	181.3 ± 34
pH	7.42 ± 0.05
pCO2	57.3 ± 4.5

### P372 Use of the orthostatic board as an additional resource for the treatment of acute respiratory distress syndrome

#### P Travassos, R Vale, W Geres, É Teixeira, L Coscrato, V Veiga, S Rojas

##### Hospital BP - A Beneficência Portuguesa De São Paulo, Neurocritical Care Unit, Sao Paulo, Brazil

**Introduction:** To analyze the use of the orthostatic board as an auxiliary device for the treatment of severe ARDS by assessing its risks and benefits.

**Methods:** We selected 91 patients, 43 females and 48 males, hospitalized in a Neurological ICU, between June 2014 and July 2018, in a physiotherapeutic follow-up with diagnosis of severe ARDS. The patients were submitted to orthotics assisted for 40 to 60 minutes and monitored HR, PAM, FR, SatO2 at 30 ° and 60 ° of inclination and the PaO2 / FiO2 ratio after the procedure. The mean number of sessions per patient was 6.6. All patients were undergoing anticoagulation in RASS -5, in the treatment of the cause of ARDS. The mean time of mechanical ventilation was 8.5 days.

**Results:** Among the patients selected, 36.3% presented tachycardia above 115 bpm, requiring intervention in 12.1% and interruption of the procedure in 6.6%. PAM arterial hypotension <65 mmHg was observed in 34.1%, requiring intervention (increase of vasopressor dose and / or change of plank angulation) in 22% and interruption of the procedure in 14.3%. Hypoxemia SatO2 <92% was observed in 8.8%, without interruption, but an improvement in PaO2 / FiO2 was observed in only 95.6% of the patients.

**Conclusions:** Assisted orthostatism as an auxiliary device for the treatment of severe ARDS was shown to be an alternative, with improvement of PaO2 / FiO2 in 95.6% of the patients, safe and without significant hemodynamic repercussions that could lead to interruption of the procedure.

### P373 Transpulmonary pressure guided open lung concept in patients with severe ARDS can avoid vvECMO

#### P Van der Zee, H Endeman, D Gommers

##### Erasmus MC, Adult Intensive Care, Rotterdam, Netherlands

**Introduction:** The EOLIA trial found that vvECMO compared to conventional mechanical ventilation (CMV) did not improve mortality in patients with severe ARDS [1]. The CMV strategy consisted of airway pressures below 30cmH_2_O. In patients with severe ARDS higher airway pressures are required to maintain lung aeration. Grasso et al. measured the transpulmonary pressure (P_L_) in patients with severe ARDS and increased PEEP until P_L_ was 25cmH_2_O, accepting airway pressures above 30cmH_2_O. Fifty percent of patients responded to an increase in airway pressure and did not require vvECMO [2]. We hypothesized that a P_L_ guided open lung concept (OLC) improves oxygenation and prevents conversion to vvECMO in patients with severe ARDS.

**Methods:** A retrospective study was conducted in a tertiary referral ICU. The records of patients referred to our ICU for advanced medical care were reviewed. Inclusion criteria were severe ARDS according to the Berlin definition and the EOLIA trial inclusion criteria for vvECMO.

**Results:** Mechanical ventilation was limited to a P_L_ of <25cmH_2_O instead of plateau pressures below 30cmH_2_O. The P_L_ guided OLC resulted in an increase in P/F ratio and none of the patients required vvECMO. During the first 6 hours peak airway pressure was increased, but was reduced within 24 hours while PEEP was maintained (Fig. 1). At 72 hours both peak airway pressures and PEEP were reduced to baseline values while P/F ratio remained stable. Only one patient (12.5%) died of disseminated invasive aspergillosis.

**Conclusions:** The P_L_ guided OLC improved oxygenation and none of the patients required vvECMO. These findings support a ventilation strategy guided by transpulmonary pressures instead of plateau pressures in patients with severe ARDS.

**References**

1. Combes A et al, NEJM 2018, 378:1965-1975.

2. Grasso S et al, ICM 2012, 38:395-403.

**Fig. 1 (abstract P373).**
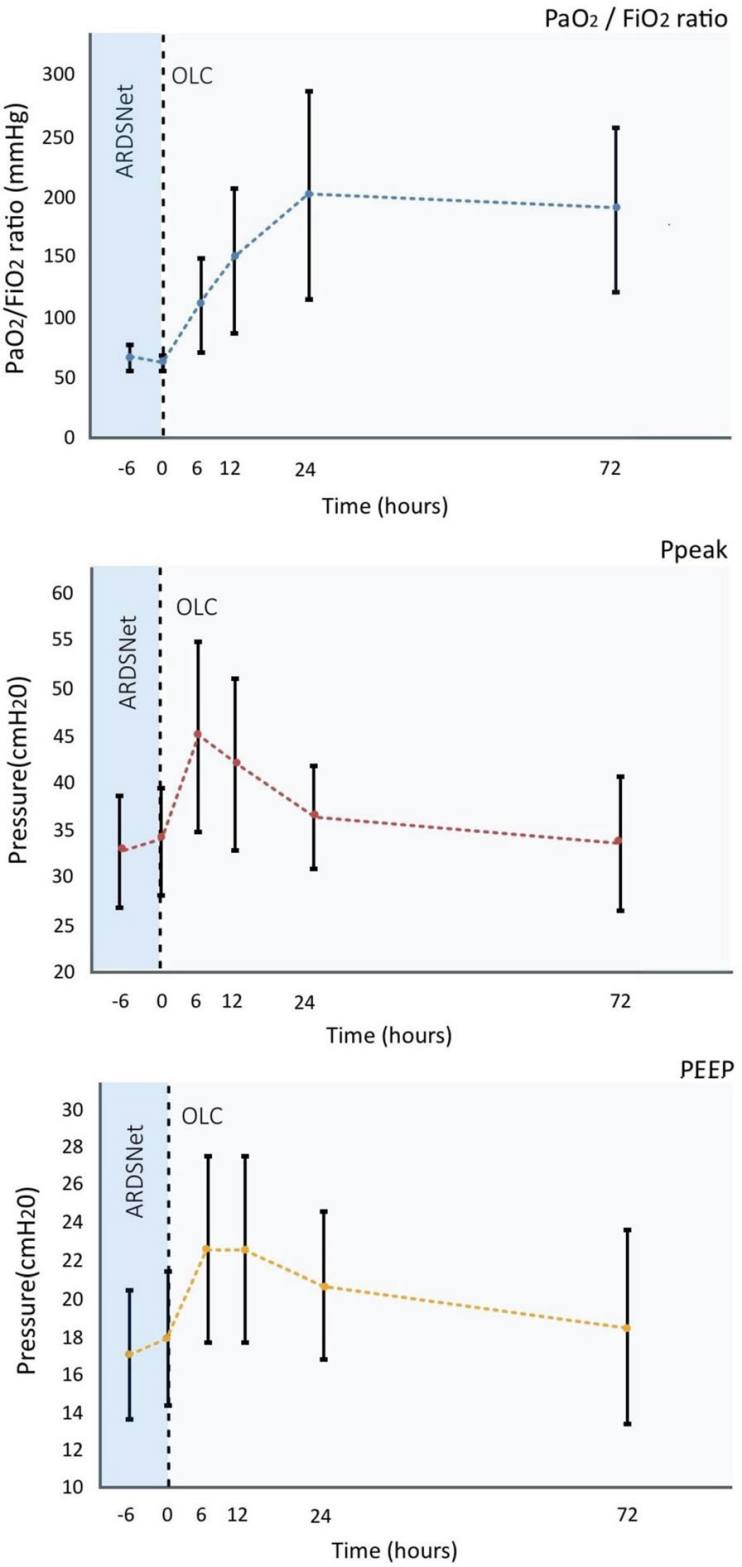
Oxygenation and airway pressure

### P374 Case series on airway pressure release ventilation (APRV) in prone position in the critical care setting

#### SJ Lee, YL Lee, A Kong, SY Ng

##### Singapore General Hospital, Department of Surgical Intensive Care Unit, Singapore, Singapore

**Introduction:** The mortality benefit conferred by early prone positioning in the treatment of acute respiratory distress syndrome (ARDS) has been well established. We also know that APRV improves oxygenation, and more recently has been shown to reduce ventilator dependent days and ICU length of stay [1,2]. However, controlled ventilation remains the mainstay mode of ventilation used during prone position. Literature looking at combined APRV and prone positioning is scarce. We aim to explore and report our institutional experience with respect to feasibility and outcomes in combining APRV and prone positioning, and perform a literature review in this area.

**Methods:** We undertook a single-centre retrospective cohort study within a surgical ICU of a tertiary hospital in Singapore between Jan 2013 – Oct 2017. Patients with ARDS who received combined prone positioning and APRV were reviewed retrospectively. A literature review of patients with ARDS who received combined intervention was also performed.

**Results:** 5 adult patients aged 21-77 years old diagnosed with ARDS received a combination of APRV and prone positioning for a duration of 16-45 h (Table 1). All the patients tolerated APRV with prone positioning well. Our patients saw an improvement of P:F ratio ranging from 59-225 upon completion of combination therapy. 3 out of 5 patients were extubated within 72 hours of turning supine, 1 was weaned to tracheostomy mask after 14 days and 1 died while on the ventilator. Only 1 case report and 1 randomized clinical trial were found on this topic upon literature review, which corroborated our findings.

**Conclusions:** In our experience, APRV is a practical and feasible alternative mode of ventilation that can be employed in the prone position, yielding significant P:F ratio improvements. The synergistic effects on improving oxygenation herald potential, especially in the subset of severe ARDS patients with refractory hypoxemia, where extracorporeal membrane oxygenation is unsuitable or unavailable.

**References**

1. Habashi NM. Crit Care Med. 2005;33:S228–40.

2. Zhou YF, et al. Intensive Care Med 2017; 43:1648-1659

**Table 1 (abstract P374).** Baseline characteristics

		Patient 1	Patient 2	Patient 3	Patient 4	Patient 5
Baseline characteristics	Age (y)	77	21	53	41	71
	Gender	M	M	M	M	M
	APACHE II	17	17	20	21	23
ARDS	Etiology	Pneumonia	Asp pneumonitis	Sepsis	Pneumonia	Sepsis
	Days of intubation before prone	0	9	12	6	0
	P:F ratio on ICU admission	88	95	222	85	39
Details of therapy	Total duration of prone (hr)	25	45	20	28	16
	P:F ratio before prone	156	91	53	56	38
	P:F ratio after turning supine	252	152	278	115	260
	Days to extubation (after turning supine)	3	2	26	not extubated	2
Outcomes	30-day mortality	No	No	No	Yes	No
	Duration of mechanical ventilation (days)	5	13	27	20	4
	LOS ICU (days)	9	14	29	20	5
	LOS hospital (days)	105	22	303	19	12
	Readmission to ICU	Yes	No	Yes	No	No
	Complications	None	None	None	Hypotension	Hypotension

LOS= Length of stay; APACHE II= Acute physiology and chronic health evaluation

### P375 Effects of prone positioning on venous return determinants and mean systemic pressure in patients with acute respiratory distress syndrome

#### C Lai, I Adda, JL Teboul, L Guérin, C Richard, X Monnet

##### Hôpitaux universitaires Paris-Sud, Hôpital de Bicêtre, Médecine Intensive - Réanimation, Le Kremlin-Bicêtre, France

**Introduction:** In acute respiratory distress syndrome (ARDS), prone positioning (PP) increases cardiac preload, but its effects on the determinants of systemic venous return (mean systemic pressure (Pms) and resistance to venous return (Rvr)) are unknown.

**Methods:** We included 19 patients with ARDS requiring PP. Prior to PP, preload reserve was assessed by a passive leg raising test or an end-expiratory occlusion test. Hemodynamic measurements, including cardiac index (CI), central venous pressure (CVP), Pms and Rvr (heart-lung interactions method), and intra-abdominal pressure (IAP) were measured in semi-recumbent position, supine horizontal position (in 12 patients) and 15 minutes after starting PP.

**Results:** PP significantly increased IAP (14±4 to 19±4mmHg), Pms (24±9 to 34±11 mmHg) and Rvr (1.9 (1.4-2.8) to 2.8 (2.3-3.7) mmHg.min.L-1). In 6 patients, CI increased >15% during PP. All these patients had preload reserve. Their Pms-CVP gradient increased by 74% (60%-176%) and the Rvr by 44% (33%-92%). In the other patients, CI did not increase >15% during PP. Ten of these patients had no preload reserve before PP. Their Pms-CVP gradient did not change during PP. The other three patients were preload-dependent at baseline. Their Pms-CVP gradient increased by 47%, but Rvr increased by 94%, suggesting conditions of Takata zones 1. The transition from the semi-recumbent to the supine horizontal position increased Pms without significantly modifying Rvr. In patients with preload reserve, this increased the Pms-CVP gradient and CI, unlike in patients without preload reserve.

**Conclusions:** PP increases Pms, CVP, Rvr and IAP. CI increases only if patients have preload reserve and if the increase of Rvr is less than the increase of the Pms-CVP gradient. The hemodynamic effects of PP are related to the increase of IAP during the transition to PP, but also to the increase of the Pms during the transfer from the semi-recumbent position to supine horizontal position.

### P376 Clinical outcomes according to the configurations of cannula in patients with acute respiratory distress syndrome under veno-venous extracorporeal membrane oxygenation: a Korean multicenter study

#### S Lim^1^, S Hong^2^, C Chung^3^, K Jeon^4^, S Lee^5^, W Cho^6^, S Park^7^, Y Cho^1^

##### ^1^82, Gumi-ro 173beon-gil, Bundang-gu, Gyeonggi-do, Division of Pulmonary and Critical Care Medicine, Department of Internal Medicine, Seongnam-si, South Korea; ^2^Asan Medical Center, Department of Pulmonary and Critical Care Medicine, Seoul, South Korea; ^3^Samsung Medical Center, Department of Critical Care Medicine, Seoul, South Korea; ^4^Samsung Medical Center, Department of Critical Care Medicine, Seoul, South Korea; ^5^Seoul National University Hospital, Division of Pulmonary and Critical Care Medicine, Department of Internal Medicine, Seoul, South Korea; ^6^Pusan National University Yangsan Hospital, Department of Internal Medicine, Yangsan-si, Gyeongsangnam-do, South Korea; ^7^Hallym University Sacred Heart Hospital, Division of Pulmonary and Critical Care Medicine, Department of Medicine, Anyang-si, Gyeonggi-do, South Korea

**Introduction:** The recirculation during veno-venous extracorporeal membrane oxygenation (VV ECMO) had been a drawback, which could limit sufficient oxygenation. Purpose of this study is to compare the short-term oxygenation in acute respiratory distress syndrome (ARDS) patients under VV ECMO according to their cannula configurations, especially in the national environment of the absence of newly developed double-lumen, single cannula.

**Methods:** Data were retrospectively analyzed from the severe ARDS patients receiving VV ECMO from 2012 to 2015 at six hospitals. Primary outcomes were PaO2 at 1, 4, and 12 hours after initiation of ECMO and cannula related complications.

**Results:** We included 335 patients and divided them into two groups according to the sites of return cannula; 157 patients were cannulated to femoral vein, and 178 patients to internal jugular vein. Baseline characteristics at admission including PaO2 were similar between two groups. PaO2 in 1 hour of the femoral group was higher than the jugular group (190.3 vs. 160.0, P = 0.108), however, there was no significant difference in the increments of PaO2 including 4 and 12 hours after initiation of ECMO between two groups. Additionally, the decrements of PaCO2 at 4 and 12 hours were lower in the jugular group. Mortality rate at 180 days after ECMO was not significantly different by the site of cannulation. Mechanical complications realted to cannulation also did not show significant difference, however femoral-jugular group showed a higher incidence of ECMO catheter-related blood stream infection (30.5% vs 15.7%, p=0.008).

**Conclusions:** Regardless of cannula configurations, ARDS patients under VV ECMO showed comparable clinical outcomes in terms of short-term oxygenation. However, the incidence of ECMO catheter-related blood stream infection was higher in femoral-jugular configuration.

### P377 Veno-venous extracorporeal membrane oxygenation (VV-ECMO) as a bridge to airway stenting

#### E Garry^1^, B Cusack^1^, E Carton^1^, K Redmond^2^, J Hastings^1^, I Conrick-Martin^1^

##### ^1^The Mater Misericordiae University Hospital, Department of Critical Care Medicine, Dublin, Ireland; ^2^The Mater Misericordiae University Hospital, Department of Cardiothoracic Surgery, Dublin, Ireland

**Introduction:** VV-ECMO is most commonly used in severe potentially reversible respiratory failure. This report looks at two patients in whom VV-ECMO was used to facilitate surgical airway stenting.

**Methods:** Case 1- A 56-year-old with recurrent respiratory arrests, on a background of Neurofibromatosis Type 1 and kyphoscoliosis. He had complex airway pathology, including, airway neurofibromas and granulation tissue, tracheobronchomalacia, severe kyphoscoliosis and a permanent tracheostomy tube. Rigid bronchoscopy was performed and following debridement of granulation tissue, a trouser-leg stent was deployed.

Case 2- A 75-year-old with progressive stridor due to recurrence of a malignant melanoma, which was causing mid-lower tracheal compression. Three tracheal stents were deployed via a rigid bronchoscope. In both cases, percutaneous bi-femoral VV-ECMO was established prior to general anaesthesia and decannulation took place the following day.

**Results:** In these cases, VV-ECMO provided stable extracorporeal gas exchange without conventional tracheal intubation. Cardio-pulmonary Bypass and Veno-Arterial ECMO have been described in patients at risk of compression of the heart and distal airway [1]. However, if the major threat is airway collapse, VV-ECMO can provide cardio-respiratory support without the problems associated with arterial cannulation and with lower anticoagulation requirements.

**Conclusions:** In carefully selected patients with large airway disease, establishment of VV-ECMO perioperatively can be beneficial. These cases demonstrate the technical feasibility and efficacy of VV-ECMO as a bridge to airway intervention.

**Consent:** Informed consent to publish has been obtained from the patients

**Reference**

1. SenDasgupta C, et al. Indian J Anaesthesia 54:565–568, 2010.

### P378 Structured implementation of a vvECMO service in a non university hospital

#### A Faltlhauser, M Argauer, D Cold, F Kullmann

##### Klinikum Weiden, Medizinische Klinik 1, Interdisciplinary ICU, Weiden in der Oberpfalz, Germany

**Introduction:** Klinikum Weiden cares for a population of app. 230000 as a sole tertiary care provider. The distance to the next ECMO service is 90km. In recent years we faced the reality, that not all of our patients with vvECMO indication had access to an ECMO service.

**Methods:** Inital determination of demand (19pts in 2016) and SWAT analysis based on ELSO criteria for the implementation of an ECMO Service led to a GANTT-Chart for the implementation process based on the German AWMF Guidelines 12/2017 [1]. All stakeholder needs (e.g. Staffing, resoruces, logistics, safety including crisis Management, cost benefit analysis) - were included. An internal quality control a risc management system (DIN ISO 2015 (3)) was established.

**Results:** We report data from the first year vvECMO Service at KW: 17 Patients fulfilled ELSOvvECMO criteria [2]. 10 were treated with vvECMO (ARDS pulmonary 5, nonpumonary 5). Mean SOFA Score was 18.2, mortality 50%, time on ECMO mean was13 (6-37) days. Weaning from ECMO was successful in 7 patients. vvECMO did not change our core clinical practice: Early Mobilisation from day two post canulation, prone position and kinetic therapy and our non sedation policy were established as in all of our other ICU patients as well.

**Conclusions:** Once all structural prerequisits are in place, staff is adequately trained and policies for risk and crisis management are implemented, vvECMO can be performed safely and successfully in a non University setting. It is more the knowledge of how to treat the disease process that led to the vvECMO indication, than the ECMO itself that limits outcome. Therefore indication and timing for vvECMO are crucial.

**References**

(1) ELSO Guidelines for Cardiopulmonary Extracorporeal Life Support, v.1.4 Aug 2017; elso.org

(2) S3-Leitlinie Invasive Beatmung und Einsatz extrakorporaler Verfahren bei akuter respiratorischer Insuffizienz 1.Auflage, 12/2017; www.awmf.de

(3) DIN ISO 2015; www.zertpunkt.de

### P379 Extracorporeal carbon dioxide removal (ECCO2R) requirements for (ultra)protective mechanical ventilation: mathematical model predictions

#### J Leypoldt^1^, J Goldstein^2^, D Pouchoulin^3^, K Harenski^4^

##### ^1^Unaffiliated, None, San Clemente, United States; ^2^Baxter World Trade SPRL, Acute Therapies, Braine-l’Alleud, Belgium; ^3^Gambro Industries, Research & Development, Meyzieu, France; ^4^Baxter International, Acute Therapies, Munich, Germany

**Introduction:** ECCO2R facilitates the use of low tidal volumes during protective or ultraprotective mechanical ventilation when managing patients with acute respiratory distress syndrome (ARDS); however, the rate of ECCO2R required to avoid hypercapnia remains unclear.

**Methods:** We determined ECCO2R requirements to maintain arterial partial pressure of carbon dioxide or CO2 (PaCO2) at clinically desirable levels in ventilated ARDS patients using a six-compartment mathematical model of CO2 and oxygen (O2) biochemistry [1] and whole-body transport [2] with the addition of an ECCO2R device for extracorporeal veno-venous removal of CO2. The model assumes steady state conditions and is comprehensive from both biochemical and physiological perspectives. O2 consumption and CO2 production rates were assumed proportional to predicted body weight (PBW) and adjusted to achieve PaO2 and PaCO2 levels at a tidal volume of 7.6 mL/(kg of PBW) as reported in LUNG SAFE [3]. Clinically desirable PaCO2 levels during mechanical ventilation were targeted at 46 mm Hg for a ventilation frequency of 20.8/min as previously reported [3].

**Results:** Model simulated PaCO2 levels without and with an ECCO2R device at various tidal volumes are tabulated in Tables 1 and 2, respectively. Table 1 shows a substantial increase in PaCO2 at a tidal volume of 6 mL/(kg of PBW) that is more pronounced when further reducing the tidal volume. Additional simulations showed that predicted ECCO2R rates were significantly influenced by ventilation frequency.

**Conclusions:** The current mathematical model predicts that ECCO2R rates that achieve clinically acceptable PaCO2 levels at tidal volumes of 5-6 mL/(kg of PBW) can likely be achieved with current technologies; achieving such PaCO2 levels with ultraprotective tidal volumes of 3-4 mL/(kg of PBW) may be challenging.

**References**

1. Rees et al. Crit Rev Biomed Eng 33:209-264, 2005

2. Andreassen et al. Crit Rev Biomed Eng 33:265-298, 2005

3. Bellani et al. JAMA 315:788-800, 2016

**Table 1 (abstract P379).** Model Predictions without ECCO2R Device

Tidal Volume (mL/kg of PBW)	PaCO2 (mm Hg)	ECCO2R Rate (mL of CO2/min)
6	58	0
5	69	0
4	86	0
3	115	0

**Table 2 (abstract P379).** Model Predictions with ECCO2R Device

Tidal Volume (mL/kg of PBW)	PaCO2 (mm Hg)	ECCO2R Rate (mL of CO2/min)
6	46	51
5	46	84
4	46	117
3	46	151

### P380 Experience of an ECMO team with VV ECMO for acute respiratory distress syndrome - success factors

#### P Travassos, W Geres, R Vale, R Schneidwind, J Souza, V Veiga, S Rojas

##### Hospital BP - A Beneficência Portuguesa De São Paulo, Neurocritical Care Unit, Sao Paulo, Brazil

**Introduction:** To evaluate the predictors of successful venous ECMO in patients with ARDS, refractory to optimized treatment.

**Methods:** Patients were retrospectively assessed from February 2014 to May 2018 to identify predictors of success. There were 13 men and 8 women with a mean age of 55.18, using the ESLO protocol for the ECMO Veno-Venosa indications. Among the causes of ARDS: pneumonia (42.86) and non-pulmonary focus (57.14%). The primary end point was hospital discharge. The variables evaluated were: Age, SOFA, MURRAY score, thrombocytopenia and the time of cannulation up to 72 hours after Orotraqueal intubation

**Results:** Of the patients evaluated, 42.86% were discharged from hospital. Among the variables related to prognosis, the Murray score (4.75 for hospital discharge vs 8.4 for death - Fisher 0.00035 and P <0.05), platelets> 150,000 at the time of implantation (85.71% for hospital discharge vs 21.43% for death - Fisher 0, 005414 and P <0.05) and the moment of implantation within 72 hours after orotracheal intubation (64.28% for hospital discharge vs 100% for death - Fisher 0.017534 and <0.05)

**Conclusions:** In our series, we found difference of results in 3 variables described. Patients with early indication, platelets> 150000 and Murray index had correlation with outcome.

### P381 Implications of immunosuppression in patients with ECMO therapy

#### J Rilinger, PM Biever, D Staudacher, D Duerschmied, C Bode, T Wengenmayer

##### Heart Center Freiburg University, Faculty of Medicine, University of Freiburg, Department of Cardiology and Angiology I, Freiburg, Germany

**Introduction:** Mortality in ARDS patients is high even with veno-venous extracorporeal membrane oxygenation (VV-ECMO) support. Immunosuppressed patients are considered to be a high risk population. There is little evidence covering primary outcome and pathogen spectrum of immunosuppressed ARDS patients on VV-ECMO therapy.

**Methods:** We report retrospective registry data on all ARDS patients treated in-house with VV- ECMO at a university hospital between 09/2012 and 12/2017. In a systematic review of medical records survival, successful ECMO weaning and pulmonary pathogen spectrum (bacterial, viral, fungal and particularly Pneumocystis jirovecii) were investigated. Moreover, status of immunosuppression was assessed.

**Results:** A total of 164 patients (age 52.1 years, male 67.1%) with complete patient data were included in the analysis. 35.5% of the patients had underlying immunosuppression (immunosuppression due to autoimmune disease, organ transplantation, chemotherapy or hematopoietic stem cell transplantation in case of oncological disease or active HIV-infection). Survival in patients without immunosuppression was 45.3% vs. 20.7% in patients with immunosuppression (p=0.002, Odds ratio 0.32) and successful ECMO weaning rate was 53.8% vs. 34.5% (p=0.022, Odds ratio 0.45, Table 1), respectively. There were significant less bacterial infections in immunosuppressed patients but more viral and fungal infections and infections with Pneumocystis jirovecii (Table 1, Figure 1A). Pulmonary infections for each subtype of immunosuppression are shown in Figure 1B.

**Conclusions:** ARDS VV-ECMO patients with underlying immunosuppression have higher mortality rates and higher rates of ECMO weaning failure. Immunosuppressed patients suffer from a different spectrum of pulmonary infections in comparison to not immunosuppressed patients.

**Table 1 (abstract P381).** ECMO patients with vs. without immunosuppression

	No Immunosuppression N = 106 (64.5%)	With Immunosuppression N = 58 (35.5%)	p-value	Odds ratio, 95%-confidence interval
Survival	45.3%	20.7%	0.002	0.32 [0.15 - 0.66]
Successful ECMO weaning	53.8%	34.5%	0.022	0.45 [0.23 - 0.88]
Pulmonal infections				
- Bacterial	50.9%	15.5%	<0.001	0.18 [0.08 - 0.40]
- Viral	10.4%	41.4%	<0.001	6.10 [2.70 - 13.76]
- Fungal	12.3%	31.0%	<0.001	5.10 [2.30 - 11.26]
- Pneumocystis jirovecii	0.9%	22.4%	<0.001	30.33 [3.85 – 239.0]

**Fig. 1 (abstract P381).**
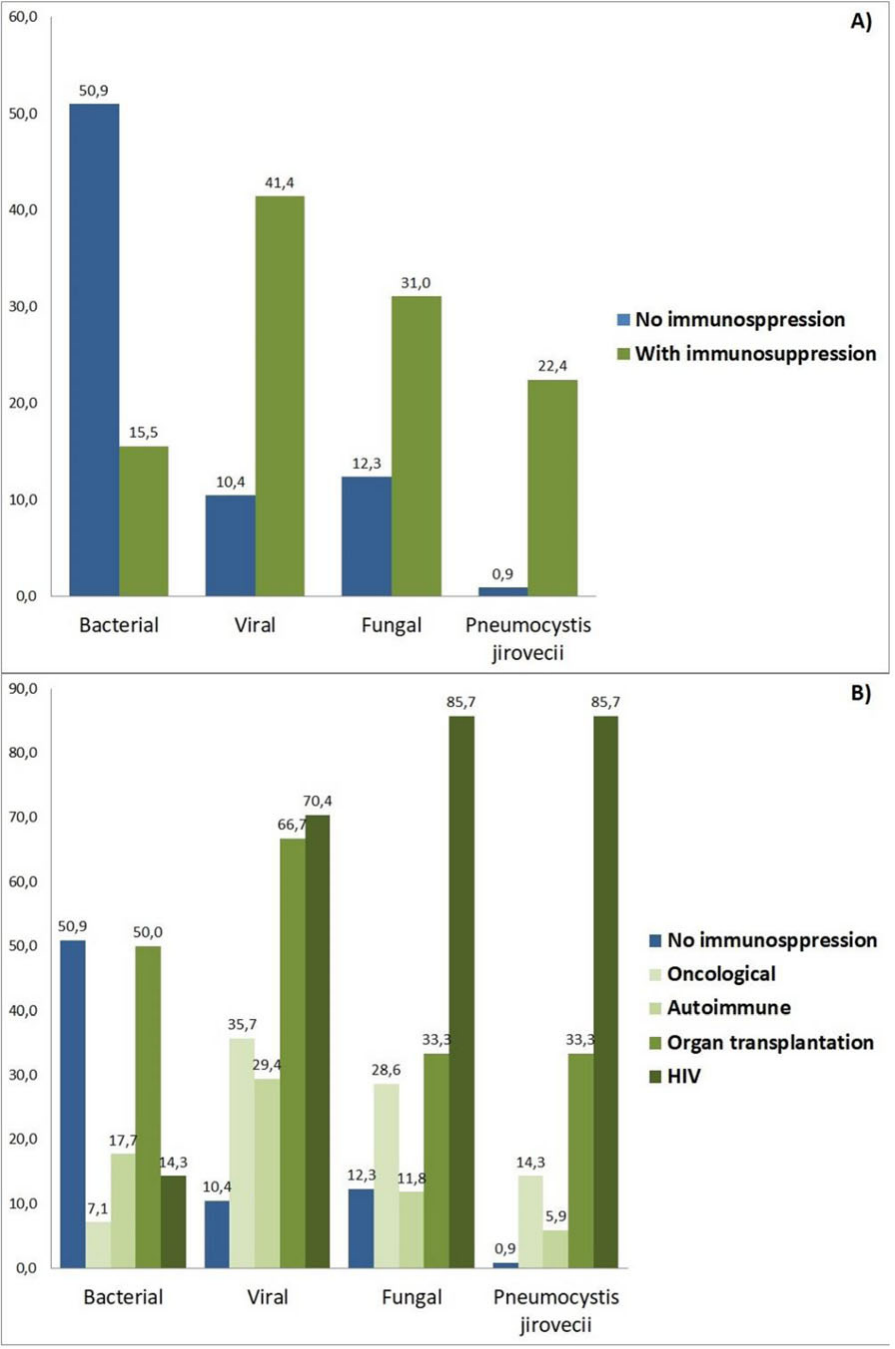
Pulmonary pathogen spectrum in ECMO patients, A) with vs. without immunosuppression, B) for each subtype of immunosuppression

### P382 Mode of death in ARDS patients requiring ECMO therapy

#### J Rilinger, D Staudacher, PM Biever, D Duerschmied, C Bode, T Wengenmayer

##### Heart Center Freiburg University, Faculty of Medicine, University of Freiburg, Department of Cardiology and Angiology I, Freiburg, Germany

**Introduction:** Mortality in ARDS patients receiving veno-venous extracorporeal membrane oxygenation (VV-ECMO) therapy is high. Little is known about the specific cause of death in these critical ill patients. Detailed analysis of mode of death might be beneficial for exploring limits of ECMO therapy.

**Methods:** We report retrospective registry data on all ARDS patients treated in-house with VV-ECMO at a university hospital between 09/2012 and 12/2017. In a systematic review of medical records, we discriminated between patients died during ECMO therapy and after ECMO weaning.

**Results:** A total of 164 patients (age 52.1 years, male 67.1%, immunosuppression 35.5%) with complete patient data were included in the analysis. Overall mortality was 63.4% (104 of 164 patients). 83.7% (87 patients) died while receiving ECMO therapy, 16.3% (17 patients) died after ECMO was weaned successfully. In the group of patients who died during therapy, 31.8% died because of ECMO weaning failure, 26.9% because of circulation failure and 6.7% because of persisting hypoxia. Death due to ECMO complications occurred in 1.9% of the cases. Neurological impairment was the reason for aborting therapy in 11.6%. After successful ECMO weaning 4.8% died due to circulatory failure and 6.7% of the patients died because of inability to wean from mechanical ventilation. In 4.8% of the cases, neurological impairment was the reason to abort therapy (Figure 1).

**Conclusions:** Mortality in ARDS patients treated with ECMO remains high. The vast majority of the patients died during extracorporeal therapy whereas inability to wean from mechanical ventilation occurred in very few cases. Effectivity of ECMO was high with low rates of remaining lethal hypoxia. Nevertheless, one third of patients receiving ECMO therapy died because of ECMO weaning failure underlining the importance of adequate patient selection.

**Fig. 1 (abstract P382).**
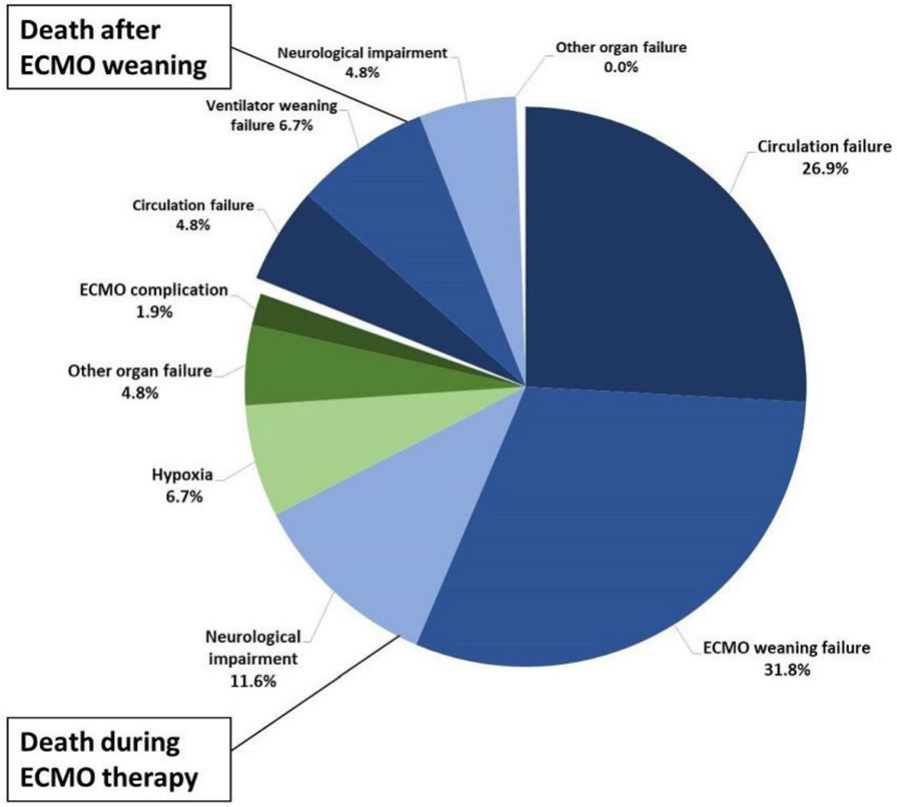
Distribution mode of death in ARDS patients requiring ECMO therapy

### P383 Non-invasive ventilation in children with acute severe asthma at a pediatric intensive care unit

#### G Hasan^1^, A Al-Eyadhy^2^, M Temsah^2^, A Alhaboob^2^, F Alsohime^2^, M Alabdulhafid^2^

##### ^1^Assiut Faculty of Medicine, Assiut University, Pediatric Intensive Care, Assiut, Egypt; ^2^King Saud University, College of Medicine, Assistant Professor of Pediatrics and Consultant Pediatric Intensivit, Riyadh, Saudi Arabia

**Introduction:** Acute asthma attack in children is a life-threatening emergency that requires urgent medical intervention. In the present study, we aim to clarify the effect of non-invasive ventilation (NIV) on the heart rate (HR), respiratory rate (RR), and fraction of inspired oxygen (FiO2) in children with acute severe asthma (ASA) who failed to respond to standard medical treatment; and to evaluate the associated complications and length of stay (LOS) at the pediatric intensive care unit (PICU).

**Methods:** This is a retrospective descriptive study of prospectively collected data. It was carried at the PICU of a tertiary university hospital, Saudi Arabia. The study included children ≤14 years old with ASA admitted to the PICU from November 2011 to November 2015 and required NIV. Outcome measures include the effect of NIV on the HR, RR, FiO2, and LOS.

**Results:** The study included 118 children with ASA and 52 (44%) of them required NIV. Of those 52 patients, 12 (23%) were excluded due to incomplete data, and 40 (34%) patients were included in the final analysis. They were 22 (55%) male and 18 (45%) female with a mean age of 66 months and a median Pediatric Index of Mortality 2 (PIM2) score of 6.3%. Of them, 28 (70%) had moderate asthma scores (≥5-9) and 12 (30%) had severe asthma scores (≥9). The median duration of NIV was 18 hours and the median LOS in the PICU was three days. At 6 hours, only RR showed a significant decrease compared to initiation of NIV (p-value <0.001) (Fig 1); while HR, RR, and FiO2 were significantly improved at 24 hours from initiation of NIV (p-value <0.001) (Fig 2).

**Conclusions:** Non-invasive ventilation, in association with standard medical treatment, was associated with clinical improvement in children with ASA not responding to standard medical treatment alone. NIV was not associated with significant complications or side effects.

**Fig. 1 (abstract P383).**
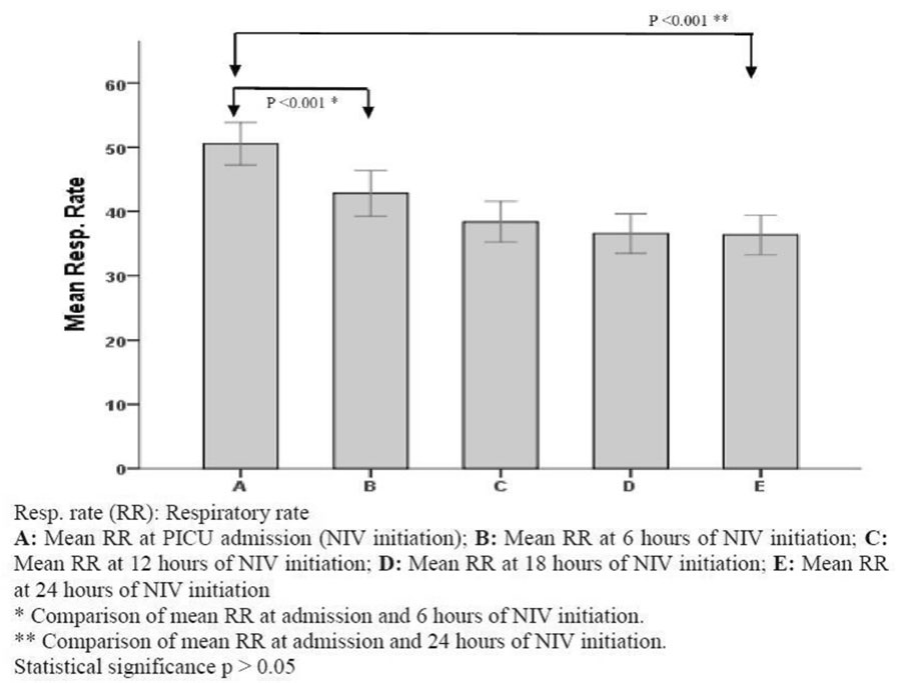
Changes in mean respiratory rate over the first 24 hours of non-invasive ventilation (NIV) initiation

**Fig. 2 (abstract P383).**
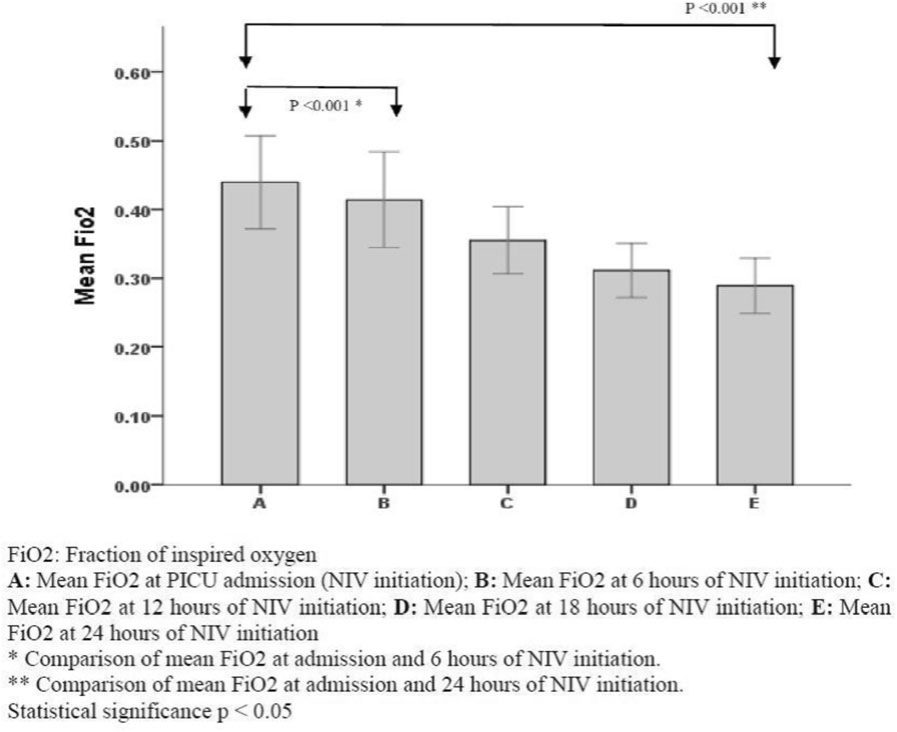
Changes in mean fraction of inspired oxygen (FiO2) over the first 24 hours of non-invasive ventilation (NIV) initiation

### P384 Neurally adjusted ventilatory assist as an alternative to pressure support ventilation in difficult-to-wean patients: a single center randomized trial

#### L Liu, Q Sun, H Qiu

##### Nanjing Zhongda Hospital, School of Medicine, Southeast University, Department of Critical Care Medicine, Nanjing, China

**Introduction:** Difficult weaning is a common problem of patients and result in prolonged weaning duration and poor outcome. Neurally adjusted ventilatory assist (NAVA) is a partial support ventilatory mode which triggers and tailors the level of assistance delivered by the ventilator to the electrical activity of the diaphragm. The objective of this study was to compare NAVA and pressure support ventilation (PSV) in patients who were difficult to wean.

**Methods:** A total of 99 difficult-to-wean patients who were able to sustained PSV in the critical care medicine unit (ICU) of the Zhongda Hospital, Southeast University were enrolled in the study (Fig 1). Patients were classified according to the reason for weaning failure and were randomly assigned to receive NAVA or PSV during weaning (Table 1). The primary outcome was the duration of weaning. Secondary outcomes included the proportion of successful weaning and patient-ventilator asynchrony.

**Results:** There were 17% (8/47) and 33% (17/52) patients in the PSV and in the NAVA group never weaned from mechanical ventilation (P = 0.073). The duration of weaning was significantly shorter in the NAVA group [2.4 (1.1-5.3) days], than in that in the PSV group [4.1(1.1-7.7) days] (P = 0.041). The proportion of patients with successful weaning was 70% (n=33/47) in NAVA group which was much higher than that in PSV group (48%, n=25/52) (Table 2). Compared with PSV, NAVA improved the rate of successful weaning in patients with single reason (74% vs. 49%, P = 0.019) but not in patients with multiple reasons for difficult weaning (50% vs. 45%, P = 0.656). NAVA decreased ineffective efforts and improved the trigger and cycling-off delays when compared with PSV. Mortality was similar in the two groups (Fig 2).

**Conclusions:** In patients who were difficult to wean, NAVA decreased duration of weaning and increased the probability of successful weaning. NAVA which improved patient-ventilator asynchrony, is safe, feasible and effective over a prolonged period of time during weaning.

**Table 1 (abstract P384).** Cause of difficult weaning

	PSV (n=52)	NAVA (n=47)	P
Cause of difficult weaning			
Airway and lung dysfunction, n (%)	33 (63)	29 (62)	0.857
Brain dysfunction, n (%)	1 (2)	1 (2)	1.000
Cardiac dysfunction, n (%)	5 (10)	6 (13)	0.573
Diaphragm/respiratory muscle function, n (%)	1 (2)	2 (4)	0.603
Multiple reasons, n (%)	11 (21)	8 (17)	0.602
Unclear, n (%)	1 (2)	1 (2)	1.000

PSV pressure support ventilation, NAVA neurally adjusted ventilatory assist

**Table 2 (abstract P384).** Primary outcome

	PSV (n=52)	NAVA (n=47)	P
Primary outcome			
Duration of weaning^a^, days	4.1(1.1-7.7)	2.4 (1.1-5.3)	0.041
other outcomes			
Successful weaning^b^, n (%)	25 (48)	33 (70)	0.040
Failed weaning n (%)	10(19)	6(13)	0.425
Patients never weaned after enrollment, n (%)	17 (33)	8 (17)	0.073
Hospital mortality, n (%)	25 (48)	16 (34)	0.157

^a^ Duration of weaning was defined as time from study enrollment to extubation or disconnection of the ventilator continuously for 24 hours or more in patients tracheotomized. Duration of weaning could be analyzed in 39 and 35 patients in NAVA and PSV groups respectively. ^b^ Successful weaning was defined as no need for re-intubation and invasive mechanical ventilation within 48 hours following extubation or continuously disconnection of ventilator for more than 48 hours in patients who were tracheotomized. PSV pressure support ventilation, NAVA neurally adjusted ventilatory assist

**Fig. 1 (abstract P384).**
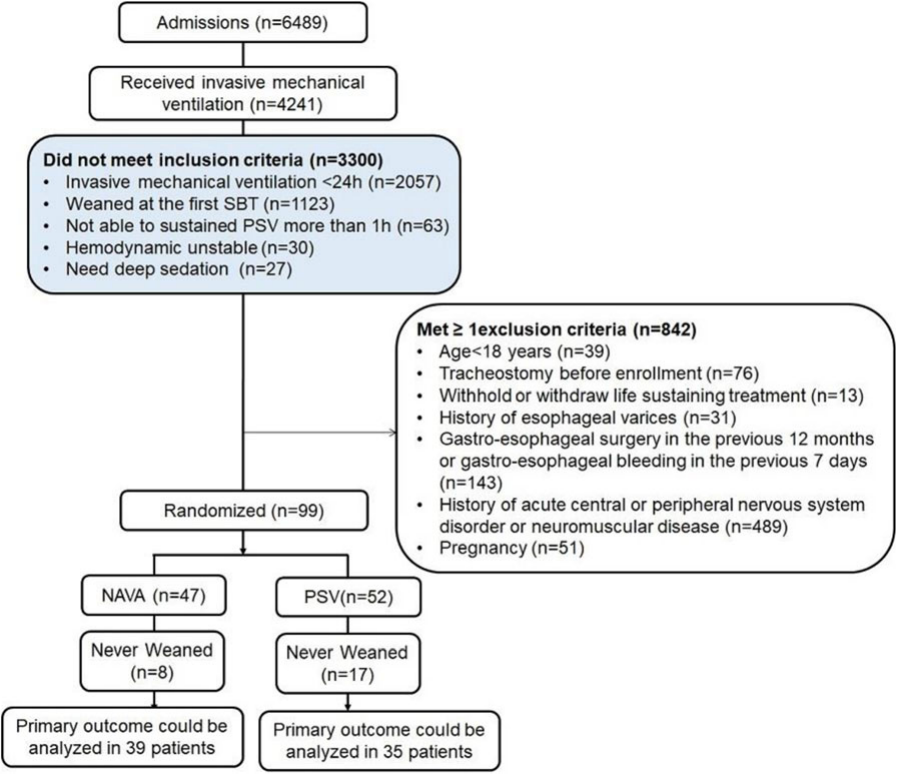
Flow diagram of the patient enrollment. NAVA neurally adjusted ventilatory assist, PSV pressure support ventilation

**Fig. 2 (abstract P384).**
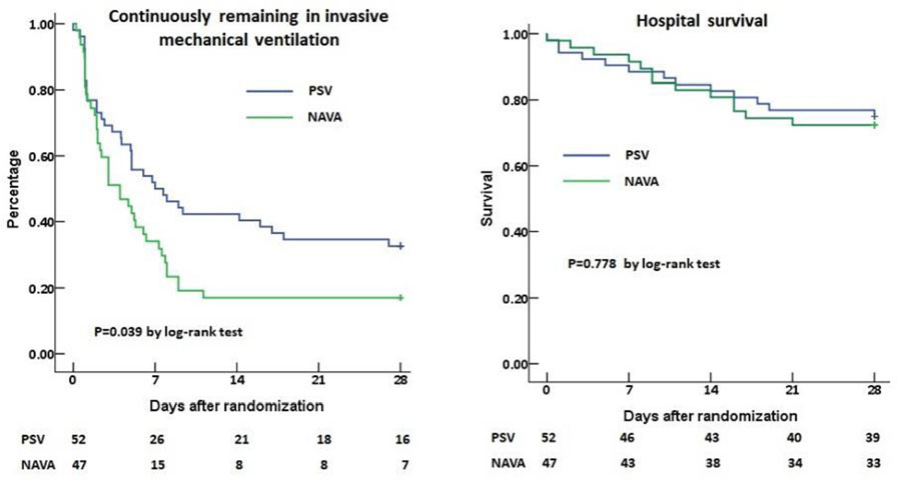
Kaplan-Meier estimates of probability of continuously remaining in invasive mechanical ventilation and survival during the 28 days following randomization. NAVA neurally adjusted ventilatory assist, PSV pressure support ventilation

### P385 Adherence to lung protective ventilation in patients admitted to surgical intensive care units in a tertiary care center in Thailand

#### A Piriyapatsom, A Trisukhonth, S Kongsareepong, O Chaiwat

##### Faculty of Medicine Siriraj Hospital, Anesthesiology, Bangkok, Thailand

**Introduction:** Lung protective ventilation (LPV) strategy is generally accepted as a standard practice in mechanically ventilated patients. The adherence rate to LPV strategy reported in literatures is 40% [1,2]. The aim of this study was to determine the mechanical ventilation (MV) management according to LPV strategy applied to patient admitted to SICU.

**Methods:** This was a prospective observational study, which included all adult patients admitted SICU and required MV support for more than 12 hours. Patients requiring MV support for more than 24 hours prior to SICU admission, those re-admitted to the SICU, those requiring non-invasive MV support, and moribund cases were excluded. The LPV strategy was defined as ventilation with tidal volume of <8 mL/kg of predicted body weight plus PEEP of at least 5 cm H2O applied to patients at SICU admission. Demographic and clinical data were recorded and analyzed.

**Results:** There were 149 patients included in this study. The mean age and mean APACHE II score were 65.3±14.1 years old and 12.6±5.5, respectively, and 65.1% were male. Half of patients (49.7%) were admitted to SICU following planned elective surgery and 73.2% required MV support following general anesthesia. The adherence rate to the LPV strategy was 37.6%. Independent factors associated with receiving LPV strategy were smoking (OR, 4.16; 95% CI, 1.95-8.87) and PaO2 (OR, 0.99; 95% CI, 0.99-1.00). Independent factors associated with SICU mortality included APCHE II score (OR, 1.24; 95% CI, 1.04-1.47), sepsis at SICU admission (OR, 5.27; 95% CI, 1.02-27.09) and LPV strategy (OR, 14.08; 95% CI 2.58-76.90).

**Conclusions:** The adherence rate to LPV strategy in patients admitted to SICU was 37.6%. Nevertheless, receiving LPV strategy was independently associated with SICU mortality. Whether this was just another marker of severity or this really caused such miserable outcome, further studies are warranted.

**References**

1. Umoh NJ et al. Crit Care Med. 2008;36:1463-8.

2. Santamaria JD et al. Crit Care Resusc. 2015;17:108-12.

### P386 Diaphragm excursion and contraction velocity in patients with grade I and II of intra-abdominal hypertension

#### V Hostić, M Širanović, M Vučić, B Rode, A Horvat, Ž Gavranović, L Videc Penavić, H Krolo Videka, A Gopčević

##### UHC Sestre milosrdnice, ICU, Department of Anesthesiology, Zagreb, Croatia

**Introduction:** The aim of this observational study was to determine diaphragmatic motility range using ultrasound (US) in patients with moderate range of intra-abdominal hypertension (IAH) following major abdominal surgery procedure after completing spontaneus breathing trial (SBT) protocol and possible validation of US in predicting successful weaning in patients with IAH.

**Methods:** Study enrolled 43 adult patients so far and all of them were successfully weaned from mechanical ventilation. We measured maximal altitude of diaphragmatic contractions and velocity of both hemi diaphragmatic poles using US sector probe, 4 MHz in both 2D and M mode as previously described [1] after completing SBT protocol. Patients with pulmonary and neuromuscular disseases were excluded. Measurements are expresed as mean value with p value of 0.05 considered significant. IBM SPSS v 20 was used for statistical analysis.

**Results:** Patients were divided into normal (8.7mmHg; n=11) and high (16.3 mmHg; n=32) group by the value of IAP. In normal group there was no difference in excursions between right and left hemidiaphragm (17.6 vs 21.5 mm; p=0.09) whereas there was difference in contraction velocity between sides (19.7 vs 19.2 mm/s; p=0.03). Regarding the high group, contraction velocity was equal between the right and left hemidiaphragm (21.3 vs 25.1 mm/s; p=0.15) but diaphragm contraction was higher at right hemidiaphragm (26.9 vs 25.0 mm; p=0.02). There was no difference between normal and high group in RSBI values (34.2 vs 40.8; p=0.35).

**Conclusions:** Current literature suggests that diaphragm US could be a useful and accurate tool to predicz extubation success or failure [2]. Preliminary results from our study suggest that the safe range of right diaphragm excursion for weaning the patients with IAH is 26.9 mm.

**References**

1. Boussuges A et al. Chest 135:391-400, 2009

2. Zambon M et al. Intensive Care Med 43:29-38, 2017

### P387 Handgrip strength does not predict spontaneous breathing trial failure or difficult or prolonged weaning of critically ill patients

#### G Friedman ^1^, P Fontella^1^, L Forgiarini^2^, T Lisboa^1^

##### ^1^UFRGS, PPG Ciências Pneumológicas, Porto Alegre, Brazil; ^2^Centro Universitário Metodista - IPA, Porto Alegre, Brazil

**Introduction:** Handgrip strength (HGS) is an alternative measure to assess peripheral muscle strength and is correlated with the Medical Research Council (MRC) scale, with promising values for diagnosing intensive care unit-acquired muscle weakness (ICUAMW) [1]. Since ICUAMW has been associated with delayed weaning from mechanical ventilation (MV) [2], we hypothesized that peripheral muscle strength evaluated by both the MRC scale score and HGS is independently associated with failure on the spontaneous breathing trial (SBT) and the duration of weaning from MV,

**Methods:** Design: A prospective observational study

Patients: Adult patients with more than 48 hours of MV who were eligible for weaning

Measurements: Evaluation for muscle strength by the MRC scale and HGS before each SBT.

Setting: Three general ICUs of 2 academic hospitals

**Results:** Before the first SBT, the MRC score (p<0.001) and HGS (p=0.010) were significantly different according to the type of ventilatory weaning. Between the SBT failure and success groups, only the MRC score was significantly different (p<0.001). In the multivariate analysis, only the MRC score was significantly associated with SBT failure (odds ratio [OR] 0.91, 95% CI 0.88-0.97, p=0.002) and difficult or prolonged weaning (OR 0.91, 95% 0.87-0.96, p=0.001). HGS exhibited good accuracy in identifying ICUAMW.

**Conclusions:** Only MRC score is independently associated with SBT failure and difficult or prolonged weaning. HGS is also associated with these two outcomes related to MV weaning and may serve as a simple tool to identify ICUAMW.

**References**

1. Ali NA et al. Am J Respir Crit Care Med 2008; 178:261

2. Cottereau G et al. Respir Care 2015; 60:1097

### P388 Minute ventilation as predictor of mortality in patients with hypoxemic respiratory failure undergoing non invasive ventilation

#### P Vargas, M Bozinovic, F Abbott, D Ramos, R Benavente, I Tike, D Cibilic, A Fuentes, M Ramirez

##### Hospital del Salvador, Unidad de Paciente Critico, Santiago, Chile

**Introduction:** There is evidence to support that in patients with hypoxemic respiratory failure (AHRF) under non invasive ventilation (NIV), high tidal volume (TV) and high respiratory rate (RR) are associated with NIV failure and possibly poor prognosis. We postulated that high minute ventilation (MV); or TV x RR; is associated with mortality in AHRF, when NIV is initiated.

**Methods:** Single-center, prospective and observational study. We included consecutives AHRF adults requiring NIV. AHRF was defined as acute dyspnea with new pulmonary infiltrates on chest radiography and PaCO2 below or equal to 45mmHg. We registered demographic and clinical parameters (including RR, MV, arterial blood gases, heart rate and blood pressure) at baseline and after 6 hours of first session of NIV, APACHE II score, diagnosis, need for intubation and ICU mortality. We performed a multivariate analysis to assess independent factors associated with mortality and ROC analysis

**Results:** 107 patients were enrolled, age 63 (±16.9). 61 were male (57%). APACHE II 14 (±7). Pneumonia (n=72; 67.3%) was the main etiology of AHRF. Length of ICU stay was 11.2 days (±10.6), 31 patients died in ICU (29%). MV was significantly higher (Univariate analysis) in non-survivors compared to survivors 18.5 l/m (±8) versus 12.9 l/m (±4.1) at baseline, and 16.7 l/m (±6) versus 12.5 l/m (±4) after 6 hours respectively. AUC to MV and mortality was 0.71 (CI 95% 0.59- 0.84), best cutoff was 16 l/m. MV was independently associated with mortality in multivariate analysis OR 1.25 (CI 95% 1.08 - 1.43) p<0.05.

**Conclusions:** MV after initiating NIV in AHRF was independent predictor of mortality, possibly reflecting major illness severity or worst NIV tolerance. It could then be considered as a useful and “logical” tool when it comes to decide if intubate and finally start invasive ventilation.

### P389 The 6-minute walk test (6MWT) in critically ill survivors of acute exacerbations of COPD (AE/COPD): does walk distance predict outcome and future exacerbations.

#### A Khedher, W Zarrougui, K Meddeb, M Zghidi, I Ben Saida, Maboujelben, N Fraj, A Azouzi, I Chouchene, M Boussarsar

##### Farhat Hached University Hospital, Medical Intensive Care Unit, Sousse, Tunisia

**Introduction:** The aim was to study the potential usefulness of the 6MWT performed the day of ICU discharge as a predictor of outcome and future exacerbations in patients admitted for AE/COPD.

**Methods:** A prospective longitudinal and observational cohort study was performed in a Tunisian medical ICU between February 2017 and March 2018, including all consecutive survivors of AE/COPD. The 6MWT is performed in accordance with international guidelines. Were collected: clinical features at admission, severity of illness and ICU course. The walked and predicted distances and their ratio (6MWT ratio) were calculated. Patients were followed up via phone calls 3 months after discharge. Area under the curve (AUC), sensitivity and specificity were used to assess test performance to predict mortality, future exacerbations and readmissions after 3 months of ICU discharge.

**Results:** Among 102 patients admitted for AE/COPD during the study period, 75(73.5%) were included. General characteristics were: age, 66.4±9.5 years old; Charlson index>3, 40(53.3%); COPD GOLD D, 65(86.7%); median SAPSII score, 27[22-34]; initial invasive mechanical ventilation, 20 (26.7%); median length of stay, 10[6-16] days. The follow-up was possible in 71(94.6%) patients. Mortality rate, exacerbation and readmission episodes after 3 months were respectively 7(9.3%), 23(30.7%) and 14(18.7%). Univariate analysis showed a significant association between 6MWT ratio and: 1) Mortality: p=0.037; 2) Future exacerbation: p= 0.000; 3) Readmission: p= 0.04. The ROC curves identified interesting AUC (AUC=0.74; P=0.038); (AUC=0.81; P=0.000) and (AUC = 0.7; P =0.019), respectively for mortality, future exacerbations and readmissions. The optimal cut-off point for the 6MWT ratio to predict mortality was 0.23 and to predict future exacerbations and readmissions was 0.4.

**Conclusions:** The 6MWT ratio performed at ICU discharge reveals interesting discriminative properties to predict early mortality, future exacerbations and readmissions in AE/COPD patients.

### P390 Diffuse alveolar haemorrhage in an intensive care unit - search and you will find

#### M Matias^1^, E Ribeiro^2^, J Baptista^3^, P Martins^3^

##### ^1^Centro Hospital e Universitário de Coimbra, Serviço de Pneumologia A, Coimbra, Portugal; ^2^Centro Hospitalar do Baixo Vouga, Serviço de Medicina Intensiva, Aveiro, Portugal; ^3^Centro Hospital e Universitário de Coimbra, Serviço de Medicina Intensiva, Coimbra, Portugal

**Introduction:** The aim of this study is to characterize patients with diffuse alveolar haemorrhage (DAH) in a intensive care unit (ICU).

**Methods:** Retrospective analyses of patients with DHA since 2002 to 2018 in the ICU. Clinical characteristics and outcome were determined by chart review.

**Results:** Eighteen patients were included, 13 (72.2%) males, with a mean age of 55.6 years. One third (33.3%) presented haemoptysis and 55.5% had bloody secretions before Bronchoalveolar Lavage (BAL). Only 1 patient had already the diagnosis of DAH on admission. Diagnosis was confirmed by finding a progressively haemorrhagic BAL fluid or haemosiderocytes in cytology. Identified DAH causes were drug toxicity/solid organ transplant in 22.2% of patients. vasculitis in 16.6%, cardiac disease, trauma/lung contusion, infection and ARDS/TEP in 11.1% each, drug toxicity and sarcoidosis in 5.5% each, and unknown mechanism in 11.1%. Time since admission to BAL execution was in average 6 days (0-16). shorter in patients presenting haemoptysis or bloody secretions. In 61.1% of patients, high doses of glucocorticoids were administered after diagnosis of DHA, with no difference in mortality compared to patients not receiving this therapy. Mortality in this sample was 38.8% (7 patients), with a mean age of 56.6 years, corresponding to the following DAH causes: infection in 2 patients, vasculitis, solid organ transplant/drug toxicity, cardiac disease and sarcoidosis in 1 patient each.

**Conclusions:** The diagnosis of DAH is difficult, and patients may be initially misdiagnosed as severe pneumonia. DAH should be considered in patients with persistent pulmonary infiltrates despite “adequate” treatment. Non-microbiologic analysis of BAL fluid is still useful as a diagnostic tool in ICU setting.

### P391 Dyspnea of unknown cause? Imaging the diaphragm

#### C Sepe, C Paolillo

##### Brescia Civil Hospital, Emergency Department, Brescia, Italy

**Introduction:** The incidence of diaphragmatic ruptures after thoraco-abdominal traumas is 0.8–5% [1] and up to 30% diaphragmatic hernias present late [2] when there is a complication. We report two cases of delayed traumatic diaphragm rupture to highlight the diagnostic difficulties.

**Methods:** Case 1 (image 1) presented left diaphragmatic hernia containing the stomach, spleen, bowel and pancreas. The patient reported a motor vehicle accident dating 4 months. He had thoracoabdominal trauma with several broken ribs on the left side. He then reported occasional pain in his left shoulder and occasional dyspnoea. Case 2 (image 2) showed right diaphragmatic hernia containing right hemicolon, right hepatic lobe and gallbladder, he reported occasional dyspnoea and recent right chest pain. He had a 25 years car accident in which three ribs broke on the right side.

**Results:** Almost 88% of the patients with delayed diaphragmatic rupture presented with complications between 9 and 12 months after trauma, Singh [3] reported a diaphragmatic rupture presenting 50 years after the traumatic event. The physical examination is often not helpful.

**Conclusions:** Those cases emphasizes on the delayed presentation, patients may be asymptomatic or produce only mild, nonspecific symptoms, such as vague abdominal pain, chest pain or recurrent dyspnoea for months or years. The best tool to guide the clinician toward the appropriate diagnosis is a high index of suspicion whenever there is a history of high velocity trauma, regardless of how remote.

**References**

1. Rossetti G et al. Chir Ital, 57:243-246, 2005.

2. Pappas G et al. Hernia, 11:257-259, 2007.

3. Singh S et al. J Trauma 49:156-9, 2000.

**Fig. 1 (abstract P391).**
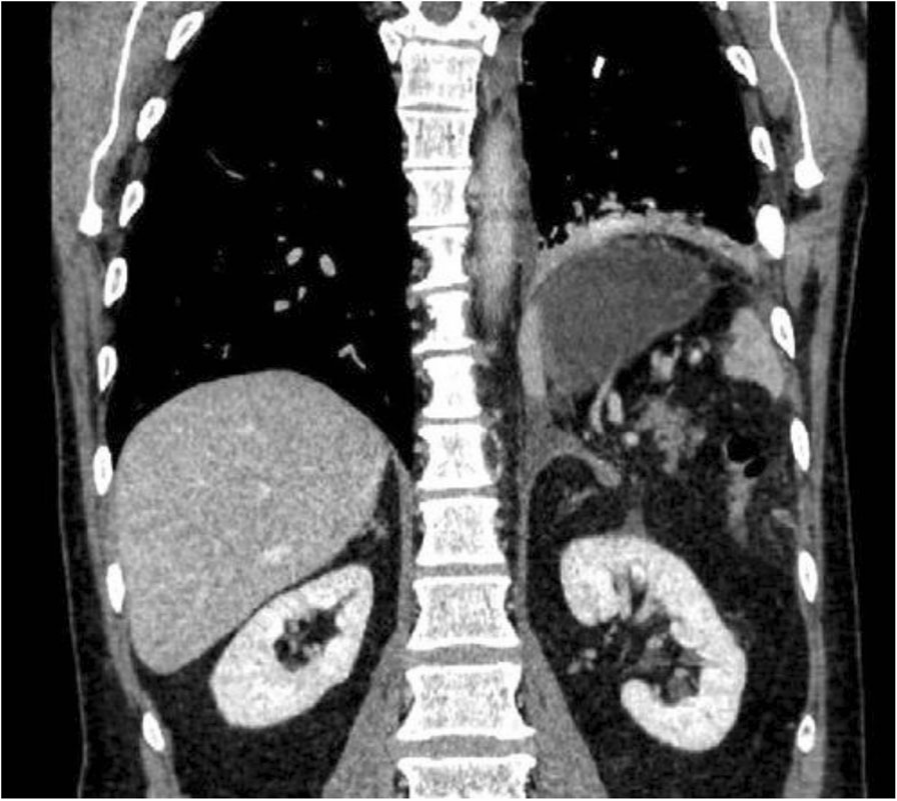
left diaphragmatic hernia containing the stomach, spleen, bowel and pancreas

**Fig. 2 (abstract P391).**
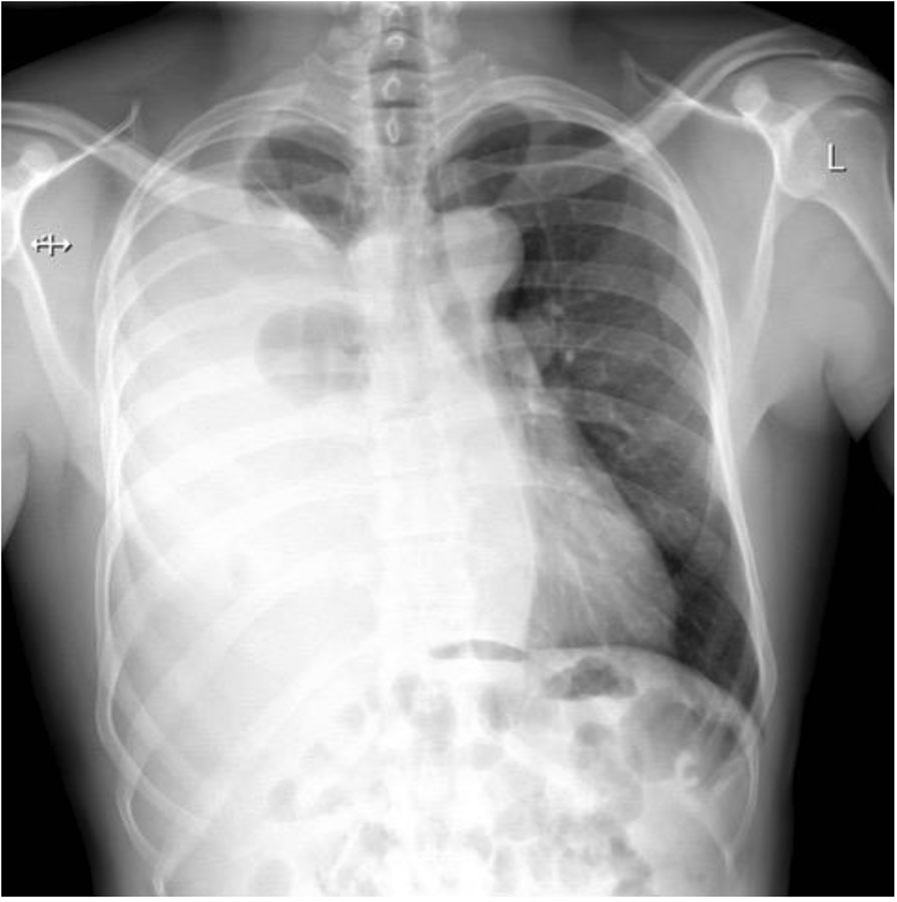
right diaphragmatic hernia containing right hemicolon, right hepatic lobe and gallbladder

### P392 Factors associated with asynchronies in pressure support ventilation (PSV), a bench study

#### E Arisi^1^, I Bianchi^1^, M Paglino^1^, A Borromini^1^, A Orlando^2^, M Pozzi^2^, GA Iotti^2^, F Mojoli^1^

##### ^1^Fondazione IRCCS Policlinico S. Matteo, Pavia, Anesthesia, Intensive Care and Pain Therapy, University of Pavia, Pavia, Italy; ^2^Fondazione IRCCS Policlinico S. Matteo, Pavia, Anesthesia and Intensive Care, Fondazione IRCCS Policlinico S. Matteo, Pavia, Pavia, Italy

**Introduction:** the aim of this study was to evaluate how different respiratory mechanics, muscle efforts, respiratory rates and pressure support levels affect patient-ventilator interaction in PSV in a simulated patient

**Methods:** We tested 5 mechanical ventilators (IMT Medical Bellavista 1000, Hamilton Medical G5 and C6, Mindray SV300, Philips V200) in PSV with the default trigger settings (Inspiratory Trigger Sensitivity (ITS) 2 l/min, Expiratory Trigger Sensitivity (ETS) 25% and Ramp 0.2ms). A IngMar Medical ASL 5000 Breathing Simulator (Software Version 3.6) was used to simulate 3 different lung mechanics: a normal patient (Compliance (C) 60 ml/cmH2O, airway resistance (R) 10 cmH20/l/s), an obstructive patient (C 90, R 20) and a restrictive patient (C 25, R 10). Each respiratory mechanic was tested at two respiratory rates (RR 15/min and 30/min), with 3 different levels of patient effort (Pmusc -3, -6 and -12cmH2O) and at two different pressure support levels (PS 10 and 20cmH2O) with a constant PEEP of 5cmH2O. All the data and waveforms patterns were recorded by the IngMar Medical ASL 5000 software and analysed with MedCalc (version 18.11 MedCalc Software, Ostend, Belgium). From the ASL raw data we obtained the Trigger Delay (TD), Delta Cycling (DC), number of Missed Efforts (ME), and number of Auto Triggers (AT). A one-way ANOVA test was used to compare these data between the 3 respiratory mechanics and the 3 muscle efforts; a paired t-test was used to compare the data between the 2 respiratory rates and the 2 pressure support levels

**Results:** all the results are shown in the Table 1 and 2 and Figure 1 and 2.

**Conclusions:** Compared to normal mechanic, the obstructive patient had more, and the restructive less, asynchronies. Lower muscle effort, higher RR and higher PS were associated with increased asynchronies

**Table 1 (abstract P392).** See text for description

	TD (sec) MEAN/ST DEV	DC (sec) MEAN/ST DEV	ME (%)	AT(%)
RESTRICTIVE	0.091*/0.043	0,049*/0.109	0*	0.11
NORMAL	0.161*/0.097	0.301*/0.295	15.36*	0.07
OBSTRUCTIVE	0.256*/0.256	0.894*/0.109	30.43*	0.18
Pmus -3	0.191*/0.122	0.525*/0.703	26.24*	5.76*
Pmus -6	0.149*/0.097	0.364*/0.543	15.98*	0
Pmus -12	0.131*/0.087	0.303*/0.303	2.3*	0

Data marked with * show a statistical significat difference (p<0.001)

**Table 2 (abstract P392).** See text for description

	TD (sec) MEAN/ST DEV	DC (sec) MEAN/ST DEV	ME (%)	AT(%)
FR 15/min	0.157/0.155	0.269/0.515	9.39	0.29
FR 30/min	0.153/0.097	0.387 $/0.562	18.55 $	0.02
PS 10 cmH2O	0.142/0.094	0.239/0.426	11.53	0.17
PS 20 cmH2O	0.169$/0.114	0.452$/0.638	19 $	0.07

Data marked with $ show a statistical significat difference (p<0.0001)

**Fig. 1 (abstract P392).**
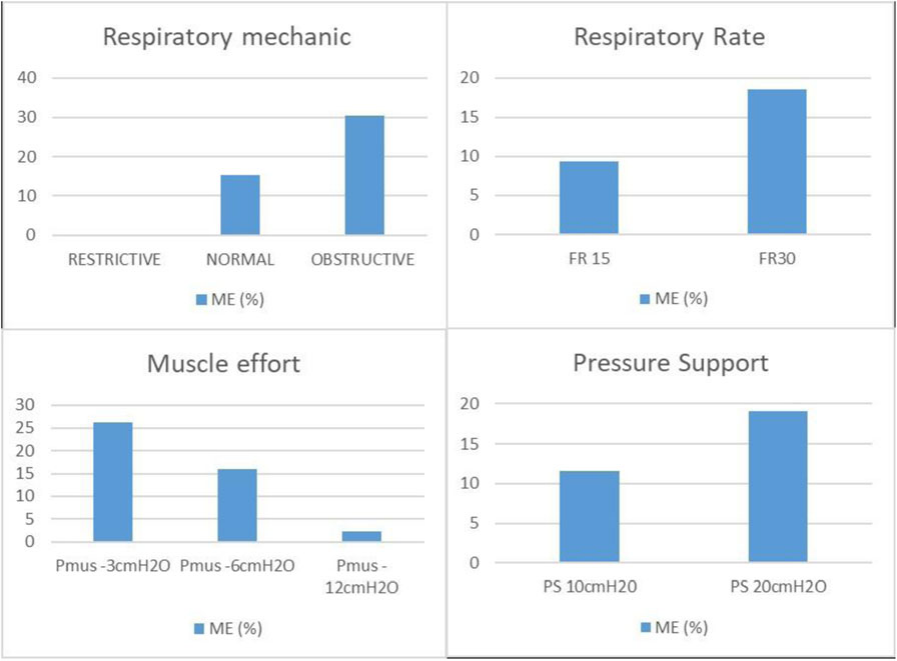
Missed inspiratory/expiratory efforts

**Fig. 2 (abstract P392).**
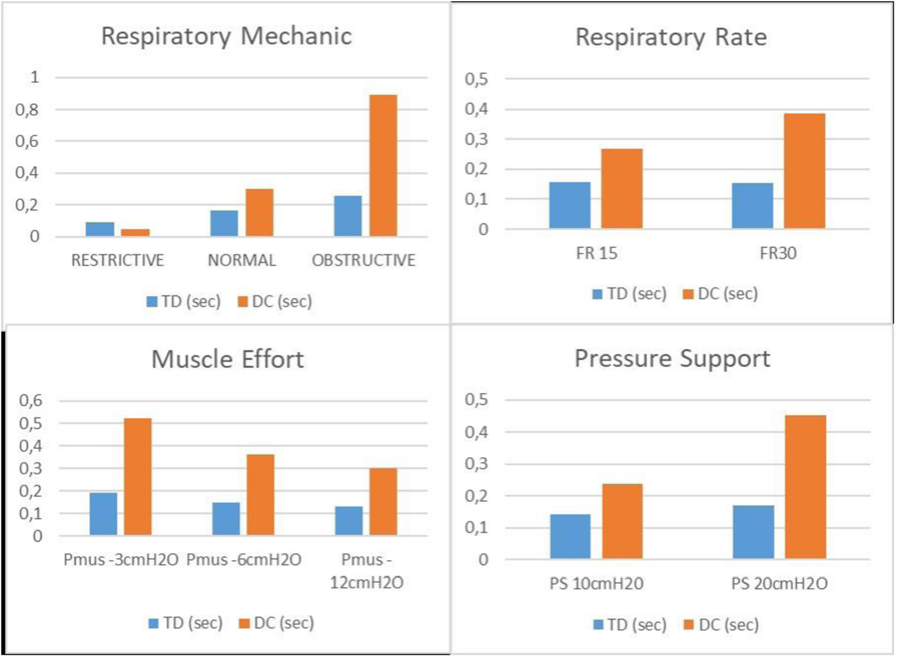
Inspiratory and expiratory delay

### P393 Effects of positive end expiratory pressure level on left and right ventricular strain

#### M Dulgeroglu^1^, A Camkiran Firat^2^, B Pirat^3^, A Sezgin^4^, A Pirat^5^

##### ^1^Guven Hospital, Intensive care, Ankara, Turkey; ^2^Baskent University Hospital, Anesthesiology, Ankara, Turkey; ^3^Baskent University Hospital, Cardiology, Ankara, Turkey; ^4^Baskent University Hospital, Cardiovascular Surgery, Ankara, Turkey; ^5^Baskent University Hospital, Intensive Care, Ankara, Turkey

**Introduction:** Patients who admitted to intensive care with respiratory failure often require positive end expiratory pressure(PEEP) in order to correct hypoxemia. However, PEEP might have a negative effect on cardiovascular hemodynamics, it is still controversial. The aim of this study was to evaluate the effects of different PEEP levels on both left and right ventricular strains by using speckle tracking imaging in patients undergoing coronary artery bypass grafting surgery (CABG) [1].

**Methods:** We prospectively analyzed 10 CABG surgery patients. After initiation of mechanical ventilation and before sternotomy 5, 10 and 20 cmH2O of PEEP were applied in 5 minutes intervals consequently. After stabilization at each PEEP level four and two chamber images of left and right ventricle were recorded using transesophageal echocardiography (TEE). SPSS 17 software was used for the statistical analysis of the data.

**Results:** The mean age of study patient (85% male) was 59.7 ± 10.5 years. Intraoperative mean, systolic and diastolic arterial blood pressures and heart rate were similar at the three PEEP levels. Mean right ventricle strain value significantly decreased at 20 cmH2O PEEP when compared with other two PEEP levels (p=0.005 for both) (Table 1). Mean SR values decreased at 20 cmH2O PEEP when compared with 5 and 10 cmH2O PEEP (p<0.05). Right ventricle velocity reduced with incremental PEEP increases (p<0.05). Conversely left ventricle strain, SR and velocity did not change significantly.

**Conclusions:** Compared with 5 and 10 cmH2O PEEP levels, right ventricle functions in terms of strain, SR, RVFAC were significantly impaired at 20 cmH2O PEEP level. However we didn’t find any significant difference on left ventricule function for all the three PEEP levels.

**Reference**

1. Franchi F et al. Biomed Research Int.2013;918548.

**Table 1 (abstract P393).** LV- RV function parameters of the patients for each PEEP level (Mean ± standard deviation)

	5 cmH2O PEEP	5 cmH2O PEEP	10 cmH2O PEEP	10 cmH2O PEEP	20 cmH2O PEEP	20 cmH2O PEEP
	LV	RV	LV	RV	LV	RV
Strain (%)	18.1 ± 3.7	24.0 ± 2.3	20.7 ± 4.0	24.0 ± 2.1	20.4 ± 5.6	18.6 ± 2.7^a^
Strain rate (1/minute)	1.0 ± 0.2	1.3 ± 0.2	1.2 ± 0.4	1.2 ± 0.2	1.1 ± 0.3	1.0 ± 0.4^a^
Velocity (cm/minute)	4.7 ± 1.5	8.1 ± 1.4	6.4 ± 2.6	7.5 ± 1.6	5.1 ± 1.5	6.1 ± 1.5^a^

^a^ Compared measurements with other two PEEP level of p<0.05. PEEP, Positive end expiratory pressure; LV, left ventricule; RV, right ventricule

### P394 Waveform-aided triggering a bench study on 5 mechanical ventilators

#### E Arisi^1^, A Orlando^2^, A Borromini^1^, M Paglino^1^, I Bianchi^1^, M Pozzi^2^, G Iotti^2^, F Mojoli^1^

##### ^1^Fondazione IRCCS Policlinico S. Matteo, Pavia, Anesthesia, Intensive Care and Pain Therapy, University of Pavia, Pavia, Italy; ^2^Fondazione IRCCS Policlinico S. Matteo, Pavia, Anesthesia and Intensive Care, Fondazione IRCCS Policlinico S. Matteo, Pavia, Pavia, Italy

**Introduction:** The aim of this study was to evaluate how different types of automated waveform-aided triggering systems affect the asynchronies in a bench setting

**Methods:** We tested 5 mechanical ventilators: IMT Medical Bellavista 1000, Hamilton Medical G5 and C6 (Fig 1), Mindray SV300, Philips V200. A IngMar Medical ASL 5000 Breathing Simulator (Software Version 3.6) was used to simulate 3 different lung mechanics: a normal patient (Compliance (C) 60 ml/cmH2O, airway resistance (R) 10 cmH20/l/s), an obstructive patient (C 90, R 20) and a restrictive patient (C 25, R 10). Each respiratory mechanic was tested at two respiratory rates (RR 15/min and 30/min), with 3 different levels of patient effort (Pmusc -3, -6 and -12cmH2O) and at two different pressure support levels (PS 10 and 20cmH2O) with a constant PEEP of 5cmH2O. All the ventilators were tested in PSV with the default trigger settings (Inspiratory Trigger Sensitivity (ITS) 2 l/min, Expiratory Trigger Sensitivity (ETS) 25% and Ramp 0.2ms) and then with all the automatic options enabled. All the data and waveforms patterns were recorded by the IngMar Medical ASL 5000 software and analysed with MedCalc (version 18.11 MedCalc Software, Ostend, Belgium). From the ASL raw data we obtained the Trigger Delay (TD), Delta Cycling (DC), number of Missed Efforts (ME), and number of Auto Triggers (AT). A paired t-test was used to compare the results between the Standard Trigger and the Automatic Trigger on each ventilator

**Results:** Data showing the overall performance of each ventilator in the whole bench test are shown in the Table. TD and DC are expressed as mean ± ST. dev

**Conclusions:** Compared to standard triggers, waveform-aided triggering systems show a better performance in almost all the tested ventilators, with a significant reduction of TD, DC and ME

**Table 1 (abstract P394).**
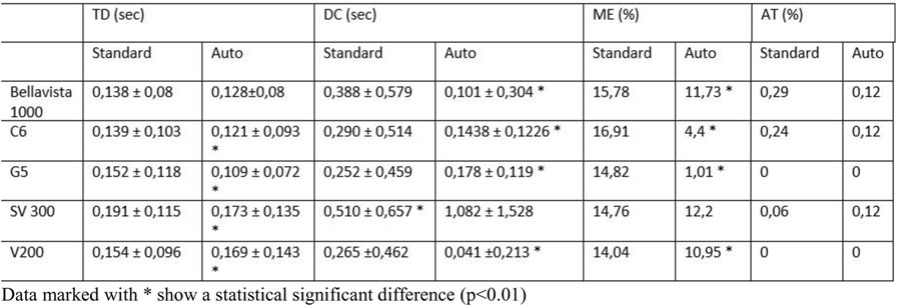
Performance of each ventilator

Data marked with * show a statistical significant difference (p<0.01)

**Fig. 1 (abstract P394).**
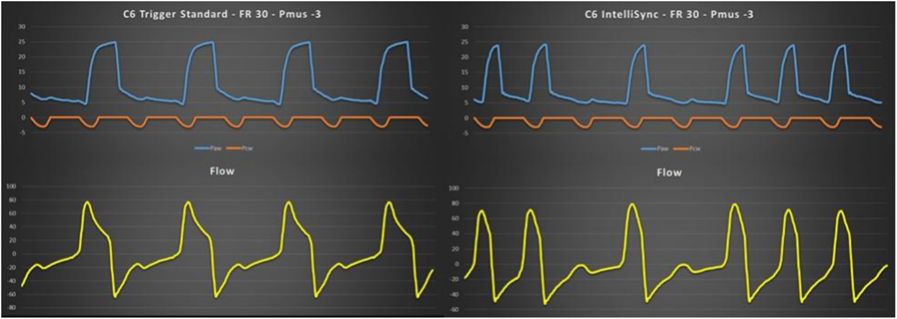
Example of waveform-aided triggering sistem on the Hamilton C6

### P395 Incidence and risk factors associated with ocular surface disorders in ventilated ICU patients and impact of protocolised eye care

#### V Bhagat^1^, JV Divatia^1^, MS Khan^1^, NR Prabu^1^, AP Kulkarni^1^, S Myatra^2^

##### ^1^Tata Memorial HOSPITAL, Department of Anaesthesia, Critical Care and Pain, Mumbai, India; ^2^Tata Memorial HOSPITAL, Department of Anaesthesia, Critical Care and Pain, Mumbai, India

**Introduction:** Critically ill patients frequently have increased risk of ocular surface disorders (OSDs) due to poor eyelid closure and reduced tear production due to sedation during mechanical ventilation. We conducted a study to look at the incidence of OSDs in our ICU with the current eye care practices and the impact of a protocolised eye care on the incidence and outcome and to determine the correlation of risk factors with the incidence of OSDs

**Methods:** This study was done in our mixed medical surgical ICU. It had a prospective cohort design and was done as before and after study in two phases (Phase I and Phase II). In phase I existing eye care practices were continued. In phase II protocolised eye care was implemented and incidence of OSDs was noted in both phases. Patients mechanically ventilated for > 24 hours were included. Study completion criteria was development of OSD, discharge from ICU or death. In both phases an Ophthalmologist (study investigator) examined the eyes every 72 hours using a hand-held slit-lamp. An independent ophthalmologist re-assessed the eye findings by the study investigator to check for bias. At the end of study inter-observer agreement was calculated

**Results:** The study included 125 (phase I: 62/phase II: 63). There was no difference in demographic variables Incidence of OSD was higher in phase I with 64.5% of patients, while in phase II it was 34.9% (p=0.001). Inter-observer agreement among the investigators was good in both the phases (Kappa index in phase I 0.927, in phase II 0.908) On univariate and multivariate analysis, increased depth of sedation (p = 0.0001) and absence of protocolised eye care (p=0.001) were associated with higher incidence of OSDs.

**Conclusions:** Incidence of Ocular surface disorders (OSDs) is high in sedated ventilated patients in ICU. Protocolised eye care helps in decreasing the incidence of OSDs. Increased depth of sedation and absence of protocolised eye care are independent risk factors associated with OSDs in ICU.

### P396 Cochrane review: BIS monitoring versus clinical assessment for sedation in mechanically ventilated adults in the intensive care unit and its impact on outcomes and resource utilization

#### R Shetty

##### Manipal Hospital Whitefield, Department of Critical Care Medicine, Bengaluru, India

**Introduction:** Optimizing sedation practice in intensive care unit (ICU) may reduce mortality, improve patient comfort and reduce cost. Current practice is to use scales to assess depth of sedation based on clinical criteria (CA). Bispectral index (BIS) monitors may overcome the constraints of the sedation scales (e.g. subjectivity). Our aim was to find out if BIS monitoring improves outcomes and resource utilization in ICU mechanically ventilated adults.

**Methods:** We searched CENTRAL, MEDLINE, Embase, CINAHL and several other databases. We selected only randomized controlled trials. We used Cochrane’s standard methodological procedures and Revman 5.3 software for data collection and analysis.

**Results:** We identified 4245 studies. Four studies (256 participants) met the inclusion criteria. BIS monitor was used in all the studies. Sedation assessment tools for CA included the Sedation-Agitation Scale (SAS), Ramsay Sedation Scale (RSS), or subjective CA utilizing traditional clinical signs. Three of the four studies were classified as high risk. There was no difference in ICU length of stay (median 8, interquartile range (IQR) 4 to 14 versus 12 (6 to 18); N=50; low quality evidence). There was clinically non-significant effect on the duration of mechanical ventilation (MD -0.02 days (95% CI -0.13 to 0.09; 2 studies; N= 155; I2 = 0%; low quality evidence)). We could not measure difference in the cost because of different sedation protocols and agents used. Clinically relevant adverse events (e.g. self-extubation) were not reported in any study. GRADE quality of evidence was very low.

**Conclusions:** In this Cochrane review we found insufficient evidence of benefit of BIS compared to clinical assessment scales for sedation monitoring in critically ill mechanically ventilated adults.

**References**

1. Shetty RM et al. Cochrane Database of Systematic reviews 2018, Issue 2. Art. No. CD011240. DOI: 10.1002/14651858.CD011240.pub2.

### P397 Is intranasal fentanyl so effective than parenteral morphine for managing acute post traumatic pain in adults?

#### N Nouira, S Lahmer, S Jaouani, W Demni, M Arafa, M Boussen, E Ben Othmane, M Ben Cheikh

##### Mongi Slim Academic Hospital, Emergency Department, Tunis, Tunisia

**Introduction:** Both fentanyl and morphine are known as opioid analgesics, which blocks the brain from receiving pain signals, the route of administration and the adverse effects affect their use. We compare the efficacy of intranasal fentanyl versus intravenous morphine adults population presenting to an emergency department (ED) with acute post traumatic severe pain.

**Methods:** We conducted a prospective, randomized, double-blind, placebo-controlled, clinical trial in a tertiary Emergency department between october 2016 and June 2017. Adults with severe post traumatic was included to receive either active intravenous morphine (3 mg immediately and then 1 mg every 3 min if persistence of severe pain maximum 10 mg) and intranasal placebo or active intranasal concentrated fentanyl (2 μ g /kg maximum 200 μ g) and intravenous placebo. Exclusion criteria: significant head injury, allergy to opiates, nasal blockage, or inability to perform pain scoring, Pain scores were rated by using a digital scale at 0, 5, 10, and 30 minutes. Routine clinical observations and adverse events were recorded.

**Results:** Were enrolled 475 patients; mean age was 37 years [SD 15], 231 patients received intravenous (i.v) morphine and 243 received intranasal fentanyl. Statistically significant differences in digital scale scores were not observed between the 2 treatment arms either preanalgesia and at 5 minutes postanalgesia. At 10 and 30 minutes the difference in mean digital scale between the morphine and fentanyl groups (with non parametric Mann -Whitney test) was statistically significant (p<0.001). There were no serious adverse events in fentanyl group.

**Conclusions:** Intranasal fentanyl was shown to be an effective analgesic in adults patients presenting to an ED with an acute postraumatic pain when compared to intravenous morphine.

### P398 Ketamine-related sclerosing cholangitis in severe burns

#### M Raineau, N Donat, C Hoffmann, A Cirodde, Jv Schaal, Y Masson, T Leclerc

##### Hopital d´instruction des armées de Percy, Centre de traitement des brûlés, Clamart, France

**Introduction:** Hepatic disorders have increased in our burn intensive care unit (BICU), resulting in cases of “ischemic sclerosing cholangitis” (ISC) with four liver transplants from 2013 on. Concomitantly, ketamine (K) use, considered innocuous and with a broad safety range, increased as co-analgesic, then as hypnotic when concern about ischemia increased. Based on scarce literature reporting K abuse-related ISCs [1], we finally restricted K use. We hypothesized that hepatic disorders were related to K use in our BICU.

**Methods:** All patients admitted to our BICU were reviewed in a retrospective study from 01/01/07 to 30/06/18 with demographic data, burn/injury severity scores, and hepatic biochemistry. Annual mean K consumption per patient was used as a population exposure inde. Three periods were compared: P1 (2007-2013) as historical period, P2 (2013-2017) from 1st ISC case on, and P3 ( 2017-2018) with implemented K restriction policy. Comparisons used ANOVA, Kruskal-Wallis or Wald tests as approriate.

**Results:** 1640 patients were included. Patients were younger but more severe in P1 (tab.1). K use in P2 was higher than in P1-P3 (p<0.01). Hepatic disorders significantly differed between periods (Table 1, Fig.1), and matched K use. No ISC and limited hepatic disorders occurred in P3.

**Conclusions:** ISCs were related to K over-use in our BICU. Burnt patients are at risk of hepatic injury [2], but K related hepatic injury likely occurred. Its not clearly understood mechanisms may involve a cumulative dose effect. Although involvement of concomitant medications is being investigated, K restriction policy seemed to contain hepatic disorders.

**References**

1. Wong et al. Clin Gastroenterol Hepatol 2014, 12:1759-62.e1.

2. Jeschke MG. In: Total Burn Care, 2017.

**Table 1 (abstract P398).** Population characteristics

	P1 n = 1072	P2 n = 421	P3 n = 147	p
Age (y)	42.8 ± 20.3	47.4 ± 20.6	45.6 ± 20.4	< 0.01
Body-Surface Area Burn (%)	17 [10-32]	13 [7-27]	13 [8-23,5]	< 0.01
Inhalation (n, %)	264 (25)	87 (21)	28 (19)	0.046
Mortality (n, %)	164 (15)	66 (16)	17 (12)	0.42
Length of stay (d)	24 [9-40]	24 [11-43]	23 [15-44]	0.11
IGSII	32.4 ± 20	34.2 ± 20	39.5 ± 18.2	< 0.01
Ketamine (mg/patient)	14930 [10410-17280]	32830 [27260-36990]	9587 [1787-9587]	< 0.01

**Table 2 (abstract P398).** Hepatic disorders

	P1 n = 1072	P2 n = 421	P3 n = 147	p
GGT > 800 (n, %)	54 (5)	71 (17)	5 (3)	< 0.01
PAL > 800 (n, %)	33 (3)	56 (13)	4 (3)	< 0.01
BiliT > 80 (n, %)	48 (4)	36 (8)	4 (3)	0.33

**Fig. 1 (abstract P398).**
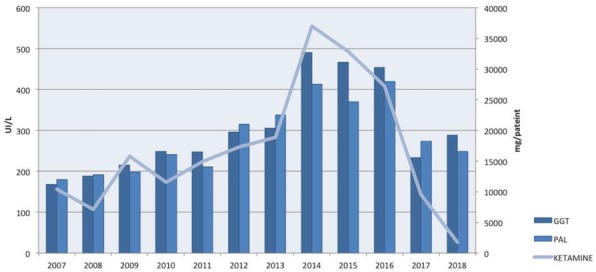
GGT and PAL modifications and ketamine consumption

### P399 Persistent post-intensive care opioid use in US veterans after major abdominal surgery

#### K Karamchandani^1^, S Pyati^2^, W Bryan^3^, M Pepin^4^, E Lehman^5^, K Raghunathan^2^

##### ^1^Penn State Health Milton S. Hershey Medical Center, Anesthesiology and Perioperative Medicine, Hershey, United States; ^2^Duke University Health System, Department of Anesthesiology, Durham, United States; ^3^Durham VA Healthcare System, Patient Safety Center for Inquiry, Durham, United States; ^4^Durham VA Healthcare System, Patient Safety Center of Inquiry, Durham, United States; ^5^Penn State Health Milton S. Hershey Medical Center, Public Health Sciences, Hershey, United States

**Introduction:** Opioid infusions are routinely administered in intensive care units (ICUs) and survivors are often discharged on opioid therapy. These patients are at a risk for chronic opioid use, addiction or abuse, overdose and opioid use disorder. Patients undergoing major abdominal surgeries often require postoperative ICU care with pain management including the use of opioid infusions. The objective of this study was to estimate the incidence of new persistent opioid use in opioid naïve patients that underwent major abdominal surgery, requiring postoperative ICU care and receiving opioid infusions, across the US Veterans Health Administration over a 17-year period.

**Methods:** Between 2000 and 2016, we identified a cohort of patients without prescriptions for opioid containing medications over the six-month period prior to admission (preoperatively opioid naïve) that then underwent major abdominal surgery. We identified patients admitted to ICU for >24 hours that also received opioid infusions. New persistent opioid use was defined as the continuation of prescriptions for opioid containing medications beyond three months after discharge.

**Results:** Of 34,442 patients that underwent major abdominal surgery nationwide within the VHA, between 2000-2016, 13,867 were opioid naïve with an ICU stay for > 24 hrs after surgery. Of these, 74.7% (n=10,367) received opioid infusions during their stay. New persistent opioid use was present in 2.26% (95% CI, 1.9-2.5), and steadily declined annually (Figure 1).

**Conclusions:** Among preoperatively opioid naïve US veterans undergoing major abdominal surgery with postoperative ICU admission, a majority received opioid infusions. Of these under 2.5% developed new persistent opioid use. Patient factors, ICU pain management strategies, and primary care practices where opioid prescriptions may be continued after initial postoperative recovery, may combine to yield the observed rates of new Persistent Post-intensive care opioid use after major abdominal surgery.

**Fig. 1 (abstract P399).**
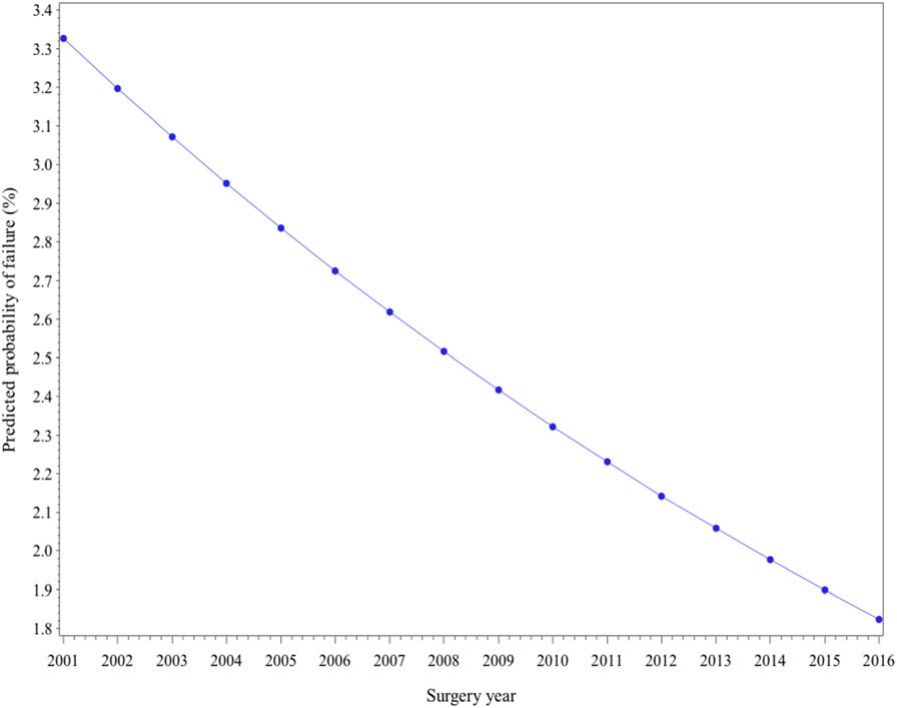
New persistent opioid use amongst US veterans requiring postoperative ICU care after undergoing major abdominal surgery and receiving opioid infusion

### P400 Outcomes and use of adjunct agents in patients receiving extracorporeal membrane oxygenation (ECMO) sedated with alfentanil vs. fentanyl: a single centre retrospective analysis

#### M Barker^1^, A Dixon^2^, L Camporota^3^, N Barrett^2^, R Wan^1^

##### ^1^Guys and St Thomas´ NHS Foundation Trust, Pharmacy/Critical Care, London, United Kingdom; ^2^Guys and St Thomas´ NHS Foundation Trust, Critical Care, London, United Kingdom; ^3^Guys and St Thomas´ NHS Foundation Trust, London, United Kingdom

**Introduction:** In November 2016, our institution switched from using alfentanil to fentanyl for analgesia and sedation in adult patients receiving ECMO. There is no published evidence comparing the clinical use of alfentanil vs fentanyl for sedation in ECMO patients, although some reported increased fentanyl sequestration into the circuit [1]. For these reasons, we conducted a retrospective observational study to explore whether there were any significant differences in patient outcome or adjunctive sedation before and after the switch.

**Methods:** Outcome data and total daily doses of alfentanil or fentanyl as well as adjunctive sedation/analgesia for each patient where obtained from our clinical information system (Philips ICCA®). Data was included from ECMO patients who were sedated with alfentanil or fentanyl from 1/1/16 to 31/10/2017 until ECMO decannulation. Patients not requiring either opiate or who were switched between the two during ECMO therapy were excluded. All medicines prescribed for the management of sedation or agitation were included. For each patient an average total daily dose of each drug, was calculated. Data was analysed using STATA®.

**Results:** Both groups were found to be statistically equivalent for mode of ECMO, age, APACHE 2 score and charlson score (p=0.202) except for BMI (p=0.007). No difference in patient outcomes were found between groups (Table 1). Patients in the alfentanil group were found to have received significantly higher median average total daily dose of quetiapine and midazolam (Table 2).

**Conclusions:** No differences in patient outcomes were found between patients sedated with alfentanil compared to fentanyl. We found a statistically significant reduction in adjunctive sedation and antipsychotic drugs in patients treated with a fentanyl-based regimen. Prospective studies will be required to confirm these results and their association with delirium and post-ICU cognitive outcomes.

**Reference**

1. Shekar K et al. Crit Care 16:R194, 2012

**Table 1 (abstract P400).** Patient outcomes

Outcome	Alfentanil-based sedation	Fentanyl-based sedation	P Value (Chi-squared test/2 sided t test)
Number of patients alive at ICU Discharge	55	72	0.553
Number of Patients Alive at Hospital Discharge	52	61	0.927
Average number of days on ECMO (95%CI)	13.46 (10.70, 16.23)	11.88 (9.57, 14.19)	0.382
Average length of Critical Care Stay, days (95%CI)	23.54 (19.78, 27.29)	22.43 (18.94, 25.91)	0.671

**Table 2 (abstract P400).** Use of adjunct agents - median of average total daily dose

Adjunct	Alfentanil-based sedation	Fentanyl-based sedation	P Value (Mann-Whitney)
Clonidine (mcg)	231.55	141.67	0.820
Dexmedetomidine infusion (mcg/kg/hr)	0.87	0.90	0.872
Lorazepam (mg)	3.79	2.5	0.324
Midazolam infusion (mg/kg/hr)	0.05	0.02	0.009
Oxycodone (mg)	38.46	16.67	0.056
Propofol infusion (mg/kg/hr)	1.06	1.00	0.509
Quetiapine (mg)	111.46	51.57	0.016

### P401 Is it safe to use a 12 hour cut off to exclude the effect of fentanyl on clinical examination in ICU patients treated with targeted temperature management (TTM) post-cardiac arrest?

#### F Baldwin^1^, O Boyd^2^, R Gray^2^, L Ortiz- Ruiz De Gordoa^2^, M Allen^3^, G Scutt^4^, B Patel^4^

##### ^1^Brighton and Sussex University Hospitals NHS Trust, Intensive Care, Brighton, United Kingdom; ^2^Brighton and Sussex University Hospitals NHS Trust, Intensive Care Unit, Brighton, United Kingdom; ^3^University of Brighton, School of Pharmacy and Biomolecular Sciences, Brighton, United Kingdom; ^4^University of Brighton, School of Pharmacy and Biomedical Sciences, Brighton, United Kingdom

**Introduction:** The European Society of Intensive Care Medicine Consensus Statement recommends that for comatose survivors of cardiac arrest 12 hours without sedation is the minimum acceptable before neurological assessment. They highlighted the need to investigate the pharmacokinetics of opioid drugs in post-cardiac arrest patients, especially those treated with controlled temperature [1].

**Methods:** Following approval by Research Ethics Committee, we measured the blood concentration of fentanyl in 24 post-cardiac arrest patients treated with TTM following cessation of continuous infusion. The fentanyl was discontinued when the patients were rewarmed to a temperature of 36.5 degrees Celsius and a blood sample taken 12 hours later. The blood was analysed using a commercial ELISA kit (Neogen Corporation). Using the total dose of fentanyl administered, the half-life of fentanyl was calculated for each patient. Patient physiological data, CYP3A4 and ABCB1 polymorphism and drug history were compared with half-life.

**Results:** The median fentanyl concentration at 12 hours was 0.82 mcg/L with a very wide range (0.07- 8.29 mcg/L). The results for calculated half lives are shown in Figure 1. There was no correlation between fentanyl level and BMI, illness severity (SAPS ll), creatinine clearance, transaminase or lactate level. There was no correlation between co-administration of drugs of metabolised by the CYP3A4 and ABCB1 enzyme systems or genotype.

**Conclusions:** There is marked variation in the concentration of fentanyl at 12 hours in patients managed with TTM following cessation of fentanyl infusion. The calculated clearance of fentanyl in some patients is greater than 48 hours and a 12 hour cut off is not safe.

**Reference**

1. Sandroni C et al. Intensive Care Med (2014) 40:1816–18314.

**Fig. 1 (abstract P401).**
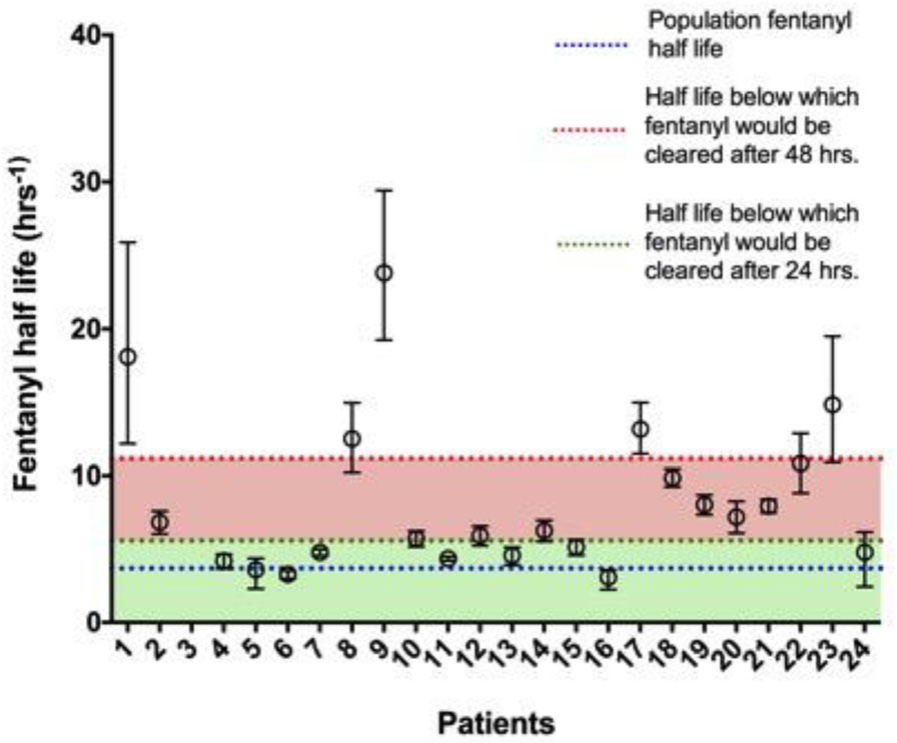
Calculated half life of fentanyl in patients following TTM

### P402 Multimodal neural block analgesia versus morphine analgesia after elective knee surgery

#### D Loncar stojiljkovic^1^, M Stojiljkovi^2^

##### ^1^SGH Jevremova, Anesthesia and Intensive Care, 11000, Serbia; ^2^Faculty of Medicin, University of Banja Luka, Department of Pharmacology, Toxicology and Clinical Pgarmacology, Banja Luka, Bosnia and Herzegovina

**Introduction:** Objective of this study was to compare the effects of three analgesic regimens, one opioid and two multimodal ones, on cardiovascular stability and pain intensity in patients undergoing elective surgery under general endotracheal anesthesia during the 24 h postoperative period.

**Methods:** Sixty elderly patients, ASA II, undergoing elective knee sugary were assigned to receive 1) morphine 5 or 10 mg iv q6h, depending on body weight, and paracetamol 1 g iv q6h (MP group), or multimodal nerve block: 2) femoral nerve block, single shot (FNB group) or 3) fascia iliaca compartment nerve block single shot (FICNB group). Measurement of pain intensity was performed with numerical pain scale (NPS). Systolic blood pressure and consumption of additional analgesics on demand were monitored, as well as the duration of nerve blocks.

**Results:** Both nerve blocks produced significantly lower pain scores than the purely pharmacological approach (MP 5.4+0.6 vs. FNB 2.8+1.6 and FICNB 2.9+1.2 after first postoperative hour). Consequently, the first group required more additional morphine and paracetamol after first hour compared to FNB and FICNB groups. Morphine was significantly more frequently added in the MP group (3.4+0.4 mg/kg iv) than in the FNB (2.1+0.5 mg/kg iv) and FICNB (2.5+0.4 mg/kg iv). Maximal duration of analgesia was 4.9+0.5 h in MP group compared to 8.8+0.7 h and 8.1+0.8 h after FNB and FICNB, respectively.

**Conclusions:** Postoperative analgesia with blocks enables better pain control, better cardiovascular stability and less adverse effect than the classical morphine-based analgesia.

### P403 Symptomatology of opioid-associated withdrawal syndrome in critically ill adults: a descriptive study

#### K Zerrouki^1^, Q Li^1^, L Delucilla^1^, J Cabot^1^, S Khadadah^2^, M Duceppe^1^, M Delisle^1^, L Burry^3^, D Jayaraman^2^, C Gélinas^4^, L Tourian^5^, M Perreault^1^

##### ^1^McGill University Health Centre, Department of Pharmacy, Montreal, Canada; ^2^McGill University Health Centre, Department of Critical Care, Montreal, Canada; ^3^Mount Sinai Hospital, Department of Pharmacy and Medicine, Toronto, Canada; ^4^McGill University, Ingram School of Nursing, Montreal, Canada; ^5^McGill University Health Centre, Department of Psychiatry, Montreal, Canada

**Introduction:** Opioids are frequently used in the intensive care unit (ICU) to relieve pain and facilitate tolerance of life-support technologies. When discontinued abruptly, patients may develop a cluster of symptoms known as opioid-associated iatrogenic withdrawal syndrome (OIWS). This phenomenon is poorly described in critically ill adults although it is associated with unfavourable outcomes, such as prolonged ICU stay. The objective of this study was to describe the signs and symptoms of OIWS in adult ICU patients.

**Methods:** A prospective observational study was conducted in two tertiary care centres in patients requiring mechanical ventilation and regular opioids for more than 72 hours. After an opioid dose reduction of at least 10%, patients were assessed daily for signs and symptoms of withdrawal using a standardized form. Concomitantly, the presence of OIWS was assessed daily by a physician using modified DSM-5 criteria. All physician evaluations were blinded and performed independently. Inter-rater reliability for DSM-5 evaluations was assessed with the kappa coefficient.

**Results:** A total of 1128 patients were screened and twenty-nine enrolled. The majority were male (72.4%) with a median age of 65. The median APACHE II score was 27. Withdrawal occurred in 20.7% of patient within a median of three days (IQR 3 to 9 days) from opioid weaning. According to investigator assessment, restlessness, agitation, anxiety, hallucinations, insomnia/sleep disturbance, mydriasis and elevated blood pressure were more prevalent in OIWS-positive patients. DSM-5 evaluations identified dysphoric mood, muscle aches, lacrimation/rhinorrhea, pupillary dilation/piloerection/sweating, diarrhea and yawning more frequently in OIWS-positive patients. The kappa coefficient showed good agreement (0.64).

**Conclusions:** OIWS in critically ill adults presents with a large spectrum of signs and symptoms that occur within a median of three days from onset of opioid weaning. Further studies are needed to confirm these preliminary findings.

### P404 Withdrawal reactions after discontinuation or rate reduction of fentanyl infusion in ventilated critically ill adults

#### S Taesotikul^1^, V Tangsujaritvijit^2^, A Trisataya^2^, C Suthisisang^3^, P Dilokpattanamongkol^4^

##### ^1^Department of Pharmaceutical Care, Faculty of Pharmacy, Chiang Mai University, Chiang Mai, Thailand; ^2^Department of Medicine, Faculty of Medicine Ramathibodi Hospital, Mahidol University, Bangkok, Thailand; ^3^Department of Pharmacology, Faculty of Pharmacy, Mahidol University, Bangkok, Thailand; ^4^Department of Pharmacy, Faculty of Pharmacy, Mahidol University, Bangkok, Thailand, Bangkok, Thailand

**Introduction:** Protocol and dosing strategies of opioid therapy in intensive care units (ICUs) have been proposed in order to optimize therapeutic effect and avoid adverse drug reactions. But, there is a gap of evidence regarding opioid withdrawal in critically ill adults [1]. The objectives of this study are to explore an incidence, risk associations and clinical impact of opioid withdrawal reactions.

**Methods:** A prospective observational study was conducted in 5 ICUs and an intermediate care unit, university hospital, Thailand. Mechanically ventilated adults receiving continuous infusion of fentanyl for ≥ 24 hours were eligible. Observation for withdrawal reaction using signs and symptoms according to the Diagnostic and Statistical Manual of Mental Disorder, 5th edition (DSM-V) criteria was performed at baseline, 1st, 3rd, 6th, 24th and 72nd hours after discontinuation or rate reduction of fentanyl. Patients who manifested at least 3 signs and/or symptoms were classified as withdrawal group. Change of heart rate and mean arterial pressure (MAP) were also hourly recorded during observational period.

**Results:** During 6 months of study period, 39 patients with 71 observations were included. Eight participants (20.5%) developed withdrawal symptoms. Body mass index (BMI), weaning rate of fentanyl infusion and increment of MAP were positively correlated with withdrawal symptoms. Furthermore, two patients in non-withdrawal group suddenly developed hypertensive urgency after interruption of fentanyl infusion. However, there was no significant difference between withdrawal group and non-withdrawal group in ventilator days and ICU length of stay.

**Conclusions:** Health care providers should observe withdrawal symptoms in mechanically ventilated adults receiving fentanyl infusion for ≥ 24 hours, especially patients with high BMI and receiving rapid weaning rate. Additionally, alteration of MAP should be further investigated as signs of opioid withdrawal.

**Reference**

1. Delvin JW et al. Crit Care Med. 36:e825-73, 2018

### P405 Intriguing urine discolorations after propofol infusion observed in an Intensive Care Unit

#### A Tsifi^1^, S Stefanova^1^, S Avgeri^1^, E Sergi^1^, A Sakkalis^1^, K Ntorlis^1^, A Voulgaridis^1^, M Katsiari^1^, M Laskou^1^, A Kounougeri^1^, C Nikolaou^1^, D Theodoridis^2^, A Alexaki^1^, Z Teneketzi^1^, C Mathas^1^

##### ^1^Konstantopouleio General Hospital of Nea Ionia- Patision, Intensive Care Unit, Athens, Greece; ^2^Konstantopouleio General Hospital of Nea Ionia- Patision, Hematology Department, Athens, Greece

**Introduction:** Propofol is a well-known sedative, commonly used in Intensive Care Units (ICU s), that on rare occasions has been reported to cause green urine and has also been associated with pink or transient white urine discoloration. It can cause several adverse effects, such as low blood pressure, pain on injection, apnea, hypertriglyceridemia and when administered in high doses it may lead to the “propofol infusion syndrome”.

**Methods:** We present two examples of interesting urine discolorations observed unexpectedly in our ICU in patients under propofol sedation requiring mechanical ventilation.

**Results:** Dark green urine discoloration as presented in Fig.1 is the result of a phenolic metabolite of propofol that is produced in the liver and is subsequently excreted in the urine, thus changing its color. It is considered a reversible phenomenon that resolves after propofol discontinuation.Respectively, pink urine discoloration as presented in Fig.2 can also be the result of propofol infusion. The increase in urine excretion of uric acid caused by propofol, in combination with a low urinary PH can lead to the formation of uric acid crystals and turn the urine pink. Discontinuation of propofol and urine alkalization can reverse the phenomenon.

**Conclusions:** Green or pink urine discoloration due to propofol is generally a benign, reversible condition. Its presence should not compel the physician in charge to perform unnecessary testing, although other causes of discoloration should be considered. As far as green urine discoloration is concerned, other factors such as drugs, dyes, certain nutritional supplements or even a Pseudomonas urinary tract infection may be at fault. On the other hand, pink urine syndrome due to propofol infusion seems to be even rarer. Although its presentation is not alarming, it may well increase the risk of uric acid lithiasis, a fact that the physician in charge should always keep in mind.

**Fig. 1 (abstract P405).**
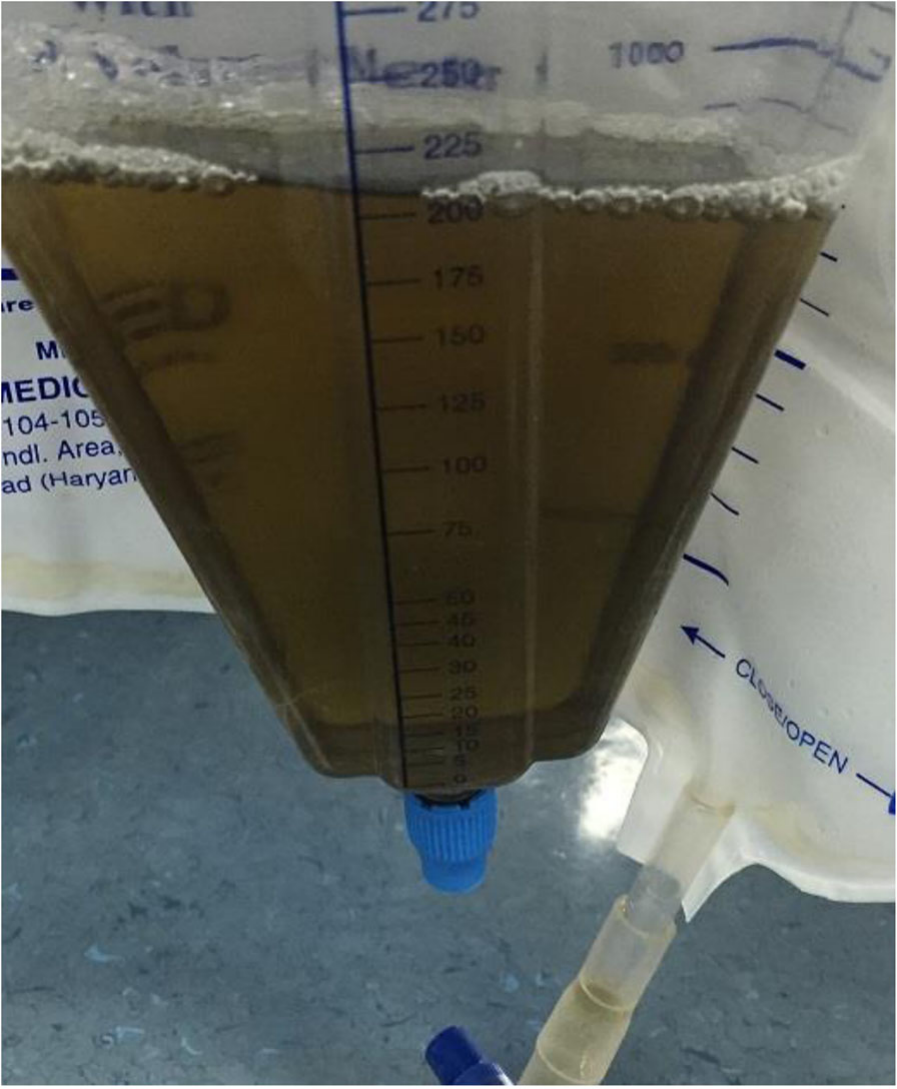
Dark green urine discoloration

**Fig. 2 (abstract P405).**
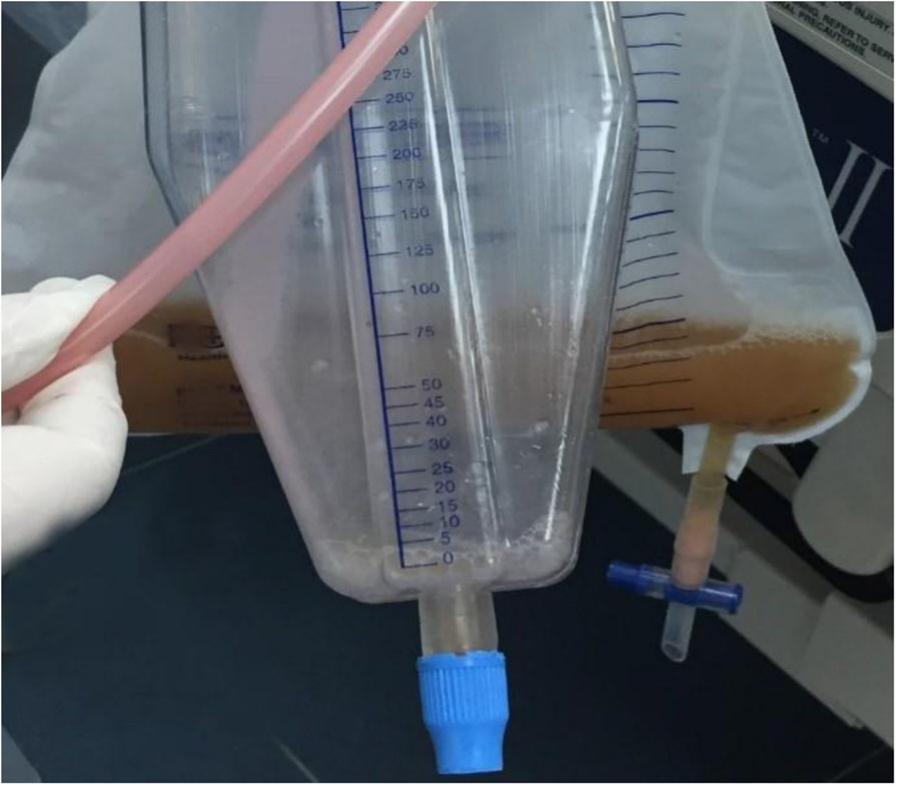
Pink urine discoloration

### P406 Propofol versus hepatic failure: is this correlation true?

#### P Pereira Travassos, R Telles da Silva Vale, W Geres da Costa, M Ferreira Pedroso, V Cordeiro Veiga, S Soriano Ordinola Rojas

##### Hospital BP - A Beneficência Portuguesa de São Paulo, Neurocritical Care Unit, São Paulo, Brazil

**Introduction:** Propofol is a drug widely used in critical patients for sedation. However, possible complications should be monitored systematically. The aim of this study was to evaluate hepatic complications in patients who used propofol for more than 48 hours. According to the institutional protocol, the maximum dose used was 4mg / kg / hour to minimize complications.

**Methods:** A retrospective analysis of 44 cases that used propofol for at least 48 hours in an intensive care unit and analysis of the hepatic alterations were performed.

**Results:** A retrospectively analyzed of 218 cases that used propofol for at least 48 hours was made. 56% of the patients were male, with a average age of 65,36± 15,21 years. The average time of use was 4,1 days, with 17 days maximum. 9% had an increase in transaminases 3 times higher than the reference value, 14% of the patients had already presented hepatopathy on admission. The values of INR, CPK, urea, creatinine and bilirubin levels were also analyzed. It was observed that 48 hours after the introduction of propofol 14% of the patients had an increase in the INR value and 5% of the patients presented a significant increase in the value of urea. 17% of the patients had bilirubin levels above the reference value due to the increase of 24% of direct bilirubin. The mean hospitalization time was 15.8 days and hospital stay equivalent to 22 days. The outcomes presented were 59% of the ob- tutes, of which none were hepatopathates.

**Conclusions:** Hepatic changes related to propofol are frequently observed and should be systematically monitored to ensure patient safety.

### P407 Concurrent use of propofol and clevidipine in critically ill patients: incidence of hypertriglyceridemia and pancreatitis

#### B Burbach, T Lam, J Jancik

##### Hennepin County Medical Center, Pharmacy, Minneapolis, United States

**Introduction:** Clevidipine (CLEV) and propofol (PROP) are lipid-based medications used in the intensive care unit (ICU) for hypertension and sedation, respectively. No data exists regarding potential adverse effects of concurrent therapy with this combination. This study aims to evaluate the incidence of hypertriglyceridemia (hTG) and pancreatitis in ICU patients using concurrent CLEV and PROP.

**Methods:** This was a single-center, retrospective chart review in patients utilizing CLEV and PROP concurrently from February 2015 to November 2018. Patients were included if they were 18 years and older, on CLEV and PROP concurrently for at least 24 hours with no more than 2 hours of interruption at a time, had at least one triglyceride (TG) level during concurrent therapy, and admitted to the medical or surgical ICU. The incidence of hTG (defined as TG equal to or greater than 500 mg/dL) and pancreatitis (provider assessment based on 2013 American College of Gastroenterology guidelines) was evaluated. Patients with and without hTG were compared to identify risk factors for the development of hTG.

**Results:** Of 145 patients screened, 30 patients were included which comprised 36 observations. The incidence of hTG was 13.9% with no patients developing pancreatitis. Patients with hTG had a higher median age compared to without hTG (64.5 vs. 38), p=0.015. In patients with hTG the median dose of CLEV and PROP were 16 mg/h and 42.3 mcg/kg/min, respectively, which was higher but not statistically significant when compared to patients without hTG. Cumulative lipid load (g/kg/d) was non-significantly higher in patients with hTG (2.6 vs. 1.9), p=0.599.

**Conclusions:** The incidence of hTG was comparable to what is cited in literature for PROP alone. Patients with hTG were older, had higher median CLEV and PROP doses, and a larger cumulative lipid load compared to patients without hTG.

### P408 Transition of dexmedetomidine infusion using oral clonidine in selected critically ill patients for cost minimization at a tertiary care hospital in U.A.E

#### S Rashid^1^, M Ur Rahman^2^, A Alia^2^, N Gebran^3^

##### ^1^Tawam Hospital, Pharmacy Department / Clinical Pharmacy, Al Ain, Abudhabi, United Arab Emirates; ^2^Tawam Hospital, Critical Care Medicine, Al Ain, Abudhabi, United Arab Emirates; ^3^Tawam Hospital, Pharmacy/Clinical Pharmacy, Al Ain, Abudhabi, United Arab Emirates

**Introduction:** The 2013 Society of critical care medicine guidelines for pain, agitation and delirium suggested use of non-benzodiazepine sedatives like dexmedetomidine which is associated with a reduced duration of mechanical ventilation, shorter length of hospital stay and a lower incidence of delirium [1]. Enteral clonidine represents a potentially less costly alternative for agitated patients with prolonged dexmedetomidine infusion. Limited literature exists examining this transition for management of agitation [2].

**Methods:** The critical care management initiated an action plan on the transition of patients with prolonged dexmedetomidine infusion to oral clonidine. A protocol was prepared with clinical pharmacist’s assistance. Risk factors were assessed and inclusion criteria were applied as per protocol. Dexmedetomidine infusion rate was reduced gradually with oral clonidine administration in selected patients. Other rescue managements were implemented as per protocol. Oral clonidine was then tapered down by reducing frequency of administration over few days.

**Results:** Post intervention data in 2017 showed significant decrease of dispensed doses and cost of the injections compared to 2016. The annual cost saving was 24% equating to 61,249 USD (Table 1, Figure 1).

**Conclusions:** Transitioning to clonidine may be safe and less costly method of managing agitated critically ill patients on prolonged dexmedetomidine infusion. More studies are needed to evaluate the efficacy and safety of this practice.

**References**

1. Gagnon DJ, et al. Pharmacotherapy. 2015

2. Terry K, et al. SAGE Open Med. 2015.

**Table 1 (abstract P408).** Dexmedetomidine cost per year in the critical care unit

Year	2015	2016	2017
Cost (USD)	245,255	254,317	193,067

**Fig. 1 (abstract P408).**
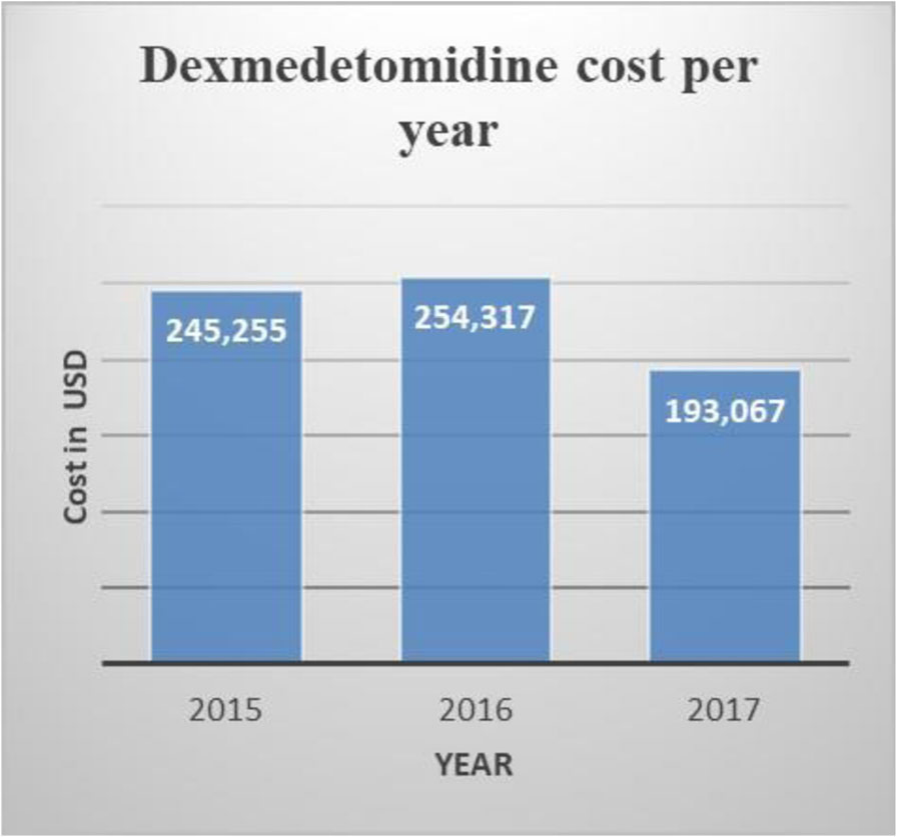
Reduced cost of 61,249 USD (24%) from 2016 to 2017

### P409 Incidence of dexmedetomidine associated fever at a level 1 trauma center

#### NA Beaupre, JT Jancik

##### Hennepin County Medical Center, Pharmacy Department, Minneapolis, United States

**Introduction:** We evaluated the incidence of dexmedetomidine associated fever (DAF) in a level 1 trauma center’s medical intensive care unit (MICU). Hypotension and bradycardia are the most commonly reported adverse effects associated with dexmedetomidine (DEX) infusion. Case reports suggest DEX can cause fevers and the clinical trials that led to the approval of DEX demonstrated fever rate to be 4-5% [1].

**Methods:** This was a single-center, retrospective chart review of patients admitted to the MICU at Hennepin County Medical Center between March and July of 2018 that were started on a DEX infusion. Patients were included if they were 18 years and older, on a DEX infusion for at least 12 hours, and had temperature data available. Fever was defined as >38.3C and other causes of fever including infections, medications, withdrawal, recent surgery, thromboembolic disease, thyroid disorders and seizures were excluded from analysis.

**Results:** Of the 246 patients screened, 120 were included. The mean age was 50 years and 63.3% were males. Of all the patients included, the mean change in temperature after initiation of DEX infusion was +1.1C from baseline. The mean initial dose was 0.3 mcg/kg/hr. Four of 120 patients (3.3%) had a DAF. Of those that had a DAF, the median initial dose was 0.3 mcg/kg/hr; the median time of infusion was 43.5 hours; and the median cumulative dose was 0.57 mcg/kg/hr. The median time to fever after initiation of DEX was 7 hours, with a range of 5 to 20 hours. The median time to fever cessation after discontinuation of DEX was 3 hours.

**Conclusions:** In our population, the incidence of dexmedetomidine associated fever was relatively rare at 3.3% and similar to current literature rates.

**Reference**

1. Hospira (2014) Precedex® (dexmedetomidine hydrochloride) [package insert]. Lake Forest, IL; Hospira, Inc.

### P410 Light sedation with dexmedetomidine in ICU patients with acute brain injury

#### S Carelli, G De Pascale, N Filetici, GM Maresca, SL Cutuli, CM Pizzo, L Montini, G Bello, MG Bocci, A Caricato, G Conti, M Antonelli

##### Fondazione Policlinico A. Gemelli IRCCS, Catholic University of the Sacred Heart, Department of Anaesthesiology and Intensive Care, Rome, Italy

**Introduction:** We aimed to evaluate safety and efficacy of light sedation with Dexmedetomidine (Dex-LS) in acute brain injury (ABI) patients.

**Methods:** Retrospective analysis on ICU patients with traumatic/medical ABI, out of the neuroprotection window and undergoing Dex-LS. Data of pre-infusion and infusion periods were compared.

**Results:** 101 patients (age 51±24, males 83.2%) were included. Traumatic ABI was the main admission diagnosis (Table 1). Subarachnoid haemorrhage (38.6%) and subdural haematoma (28.7%) were the main injury types; most patients reported combined lesions. Median time to Dex-LS start was 4 [2-8] days and its main features are listed in Table 2. Median Richmond Agitation-Sedation Scale value significantly increased during Dex-LS (-2[IQR -3/0] vs 0[IQR -1/0], p<0.01). Out of 101 patients, 80 underwent mechanical ventilation (MV) during both pre-infusion and infusion periods, 11 were weaned before Dex-LS and 10 never received MV. During Dex-LS the median duration of MV decreased (96[48-156] vs 39[12-72] hours, p<0.01) while the MV weaning rate significantly increased (Fig 1); 16 of the 28 patients who had failed weaning attempts during the pre-infusion period were also weaned. The spontaneous breathing rate significantly rose during Dex-LS (20.8% vs 72.3%, p<0.01). Bradycardia events occurrence was significantly higher during Dex-LS (2% vs 23%, p<0.01) but never imposed Dex suspension. Arterial hypotension requiring vasopressors was significantly more frequent in the pre-infusion period (41.6% vs 26.7%, p=0.03). Neither epileptic activities rate nor median intracranial pressure values increased during Dex-LS (p=1 and p=0.16, respectively). Overall ICU and hospital mortality rates were 3.9% and 7.9%, respectively.

**Conclusions:** Dex-LS among ICU patients affected by ABI turned out to be feasible and safe. It enabled discontinuation from MV and maintenance of spontaneous breathing in the majority of cases, including most patients who had experienced a weaning failure before Dex infusion.

**Table 1 (abstract P410).** Patients clinical characteristics at ICU admission and at Dex-LS start

Polytrauma with traumatic ABI (%)	64 (63.4)
Isolated traumatic ABI (%)	14 (13.8)
Medical ABI (%)	23 (22.8)
Injury Severity Score±SD	23.7 ± 11.1
Head-Abbreviated Injury Scale±SD	3 ± 1.1
GCS at admission [IQR]	10 [7 – 14]
SAPS II at Dex-LS start±SD	36.2 ± 14
SOFA at Dex-LS start [IQR]	4 [3-7]

**Table 2 (abstract P410).** Main features of Dex-LS

Dex length of infusion, hours [IQR]	64 [32.5-120]
Dex start dosage (mcg/kg/h)±SD	0.7 ± 0.3
Dex median dosage (mcg/kg/h)±SD	0.6 ± 0.3
Dex maximum dosage (mcg/kg/h)±SD	0.9 ± 0.4
Dex dosage at suspension (mcg/kg/h)±SD	0.5 ± 0.4
Propofol co-infusion (%)	35 (34.7)
Remifentanil co-infusion (%)	59 (58.4)
Antipsicotic drugs co-administration (%)	40 (39.6)

**Fig. 1 (abstract P410).**
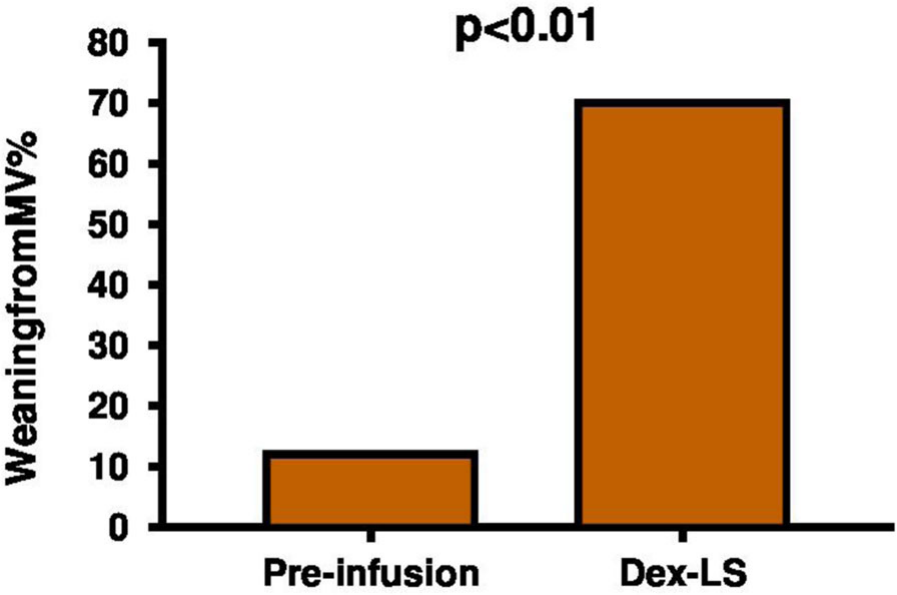
Rate of weaning from MV during pre-infusion period and Dex-LS

### P411 Delirium score in the ICU. A pilot study

#### M Smith^1^, T Haberlandt^1^, LS Nielsen^1^, AC Brøchner^2^, H Jensen^1^

##### ^1^Hospital Lillebaelt, Department of Anesthesiology and Intensive Care, Kolding, Denmark; ^2^Hospital Lillebaelt, Department of Anesthesiology and Intensive Care, The Department of Regional Health Research, Region of Southern Denmark, Kolding, Denmark

**Introduction:** The aim of the study was to compare the Confusing Assessment Method of the Intensive Care Unit (CAM-ICU) and the Nursing Delirium Scoring Scale (Nu-DESC) for assessment of delirium in the ICU. Furthermore we wanted to test the interpersonal variation of the Nu-DESC. Delirium is proved to be associated with increased mortality [1]. Nu-DESC is an observational five-item scale that does not require patient participation and is adapted to the fluctuating nature of delirium. Each item can be scored from 0 to 2. Delirium is defined with a score > 2. The Nu-DESC has recently been translated into Danish (Nu-DESC DK) but has not been validated.

**Methods:** ICU patients, who met the inclusion-criteria for the CAM-ICU were scored with both CAM-ICU and Nu-DESC DK. Patients were scored of two independent nurses at approximately the same time every day.

**Results:** A total of 24 patients were enrolled, and 54 comparisons between CAM-ICU and Nu-DESC DK were registered (Figure 1).There was agreement between Nu-DESC and CAM-ICU in 46 of registrations (hereof 44 registrations were delirium negative). In interpersonal variation, 14 registrations were made. The conclusion was identical in 86% of registrations, but only 57% agreed in all 5 scoring-scale items (all negative).

**Conclusions:** A high agreement between Nu-DESC and CAM-ICU was found however the comparison was based on predominately patients with negative delirium score. The interpersonal variation of Nu-DESC scoring was substantial. A future validation of the Nu-DESC DK as a screening tool in the ICU requires thorough training and instructions to minimize interpersonal variation.

**Reference**

1) A. Hargrave et al. Psychosomatics, 58(6): 594–603, 2017.

**Fig. 1 (abstract P411).**
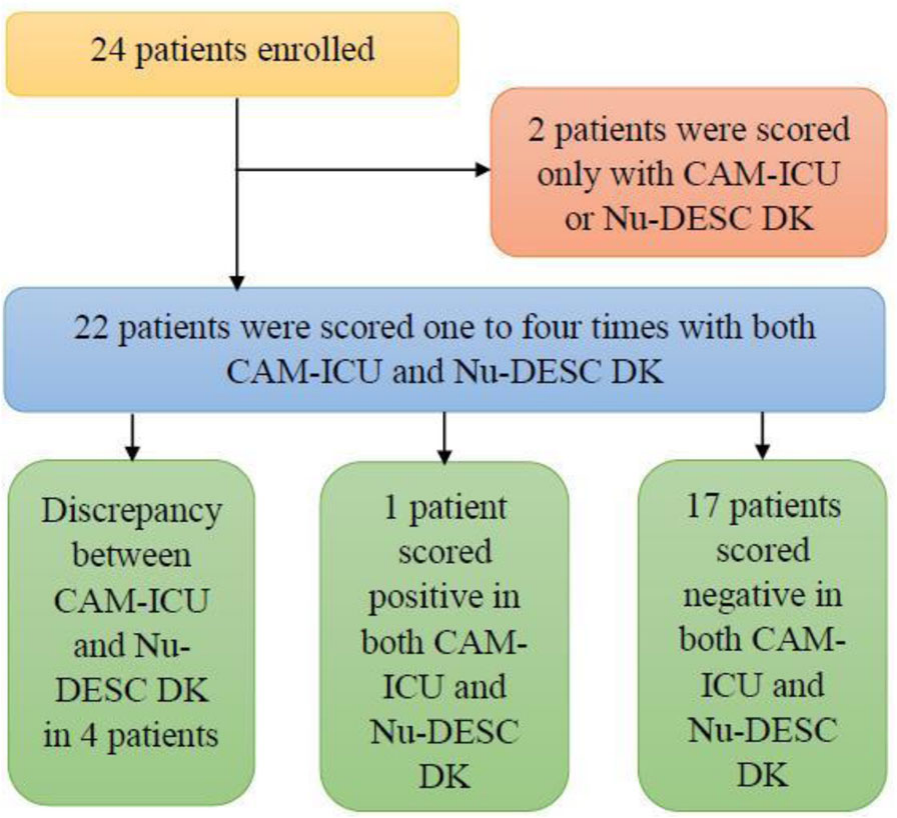
Flowchart of enrolled patients

### P412 Prevalence and risk factors for delirium in acute stroke patients: a prospective cohort study

#### T Nishida, R Kajikawa, T Murakami, T Yoshihara, A Wakayama

##### Osaka Neurological Institute, Neurosurgery, Osaka, Japan

**Introduction:** Delirium is common among critically ill patients, leading to poor hospital outcomes. However, its epidemiology in the acute stroke setting is not well established. The purpose of this study was to investigate the prevalence and risk factors for the development of delirium in acute stroke patients.

**Methods:** We conducted a prospective study of acute stroke patients consecutively admitted to our stroke care unit from July 2018 to October 2018. The diagnosis of delirium was based on the Intensive Care Delirium Screening Checklist (ICDSC). Patients with an ICDSC score of 4 or higher were considered delirious, and those with a score of 3 or less were not. Baseline demographics, information regarding preadmission risk factors for delirium and clinico-radiological profiles were compared between patients with and without delirium. Logistic regression analyses were used to determine the associated risk factors.

**Results:** Among the 131 patients assessed, 44 (34%) patients presented with delirium. Patients with delirium were older (84 vs. 71 years, p <0.01) and had higher National Institutes of Health Stroke Scale (NIHSS) scores (9 vs. 2, p <0.01) on admission. Risk of delirium was independently associated with a history of dementia (odds ratio [OR], 4.27; 95% confidential interval [CI], 1.17-16.58; p = 0.030) and hemorrhagic stroke (OR, 3.64; 95% CI, 1.10-12.55; p = 0.035).

**Conclusions:** Delirium was observed in one-third of patients admitted with an acute stroke. Older age, higher NIHSS score, dementia and hemorrhagic stroke were predictors of delirium.

### P413 Postoperative delirium in urgent surgery

#### V Pajtic^1^, L Gvozdenovic^2^

##### ^1^Clinical centre of Vojvodina ( University of Novi Sad, Medical faculty), Emergency Center, Novi Sad, Serbia; ^2^Clinical centre of Vojvodina (University of Novi Sad, Medical faculty), University of Novi Sad, Medical faculty, Novi Sad, Serbia

**Introduction:** Postoperative delirium can be presented s an altered mental state of the patient immediately after surgery in general anaesthesia where the patient can not mentally and physically satisfy the needs and expectations in relation to the condition in which he is.

**Methods:** A prospective study, for a year, covered 100 patients who were admitted to the Emergency Center, Clinical Center of Vojvodina. After examination, taking anamnestic data and diagnosis, they are subjected to emergency abdominal (cholecystectomia and appendectomia) or orthopedic (Fractura femoris) surgery in general anesthesia within 4 hours of admission. The study included patients aged over 18 years, ASA I, II, III, IV groups, who voluntarily and with the signed consent accepted to participate in the study.

**Results:** The results obtained showed a statistically significant fact that fewer points on the test, from 11 to 20 points, received older patients who underwent an urgent surgical procedure, over 60 years of age, of which 27% . Also statistically significant data were obtained that patients who used a higher amount of sedatives during emergency surgery, 47% had a worse test result than under 20 points due to increased preoperative anxiety.

**Conclusions:** The older population is more susceptible to postoperative delirium, especially in emergency surgery situations, which they carry, unpreparedness for surgery, increased use of medication for calm, unpredictability of the duration of surgery, and therefore anesthesia as well the use of anticholinergics, which is sometimes impossible to avoid in operative procedures such as gall bladder surgery. The results of the study suggest that in cases of emergency surgery, the use of protocols for postoperative delirium should be planned regularly to prevent or at least mitigate the clinical picture of delirium that can lead to complications postoperatively.

### P414 Prediction and detection of delirium during perioperative care – a quality improvement project

#### A Boedo^1^, S Mohammed^2^, P Costa^1^, S Mandalia^1^, M Vizcaychipi^3^

##### ^1^Chelsea and Westminster Hospital, Planned Care Division, London, United Kingdom; ^2^Imperial College London, School of Medicine, London, United Kingdom; ^3^Chelsea and Westminster Hospital, Anaesthesia and Intensive Care Medicine, London, United Kingdom

**Introduction:** Delirium is a serious and often underestimated condition with implications for morbidity, mortality and healthcare costs. As it presents in a wide range of settings from admission to discharge, early prediction and risk assessment are essential. E-PRE-DELIRIC is a delirium prediction score which has been validated in ITU patients but not in other populations, and we conducted a quality improvement project using this score to assess its utility in other settings.

**Methods:** Data was gathered from three patient categories: those undergoing elective surgery (ES), admissions to the Emergency Observation Unit (EOU) in the A&E, and patients with fractured neck of femur (NOF). Clinical notes were reviewed to collect data to calculate E-PRE-DELIRIC score at admission, along with a number of other clinical variables including incidence of delirium, and statistical analysis performed.

**Results:** A total of 103 patients were included, with 52 in the ES group, 32 in the EOU group, and 19 in the NOF group respectively, with an overall average E-PRE-DELIRIC score of 17.5%. ES had a 5.4% average E-PRE-DELIRIC score, a mean age of 56 and no cases of delirium. The EOU group had an average age of 63, a 20.5% average E-PRE-DELIRIC score and no incidence of delirium. The NOF group had a mean age of 84 and an average E-PRE-DELIRIC score calculated on admission of 45.3%. This was the only group in which 5 patients developed delirium. A 50% cut off was demonstrated to be the most accurate to predict delirium in this population with a sensitivity of 0.80 and a specificity of 0.78.

**Conclusions:** Despite the limitation of a small sample size, this project has shown that E-PRE-DELIRIC score could be a useful tool to predict patients at high risk of delirium in a non-ITU setting, with a 50% cut off in hip fracture patients. Further investigation should be conducted into the potential use of E-PRE-DELIRIC in non-ITU patients.

### P415 Comparison of long-term mortality between patients with and without delirium during admission in medical intensive care units in a university hospital

#### N Kongpolprom

##### King Chulalongkorn Memorial Hospital, Pulmonary Unit, Bangkok, Thailand

**Introduction:** Delirium is a common complication in critically ill patients. Moreover, delirium is associated with poor clinical outcomes, including increased duration of mechanical ventilation, prolonged length of stay in ICU, and high hospital mortality. However, there are limited data of long-term mortality in delirious patients. This study aimed to compare 2-year mortality between patients with and without delirium during ICU admission.

**Methods:** We conducted a prospective cohort study in 153 hospital survivors who were admitted in our medical ICUs between 1 October 2016 and 31 March 2017. During this period, there were 270 patients admitted in the ICUs, including 128(47.4%) delirious patients, 114 (42.22%) non-delirious patients and 28(10.37%) comatose patients. Subsequently, 60 of 128 (46.88%) delirious patients and 93 of 114 (81.58%) non-delirious patients could be discharged from the hospital. We evaluated the 2-year mortality in the hospital survivors.

**Results:** Totally, 153 patients participated in our study. The majority of them (55.1%) were male with the median age of 64[45, 76.25] years and the median APACHE II score on the first day of ICU admission of 13[8, 18]. The 2-year mortality rate of the hospital survivors who had the history of delirium during ICU admission tended to be higher than the rate of those without delirium; 31.7% (19/60) in the delirium group VS 24.7% (23/93) in the non-delirium group. The median survival times after hospital discharge in the non-survivors were not different; 3.25[1.25, 11.75] months in the delirium group VS 4.5[1.38, 12] months in the non-delirium group. In addition, causes of death were associated with their underlying diseases.

**Conclusions:** The 2-year mortality rate tended to be increased in the hospital survivors with the history of delirium during ICU admission.

### P416 Risk factors associated with the onset of delirium in a post-operative cardiac surgical ICU

#### V Ajello, G Podagrosi, M Flaminio, M Moresco, A Farinaccio, P Prati, D Colella

##### Policlinico Tor Vergata, Cardio-Thoracic anesthesia, Rome, Italy

**Introduction:** Delirium is a disabling mental disorder associated with increased morbidity, decreased functional status, cognitive decline and increased long-term mortality. Despite the impact of this condition, few studies identified risk factors associated with this condition in the specific setting of cardiac surgery. In this pilot observational study we assessed the risk associated with the development of delirium in a cohort of patients who underwent cardiac surgery

**Methods:** We prospectively included 145 patients (66,4 years, 42 F/103 M) who underwent cardiac surgery between April and June 2018 at cardiac surgical ICU of Tor Vergata Policlinic in Rome, Italy. Data about pre-intra and postoperatory period were collected. The presence of delirium was diagnosed with the CAM–ICU scale. Univariate logistic regression was used to evaluate perioperative risk factors associated with delirium.

**Results:** Among the 255 patients evaluated, 13 developed symptoms of delirium (5.09%). Risk of delirium was associated with preoperatory EuroSCORE II (p=0.028) and history of previous cardiac surgery (p=0.042). Moreover, in the intraoperatory period the risk of Delirium was associated with red blood cell transfusion, intervention for Aortic Dissection (p=0.013), Hypothermic Circulatory Arrest (HCA) with Anterograde cerebral perfusion (ACP) (p=0.036) (Table 1). In the postoperatory period risk of Delirium was associated with levels of creatinine clearance (p=0.035) and C-reactive protein (CRP) (p=0.029).

**Conclusions:** Delirium is relatively frequent in the cardiac surgical ICU. High EuroScore correlates with the onset of delirium, suggesting that delirium is linked with preoperatory comorbidities. The complexity of surgery has a big influence on the development of delirium, especially in the cases of aortic dissection. Delirium was associated with intraoperatory blood transfusions. Finally, our data point to a bridge between postoperatory electrolytic disturbances, as well as inflammation as factors potentially triggering delirium onset.

**Table 1 (abstract P416).** Risk factors associated with the onset of delirium

Variable	p value	OR	“C.I. Lower – Upper”
EuroSCORE II	0.028	1.19	1.02 - 1.4
Creatinin Clearance	0.035	0.96	0.93 - 0.99
Previous Cardiac Surgery	0.042	4.88	1.05 - 22.50
Aortic Dissection	0.013	11	1.67 - 72.39
HCA ACP	0.036	9	1.15 - 70.42
CRP	0.029	1	1.00 - 1.01
Intraoperative Blood Trasfusion	0.058	5	0.94 - 26.37

### P417 Audit of sepsis mortality in critical care

#### RQ Malik^1^, S Shah^1^, I Momadov^2^, N Sudhan^1^

##### ^1^BHR TRust, Queens Hospital, Critical Care, Romford, United Kingdom; ^2^BHR TRust, Queens Hospital, Romford, United Kingdom

**Introduction:** We did a retrospective case note study of mortality due to sepsis of our unit over three months as observational study in which we noted the causes of deaths, origin of sepsis, organism, patient characteristics and ICNARC Physiology scores and ICNARC H2015 model predicted risk of acute hospital mortality percentage.

**Methods:** ICNARC data base was used to gather the data and coding was used to identify the patients with sepsis for three months. Patients mortality attributed to sepsis were identified from mortality list.Causes of death were noted from patients notes and death certificates.Cyber Lab was used to access the data and case note were ordered for review.Patients characteristics were noted including DNACPR orders and treatment withdrawal orders. Scores (apache scores, ICNARC Physiology scores, ICNARC H2015 predicted risk models of acute hospital mortality percentage) were noted.

**Results:** Mortality percentage was found to be 17% as per codig which was reduced to 12% as 5% deaths were attributed to other causes. 44% patient had DNACPR in first 24hrs. Average length of stay was 5.58 days with median of 2.53 days.Median age was 66yrs in surviving age group and 78years in other. ICNARC physiology score 23 with predicted risk of 56.8%. Commonest cause was found Pneumonia 49% followed by Urine tract infection. 20% patients were with no source identification.

**Conclusions:** Conclusion was made that we do need to improve the coding as significant percentage was mentioned as sepsis as cause of death where clinicians differed. Pneumonia was found to be the commonest killer in critical care followed by urine tract infection. It was pointed to be useful to carry out further audit targeting Pneumonia .Review of ICNARC Case Mix Program, development of ICNARC Physiology score, which provides excellent local use with downside of lacking international comparison was done also.

### P418 Access to high-performing hospitals for sepsis care in New York state

#### LK Stratton^1^, JM Kahn^2^, DC Angus^2^, IJ Barbash^3^, CD Drake^4^, NM Mohr^5^, CW Seymour^2^, DJ Wallace^2^

##### ^1^University of Pittsburgh Medical Center, Critical Care Medicine, Pittsburgh, United States; ^2^University of Pittsburgh Medical Center, Department of Critical Care Medicine, Pittsburgh, United States; ^3^University of Pittsburgh Medical Center, Department of Medicine, Pittsburgh, United States; ^4^University of Pittsburgh Graduate School of Public Health, Department of Health Policy and Management, Pittsburgh, United States; ^5^University of Iowa Health Care, Department of Emergency Medicine, Iowa City, United States

**Introduction:** Hospitals vary widely in the quality of care they provide for septic patients. Since many septic patients present to their nearest hospital, local variations in care quality may lead to geographic disparities in access to optimal sepsis care. We sought to better understand geographic access to high quality sepsis care, taking advantage of publicly reported data on sepsis management and outcomes in a large US state.

**Methods:** We performed a cross-sectional analysis of geographic access to high quality sepsis care, taking advantage of a 2013 New York state initiative that mandates public reporting of sepsis quality data to the state government. We linked these data to the locations of hospitals in New York state from the US Centers for Medicare and Medicaid Services and population data from the US Census Bureau for 2016. We defined hospital sepsis performance using self-reported risk-adjusted mortality rates (RAMR) and defined high-performing hospitals as those with a RAMR <20%, which represents the lower end of short-term mortality typically observed in sepsis. We used ArcGIS to generate drive-time estimates and assess population access to high performing acute care hospitals for sepsis care.

**Results:** 161 hospitals publicly reported treating 47,081 cases of sepsis from a population of 19,569,040 persons. Overall access to an acute care hospital was excellent at the 45-minute drive threshold (99.3%), good at the 30-minute threshold (95.6%), and marginal at the 15-minute threshold (84.4%). We classified 46 hospitals (28.6%) as high-performing based on a RAMR <20%. High-performing hospitals reported 12,666 (26.9%) of the total sepsis cases. High-performing hospitals were geographically dispersed across the state, although population access diminished substantially with increasing drive times (92.6% at 45-minutes, 83.5% at 30-minutes, and 59.8% at 15-minutes; Figure 1).

**Conclusions:** One in six people do not have timely access to a high performing hospital for sepsis care using a 30-minute threshold.

**Fig. 1 (abstract P418).**
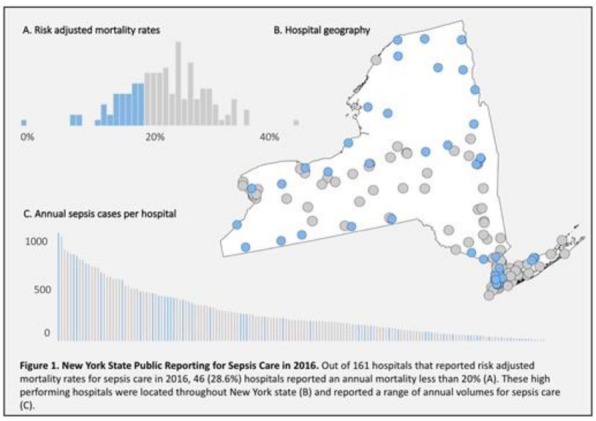
New York State Public Reporting for Sepsis Care in 2016. Out of 161 hospitals that reported risk-adjusted mortality rates for sepsis care in 2016, 46 (28.6%) hospitals reported an annual mortality of less than 20% (A). These high performing hospitals were located throughout New York state (B) and reported a range of annual volumes for sepsis care (C)

### P419 After-hour admission and clinical outcomes in hospitalized patients with sepsis – a nationwide cohort study

#### S Bernet^1^, L Gut^1^, U Wagner^2^, B Mueller^1^, P Schuetz^1^, A Kutz^1^

##### ^1^Kantonsspital Aarau, Division of General and Emergency Medicine, Aarau, Switzerland; ^2^Swiss Federal Office for Statistics, Swiss Federal Office for Statistics, Neuchatel, Switzerland

**Introduction:** Sepsis is associated with detrimental outcomes requiring early diagnosis and bundled therapeutic intervention. During after-hours care level of staffing and the availability of diagnostic resources is lower. We therefore aimed to assess whether in-hospital mortality rates differ between after-hour and routine-hour admission in patients with sepsis.

**Methods:** We analyzed all acute care admissions for the main diagnosis of sepsis in Switzerland between 2006 and 2016 using administrative data. As a primary outcome we compared in-hospital mortality among septic patients admitted during routine-hours (8 am to 8 pm) with those admitted during after-hours: night (8 pm to 8 am) and weekend. Secondary outcomes were intensive care unit (ICU) admission, intubation rate, and 30-day readmission rate.

**Results:** In total, we included 86`597 hospitalizations, 60% during routine-hours, 23% at night, and 17% on weekends. Compared with routine-hour admissions, multivariate analyses revealed a higher risk for in-hospital mortality in patients admitted on weekends (Odds ratio [OR] 1.05, 95% confidence interval [CI] 1.01-1.10), but lower risk in patients admitted at night (OR 0.93, 95% CI 0.89-0.97).

Adjusted risks for ICU admissions and intubation rates were higher during nightshifts (ICU: OR 1.27, 95% CI 1.23-1.32; intubation: OR 1.30, 95% CI 1.24-1.36) and during weekends (ICU: OR 1.14, 95% CI 1.10-1.19; intubation: OR 1.18, 95% CI 1.12-1.25) in comparison with routine-hour admissions. 30-day readmission rates did not differ between routine- and after-hours. Results also showed variations in clinical outcomes between different regions in Switzerland.

**Conclusions:** For patients with sepsis, admission on weekends is associated with higher in-hospital mortality, increased ICU admission and intubation rates. Whether our results are related to differences in staffing and lower access to care remains to be investigated.

### P420 Improving patient safety in a large teaching hospital through the introduction of a multidisciplinary critical care medication safety group (CCMSG)

#### R Sloss^1^, F Ahmad^1^, J Townsend^2^, R Mehta^1^

##### ^1^King´s College Hospital, Pharmacy Department, London, United Kingdom; ^2^King´s College Hospital, King´s Critical Care, London, United Kingdom

**Introduction:** Medication errors are estimated to account for 78% of all medical errors in intensive care, with an average of 1.7 incidents per patient per day [1]. This poses a significant safety risk. A previous study found that the implementation of a multidisciplinary medication safety group in intensive care increased reporting of errors and near misses [2]. The purpose of our work was to set up a multidisciplinary group to provide a forum to review and improve medication safety at all stages of the process. Here we discuss some of the initiatives and outcomes implemented in the last 12 months.

**Methods:** CCMSG was formed in 2013, under the leadership of the critical care pharmacy team, with representation from medical and nursing disciplines. The group meet fortnightly to analyse trends in medication errors, implement changes to local practice and review outcomes to improve patient safety. The cohesive, multidisciplinary nature of the group allows medication safety initiatives to be delivered in the most effective way.

**Results:** On average, CCMSG reviewed 21 medication errors per month. The most common high risk drug classes involved are seen in Table 1. Medication safety initiatives implemented were based on these trends and included writing guidelines and policies, bedside education, teaching and training, informatics optimisation and operational changes. Examples are seen in Table 2.

**Conclusions:** Initiation of a CCMSG provides a cohesive approach to facilitate the implementation of targeted safety initiatives, which are proven to reduce some of the most common medication errors in critical care. In addition, these often result in optimisation of operational and financial inefficiencies.

**References**

1. Moven, E et al. (2008). Crit Care 12, 208.

2. Wong, A et al. (2017). Intensive Care Med Exp 5,408-9.

**Table 1 (abstract P420).** Drug classes involved in adverse incidents

Drug Class	Number of Adverse Incidents (AIs)	% of Total AIs (n = 254)
Controlled Drugs (CD)	59	23%
Antimicrobials	56	22%
Insulin	14	6%
Anticoagulants	23	9%
Potassium	2	0.8%

**Table 2 (abstract P420).** Medication safety initiatives

CCMSG Initiative	Outcome
Verbal orders policy	Process to give drugs in time critical situations
Argatroban monitoring document	AIs reduced by 100% and expenditure by 79%
Vancomycin guideline update and bedside teaching	AIs reduced from 71% to 55% of all antibiotic AIs
Nurse drug education packs	Reinforced learning and facilitated reflection
Glycaemic control guideline linked to prescription	Insulin AIs reduced by 12.5%
Introduction of bottle adaptors for liquid CDs	CD balance discrepancy AIs reduced by 82%
Nebulised mucolytic drug switch	Evidence based switch and cost saving

### P421 Adverse events during in-hospital transport of critical patients in large hospital

#### P Travassos, W Geres, R Vale, V Veiga, S Rojas

##### Hospital BP - A Beneficência Portuguesa De São Paulo, Neurocritical Care Unit, Sao Paulo, Brazil

**Introduction:** Intra-hospital transport is related to a high incidence of adverse events and negative outcomes. The objective of this work is to describe the incidence of clinical and non-clinical events during in-hospital transport of critical patients and to analyze the associated risk factors

**Methods:** A cohort study with retrospective collection, from October 2016 to October 2017, which analyzed all intrahospital transports for diagnostic and therapeutic purposes in a large hospital, and evaluated the adverse events and related risk factors.

**Results:** In the period, 1559 intrahospital transports were performed in 1348 patients, with a mean age of 66 ± 17 years, with mean transport time of 43 ± 34 minutes. 19.8% of patients were on vasoactive drugs; 13.7% in sedatives and 10.6% in mechanical ventilation. Clinical events occurred in 117 transports (7.5%) and non-clinical in 125 transports (8.0%). Communication failures were prevalent, however, by applying multivariate analysis, the use of sedatives, noradrenaline, nitroprusside and time of transport were associated with clinical adverse events. Use of dobutamine and transport time have been associated with non-clinical events. At the end of transport, 98.1% of the patients presented clinical conditions unchanged in relation to their baseline state.

**Conclusions:** Intrahospital transport is related to a high incidence of adverse events, and the time of transport and use of sedatives and vasoactive drugs have been related to these events.

### P422 Lessons learnt implementing a business continuity plan in critical care, during an informatics system downtime in a Central London teaching hospital

#### T Lalmahomed, R West, F Master, R Mehta, R Sloss, S Shah, P Hopkins

##### Kings College Hospital, Critical Care, London, United Kingdom

**Introduction:** CIS/hospital electronic medical records downtime can cause major disruptions to workflow, patient care, key communication and information continuity [1]. Here we describe the consequences of deploying a business continuity plan (BCP) designed to support a critical care clinical informatics system (CIS) failure, during an 8-hour unplanned downtime in a large central London ICU.

**Methods:** The institutional BCP was developed through an iterative process based on CIS provider recommendations and internal workflow knowledge. It consisted of a Web offline chart (WOC) that is accessible at every computer connected to the network (in the event of a CIS server fault), and via hard copy from designated back up computers connected to a printer (in the event of whole network loss). Operational and clinical consequences were recorded during informal and formal debrief of the informatics team. The decision making around ´drop-to-paper´ was reviewed.

**Results**

- The BCP permitted ´drop–to-paper´, service continuity and controlled uptime
- Patchy network loss and lack of a general institutional BCP delayed initial system failure diagnosis (network vs primary server); reduced reliability of ´read-only´ data and delayed ´drop-to-paper´
- Day-to-night handover during downtime led to loss of ´memory´ of key patient data/events, and should have accelerated decision to ´drop-to-paper´
- Transfer of prescriptions was time consuming, distracting (occupied CIS team) and prone to error

**Conclusions:** Previous end-to-end testing of the BCP had not identified many of the observations and recommendations that came from the analysis of an actual period of unplanned downtime. We recommend sharing of similar experiences and scheduled high-fidelity simulated downtime in other institutions to replicate real world conditions, particularly in a critical care setting.

**Reference**

[1] Nelson N. J Critical Care, 22: 45-50, 2007.

### P423 Factors associated with medical patients admitted in intermediate ward who subsequently transfers to intensive care unit of Ramathibodi hospital

#### P Theerawit, S Panpimanmas, T Petnak, Y Sutherasan

##### Ramathibodi Hospital, Medicine, Bangkok, Thailand

**Introduction:** Many patients transferred to intermediate care unit became worsening and finally need ICU admission in few days. This study aimed to identify what were risk factors associated with ICU transfer among patients initially hospitalized at IMCU.

**Methods:** We conducted retrospective study in patients hospitalized at IMCU. Medical record review was performed. Main interested variables were physiologic parameters. The comparison was made by Chi-Square or Student’s T-test depending on type of data. Multiple logistic regression analysis, to identify risk factors.

**Results:** A total of 1,650 records were reviewed. Patients of 140 were excluded due to being non-resuscitated patients, thus the remaining 1,510 patients were included. A number of patients finally transferred to ICU were One hundred and twenty-three. The median length of stay in IMCU of those patients was two days. Logistic regression analysis revealed that immunocompromised status (OR 2.19; 95%CI 1.45-3.31; P<0.001), SBP (OR 0.97; 95%CI 0.96-0.97; P<0.001), RR (OR 1.03; 95%CI 1.01-1.05; P=0.018) and GCS (OR 0.89; 95%CI 0.85-0.94; P<0.001) were predictors of ICU transfer. We developed a simple score to predicting ICU transfer from previous variables and performed analysis of AUC of ROC, which was compared to that of APACHE II. The result showed the AUC of ROC of a new score was slightly higher than the APACHE II, namely 0.797 vs. 0.720 respectively.

**Conclusions:** The immunocompromised patients take two times higher risk than the immunocompetent ones regarding ICU transfer. The other risk factors are lower GCS, lower SBP, and higher RR. A newly developed score may be a promising tool for predicting and triaging site of care in patients who require IMCU admission.

### P424 Early readmission

#### P Travassos, R Vale, W Geres, V Veiga, S Rojas

##### Hospital BP - A Beneficência Portuguesa De São Paulo, Neurocritical Care Unit, Sao Paulo, Brazil

**Introduction:** Discharge from the ICU patient is a critical moment because it exposes him to preventable errors and adverse events, so the importance of well defined criteria. The readmission rate varies between 4-6% and mortality between 4-7% (REF). The objective is To evaluate the early readmission rate and the main associated characteristics in Neurological ICU in the 4 year period, from 2014 to 2018

**Methods:** A review of all early readmissions (<48 hours) was carried out in the period from 2014 to 2018, using a database of the hospital management system and evaluated compliance with the ICU discharge criteria according to the ICU discharge protocol and its correlation with readmission.

**Results:** In the analyzed period, 9,319 patients were admitted to the ICU, of which 25 were readmitted (0.32%), 7 in 2017, 7 in 2015, 4 in 2016, 2 in 2017 and 5 in 2018. The reasons for rehospitalization were lowering the level of respiratory failure, convulsive crisis, acute pulmonary edema, hyponatremia, sudden motor deficit and cardiac arrhythmia. Of the 25 patients, only 1 (4%) did not present criteria according to the ICU discharge protocol. Patients who were readmitted had an increase in their length of stay in the ICU (8.4 days x 5.6 days), and there was no correlation with increased mortality in the sample. Mean age was 68.7 years. Occupancy rate was 88.14%. SAP3 average was 43.4. Mean SOFA on admission was 2.28. 39.57% of the patients were surgical.

**Conclusions:** Despite the high complexity, there is a low rate of early readmissions in the unit. This evidences a safe process of discharge from the ICU and quality of care

### P425 How composition and processes of the interdisciplinary team impacts transition of International Medical Graduates (IMGs) into their ICU fellowship

#### A Mecklenburg^1^, U Najeeb^2^, L Rose^3^, L Lingard^4^, D Piquette^3^

##### ^1^Interdepartmental Division of Critical Care, University of Toronto, University of Toronto, Toronto, Canada; ^2^Sunnybrook Health Sciences Centre, Department of Medicine, University of Toronto, Toronto, Canada; ^3^Interdepartmental Division of Critical Care, University of Toronto, Toronto, Canada; ^4^Centre for Education Research and Innovation, Schulich School of Medicine and Dentistry, Western University, London, Canada

**Introduction:** The practice of critical care is complex. The inability or delayed ability of IMGs to adapt to their new training environment and professional roles at the beginning of their ICU fellowship may compromise trainee education and patient care [1]. We explored how the composition and processes of the interdisciplinary ICU team affects this transition.

**Methods:** We conducted a qualitative single centered study based on 23 individual interviews with 16 IMG fellows enrolled in the Adult Critical Care Fellowship Program at the University of Toronto. Fellow’s perceptions of their role and transition within the interdisciplinary ICU team were explored. We used a constructivist grounded theory approach based on purposive and theoretical sampling, inductive thematic coding, and constant comparison to build an iterative, interdisciplinary theoretical understanding of IMG fellows’ transition into ICU teams.

**Results:** Our analysis revealed two main challenges related to the interdisciplinary team: 1) the preponderant role of different team members (e.g. nurses and respiratory therapists), and, 2) the elaborated orchestration of rounds. Early in their fellowship, IMGs had a limited understanding of their role within the interdisciplinary team. As a result, fellows reported experiencing many professional losses regarding their skills, autonomy, appreciation, efficiency, and personal work standards. Combined, these losses threatened fellows’ self-confidence. IMGs negotiated this period by relying on various strategies: trusting honed clinical skills, building trust with certain team members, changing own attitudes towards teamwork, or seeking social support.

**Conclusions:** The interdisciplinary ICU team plays an important role in IMG fellows’ transition and the unique composition and processes of the ICU team are instrumental in the dynamic process of collapse and re-construction of IMG fellows professional identity early in their fellowship.

**Reference**

(1) Wheelan S et al.; J Crit Care, 12: 1–9, 2004

### P426 Discharging patients directly home from critical care: a clinical audit study

#### A Chubb, K Abernethy, J Meyer, P Parulekar

##### St Thomas´ Hospital, London, United Kingdom

**Introduction:** An increasing number of patients are being discharged directly home from critical care units and this is currently viewed as a negative quality indicator [1]. The purpose of this audit was to characterise a cohort of patients who can be safely discharged directly home from adult critical care at St Thomas´ Hospital (STH).

**Methods:** Retrospective observational study of two groups of patients; 1) those discharged directly home from critical care, 2) those discharged within two days of step down to a ward from critical care (admissions 1st June-31st October 2017). The clinical notes of these patients were reviewed via online systems.

**Results:** Baseline demographics of the 58 patients in Group 1 and 90 patients in Group 2 were similar (mean age of 51 years, versus 46 years, p=0.34); average length of stay in critical care was also similar (2.9 days versus 2.5 days respectively p= 0.72). In Group 1, 24 of 168 ICU days were after considered fit for step down versus 40 of 222 days in Group 2, p=0.32 (Fig 1, 2). In Group 1, drug related presentations were more common (27% versus 13% p=0.03), fewer patients had specialist follow up post discharge (36% versus 87%, p<0.001). In Group 1, 7 patients (12%) were readmitted within 28 days, 3 to critical care. In Group 2, 7 patients (8%) were readmitted, 1 to critical care (p=0.38 and 0.14 respectively); none of these readmissions were felt to have been preventable.

**Conclusions:** There is a cohort of patients suitable for discharge directly home from critical care who did not spend significantly longer in ICU awaiting discharge than those who were stepped down to the ward. Identifying these patients early, potentially by their diagnosis, and creating a pathway including access to specialist follow up clinic could allow prompt discharge directly from critical care, thus improving patient satisfaction and reducing hospital-acquired morbidity healthcare costs [1].

**Reference**

1. Lau V et al. J Intensive Care Med 33:121-127, 2016

**Fig. 1 (abstract P426).**
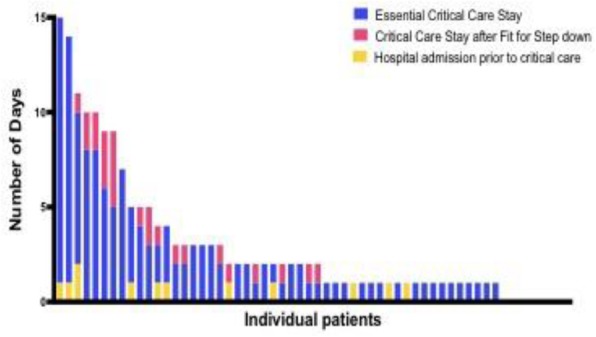
Patient journey of Group 1: those patients discharged directly home from critical care

**Fig. 2 (abstract P426).**
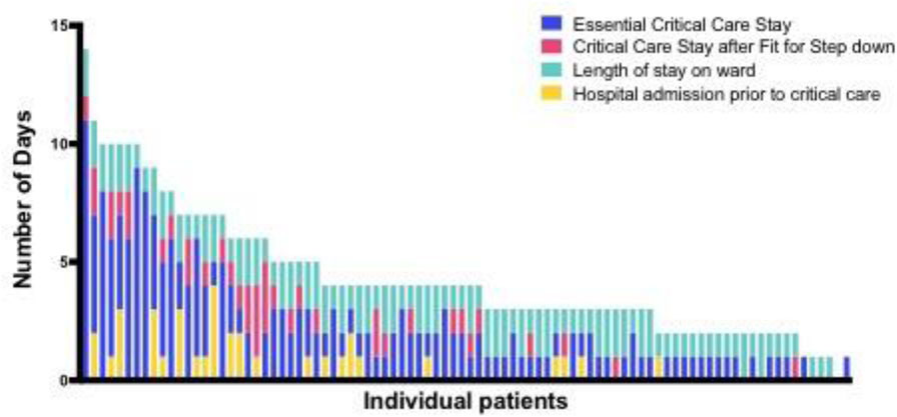
Patient journey of Group 2: those patients discharged home 2 days after step down from critical care

### P427 Discharge decision ICU: what is the role of situation awareness?

#### J Oostrom^1^, P Hoegen^2^, R So^1^

##### ^1^Albert Schweitzer Hospital, Intensive Care Unit, Dordrecht, Netherlands; ^2^Academy of Part-time Studies, School of Health and Social care, Avans University of Applied Science, Breda, Netherlands

**Introduction:** This research aims to explore the role of situation awareness in the decision-making of patient discharge from the Intensive Care Unit (ICU). The discharge of these patients is a complex and, moreover, a challenging transition of care. Readmissions are undesirable given the association with a more extended hospital stay and a possible chance of higher mortality. Little is known on how the decision-making process takes place and accordingly, the role of situation awareness of patient discharge from the ICU. In order to improve the quality of care of patient discharge from the ICU, further research is necessary.

**Methods:** This research concerns a qualitative study in which various health care providers, working in an ICU adults of a large teaching hospital, were interviewed. Through purposive sampling, six nurses, two physician assistants, two intensivists and a physiotherapist were included. On the obtained data a thematic analysis was applied, based on the principles of the Grounded Theory.

**Results:** The discharge decision of ICU patients seems mainly based on the team´s situation awareness, with the initiating role of the intensivist and the guiding role of the nurse. Furthermore, there is an additional role for the physician-assistant and a consultative role for physiotherapy in the process of the decision-making. Worries of patients and family seem not to affect the decision-making directly. In the decision-making process, the well-being of the patients and the possibility to provide the most suitable and best possible care were central. Organizational factors, such as an urgent demand for ICU beds do count but seem not to push the decision to transfer patients from the ICU to the regular hospital ward.

**Conclusions:** The decision to dismiss ICU patients is a complex process with different disciplines and a variety of factors involved. Obtained knowledge and insights into the role of situation awareness provide starting points for improving the quality of the discharge process of ICU patients.

### P428 Resource utilization among elderly ICU patients

#### E Moreira^1^, A Carámbula^1^, M Barbato^1^, P Alzugaray^2^, A Cebey^3^, J Baraibar^4^, C Pan^4^, D Gonzalez^5^, G Burghi^1^

##### ^1^Hospital Maciel, Intensive Care Unit, Montevideo, Uruguay; ^2^Sanatorio Americano, Intensive Care Unit, Montevideo, Uruguay; ^3^COMERO, Intensive Care Unit, Rocha, Uruguay; ^4^COMECA, Intensive Care Unit, Canelones, Uruguay; ^5^CAMOC, Intensive Care Unit, Carmelo, Uruguay

**Introduction:** In the last decades elderly patients are more frequently admitted to intensive care units (ICU). Although older patients have a poorer prognosis than younger patients, little is known about the use of resources in this population. The purpose of this study was to compare the outcome and intensity of treatment of youngest patients (18-79 years old) versus oldest (≥80 years old).

**Methods:** Patients from 6 mixed Uruguayan ICUs were included. ICUs demographic data, SAPS 3 score, resource utlization, clinical and outcome data were retrieved from an electronic ICU quality registry (Epimed Monitor System). Severity was evaluated by using SAPS III leaving out age points.

**Results:** We analyse 4020 patients, of them 11.5% (464) were older than 80 years old. Older population had a higher SAPS III leaving out age points [ 30 (18.25-44) vs 25 (13-39), p<0.001] and a worse ICU mortality (29% vs 17.8%, p<0.001). Despite respiratory failure in the first 24 hours was equal in both groups (28% in more than 80 years old vs 24% in youngers, p=0.11), invasive mechanical ventlation was less used in the elderly population (37 vs 41%%, p=0.01). Among older population, the use of central venous catheter (50% vs 56%, p=0.01) and arterial catheter (22% vs 36%, p<0.001) during ICU stay was less frequent.

**Conclusions:** Despite the fact that older people was more severe illnes, and similar frequency of respiratory failure, the use of mechanical ventilation, the use of central venous catheter and arterial catheter was less frequent.

### P429 The addition of a simulation fellow within the intensive care team and introduction of in situ simulation

#### N Bhalla, D Hepburn, G Phillips

##### Royal Gwent Hospital, Intensive Care Unit, Newport, United Kingdom

**Introduction:** Traditionally, simulation based medical education has been carried out in off site simulation centres, however, we trialled the addition of a simulation fellow, within our intensive care team, to run an in situ simulation (ISS) program on our intensive care unit over a 3 month period.

**Methods:** Our multi-disciplinary ISS program, led by a simulation fellow, incorporated participants, observers and facilitators including doctors (junior trainees up to consultants of varying medical specialties), nursing staff, healthcare support workers, operating department practitioners, physiotherapists and medical students. We ran simulated emergency scenarios and technical skills sessions. With every scenario, we collected data on participant and observer feedback using the World Health Organisation participant feedback form and conducted a satisfaction survey at the end of our trial period.

**Results:** Our results, highlighted in Table 1, show participants found ISS led by a simulation fellow realistic, well structured and organised. It was useful for testing and understanding our response systems, identifying strengths and gaps and establishing individual roles/functions within emergencies; overall leaving us feeling better prepared for critical care emergencies. From our satisfaction survey, 100% of participants found the simulation fellow a useful addition to the intensive care team and expressed the need for more in situ simulation.

**Conclusions:** The addition of a simulation fellow allowed for numerous disciplines within the critical care team to be involved in challenging emergency scenarios (Fig 1, 2), with the additional realism of being on the intensive care unit playing the role they would in real life; as well as having opportunity for spontaneous discussion and learning. From this they reported great benefit and satisfaction. Following our initial success with this program, we plan to have a simulation fellow as an ongoing role within our critical care team.

**Table 1 (abstract P429).** Results from the WHO participant feedback form (Likart scale from 1 to 5 (with 5 being strongly agree and 1 being do not agree) for the following statements: 1.) The exercise was well structured and organised. 2.) The scenario was realistic. 3.) The briefing before the exercise was useful and prepared me for the exercise. 4.) The exercise allowed us to test our response plans and systems. 5.) The exercise improved my understanding of my role and function during an emergency response. 6.) The exercise helped me to identify some of my strengths as well as some of the gaps in my understanding of response systems, plans and procedures. 7.) At the end of the exercise, I think we are better prepared for a health emergency

Statement	Average Score	Number of Responses
1	4.6	39
2	4.5	39
3	4.3	36
4	4.5	39
5	4.4	39
6	4.6	36
7	4.5	39

**Fig. 1 (abstract P429).**
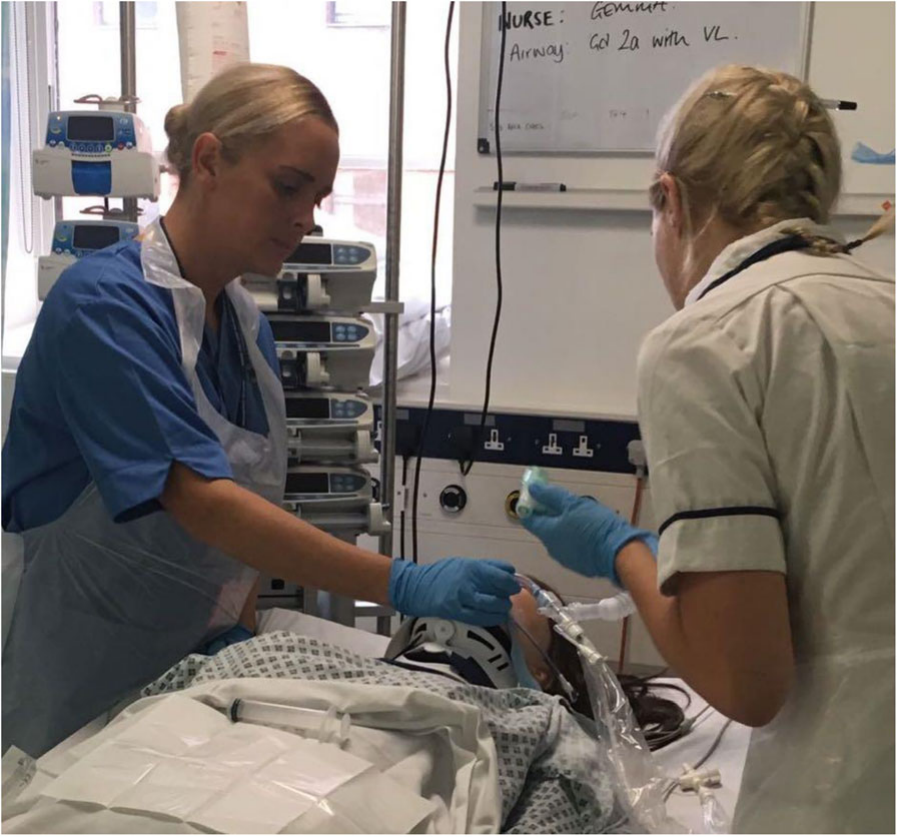
Experienced Critical Care Nurse and Junior Physiotherapist conducting non-directed bronchoalveolar lavage leading to a dislodged endotracheal tube. Picture reproduced with permission from the subjects

**Fig. 2 (abstract P429).**
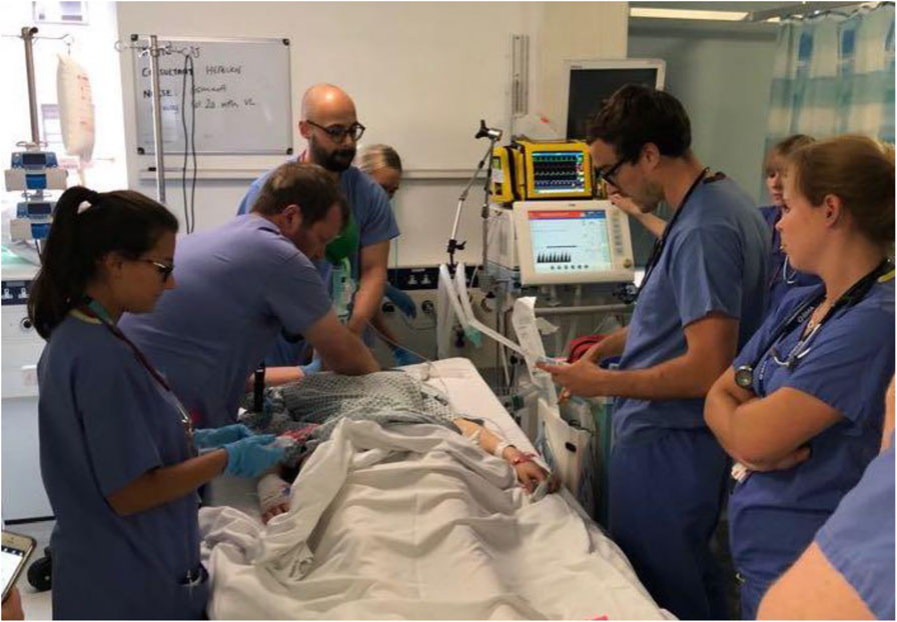
Call for help and response to conduct re-Intubation of a dislodged endotracheal tube. Picture reproduced with permission from the subjects

### P430 Impact of multidisciplinary team in readmission in a Brazilian cardiac intensive care unit

#### C Bosso^1^, P Ribeiro^2^, S Silva^3^, R Caetano^4^, A Cardoso^1^, M Ribeiro^3^

##### ^1^Instituto do coração de Presidente Prudente, Cardiology, Presidente Prudente, Brazil; ^2^Universidade do Oeste Paulista - UNOESTE, UNOESTE, Presidente Prudente, Brazil; ^3^Universidade do Oeste Paulista - UNOESTE, Presidente Prudente, Brazil; ^4^Santa Casa de Misericórdia de Presidente Prudente, Cardiology, Presidente Prudente, Brazil

**Introduction:** The aim of this study is to determine the importance of the multidisciplinary team at readmission rates in a cardiac intensive care unit (CICU).

**Methods:** Retrospective study with analysis of 2945 patients in a CICU of a medium size Brazilian hospital. The years of 2012 and 2013 represent the reduced team (physician, nurse and physiotherapist) and 2015 and 2016 the complete multidisciplinary team (additional presence of phonoaudiologist, psychologist, pharmacist, dentist and nutritional professional). The risk of mortality was determined by SAPS3 score. In order to compare the teams, it was utilized Odd ratio of a logistical sample to the discrete data, and T-Student test to the continuous data. The data analysis was executed from the software RStudio (1.1.456), and the significance level adopted was 5%.

**Results:** The number of patients was of N=2945 (1422 from the reduced team and 1523 from the multidisciplinary team). The age, sex and BMI didn`t present significant difference between groups. The average age of the sample was 68±13 years old (p=0.13). The male sex represented 58% (p=0.93), and the BMI was around 29.2±23.3 (p=0.12). The main diagnoses were similar in both groups - coronary angiography with stent (16%), unstable angina and non ST elevation myocardial infarction (9%). Table 1 shows the average, standard deviation, p-value to T-Student test to SAPS 3 score and lengh of stay (days), according to both reduced and multidisciplinary teams. Table 2 exposes the mortality rate and readmission for both teams. The figure shows the Odds ratio and its IC 95% to the comparison of the mortality, readmission, 24 hours readmission and 48 hours readmission rates between the teams.

**Conclusions:** The multidisciplinary team performance reduced the number of hospital readmissions in 24 and 48 hours in a CICU.

**Table 1 (abstract P430).** Average, standard deviation and p-value for the T-Student test to the SAPS Points and the staying time (days), according to reduced and multi-disciplinary teams

Variable	Group	Mean ± standard deviation	p-value (T-Student)
SAPS 3 Points	Reduced Team / Multi-disciplinary team	39.9 ± 13.1 / 42.8 ± 12.7	<0.001
Lengh of stay (days)	Reduced Team / Multi-disciplinary team	3.4±4.2 / 3.6±5.3	0.11

**Table 2 (abstract P430).** Mortality and readmission rates, according to the reduced and multi-disciplinary teams

Variable	Group	%	OR [CI 95%]	p-value
Mortality rate	Reduced Team / Multi-disciplinary team	10 / 11	1.05 [0.83; 1.33]	0.71
Readmission	Reduced Team / Multi-disciplinary team	18.9 / 9.8	0.47 [0.38; 0.58]	<0.001
24h Readmission	Reduced Team / Multi-disciplinary team	0.63 / 0.07	0.1 [0.01; 0.82]	0.019
48h Readmission	Reduced Team / Multi-disciplinary team	1.27 / 0.33	0.26 [0.09; 0.69]	0.007

**Fig. 1 (abstract P430).**
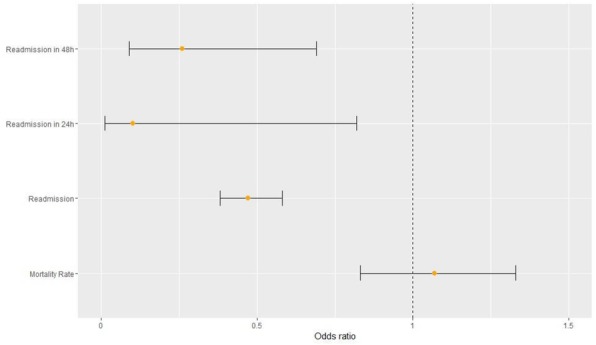
Odds ratio and IC 95% to the comparison of mortality, readmission, 24h readmission and 48h readmission rates between the teams

### P431 The evaluation of the usability of a critical care information system (CCIS)

#### R West, D Hadfield, T Lalmahomed

##### Kings College Hospital, London, United Kingdom

**Introduction:** Critical Care Information Systems (CCIS) support clinical processes by storing and managing data, but poor usability can lead to staff dissatisfaction and increased workload, promoting workarounds that may compromise patient safety [1]. The purpose of the study was to evaluate the usability of a Philips IntelliSpace Critical Care and Anaesthesia (ICCA) CCIS, recently implemented in 51 beds across three critical care units of a large UK teaching hospital.

**Methods:** A prospective, mixed method observational study conducted in May 2018, comprising of (1) an audit assessing the ease of linking bedside devices to ICCA, (2) an audit assessing the usability of co-signing medications in ICCA compared with a non-ICCA paper unit, (3) an online survey, distributed via email to all ICCA unit nurses (n=291) assessing the perceived usability of ICCA. Combined audit and survey results are presented below.

**Results:** 85 surveys were completed (response rate 29%). 28 bedside devices were in use. Automatic charting was the most useful function (35%, n=85), but poor usability of hardware compromised connectivity (57%, n=28) and linking devices the biggest challenge (20%, n=85) (Figure 1). Poor compliance with co-signing in ICCA (66%, n=161) compared to paper (93%, n=183) (Figure 2) and the reported difficulty in co-signing (8%, n=85) reveals significant usability concerns and potential safety issues. 40% (n=85) found ICCA intuitive, though 16% (n=85) found navigating the interface difficult and reported concerns with losing saved work (19%, n=85).

**Conclusions:** This study highlights important usability issues that may impact staff satisfaction, workload and potentially patient safety. Specific recommendations include: (1) a bedside guide to aid device connectivity; (2) a redesign of the co-sign function in collaboration with the manufacturer; and (3) specialist ICCA staff to provide an increased clinical presence and bedside training.

**Reference**

[1] Hudson D et al. Int J Med Informatics 112, 131-136, 2018

**Fig. 1 (abstract P431).**
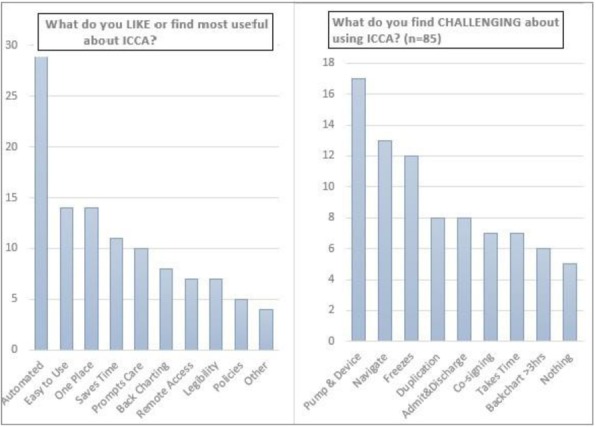
Responses to question 1 & 2 of the usability survey (n=85)

**Fig. 2 (abstract P431).**
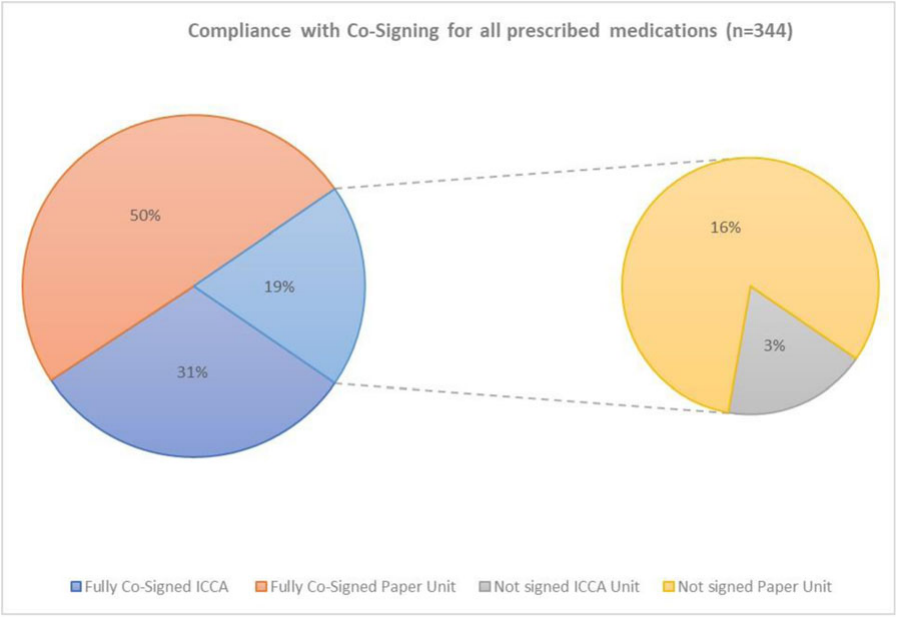
Compliance with co-signing for all prescribed medications (n=344)

### P432 The environmental and cost saving impact of changes to waste management in ITU/HDU

#### W Buxton, J Saffin, B Rose

##### Lewisham Hospital, ITU, London, United Kingdom

**Introduction:** Hypothesis: not all waste in ITU/HDU is clinical waste. By splitting waste into its components substantial cost and environmental savings may be achieved. Hospital waste falls under 3 headline categories [1]:

- Clinical - £510/ ton to incinerate- large environmental impact.
- Domestic £110/ ton to dispose of- landfill
- Recyclable £75/ ton

**Methods:** During the initial audit 24 hours’ worth of waste from one ITU bed was manually divided into the categories above.

**Results:** Based on these figures it was estimated that a saving of £1745 per year would be made (£87.25 per bed space) over the course of a year should domestic waste bins be placed across the 20 bed ICU/HDU. A business case was made, and every bay had a domestic waste bin installed with poster signs for explanation.The re-audit in which all domestic waste across the unit was weighed produced an even greater figure of a saving of £205 per bed space (£4100) per year.

**Conclusions:** Introducing a domestic waste bin may save approximately £200 per year per bed. In a typical ITU such as Lewisham (10 ITU beds/10 HDU beds) that may mean a saving of £4000 per year (with 100% capacity). There are also environmental benefits, burning of plastics releases harmful dioxins. The authors wish to make intensive care units and indeed all areas of the hospital aware of the cost and environmental impact associated with disposing of waste in incorrect categories. We hope that our quality improvement project demonstrates how easily money may be saved and environmental footprint reduced.

**Reference**

[1] https://www.rcn.org.uk/-/media/royal-college-of-nursing/documents/publications/2011/april/pub-004108.pdf

### P433 Association between resilience and level of experience in intensive care doctors in India

#### J Gopaldas, A Siyal

##### Manipal Hospital, Bangalore, Critical Care Medicine, Bangalore, India

**Introduction:** Attrition of doctors in intensive care unit (ICU) is one of the highest amongst all medical specialities globally, and is strongly associated with stress and Burn Out Syndrome (BOS). Factors that contribute to BOS are low pre-morbid resilience and low level of ICU experience. Studies from India have shown high levels of stress in intensive care doctors (>40%), but there are no published studies measuring pre-morbid resilience and risk of burnout in relation to years of experience amongst ICU doctors. Our main aim was to measure cross sectional resilience levels in ICU doctors compared between those with less than 5 years of experience to those with 5 years or more. A secondary aim was to assess the impact of other factors that may contribute to low scores.

**Methods:** An anonymised survey was conducted involving 125 doctors in ICUs across 6 different states in India, using the Connor-Davidson Resilience Scale (CD-RISC 25), which is validated in Indian population.

**Results:** A statistically significant correlation was found between low levels of resilience in ICU doctors with under 5 years of experience (60% of study population with p: 0.0018). A further association of low scores was found in doctors doing night shifts, in which there was an over representation of doctors with less than 5 years of experience. Other factors, such as gender, workload, exposure to end of life care, type of ICU (open/closed), trainee status, did not show a significant association to levels of resilience.

**Conclusions:** Our study shows that interventions that focus on improving resilience should target doctors with low level of experience and those doing night shifts, to gain maximum reduction in burnout and attrition.

### P434 Clinical characteristics of extremely elderly patients in a Brazilian cardiac intensive care unit

#### C Bosso^1^, P Ribeiro^2^, R Caetano^3^, M Valerio^2^, A Cardoso^1^, S Silva^2^

##### ^1^Instituto do coração de Presidente Prudente, Cardiology, Presidente Prudente, Brazil; ^2^Universidade do Oeste Paulista - UNOESTE, Presidente Prudente, Brazil; ^3^Santa Casa de Misericórdia de Presidente Prudente, Presidente Prudente, Brazil

**Introduction:** This study aims to evaluate the epidemiological characteristics and clinical outcomes of extremely elderly patients admitted at the cardiac intensive care unit (CICU).

**Methods:** The database from a Brazilian CICU was used to analyse 3715 admissions, from January 2013 to December 2017. N = 3634 in the group under 90 years and N = 81 over 90 years (including two patients over 100 years). Data analysis was performed using RStudio software (1.1.456), and the significance level adopted was 5%. A logistic regression model was used to test the difference between the mortality and readmission rates in <90 and ≥ 90 groups, which enabled the calculation of odds ratios. Chi-square test was used to evaluate categorical variables and T-Student test to some quantitative variables. The ROC curve was constructed to verify the sensitivity of prediction of mortality through different SAPS 3 scores.

**Results:** Among the <90 and ≥ 90 groups, respectively 59% and 37% was male (p = 0.0001). Mean weight of the> 90 years was 76 ± 16 kg and <90 years was 61 ± 11 (p <0.0001). Odds values indicated a significant difference only for the mortality rate, which was more than double among ≥ 90. Readmissions in any time, 24h and 48h as well the mortality is shown in Table 1 and odds in Figure 1. There was a significant difference in SAPS 3 points between groups (Table 2). The ≥ 90 group presented an average of 10 points higher on the severity scale when compared with those in the <90 group. There was no significant difference in lengh of stay. The highest amount provided by SAPS3 scores was 63% and a specificity of 86% for hospital mortality not group <90 years. In ≥ 90 group the highest sensitivity was 53% and the specificity was 84%. ROC curve for SAPS3 is shown in Figure 2.

**Conclusions:** The extremely elderly patients of a CICU is more severe, with higher mortality and have the same lengh of stay and readmission rates.

**Table 1 (abstract P434).** Mortality rate and rehospitalization according to groups <90 years and ≥ 90 years

Variable	Group	%	OR [CI 95%]	p-value
Mortality rate	< 90 years / ≥90 years	10 / 23	2.62 [1.55; 4.42]	0.0003
Readmission	< 90 years / ≥ 90 years	15 / 9	0.52 [0.24; 1.14]	0.16
24h Readmission	< 90 years / ≥90 years	0.2 / 0	0 [0;0]	0.991
48h Readmission	< 90 years /≥90 years	0.61 / 1.23	2.05 [0.27; 15.41]	0.48

**Table 2 (abstract P434).** Average, standard deviation and p-value for T-Student test to SAPS Points and lengh of stay (days), according to groups <90 years and ≥90 years

Variable	Group	Mean ± standard deviation	p-value (T-Student)
SAPS 3 Points	< 90 years / ≥ 90 years	42 ± 13.2 / 52 ± 11.8	<0.001
SAPS 3 Death Probability	90 years / ≥ 90 years	13.1 ± 15.7 / 23.1 ± 18	<0.001
Lengh of stay	90 years / ≥ 90 years	3.5±5 / 3.1±3.6	0.39

**Fig. 1 (abstract P434).**
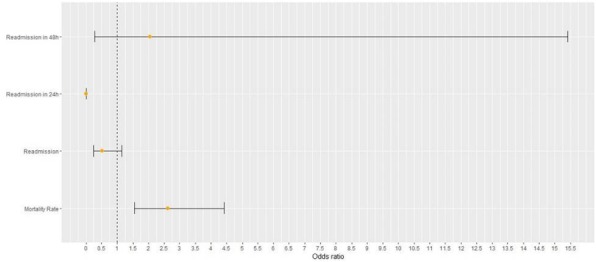
Odds ratio and IC 95% to the comparison of Mortality, Readmission, 24h Readmission and 48h Readmission rates between to groups <90 years and ≥ 90 years

**Fig. 2 (abstract P434).**
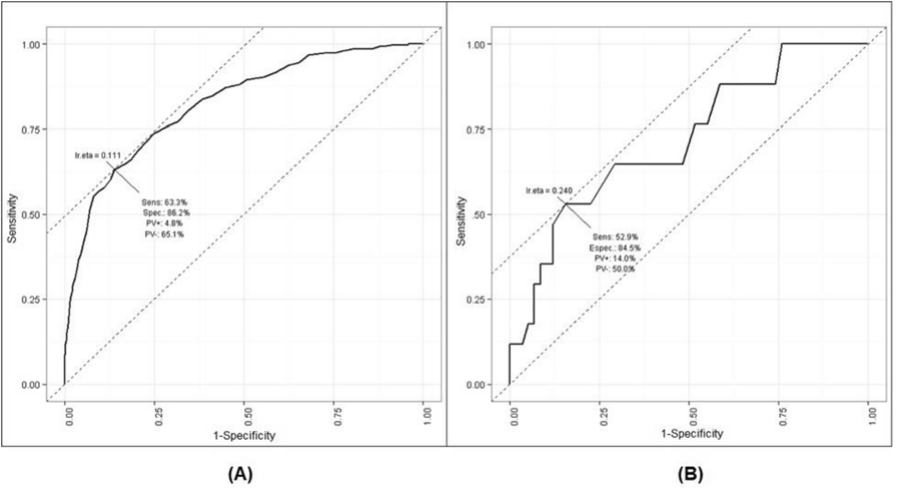
SAPS 3 ROC curve as predictor of hospital mortality in the group: (A) <90 years and (B) ≥ 90 years

### P435 What is the prevalence and impact of non-urgent interruptions within a critical care environment and how can these be minimised?

#### E Castren, S Hutchinson

##### Norfolk & Norwich University Hospital, Critical Care Complex, Norwich, United Kingdom

**Introduction:** The purpose was to assess the prevalence and impact of non-urgent interruptions (NUI) within critical care (CC).A root cause analysis of a never event in our CC discussed NUI as a contributory factor, paralleled by learning from serious incidents.The negative impact of NUI is well evidenced, resulting in delayed task completion, increased stress, and affecting patient safety.

**Methods:** Any NUI during a consultant ward round (CWR) or invasive procedure (IP), not relating directly to the current clinical episode, was included. Qualitative data was collected by a survey, assessing the CC multidisciplinary teams(MDT) perception of NUI.

**Results:** One third of reviews during the CWR, and 40%of IPs, had a NUI. Adverse effects included prescription omissions, delayed CWR, near-miss with a CVC, and failed PICC insertion. Overall, 86% of staff considered NUI a problem; 95% had experienced NUI that led to distraction in train of thought. 45% felt that NUI had led to an error: 83% of doctors, versus 20% of nurses. 86% overall felt NUI contributed to stress at work. Reasons for interruptions included: feeling overloaded, needing to resolve concerns before forgetting/being distracted, unable to prioritise, and to shift responsibility.Lack of leadership or clinical supervision providing a point of contact for problems during shifts was mentioned as contributory. Senior staff raised that whilst attempts have been made to level hierarchy, allowing a voice for all to express concerns contributes to interruptions. Potential solutions included awareness on impact of NUI, jobs book, ´sterile cockpit´ during IPs, and increased clinical supervision during shifts.

**Conclusions:** We have demonstrated the prevalence and consequences of NUI within CC is significant.The impact on staff is significant, both for contribution to errors and also the negative impact on stress in the workplace. Identified potential solution will be implemented.

### P436 The impact of an education package on the knowledge, skills and self-rated confidence of medical and nursing staff managing airway & tracheostomy/laryngectomy emergencies in critical care

#### L O´Connor^1^, K Rimmer^2^, C Welsh^1^

##### ^1^Sunderland Royal Hospital, Anaesthesia & Intensive Care Medicine, Sunderland, United Kingdom; ^2^Royal Victoria Infirmary, Anaesthesia, Newcastle upon Tyne, United Kingdom

**Introduction:** Airway emergencies (AE) in critical care can incur a significant morbidity & mortality [1]. Medical & nursing staff undergo airway & tracheostomy/laryngectomy emergency management (T/LEM) training early in their career, but this is often unconsolidated. In 2017 a course was introduced to support the continued professional development of staff working in Sunderland Royal Hospital Integrated Critical Care unit.

**Methods:** The course comprises of pre-course reading, lectures, a front of neck access (FONA) workshop, orientation to equipment & high fidelity simulation. After the course candidates complete a questionnaire evaluating their prior knowledge of the UK Difficult Airway Society(DAS) & National Tracheostomy Patient Safety(NTPS) guidelines, personal experience of AE & T/LE, & self-rated confidence.

**Results:** Nursing staff reported lower rates of formal education in AE (27%) & T/LEM (4%) compared with the medical candidates & fewer had been directly involved in an emergency. Prior to the course 73% of staff were aware of the latest DAS guidelines & 46% of the NTPS. Overall, 89% of candidates were very satisfied with the course, & 30% of medical & 59% of nursing staff reported an increase in self-rated confidence score by 1 point, & 32% & 28% by 2+ points from their baseline.

**Conclusions:** Introduction of the course improved the awareness of contemporary guidelines, self-rated confidence in the event of encountering an airway or T/LE, equipment familiarity & performing FONA in the event of failure to oxygenate during intubation on critical care. This illustrates a compact, comprehensive education programme is an efficient & pragmatic way of delivering regular updates for critical care staff.

**Reference**

[1] Cook T et al. 4th National Audit Project of The Royal College of Anaesthetists and The Difficult Airway Society. Major complications of airway management. Available from: https://www.rcoa.ac.uk/system/files/CSQ-NAP4-Full.pdf

### P437 The development of a framework for improvement of intensive care delivery: a systemic intervention

#### J Scribante^1^, T Andrew^2^, S Bhagwanjee^3^, A Van Nieuwkerk^1^

##### ^1^University of the Witwatersrand, Department of Anaesthesiology, Johannesburg, South Africa; ^2^Durban University of Technology, Enginering and the Build Environment, Durban, South Africa; ^3^University of Washington, Anaesthesiology, Seatle, United States

**Introduction:** The aim of this research was to develop a systemic framework for the improvement of intensive care delivery.

**Methods:** The factors affecting the delivery of intensive care was elucidated by a comprehensive review of the intensive care literature. A further understanding of intensive care delivery in South Africa was obtained by “making sense of the mess” with eight workshops and 20 interviews using a systems approach. Systemic intervention served as the meta-methodology and methods and techniques from interactive planning, critical systems heuristics, soft systems methodology and the viable system model were employed.

**Results:** Making sense of the mess emphasised the complexity of intensive care delivery, on both a situational and a cognitive level. It became clear that a single methodology would not suffice, but that a pluralist methodology was required to guide improvement in intensive care delivery. Based on this understanding, nine principles were formulated to guide the development of a framework. Systemic intervention was again used as the meta-methodology. Interactive planning was identified as the key methodology, incorporating methods and techniques used in the making sense of the mess phase to build a systemic framework for the improvement of intensive care delivery. Embedded in the proposed framework are matters relating to systemicity, complexity, flexibility, empowerment, and transformation of intensive care delivery. The proposed framework allows for multiple-perspectives, including that of marginalised stakeholders, the mitigation of multi-vested interests and power relationships (Fig 1). It is both flexible and adaptable to promote learning about the complex problems of intensive care delivery and it accommodates the strengths of various relevant approaches to complex problem solving.

**Conclusions:** The proposed framework aims to facilitate sustainable improvement of intensive care delivery and to ensure the “just-use” of resources to foster distributive justice.

**Fig. 1 (abstract P437).**
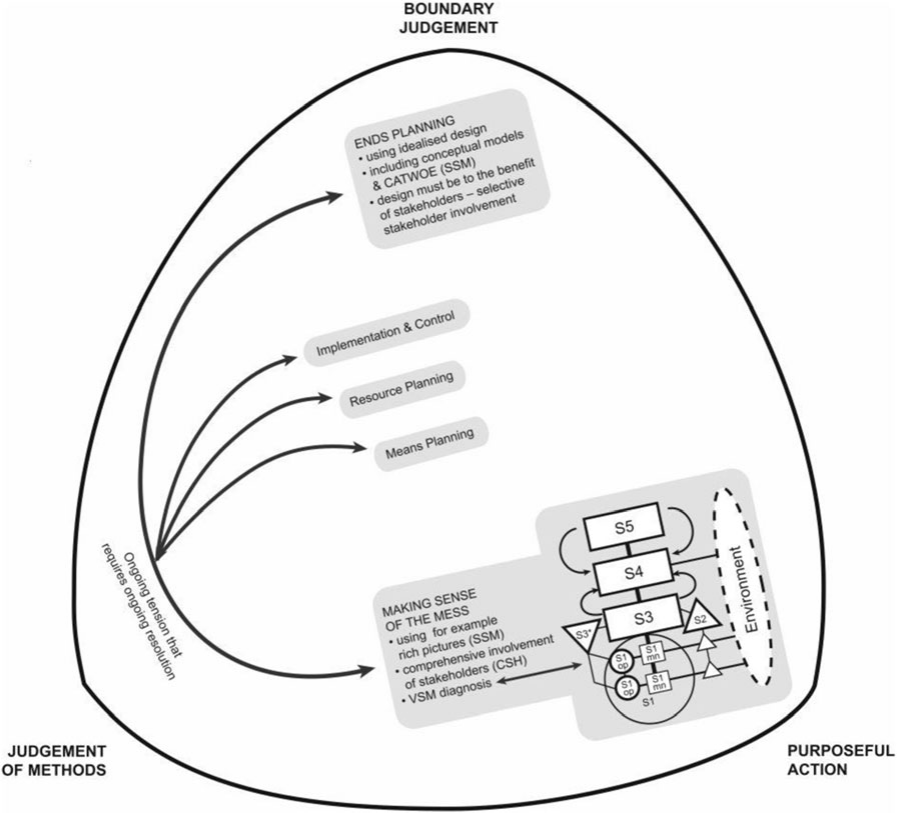
Framework for the improvement of intensive care delivery

### P438 The perioperative management of adult renal transplantation across the United Kingdom: a survey of practice

#### C Morkane^1^, J Fabes^2^, N Banga^3^, P Berry^4^, C Kirwan^5^

##### ^1^Royal Free Hospital, Anaesthetics/ICM, London, United Kingdom; ^2^Royal Free Hospital, Division of Surgery and Interventional Science (University College London) & Royal Free Perioperative Research Group, London, United Kingdom; ^3^Royal Free Hospital, Renal Transplant & Endocrine Surgery, London, United Kingdom; ^4^Royal Free Hospital, London, United Kingdom; ^5^Royal London Hospital, Department of Critical Care Medicine, London, United Kingdom

**Introduction:** There is a limited evidence base to guide perioperative management of patients undergoing renal transplantation and no national consensus in the UK. We developed an electronic survey to provide an overview of UK-wide renal transplant perioperative practice and determine the need for future guidelines on patient management.

**Methods:** A 29-question survey was developed to encompass the entire renal transplant perioperative pathway with input from clinicians with expertise from renal transplant surgery, anaesthesia, nephrology and intensive care. The survey was sent to lead renal anaesthetists at each of the 23 transplant centres across the UK.

**Results:** Twenty-two centres (96%) returned complete responses. There was limited evidence of guideline-based approaches to preoperative work-up, with marked variety in modality of preoperative cardiorespiratory function testing performed. Questions regarding intraoperative fluid management (Fig 1), blood pressure targets and vasopressor administration (Fig 2) identified a broad range of practice. Of note, the routine use of goal-directed fluid therapy based on cardiac-output estimation was reported in six (27%) centres whilst nine centres (41%) continue to target a specific central venous pressure (CVP) intra-operatively. A dedicated renal ward was the most common postoperative destination for renal transplant recipients (62% of centres), whilst a renal or transplant-specific HDU provided postoperative care in 8 (38%) centres. The need for care in an ICU setting was decided on a case-by-case basis.

**Conclusions:** This questionnaire highlighted a high degree of heterogeneity in current UK practice as regards the perioperative management of renal transplant recipients. Development of evidence-based national consensus guidelines to standardise the perioperative care of these patients is recommended.

**Fig. 1 (abstract P438).**
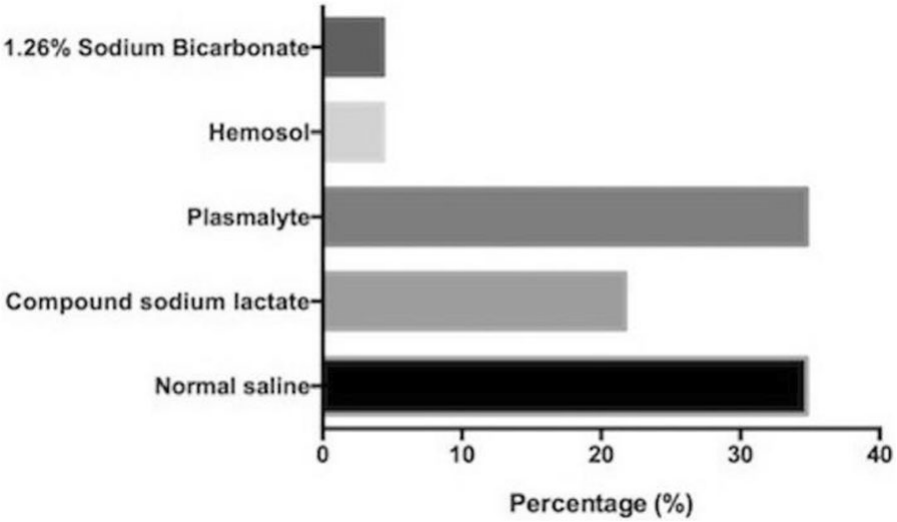
Responses to the question: which intravenous fluid is predominantly used intraoperatively during renal transplantation in your centre?

**Fig. 2 (abstract P438).**
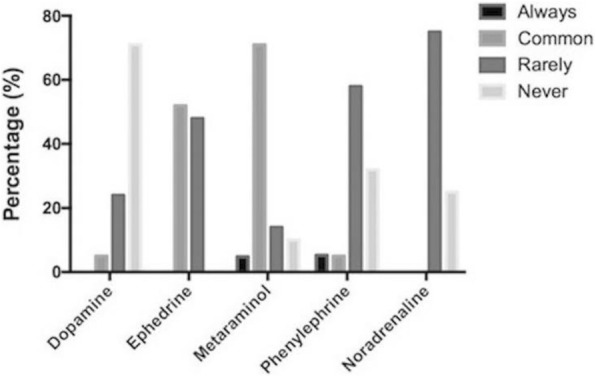
Responses to the question ‘regarding vasoactive drugs, does your centre use the following frequently, rarely or never: dopamine, ephedrine, metaraminol, phenylephrine, noradrenaline’

### P439 Postoperative ICU management after elective surgery. Is there any benefit?

#### G Micha^1^, V Chantziara^1^, F Kaminari^2^, A Vassi^2^, G Katsagani^2^, A Efthymiou^1^

##### ^1^Saint Savvas Hospital, ICU, Athens, Greece; ^2^Saint Savvas Hospital, Athens, Greece

**Introduction:** Postoperative care of high risk patients in the ICU used to be considered the gold standard of care in terms of reducing perioperative mortality [1]. New evidence comes to question this practice [2]. The primary objective of our study was to detect any benefit of postoperative ICU care after elective surgery in terms of patient’s outcome, length of hospital stay, complications and cost.

**Methods:** A 6-month retrospective analysis of high perioperative risk patients who were about to be subjected into an elective operation were included into the study. Subsequently they were allocated into two groups. Group I patients were those admitted into the ICU for postoperative care while those admitted into the standard ward consisted Group II. Demographic data, length of hospital stay, outcome, need of mechanical ventilation, complications and total cost were recorded.

**Results:** A total of 78 patients were recorded, 39 in each Group. There was no statistical difference regarding the demographic data between the two study groups. Seven patients died before hospital discharge (5 in Group I and 3 in Group II, p>0.005). There was no impact of ICU admission on length of hospital stay (p=0.03) which is primarily affected by the need of mechanical ventilation (p=0.04) and reoperation (p<0.05). The total cost and the postoperative cost of hospital care did not statistically differ among study groups.

**Conclusions:** According to our study the need of postoperative care of high risk patients in the ICU is rather questionable in terms of perioperative mortality, length of hospital stay and cost of care.

**References**

1. Pearse RM et al. BMJ 343:d5759

2. Kahan BC, et al. Intensive Care Med. 43:971–979, 2017

### P440 Totally implantable venous access ports (TIVAPs) in cancer patients (pts): safety of implantation in intensive care unit (ICU) and opportunity for immediate use

#### A Pronina, I Kurmukov, S Kashia

##### “N.N. Blokhin National Medical Research Center of Oncology” of the Ministry of Health of the Russian Federation, Moscow, Russia

**Introduction:** TIVAP is a preferred vascular access device for patients with solid tumors and radiological-guided insertion is a standard of care. However, many hospitals have no access to interventional radiology service. Our study aimed to determine whether it is safe to place TIVAPs in ICU for immediate administration of chemotherapy.

**Methods:** We analysed prospectively maintained database of our department and collected data for adult pts with TIVAPs implanted between 02/2008 and 10/2018. The median age was 55 (range 18-82) years, 59% were women. All procedures were performed by 3 trained physicians with experience in ultrasound (US). Puncture technique was used and tip location was controlled with electrocardiographic (ECG) and US with subsequent chest X-ray confirmation. Pts were followed up for at least 30 days after the procedure for complications, functioning of TIVAP and surgical wound healing.

**Results:** All 277 TIVAPs were successfully implanted in 276 pts. Infraclavicular route was used in 231 cases (83.3%). Difficulties with indwelling guide wire were observed in 11 (4.76%) pts but did not precluded implantation. Placement complications included pneumothorax (n = 3), catheter malposition (n = 3) and artery bleeding (n = 1). These complications required additional therapy but were managed successfully and resolved without consequences. In the rest 46 cases internal jugular vein (JV) was used. Complications were not observed. ECG and US navigation provided optimal tip location control in these situations. Surgical wound healed after 10-14 days and chemotherapy initiation did not affect healing. All TIVAPs had adequate functioning 30 days after placement.

**Conclusions:** It is feasible to implant TIVAPs in ICU. These devices can be used on the implantation day without jeopardizing patient safety. JV catheterization seems to be optimal approach and US navigation and ECG are sufficient methods for placement control.

### P441 A review of prescribing & administration errors associated with a critical care informatics system unscheduled downtime in a central London teaching hospital

#### R Mehta^1^, R Sloss^2^, H Shahbhakti^2^, F Master^2^, A Choy^2^, P Hopkins^2^

##### ^1^Kings College Hospital, Pharmacy Department, London, United Kingdom; ^2^Kings College Hospital, London, United Kingdom

**Introduction:** There is increasing use of Clinical Information Systems to improve patient safety and quality of care in Critical Care. With all these systems, a rigorous business continuity access (BCA) plan needs to be in place so patient safety is not compromised [1] and ensure continuity of care. Here we evaluate the types of medication errors that occurred during a period of unscheduled downtime; potential contributory factors [2] and the number of errors involving critical medicines [3] were analysed.

**Methods:** During the unscheduled downtime, all prescribing and administration of medicines were transferred to a paper based system using the patients’ Web Offline Chart (WOC – Philips Healthcare). Pharmacists at the time double checked the paper charts that were transcribed, to mitigate errors but this was not consistent due to the timing of the event. We retrospectively compared the paper drug charts against the electronic prescriptions and noted all errors for 47 patients.

**Results:** In total 26 medication errors were identified & 1 allergy omission (Table 1). Pharmacists double checked 68% of the paper charts.

**Conclusions:** Our data highlights the risks associated with unscheduled electronic patient management system downtime and the heterogeneity of the types of errors & potential contributory factors. It underscores the need for robust local BCA plan implementation, critical review of the WOC document and regular staff training around potential unscheduled system downtime.

**References**

1. Guidelines for Provision for Intensive Care Services (GPICS), Version 2, October 2018 (in draft consultation)

2. Lawton R et al. BMJ Quality & Safety. 21:369-380, 2012

3. Specialist Pharmacy Services. Reducing Harm from omitted and delayed medicines in hospital. A tool to support local implementation. April 2017

**Table 1 (abstract P441).** Medication errors

Error type	Number of errors	Cause & effect	Potential Contributory factors	Number of critical medicines involved
Prescribing errors	11	Wrong frequency, omissions, duplicates, unintentional	Individual, task, workload & staffing issues	1
Prescribing errors	6	No frequency on WOC - omission/duplication of drug	Individual & task	5
Administration errors	5	Scheduled time of dose had passed, no STAT dose	Staff training & local policies & procedures	2
Administartion errors	3	Administration omissions	Individual & task	not applicable
Administration errors	1	Use of supplemental charts, not familiar to nurse	Staff training & education	1

### P442 Transfer of care of medicines in a large teaching hospital – how safe is it really?

#### A Walsh^1^, V Metaxa^2^, R Mehta^3^

##### ^1^Kings College Hospital, King´s Critical Care, London, United Kingdom; ^2^Kings College Hospital, London, United Kingdom; ^3^Kings College Hospital, Pharmacy Department, London, United Kingdom

**Introduction:** The transfer of patient care (TOC) between the intensive care unit (ICU) and hospital ward is associated with a high risk of medical errors [1].According to UK National data between 30-70% of patients have an error or unintentional medication change made when moving between care settings [2]. Currently different prescribing systems without interoperability are used between ICU areas & ward settings in our institution, resulting in medications needing to be re-prescribed on transfer. We aimed to evaluate the time delay in medication re-prescribing, number of unintentional omissions of drug doses and reasons, as well as percentage of critical medicines [3] omitted in the first 24h following discharge.

**Methods:** Over a 2 month period, 79 discharged patients (50% of all discharges) from two ICU units were included. The ICU discharge letter which contained the medication list on transfer was compared against the ward based electronic drug chart to identify all unintentional omitted medication doses during the first 24 hours. The starting time point was when the patient physically left ICU.

**Results:** 13/79 (16%) of patients had their medication prescribed more than 4 hours post discharge. There were a total of 269/1,145 (23%) unintentional omitted doses (Table 1). Of these 104/269 (39%) were considered critical medicines (Table 2).

**Conclusions:** This data confirms the risk associated with TOC especially around medicines. The need of interoperable electronic prescribing systems is one solution and could improve patient safety by streamlining the process.

**References**

1. Buchner D, L et al. BMJ Open.5:e007913, 2015

2. National Institute of Clinical Excellence (NICE). Medicines optimisation: the safe and effective use of medicines. March 2015

3. Specialist Pharmacy Services. Reducing Harm from omitted and delayed medicines. A tool to support local implementation. April 2017

**Table 1 (abstract P442).** Omission reasons

Omission Reasons	Percentage of Omitted Doses
Prescribing omission	52
Medication due before ward drug chart written	35
Incorrect time	6
Medication not available	5
Unable to establish	1
Incorrect dose	1

**Table 2 (abstract P442).** Critical medication omissions

Drug Class	Percentage of Omitted Doses
Antimicrobials	38
Anticoagulants (prophylaxis)	17
Cardiovascular	17
Antiplatelets	7
Corticosteroids	3
Antidiabetics	3
Other	15

### P443 An exploration of the relationships between staff perceptions of safety in adult intensive care (ICU) and measures of safe staffing and workload

#### C Leon-Villapalos, M Wells, SJ Brett

##### Imperial College Healthcare NHS Trust, London, United Kingdom

**Introduction:** Staff perceptions of safety may contribute to workforce stress and be organisationally important [1]. This study explored the feasibility of capturing perceptions of safety with a bedside professional reported (BPR) shift safety score, and explored relationships between BPR and measures of staffing and workload.

**Methods:** UK Health Research Authority approval was obtained (ID249248). Data were collected for 29 consecutive days at Imperial College Healthcare Trust (70 general critical care beds on 3 sites).The BPR asked all ICU staff to rate each shift as “safe, unsafe, or very unsafe”. Responses were described and correlated with data on organisational staffing (care hours per patient day CHPPD) and nursing intensity (total number of organs in failure/ total number of nurses).

**Results:** A total of 2836 BPR scores were recorded (response rate 57%). We noted heterogenous responses between sites and days, and within shifts, only 14 % of shifts were unanimously rated. Whilst 34% of shifts were rated by staff as “unsafe” or “very unsafe”, organisational metrics recorded only 5% as ‘unsafe’. We did not find a correlation between measures of staffing (CHPPD) and perceptions of safety (Figure 1). Preliminary analyses suggest that staff perceptions of safety are not well correlated with nursing intensity (Figure 2), although these numbers commonly inform staffing metrics.

**Conclusions:** Completing the BPR tool was feasible and acceptable to staff. Responses showed variations in perceptions of safety and a gap between organisational metrics and individual perceptions. Factors that commonly drive workforce metrics may not correlate with staff perceptions of safety. The BPR is a pragmatic, staff driven, tool to augment other measures of safety and is applicable to various ICU settings. Further research is needed to explore staff perceptions in order to understand the importance of this organisationally, and for staff stress.

**Reference**

1. Seaman J, JAMA, 320: 1981-1982, 2018

**Fig. 1 (abstract P443).**
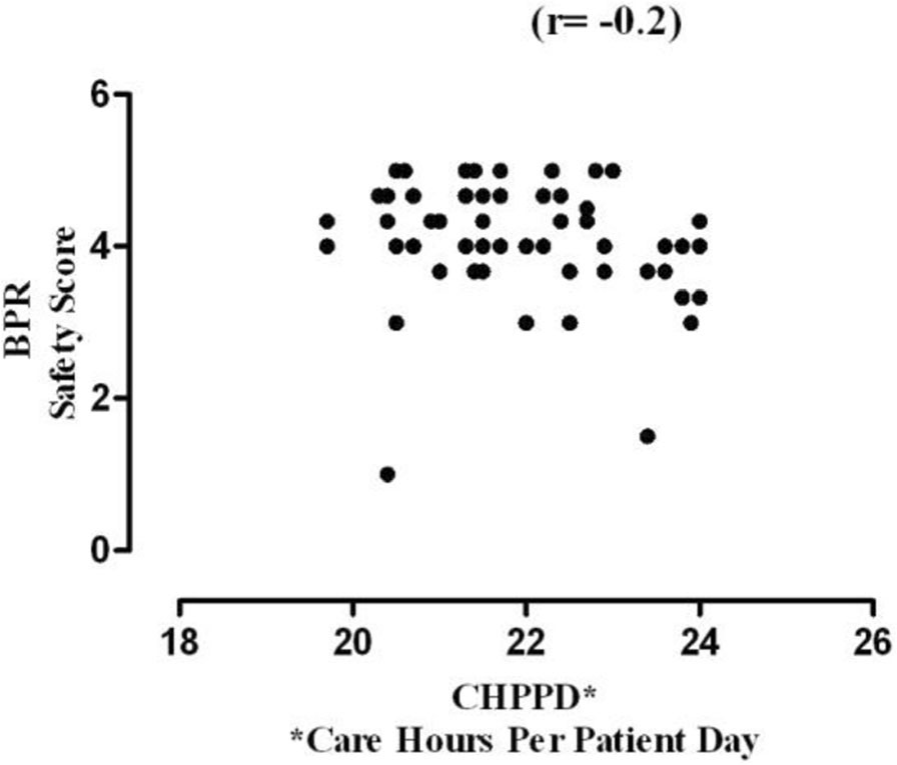
Care hours per patient day and BPR safety score (1 = very unsafe, 5 = safe)

**Fig. 2 (abstract P443).**
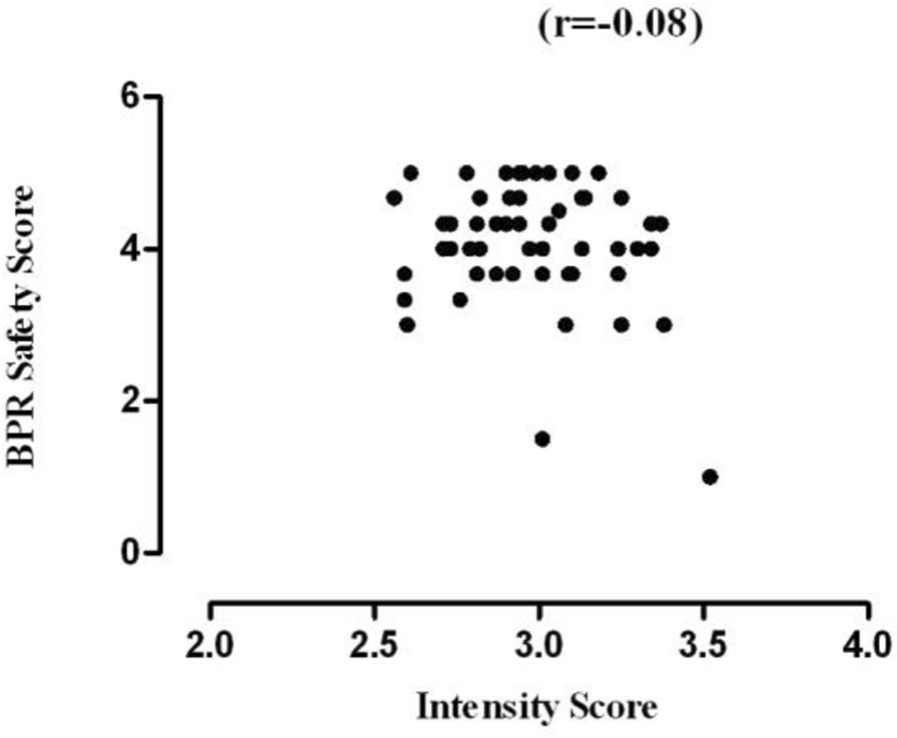
Nursing Intensity (total number of organs in failure/ total number of nurses/ day (2 = low intensity 4 = high intensity) and BPR safety score (1= very unsafe, 5 =safe)

### P444 Understanding the delivery of intensive care in South Africa

#### J Scribante^1^, T Andrew^2^, S Bhagwanjee^3^, A Van Nieuwkerk^4^

##### ^1^University of the Witwatersrand, Department of Anaesthesiology, Johannesburg, South Africa; ^2^Durban University of Technology, Faculty of Enginering and the Built Environment, Durban, South Africa; ^3^University of Washington, Anaesthesiology, Seattle, United States; ^4^University of the Witwatersrand, School of Governance, Johannesburg, South Africa

**Introduction:** Delivery of intensive care (ICU) is complex because of multiple stakeholders with varied perspectives and conflicting goals that interact and are interdependent. To inform the development of a framework for the improvement of ICU delivery in South Africa, it was essential to first understand ICU delivery or “make sense of the mess”.

**Methods:** A systemic approach such as systems thinking is required to holistically explore and understand the complexity of ICU. No methodology is perfect and methodological pluralism as proposed by Systemic Intervention, a systems thinking approach, was used for a more flexible and responsive intervention. The methods used was the making sense of the mess phase of Interactive Planning, stakeholder analysis as describe by Critical Systems Heuristics, rich pictures from Soft Systems Thinking and Viable Systems Model diagnosis. Making sense of the mess was done in 2 phases: first the mess was formulated with rich pictures generated in 8 workshops and 20 interviews. The discussions of the rich pictures by the respective stakeholders were transcribed and analysed using Braun and Clark’s thematic analysis. Secondly, based on the data generated from phase 1 a diagnosis of the viability of the ICU system was made.

**Results:** The data from the 2 phases were very rich and complex and 6 themes emerged (Figure 1). These themes were interdependent and resulted in disorganised ICU delivery with limited opportunities for learning to improve ICU delivery with dichotomies that existed at various levels of ICU. It was a problem to present the complex data in the traditional linear manner due to the interdependence of the themes. The analysis is presented as 6 stories, a known approach in the complexity discipline, where the themes of the analyses are portrayed.

**Conclusions:** The making-sense-of-the-mess phase confirmed the complexity of ICU delivery, at both a situational and a cognitive level and with this understanding a framework for the improvement of ICU delivery could be developed.

**Fig. 1 (abstract P444).**
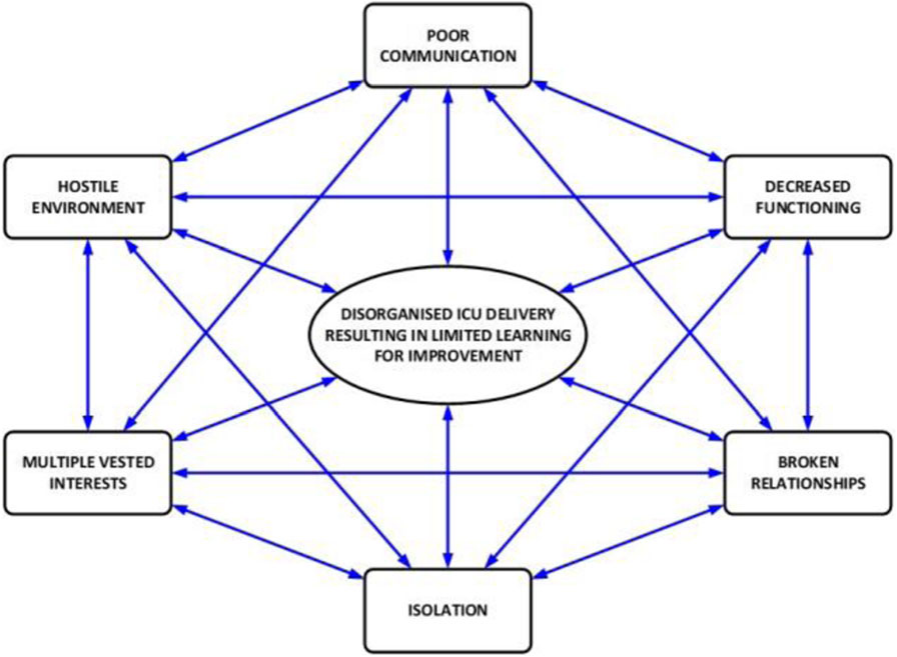
The six themes identified and the resulting disorganised delivery of ICU

### P445 Validating the hierarchy of intervention effectiveness for prescribing nudges in critical care

#### J Warburton^1^, N Jones^2^, R Cooper^2^, P Turner^2^, C Bourdeaux^1^

##### ^1^University Hospitals Bristol NHS Foundation Trust, Critical Care, Bristol, United Kingdom; ^2^University Hospitals Bristol NHS Foundation Trust, Pharmacy, Bristol, United Kingdom

**Introduction:** Improving prescribing practice involves changing prescriber behaviour. Education is assumed to change behaviour but other approaches may be more effective (Figure 1) [1]. Changes to the presentation of information and the configuration of choices have potential to rectify common prescribing errors through subtle ‘nudges’ [2]. The implementation of clinical information systems (CIS), including electronic prescribing, provides an opportunity to deploy strategies such as standard orders, dose limits, and product level prescribing. With an infinite number of configuration options available, clinical leaders need to know which interventions are most effective. We evaluated several of these strategies in a before and after observation study

**Methods:** Interventions, utilising CIS nudges, were chosen to improve four areas of prescribing practice in a tertiary critical care unit using methods matched to the top 4 levels of the hierarchy. Data were collected for 2 months before and after interventions to map changes in compliance with a pre-defined standard except for the standardisation intervention where 4 months’ data were collected due to low prescription numbers. No education on changes was given during the baseline data collection so any change in performance after the go-live date is entirely attributable to the intervention.

**Results:** The change in performance for each level ranks the intervention levels in the order (highest first) forced function, automation and standardisation (Table 1). The use of point of prescribing reminders was not associated with a significant difference in performance.

**Conclusions:** The effectiveness of intervention levels seen in practice is consistent with that of the model. Further studies could be undertaken to strengthen these conclusions but in the meantime the approach to changing practice using CIS nudges should focus on standardisation or above.

**References**

1. Cafazzo JA et al. Healthcare Quarterly 15:24-29, 2012

2. Patel MS et al. NEJM 378:214-216, 2018

**Table 1 (abstract P445).** Before and after performance against defined standards

Level	Compliance	Compliance	Absolute % improvement
	Before	After	(p<0.01)*
Forced Function	7/211	219/221	96
Automation	41/332	294/298	87
Standard.	2/12	11/14	62
Reminder	22/35	18/30	-3

**Fig. 1 (abstract P445).**
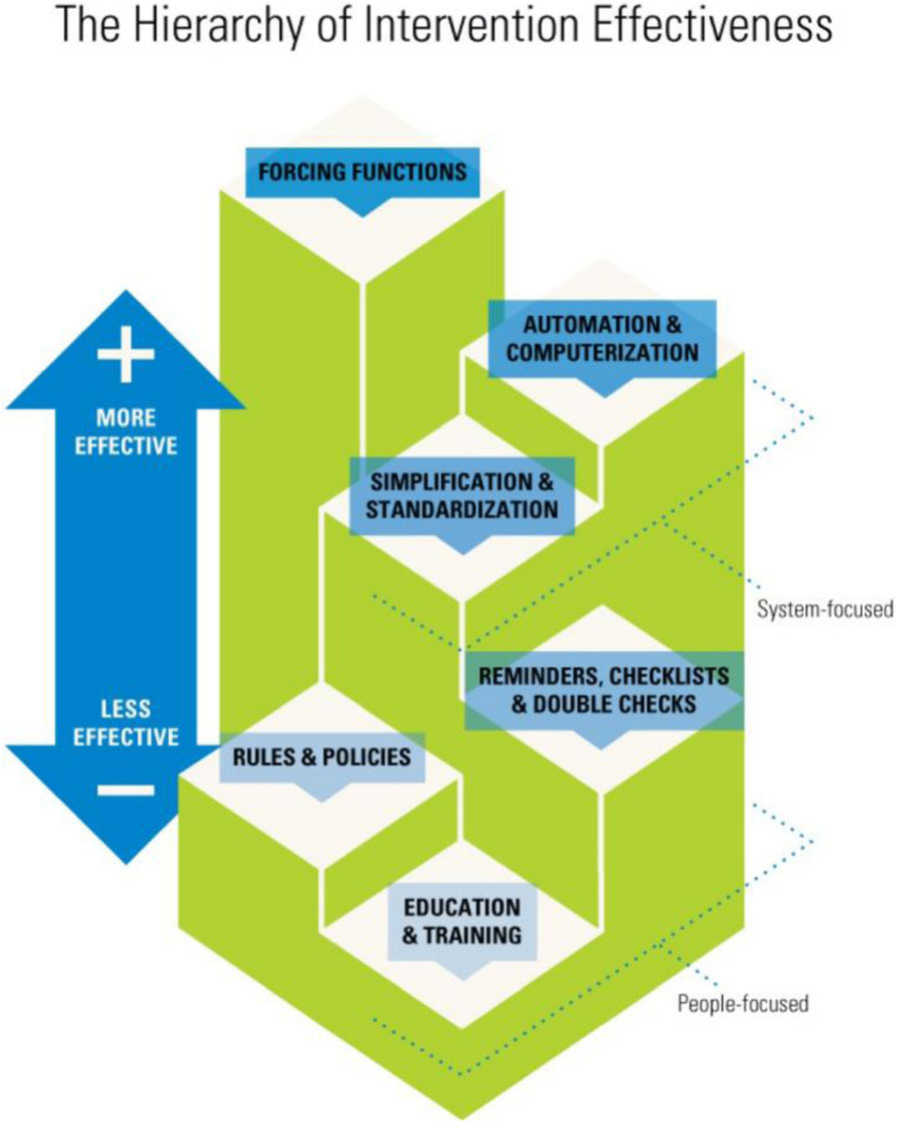
Hierarchy of intervention effectiveness [from 1]

### P446 Understanding sound pressure levels in the intensive care unit

#### J Darbyshire, JD Young, J Bedford, O Redfern, R Hatch

##### University of Oxford, Nuffield Department of Clinical Neurosciences, Oxford, United Kingdom

**Introduction:** Intensive care unit (ICU) sound pressure levels (SPL) are persistently above World Health Organisation recommendations for clinical areas [1]. This may impact patient recovery. Standard SPL monitoring records single values for each 24h period (LAeq24). We hypothesise this reporting rate is unsuitable for ICU.

**Methods:** We measured SPL October 2016 – May 2018, logging frequency (Hz), SPL (dB), and loudness (perception of sound) every second [2]. The resulting dataset was of a size that conventional statistics programs would require computational resources not easily obtainable on standard university commodity hardware. We processed the full dataset without sampling by using distributed task dispatching, parallelism and scheduling of a cluster computing framework (Apache Spark). We created a system consisting of a single workstation (6 cores; 32GB RAM) running Ubuntu 18.04 LTS, Oracle Java 1.8, Apache Spark 2.3, Scala 2.11, R Core 3.5, R Studio 3.5 and sparklyr 0.8.4. We utilised the sparklyr library in R Studio to run arbitrary R code using the dplyr library. We analysed aggregate data in R Core & used ggplot (v3) to create visuals.

**Results:** We achieved more complex analysis than standard SPL reporting with relatively modest computing resources. Specifically we identified lower SPL peaks in the early hours & loudness levels considerably higher than parallel SPL.

**Conclusions:** Simple LAeq24 do not facilitate reflection on practice thus impetus for change is limited. Loudness data highlight the patient experience of SPL in the ICU is more intrusive than LAeq24 indicates due to high sensitivity to sounds ~2–4kHz, a common frequency range for alarms. Higher fidelity increases understanding of SPL which can lead to targeted interventions to reduce patient disturbance.

**References**

[1] Khademi et al, Rev Clin Med 2, 58-64, 2015;

[2] Mueller-Trapet et al, Applied Acoustics, 139, 93-100, 2018

### P447 Effect of checklist for early recognition and treatment of acute illness for morning rounds on the prognosis of critically ill patients

#### C Hou, Y Kang, X Liao

##### West China Hospital, Critical Care Medicine, Chengdu, Sichuan Province, China, China

**Introduction:** To investigate the effects of Checklist for Early Recognition and Treatment of Acute Illness(CERTAIN) from Mayo Clinc on the prognosis of patients in the intensive care unit (ICU) in China [1].

**Methods:** From January 2016 to September 2016, a total of 130 patients were admitted as the control group. Another 75 patients were provided with CERTAIN checklist for morning rounds from April 2017 to November 2017. Ventilator-free duration in ICU, central venous catheter duration, urinary catheter duration, rates of deep vein thrombosis (DVT) and stress ulcer prophylaxis, rates of de-escalation antibiotic therapy, DVT prophylaxis duration, stress ulcer prophylaxis duration, ICU and hospital mortality, 28-day mortality, rate of central venous catheter infection, length of stay in ICU and hospital between two groups were analyzed.

**Results:** Rate and duration of DVT prophylaxis in the intervention group were 81.3% and 3(2,6) days respectively, in the control group were 67.7% and 5(3,10) days, the differences between two groups were statistically significant(P<0.05) (Table 1). There were no differences in ventilator-free duration in ICU, central venous catheter duration, urinary catheter duration, rate of stress ulcer prophylaxis, rates of de-escalation antibiotic therapy, stress ulcer prophylaxis duration, ICU and hospital mortality, 28-day mortality, rate of central venous catheter(CVC) infection, length of stay in ICU and hospital between two groups (Table 2).

**Conclusions:** Electronic checklist in ward rounds can increase the rate of DVT prophylaxis and reduce the duration, but it cannot improve the prognosis of critically ill patients.

**Reference**

1. Duclos G, et al (2018). Anaesth Crit Care Pain Med 37: 25-33

**Table 1 (abstract P447).** Process of care outcomes

Process of care outcomes	Control Group	Intervention Group	OR(95%CI)	P
Ventilator-free days in ICU/(day), mean (SD)	9.15(8.68)	11.54(9.57)	-	0.09
DVT prophylaxis £¬No./total No. of patients (%)	88/130(67.70)	61/75(81.30)	0.48(0.24-0.97)	0.03
DVT prophylaxis duration/(day) M (P25, P75)	5.00(3.00,10.00)	3(2.00,6.00)	-	<0.01
Stress ulcer prophylaxis£¬No./total No. of patients	104/130(80.00)	56/71(84.00)	-	0.845
Stress ulcer prophylaxis duration/(day), mean (SD)	5.48(5.05)	4.27(3.71)	-	0.116
Urinary catheter duration/(day), mean (SD)	7.04(5.70)	7.35(6.12)	-	0.75
CVC duration/(day), mean (SD)	5.29(4.52)	5.32(4.00)	-	0.98

**Table 2 (abstract P447).** Clinical outcomes

Outcomes	Control Group	Intervention Group	OR(95%CI)	P
in-hospital mortality, No. (%)	13(10.00)	4(5.33)	0.51(0.16-1.62)	0.24
CVC infection, No./total No. of patients (%)	1/51(1.10)	1/38(2.60)	1.35(0.08-22.32)	1.00
ICU mortality, No (%)	5(3.80)	1(2.20)	0.40(0.11-1.47)	0.55
28-day mortality, No (%)	24(18.50)	11(14.70)	0.76(0.35-1.65)	0.49
ICU length of stay/d, mean (SD)	9.12(8.62)	11.05(9.59)	-	0.14
Hospital length of stay/ d, mean (SD)	27.81(27.54)	26.84(17.68)	-	0.79

### P448 Mobilising ventilated patients early with interdisciplinary teams (MOVE IT)

#### XW Ling^1^, YH Lim^2^, HK Ong^3^, V Palanichamy^3^, K Leong^1^, XY Ling^1^, K Lee^4^, VK Ho^1^

##### ^1^Singapore General Hospital, Division of Anaesthesiology, Singapore, Singapore; ^2^Singapore General Hospital, Division of Nursing, Singapore, Singapore; ^3^Singapore General Hospital, Department of Physiotherapy, Singapore, Singapore; ^4^Singapore General Hospital, Department of Respiratory and Critical Care, Singapore, Singapore

**Introduction:** Survivors of critical illness face significant long term impairments in mental and physical function. Early mobilisation (EM) in the intensive care unit has been suggested to improve functional outcomes and reduce delirium in the ICU. We hypothesized that implementing a protocol for EM in the ICU would improve mobilisation rates while remaining safe.

**Methods:** Design: Prospective non-blinded observational cohort study, based on a Quality Improvement project. Data was collected from July to August 2016 pre-implementation and from November 2016 to February 2017 post implementation.

Setting: Medical and surgical ICUs of a tertiary hospital in Singapore.

Participants: All patients aged 21-99 years admitted to ICU projected to stay > 24 hours.

Interventions: We developed and implemented a protocol for early identification and mobilisation of suitable patients in ICU.

Outcome measures: The primary outcome measure was the mobilisation rate, defined as the number of days each patient underwent mobilisation, divided by the number of days each patient was eligible for mobilisation. Secondary outcome measures included incidence of adverse events during mobilisation, length of mechanical ventilation, ICU and hospital stay and in-hospital mortality.

**Results:** A total of 336 patients were analysed, of which 58% were male with a mean age of 62.7 years. MICU patients comprised 46% of the cohort. The mean APACHE II score was comparable in the pre- and post-implementation cohort (19.3% vs 20%). Following protocol implementation, the mobilisation rate increased from 0.38 to 0.57 (p=0.005), and inpatient mortality reduced from 38% to 26.6% (p=0.04). Other secondary outcomes did not differ significantly.

**Conclusions:** The implementation of an EM protocol in the intensive care units in our institution was both safe and effective. The reduced mortality we observed is intriguing and bears further study.

### P449 Cognitive and functional status assessment in neurocritical patients accompanied by occupational therapy

#### P Travassos, F Mendes, R Arboleda, S Rojas, R Vale, V Veiga, W Costa

##### Hospital BP - A Beneficência Portuguesa De São Paulo, Neurocritical Care Unit, Sao Paulo, Brazil

**Introduction:** To assess cognitive and functional status in patients followed by occupational therapy in neurocritical patients.

**Methods:** A retrospective analysis of the patients hospitalized in a neurological intensive care unit, followed by occupational therapy from March to June 2018, was carried out. The cognitive status was assessed through CAM-ICU (Confusion Assessment Method) and MEEM (Mini Mental State Examination), and functional status by means of the muscular strength degree MRC (Medical Research Council) and MIF (Functional Independence Measure), applied on admission and discharge from the ICU.

**Results:** During the period, 50 patients were followed up, of which 32 were female, with a mean age of 68.4 years and an ICU stay of 5.05 days. The activation of the occupational therapy team occurred in the first 2 days of hospitalization in 80% of the cases. Among the patients evaluated, there was an improvement in cognitive ability in 94.1% of ICU discharge, in relation to admission. The motor capacity showed improvement of 80.4% during the evaluation period.

**Conclusions:** Early occupational therapy may favor occupational performance and promote quality of life, exploring the interests, needs, and functional and cognitive abilities of individuals, minimizing the impact of long-term stay in the hospital environment.

### P450 “I See You” in ICU – Family meetings to improve the experience of family members during hospitalization

#### R Mor Levy

##### Sheba Medical Center, ICU, Ramat Gan, Israel

**Introduction:** The goal of the project “I See You” is family-centered-care based on family meetings that improve the experience of the patient´s family members during hospitalization in the ICU. The meetings focus on relaying information, raising knowledge and addressing the social and emotional needs of families. Providing support along with information was found to be the strongest predictor of family satisfaction and could lead to improve cooperation between family and staff [1].

**Methods:** Meetings and questionnaire: Family meetings consist of a multidisciplinary team, a group facilitator and combined with a multimedia presentation about the unit and equipment. In addition, they focus on social and emotional needs: managing daily routine, sharing problems, fears and anxieties and more. At the end of the session a questionnaire was given to assess the impact of the intervention. Sharing Data: At the end of the first quarter, the data from meeting was summarized and sent to the staff alongside tools for effective communication.

**Results:** The project began in February 2018. To date, 162 family members of 74 patients have attended the sessions. The topics discussed by the participants include: contact with the patient, prevention of infections, Procedures, visits, conversations with doctors, medical confidentiality; Guardianship; tracheotomy and Social issues (Fig 1). A sample of questionnaires was transferred to 57 participants report satisfaction at a very high level.

**Conclusions:** The meeting received a very positive feedback from the participants. The project has achieved its goals and therefore it has been decided to be continued.

**Reference**

1. Khalaila, R. (2012). J Adv Nurs 69:1172 – 1182

**Fig. 1 (abstract P450).**
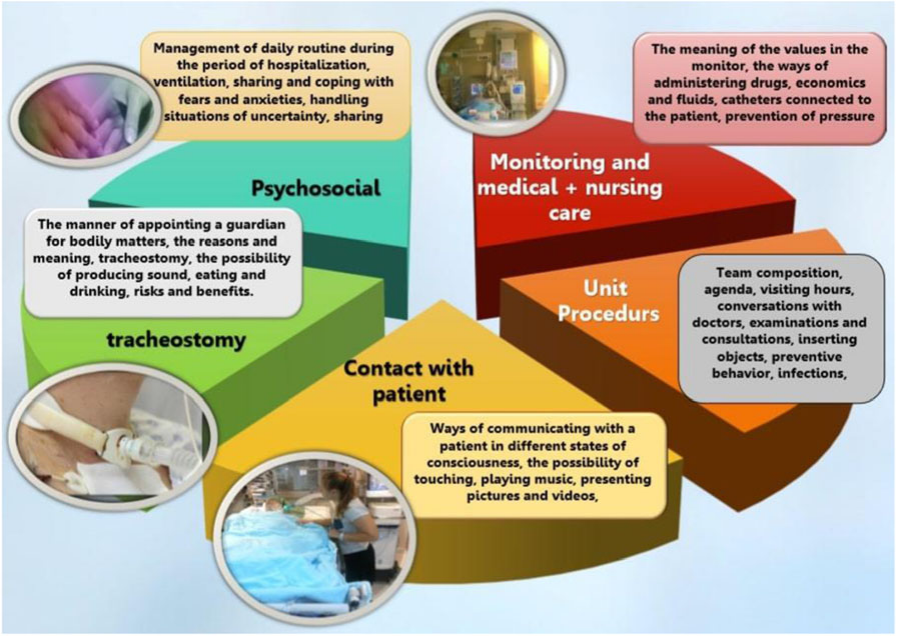
Pie

### P451 Validation of Boyd criteria and POSSUM-score on mortality and morbidity in general surgical intensive care unit

#### K Chittawatanarat, Y Chatsrisuwan

##### Faculty of Medicine, Chiang Mai University, Department of Surgery, Chiang Mai, Thailand

**Introduction:** POSSUM score and Boyd criteria are used to predict the outcome for high risk surgical patients. The aim of this study was to validation of these two measurement tools on mortality and morbidity in a University-based surgical intensive care unit (SICU) in Thailand.

**Methods:** Nine hundred and fifty two patients were enrolled onto this prospective review. All patients who had been admitted to SICU in a University-based hospital were included. All patients were collected for Boyd criteria and POSSUM score and outcomes and morbidity during SICU admission and discharge.

**Results:** A total of 952 patients were admitted to SICU at a University based Hospital in Northern region of Thailand. Eleven patients were excluded from the study due to data missing. Regarding 10 risk factors of Boyd criteria, only 6 admission risk factors significant of odds ratio (95% confidence interval) on mortality or morbidity were acute renal failure [2.5(1.43 – 4.35)], respiratory failure [10.55(3.72 – 29.97)], septicemia [3.74(2.07 – 6.78)], acute massive blood loss [3.15(1.69 – 5.88)], older age [1.44(1.03 – 2.02)], and late-stage vascular disease [2.04(1.24 – 3.36)]. Regarding the POSSUM score, the mortality and morbidity were significant higher on median (IQR) of physiologic score [29(23 – 36) vs. 21 (18 – 26); p<0.01] and operative score [12(9 – 17) vs. 11(8 – 15); p<0.01].

**Conclusions:** Only 6 of 10 variables in Boyd criteria were significant associated with morbidity or mortality. The physiologic score and operative score were significant higher in the patient on mortality and morbidity after SICU admission.

### P452 Effects of structural hospital characteristics on risk-adjusted hospital mortality in patients with severe sepsis – analysis of German national administrative data

#### D Schwarzkopf^1^, C Fleischmann-Struzeck^1^, K Reinhart^2^, D Thomas-Rüddel^2^

##### ^1^Jena University Hospital, Center for Sepsis Control and Care, Jena, Germany; ^2^Jena University Hospital, Department of Anaesthesiology and Intensive Care Medicine, Jena, Germany

**Introduction:** Administrative data can be used to estimate risk-adjusted mortality in severe sepsis for quality comparisons between hospitals. There is little knowledge on the effects of structural hospital characteristics on mortality.

**Methods:** Patients with severe sepsis were identified in German national claims data. Based on a previously reported model [1] risk- and reliability adjusted mortality (RSMR) per hospital was calculated. RSMR were linked to structural characteristics of hospitals obtained from legally obliged quality reports. The relationship between structural characteristics and presence of an RSMR above the national average was analyzed in logistic regressions. A backward selection was used to identify most relevant predictors.

**Results:** 134.000 cases from 1276 hospitals were used to calculate the RSMR per hospital. Observed hospital mortality was 42%. Median RSMR was 42% [IQR: 38%-47%]. In univariate analysis a RSMR above national average was predicted by academic teaching status of the hospital, having a higher number of beds, a higher number of cases per bed, and a higher number of specialized technical equipment (all p < 0.01). Hospitals owned by non-profit organizations showed higher mortality compared to private or state owned hospitals (p < 0.001). After backward selection of predictors only number of specialized equipment (OR=1.07 [CI 1.04, 2], p < 0.001) and ownership (private: OR=0.57 [0.42, 0.78], state: OR=0.6 [0.45, 0.82], p < 0.001) remained in the model.

**Conclusions:** Hospitals of the highest levels of care – indicated by number of technical equipment – might be disadvantaged in current risk-adjustment models based on individual patient claims data. Structural characteristics might be needed to be included in risk-modeling. Whether hospitals owned by non-profit organizations have quality issues or different coding strategies in claims data needs further investigation.

**Reference**

[1] Schwarzkopf D et al. PLoS One 13:e0194371, 2018

### P453 Prehospital quick sequential organ failure assessment score to predict in-hospital mortality among patients with trauma: an analysis of a nationwide registry

#### N Shibata, K Miyamoto, S Kato

##### Wakayama Medical University, Department of Emergency and Critical Care Medicine, Wakayama, Japan

**Introduction:** The quick sequential organ failure assessment (qSOFA) score is a simple tool used to identify severe patients with infection. As this score is calculated from three variables that can be measured at the scene of trauma-systolic blood pressure, respiratory rate and consciousness-the prehospital qSOFA score may also be a good predictor of mortality in trauma patients. So we evaluated the discriminative ability of the prehospital qSOFA score in patients with trauma for in-hospital mortality.

**Methods:** This is a retrospective multicenter study using the data from nationwide trauma registry in Japan. We included 42722 patients with trauma aged ≥18 years old transferred to hospitals from scene. Primary outcome is in-hospital mortality.

**Results:** The mean age was 59.4±21.5 years old and 27069 patients (63%) were male. In-hospital mortality occurred in 2612 patients (6%). In-hospital mortality in each qSOFA score was 105/11783(0.9%), 941/17839(5%), 1280/11132(12%) and 286/1968(15%) in qSOFA score 0, 1, 2 and 3, respectively (P<0.0001 for trend). Area under receiver operating characteristics curve (AUROC) of the aqSOFA score for in-hospital mortality was 0.70(95% confidence interval 0.69-0.71). If we use the cutoff ≥1, sensitivity and specificity of the qSOFA score were 0.96 and 0.29.

**Conclusions:** In patients with trauma, the prehospital qSOFA score was strongly associated with in-hospital mortality. We can identify patients with very low risk of death by using the cutoff ≥1 of the prehospital qSOFA score.

### P454 Prospective validation of qSOFA score for the diagnosis and prognosis of sepsis in the emergency department

#### A Safarika^1^, I Mitrou^1^, S Kapsokolis^1^, G Giannikopoulos^2^, K Katsaros^3^, N Voloudakis^4^, N Tsokos^5^, P Koutoukas^6^, E Giamarellos-Bourboulis^1^

##### ^1^National and Kapodistrian University of Athens, 4th Department of Internal Medicine, Athens, Greece; ^2^Syros General Hospital, Department of Internal Medicine, Syros, Greece; ^3^Argos General Hospital, Department of Surgery, Nafplion, Greece; ^4^Aristotle University, 2ns Department of Surgery, Thessaloniki, Greece; ^5^Chalkida General Hospital, Department of Internal Medicine, Chalkida, Greece; ^6^Sparti General Hospital, Department of Internal Medicine, Sparti, Greece

**Introduction:** Only one prospective study is available of the validation of the diagnostic and prognostic role of qSOFA (quick SOFA score) in the emergency department (ED). A prospective study was conducted in Greek EDs.

**Methods:** The PROMPT study (ClinicalTrials.gov NCT03295825) run in the ED of six hospitals in Greece among patients with suspected infection and presence of at least one of fever, hypothermia, tachycardia, tachypnea and chills. Clinical data were collected and the 28-day outcome was recorded. Sepsis was defined by the Sepsis-3 criteria.

**Results:** The sensitivity and the specificity of at least 2 signs of qSOFA for the diagnosis of sepsis was 78.4% and 96.8% respectively and for the prognosis of 28-day mortality 45.7% and 94.2% respectively. The odds ratio for 28-day mortality when qSOFA was equal to or more than 2 was 59.67 among patients with Charlson’s comorbidity index (CCI) equal to or less than 2; this was 7.45 among patients with CCI more than 2 (p: 0.038 between the two ORs by the Breslow-Day’s test; p: 0.040 by the Tarone’s test).

**Conclusions:** Data validated the sensitivity of qSOFA for the diagnosis of sepsis. CCI was an independent predictor of severity. qSOFA could better predict unfavorable outcome among patients with low CCI.

### P455 Comparative accuracy between two sepsis severity scores in predicting hospital mortality among sepsis patients admitted to intensive care unit

#### N Sathaporn, B Khwannimit

##### Prince of Songkla University, Internal Medicine, Hat Yai, Thailand

**Introduction:** Recently, the New York Sepsis Severity Score (NYSSS) was developed to predict hospital mortality in sepsis patients. The aim of this study was to compare the accuracy of NYSSS with the Sepsis Severity Score (SSS) and other standard severity scores for predicting hospital mortality in sepsis patients.

**Methods:** A retrospective analysis was conducted in a medical intensive care unit of a tertiary university hospital. The performance of severity scores was evaluated by discrimination, calibration, and overall performance. The primary outcome was in-hospital mortality.

**Results:** Overall 1,680 sepsis patients were enrolled, 895 patients (53.3%) were classified to septic shock by Sepsis-3 definition. Hospital mortality rate was 44.4%. The NYSSS predicted hospital mortality 34.6+/-21.5%, which underestimated prediction with SMR 1.28 (95%CI 1.19-1.38). However, the SSS predicted hospital mortality 47+/-22.2%, which slightly overestimated mortality prediction with SMR 0.94 (95%CI 0.88-1.01). The NYSSS had the moderate discrimination with an AUC of 0.772 (95% CI 0.750-0.794), in contrast to the SSS presented good discrimination with an AUC of 0.889 (95%CI 0.873-0.904). The AUC of SSS was statistically higher than that of NYSSS (p<0.0001). Nevertheless the APACHE IV and SAPS II showed the best discrimination with AUC of 0.937. The AUC of the NYSSS and SSS was significant lower than that of APACHE II, III, IV, SAPS II and SAPS 3 (Figure 1). The calibration of all severity scores was poor with the Hosmer-Lemeshow goodness-of-fit H test < 0.05. The NYSSS was the lowest overall performance with Brier score 0.201. The APACHE IV present the best overall performance with Brier scores 0.107.

**Conclusions:** The SSS indicated better discrimination and overall performance than the NYSSS. However the calibration of both sepsis severity scores and another severity score were poor. Furthermore, specific severity score for sepsis mortality prediction needs to be modified or customized to improve the performance.

**Fig. 1 (abstract P455).**
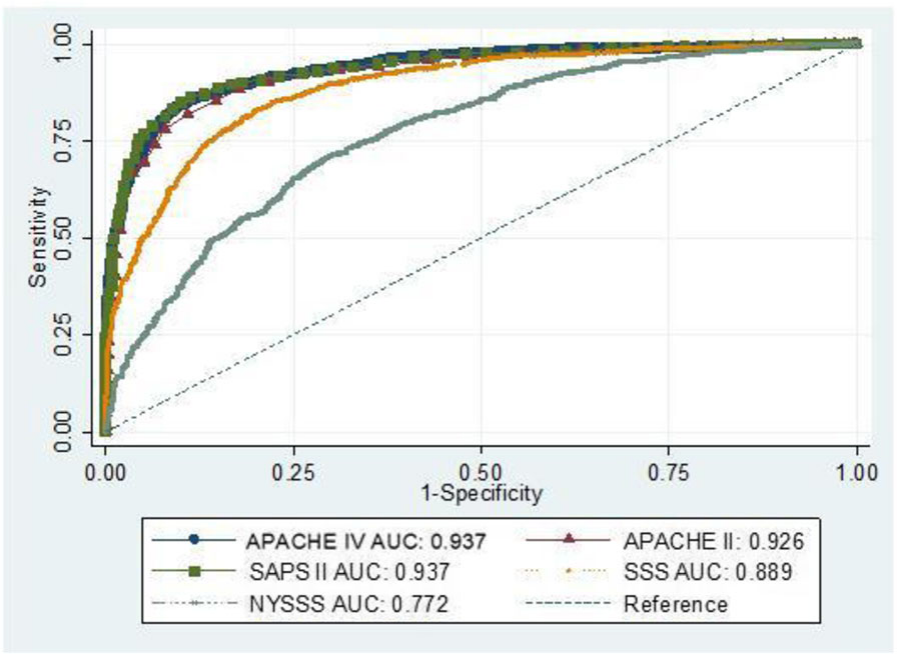
Comparison the area under the receiver operating characteristic curve of NYSSS and SSS with other severity scores for predicting hospital mortality in ICU sepsis patients

### P456 Can early changes in metabolic markers predict outcome?

#### T Aquilina^1^, T Khan^2^, O Boyd^1^

##### ^1^Brighton and Sussex University Hospitals NHS Trust, Brighton, UK, Brighton, United Kingdom; ^2^Brighton and Sussex Medical School, Brighton, UK, Brighton, United Kingdom

**Introduction:** Metabolic markers, especially lactate, have been shown to predict mortality in acutely unwell patients. We hypothesised that early changes in metabolic markers over time would better predict mortality and length of stay, with patients who correct their metabolic derangement having lower risk of death and reduced length of stay (LOS).

**Methods:** Single centre, retrospective cohort study in a 31 bed ICU. We included all patients who had an arterial measurement of lactate, PaCO2, base excess (BE) and pH on admission and at 6 hours after admission to ICU between 01/01/2016 and 31/12/2017. The ‘clearance’ of these markers was calculated using the equation ((value at admission – value at 6 hours)/value at admission). Clearance calculations only included those patients with deranged results on admission (Lactate>2mmol/l, BE<-2mmol/l, pH<7.35, PaCO2>6.0kPa). ROC analysis was used to predict in-hospital mortality and length of stay, using both the initial admission values, and using the clearance value, as well as ICNARC and APACHE II scores for comparison. If a patient was admitted twice in the time period, only the first admission was included.

**Results:** 1506 patients were included (sex ratio 1.5, mean age 61.6). The number of patients with deranged markers at admission are as follows: lactate n=531, BE n=816, pH n=633, PaCO2 n=376. For predicting mortality, Lactate, BE, and pH clearance had area under the ROC curve (AUC) =0.629, 0.621 and 0.535, lower than the AUC for the admission values (lactate=0.688, BE=0.665, pH=0.668) (Fig 1&2, Table 1). None of the values tested had a AUC greater than 0.6 for predicting length of stay.

**Conclusions:** The clearances of metabolic markers over the initial 6 hours after ICU admission does not provide better prognostic information than the value at admission. Initial lactate level was the best predictor of mortality, but compared poorly to ICNARC score. Metabolic markers do not accurately predict length of stay.

**Table 1 (abstract P456).** Area under the curve (AUC) for ROC curves predicting in-hospital mortality

	Initial value	95% confidence interval	Clearance value	95% confidence interval
	AUC		AUC	
Lactate	0.688	0.654-0.722	0.629	0.579-0.68
BE	0.665	0.629-0.701	0.621	0.578-0.663
pH	0.668	0.631-0.704	0.535	0.484-0.586
PaCO2	0.559	0.451-0.528	0.561	0.493-0.63
APACHE II	0.724	0.691-0.757		
ICNARC	0.836	0.812-0.861		

**Fig. 1 (abstract P456).**
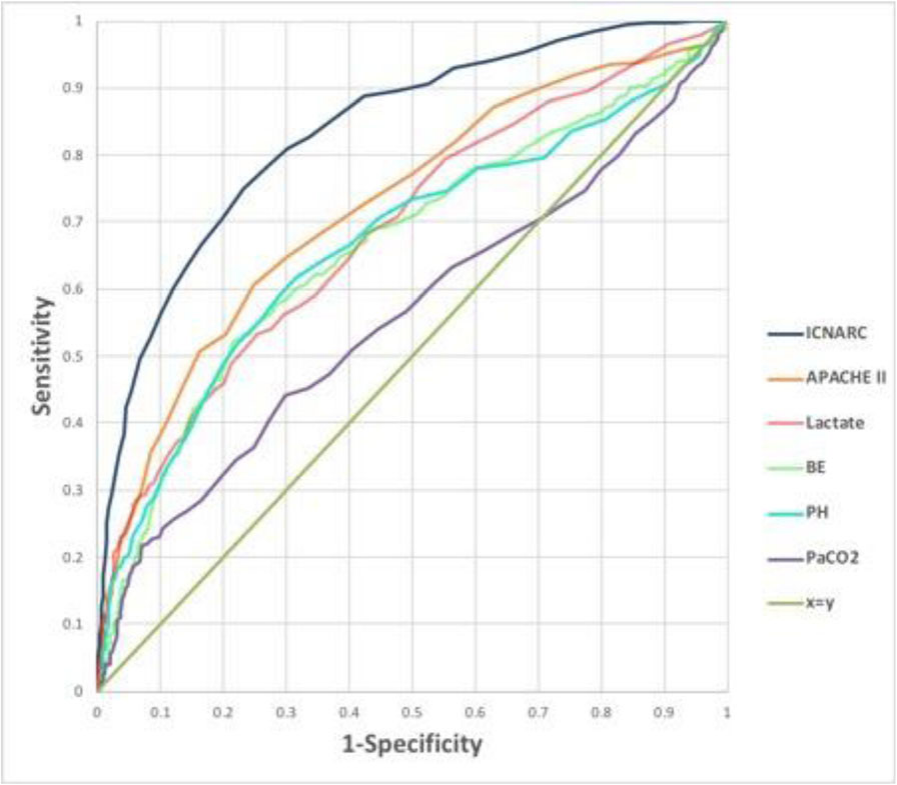
ROC showing prediction of in-hospital mortality based on initial levels of metabolic markers, as well as ICNARC and APACHE II score

**Fig. 2 (abstract P456).**
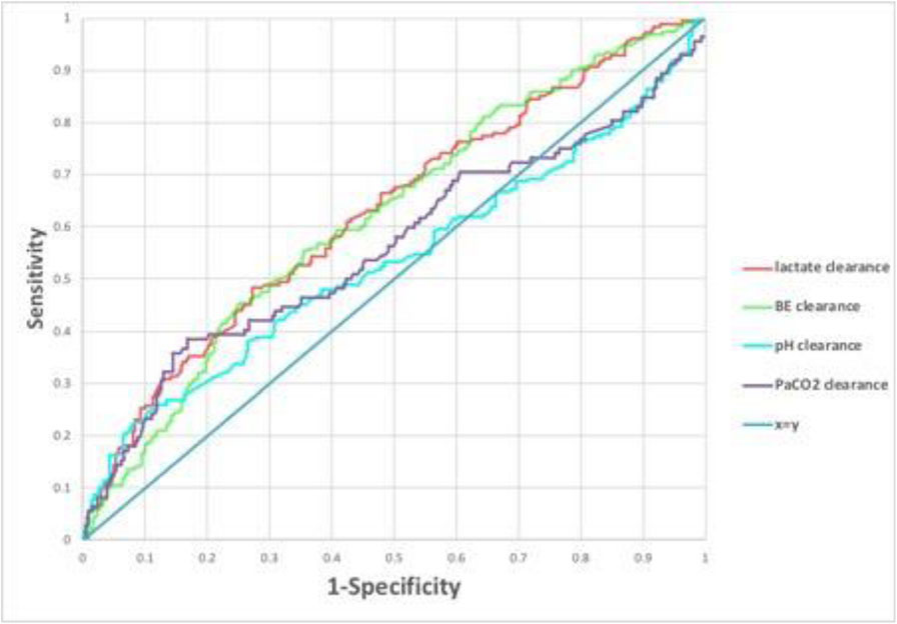
ROC showing prediction of in-hospital mortality based on clearance of lactate, BE, pH and PaCO2

### P457 Aromatic metabolites as prognostic criteria in ICU

#### N Beloborodova^1^, Y Sarshor^2^, A Nikiforov^2^, E Chernevskaya^3^

##### ^1^Negovsky Research Institute of General Reanimatology Federal Research and Clinical Center of Intensive Care Medicine and Rehabilitology, Lab.metabolisms in critical state, Moscow, Russia; ^2^Veresaev State Clinical Hospital, Moscow, Russia, Russia; ^3^Negovsky Research Institute of General Reanimatology Federal Research and Clinical Center of Intensive Care Medicine and Rehabilitology, Moscow, Russia

**Introduction:** Aromatic microbial metabolites (AMM), such as phenyllactic (PhLA), p-hydroxyphenylacetic (p-HPhAA), and p-hydroxyphenyllactic (p-HPhLA) are involved in the pathogenesis of septic shock and are associated with mortality [1]. According to previous studies, AMM have a high prognostic value in patients with abdominal infection [2, 3]. We hypothesize that AMM have the prognostic value in patients with pneumonia in ICU.

**Methods:** Data of patients with community-acquired pneumonia was obtained on admission to ICU. The levels of AMM (PhLA, p-HPhLA and p-HPhAA) were measured in blood serum using gas chromatography with flame ionization detector and compared in 2 groups of patients: with favorable and with lethal outcome (Mann-Whitney U-test). Spearman’s correlations between AMM and clinical and laboratory data were calculated. Using method of logistic regression and ROC analysis, we measured the prognostic value of AMM.

**Results:** 54 patients were included in the analysis. Patients with favorable (n=35) and with lethal (n=19) outcome differed in APACHE II (8 (6-12) vs 18 (12-22), p<0.001), SOFA (2(1-4) vs 7(6-10), p<0.001), concentrations of PhLA (0.6 (0.3-1.1) vs 1.9 (0.7-3.1) μÌ, p<0.001), p-HPhLA (1.8 (1.4-3.1) vs 4.2 (2.9-9) μÌ, p<0.001), p-HPhAA (1.5 (0.8-3.2) vs 3.7 (0.9-9.3) μÌ, p=0.05) and their sum ∑3PhCA (4.3 (2.8-8.4) vs 13.8 (4.9-35.1) μÌ, p<0.001). Correlation analysis was made (Table 1). It was revealed, that some AMM have similar prognostic characteristics in comparison with SOFA and CURB-65 scales; high level of AMM is associated with high risk of death (ROC-analysis - Fig. 1).

**Conclusions:** Serum concentrations of AMM can be used as independent and practical criteria for the assessing of prognosis in patients with infection in ICU.

**Acknowledgement**

This study was supported by the Russian Science Foundation, Grant Number: 15-15-00110

**References**

1) Beloborodova N et al Shock 50,3:273-279,2018

2) Beloborodova N et al. Crit Care 20, Suppl 3:P19,2016

3) Beloborodova N et al. Shock 44, Suppl 2:13, 2015

**Table 1 (abstract P457).** Correlation between AMM and clinical / laboratory data

	PhLA	p-HPhLA	p-HPhAA	?3AMM
APACHE II	0.56***	0.49***	0.54***	0.65***
SOFA	0.52***	0.41***	0.47***	0.53***
CURB -65	0.32*	0.33***	0.57**	0.52***
Lactate	0.67**	0.76**	0.42**	0.63**
Lethal outcome	0.6***	0.56***	0.32***	0.56***

Correlations are significant at: * p <0.05, ** p <0.01, *** p <0.001

**Fig. 1 (abstract P457).**
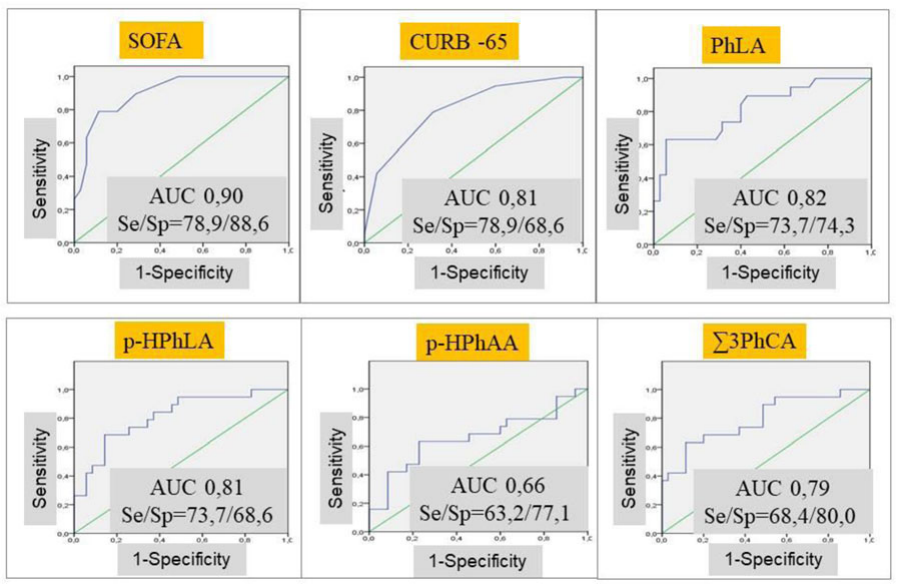
ROC-curves of the lethal outcome predictors

### P458 Mean platelet volume as a prognosis factor in cancer patients admitted to intensive care

#### P Semedo, S Fernandes

##### Centro Hospitalar Universitário Lisboa Norte, Serviço de Medicina Intensiva, Lisbon, Portugal

**Introduction:** Critically ill patients (pts) with cancer are increasingly admitted in the intensive care unit (ICU), but there are still questions about benefit from critical care admission. Hemogram parameters such as mean platelet volume (MPV) and neutrophil-lymphocyte ratio (NLR) have been associated with response to oncologic treatment. We aimed to determine the prognostic value of MPV in critically ill cancer patients.

**Methods:** Retrospective cohort study including patients (pts) with cancer admitted to a level III 22-bed ICU from a tertiary university-affiliated urban referral hospital ICU, between January-2014 and September-2018. Pts with central nervous system neoplasms or submitted to elective surgeries were excluded. Descriptive analysis and χ test, Pearson´s, Wilcoxon rank-sum, uni and multivariate logistic regressions were used when appropriate.

**Results:** From a total of 105 pts identified, 57.1% (n= 60) were admitted after emergent surgery and 42.9% (n=45) for medical reasons. Global ICU mortality was 25.7% (n=25). In comparison to survivors, the patients that died had a similar age [73.2 (interquartile range (IIQ) 61.0-80.4) vs 75.1 (IIQ 64.5-83.0) years] but as expected a higher SAPSII score [37. (IIQ 27.0-68.0) vs 70.0 (IIQ 68.0-80.0)]. MPV median was lower in survivors when compared with non-survivors [8.3 (IIQ 8.0-22.8) vs 9.1 (IIQ 9.6-9.9), p=0.005]. The other hemogram parameters did not differ between groups (Table 1). When adjusted for severity score, in patients submitted to emergent surgery, the MPV value was still independently associated with mortality (OR 1.146 CI 1.057-1.243, p=0.042), and its ROC curve (AUC) was 0.861 to mortality (Figure 1).

**Conclusions:** MPV is a cheap and easily accessible marker which can add prognostic value in this specific population. In the future, we will validate it in a larger cohort of cancer pts admitted to intensive care.

**Table 1 (abstract P458).** Descriptive analysis of clinical and hemogram related variables

	Survivors (n=78)	Non-survivors (n=27)	p-value
Admission due to urgent surgery, n (%)	49 (62.8)	11 (40.7)	0.070
Gastrointestinal tumor localization, n(%)	47 (60.3)	17 (63.0)	0.676
NLR, median (IIQ)	10.3 (13.6-157.1)	6.9 (19.9-37.9)	0.676
PLR, median (IIQ)	257.2 (270.1-1349.2)	202.3 (436.3-633.3)	0.747
LMR, median (IIQ)	1.9 (1.9-8.8)	2.0 (3.3-12.4)	0.207
MPVPCR, median (IIQ)	3.1 (3.5-22.8)	4.0 (4.7-67.0)	0.120
MPV, median (IIQ)	8.3 (8.0-22.8)	9.1 (9.6-9.9)	0.005

**Fig. 1 (abstract P458).**
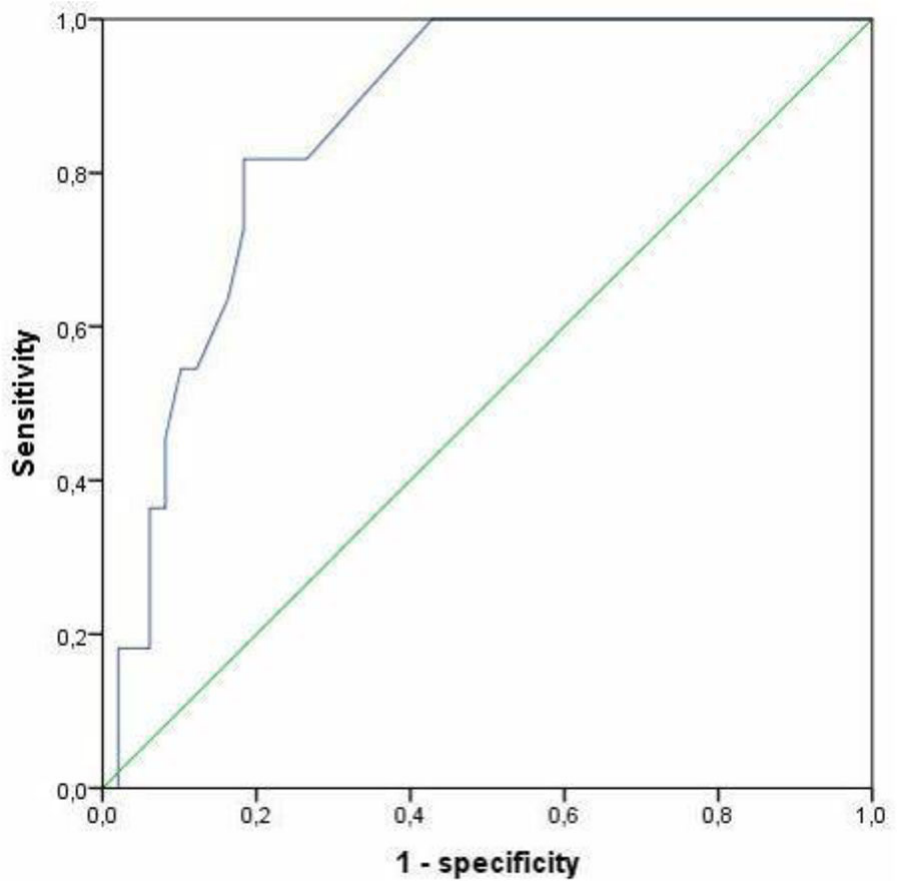
MPV receiver operating characteristic (ROC) curve for ICU mortality

### P459 Haematological malignancy in critical care: outcomes and risk factors

#### C Denny^1^, A Lwin^1^, V Bhardwaj^1^, J Radhakrishnan^1^, S Elshazly^2^

##### ^1^Broomfield Hospital, Mid Essex NHS Trust, Critical Care, Chelmsford, United Kingdom; ^2^Broomfield Hospital, Mid Essex NHS Trust, Haematology, Chelmsford, United Kingdom

**Introduction:** About 7% of patients admitted to hospital with a haematological malignancy will become critically ill [1]. Life expectancy in these patients is poor with a 6 month mortality of 60% or more in specialist units [2]. In contrast, patients without critical illness can expect a 5 year survival rate exceeding 60% for many cancers. This disparity results in differences of opinion on the best strategy for such patients among haematologists and critical care physicians. We conducted a local quality improvement project to quantify mortality and risk factors in critically ill patients with a haematological malignancy in our hospital.

**Methods:** Patients admitted to the critical care unit of Broomfield Hospital, a district general hospital with tertiary specialist services, from January 2011 to December 2017 with haematological malignancy were included in the analysis. Patients in remission for more than 20 years and patients admitted following elective surgery were excluded from analysis. Death in critical care or in hospital after critical care discharge were the primary outcomes. Mortality was correlated with demographic data using simple statistical measures and regression analysis.

**Results:** 93 patients were included in the analysis. Overall mortality was 45%(n=42). Survivors tended to be younger (65 vs 71 years) but had similar clinical frailty scores. Early critical care admission (within 24 hours) was associated with better survival (72.5 vs 35.7%). Non-survivors had a greater incidence of sepsis and respiratory failure, and required more ventilatory and vasopressor support. Mortality was higher in patients requiring more than one organ support.

**Conclusions:** The overall mortality in our data is lesser than previously published data but supports the conclusion that mortality is determined primarily by the number of organs supported with the effects of malignancy playing a secondary role.

**References**

1. Massion et al. Crit Care Med, 30, 2260–70, 2002.

2. Bird et al. Br J Anaesth, 108, 452–9, 2012.

### P460 Cancer patients in the ICU: where do we stand?

#### A Catarino^1^, L Machado^2^, B Marques^3^, A Marques^1^, P Casanova^1^, P Martins^1^

##### ^1^CHUC - Centro Hospitalar e Universitário de Coimbra, Serviço Medicina Intensiva, Coimbra, Portugal; ^2^CHUC - Centro Hospitalar e Universitário de Coimbra, Serviço Medicina Interna, Coimbra, Portugal; ^3^CHUC - Centro Hospitalar e Universitário de Coimbra, Serviço Hematologia, Coimbra, Portugal

**Introduction:** The aim of this study was to evaluate the characteristics of the oncologic patients in our ICU, namely the admission diagnosis, related or not with cancer or cancer therapy and mortality. The number of patients living with cancer has been increasing. ICU admission may be required for management of acute illnesses associated with the underlying malignancy or complications of therapy.

**Methods:** Retrospective study of 99 oncologic patients admitted to the ICU between January and December of 2016.

**Results:** Of the 638 patients admitted to the ICU in 12 months, 99 (15.5%) had cancer: 70 (70.7%) solid tumors and 29 (29.3%) hematological cancer. SAPS II score was 50.5±17.6 in solid and 45.9±17.5 in hematological tumors patients. Twenty-two (81,5%) patients with hematological malignancies were admitted due to medical complications, the majority (18; 66.7%) directly related with cancer or cancer treatment. On the contrary, in solid tumors, surgical pathology was the main cause of ICU admission: 56 (77.8%) cases, being 31 (55.4%) complications of cancer related elective surgery, 20 (35.7%) urgent surgery related to the tumor and 5 (8.9%) urgent surgery unrelated to cancer. Oncologic patients had higher ICU mortality rate (39.4%) when compared with non-cancer population (29.6%). Hematological patients had 63% of ICU mortality rate and solid tumor 30.6%.

**Conclusions:** Solid tumors patients are more likely to be a surgical admission and hematological patient are at higher risk of a medical complication. The mortality of patients with solid cancer was similar to non-cancer ICU patients, but patients with haematological malignancies had a much worse outcome.

### P461 Does frailty score at intensive care unit admission affect mortality at one year? A retrospective observational cohort study

#### D Hewitt, M Booth

##### Glasgow Royal Infirmary, Intensive Care Unit, Glasgow, United Kingdom

**Introduction:** This study primarily aimed to investigate frailty´s impact on mortality, 1 year after ICU admission. Frailty is a syndrome characterised by decreased reserve and greater vulnerability. Consequently, we hypothesised that frail patients would exhibit decreased survival compared to non-frail patients. If true, frailty scoring [1] could increase predictive accuracy of current scoring systems and facilitate better informed decisions. To validate this, the relationship between frailty score and outcomes must be tested extensively.

**Methods:** This single-centre retrospective observational cohort study examined prospectively collected clinical data from 400 critically ill patients. Frailty was assessed using the Clinical Frailty Scale [1] (CFS) and defined as CFS ≥5. Unadjusted and adjusted analyses tested the relationships of frailty, covariates and outcomes.

**Results:** Of 400 eligible patients, 111 (27.8%) were frail and 289 (72.3%) were non-frail. Frail patients were older (62 vs 56, p<0.001) and had higher APACHE-II scores (22 vs 19, p<0.001). Females were more likely to be frail than males (34.1% vs 22.9% frail, p=0.018) (Table 1). Frail patients were less likely to survive ICU (p=0.03), hospital (p=0.003) or to 1 year (p<0.001) (Figure 1). Increasing levels of frailty were associated with increasing risks of death at 1 year (p<0.001) (Figure 2). Frailty significantly increased 1-year mortality hazards in unadjusted analyses (HR 1.96; 95%CI; 1.41-2.72; p<0.001) and covariate-adjusted analyses (HR 1.41; 95%CI 1.00-1.98; p=0.0497) (Table 2).

**Conclusions:** Frailty was common and associated with greater age, more severe illness and female gender. Frailty was significantly associated with heightened mortality risks in both unadjusted and covariate-adjusted analyses. Frailty scoring may encapsulate variables affecting mortality which are omitted in current predictive systems, making it a promising risk stratification and decision-making tool in ICU.

**Reference**

[1] Rockwood K et al. CMAJ. 2005;173:489-495

**Table 1 (abstract P461).** Patient characteristics at inclusion. Frail patients were statistically significantly older, more likely to be female and more severely sick

Variable	Non-Frail (CFS 1-4)	Frail (CFS 5-9)	p value
Number (%)	289 (72.3)	111 (27.8)	
Age (median [IQR])	56.00 [44.00, 67.00]	62.00 [53.50, 74.00]	<0.001
Sex, Female (%)	114 (39.4)	59 (53.2)	0.018
Admission type, Medical (%)	125 (43.3)	52 (46.8)	0.592
Unplanned ICU Admission (%)	258 (89.3)	98 (88.3)	0.918
APACHE II Score (median [IQR])	19.00 [14.00, 24.00]	22.00 [19.00, 28.50]	<0.001

**Table 2 (abstract P461).** Unadjusted-adjusted 1-year mortality Cox proportional hazards. Frailty was significantly associated with heightened mortality hazards in both unadjusted and covariate adjusted analyses

Variable	Hazard Ratio (95% Confidence Interval)	p value	Adjusted Hazard Ratio (95% Confidence Interval)	p value
Age	1.03 (1.01-1.04)	<0.0001	1.02 (1.00-1.03)	0.0130
Sex	1.11 (0.80-1.53)	0.5416		
Frail	1.96 (1.41-2.72)	<0.0001	1.41 (1.00-1.98)	0.0497
Medical Admission	1.46 (1.06-2.02)	0.0200	1.14 (0.81-1.61)	0.4648
Unplanned ICU Admission	2.35 (1.20-4.61)	0.0130	2.01 (1.00-4.06)	0.0510
APACHE-II Score	1.09 (1.07-1.11)	<0.0001	1.07 (1.05-1.10)	<0.0001

**Fig. 1 (abstract P461).**
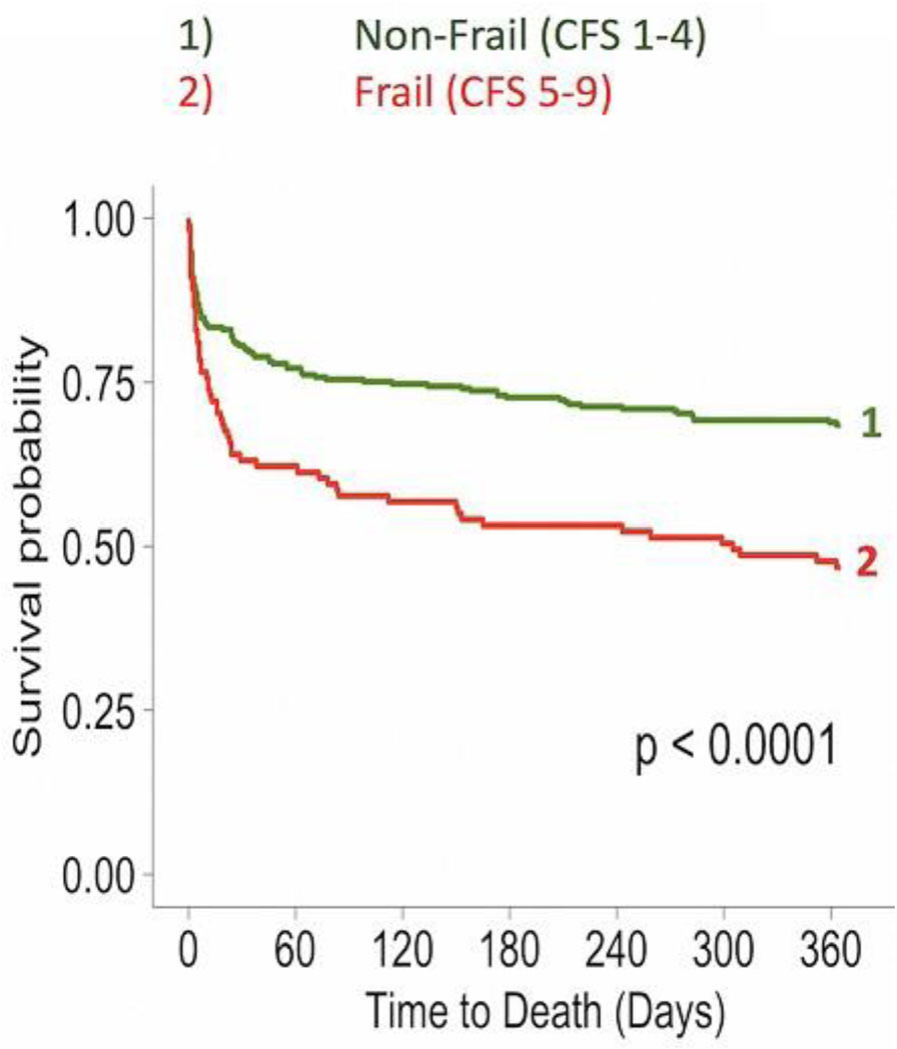
Unadjusted survival curves stratified by frailty status. Frail patients were statistically significantly less likely to survive to 1 year

**Fig. 2 (abstract P461).**
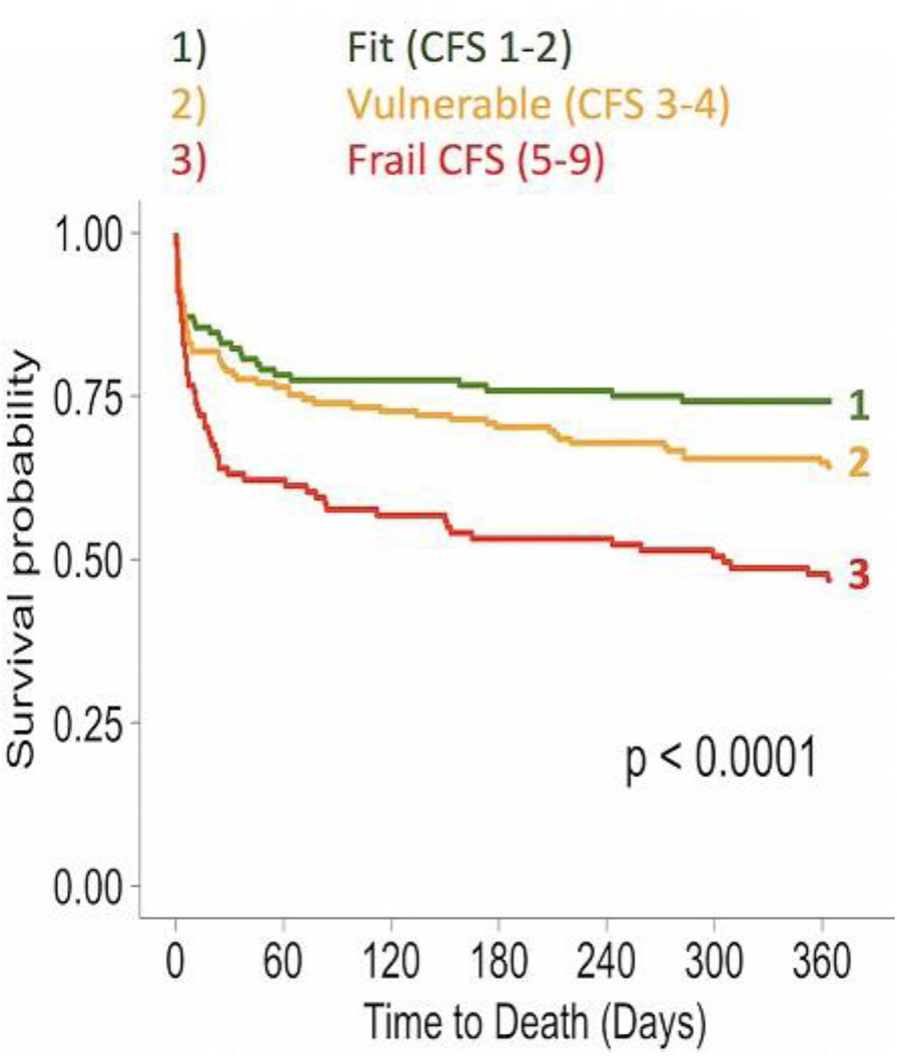
Unadjusted survival curves stratified by frailty subgroups. Increasing levels of frailty were associated with increasing risks of death at all points up to 1 year

### P462 Frailty, outcomes, recovery and care steps of critically ill patients (FORECAST) pilot study

#### J Muscedere^1^, J Boyd^2^, D Maslove^2^, S Sibley^2^, M Hunt^2^, P Norman^2^, A Day^1^

##### ^1^Queens University, Critical Care Medicine, Kingston, Canada; ^2^Queens University, Department of Critical Care Medicine, Kingston, Canada

**Introduction:** Frailty in the critically ill is associated with increased morbidity and mortality but the optimal timing of frailty assessment, how to best measure frailty, reasons for adverse outcomes and how critical illness impacts frailty are unknown [1]. In preparation for a multi-center study designed to address these knowledge gaps, we conducted a pilot study whose aim was to assess feasibility as determined by recruitment rates, ability to assess frailty at ICU admission and hospital discharge, ability to measure ICU and hospital processes of care and ability to conduct 6-month assessments.

**Methods:** Single center observational study enrolling consecutive patients > 55 y.o. who required mechanical ventilation, vasopressors or treatment of acute renal failure > 24 hours. Frailty was measured at ICU admission and hospital discharge with the Clinical Frailty Scale (CFS) and Frailty Index (FI). Processes of care were measured during ICU and hospital stay along. A 6 month in-person assessment was also conducted..

**Results:** Characteristics of the 49 patients enrolled are in Table 1. Feasibility criteria were: consent rate - 74%, enrollment rate – 1/week. At hospital discharge, 37 (76%) were alive; 33 (89%) had frailty assessments done. At 6 months, 27/41 (66%) were alive and 22 (81%) completed follow-up. Processes of care were readily captured during the ICU stay but were difficult to capture during the ward stay. Frailty metrics are outlined in Table 2. On admission, FI and CFS correlation was 0.8 with Kappa = 0.57 (95% CI .34 – 0.8); on hospital discharge they were 0.36, Kappa = 0.24 (95% CI -.11 - .59)

**Conclusions:** A multi-center study is feasible but follow-up losses due to mortality and inability to return for assessment will require sample size adjustment. Frailty characterization is method dependent, can be done on hospital discharge but varies with time of assessment. These findings will need to be confirmed in our larger study currently in progress.

**Reference**

1. Muscedere J et al, Intensive Care Med 2017; 43: 1105

**Table 1 (abstract P462).** Baseline characteristics (n = 49)

Age	68.7 ± 7.9
Sex	18 (37%)
APACHE II	22.3 ± 6.2
Medical Admission	37 (76%)
Full Resuscitation Status	47 (96%)
Charlson CI	1.8 ± 1.5
FI	0.2 ± 0.1
CFS	3.8 ± 1.8

**Table 2 (abstract P462).** Frailty characterization on hospital admission and discharge

	CFS Not Frail	CFS Frail	FI Not Frail	FI Frail
	CFS < 5	CFS ≥ 5	FI < 0.2	FI ≥ 0.2
ICU Admission (n = 49)	32 (65%), (2.8 ± 1.0)	17 (35%), (5.8 ± 0.8)	26 (53%), (0.1 ± 0.1)	23 (47%), (0.4 ± 0.1)
Hospital Discharge (n = 33)	11 (33%), (3.2 ± 0.9)	22 (67%), (5.7 ± 0.8)	12 (38%), (0.1 ± 0.05)	20 (63%), (0.4 ± 0.1)
Hospital Mortality	4 (12.5%)	8 (47.1%)	1 (3.8%)	11 (47.8%)

### P463 Neural networks allow early prediction of clinical interventions on the ICU

#### F Catling, AH Wolff

##### Barnet Hospital, Intensive Care Unit, London, United Kingdom

**Introduction:** This study presents a neural network (NN) model designed to predict clinical interventions on the ICU. The model is also used for predicting death. These events are relatively rare, and are therefore challenging for conventional models to predict. If they could be predicted earlier and more reliably, then staff could consider a variety of preemptive actions, in a more-timely manner.

**Methods:** We developed our model using data from 4544 patient admissions to a UK-based ICU between January 2014 and October 2018. This comprised patient demographics and 35370 days of longitudinal measurements at hourly intervals, including vital signs, blood gases, laboratory results and nursing observations. We used a feedforward NN to encode the demographic data and a temporal convolutional NN to encode the longitudinal data. For each hour of each admission, we use the combined NN outputs to predict risk of each event in the following t hours, where t was prespecified differently for each event to complement clinical workflow. The predicted events were intubation, successful extubation and death (t=6); fluid challenge and commencing noradrenaline (t=3); up- and down-titrating FiO2 (t=1). Model training, tuning and final evaluation were performed on 70%, 10% and 20% of the data respectively. Model parameters were tuned by Bayesian optimisation.

**Results:** Model performance is summarised in Table 1. Number of events (% of admissions with at least 1 event) in the dataset was 2271 (23.6%) for intubation, 2601 (29.4%) for successful extubation, 795 (17.3%) for death, 7539 (41.4%) for fluid challenge, 1504 (30.9%) for commencing noradrenaline, 44075 (83.3%) for up- and 57250 (89.4%) for down-titrating FiO2. Median (IQR) admission length in the dataset was 4.69 (2.17 - 8.92) days.

**Conclusions:** Our model shows good discrimination in early prediction of clinical interventions as well as death on the ICU. We plan to extend the model to predict other events, and to conduct a rigorous external validation.

**Table 1 (abstract P463).** Predictive performance of the tuned model on the final evaluation data

Event	AUROC (95% CI)	Average precision (95% CI)
Intubation	0.835 (0.823 - 0.846)	0.145 (0.129 - 0.167)
Extubation (successful)	0.873 (0.860 - 0.887)	0.402 (0.364 - 0.434)
Fluid challenge	0.831 (0.824 - 0.839)	0.195 (0.183 - 0.209)
Noradrenaline (commencing)	0.982 (0.977 - 0.986)	0.456 (0.419 - 0.498)
FiO2 (up-titrating)	0.887 (0.884 - 0.890)	0.664 (0.656 - 0.670)
FiO2 (down-titrating)	0.903 (0.901 - 0.905)	0.712 (0.706 - 0.717)
Death	0.973 (0.968 - 0.977)	0.443 (0.414 - 0.480)

### P464 An airway pressures score for the prediction of mortality in mechanically ventilated patients

#### W Zarrougui, N Fraj, Maboujelben, E Ennouri, M Zghidi, H Zorgati, A Khedher, A Azouzi, I Ben Saida, K Meddeb, M Boussarsar

##### Farhat Hached University Hospital, Sousse, Tunisia; ^2^Farhat Hached University Hospital, Medical Intensive Care Unit, Sousse, Tunisia

**Introduction:** The aim was to develop and validate a mortality airway pressures (Paw) prediction score (Paw-MPS) for mechanically ventilated (MV) patients.

**Methods:** A retrospective chart reviews of 304 consecutive MV patients was conducted in our MICU from November 2015 to February 2018. Were recorded data regarding demographics, clinical variables, Paw (at admission and at day 4), high pressure ratio (HPR = number of days with high pressures: Peak ≥40 and/or plateau ≥30; and/or driving pressure ≥14; and/or auto-PEEP ≥6; divided by LOS), trends of Paw (Paw at day 4 - Paw at admission) and outcomes. The patients were divided into two groups: a construction group (n=200) and a validation group(n=104). The Paw-MPS was developed and validated by analyzing in a multivariate regression model the different Paw.

**Results:** The main characteristics of the construction population (n=200) were: mean age, 54±19 years; mean SAPSII, 32±13; pH, 7.3±0.1; pCO2, 49±21 mmHg; PaO2/FiO2, 211±106 mmHg; median MV duration, 6[3-13] days; LOS, 9[5-16] days and mortality, 104(52%). Paw were respectively for peak, plateau, driving, and auto-PEEP at admission: 32.1±9.5, 20.2±6, 13.1±5, 3[0-8] cmH2O and at day 4: 32.01±10, 20.7±7, 13.6±5 and 2[0-6] cmH2O. Median HPR was 0.13 [0-0.7]. Three independent mortality risk factors were identified: plateau at day 4 (OR, 1.1; 95%CI, [1.01-1.2]; p=0.027), delta Peak (OR, 1.086; 95%CI, [1.01-1.15]; p=0.011) and HPR (OR, 6.4; 95%CI, [1.77- 23]; p=0.005). Optimal cutoff points were selected on the ROC curves: plateau at day 4=18, delta Peak=2 and HPR=0.34. Were assigned respectively a point value of 1, 1, and 2 to these predictors based on their beta coefficient in the predictive model. The score yielded a ROC-AUC: (AUC=0.79; 95%CI, [0.72- 0.86]; p=0.000). Using the validation data set (n=104), the score had an ROC-AUC=0.8 and similar estimated probabilities for mortality.

**Conclusions:** The Paw-MPS seems to demonstrate interesting discriminative properties to predict mortality.

### P465 What is the role of the pulmonary embolism severity index (PESI) and RV/LV ratio as clinical risk assessment tools for patients undergoing ultrasound-assisted catheter-directed thrombolysis (UACDT)?

#### A Khan^1^, L Ramirez^2^, K Omonuwa^3^, J Arampulikan^4^

##### ^1^Lincoln Hospital, Internal Medicine, Bronx, United States; ^2^Lincoln Hospital, Bronx, United States; ^3^Lincoln Hospital, Pulmonary/Critical Care, Bronx, United States; ^4^Lincoln Hospital, Interventional Radiology, Bronx, United States

**Introduction:** To evaluate if the pulmonary embolism severity index (PESI) score correlates with RV/LV ratio, biomarkers of cardiac injury, fibrinogen and Length of stay(LOS). Also to evaluate the correlation between RV/LV ratio with biomarkers of cardiac injury, fibrinogen and LOS for patients who underwent UACDT.

**Methods:** A retrospective review of patients with sub-massive pulmonary embolism (PE) who underwent ultrasound-assisted catheter-directed thrombolysis (UACDT) was performed. PESI score, RV/LV ratio, length of stay(LOS), fibrinogen levels, troponin levesl, and brain natriuretic peptide(BNP) levels, were calculated and collected prior to UACDT. Spearman’s rank correlation coefficient was calculated for all non-parametric variables.

**Results:** 31 patients, 17 males and 14 females, were included in the study. The mean (±SD) age was 54±17 years. The mean PESI score was 93±38. Mean RV/LV ratio was 1.34±0.35. A significant correlation between the RV/LV ratio and both fibrinogen and troponin level (p=0.0013, p=0.0167) was noted. No significant correlation existed between PESI score and RV/LV (p=0.85). No significant correlation existed between both RV/LV ratio and PESI score with length of stay (p=0.1649) after UACDT. There were no noted mortality or complications.

**Conclusions:** PESI score is used as a prognostic factor for the patients with PE, however, our study shows that PESI score does not correlate with RV/LV ratio or length of stay after the UACDT. There was inverse correlation between RV/LV ratio and fibrinogen. There was also positive correlation between RV/LV ratio and troponin for patients with and without heart failure. According to our data, there may be limited use of PESI score and RV/LV ratio for risk stratification of PE patients undergoing UACDT.

### P466 Clinical outcome prediction after admission in intermediate level critical care unit: role of severity scores

#### G Nobre de Jesus, F Bessa, J Amorim, J Santos, C Candeias, S Fernandes

##### Centro Hospitalar Universitário Lisboa Norte, Serviço de Medicina Intensiva, Lisbon, Portugal

**Introduction:** Conventional scores for prediction of risk and outcome, such as SAPSII and SOFA, have not been validated for patients admitted to level II critical care units (intermediate level or ImCUs). We compared the performance of SAPSII and SOFA scores with the Intermediate Care Unit Severity Score (IMCUSS) in a general population admitted to ImCU.

**Methods:** We conducted a prospective observational cohort study in a 31-bed level II-III ICU from a university-affiliated hospital, during a three-month period. We applied SAPSII, SOFA day one and IMCUSS to all patients admitted during that period. Primary outcome was a composite of hospital mortality and need to increase level of care. Additionally, we tested the relevance of each variable within each score to predict the outcome.

**Results:** We included 108 patients with a mean age of 61.7±18.6 years. 63 patients were considered “step-down” (transferred from our level III beds), and the remaining originated from the emergency department (n=17), operating room (n=21) and surgical or medical wards (n=7). We recorded mean SAPSII=24.6±10.1, SOFA=3.0±2.3 and IMCUSS=19.6±11.1. During this period the primary outcome occurred in 19 patients (17.6%), but only two patients died during ImCU stay (ImCU mortality=1.9%). In single logistic regression, only IMCUSS was associated with the composite outcome (OR=1.05; p=0.016). The need for non-invasive ventilation at admission (OR=5.7; p=0.02) and immunosuppression (OR=3.6; p=0.04) were the only variables within the score able to correlate with the outcome (Table 1). Primary allocation of the patient and previous hospital length of stay did not affect the outcome.

**Conclusions:** In this cohort, the IMCUSS was the only severity score able to predict hospital mortality or need for increased level of care. We will extend the study in order to validate IMCUSS as recommended standard to establish the risk and severity of patients admitted to ImCUs.

**Table 1 (abstract P466).** Univariate logistic regression for independent variables included in the scores

Variables at admission	OR	95% CI	p value
Age	1.03	0.99-1.06	0.11
Previous hospital length of stay ≥ 7 days	2.20	0.79-5.99	0.13
Health-care related infection	2.09	0.6-6.80	0.22
Immunosuppression	3.60	1.03-12.7	0.04
GCS ≤ 12	0.89	0.73-1.07	0.22
Non-Invasive ventilation	5.70	1.28-25.16	0.02
Urea ≥ 60 mg/dL	0.99	0.98-1.01	0.79

### P467 Critically ill patients with cancer – trends and characteristics of admissions into a mixed ICU

#### I Furtado^1^, C Gonçalves^2^, A Bastos^2^, T Cardoso^2^, I Aragão^2^

##### ^1^Centro Hospitalar do Porto, Infecciologia, Porto, Portugal; ^2^Centro Hospitalar do Porto, UCIP, Porto, Portugal

**Introduction:** The increase in live expectancy of patients living with cancer along the progress in cancer therapy has led to a growing proportion of patients being admitted into intensive care (ICU). Objectives: To determine and characterize the proportion of patients with cancer admitted into a mixed ICU comparing the evolution between 2010 and 2017.

**Methods:** Retrospective cohort of all cancer patients admitted to a Portuguese mixed ICU of a tertiary care University hospital between January 2016 and December 2017. These results were compared with the ones obtained in a similar study conducted between January 2010 and October 2012.

**Results:** During the study period 847 patients were admitted into the ICU, of which 101 (12%) with cancer. Most were male (68%) and with a mean age of 65 years (±14). General characteristics of these patients are presented in Table 1. Mean SAPS II was 52 predicting a hospital mortality of 50%, that was approximately the same of observed mortality. Admissions related to cancer had a higher ICU mortality (53 vs 29%, p<0.05). Even though the Charlson index was lower in patients with hematologic cancer (5 vs 7), both ICU and hospital mortality were significantly higher in this group.

**Conclusions:** In our unit an increasing proportion of patients is being admitted reaching 12% in the last period. Cancer-related illness admissions and hematologic cancers had a significantly higher mortality. SAPS II was a good predictor of the mortality in this type of patients.

**Reference**

1. Predicted and observed outcome of cancer patients in intensive care unit and hospital P1034 26th ESICM ANNUAL CONGRESS, 2013

**Table 1 (abstract P467).** Comparison of two groups of patients with cancer admitted into a mixed ICU

	2016-2017	2010-2012
Number of patients with cancer/total admissions (%)	101/847 (12)	86 / 1129 (8)
Solid tumours, n (%)	77 (76)	62 (72)
Diagnosed in the previous year, n (%)	42 (42)	44 (51)
Cancer related illness admissions, n (%)	38 (38)	19 (22)
SAPS II ±SD	52 ±19	55 ±21
ICU mortality, n (%)	39 (39)	39 (45)
Hospital mortality, n (%)	49 (49)	46 (53)

### P468 Outcomes and quality of life of elderly ICU survivors

#### H Zorgati, I Ben Saida, D Ben braiek, S Ennoumi, N Fraj, Z Wafa, M Boujelbene, A Azouzi, M Boussarssar

##### Faculty of Medecine University of Sousse, Intensive Care Unit of Farhat Hached Teaching hospital, Sousse, Tunisia

**Introduction:** Given the ageing of the world´s population, the demands of critical care resources for elderly patients has increased during the past decade. However, little is known about quality of life and outcomes of elderly ICU survivors. The aim of the study is to assess outcomes of elderly ICU survivors at least 6 months after discharge: quality of life and mortality.

**Methods:** It is a retrospective study performed in a medical adult ICU between January 2016 to December 2017. The study included all elderly survivors ( ≥65 years) after ICU admission. Outcomes were assessed by telephone interviews at least 6 months after ICU discharge. The primary outcome was assessing the quality of life after ICU stay, measured by Euro Qol 5D questionnaire. The EQ-5D descriptive system contains five dimensions (mobility, self-care, usual activities, pain and discomfort, and anxiety and depression). For each dimension, there are five levels (no problems, slight problems, moderate problems, severe problems and unable to/extreme problems). The second outcome was mortality.

**Results:** During the study period, 486 patients were admitted and 178 (36.6%) were ≥ 65 years old. ICU mortality rate was 84 (47.2%). Ninety-four (52.8%) were discharged alive. Twenty-nine (30.8%) died after ICU discharge with mean delay 5.13 ± 4.61 months. Thirty eight (21.3%) elderly ICU survivors were contacted by phone, mean age was 74±6; male, 21(55.3%); median SAPS II 31.5[27.75-37]; median charlson index 4.5 [4.5, 25]; median length of ICU stay 6.5[4-10.5]; invasive mechanical ventilation 13(34,2%) and vasopressors use 11(28,9%). The Mean EQ-VAS was 72.76 ±24.34 and ranged from 5 to 100. Quality of life (EuroQoL 5D) scores are resumed in Figure 1.

**Conclusions:** Most elderly survivors patients showed a good health related quality of life using the EuroQoL 5D-5L after ICU discharge.

**Fig. 1 (abstract P468).**
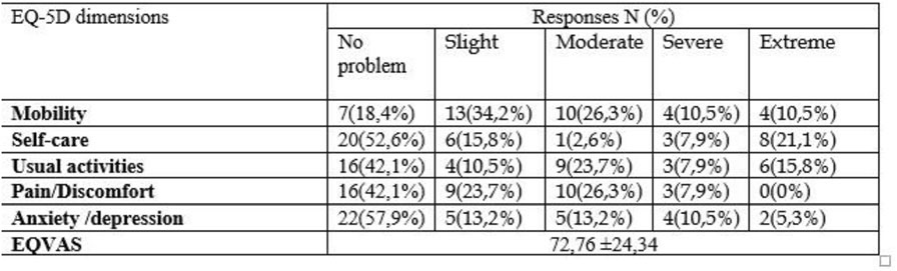
Quality of life (EuroQoL 5D) scores after ICU discharge

### P469 Five-year mortality and morbidity impact of prolonged ICU stay

#### N Van aerde^1^, G Hermans^2^, P Meersseman^3^, H Van Mechelen^4^, Y Debaveye^5^, A Wilmer^3^, J Gunst^5^, MP Casaer^5^, J Dubois^6^, PJ Wouters^5^, R Gosselink^7^, G Van den Berghe^5^

##### ^1^Laboratory of cellular and molecular medicine, Intensive care medicine, Leuven, Belgium; ^2^Laboratory of Cellular and Molecular Medicine, Medical Intensive Care Unit, Leuven, Belgium; ^3^University hospitals of Leuven, Medical Intensive Care Unit, Leuven, Belgium; ^4^Catholic University of Leuven, Catholic University of Leuven, Leuven, Belgium; ^5^University hospitals of Leuven, Intensive Care Medicine, Leuven, Belgium; ^6^Jessa Hospitals, Intensive Care Medicine, Hasselt, Belgium; ^7^Catholic University of Leuven, Rehabilitation sciences, Leuven, Belgium

**Introduction:** Long-term impact of critical illness may be affected by duration of critical illness and intensive care. We investigated differences in mortality and morbidity after short (<8 days) and prolonged (≥8 days) ICU-stay.

**Methods:** Prospective, 5-year follow-up study of former EPaNIC-patients (ClinicalTrials.gov:NCT00512122, N=4640). Mortality was assessed in all. For morbidity analyses, all long-stay and a random sample (30%) of short-stay survivors were contacted. Primary outcomes were total and post-28-day 5-year mortality. Secondary outcomes comprised handgrip strength (HGF,%pred), 6-minute-walk distance (6MWD,%pred) and SF-36 Physical Function (PF SF-36, 0-100). 1-to-1 propensity-score matching of short- and long-stay patients was performed for baseline factors, comorbidities and illness severity. Multivariable regression analyses were performed to identify ICU-factors explaining outcome differences.

**Results:** Total and post-28-day 5-year mortality was higher in long-stayers than in matched short-stayers (respectively 48 vs 36%, N=1928, P<0.001 and 41 vs 30%, N=1648, P<0.001). In multivariable cox regression analysis, ICU-risk factors comprised hypoglycaemia, corticosteroids, NMBA, benzodiazepines, mechanical ventilation, new dialysis, new infection, liver dysfunction, whereas clonidine may be protective. Among 276 long- and 398 short-stay 5-year survivors HGF, 6MWD and PF SF-36 were lower in long-stayers (matched set HGF: 83% (95%CI:60%-100%) vs 87% (95%CI:73%-103%), P=0.020; 6MWD: 85% (95%CI:69%-101%) vs 94% (95%CI:76%-105%) P=0.005; PF-SF36: 65 (95%CI:35-90) vs 75 (95%CI:55-90), P=0.002). Multivariable regression identified associations with benzodiazepines (HGF and PF-SF36), vasopressors (PF-SF36) and opioids (6MWD).

**Conclusions:** Prolonged ICU-stay is associated with excess 5 year mortality and morbidity, partially explained by potentially modifiable ICU-factors.

### P470 Predictive factors of post-traumatic stress disorder of a general intensive care unit survivors

#### L Pires^1^, M Santa Rita^2^, M Dykyy^2^, B Banheiro^2^, G Cabral Campello^2^

##### ^1^Centro Hospitalar Universitario do Algarve - Unidade de Portimao, Intensive Care, Portimao, Portugal; ^2^Centro Hospitalar Universitario do Algarve - Unidade de Portimao, Intensive care, PORTIMAO, Portugal

**Introduction:** ICU survivors suffer from post-traumatic stress disorder (PTSD). The reported risk factors related with the admission disease include higher severity, length of invasive mechanical ventilation (IMV), longer ICU stay and trauma patients.

**Methods:** This study aimed to identify predictive factors of PTSD, in patients who had been treated in the general ICU. Retrospective study of ICU survivors, aged 18 years or more, admitted in the ICU for at least 48 hours, from january 2017 to march 2018. PTSD related symptoms were accessed with the post traumatic stress syndrome 14 questions inventory (PTSS-14) at the post ICU follow up clinic, six months after the acute stress event. The post ICU consultation was carry out by an ICU doctor and an ICU nurse. Exclusion criteria: previous severe psiquiatric disorders, not able to respond the questionnaire, ICU stay less than 48 hours

**Results:** 211 survivors were invited to the follow-up clinic, 148 (70%) went to the consultation. 42 meet the exclusion criteria. We included in the study 106 patients: median age 66 years; 58 (55%) were men, the median ICU length of stay was 9,7 days; median SAPS II was 39; medical 67%, surgical 24% and trauma 9%. 60 patients (57%) were on IMV and the median ventilation days was 7. PTSD scores ranged from 14 to 70. Delusional memories were found in 22 patients (21%). Three (2.8 %) had a PTSS 14 score > 45: all had memories of the ICU stay and one had delusional memories. The median age of this subgroup was 69 years, 67% females, no IMV, the ICU length of stay was inferior (7 days) and the median SAPS II was also inferior (33).

**Conclusions:** In this study the rate of PTSD was lower 2.8% and related with a lower SAPS II and the presence of memories of the ICU stay. No relation was found with delusional memories, IMV or superior ICU length of stay. Patients with lower illness severity and without IMV, should be elective to the follow up-clinics.

### P471 Long-term effects of a sepsis aftercare intervention

#### K Schmidt^1^, D Schwarzkopf^2^, L Baldwin^3^, F Martin^4^, A Freytag^1^, C Heintze^5^, K Reinhart^6^, N Schneider^1^, S Worrack^1^, M Wensing^7^, J Gensichen^8^

##### ^1^Jena University Hospital, Institute of General Practice and Family Medicine, Jena, Germany; ^2^Jena University Hospital, Center for Sepsis Control and Care, Jena, Germany; ^3^University of Washington, Department of Family Medicine, Seattle, United States; ^4^Jena University Hospital, Center for Clinical Studies, Jena, Germany; ^5^Charité Universitätsmedizin, Institute of General Practice, Berlin, Germany; ^6^Jena University Hospital, Department of Anaesthesiology and Intensive Care Medicine, Jena, Germany; ^7^Heidelberg University Hospital, Department General Practice and Health Services Research, Heidelberg, Germany; ^8^University Hospital, LMU Munich, Institute of General Practice and Family Medicine, Munich, Germany

**Introduction:** Sepsis survivors face mental and physical sequelae even years after discharge from the intensive care unit (ICU). Effects of a primary care management intervention in sepsis aftercare were tested. Exploratory analyses suggest better functional outcomes within the intervention group compared to the control group at six and 12 months after ICU discharge. Longer term effects of the intervention have not been reported.

**Methods:** A randomized controlled trial was conducted, enrolling 291 patients who survived sepsis (including septic shock), recruited from nine German ICUs. Participants were randomized to usual care (n=143) or to a 12-months intervention (n=148). The intervention included training of patients and their primary care physicians (PCP) in evidence-based post-sepsis care, case management provided by trained nurses and clinical decision support for PCPs by consulting physicians. Usual care was provided by PCPs in the control group. The primary outcome of the trial was the change in mental health–related quality at 6-months after ICU discharge. Secondary outcomes included measures of mental and physical health. Data were collected by telephone interviews using validated questionnaires at the 24-months follow-up (12 months after the 1-year intervention).

**Results:** 186 [63.9%, 98 intervention, 88 control] of 291 patients completed the 24-months follow-up. Unlike the intervention group, the control group showed a significant increase of posttraumatic symptoms (diff. PTSS-10 to baseline, mean (SD) 3.7(11.8) control vs. -0.7(12.1) intervention; p=0.016). There were no significant differences in the MCS and all other secondary outcomes between intervention and control group.

**Conclusions:** 12 months after completion, the primary care management intervention had no effect on mental health–related quality of life and physical function among survivors of sepsis. Increase in PTSD symptoms in the control group may suggest a possible protective effect of the intervention.

### P472 Risk-factors for posttraumatic stress in sepsis survivors – a 24-month follow-up study

#### D Schwarzkopf^1^, J Gensichen^2^, G Schelling^3^, F Brunkhorst^4^, C Heintze^5^, K Reinhart^6^, M Vogel^7^, K Schmidt^7^

##### ^1^Jena University Hospital, Center for Sepsis Control and Care, Jena, Germany; ^2^University Hospital, LMU Munich, Institute of General Practice and Family Medicine, Munich, Germany; ^3^University Hospital, LMU Munich, Department of Anaesthesiology, Munich, Germany; ^4^Jena University Hospital, Center for Clinical Studies, Jena, Germany; ^5^Charité Universitätsmedizin, Institute of General Practice, Berlin, Germany; ^6^Jena University Hospital, Department of Anaesthesiology and Intensive Care Medicine, Jena, Germany; ^7^Jena University Hospital, Institute of General Practice and Family Medicine, Jena, Germany

**Introduction:** Survivors of sepsis often show symptoms of post-traumatic stress disorder (PTSD). Only few studies report on courses of more than 12 month after discharge from the ICU. The aim of this study was to identify predictors for changes in PTSD symptoms over time up to 24 month.

**Methods:** Follow-up data of the SMOOTH trial – a RCT to evaluate a primary care management intervention on sepsis survivors – were analyzed. Included patients were surveyed by phone for PTSD-symptoms at one, 6, 12 and 24 months after discharge from ICU using the Post-Traumatic-Stress-Scale (PTSS-10). Scores changes between follow-up periods were analyzed using latent-change scores in structural equation models. Predictors were clinical and socio-demographic baseline characteristics as well as physical, cognitive and functional sepsis sequelae assessed by validated questionnaires.

**Results:** 291 patients were included of which 186 participated in the 24 month follow-up. A decrease of PTSD symptoms between 6 and 12 months was predicted by higher education (B=-0.32, p=0.04), while higher pain intensity at one month predicted an increase (B=0.01, p=0.041). Increasing PTSD symptoms between 12 and 24 months were predicted by reporting more than two traumatic memories at one month (B=0.44, p=0.004), more sleep problems (B=0.22, p=0.08) and worse cognitive performance at 6 months (B=-0.03, p=0.043) as well as more neuropathic symptoms at 12 months (B=0.04, p=0.015).

**Conclusions:** Sepsis patients that suffer from physical, cognitive and functional impairments after ICU discharge may be at increased risk for developing late-onset PTSD. These predictors need to be replicated by future studies.

### P473 Early versus late readmission to the intensive care unit: a ten-year retrospective study

#### V Karamouzos^1^, N Ntoulias^2^, D Aretha^3^, A Solomou^3^, C Sklavou^3^, D Logothetis^3^, T Vrettos^3^, M Papadimitriou- Olivgieris^4^, D Velissaris^5^, F Fligou^3^

##### ^1^Patras General University Hospital, Intensive Care Unit, Patras, Greece; ^2^University of Patras, University of Patras, Patras, Greece; ^3^Patras General University Hospital, Intensive Care Unit, Patras, Greece; ^4^Patras General University Hospital, Division of Infectious diseases, Patras, Greece; ^5^Patras General University Hospital, Internal medicine department, Patras, Greece

**Introduction:** Intensive care unit (ICU) readmissions vary between 7%-8% and are related to higher length of stay and mortality. The purpose of this study was to compare early versus late ICU readmission, in a Mediterranean university hospital lacking a high dependency or a step-down unit.

**Methods:** In this single center, retrospective study, we searched our patient database for readmitted patients in our unit from 01/03/2008 to 31/02/2018. Out of a total of 3250 patients, 173 (5.32%) were readmitted within 60 days. 29 patients were excluded due to a planned readmission. Epidemiological data, admission type, Apache II score, length of stay, and 28-day survival were recorded for 144 patients.

**Results:** 60 (41.6%) patients were readmitted within 72 hours and 84 (58.4%) in 4 to 60 days. The two groups didn’t differ in age, gender, Charlson comorbidity index and length of stay on both admissions. Elective surgery was the most common type of admission (34.7%) followed by medical (28.4%), emergency surgery (27%) and trauma (9.7%). The mean time to readmission in the late group was 17.9 (±17.8) days. Patients in the late group had higher APACHE II score on their first and second admission, (17.3±5.5 vs 14.2±6.1; p=0.008) and (18.7±6.9 vs 14.4±6.7; p=0.002) respectively. Respiratory insufficiency was the most common cause of readmission in both groups followed by sepsis and cardiac arrest. Finally in the early group, fewer patients had an artificial airway (33.3% vs 58.3%; p=0.003) and the mortality was significantly lower (30% vs 51.2%; p=0.011) (Table 1).

**Conclusions:** Patients readmitted late in the ICU, had higher APACHE II score, were more likely to have an artificial airway and their mortality was higher in comparison to those admitted early. This group of severely ill patients is discharged from an ICU to a hospital ward with limited resources and non-specialized personnel. Step down units may be the key to facilitate an easier transition from a high care environment, reduce readmission rates and mortality.

**Table 1 (abstract P473).** See text for description

	3 days	4-60 days	p
Patients readmitted (%)	60 (41,6)	84 (58,4)	
Time to readmission (sd)	1.38 (±1)	17.9 (±17.8)	
Length of stay (sd)	13.5 (±16)	20,79 (±21)	ns
APACHE ΙΙ (sd)	14.4 (±6.7)	18.7 (±6.9)	0.002
Artificial airway (%)	20 (33.3)	49 (58,3)	0.003
Mortality (%)	18 (30)	43 (51,2)	0.011

sd: standard deviation, APACHE: acute physiology and chronic health evaluation

### P474 Readability and complexity of ICU-related online resources

#### L Martinek, S Paulasir, C Parsons, A Fleishman, A Gupta

##### Beth Israel Deaconess Medical Center/Harvard Medical School, Acute Care Surgery, Trauma and Surgical Critical Care, Boston, United States

**Introduction:** Critically ill patients and their families are often confronted with an overwhelming amount of clinical information shortly after hospital admission. Their reliance on Internet resources for additional information is increasing, particularly for unfamiliar medical terminology. Yet, little is known about whether these online resources meet the recommended reading level and complexity appropriate for the average reader.

**Methods:** An online search of 40 websites containing four common critical care diagnoses in the ICU (respiratory failure, renal failure, sepsis and delirium) was performed. A total of 6 readability formulas were used. The Flesch-Kincaid grade reading level (GRL) and Flesch reading ease (FRE) were used in the final analysis. Document complexity was evaluated using the PMOSE/iKIRSCH formula.

**Results:** Websites on respiratory failure were written at the 13th GRL with FRE of 29.6. Renal failure resources had a 9th GRL with FRE of 54.7. Sepsis websites had an 8th GRL with FRE of 48.3. Delirium websites had a 12th GRL with FRE of 31.6. When comparing website types (government, non-profit and private), ANOVA showed a difference in FRE across all 3 groups and government websites had a significantly lower Flesch-Kincaid grade level compared to non-profit and private sites. 82.5% of all websites had a very low document complexity score.

**Conclusions:** Online resources used by intensive care unit patients and families tend to be written at higher than the recommended 6th GRL, with government sites better meeting this target than non-profit and private organizations. Online resources should be improved to lower this unfortunate barrier to patient education.

### P475 Incapacity and disclosures to the police: the law post GDPR

#### A D´Sa

##### Addenbrookes Hospital, Neuro Critical Care, Cambridge, United Kingdom

**Introduction:** The recent enactment of the Data Protection Act 2018, the General Data Protection Regulations, and a series of data breaches in the healthcare sector, have renewed interest in how our patients’ information is collected, used and shared. The complex framework of laws and regulations governing the use and disclosure of personal data may lead to professional and financial consequences if information is disclosed inappropriately. Disclosures to the police when they concern incapacitous patients are particularly challenging, as the disclosure may have no direct benefit to the patient and may cause the patient considerable harm.

**Methods:** We have reviewed the relevant laws and regulations to identify the circumstances in which doctors must release information regarding incapacitous patients to the police. The laws and regulations are examined to identify the extent of the disclosure required, and any requirements for the disclosure to be lawful. We have also identified laws which confer a power to disclose information about incapacitous patients, and the circumstances in which these powers can be used.

**Results:** In conjunction with a local police constabulary we have developed an information request form which makes it easier for those requesting and disclosing information to understand the legal basis of the disclosure. We have also developed guidelines to allow practitioners to understand where a disclosure is obligatory or discretionary.

**Conclusions:** The next stage of the project is to audit disclosures of information in the intensive care unit, and identify whether information is being released lawfully and following the correct procedure.

**Reference**

Regulation (EU) 2016/679 of the European Parliament and of the Council of 27 April 2016 on the protection of natural persons with regard to the processing of personal data and on the free movement of such data

### P476 Families of patients in ICU: A scoping review of their needs and satisfaction with care

#### P Scott^1^, A Shepherd^2^, P Thomson^2^

##### ^1^Forth Valley Royal Hospital, Intensive Care Unit, Larbert, United Kingdom; ^2^University of Stirling, Faculty of Health Sciences and Sport, Stirling, United Kingdom

**Introduction:** Family members are affected both physically and psychologically when their relative is admitted to ICU. There is limited knowledge describing their experiences and structured interventions that might support them during their relative’s critical illness. The aim of this review is to describe published literature on the needs and experiences of relatives of adult critically ill patients and interventions to improve family satisfaction and psychological well-being.

**Methods:** Design: Scoping review. Standardised processes of study identification, data extraction on study design, sample size, sample characteristics and outcomes measured (Figure 1).

**Results:** From 469 references, 43 studies were identified for inclusion

Four key themes were identified:

1. Different perspectives on meeting family needs
2. Family satisfaction with ICU care
3. Factors impacting on family health and well-being and capacity to cope
4. Psychosocial interventions

**Conclusions:** Family members of patients in ICU experience unmet information and assurance needs which impacts on their physical and mental health. Structured written as well as oral information show some effect in improving satisfaction and reducing psychological burden. ICU’s who are able to support interventions based on meeting family information needs, in addition to reducing psychological burden and increasing satisfaction will enable each family to provide more support to their relative within the ICU.

**Fig. 1 (abstract P476).**
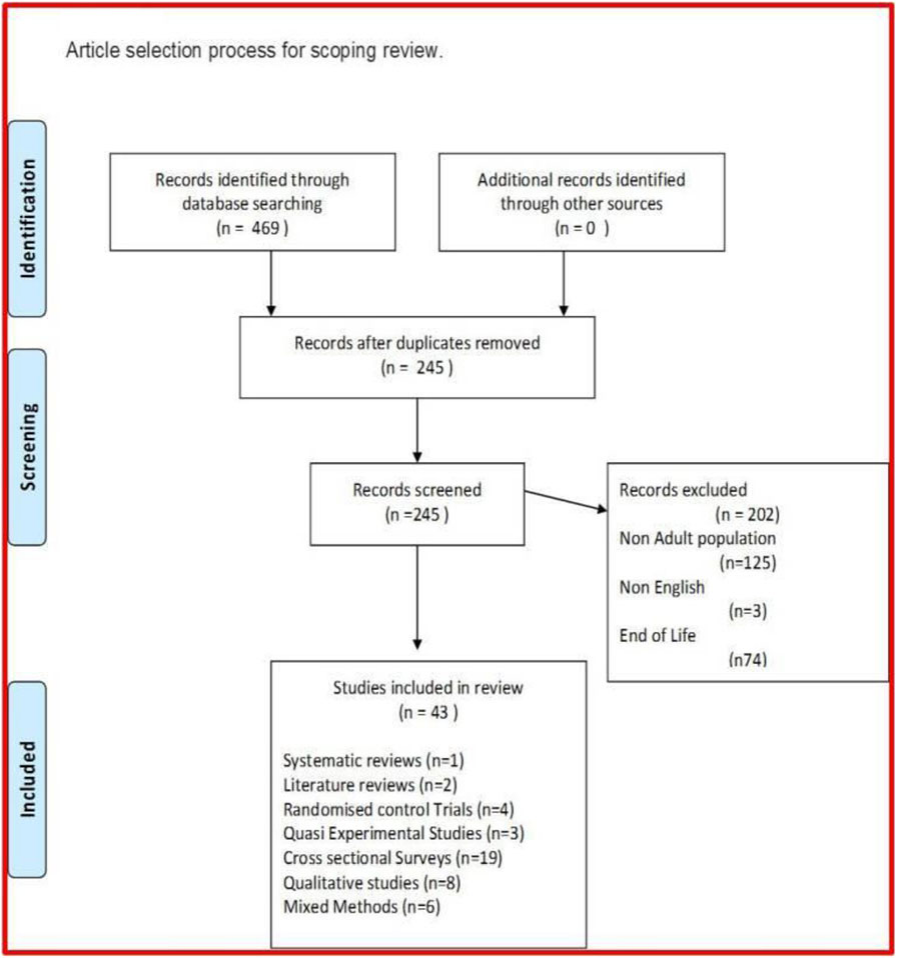
Article selection process for scoping review

### P477 Effectiveness of a structured communication strategy on satisfaction, anxiety and uncertainty in family members of the critically ill

#### P Scott^1^, P Thomson^2^, A Shepherd^2^

##### ^1^Forth Valley Royal Hospital, Intensive Care unit, Larbert, United Kingdom; ^2^University of Stirling, Faculty of Health Sciences and Sport, Stirling, United Kingdom

**Introduction:** Unmet informational needs lead to dissatisfaction with care and psychological distress. Identifying interventions to help meet specific needs is a crucial and necessary step in providing family centred care in ICU. We aimed to implement and evaluate the impact of delivering a structured communication strategy on levels of anxiety, uncertainty and satisfaction with care and decision making in families of critically ill adults.

**Methods:** A quasi experimental study with pre and post test design. A convenience sample of 52 family members were recruited from July 2016 to February 2018. The intervention group (n=26) received both oral and printed information to guide them in preparing for a structured family meeting. The control group (n=26) received usual routine care and existing family informational support. Anxiety, uncertainty and family satisfaction were measured in the two groups on ICU admission and ICU discharge.

**Results:** Mean anxiety, uncertainty and satisfaction with care and decision making scores pre and post intervention were compared. There were no significant differences in mean anxiety, uncertainty or satisfaction scores between the two groups before the intervention (p>0.05). Mean scores on anxiety (45.5vs 46.9), and uncertainty (72.4 vs 76.7) were lower post intervention, but not significantly so (Figure 1&2). Total satisfaction, satisfaction with care and satisfaction with decision making mean scores were similar in both groups before and after the intervention (p.0.05).

**Conclusions:** Providing relatives with a combination of targeted written and oral information delivered by nursing and medical staff reduced anxiety and uncertainty with this reduction being evident through to discharge from ICU. Although not statistically significant, there was what may be seen as a suggestion of a clinically significant drop in anxiety and uncertainty following the intervention

**Reference**

Nelson J (2009) Family meeting made simpler: A toolkit for ICU. Journal of Critical Care. 24 (4)

**Fig. 1 (abstract P477).**
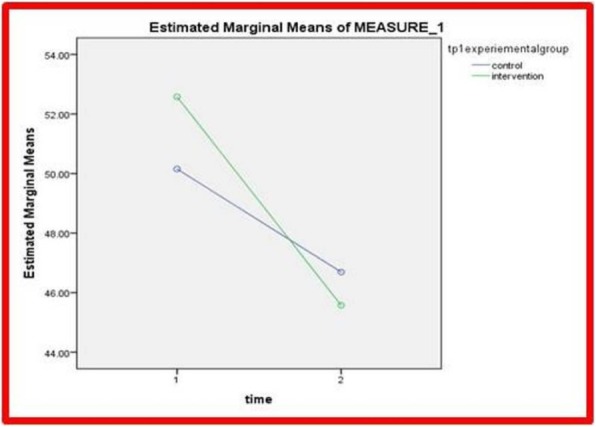
State anxiety scores on ICU admission and on ICU discharge

**Fig. 2 (abstract P477).**
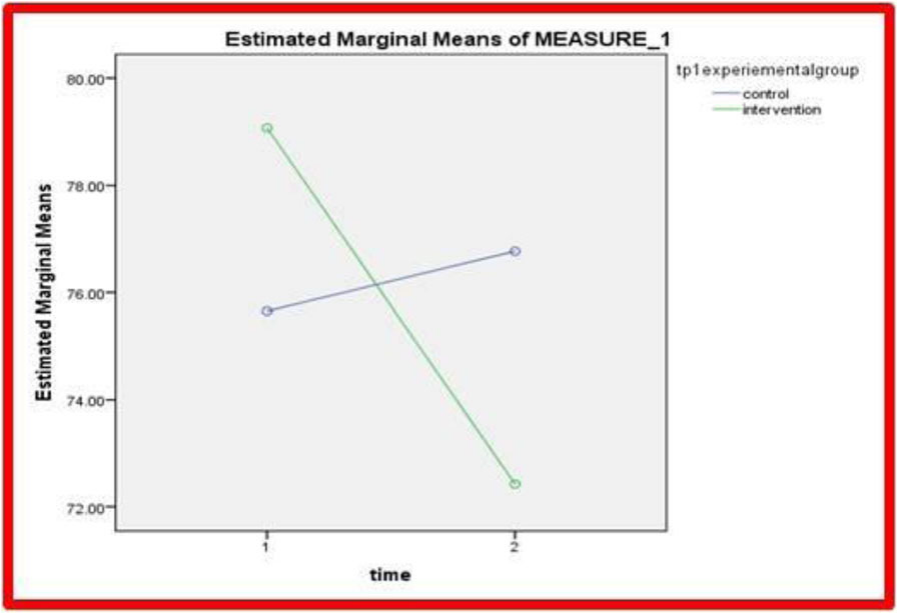
Uncertainty in illness scores on ICU admission and on ICU discharge

### P478 Barriers and challenges in the process of achieving informed consent from intensive care unit patients

#### J Dahlberg, A Robertsen, S Beitland

##### University of Oslo, Medical faculty, Oslo, Norway

**Introduction:** Clinical studies in intensive care unit (ICU) patients are warranted in order to improve healthcare. The aim of this study was to analyse barriers and challenges in the process of achieving informed consent from ICU patients.

**Methods:** We analysed patients considered for inclusion in a prospective observational study of venous thromboembolism in the ICU, i.e. the Norwegian Intensive Care Unit Dalteparin Effect (NORIDES) study. Data were collected from the screening log, consent forms and associated research notes of the NORIDES study.

**Results:** We observed that 58 of 279 (20%) eligible patients according to inclusion and exclusion criteria were omitted from the NORIDES study due to barriers and challenges in the process of receiving informed consent. 21 were categorized as psychiatric diseases consisting of known psychosis or recent suicide attempt, 19 likely or actual treatment withdrawals and 18 due to language barriers among non-Norwegians. Among the 70 patients included in the NORIDES study, 29 (41%) consents were from patients and 41 (59%) obtained from their next of kind. From the patient consents, 11 (38%) consents were oral and 18 (62%) were written. 6 patients were physically unable to sign, and 7 patients did not recognize their own signature. The study further pointed at some specific challenges in the process of consent, herein questionable competence to give consent, failure to remember being asked/included, inability to separate research from treatment etc. There were also difficulties in evaluating who was next of kin and how to reach them.

**Conclusions:** Barriers and challenges in obtaining informed consent from ICU patients led to exclusion of one fifth of the eligible patients in our study. Informed consent directly from patients was obtained from less than half of the included patients. Obstacles in the process of achieving informed consent were practical, medical, ethical and/or legal.

### P479 Determinants of end-of-life decision-making in the intensive care unit

#### P Eiben, C Brathwaite-Shirley, S Canestrini

##### King´s College Hospital NHS Foundation Trust, London, United Kingdom

**Introduction:** Although the majority of intensive care unit (ICU) deaths follow the decision to forgo Life Sustaining Treatment (LST), variability in patterns is commonly observed [1,2]. We reviewed End of Life (EoL) practice at our institution in order to explore: (i) patient characteristics affecting EoL decision-making, (ii) communication among surrogate decision-makers, and (iii) EoL management.

**Methods:** We retrospectively analyzed data from consecutive patients who died in our ten-bed ICU over 16 months (study period). Patient demographics, APACHE II, functional status, diagnosis on admission, ICU length of stay (LOS) were collected; family/next-of-kin (NOK) involvement and rationale for LST limitation were recorded (Table 1). Data are shown as mean ± SD/median (IQR). T-test and Mann-Whitney Test were used to compare means and medians respectively.

**Results:** 157 patients were included. 87 (55.4%) underwent Withdrawal of Life Sustaining Treatment (WLST) (W-Group, Table 1), 70 (44.6%) did not (NW-Group). W-Group and NW-Group had similar APACHE II (23.2 ± 9.9 v 24.4 ± 6.3; P=0.39), age (70.3 ± 11.8 v 70.2 ± 12.6; P=0.96) and functional status [10.5 (4-47) v 8.5 (4-52), P=0.886] (Table 2). W-Group had higher ICU LOS [4 (2-7) v 2 (1-5); P<0.001]. Family/NOK involvement was observed in 89% of cases. Rates of documentation for Do Not Resuscitate (DNR) order, reason for WLST and consensus were 83.6%, 87.7% and 91.8% respectively. Refractory multiorgan failure was the most common reason for WLST (61.6%). 35.6% of patients were referred to the organ donation service as part of EoL management. 89% died in ICU.

**Conclusions:** Our analysis shows that in our institution EoL deliberations follow a shared decision-making process. Lack of family/NOK involvement and incomplete documentation was exceptional. The significant difference in LOS between W-Group and NW-Group, in the face of similar APACHE II, warrants further investigation.

**References**

1. Sprung CL et al. JAMA 290(6):790-797, 2003

2. https://www.icnarc.org

**Table 1 (abstract P479).** Characteristics of the W-Group

Variable	W-Group (n=73)
Age (years, mean ± SD)	70.3 ± 11.8
Female sex, n (%)	34 (46.6%)
Diagnosis on admission: medical, n (%)	65 (89.0%)
Inotropes/vasopressors at time of WLST, n (%)	49 (67.1%)
RRT at time of WLST, n (%)	28 (38.4%)
DNR order, n (%)	61 (83.6%)
Organ donation services involved, n (%)	26 (35.6%)

**Table 2 (abstract P479).** Functional status on admission. ADL – activities of daily living

Abbreviation	Definition
A	No assistance with ADL
B	Minor assistance with ADL
C	Major assistance with ADL
D	Total assistance with ADL

### P480 Medical and nurses´ teams´ perception of inappropriate treatments in intermediate care units patients with order of life-support limitation

#### M Nguyen tan^1^, N Van Grunderbeeck^1^, F Lambiotte^2^, H Georges^3^, M Granier^4^, A Moreau^5^, E Parmentier^5^, P Molmy^1^, R Krouchi^6^, M Nyunga^7^, O Nigeon^1^, D Thévenin^1^

##### ^1^CH Lens, Réanimation polyvalente/USC, Lens, France; ^2^CH Valenciennes, Réanimation Polyvalente/USC, Valenciennes, France; ^3^CH Dron, Réanimation/USC et Maladies Infectieuses, Tourcoing, France; ^4^CH Arras, Réanimation Polyvalente/USC, Arras, France; ^5^CHRU Lille, Réanimation Médicale/USC, Lille, France; ^6^CH Dunkerque, Réanimation Polyvalente/USC, Dunkerque, France; ^7^CH Victor Provo, Réanimation Médicale, Roubaix, France

**Introduction:** In Intensive Care Units, perceived Inappropriate Treatments (PIT) have been associated with negative impact on caregivers, such as burn-out [1]. Intermediate Care Units (IctdCU) are hardly studied. Our study aimed to assess relationship between PIT of patients with life-supporting limitations, and burn-out or intention to leave of caregivers in this setting.

**Methods:** Observational, multicentric retrospective study in 7 ItdCUs of public hospitals in the North of France from December 2017 to April 2018. An anonymous questionnaire of 28 items assessed working environment, burn-out, PIT, and intention to leave in senior and junior physicians, nurses and caregivers. Univariate and logistic regression analysis were performed.

**Results:** A hundred and ninety-seven questionnaires were analyzed (participation rate 41.6%). Univariate analysis revealed that burn-out, PIT and intention to leave were greater in units where nurses´ teams included no activity in the ICU, compared to “shared” work in ICU and IdtCU. In multivariate analysis, perception of non beneficial treatment of patients with life support witholding was associated with: bad collaboration with other units (Odds Ratio 1.29; IC 95% [1.10 - 1.52]); burn-out state (Odds Ratio 1.40; IC95% [1.22-1.60]). Favourable work relationship (between physicians and nurses´teams) were associated with a lower perception of disproportionate treatments (Odds Ratio 0.86; IC95% [0.75 - 0.98]). Senior physicians were more satisfied of the working atmosphere, and perceived less inappropriate treatments.

**Conclusions:** PIT in IdtCU is linked to work ambience and burn-out. Limits include study of patients´ patterns and deeper study of interplay between PIT and burn-out. Multidisciplinary teamwork and units´ interplay seem mandatory to improve practice.

**Reference**

1. Schwarzkopf D, et al. Crit Care Med 2017;45:e265-73.

### P481 Profile of intensive care unit (ICU) patients on whom life-sustaining medical treatment were withdrawn or withheld

#### S Chatterjee^1^, M Bhattacharya^1^, S Sinha^1^, A Roy Choudhury^2^, T Chatterjee^2^, S Todi^1^

##### ^1^AMRI Hospitals, Critical Care, Kolkata, India; ^2^Jadavpore University, Clinical Research, Kolkata, India

**Introduction:** The study compared clinical profile of patients whose life-sustaining medical treatment was withdrawn or withheld(WDWH-group) with those on active treatment(active- treatment group).

**Methods:** Case-control study of patients admitted to a tertiary care ICU in India between August-2014 to April-2016. Subjects in WDWH group defined as cases; for every case three patients from active-treatment group randomly selected as controls. Patients discharged against medical advice excluded. Variables collected-age, sex, APACHE IV score, diagnostic-category and co-morbidities. Primary outcomes were ICU and hospital mortality. Secondary outcomes included ICU and hospital length of stay(LOS). Outcomes compared between cases and controls. All analyses were adjusted for age, sex, co-morbidities and disease severity.

**Results:** There were 49 cases and 150 controls. Mean age significantly differed between cases and controls(77.8 ±11.7vs.67.3±16.6years; p=0.009). Significant difference was noted in mean APACHE IV score (74.9±26.1 for cases vs.55±27.9 for controls; p<0.0001). Malignant diseases and chronic renal failure were more prevalent among cases compared to controls(16.3%vs.5.3%, p=0.04; 24.5%vs.10%, p=0.01 respectively). ICU and hospital mortality was also significantly higher among cases compared to controls(ICU mortality-63.3% vs.9.3%, p<0.0001 and hospital mortality-66.7% vs.10.9%, p<0.0001 respectively). While median ICU LOS differed significantly between groups (cases-4 days (IQR 2-5) vs. controls-3days (IQR 2-4), p=004) hospital LOS was similar (cases-6 days (IQR 4-8) vs. controls-6 days (IQR 3-10), p=0.7). Adjusted analysis showed cases significantly more likely to die in ICU compared to controls (OR 15.3, 95% CI 6.9-20.9; p<0.0001).

**Conclusions:** ICU patients whose life-sustaining treatment was withdrawn or withheld had higher illness-severity scores, were older, had longer ICU LOS and higher mortality than those in active-treatment group.

### P482 Healthcare providers needs and coping strategies regarding end of life care of critically ill patients

#### R Castro^1^, K Villarroel^2^, M San Martin^2^, V Oviedo^1^, M Amthauer^1^, P Perez^3^, J Espinoza^4^, N Navarro^4^, M Gonzalez^5^, M Vera^1^, C Daniels^6^, M Bernales^2^, P Repetto^2^

##### ^1^Pontificia Universidad Catolica de Chile, Medicina Intensiva, Santiago centro, Chile; ^2^Pontificia Universidad Catolica de Chile, Psicología, Santiago centro, Chile; ^3^Pontificia Universidad Catolica de Chile, Medicina Paliativa y Cuidados Continuos, Santiago centro, Chile; ^4^Pontificia Universidad Catolica de Chile, Unidad de Paciente Critico. Hospital Clinico UC CHRISTUS, Santiago centro, Chile; ^5^Pontificia Universidad Catolica de Chile, Psiquiatría, Santiago centro, Chile; ^6^Pontificia Universidad Catolica de Chile, Santiago centro, Chile

**Introduction:** Caring for the critically ill patient is a complex task and becomes tougher when a death process takes place. A number of needs and coping strategies emerge from the healthcare providers before these issues but are mostly displayed out of individual skills and intuition. If those approaches are unappropriate and the needs are not met, patients’ death process may be burdensome for caregivers. This could affect the quality of care for patients and families during the whole end-of-life care process. The aim of our study was to explore the different needs and coping strategies used by ICU healthcare providers when facing patients in the dying process.

**Methods:** Qualitative and collective case study. Ten semi-structured interviews were conducted in ICU personnel (3 physicians and 7 nursing professionals). A thematic analysis was done using NVivo 11 software. Local Ethics Committee approved the study.

**Results:** Respondents were 70% women, had 36.7 ± 8.2 years-old and 11.1 ± 8.2 years of ICU experience. Main needs identified in ICU healthcare providers refer to a lack of tools for doing emotional containment when delivering bad news to families, handling personal mourning, the need to perceive consistency regarding end-of-life care management across the ICU team, and a wish of having regular training from a psychologist. Main identified coping strategies included closing rituals, finding quiet spaces to spend time, and asking for counselling with more expert colleagues. A need for systematic, although basic training on these issues from qualified professionals is demanded.

**Conclusions:** Usually, basic needs from patients and families in the process of dying are well addressed, but healthcare providers’ needs are underrecognized and coping strategies mostly unknown. Visibilization of those needs and basic but formal training in emotional containment, self-care and coping strategies are greatly desired.

**Acknowledgement**

Funded by VRI-Pastoral UC 2017.

### P483 Frequence and factors associated with the want to share in end of life decisions among ICU patients relatives

#### V Martinez, K Goinheix, E Moreira, A Carámbula, M Barbato, G Burghi

##### Hospital Maciel, Intensive Care Unit, Montevideo, Uruguay

**Introduction:** In the intensive care unit (ICU), patients often exhibit cognitive impairments that prevent them from participating in decisions related to therapeutic options at the end of life. Consequently, their families are often asked to speak for them when difficult decisions must be made. The main of this study was to determine the frequence in wich family want to share in end of life decisions and factors associated with this desire.

**Methods:** A prospective study was conducted in one mixed ICU in Montevideo. Relatives of Patients were invited to participate in this study after 72 hours in the ICU and completed a survey that included the Hospital Anxiety and Depression Scale.

**Results:** We analized 135 relatives from 96 patients hospitalized in the intensive care unit. The relationship with the patient was as follows: 23% spouses, 23% siblings, 21% grown children, 14% parents, and 19% other family members and friends. Of them, 41.5% reported a desire to share in end of life decisions. Anxiety and depression symtoms were present in 60% and 41% respectively. Factors asociated with the desire of involvment in end of life decisions by bivariate analysis were: female sex (48% vs 29%, p=0.03), presence of anxiety (51% vs 28%, p=0.008) and patient ECOG 2-4 (70% vs 34%, p=0.05). Multivariate analysis shows that the presence of anxiety is the only independent factor associated with the desire to participate in end of life decisions (OR 2.20, IC 95% 1.02-4.73; p=0.04).

**Conclusions:** Have a loved one in ICU is often associated with Anxiety and Depression after 72 hours of admission. Only 41% of the relatives want to participate in end of life decisions. The presence of anxiety is independently associated with the want to share in decisions making process.

### P484 Female sex, increased age and severity of clinical condition are associated with decisions to withdraw or withhold intensive care in Swedish intensive care units

#### L Block^1^, M Petzold^2^, S Naredi^3^

##### ^1^The Institute of Clinical Sciences, Sahlgrenska Academy, University of Gothenburg, Gothenburg, Sweden; ^2^University of Gothenburg, Dept Public Health and Community Medicine, Sahlgrenska Academy, Gothenburg, Sweden; ^3^The Institute of Clinical Sciences, Sahlgrenska Academy, Inst Clinical Sciences, Sahlgrenska Academy, University of Gothenburg, Gothenburg, Sweden

**Introduction:** Intensive care aims to treat failure of vital organ systems. Sometimes, a patient’s condition is of such a degree that intensive care is no longer beneficial, and decisions to withdraw or withhold intensive care are made. This means that life-sustaining treatments are terminated or not initiated. We aimed to identify variables that are independent factors for the decision to withdraw or withhold intensive care.

**Methods:** Registry study using extracted data from a national quality registry the Swedish Intensive Care Registry (SIR) 2014-2016. Data are delivered to the registry by nurses and doctors daily, during each patients’ stay in the intensive care unit (ICU). A total of 97,095 intensive care cases reported to the SIR from 2014-2016.

**Results:** Data regarding each patient´s age, sex, diagnoses, condition at admission (expressed as Simplified Acute Physiology Score version 3, SAPS 3), comorbidities and registered decisions to withdraw or withhold intensive care were analyzed. Of the 97,095 cases reported, 41.9% were women and 58.1% men, and 47.1% were 61–80 years old. A total of 15.4% received a decision to withdraw or withhold intensive care, accounting for 16.2% of all women and 14.8 % of all men, p<0.001. Independent variables associated with increased odds of receiving a decision to withdraw or withhold intensive care were older age, worse condition at admission, and female sex. Female sex was associated with an increased odds of receiving a decision to withdraw or withhold intensive care by 18% (CI 13.4–23.0%) after adjustments for condition at admission and age.

**Conclusions:** Older age, worse condition at admission and female sex was found to be independent variables associated with an increased odds to receive a decision to withdraw or withhold intensive care.

